# Targeting the CD4^+^ effector memory T cells re-expressing CD45RA suppressor galectin-7: a multi-omics-guided therapeutic strategy for idiopathic pulmonary fibrosis

**DOI:** 10.1186/s43556-026-00542-0

**Published:** 2026-10-05

**Authors:** Tao Yan, Jichang Liu, Yong Liu, Wenchao Gu, Jia Liu, Yuanshu Li, Rongrong Lv, Chuanpeng Zhang, Jiaqi Ding, Chenlong Zha, Chaoqun Wang, Fei Gao, Man Huang, Jingyu Chen

**Affiliations:** 1 Department of Lung Transplantation, The Second Affiliated Hospital Zhejiang University School of Medicine, Hangzhou, 310000 China; 2 Lung Transplantation Center, The Affiliated Wuxi People’s Hospital of Nanjing Medical University, Wuxi People’s Hospital, Wuxi Medical Center, Nanjing Medical University, Wuxi, 214023 China; 3 Department of Artificial Intelligence Medicine, Graduate School of Medicine, Chiba University, Chiba, Japan; 4 Zhongshan Institute for Drug Discovery, Shanghai Institute of Materia Medica, Chinese Academy of Sciences (CAS), Zhongshan, 528400 China; 5 Shanghai Institute of Materia Medica, Chinese Academy of Sciences, Shanghai, 201203 China; 6 State Key Laboratory of Drug Research, Shanghai Institute of Materia Medica, Chinese Academy of Sciences, Shanghai, 201203 China; 7 University of Chinese Academy of Sciences, Beijing, 100049 China; 8 Emergency Department, The Affiliated Wuxi People’s Hospital of Nanjing Medical University, Wuxi People’s Hospital, Wuxi Medical Center, Nanjing Medical University, Wuxi, 214023 China; 9 Department of General Intensive Care Unit, The Second Affiliated Hospital of Zhejiang University School of Medicine, Hangzhou, 310052 China; 10 Key Laboratory of Multiple Organ Failure(Zhejiang University), Ministry of Education, Zhejiang, China; 11 Jingyu Chen Lung Transplantation Laboratory, The Second Affiliated Hospital Zhejiang University School of Medicine, Hangzhou, 310000 China; 12 Department of Thoracic Surgery, Affiliated Hospital of Qingdao University, Qingdao, China

**Keywords:** Mendelian Randomization, Idiopathic Pulmonary Fibrosis, CD4^+^ Effector Memory T Cells Re-expressing CD45RA, Metaplastic KRT5^+^ Basal Cell, Galectin-7

## Abstract

Idiopathic pulmonary fibrosis (IPF) is a chronic, progressive interstitial lung disease with limited therapeutic options. Although single-cell and spatial transcriptomics have revealed cellular heterogeneity within the IPF lung microenvironment, the immune drivers with causal relevance remain poorly defined. Here, through large-scale Mendelian randomization integrating multi-omics data, we identified a consistent negative causal association between the proportion of CD4⁺ effector memory T cells re-expressing CD45RA (TEMRA) and IPF risk. Multi-platform validation—including single-cell RNA sequencing, flow cytometry, and multiplex immunofluorescence—confirmed a significant reduction in CD4⁺ TEMRA frequency in both peripheral blood and lung tissues of IPF patients, establishing this subset as a clinically relevant biomarker. Spatial transcriptomics further revealed a negative correlation between TEMRA differentiation signatures and local fibrotic burden. Mechanistically, integrated single-cell analyses pinpointed metaplastic KRT5⁺ basal cells as a key epithelial population with the capacity to disrupt immune homeostasis. These aberrant epithelial cells upregulate Galectin-7 (*LGALS7*), which we demonstrate competes with IL-7 for receptor binding, suppresses *STAT5* phosphorylation, and limits CD4⁺ TEMRA differentiation, thereby uncovering a previously unrecognized epithelial–immune crosstalk axis in IPF. Guided by these findings, virtual screening of 20,000 compounds identified neoeriocitrin as a Galectin-7 inhibitor that restores *STAT5* phosphorylation in vitro and attenuates pulmonary fibrosis in vivo. This study positions Galectin-7 as a druggable target and establishes a novel framework linking epithelial dysfunction to immune dysregulation in IPF.

**Supplementary Information:**

The online version contains supplementary material available at https://doi.org/10.1186/s43556-026-00542-0.

## Introduction

Idiopathic pulmonary fibrosis (IPF) is a chronic, progressive fibrotic interstitial lung disease characterized by repetitive alveolar epithelial injury, aberrant myofibroblast activation, and excessive extracellular matrix deposition, with fibroblastic foci destroying normal lung architecture [1–3]. Pathogenesis involves TGF-β/Smad, Wnt/β-catenin, LPA-autotaxin, and impaired PGE₂ signaling [1, 4]. Global age-standardized prevalence is 10–60 per 100,000, predominantly affecting older adults (> 60 years) [5]. While pirfenidone and nintedanib slow disease progression, they do not halt fibrosis. Nerandomilast, a selective PDE4B inhibitor, shows promise in phase II/III trials but remains investigational [6]. Nevertheless, IPF remains incurable, underscoring the need to delineate its pathogenic mechanisms and identify new therapeutic targets.

Unquestionably, immune dysregulation is a key feature of idiopathic pulmonary fibrosis [7–9]. T cells are crucial in mediating adaptive immunity and play an essential role in IPF [10]. Based on the expression of CD45RA and CCR7, human T cells can be classified into the following four subpopulations: CD45RA^+^CCR7^+^ naïve (TN), CD45RA^−^CCR7^+^ central memory (TCM), CD45RA^−^CCR7^−^ effector memory (TEM), and CD45RA^+^CCR7^−^ effector memory T cells re-expressing CD45RA (TEMRA) [11]. CD4^+^ TEMRA cells, traditionally implicated in antiviral and antitumor immunity [12], were recently reported increased in IPF hilar lymph nodes; however, their abundance and function in peripheral blood and lung parenchyma remain undefined [13].​

Recent advances in single‑cell RNA sequencing (scRNA‑seq) and spatial transcriptomics have revealed altered cellular composition and dysregulated intercellular communication in the IPF microenvironment, including senescent alveolar type II (AT2) cells [14] and a pathogenic CTHRC1⁺ fibroblast population [15]. Spatially resolved trajectories suggest progressive loss of alveolar homeostasis, compensatory airway‑like transition of AT2 cells, and accumulation of SPP1⁺ macrophages [16]. However, most findings derive from relatively small cohorts, limiting clinical generalizability and precluding causal inference regarding immune–fibrosis relationships. Mendelian randomization (MR), leveraging genome‑wide association data and large‑scale immune trait summaries [17], enables causal inference and is well suited to address this gap [18]. Prior MR studies have implicated short telomeres [19] and elevated interleukin (IL)‑14 levels [20] as risk factors, and higher FUT3 levels as protective [21]; Nevertheless, causal immune cell phenotypes in IPF remain incompletely characterized.

Here, we hypothesized that CD4⁺ TEMRA cells are causally protective against IPF development and are suppressed by Galectin-7 (*LGALS7*) produced by metaplastic basal cells in the IPF epithelium. To test this, we integrated two-sample MR with multi-omics profiling of IPF patient samples and validated CD4⁺ TEMRA reduction in peripheral blood and lung tissue by flow cytometry. Mechanistically, we demonstrate that Galectin-7 competes with IL-7 for receptor binding and inhibits STAT5 phosphorylation, thereby limiting TEMRA differentiation. Guided by these findings, virtual screening identified neoeriocitrin as a Galectin-7 inhibitor, which restored CD4⁺ TEMRA-associated signaling in vitro and attenuated fibrosis in vivo. These results uncover a previously unrecognized epithelial–immune crosstalk axis and nominate Galectin-7 as a tractable therapeutic target in IPF.

## Results

### CD4^+^ TEMRA cells were screened as a causal immune trait for IPF

**Fig. 1:**
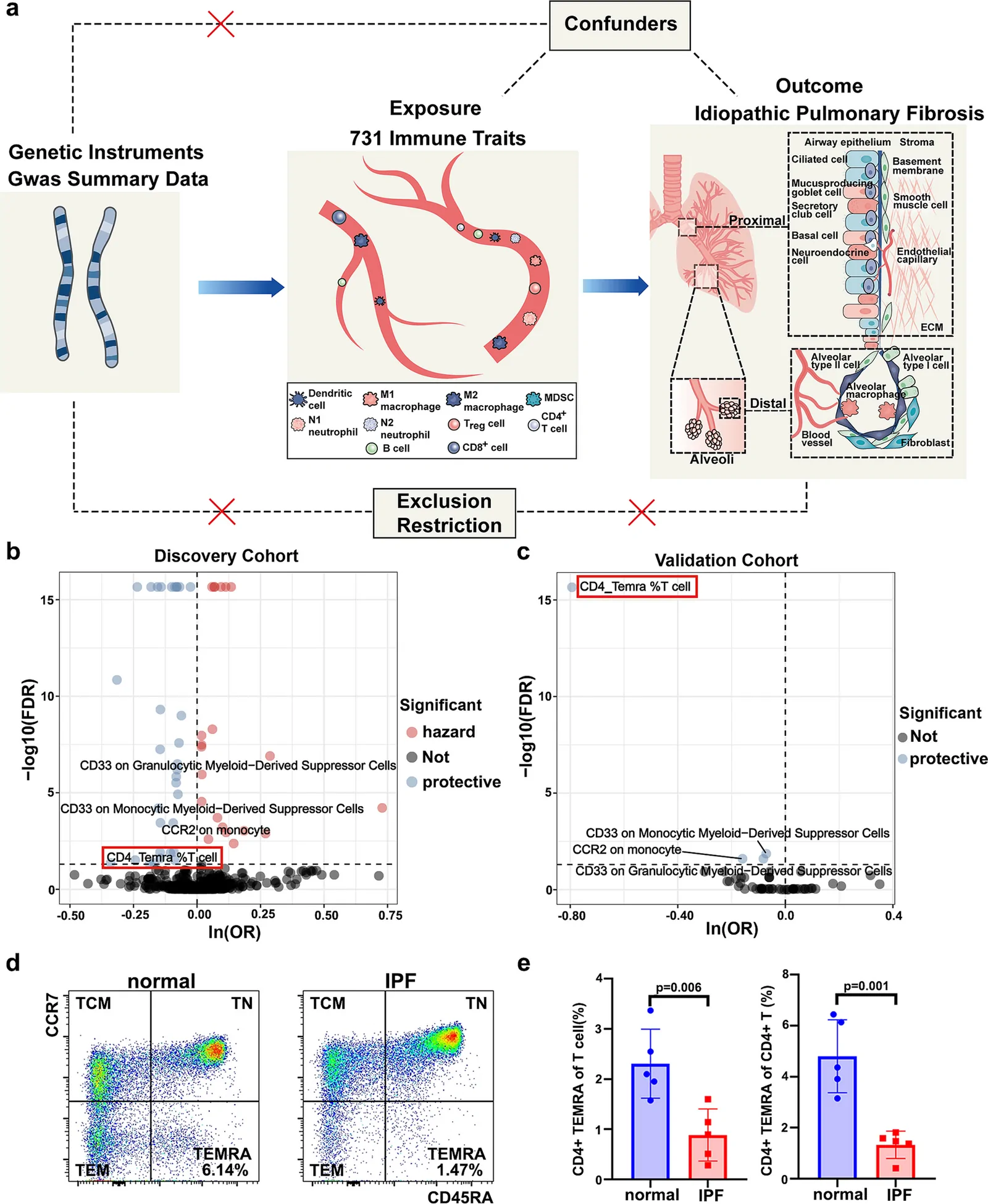
CD4^+^ effector memory T cells re-expressing CD45RA (TEMRA) cells were screened as a causal immune trait for IPF. **a** The flow chat of mendelian randomization analyses identifying the causal relationship between immune traits and IPF. The volcano plots displayed the causal effects of immune traits on IPF in the discovery cohort (**b**) and validation cohort (**c**). **d** Flow cytometry detected the level of CD4^+^ TEMRA in the peripheral blood mononuclear cells (PBMCs) of normal subjects and IPF patients. **e** The histogram compared CD4.^+^ TEMRA abundance between IPF patients and normal subjects. All statistical data were presented as the mean ± SD; *n* = 5

The MR analysis workflow is shown in Fig. 1a and detailed descriptions are provided in Table S1−6. Two-sample Mendelian randomization identified 53 immune traits causally associated with IPF risk (false discovery rate, FDR < 0.05). Among these, only CD4^+^ TEMRA/T cells (%) showed consistent protection across discovery (Fig. 1b) and validation cohorts (Fig. 1c) [discovery cohort: odds ratio (OR) (95% confidence interval, CI): 0.712 (0.565–0.896); validation cohort: OR (95% CI): 0.452 (0.397–0.515), Fig. S1a]. Sensitivity analyses excluded heterogeneity, pleiotropy, and reverse causation (Table S7-9).

Flow cytometry confirmed reduced CD4^+^ TEMRA frequencies in IPF peripheral blood mononuclear cells (PBMCs) versus controls (0.88% vs. 2.31% of T cells, *P* = 0.006; 1.33% vs. 4.80% of CD4^+^ T cells; Fig. 1d, e).

### Core CD4^+^ TEMRA gene signature stratified IPF patients into distinct prognostic groups

**Fig. 2:**
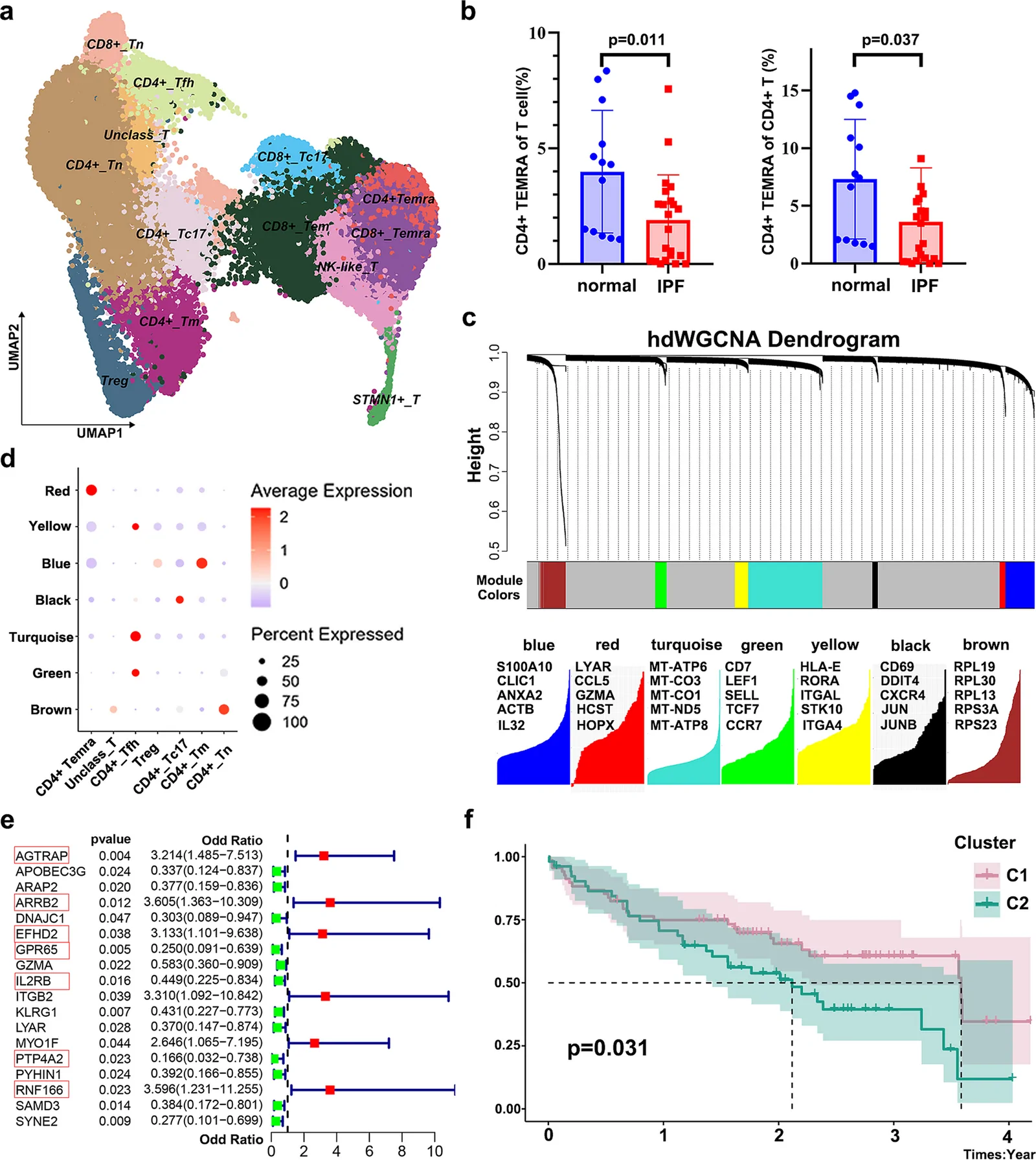
Core CD4^+^ TEMRA gene signature stratified ipf patients into distinct prognostic groups. **a** UMAP dimensional plot displayed the distribution of CD4^+^ TEMRA subtype in peripheral blood T cells. **b** The histogram showed the difference in abundance of CD4^+^ TEMRA in the PBMCs of IPF patients and normal subjects. **c** hdWGCNA identified seven distinct co-expression modules, with key eigengenes illustrated for each module.​ **d** The dot plot shows enrichment of red module genes in CD4^+^ TEMRA. **e** The single-factor logistic regression analysis identified a total of 18 genes with statistically significant differences in odds ratio. **f** Kaplan–Meier analysis showed significantly longer median overall survival in Subtype 1 compared to Subtype 2 (log-rank *p* = 0.031), indicating robust prognostic value

The publicly available scRNA-seq dataset GSE233844 from IPF peripheral blood, including 13 normal controls and 25 IPF samples. T cells were annotated into 13 subsets (Fig. 2a). Uniform Manifold Approximation and Projection (UMAP) visualization revealed clear separation between CD4^+^ and CD8^+^ lineages. Notably, CD4^+^ TEMRA clustered in proximity to CD8^+^ TEMRA. Differentially expressed gene (DEG) analysis identified significant enrichment of cytotoxic markers (*FGFBP2*, *NKG7*, *GZMH*, *GNLY*) in CD4^+^ and CD8^+^ TEMRA (Fig. S2a). CD4^+^ TEMRA abundance was significantly reduced in IPF versus controls among total T cells (mean 1.893% vs 3.991%; *P* = 0.011) and within the CD4^+^ compartment (mean 3.612% vs 7.303%; *P* = 0.037), after excluding three outliers (Fig. 2b, Fig. S2b).

The hdWGCNA analysis delineated a CD4^+^ TEMRA enriched module (red; Fig. 2c, d), and GO enrichment analysis confirmed significant involvement of these genes in T cell-mediated cytotoxicity, cell lysis, and cell killing—pathways central to effector T cell function (Fig. S2c). LASSO regression selected a 7-gene signature (AGTRAP, ARRB2, EFHD2, GPR65, IL2RB, PTP4A2, RNF166) from red module gene set that stratified IPF patients into two prognostic subtypes (Fig. 2e, f; Fig. S2d-f). DEG analysis (log₂FC > 0.585, *p* < 0.05) and subsequent enrichment analysis revealed significant upregulation of ROS metabolism in group with worse prognosis (Fig. S2g, h).

### Regional pro-TEMRA differentiation of CD4^+^ T cells predicts milder lung fibrosis​

**Fig. 3:**
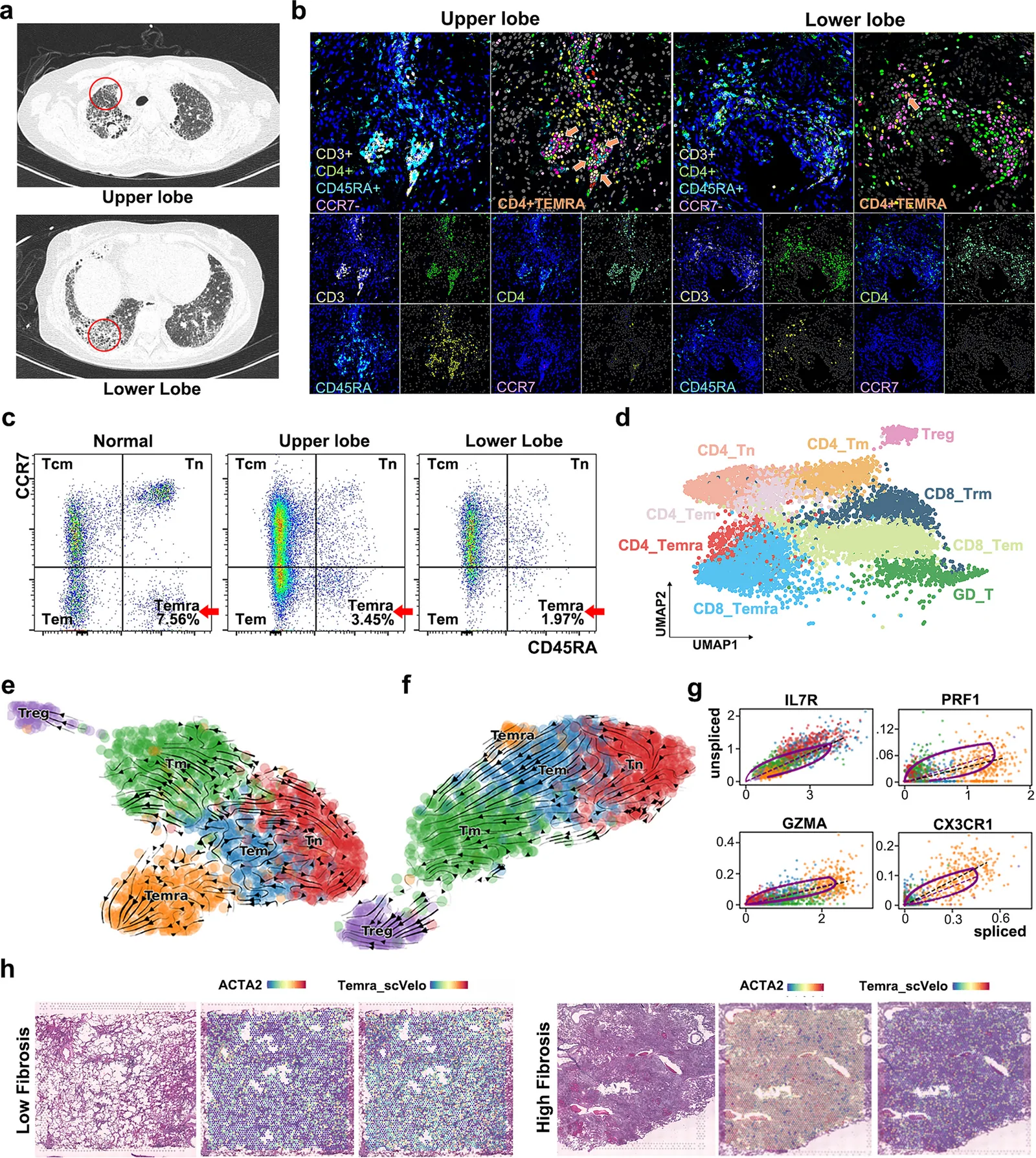
Regional pro-TEMRA differentiation of CD4^+^ T cells predicts milder lung fibrosis. **a** The CT imaging exhibited the fibrosis in the medial upper lobe and lateral lower lobe of the same IPF patient. **b** The mIHC suggested CD4^+^ TEMRA was significantly less infiltrated in more diseased lower lobes than in upper lobes. **c** The proportions of CD4^+^ TEMRA in lung tissue of normal, upper lobe of IPF, and lower lobe of IPF were detected by flow cytometry. **d** UMAP dimensional plot displayed 9 distinct T cell subpopulations. Developmental trajectories revealed CD4^+^ TEMRA as terminally differentiated cells derived mainly from CD4^+^ Tem in the normal lung tissues (**e**) and IPF (**f**). **g** The top 15 genes contributing most strongly to CD4^+^ TEMRA in normal lung tissues were identified as putative driver genes including IL7R, PRF1, GZMA and CX3CR1, and others were shown in Fig. S3g. **h** The spatial transcriptomic analysis indicates that the gene set promoting TEMRA differentiation is expressed at a higher level in regions with lower ACTA2 expression and lower fibrosis severity, while in regions with higher fibrosis severity, the ACTA2 expression level increases significantly and the expression level of the gene set decreases

While CD4⁺ TEMRA cells demonstrated prognostic value in peripheral blood, their direct association with pulmonary fibrosis severity required tissue-level validation. Computed tomography imaging revealed substantial regional heterogeneity within IPF lungs, with upper lobes exhibiting less fibrotic involvement compared to lower lobes in the same individual (Fig. 3a). Spatial validation using multiplex immunofluorescence coupled with automated quantitative pathology confirmed significantly reduced CD4⁺ TEMRA infiltration in fibrotic lower lobe regions versus upper lobe areas (4.50% vs. 21.57%; *p* = 0.014) (Fig. 3b, Fig. S3a). This spatial gradient reinforces the protective role of CD4⁺ TEMRA cells against fibrotic progression in IPF. Flow cytometry identified a progressive decline in CD4⁺ TEMRA frequency from normal lung tissue (7.56%) to minimally affected IPF upper lobes (3.45%) and severely fibrotic IPF lower lobes (1.97%) (Fig. 3c).

Independent scRNA‑seq of IPF and control lung tissues corroborated reduced CD4⁺ TEMRA abundance and highlighted their cytotoxic signature (GZMH, GNLY, FGFBP2; Fig. 3d; Fig. S3b-d). SCENIC identified KLF3, TBX21 and NFATC2 as key regulators of TEMRA identity (Fig. S3e).

RNA velocity mapped CD4⁺ TEMRA as a terminal differentiation state under homeostasis, whereas IPF redirected CD4⁺ T cell fate toward Tregs (Fig. 3e, f). Furthermore, 15 genes contributing most strongly to CD4^+^ TEMRA were identified as putative drivers (Fig. 3g, Fig. S3f). Among these, *CCL5*, *GZMH*, and *GZMA*—highly expressed in CD4^+^ TEMRA—showed prognostic value in bronchoalveolar lavage samples from IPF patients (Fig. S3g). Notably, CD4^+^ TEMRA cells exhibited the highest expression levels of spliced RNAs encoding canonical perforin-family genes—including *GZMH*, *GZMA*, and *GZMB*—among all CD4^+^ T cell subsets. Concurrently, they also showed the highest unspliced-to-spliced RNA ratios for these genes, suggesting sustained high expression of perforin-family mediators in the near term. As the terminal differentiation stage within the CD4^+^ T cell lineage, CD4^+^ TEMRA cells thus represent a peak in cytotoxic potential.

Spatial transcriptomics revealed a negative correlation between a 15‑gene TEMRA‑differentiation signature and ACTA2 expression, linking robust TEMRA commitment to attenuated local fibrosis (Fig. 3h; Fig. S3h).

### IL-7-Mediated promotion of TEMRA differentiation attenuates fibrosis ​

IL7 was predominantly expressed by ciliated and lymphatic endothelial cells, downregulated in IPF lungs (Fig. S4a–d). Colocalization analysis supported a causal link between IL7 and IPF risk (PPH4 = 0.83; Fig. S4e, f), and high IL7 expression correlated with better patient survival (Fig. S4g).

**Fig. 4:**
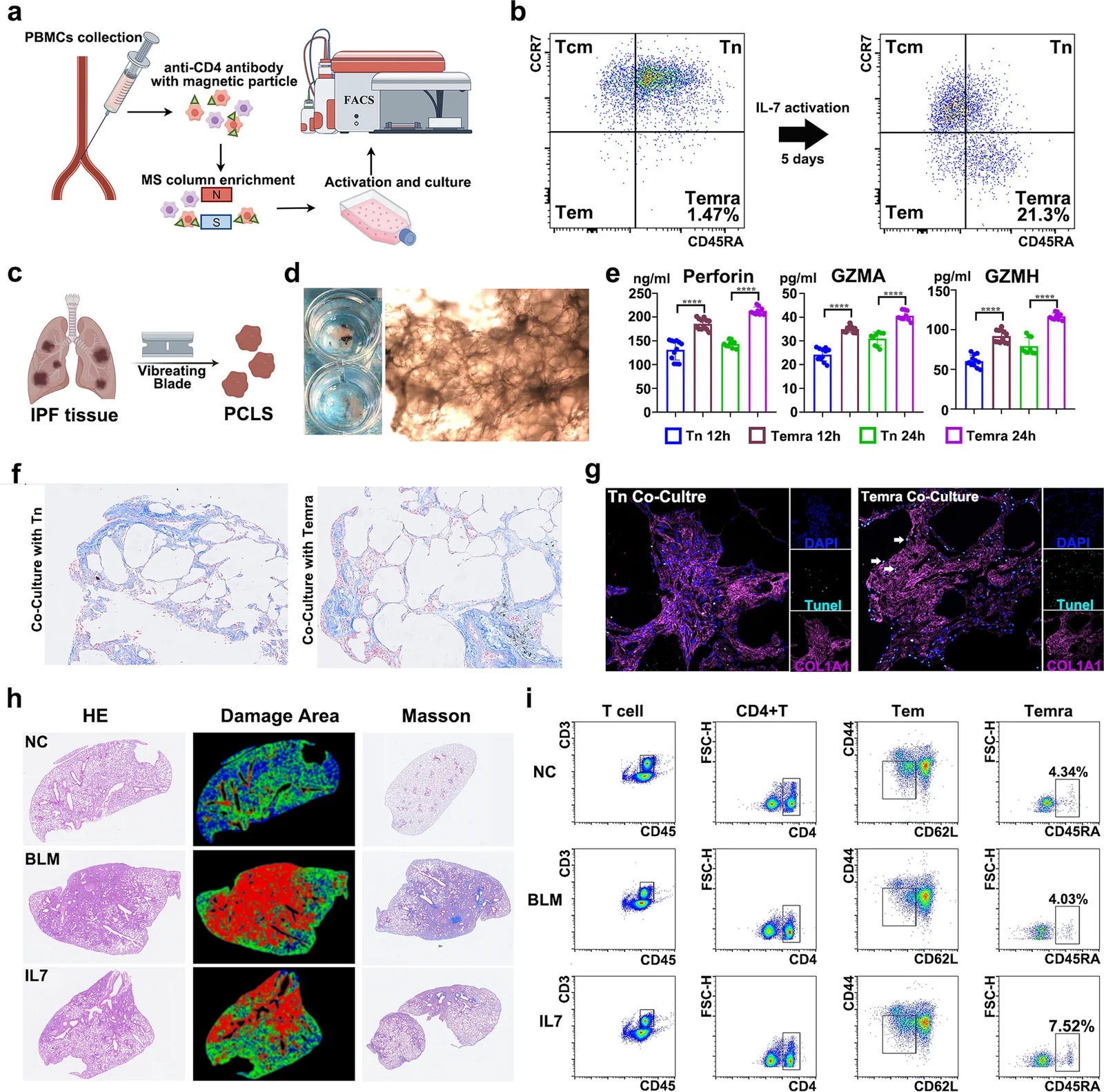
IL-7-Mediated promotion of TEMRA differentiation attenuates fibrosis. **a** Schematic diagram of the experimental workflow: CD4⁺ T cells were isolated from healthy donor PBMCs by magnetic bead-based positive selection, cultured with IL-7 (25 ng/mL) for 5 days, and FACS-sorted for CD4⁺ TEMRA cells, which were then co-cultured with IPF lung-derived PCLS. **b** Flow cytometric analysis showing the proportion of CD4⁺ TEMRA cells among positively selected CD4⁺ T cells after 5 days of IL-7 stimulation (> 20%). **c** Schematic diagram of PCLS. **d** PCLS under light microscopy. **e** ELISA quantification of GZMH, GZMA, and perforin levels in the supernatant of CD4⁺ TEMRA (12 h, *n* = 12; 24 h, *n* = 9) versus CD4⁺ Tn cells (12 h, *n* = 12; 24 h, *n* = 9). **f** Masson’s trichrome staining showing reduced collagen deposition in PCLS co-cultured with CD4⁺ TEMRA cells compared with CD4⁺ TN cells. **g** The fibroblast apoptosis within collagen-rich areas of PCLS. **h** The LungDamage algorithm calculates the areas of lung fibrosis damage and Masson’s trichrome staining in mice. **i** At 21 days, the proportion of CD4^+^ TEMRA in the treatment group and the control group was significantly higher than that in the fibrosis model group

​​CD4⁺ T cells were isolated from healthy donor peripheral blood using magnetic bead-based positive selection and cultured in vitro for fluorescence-activated cell sorting (FACS) (Fig. 4a). After confirming that in vitro culture with recombinant human IL‑7 (25 ng/mL) for 5 days significantly increased the proportion of CD4⁺ TEMRA cells to > 20% among positively selected CD4⁺ T cells (as assessed by flow cytometry, Fig. 4b), these cells were FACS‑sorted for subsequent anti‑fibrotic assays via PCLS–CD4⁺ TEMRA cell coculture system (Fig. 4c, d). ELISA revealed that the levels of cytotoxic effector molecules, including GZMH, GZMA, and perforin, were significantly higher in the supernatant of CD4⁺ TEMRA cells than in that of CD4⁺ Tn cells (Fig. 4e). Furthermore, IPF lung-derived PCLS co-cultured with CD4⁺ TEMRA cells exhibited reduced fibrotic deposition compared with CD4⁺ Tn cells (Fig. 4f, Fig. S4h). Notably, within collagen-rich areas, an increased level of fibroblast apoptosis was observed in the TEMRA-co-cultured PCLS compared with control PCLS (Fig. 4g).

To further validate the protective role of IL-7 and TEMRA cells, a murine pulmonary fibrosis model was established via intratracheal instillation of bleomycin (BLM). Animals were divided into three groups: (1) control group receiving PBS; (2) fibrosis model group treated with BLM alone; and (3) treatment group (BLM + IL-7) administered recombinant IL-7 protein intraperitoneally (10 μg/kg every 72 h) starting 7 days post-BLM exposure. On day 21 post-modeling, animals were euthanized for lung tissue analysis. Quantitative assessment of lung injury revealed a significantly lower percentage area of severe injury in the IL-7 treatment group compared to the BLM-induced model group (28.88% vs 64.43%, respectively; *p* = 0.005; Fig. 4h, Fig. S4i). Quantitative analysis of Masson’s trichrome staining showed that the collagen volume fraction (CVF) in the BLM + IL-7 group was significantly reduced compared with the BLM group (*p* < 0.001; Fig. 4h, Fig. S4j). From the initiation of modeling, peripheral blood was collected periodically via the retro-orbital venous plexus for multicolor flow cytometry. Longitudinal monitoring revealed a sustained increase in the proportion of CD4⁺ TEMRA cells among CD4⁺ T cells in the BLM + IL-7 group compared to the BLM group (Fig. S4k). By day 21, a significant difference in CD4⁺ TEMRA frequency was observed between the treatment and model groups (2.309% vs 1.597%; *p* = 0.0292; Fig. 4i, Fig. S4l).

### Galectin-7 reduces TEMRA cell frequency and exacerbates pulmonary fibrosis​

**Fig. 5:**
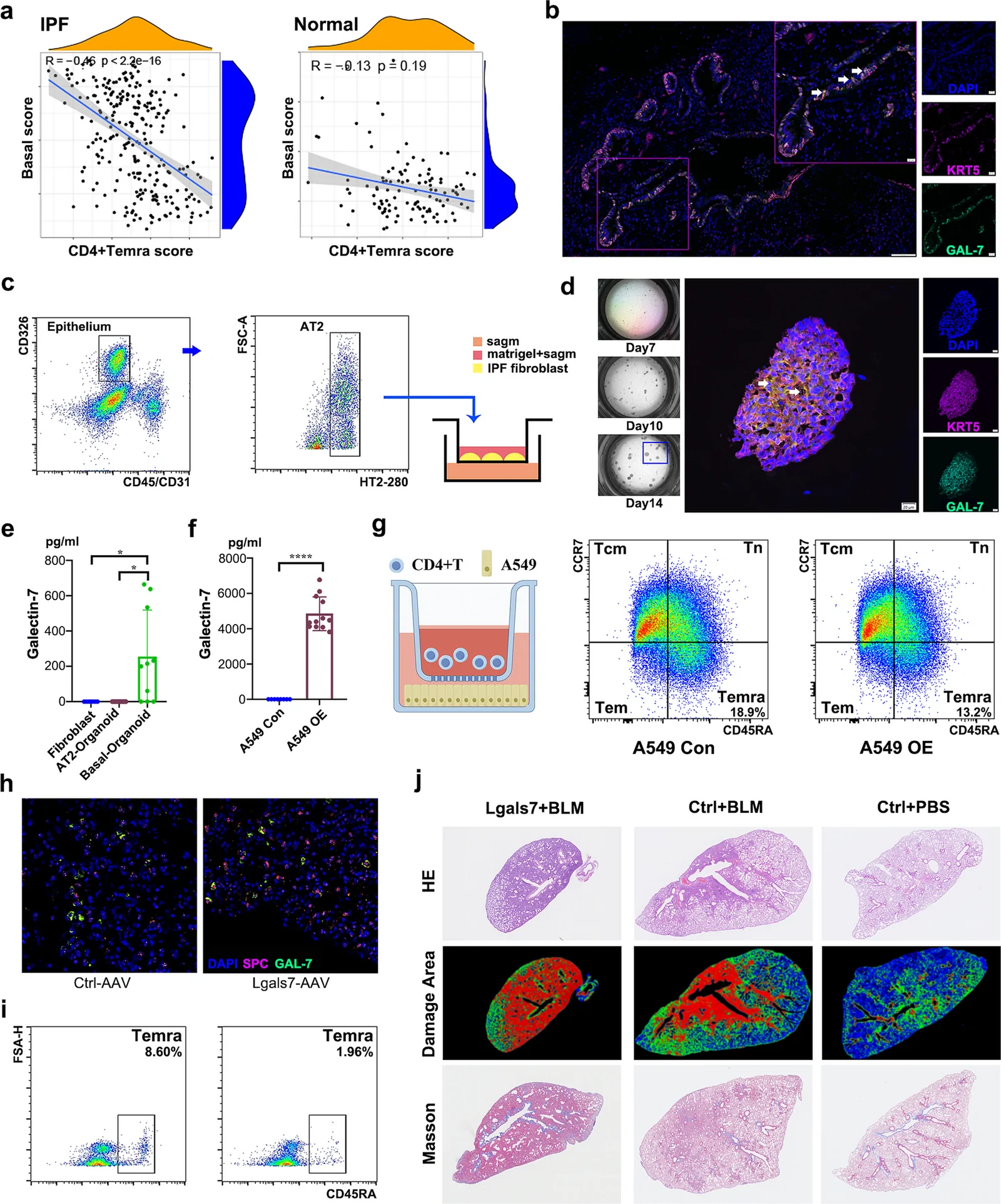
Galectin-7 reduces TEMRA cell frequency and exacerbates pulmonary fibrosis. **a** The ssGSEA of scRNA-seq-derived signature genes revealed a robust negative correlation between basal cells and CD4⁺ TEMRA cells in IPF lungs (R = –0.46, *p* < 2.2e–16), which was absent in normal controls (R = –0.13, *p* = 0.19). **b** KRT5 and Galectin-7 were highly expressed and significantly co-localized in IPF lung tissue. **c** Schematic of the organoid co-culture model: AT2 cells were sorted from donor lungs and cultured to form metaplastic basal cell organoids; conditioned medium was collected for Galectin-7 ELISA. **d** Immunofluorescence showing KRT5 and Galectin-7 co-localization in AT2-derived organoids. Scale bar, 50 μm. **e**​ Galectin-7 protein levels in supernatants of primary IPF fibroblasts (*n* = 9), AT2-only organoids (*n* = 13), and metaplastic basal cell organoids (*n* = 10). **f**​ Galectin-7 protein levels in supernatant of A549 cells transfected with *LGALS7* overexpression plasmid (*n* = 12) or empty vector (*n* = 8). **g**​ Flow cytometric analysis of CD4⁺ TEMRA proportion after co-culture with *LGALS7*-overexpressing A549 cells. **h** Immunofluorescence quantification of Galectin-7 signal intensity in lung epithelium of Ctrl + BLM versus *Lgals7* + BLM mice. **i**​ Flow cytometric analysis of CD4⁺ TEMRA frequency among CD4⁺ T cells in peripheral blood on day 14. **j** Representative Masson’s trichrome staining and quantification of CVF and lung injury area in Ctrl + PBS, Ctrl + BLM, and *Lgals7* + BLM groups. Data are presented as mean ± SD. *P < 0.05, **P < 0.01

Analysis of scRNA-seq data via ssGSEA demonstrated that basal cells were significantly and negatively correlated with CD4⁺ TEMRA abundance specifically in IPF (R = −0.46, *p* < 2.2e-16), but not in normal lung tissue (R = −0.13, *p* = 0.19, Fig. 5a, Fig. S5a). Integration of the published gene set characterizing AT2-metaplastic basal cells versus normal basal cells [22] with two additional datasets—DEGs between IPF and normal basal cells from GSE146981 and genes negatively correlated with CD4^+^ TEMRA abundance from GSE47460—was performed. Intersection of these three datasets uniquely identified *LGALS7* as the common gene satisfying all criteria. (Fig. S5b). Interestingly, *LGALS7* expression was restricted to basal cells specifically in IPF lungs (Fig. S5c-d). Immunofluorescence analysis further demonstrated co-localization of KRT5 and Galectin-7 in IPF lung tissues (Fig. 5b). Meanwhile, the expression of *LGALS7* was significantly negatively correlated with CD4^+^ TEMRA, and IPF patients with high expression of *LGALS7* exhibited worse prognosis (Fig. S5e-g).

While fibroblast co-culture has been established as a robust method to induce the metaplastic transition of primary human AT2 cells into basal cells (Fig. 5c), the present study provides the first evidence that these induced cells express Galectin-7, as demonstrated by immunofluorescence staining (Fig. 5d). Metaplastic basal cell organoids, but not AT2 organoids or primary fibroblasts, secreted Galectin‑7 (Fig. 5e). *LGALS7* overexpression in A549 cells recapitulated this phenotype and suppressed CD4⁺ TEMRA differentiation in co‑culture assays (Fig. 5f, g; Fig S5h).

AAV‑Lung3‑mediated *Lgals7* overexpression in mouse lung epithelium (Fig. S5i) Immunofluorescence (Sftpc⁺/Galectin-7⁺ co-staining) further verified a 1.766-fold increase in Galectin-7 signal intensity in epithelium cells of the *Lgals7* + BLM group compared to the Ctrl + BLM group (*p* = 0.007, *n* = 3; Fig. 5h, Fig. S5j).

Flow cytometric analysis of peripheral blood on day 14 showed a significant reduction in the frequency of CD4⁺ TEMRA cells among CD4⁺ T cells in the *Lgals7* + BLM group (4.3%) compared to the Ctrl + BLM group (10.4%; *p* = 0.0261; Fig. 5i, Fig. S5k). Masson’s trichrome staining revealed diffuse collagen deposition (blue staining) in alveolar septa and perivascular regions in the *Lgals7* + BLM group. The CVF was significantly higher in this group (26.50 ± 2.9%) than in the Ctrl + BLM group (21.40 ± 3.1%; *p* = 0.030), suggesting that *Lgals7-*overexpression exacerbates fibrosis by promoting extracellular matrix remodeling (Fig. 5j, Fig. S5l). Quantitative assessment of lung injury further confirmed that *Lgals7-*overexpression (72.58 ± 11.8%) exacerbated pulmonary damage, with the *LGALS7* + BLM group exhibiting significantly more severe injury compared to the Ctrl + BLM group (29.85 ± 6.83%, Fig. 5j, Fig. S5m).

### Galectin-7 competes with IL-7 for receptor binding and suppresses STAT5 phosphorylation

**Fig. 6:**
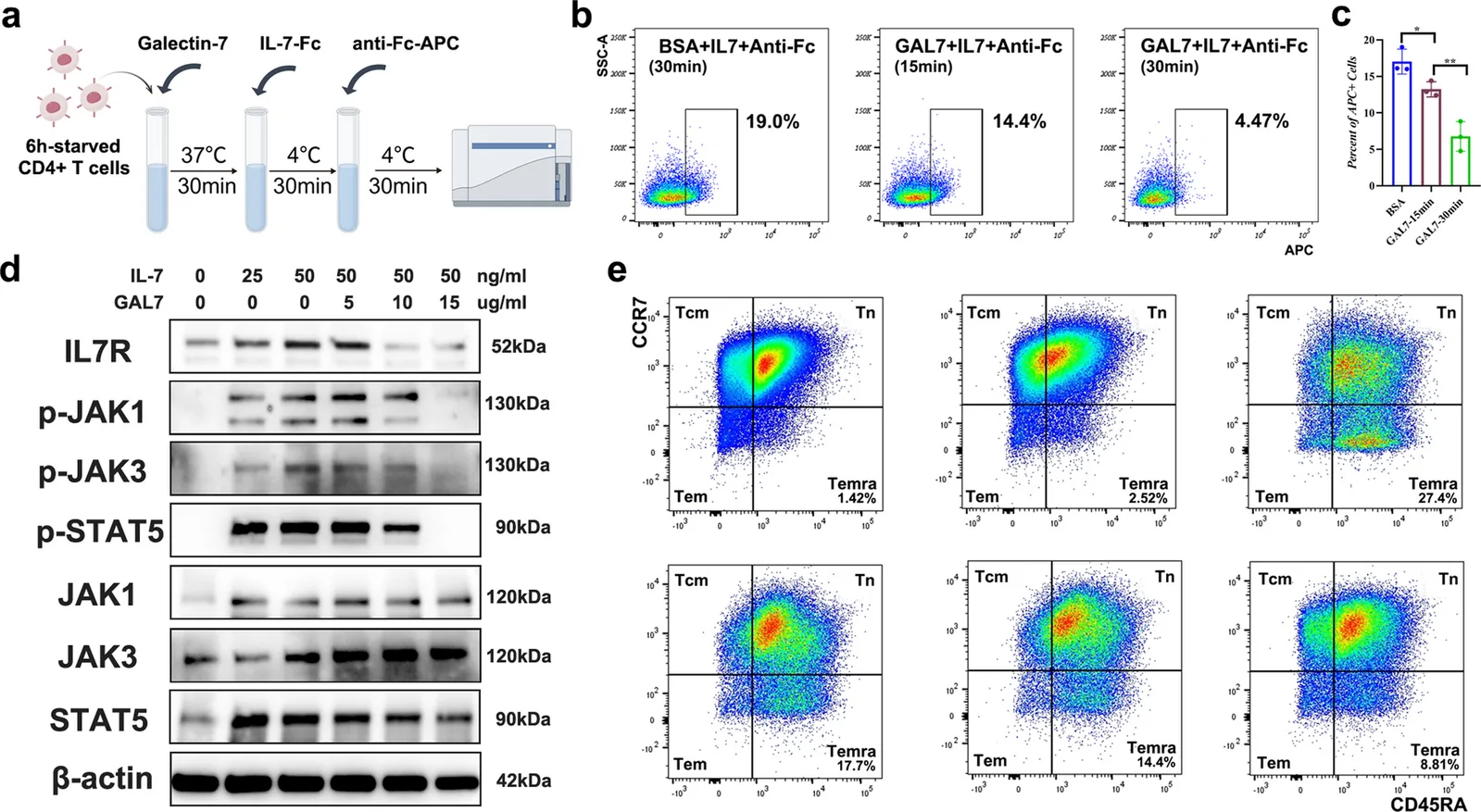
Galectin-7 competes with IL-7 for receptor binding and suppresses STAT5 phosphorylation. **a** Schematic of the competitive binding assay: starved CD4⁺ T cells were pre-incubated with Galectin-7 (15 μg/mL) for 15 or 30 min, washed, incubated with IL-7-Fc, and stained with APC-conjugated anti-Fc antibody. **b** Flow cytometry showed that cells pre-treated with Galectin-7 for 30 min displayed markedly lower APC signals than those treated for 15 min or with BSA **c** Cells were treated with BSA for 30 min as a control (*n* = 3, 17.03 ± 1.70), or with Galectin-7 for 15 min (*n* = 3, 13.23 ± 1.04) or 30 min (*n* = 3, 6.77 ± 2.04). **d** Western blot analysis of p-STAT5, STAT5, p-JAK1, JAK1, p-JAK3, JAK3, and IL-7Rα in CD4⁺ T cells stimulated with IL-7 (0, 25, 50 ng/mL) with or without Galectin-7 (5, 10, 15 μg/mL). β-actin served as loading control. **e** Flow cytometric analysis of CD4⁺ TEMRA proportion after treatment with IL-7 (50 ng/mL) and increasing concentrations of STAT5-IN-1

Galectin‑7 competed with IL‑7 for receptor binding, dose‑dependently suppressing IL‑7‑induced STAT5 and JAK1 phosphorylation (Fig. 6a–d). Pharmacological STAT5 inhibition mimicked Galectin‑7 effects, abrogating IL‑7‑driven TEMRA differentiation (Fig. 6e).

To assess the functional impact of STAT5 inhibition, we treated CD4⁺ T cells with the STAT5 inhibitor STAT5-IN-1 prior to IL-7 stimulation. IL-7 alone markedly increased the CD4⁺ TEMRA proportion (from 1.153% to 25.33%, *p* < 0.0001). This effect was progressively attenuated by increasing concentrations (10 μM, 25 μM and 50 μM) of STAT5-IN-1 (Fig. 6e, Fig. S5n). Together, these results suggest that Galectin-7 inhibits IL-7-induced STAT5 signaling by competing for receptor binding, thereby limiting CD4⁺ TEMRA cell differentiation.

### Targeting Galectin-7 to restore CD4^+^ TEMRA abundance and identify a novel inhibitor

**Fig. 7:**
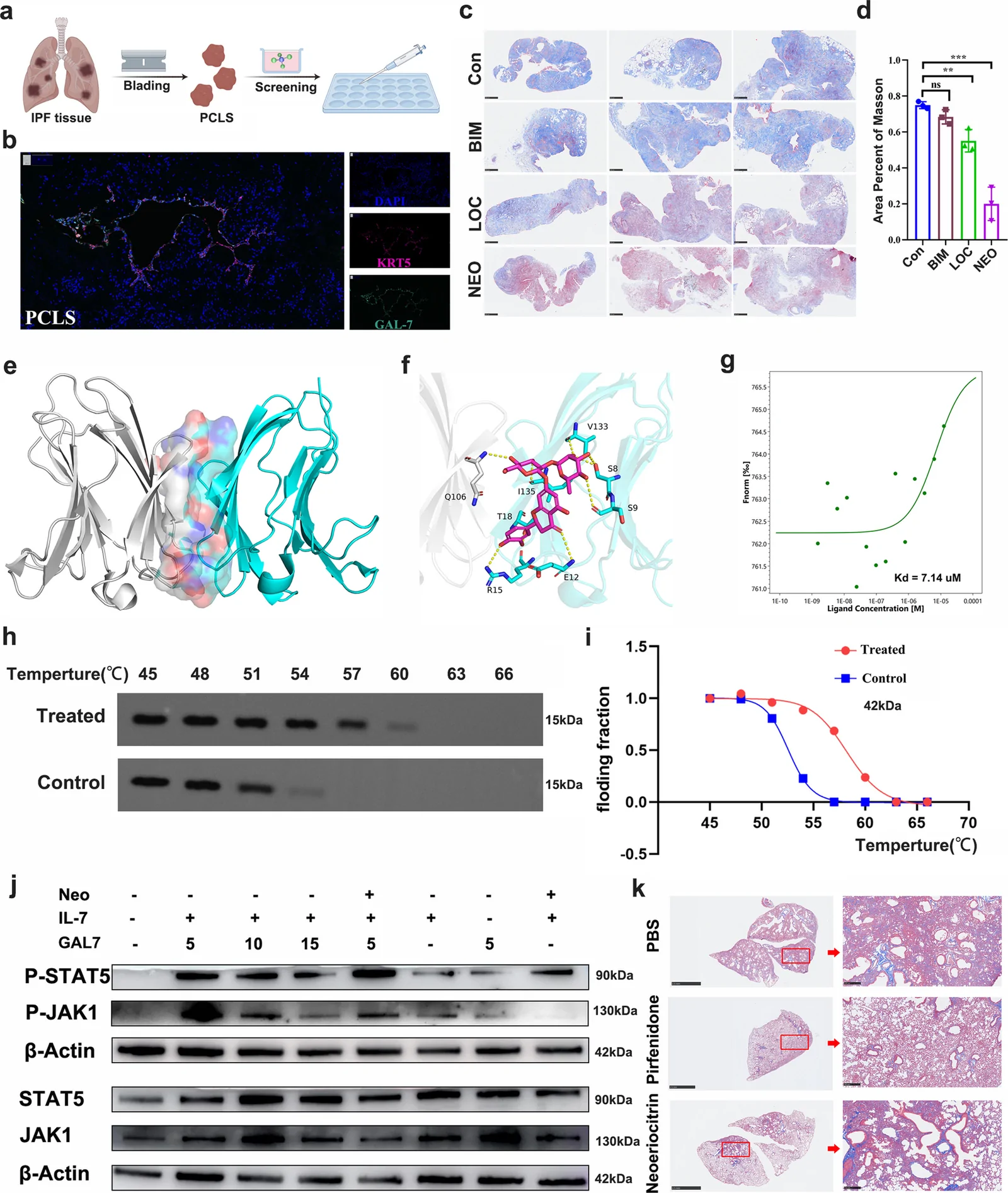
Targeting Galectin-7 to restore CD4^+^ TEMRA abundance and identify a novel inhibitor. **a** The efficacy of the small molecule compounds obtained through virtual screening was verified by PCLS. **b** Immunofluorescence analysis indicated that there are Galectin-7-positive basal cells in PCLS. **c** Masson staining results of the control group and treatment groups based on PCLS. **d** Quantitative analysis of the stained PCLS (n=3 per group) revealed that Neoeriocitrin significantly decreased collagen deposition (Mean ± SD: 0.200 ± 0.092), representing approximately a 73% reduction relative to the control group (0.749 ± 0.020). In contrast, Locustatachykinin I acetate showed a moderate decrease (0.551 ± 0.064), while BIM 23042 Acetate had minimal effect (0.684 ± 0.040) compared to the control. **e** A small molecule was designed to bind a cryptic pocket at the dimer interface, locking it into a stable state. **f** Structural basis of Neoeriocitrin (magenta) recognition by Galectin-7.​The binding site is formed by residues from chain A (gray) and chain B (cyan) of the protein dimer **g** MST assay proved high affinity between Galectin-7 and neoeriocitrin. **h** Cellular thermal shift assay (CETSA): HEK293 cell lysates containing Galectin-7 were incubated with or without neoeriocitrin (100 μM) and heated from 45℃ to 66℃. Western blot shows soluble Galectin-7 levels. **i** Quantification of the thermal denaturation curves further confirmed a marked rightward shift in the presence of neoeriocitrin. **j** Western blot analysis of p-STAT5, STAT5, p-JAK1, and JAK1 in CD4^+^ T cells stimulated with IL-7 and Galectin-7, with or without neoeriocitrin (10 μM). **k** Representative Masson’s trichrome staining of lung sections from AAV-*Lgals7* mice treated with aerosolized vehicle, pirfenidone, or neoeriocitrin. Quantification of CVF is shown

High‑throughput PCLS screening of 20,000 compounds identified neoeriocitrin as the most potent collagen‑reducing agent (~73% decrease vs. control; Fig. 7a–d). Molecular docking and microscale thermophoresis confirmed neoeriocitrin binding to the Galectin‑7 dimer interface (Kd = 7.14 μM; Fig. 7e–g). Cellular thermal shift assays demonstrated that neoeriocitrin stabilized Galectin‑7 against thermal denaturation (Fig. 7h, i).

In vitro, human PBMC-derived CD4⁺ T cells were stimulated with IL-7 and Galectin-7 in the presence or absence of neoeriocitrin. Western blot analysis revealed that treatment with 10 µM neoeriocitrin partially restored the phosphorylation levels of STAT5 and JAK1 that had been suppressed by Galectin-7 (Fig. 7j). Notably, neoeriocitrin alone did not significantly affect STAT5 phosphorylation, indicating that its effect is specific to counteracting Galectin-7-mediated inhibition. In vivo, we utilized an AAV-based model in which *Lgals7* was overexpressed in mouse lung epithelial cells. Mice were treated with aerosolized pirfenidone or neoeriocitrin. Both compounds significantly attenuated pulmonary fibrosis compared with the vehicle control group (Fig. 7k, Fig. S6a). Collectively, these findings highlight the clinical translational potential of neoeriocitrin as a novel therapeutic candidate for IPF.

## Discussion

This study establishes CD4⁺ TEMRA as a causally protective immune subset in IPF, extending its canonical antiviral and antitumor roles to tissue remodeling diseases. Unlike previous studies that predominantly focused on the antiviral and anti-tumor roles of CD4⁺ TEMRA cells in infectious diseases and malignancies [23–25], the present study extends these findings to a tissue-remodeling disease, IPF, by demonstrating a previously unrecognized protective role of this subset against fibrotic progression. Flow cytometry and single-cell RNA sequencing consistently demonstrated a reduction in the proportion of CD4⁺ TEMRA cells in both peripheral blood and lung tissues of IPF patients, while scRNA-seq analysis further revealed their potent cytotoxic potential characterized by high expression of *GNLY*, *GZMH*, *FGFBP2*, and so forth [26, 27] and a low exhausted state characterized by low expression of *CD7* [28] and *FOS* [29].

There are two opposing views regarding the nature of CD4⁺ TEMRA cells. Some studies have characterized them as senescence-associated T cells that accumulate with age in healthy individuals [12]. In contrast, a recent study demonstrated that terminal effector CD4⁺ T cells and TEMRA cells exhibit high clonality and cytotoxicity, with their abundance remaining stable across age groups [30]. Our findings support the latter interpretation, as the reduction of CD4⁺ TEMRA in IPF patients was independent of age and correlated with disease severity rather than chronological aging. Cellular differentiation trajectory analysis, supported by driver gene sets, indicates that CD4⁺ TEMRA cells—as the differentiation terminus of TEM cells—can partially reflect the cytotoxic potency that TEM cells may attain in a subsequent period. Thus, the abundance of CD4⁺ TEMRA cells may be regarded as a biomarker for TEM cell cytotoxicity. Furthermore, spatial transcriptomics revealed a negative correlation between the expression level of gene sets driving TEMRA differentiation and the degree of regional fibrosis.​ The observed impairment in T cell cytotoxicity from our multi-omics analysis points to the restoration of this function as a key therapeutic goal. This strategy directly mirrors the increasingly explored use of CAR-T treatments against fibrotic conditions [31, 32]. By employing a novel LNP-mRNA system to generate transient FAP-targeted CAR-T cells in vivo, a recent study demonstrated a powerful therapeutic strategy against pulmonary fibrosis [33]. The research revealed that resolving ECM stiffness restored alveolar epithelial differentiation and immune homeostasis, ultimately promoting lung regeneration.

In parallel to CAR-T approaches, recent research has also explored the use of the CTLA-4 inhibitor ipilimumab, a monoclonal antibody that augments T cell function, to accelerate the resolution of pulmonary fibrosis [34]. However, a key distinction lies in the experimental systems used. While previous studies relied on murine pulmonary fibrosis models [33, 34], which do not fully recapitulate human IPF pathophysiology [35], our study directly interrogated the human IPF microenvironment using patient-derived lung tissues, organoids, and PCLS. This approach allowed us to identify Galectin-7 as a clinically relevant mediator of T-cell dysfunction that may not be readily captured in conventional animal models. Our findings reveal that the reduction of CD4⁺ TEMRA cells resulted from a dysregulated cytokine milieu, characterized by diminished levels of the positive regulator IL-7 and the emergence of the negative regulator Galectin-7. Although IL-7 is critical for T cell proliferation and functional activation [36], supplementation of IL-7 alone failed to restore the CD4⁺ TEMRA population in a Galectin-7-presenting organoid co-culture system. Notably, the enrichment of Galectin-7 in IPF metaplastic basal cells gains significance in light of our prior work showing a fundamental species-specific difference: human AT2 cells can be reprogrammed into a pathological basal cell state by IPF fibroblasts, whereas murine AT2 cells cannot [22]. Similarly, the negative regulatory relationship between Galectin-7^+^ basal cells and CD4⁺ TEMRA cells identified in this study provides direct mechanistic validation for the initial Mendelian randomization results, further clarifying the crucial role of CD4⁺ TEMRA cells in IPF.

Unlike Galectin‑3 [37–39], which is broadly expressed and has shown limited clinical benefit in IPF trials [40], Galectin‑7 is selectively upregulated in metaplastic basal cells, offering a more specific therapeutic target. This restricted expression pattern, combined with our functional validation in human lung organoid and PCLS models, positions Galectin-7 as a more specific and potentially safer therapeutic target for IPF. In non-melanoma skin cancer, Galectin-7 has been demonstrated to inhibit T-cell activity, thereby facilitating immune evasion [41]. The limited clinical success of Galectin-3 inhibitors [40] heightens the therapeutic relevance of Galectin-7, which has been functionally verified in human lung organoid and PCLS models, positioning it as a compelling target worthy of further development for IPF. Interestingly, among IPF patients stratified based on CD4⁺ TEMRA signature genes, the subgroup with poor prognosis exhibited significantly upregulated ROS metabolism. The Galectin family plays important roles in ROS generation; for instance, Galectin-8 and Galectin-9 can induce ROS production in neutrophils [42]. Concurrently, ROS significantly influences the function of Galectin family members—under infectious or inflammatory conditions, increased ROS can upregulate the expression of Galectin-3 in immune cells, thereby modulating inflammatory responses [43]. The intricate interplay between ROS and Galectin family members provides valuable new insights for elucidating the mechanism of action of Galectin-7 in IPF.

​​Based on an integrated approach of virtual screening and validation using PCLS, we have preliminarily identified neoeriocitrin as a promising compound that targets Galectin-7 and exhibits potential for treating fibrosis. Importantly, the therapeutic potential of neoeriocitrin is independently supported by prior studies demonstrating the anti-fibrotic efficacy of QFAE-nB, a flavonoid extract containing neoeriocitrin as one of its six principal constituents, in a mouse model of pulmonary fibrosis [44]. Our findings extend this observation by identifying Galectin-7 as a specific molecular target of neoeriocitrin, thereby providing a mechanistic basis for its anti-fibrotic activity that was not addressed in previous studies. Considering the relatively low upregulation of *Lgals7* expression in the bleomycin-induced mouse model of pulmonary fibrosis, it is plausible that neoeriocitrin may exert additional anti-fibrotic effects through pathways independent of Galectin-7 targeting.

Several limitations of this study should be acknowledged. First, although Mendelian randomization analysis established a causal relationship between CD4⁺ TEMRA cells and IPF risk, the precise molecular mechanisms by which these cells exert their anti-fibrotic effects in the lung microenvironment warrant further investigation. Second, the identification of Galectin-7 as a therapeutic target and the discovery of neoeriocitrin were primarily based on in silico virtual screening and ex vivo PCLS validation; additional in vivo pharmacokinetic, toxicity, and efficacy studies in more physiologically relevant animal models are required before clinical translation. Third, the bleomycin-induced mouse model does not fully recapitulate the chronic, progressive nature of human IPF, and the relatively modest upregulation of *Lgals7* in this model may limit the evaluation of Galectin-7-targeted therapies. Fourth, the sample size for certain validation experiments, particularly the organoid cultures and PCLS assays, was constrained by the limited availability of human lung tissue from transplant donors. Finally, while we demonstrated that neoeriocitrin targets Galectin-7, the possibility of off-target effects contributing to its anti-fibrotic activity cannot be excluded, given the known pleiotropic effects of flavonoid compounds. Future studies with larger cohorts, improved animal models, and more comprehensive pharmacological profiling are warranted to confirm and extend these findings.

In summary, we uncover an epithelial–immune axis wherein metaplastic basal cell‑derived Galectin‑7 suppresses IL‑7–STAT5 signaling and limits CD4⁺ TEMRA differentiation, thereby promoting fibrosis. Targeting this axis with neoeriocitrin represents a promising disease‑modifying strategy for IPF.

## Methods

### Mendelian randomization analysis of immune cell traits and IPF risk

We obtained GWAS summary statistics for 731 immune cell traits (GCST0001391–GCST0002121) from the GWAS Catalog, encompassing absolute counts, relative counts, morphological parameters, and median fluorescence intensities from 3,757 individuals and approximately 22 million SNPs [17]. IPF data were obtained from two independent cohorts: a discovery cohort (4,125 cases, 20,464 controls) from the IPF Genetics Consortium [45] and a validation cohort (1,028 cases, 196,986 controls) from the MRC-IEU (Table S1). All data were derived from European-ancestry populations.

Genetic instrumental variables (IVs) were selected based on three key MR assumptions: (1) strong association with exposure (*P* < 5 × 10⁻⁸), (2) independence from confounders, and (3) influence on outcome only through exposure. We applied additional filters including linkage disequilibrium (R^2^ < 0.001 within 10,000 kb), instrument strength (F-statistic > 10), and minor allele frequency (> 0.01). Palindromic SNPs were excluded during effect allele harmonization.

Inverse-variance weighted (IVW) and Wald ratio methods served as primary analytical approaches, supplemented by MR-Egger, weighted median, and mode-based methods. Effect sizes were reported as ORs with 95% confidence intervals, with FDR correction for multiple testing. Sensitivity analyses included Cochran’s Q test for heterogeneity, MR-Egger intercept test for directional pleiotropy, and MR-PRESSO global test with outlier removal [46]. Immune cell traits showing significant associations (FDR < 0.05) without evidence of heterogeneity or pleiotropy in the discovery cohort were advanced to validation.

Steiger filtering (FDR < 0.05) confirmed causal directionality, while bidirectional MR analysis evaluated reverse causation. Phenotype scanning via Phenoscanner identified potential pleiotropic associations between CD4^+^TEMRA-associated SNPs and IPF risk factors, applying criteria including genome-wide significance (*P* < 5 × 10⁻⁸), European ancestry, and documented biological relevance to IPF pathogenesis [47].

### Human specimens and study approvals

Human lung tissues and peripheral blood were collected from patients with IPF undergoing lung transplantation and from brain-dead organ donors deemed unsuitable for transplantation, with written informed consent obtained from patients or legally authorized representatives. Normal lung specimens were selected from the distal-most regions of donor lungs. IPF diagnosis followed the 2018 ATS/ERS guideline. Cases with comorbid interstitial lung diseases, active infection, or malignancy were excluded. Sample sizes: IPF lung *n* = 4, normal lung *n* = 4 (WUXI scRNA-seq cohort); IPF PBMC *n* = 5, control PBMC *n* = 5 (flow cytometry); additional specimens as detailed in Table S11. The study was approved by the Ethics Committees of Nanjing Medical University (2020–374) and Wuxi People’s Hospital (KY24162).

### Tissue dissociation

Freshly resected human pulmonary tissues were cut into small fragments and washed in PBS and Hank’s Balanced Salt Solution (HBSS) for 10 min. The fragments were compressed to remove liquid and then cut into 1 mm^3^ pieces. The HBSS involving 15 U/mL Dispase II (Catalog # 17,105,041, Thermo Fisher), 225 U/mL collagenase type I (Catalog # 17,100,017, Thermo Fisher), 100 U/mL Dnase (Catalog # DN25, Sigma-Aldrich), and 1% penicillin/streptomycin/fungizone were used to digest the pieces for 45 min at 37 °C by gentle rocking. The suspension was mixed with an equal volume Dulbecco’s Modified Eagle Medium to stop digestion and then continuously filtered through gauze and 100-µm, 70-µm, and 40-µm strainers. Red blood cells were removed using red blood cell lysis buffer.

### Single-Cell RNA Sequencing (scRNA-seq) and data processing

Single-cell suspensions were processed using the 10 × Genomics Chromium platform to generate barcoded libraries with the Single Cell 3’ Reagent Kit. Briefly, single cells were encapsulated in gel bead-in-emulsions where mRNA was reverse-transcribed to produce barcoded cDNA. After amplification, Illumina-compatible libraries were constructed and sequenced.

Raw sequencing data were processed using Cell Ranger to generate feature-barcode matrices. Quality control was performed to remove doublets and low-quality cells based on UMI counts (≥ 1000), gene detection (300–6000 genes/cell), and mitochondrial content (≤ 20%). Batch effects were corrected using Harmony, and cells were clustered via UMAP visualization. Cell types were annotated using established markers [48–50], and differential expression analysis was conducted using Seurat. Cell subtype abundances were quantified in bulk RNA-seq data using ssGSEA. The single-cell RNA velocity analysis was applied to the scRNA-seq data to explore the dynamic aspects of gene expression [51]. The hdWGCNA package in R was used to construct a weighted gene co-expression network. The NMF algorithm from the “NMF” package (version 0.20.6) was employed to obtain distinct subtypes of patients with IPF from the GSE28221 dataset based on genes identified by hdWGCNA [52].

### Cell culture, plasmid transfection, and lentiviral transduction

The human alveolar epithelial cell line A549 (Catalog # CL-0014, Pricella) was obtained from Wuhan Pricella Biotechnology Co., Ltd. The human embryonic kidney cell line HEK-293 T was purchased from Shanghai Anwei Biotechnology Co., Ltd. (Shanghai, China). Both cell lines were cultured in DMEM/F12, 10% FBS, 37 °C, 5% CO_2_ and passaged for fewer than 10 generations before experiments. The *LGALS7-*overexpression plasmid was constructed and provided by GeneChem (Shanghai, China). For transient transfection, A549 cells were seeded in 6-well plates at a density of 2 × 10^5^ cells per well and transfected with 2.5 μg of *LGALS7* plasmid or empty vector control using Lipofectamine 3000 (Catalog # L3000015, Invitrogen) according to the manufacturer’s instructions. Culture medium was replaced 6 h post-transfection, and cells were harvested 48 h later for subsequent experiments.

### Multiplex immunofluorescence for TEMRA detection

Multiplex immunofluorescence staining was performed using a 7-color fluorescence immunohistochemistry kit (PhenoVision Bio) following manufacturer’s protocol. In short, tissue sections were deparaffinized, rehydrated, and transferred into citrate buffer pH6.0 for antigenic repair. The sections were then washed and incubated with H_2_O_2_ and blocking reagents for 10 min, respectively. Primary antibody was applied on each section for 30 min at room temperature (RT). Bound primary antibodies were detected using PVB anti-Rb/Mm-HRP detection followed by PVB tyramide signal amplification fluorophore for 10 min. The slides were counterstained with spectral 4’,6-diamidino-2-phenylindole (PhenoVision Bio) and cover-slipped.

### Quantitative real-time PCR (qRT-PCR)

Total RNA was isolated using the RNAeasy™ Animal RNA Isolation Kit with Spin Column (Catalog # R0026, Beyotime) according to the manufacturer’s protocol. Genomic DNA removal and reverse transcription were performed using the Fast All-in-One RT Kit with gDNA Remover (Catalog # RT001, ES Science). qRT-PCR was conducted with ChamQ SYBR qPCR Master Mix (Catalog # Q331, Vazyme) on a LightCycler® 480 II system (Roche). GAPDH served as the internal control, and relative gene expression was calculated using the 2^ − ΔΔCt method. Primer sequences are provided in Supplementary Table 12.

### Western blotting

Total protein was extracted from mouse lung tissues or cells using RIPA lysis buffer (Catalog # PC101, Epizyme) supplemented with protease and phosphatase inhibitor (Catalog # P1045, Beyotime). Protein concentration was determined using a BCA Protein Assay Kit (Catalog # P0012S, Beyotime). Equal amounts of protein (30–50 µg) were separated by 10% SDS-PAGE and transferred onto PVDF membranes. After blocking with 5% non-fat milk in TBST for 1 h at room temperature, membranes were incubated overnight at 4 °C with primary antibodies against p-STAT5 (Tyr694, Catalog # 9314S, CST, 1:1000), STAT5 (Catalog # 94,205, CST, 1:1000), p-JAK1 (Tyr1034/1035, Catalog # 74129S, CST, 1:1000), JAK1 (Catalog # 3344S, CST, 1:1000), p-JAK3 (Tyr980/981, Catalog # 5031S, CST, 1:1000), JAK3 (Catalog # 8827S, CST, 1:1000), IL-7Rα/CD127 (Catalog # 17,626–1-AP, Proteintech, 1:1000), and β-actin (Catalog # GB15001, Servicebio, 1:5000) as loading control. Membranes were then incubated with HRP-conjugated goat anti-mouse IgG (Catalog # SA00001-1, Proteintech, 1:5000) or HRP-conjugated goat anti-rabbit IgG (Catalog # SA00001-2, Proteintech, 1:5000). Protein bands were detected using SuperPico ECL Chemiluminescence Kit (Catalog # E422-01, Vazyme) and visualized with a chemiluminescence imaging system (Bio-Rad).

### Enzyme-linked immunosorbent assay (ELISA)

Cell culture supernatants were collected and centrifuged at 1,000 × g for 10 min to remove debris. Levels of Galectin-7, GZMH, GZMA, and perforin were measured using commercial ELISA kits (R&D Systems) according to the manufacturer’s instructions. Absorbance was read at 450 nm using a microplate reader.

### Cellular thermal shift assay (CETSA)

Briefly, HEK293 cell lysates were incubated with 100 μM neoeriocitrin or vehicle control (PBS) for 2 h at room temperature. Aliquots (50μL) were heated at indicated temperatures (45–66 °C) for 3 min, cooled for 3 min at room temperature, and centrifuged at 12,000 rpm for 10 min at 4 °C. Supernatants were collected, mixed with reducing loading buffer, and boiled at 95 °C for 10 min. Soluble Galectin-7 protein levels were detected by western blotting using anti-Galectin-7 antibody (Catalog # 83,267, Cell Signaling, 1:3000). Thermal denaturation curves were generated by quantifying band intensities relative to the lowest temperature (45 °C).

### Construction of human alveolar organoid

The single-cell suspensions were labeled with anti-CD45-BV421 (Catalog # 304,032, BioLegend), anti-CD31-BV421 (Catalog # 303,124, BioLegend), anti-CD326-AF647 (Catalog # 324,212, BioLegend), anti-HTII-280 (Catalog # 303,118, Terrace Biotech Terrace Biotech), and anti-mouse IgM-AF488 (Catalog # A-21042, Thermo Fisher, 1:1,000). Doublets and dead cells were excluded based on forward and side scatters and 7-AAD. The AT2 were sorted as live/CD45^−^/CD31^−^/CD32^+^/HTII-280^+^ cells via Sony MA900 flow cytometer (SONY). The organoid culture system of pathological basal cells was described in a previous study [22].

### T-cell detection and sorting via flow cytometry

The peripheral blood samples were stained and processed for erythrocyte lysis. For tissue samples, we used the digestive system mentioned earlier, but Dispase II was excluded. After FcR blocking, the single-cell suspensions were incubated with anti-CD45-AF488 (Catalog # 304,017, BioLegend), anti-CD3-BV510 (Catalog # 300,448, BioLegend), anti-CD4-BV605 (Catalog # 300,556, BioLegend), anti-CD45RA-PE (Catalog # 304,107, BioLegend), and anti-CCR7-AF647 (Catalog # 353,218, BioLegend) for 30 min at 4 °C. Doublets and dead cells were excluded based on forward and side scatters and 7-AAD. The CD4^+^ TEMRA were detected as live/CD45^+^/CD3^+^/CD4^+^/CD45RA^+^/CCR7^−^ cells via a 3-laser URIT BF-730 flow cytometer (URIT Medical Electronic).

### Mouse model of pulmonary fibrosis​​

Male C57BL/6 J mice (aged 8–10 weeks, weighing 26–30 g) were purchased from the Laboratory Animal Center of Zhejiang University (Hangzhou, China). Mice were housed in a specific pathogen-free environment under controlled temperature (22–24 °C) and humidity (50–60%), with a 12-h light/dark cycle and ad libitum access to standard laboratory chow and sterile water. Animal experiments were approved separately by the Ethics Committee of the Second Affiliated Hospital of Zhejiang University (AIRB-2025–2628). The pulmonary fibrosis was induced via intratracheal administration of bleomycin (2.5 U/kg, Catalog # B802467, Macklin); the blank control group received an equal volume of saline. To evaluate the in vivo efficacy of neoeriocitrin, an AAV-Lung3-mediated *Lgals7*-overexpression mouse model of pulmonary fibrosis was established as described above. Starting on day 10 post-bleomycin instillation, mice received aerosolized pirfenidone (50 mg/kg) or neoeriocitrin (10 mg/kg) via a micro-sprayer (Yuyan Instruments) every 72 h (three times per week). The doses were normalized to body weight and administered intratracheally under brief isoflurane anesthesia. The vehicle control group received an equal volume of sterile PBS. All mice were sacrificed on day 21 post-bleomycin, and lung tissues were collected for histological analysis.

### Immunofluorescence staining​​

Paraffin-embedded lung sections were deparaffinized and rehydrated. OCT-embedded slides were fixed with 4% PFA. Antigen retrieval was performed on all slides. After blocking, sections were incubated overnight at 4 °C with primary antibodies: rabbit anti-Galectin-7 and chicken anti-Keratin 5. Following washes, sections were incubated with fluorescently labeled secondary antibodies (Alexa Fluor 647 and CY3) for 1 h at room temperature. Nuclei were counterstained with DAPI, and slides were mounted for imaging.

### Virtual screening

The crystal structure of human Galectin-7 (PDB ID: 7XAC) was retrieved from the Protein Data Bank and prepared using the Protein Preparation Wizard module in Schrödinger (Schrödinger, LLC, New York, NY, 2021). Bond orders were assigned, hydrogen atoms were added, disulfide bonds were allocated, and protonation states were predicted at pH 7.0 using PROPKA. The structure was refined with restrained energy minimization using the OPLS4 force field, converging heavy-atom RMSD to 0.3 Å, to resolve steric clashes and optimize side-chain conformations.

A library of approximately 20,000 bioactive compounds (TargetMol Bioactive Compound Library) was prepared using the LigPrep module (Schrödinger, LLC, New York, NY, 2021). Protonation states were generated with Epik at pH 7.0 ± 2.0, salts were removed, tautomers were enumerated, and up to 32 stereoisomers and conformers per molecule were generated to ensure adequate conformational sampling.

Molecular docking was performed using Glide (Schrödinger) in standard precision mode. The docking grid was centered on the ligand-binding pocket of Galectin-7, defined based on the co-crystallized ligand position. Initial docking yielded 1,516 ligand–pose complexes. The reliability of the scoring function was assessed by examining the correlation between docking score and molecular weight, which showed a weak linear correlation (R^2^ = 0.18), indicating that favorable scores were not driven by molecular size alone.

To refine the hit list, protein–ligand interaction fingerprint analysis was performed to identify key interacting residues, including GLU12, ARG15, ILE135, PHE136, and GLN106. Redundant poses were removed, retaining 590 unique molecules. These were further filtered by MM-GBSA binding free energy (ΔGbind < − 40 kcal/mol), yielding 359 candidates. Structural diversity analysis using MOE software with a 70% similarity threshold grouped these into 120 clusters, and the top-scoring molecule from each cluster was retained. Finally, physicochemical properties and drug-likeness were evaluated using ADMETlab 3.0, applying Lipinski’s rule of five, Pfizer and GSK filters, and PAINS alerts. This multi-step filtering strategy ensured the selection of structurally diverse, drug-like candidates with favorable predicted ADMET profiles.

### MST assay​​

Binding affinity between Galectin-7 and candidate ligands was quantified by MST. Galectin-7 was labeled with AF647 and used at 20 nM. A 16-point, 1:2 serial dilution of each ligand (from 25 µM) was prepared. Ligand and protein solutions were mixed 1:1, incubated for 15 min, and loaded into standard capillaries. MST measurements were performed with optimized laser power and temperature settings. Dose–response curves were fitted to determine Kd.

### PCLS and drug treatment

Lung parenchyma with relatively well-inflated regions was preferentially selected. Tissue cores were excised using a sterile 1 cm diameter manual skin biopsy punch. The tissue core was immediately embedded in 2% low-melting-point agarose (Catalog # A0576, Sigma-Aldrich) pre-warmed to 37 °C and cooled on ice using a Compresstome® cooling block until solidification. The agarose-embedded specimen was mounted in a 15.5 mm specimen tube (Catalog # VF-SPS-VM-15.5-BOS, Precisionary Instruments) and sectioned at 500 µm thickness using a Compresstome® VF-510-OZ vibratome (Precisionary Instrument) with the following parameters: advance speed 1, oscillation frequency 8 and a fresh double-edge stainless-steel blade (Catalog # VF-BL-VM-SSB, Personna). Slices were collected in ice-cold PBS supplemented with penicillin–streptomycin and allowed to recover for 1 h at 37 °C in a 5% CO₂ incubator.

Viable slices were transferred to 24-well plates (one slice per well) containing Small Airway Growth Medium (Catalog # CC-3110, SAGM, Lonza) supplemented with 1% penicillin–streptomycin and incubated at 37 °C under 5% CO₂. Culture medium was changed every 2 days. For drug treatment experiments, PCLS were incubated with neoeriocitrin (10 µM) or vehicle control (DMSO ≤ 0.1%) for 7 days, with medium and drug refreshed every 2 days. At the indicated time points, slice viability was assessed using the Cell Counting Kit‑8 (CCK8, Catalog # CK04, Dojindo) assay. Briefly, PCLS were transferred to fresh medium containing 10% CCK‑8 reagent and incubated at 37 °C for 2 h. Absorbance was measured at 450 nm using a microplate reader (Bio‑Tek, Synergy H1). The PCLS maintained in Small Airway Growth Medium remained viable for extended periods in vitro, thereby supporting downstream experimental assays. (Fig. S6b).

### Statistical analysis

All statistical analyses and data visualizations were performed using R (version 4.2.1) and Python software (version 3.8.16). Pearson’s correlation coefficients were employed to calculate correlations between two continuous variables. For quantitative data, the unpaired-sample Student *t* test was used to compare values between subgroups. A *P* value < 0.05 indicated a statistically significant difference.

## Supplementary Information

Supplementary Material 1.

## Data Availability

The human RNA-seq data that support the findings of this study have been published in the GEO under the following accession codes: GSE47460 (lung tissue), GSE150068 (organoids), GSE38958 (PBMCs), GSE33566 (PBMCs), GSE28042 (PBMCs with prognostic information), GSE27957 (PBMCs with prognostic information), GSE132771 (mesenchymal cells), and GSE146981 (epithelial cells). 10 × Visium spatial data of IPF were retrieved from the BioStudies under accession numbers S-BSST1410 and were processed with Seurat. Raw sequencing data of the WUXI cohort will be available upon peer-reviewed publication and had been updated to National Genomics Data Center with id HRA012369.
