# Supplementary material for: Targeting the CD4+ effector memory T cells re-expressing CD45RA suppressor galectin-7: a multi-omics-guided therapeutic strategy for idiopathic pulmonary fibrosis
Source: Mol Biomed. 2026 Oct 5;7:196. doi: 10.1186/s43556-026-00542-0 (PMC13635077; doi:10.1186/s43556-026-00542-0)
Supplement: Supplementary file 1 [file 43556_2026_542_MOESM1_ESM.pdf]

Supplementary Information

Targeting the CD4$^+$ effector memory T cells re-expressing CD45RA Suppressor Galectin-7: A Multi-Omics-Guided Therapeutic Strategy for Idiopathic Pulmonary Fibrosis

Tao Yan[1,4,11]†, Jichang Liu[2]†, Yong Liu[2]†, Wenchao Gu[3], Jia Liu[2], Yuanshu Li[4], Rongrong Lv[5], Chuanpeng Zhang[2], Jiaqi Ding[12], Chenlong Zha[2], Chaoqun Wang[4,5,6,7]*, Fei Gao[8]*, Man Huang[9,10]*, Jingyu Chen[1,2,11,12]*

1 Department of Lung Transplantation, The Second Affiliated Hospital, Zhejiang University School of Medicine, Hangzhou 310000, China.

2 Lung Transplantation Center, The Affiliated Wuxi People's Hospital of Nanjing Medical University, Wuxi Medical Center, Nanjing Medical University, Wuxi 214023, China.

3 Department of Artificial Intelligence Medicine, Graduate School of Medicine, Chiba University, Chiba, Japan.

4 Zhongshan Institute for Drug Discovery, Shanghai Institute of Materia Medica, Chinese Academy of Sciences, Zhongshan 528400, China.

5 Shanghai Institute of Materia Medica, Chinese Academy of Sciences, Shanghai 201203, China.

6 State Key Laboratory of Drug Research, Shanghai Institute of Materia Medica, Chinese Academy of Sciences, Shanghai 201203, China.

7 University of Chinese Academy of Sciences, Beijing 100049, China.

8 Department of Emergency, The Affiliated Wuxi People's Hospital of Nanjing Medical University, Wuxi Medical Center, Nanjing Medical University, Wuxi 214023, China.

9 Department of General Intensive Care Unit, The Second Affiliated Hospital, Zhejiang University School of Medicine, Hangzhou 310052, China.

10 Key Laboratory of Multiple Organ Failure (Zhejiang University), Ministry of Education, Zhejiang, China.

11 Jingyu Chen Lung Transplantation Laboratory, The Second Affiliated Hospital Zhejiang University School of Medicine, Hangzhou, 310000, China.
12 Department of Thoracic Surgery, Affiliated Hospital of Qingdao University, Qingdao, China.

*Corresponding authors: Chaoqun Wang (cqwang@simm.ac.cn); Fei Gao (gaofeimed@126.com); Man Huang (huangman@zju.edu.cn); Jingyu Chen (chenjy@wuxiph.com)
†These authors contributed equally to this work.
The authors have declared that no conflict of interest exists.

a

id.exposure	nsnp	method	pval	OR(95% CI)
CD4+Temra %T cell_Discovery	2	IVW−mre	**0.004**	0.712 (0.565 to 0.896)
CD4+Temra %T cell_Validation	2	IVW−mre	**<0.001**	0.452 (0.397 to 0.515)
CD33 on Granulocytic MDSCs_Discovery	3	IVW−mre	**<0.001**	1.019 (1.012 to 1.026)
	3	MR Egger	0.749	1.026 (0.908 to 1.159)
	3	Simple mode	0.790	1.015 (0.920 to 1.121)
	3	Weighted median	0.538	1.020 (0.958 to 1.086)
	3	Weighted mode	0.616	1.021 (0.954 to 1.092)
CD33 on Granulocytic MDSCs_Validation	3	IVW−mre	**0.002**	0.922 (0.876 to 0.970)
	3	MR Egger	0.485	0.899 (0.736 to 1.097)
	3	Simple mode	0.377	0.907 (0.765 to 1.075)
	3	Weighted median	0.101	0.915 (0.822 to 1.018)
	3	Weighted mode	0.257	0.914 (0.817 to 1.023)
CD33 on Monocytic MDSCs_Discovery	3	IVW−mre	**<0.001**	1.018 (1.011 to 1.026)
	3	MR Egger	0.656	1.026 (0.944 to 1.115)
	3	Simple mode	0.936	1.004 (0.925 to 1.089)
	3	Weighted median	0.437	1.019 (0.972 to 1.067)
	3	Weighted mode	0.500	1.020 (0.972 to 1.071)
CD33 on Monocytic MDSCs_Validation	3	IVW−mre	**<0.001**	0.932 (0.895 to 0.970)
	3	MR Egger	0.410	0.911 (0.794 to 1.045)
	3	Simple mode	0.399	0.926 (0.804 to 1.067)
	3	Weighted median	0.059	0.925 (0.854 to 1.003)
	3	Weighted mode	0.207	0.925 (0.852 to 1.005)
CCR2 on monocyte_Discovery	2	IVW−mre	**<0.001**	1.046 (1.022 to 1.071)
CCR2 on monocyte_Validation	2	IVW−mre	**0.002**	0.853 (0.771 to 0.943)

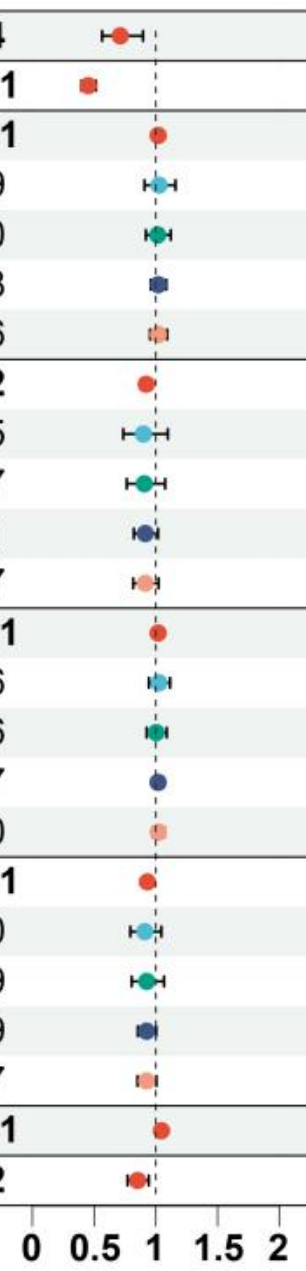

Supplementary figure 1 (a) The forest plot showed the common four immune traits with causal association with IPF in the discovery and validation cohorts.

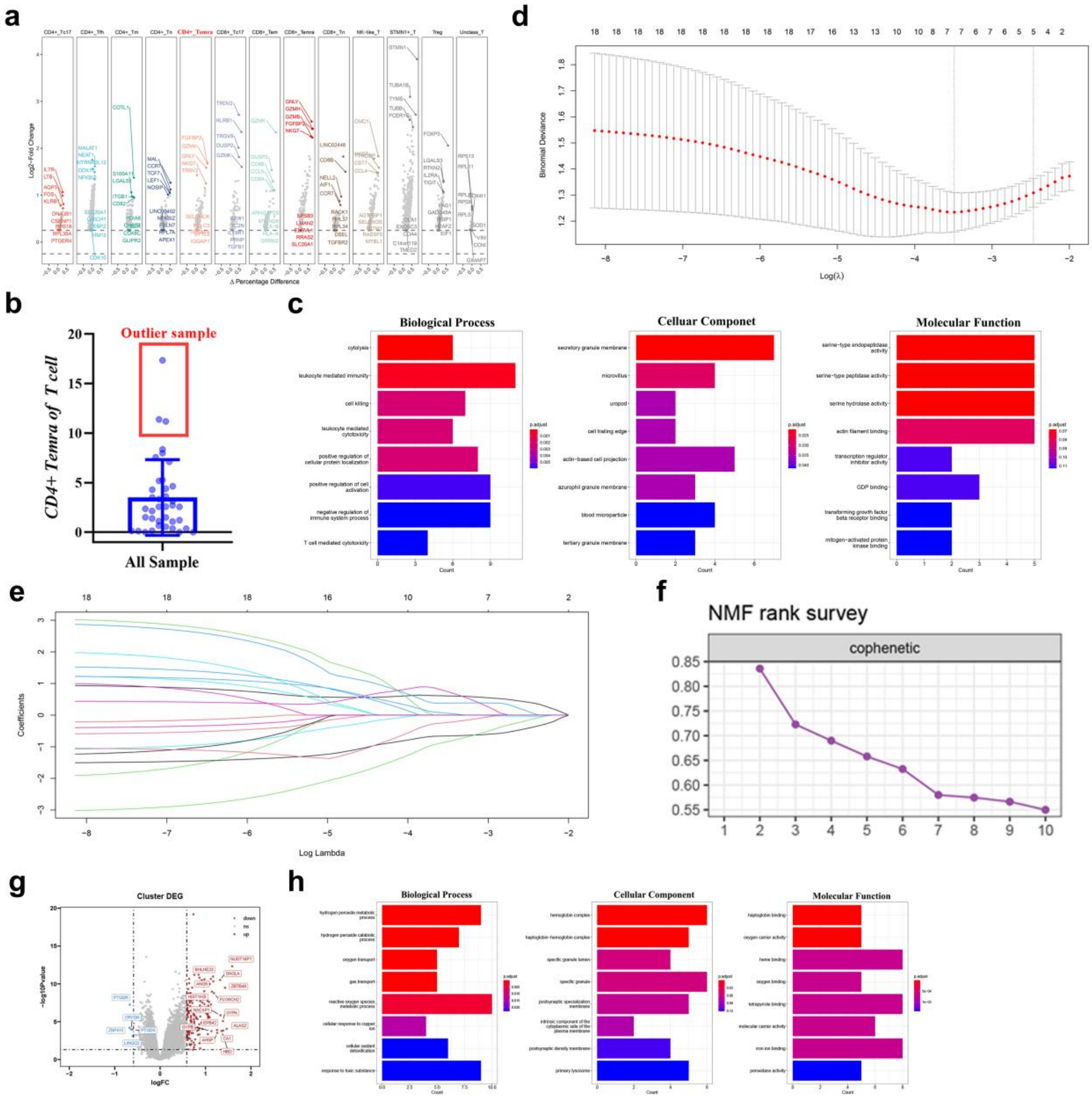

Supplementary figure 2 (a) The high-expressed gene sets of T cell subtypes. (b) Show the outliers of the proportion of $CD4^+$ TEMRA in patients with IPF. (c) Carried out GO enrichment analysis based on the red module genes. (d) The cross-validation curve of LASSO regression indicates that when the number of variables is 7, the model achieves optimal fit. (e) The LASSO regression coefficient path diagram shows that as the horizontal coordinate log Lambda increases, the vertical coordinate value of the regression coefficient continuously converges and eventually converges to 0. (f) Based on the cophenetic coefficient of the NMF clustering parameters, which is the co-expression correlation coefficient, the optimal number of subgroups is selected as 2. (g) By using subtype 1 as the reference for DEG analysis, a total of 5 down-regulated genes and 152 up-regulated genes were identified (h) The biological process module indicated that the DEGs were significantly enriched in the ROS metabolic pathway, while the other modules all suggested associations with oxygen metabolism.

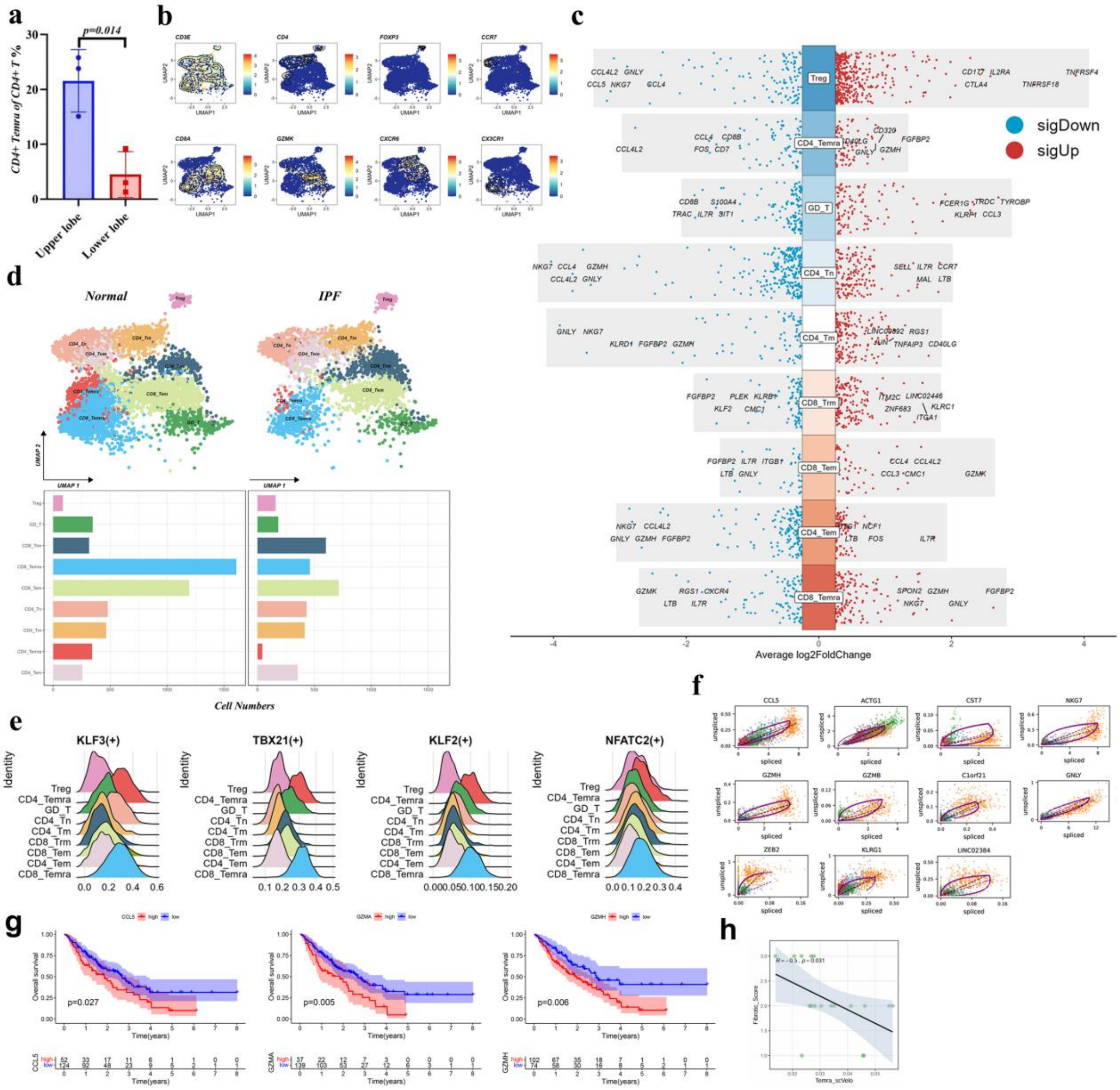

Supplementary figure 3 (a) The quantitative image analysis of mIHC. (b) Contour maps display the characteristic marker expression of 9 T cell subpopulations. (c) Differentially expressed genes characteristic of each T cell subpopulation are displayed, with red and blue dots representing significantly upregulated and downregulated genes, respectively. (d) The infiltrating differences of 9 T cell subpopulations between IPF and normal lung tissues were compared. (e) SCENIC analysis identified transcription factors characterized in each T cell subpopulation. KLF3, TBX21, KLF2 and NFATC2 were highly expressed and might act as key driver genes for the $CD4^{+}$ TEMRA subpopulation. (f) The top 15 genes contributing most strongly to $CD4^{+}$ TEMRA in normal lung tissues. (g) Kaplan-Meier survival analyses of IPF patients with high and low expression of CCL5 (left), GZMA (middle) and GZMH (right). Log-rank test was used to detect the statistical significance. (h) The correlation analysis revealed that the gene set promoting TEMRA differentiation was significantly negatively correlated with the expression level of ACTA2.

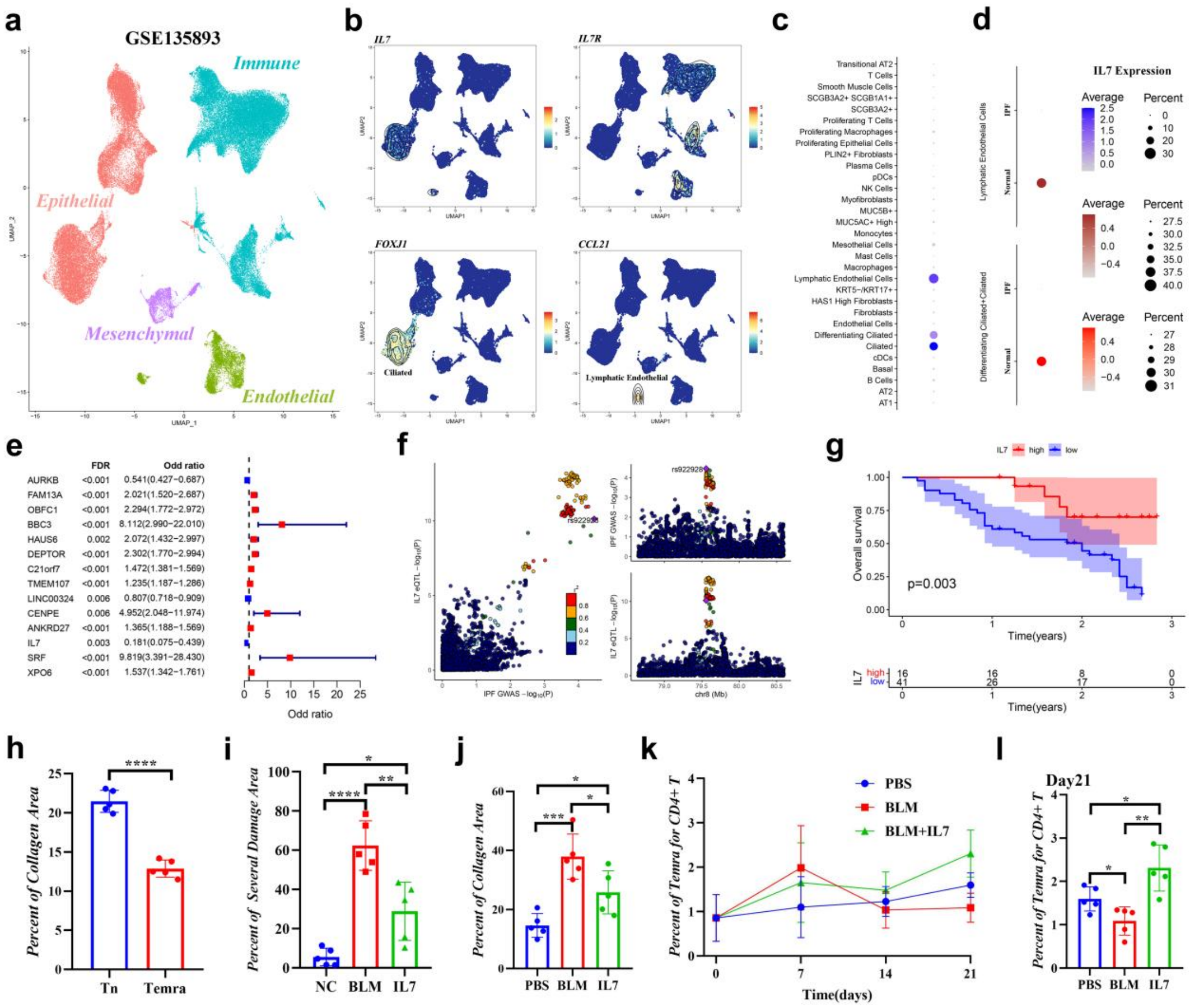

Supplementary figure 4 (a) The single-cell RNA sequencing data identified the epithelial, mesenchymal, immune and endothelial lineages in lung tissues from IPF patients and normal subjects. (b) Contour map shows that the expression of *IL7* is concentrated in ciliated cells and lymphatic endothelial cells. (c) Distribution of IL-7 expression in overall cell subgroups via dot plot. (d) Differences in IL-7 expression in lymphatic endothelial cells between IPF and normal groups. (e) MR analysis for screening genes related to the onset of IPF (f) A high posterior probability for hypothesis 4 (PPH4 = 0.83) indicates that the Mendelian randomization association is unlikely to be confounded by linkage disequilibrium. (g) High expression of *IL7* in peripheral blood indicates a favorable prognosis. (h) Quantitative results of CVF for PCLS co-culture with Tn and Temra. (i) The LungDamage algorithm calculates the areas of lung fibrosis damage in mice. (j) Quantitative results of CVF in the three groups (all n = 5). PBS, control group; BLM, fibrosis model group; BLM+IL7, treatment group. (k) Changes in the proportion of $CD4^+$ TEMRA in peripheral blood of the three groups of mice from 0 to 21 days. (l) At 21 days, the proportion of $CD4^+$ TEMRA in the treatment group and the control group was significantly higher than that in the fibrosis model group.

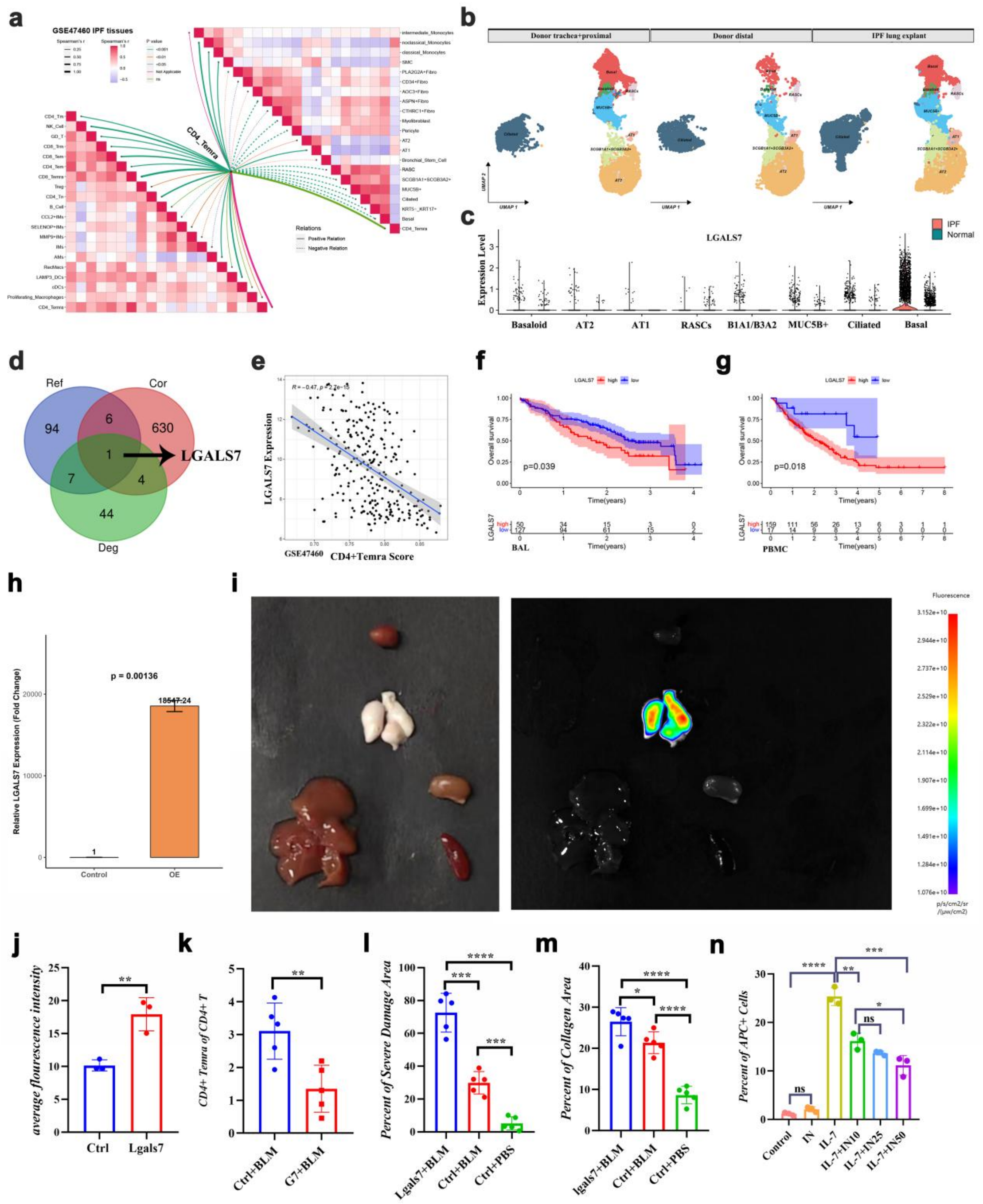

Supplementary figure 5 (a) ssGSEA-based deconvolution of scRNA-seq profiles revealed a disease-specific inverse association between basal cells and CD4^{+} TEMRA cells in IPF, which was absent in normal lung tissue. (b) UMAP dimensional plot and histogram displayed the epithelial cell subpopulations in donor proximal airway, distal lung and IPF distal lung tissues. (c) *LGALS7* is specifically highly expressed in the basal cells of IPF. (d) Venn plot showed that *LGALS7* was only candidate between Cor (negative correlation between CD4^{+} TEMRA abundance), Deg (differentially expressed genes set of basal cell between IPF and normal lung) and Ref (The differentially expressed gene set between the AT2 differentiated basal cells in the previously reported organoid models and the normal human lung basal cells).(e) Correlation of *LGALS7* and CD4^{+} TEMRA abundance calculated via ssGSEA in the

IPF lung tissues (GSE47460). (f) Kaplan-Meier survival analyses of IPF patients with high- and low-*LGALS7* expression in bronchoalveolar lavage fluid and PBMCs (g) and was tested by log-rank test. (h) PCR test the upregulation of *LGALS7* of A549 after plasmid transfection. (i) Compare the fluorescence intensities of the heart, liver, spleen, lung and kidney in mice, and verify the specific expression of Lgals7 in the mouse lungs. (j) Compare the fluorescence intensities of Galectin-7 between *Lgals7*-overexpression and control group. (k) Flow cytometry was used to examine the changes in the proportion of $CD4^+$ TEMRA in the peripheral blood of mice (sampled from the orbital vein). Galectin-7 significantly decreased the proportion of $CD4^+$ TEMRA. (l) The collagen deposition in each group was observed. (m) Quantitative analysis showed that the CVF in the *Lgals7*+BLM group was significantly higher than that in the other two groups. NC+PBS, control group treated with empty virus and PBS; G7+BLM, fibrosis model with overexpressed gene; NC+BLM, fibrosis model treated with empty virus. (n) Quantitative analysis of $CD4^+$ TEMRA percent of control group, only STAT5-IN-1 treatment, IL-7 treat and IL-7 treat with 10 μM, 25 μM and 50 μM.

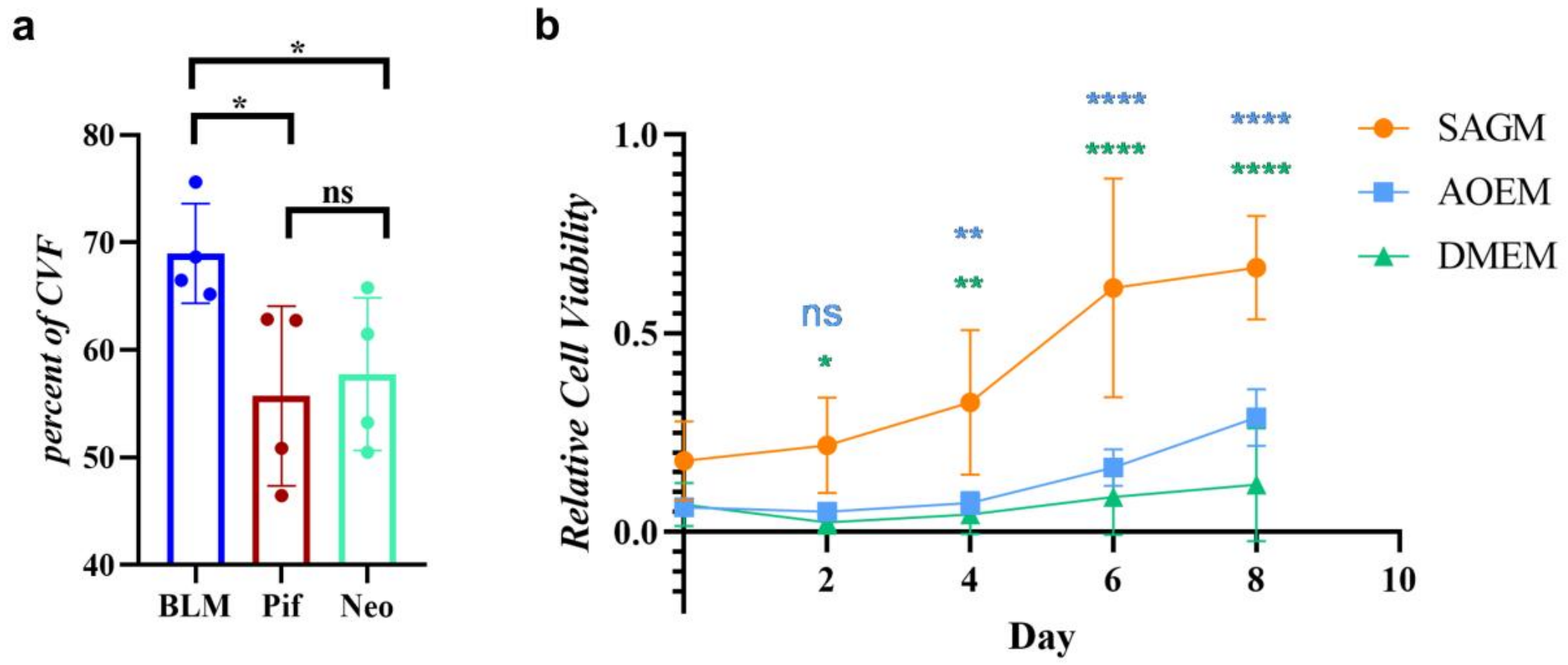

Supplementary figure 6 (a) Quantitative analysis of Masson's trichrome staining data across different treatment groups. BLM (Bleomycin model, 69.24 ± 2.65), Pif (Pirfenidone treatment, 55.72 ± 4.11), and Neo (neoeriocitrin treatment, 57.52 ± 3.83). (b) CCK-8 assay showing PCLS viability under indicated culture conditions.

Supplementary Table 1. Source of exposure and outcome data
Supplementary Table 2. Instrumental varibales of immune traits used for MR analysis after
Supplementary Table 3. Mendelian randomization results for the association between immune traits
Supplementary Table 4. Steiger filtering results for the association between immune traits and IPF risk
Supplementary Table 5. Instrumental varibales of immune traits used for MR analysis after
Supplementary Table 6. Mendelian randomization results for the association between immune traits
Supplementary Table 7. Steiger filtering results for the association between immune traits and IPF risk
Supplementary Table 8. Reverse mendelian randomization results for the association between IPF and
Supplementary Table 9. Previously-reported genome-wide significant association of SNPs as genetic
Supplementary Table 10. The detailed characteristics of 120 small molecule compounds obtained
Supplementary Table 11. Clinical information of donor and IPF patients involved in scRNA-seq and

Supplementary Table 1

Traits	Accession numbers/Source	Population
731 immune traits	GCST0001391-GCST0002121	European
IPF (Discovery cohort)	The International IPF Genetics Consortium	European
IPF (Validation cohort)	finn-b-IPF	European

Reference	Case	Control	Total	Variants
Valeria et al., 2020	NA	NA	up to 3,757	up to 22 million
Richard et al. 2022	4,125	20,464	24,589	7,554,248
NA	1,028	196,986	198,014	16,380,413

Supplementary Table 2

Exposure	Id.exposure	Types	Outcome
FSC-A on Natural Killer	GCST90001969	blood protein measurement	IPF
FSC-A on Natural Killer	GCST90001969	blood protein measurement	IPF
FSC-A on Natural Killer	GCST90001969	blood protein measurement	IPF
SSC-A on Natural Killer	GCST90002076	blood protein measurement	IPF
CD20 on IgD+ CD38dim B cell	GCST90001752	blood protein measurement	IPF
CD20 on IgD+ CD38dim B cell	GCST90001752	blood protein measurement	IPF
CD20 on IgD+ CD38dim B cell	GCST90001752	blood protein measurement	IPF
CD20 on IgD+ CD38dim B cell	GCST90001752	blood protein measurement	IPF
CD20 on IgD+ CD38dim B cell	GCST90001752	blood protein measurement	IPF
CD20 on IgD+ CD38dim B cell	GCST90001752	blood protein measurement	IPF
CD45RA+ CD28- CD8+ T cell %T cell	GCST90001700	lymphocyte count	IPF
CD45RA+ CD28- CD8+ T cell %T cell	GCST90001700	lymphocyte count	IPF
CD45RA+ CD28- CD8+ T cell %T cell	GCST90001700	lymphocyte count	IPF
CD45RA+ CD28- CD8+ T cell %T cell	GCST90001700	lymphocyte count	IPF
CD45RA+ CD28- CD8+ T cell %T cell	GCST90001700	lymphocyte count	IPF
CD45RA+ CD28- CD8+ T cell %T cell	GCST90001700	lymphocyte count	IPF
CD45RA+ CD28- CD8+ T cell %T cell	GCST90001700	lymphocyte count	IPF
CD45RA+ CD28- CD8+ T cell %T cell	GCST90001700	lymphocyte count	IPF
CD45RA+ CD28- CD8+ T cell %T cell	GCST90001700	lymphocyte count	IPF
CD45RA+ CD28- CD8+ T cell %T cell	GCST90001700	lymphocyte count	IPF
CD45RA+ CD28- CD8+ T cell %T cell	GCST90001700	lymphocyte count	IPF
CD45RA+ CD28- CD8+ T cell %T cell	GCST90001700	lymphocyte count	IPF
CD45RA+ CD28- CD8+ T cell %T cell	GCST90001700	lymphocyte count	IPF
CD45RA+ CD28- CD8+ T cell %T cell	GCST90001700	lymphocyte count	IPF
CD45RA+ CD28- CD8+ T cell %T cell	GCST90001700	lymphocyte count	IPF
CD45RA+ CD28- CD8+ T cell %T cell	GCST90001700	lymphocyte count	IPF
CD45RA+ CD28- CD8+ T cell %T cell	GCST90001700	lymphocyte count	IPF
CD45RA+ CD28- CD8+ T cell %T cell	GCST90001700	lymphocyte count	IPF
CD45RA+ CD28- CD8+ T cell %T cell	GCST90001700	lymphocyte count	IPF
CD45RA+ CD28- CD8+ T cell %T cell	GCST90001700	lymphocyte count	IPF
CD45RA+ CD28- CD8+ T cell %T cell	GCST90001700	lymphocyte count	IPF
CD45RA+ CD28- CD8+ T cell %T cell	GCST90001700	lymphocyte count	IPF
CD45RA+ CD28- CD8+ T cell %T cell	GCST90001700	lymphocyte count	IPF
CD45RA+ CD28- CD8+ T cell %T cell	GCST90001700	lymphocyte count	IPF
CD45RA+ CD28- CD8+ T cell %T cell	GCST90001700	lymphocyte count	IPF
CD45RA+ CD28- CD8+ T cell %T cell	GCST90001700	lymphocyte count	IPF
CD45RA+ CD28- CD8+ T cell %T cell	GCST90001700	lymphocyte count	IPF
CD45RA+ CD28- CD8+ T cell %T cell	GCST90001700	lymphocyte count	IPF
CD45RA+ CD28- CD8+ T cell %T cell	GCST90001700	lymphocyte count	IPF
CD45RA+ CD28- CD8+ T cell %T cell	GCST90001700	lymphocyte count	IPF
CD45RA+ CD28- CD8+ T cell %T cell	GCST90001700	lymphocyte count	IPF
CD45RA+ CD28- CD8+ T cell %T cell	GCST90001700	lymphocyte count	IPF
CD45RA+ CD28- CD8+ T cell %T cell	GCST90001700	lymphocyte count	IPF
CD45RA+ CD28- CD8+ T cell %T cell	GCST90001700	lymphocyte count	IPF
CD45RA+ CD28- CD8+ T cell %T cell	GCST90001700	lymphocyte count	IPF
CD45RA+ CD28- CD8+ T cell %T cell	GCST90001700	lymphocyte count	IPF
Naive CD8+ T cell %T cell	GCST90001553	lymphocyte count	IPF
Naive CD8+ T cell %T cell	GCST90001553	lymphocyte count	IPF
Naive CD8+ T cell %T cell	GCST90001553	lymphocyte count	IPF
IgD on transitional B cell	GCST90001828	blood protein measurement	IPF
IgD on transitional B cell	GCST90001828	blood protein measurement	IPF
IgD on transitional B cell	GCST90001828	blood protein measurement	IPF
SSC-A on Natural Killer T	GCST90002084	blood protein measurement	IPF
SSC-A on Natural Killer T	GCST90002084	blood protein measurement	IPF
CD19 on CD20- CD38- B cell	GCST90001722	blood protein measurement	IPF
CD45RA+ CD28- CD8+ T cell Absolute	GCST90001698	lymphocyte count	IPF

CD45RA+ CD28- CD8+ T cell Absolute	GCST90001698	lymphocyte count	IPF
CD45RA+ CD28- CD8+ T cell Absolute	GCST90001698	lymphocyte count	IPF
CD45RA+ CD28- CD8+ T cell Absolute	GCST90001698	lymphocyte count	IPF
CD45RA+ CD28- CD8+ T cell Absolute	GCST90001698	lymphocyte count	IPF
CD45RA+ CD28- CD8+ T cell Absolute	GCST90001698	lymphocyte count	IPF
CD45RA+ CD28- CD8+ T cell Absolute	GCST90001698	lymphocyte count	IPF
CD45RA+ CD28- CD8+ T cell Absolute	GCST90001698	lymphocyte count	IPF
CD45RA+ CD28- CD8+ T cell Absolute	GCST90001698	lymphocyte count	IPF
CD45RA+ CD28- CD8+ T cell Absolute	GCST90001698	lymphocyte count	IPF
CD45RA+ CD28- CD8+ T cell Absolute	GCST90001698	lymphocyte count	IPF
CD45RA+ CD28- CD8+ T cell Absolute	GCST90001698	lymphocyte count	IPF
CD45RA+ CD28- CD8+ T cell Absolute	GCST90001698	lymphocyte count	IPF
CD45RA+ CD28- CD8+ T cell Absolute	GCST90001698	lymphocyte count	IPF
CD45RA+ CD28- CD8+ T cell Absolute	GCST90001698	lymphocyte count	IPF
CD45RA+ CD28- CD8+ T cell Absolute	GCST90001698	lymphocyte count	IPF
CD45RA+ CD28- CD8+ T cell Absolute	GCST90001698	lymphocyte count	IPF
CD45RA+ CD28- CD8+ T cell Absolute	GCST90001698	lymphocyte count	IPF
CD45RA+ CD28- CD8+ T cell Absolute	GCST90001698	lymphocyte count	IPF
CD45RA+ CD28- CD8+ T cell Absolute	GCST90001698	lymphocyte count	IPF
CD45RA+ CD28- CD8+ T cell Absolute	GCST90001698	lymphocyte count	IPF
CD45RA+ CD28- CD8+ T cell Absolute	GCST90001698	lymphocyte count	IPF
CD45RA+ CD28- CD8+ T cell Absolute	GCST90001698	lymphocyte count	IPF
CD45RA+ CD28- CD8+ T cell Absolute	GCST90001698	lymphocyte count	IPF
CD45RA+ CD28- CD8+ T cell Absolute	GCST90001698	lymphocyte count	IPF
CD45RA+ CD28- CD8+ T cell Absolute	GCST90001698	lymphocyte count	IPF
CD45RA+ CD28- CD8+ T cell Absolute	GCST90001698	lymphocyte count	IPF
CD45RA+ CD28- CD8+ T cell Absolute	GCST90001698	lymphocyte count	IPF
CD45RA+ CD28- CD8+ T cell Absolute	GCST90001698	lymphocyte count	IPF
CD45RA+ CD28- CD8+ T cell Absolute	GCST90001698	lymphocyte count	IPF
CD45RA+ CD28- CD8+ T cell Absolute	GCST90001698	lymphocyte count	IPF
CD45RA+ CD28- CD8+ T cell Absolute	GCST90001698	lymphocyte count	IPF
CD45RA+ CD28- CD8+ T cell Absolute	GCST90001698	lymphocyte count	IPF
CD45RA+ CD28- CD8+ T cell Absolute	GCST90001698	lymphocyte count	IPF
CD45RA+ CD28- CD8+ T cell Absolute	GCST90001698	lymphocyte count	IPF
CD45RA+ CD28- CD8+ T cell Absolute	GCST90001698	lymphocyte count	IPF
CD45RA+ CD28- CD8+ T cell Absolute	GCST90001698	lymphocyte count	IPF
CD45RA+ CD28- CD8+ T cell Absolute	GCST90001698	lymphocyte count	IPF
CD45RA+ CD28- CD8+ T cell Absolute	GCST90001698	lymphocyte count	IPF
CD45RA+ CD28- CD8+ T cell Absolute	GCST90001698	lymphocyte count	IPF
CD45RA+ CD28- CD8+ T cell Absolute	GCST90001698	lymphocyte count	IPF
CD45RA+ CD28- CD8+ T cell Absolute	GCST90001698	lymphocyte count	IPF
CD45RA+ CD28- CD8+ T cell Absolute	GCST90001698	lymphocyte count	IPF
CD45RA+ CD28- CD8+ T cell Absolute	GCST90001698	lymphocyte count	IPF
CD45RA+ CD28- CD8+ T cell Absolute	GCST90001698	lymphocyte count	IPF
CD45RA+ CD28- CD8+ T cell Absolute	GCST90001698	lymphocyte count	IPF
CD45RA+ CD28- CD8+ T cell Absolute	GCST90001698	lymphocyte count	IPF
CD45RA+ CD28- CD8+ T cell Absolute	GCST90001698	lymphocyte count	IPF
CD45RA+ CD28- CD8+ T cell Absolute	GCST90001698	lymphocyte count	IPF
CD45RA+ CD28- CD8+ T cell Absolute	GCST90001698	lymphocyte count	IPF
CD45RA+ CD28- CD8+ T cell Absolute	GCST90001698	lymphocyte count	IPF
CD45RA+ CD28- CD8+ T cell Absolute	GCST90001698	lymphocyte count	IPF
CD45RA+ CD28- CD8+ T cell Absolute	GCST90001698	lymphocyte count	IPF
CD45RA+ CD28- CD8+ T cell Absolute	GCST90001698	lymphocyte count	IPF
CD45RA+ CD28- CD8+ T cell Absolute	GCST90001698	lymphocyte count	IPF
CD45RA+ CD28- CD8+ T cell Absolute	GCST90001698	lymphocyte count	IPF
CD45RA+ CD28- CD8+ T cell Absolute	GCST90001698	lymphocyte count	IPF
CD45RA+ CD28- CD8+ T cell Absolute	GCST90001698	lymphocyte count	IPF
CD45RA+ CD28- CD8+ T cell Absolute	GCST90001698	lymphocyte count	IPF

CD45RA+ CD28- CD8+ T cell Absolute	GCST90001698	lymphocyte count	IPF
CD45RA+ CD28- CD8+ T cell Absolute	GCST90001698	lymphocyte count	IPF
CD45RA+ CD28- CD8+ T cell Absolute	GCST90001698	lymphocyte count	IPF
CD45RA+ CD28- CD8+ T cell Absolute	GCST90001698	lymphocyte count	IPF
CD45RA+ CD28- CD8+ T cell Absolute	GCST90001698	lymphocyte count	IPF
CD45RA+ CD28- CD8+ T cell Absolute	GCST90001698	lymphocyte count	IPF
CD45RA+ CD28- CD8+ T cell Absolute	GCST90001698	lymphocyte count	IPF
CD45RA+ CD28- CD8+ T cell Absolute	GCST90001698	lymphocyte count	IPF
CD45RA+ CD28- CD8+ T cell Absolute	GCST90001698	lymphocyte count	IPF
CD45RA+ CD28- CD8+ T cell Absolute	GCST90001698	lymphocyte count	IPF
CD45RA+ CD28- CD8+ T cell Absolute	GCST90001698	lymphocyte count	IPF
CD45RA+ CD28- CD8+ T cell Absolute	GCST90001698	lymphocyte count	IPF
CD45RA+ CD28- CD8+ T cell Absolute	GCST90001698	lymphocyte count	IPF
CD45RA+ CD28- CD8+ T cell Absolute	GCST90001698	lymphocyte count	IPF
CD45RA+ CD28- CD8+ T cell Absolute	GCST90001698	lymphocyte count	IPF
CD45RA+ CD28- CD8+ T cell Absolute	GCST90001698	lymphocyte count	IPF
CD45RA+ CD28- CD8+ T cell Absolute	GCST90001698	lymphocyte count	IPF
CD45RA+ CD28- CD8+ T cell Absolute	GCST90001698	lymphocyte count	IPF
CD45RA+ CD28- CD8+ T cell Absolute	GCST90001698	lymphocyte count	IPF
CD45RA+ CD28- CD8+ T cell Absolute	GCST90001698	lymphocyte count	IPF
CD45RA+ CD28- CD8+ T cell Absolute	GCST90001698	lymphocyte count	IPF
CD45RA+ CD28- CD8+ T cell Absolute	GCST90001698	lymphocyte count	IPF
CD45RA+ CD28- CD8+ T cell Absolute	GCST90001698	lymphocyte count	IPF
CD45RA+ CD28- CD8+ T cell Absolute	GCST90001698	lymphocyte count	IPF
CD45RA+ CD28- CD8+ T cell Absolute	GCST90001698	lymphocyte count	IPF
CD45RA+ CD28- CD8+ T cell Absolute	GCST90001698	lymphocyte count	IPF
CD45RA+ CD28- CD8+ T cell Absolute	GCST90001698	lymphocyte count	IPF
CD45RA+ CD28- CD8+ T cell Absolute	GCST90001698	lymphocyte count	IPF
CD45RA+ CD28- CD8+ T cell Absolute	GCST90001698	lymphocyte count	IPF
CD45RA+ CD28- CD8+ T cell Absolute	GCST90001698	lymphocyte count	IPF
CD45RA+ CD28- CD8+ T cell Absolute	GCST90001698	lymphocyte count	IPF
CD45RA+ CD28- CD8+ T cell Absolute	GCST90001698	lymphocyte count	IPF
CD45RA+ CD28- CD8+ T cell Absolute	GCST90001698	lymphocyte count	IPF
CD45RA+ CD28- CD8+ T cell Absolute	GCST90001698	lymphocyte count	IPF
CD45RA+ CD28- CD8+ T cell Absolute	GCST90001698	lymphocyte count	IPF
CD45RA+ CD28- CD8+ T cell Absolute	GCST90001698	lymphocyte count	IPF
CD45RA+ CD28- CD8+ T cell Absolute	GCST90001698	lymphocyte count	IPF
CD45RA+ CD28- CD8+ T cell Absolute	GCST90001698	lymphocyte count	IPF
CD45RA+ CD28- CD8+ T cell Absolute	GCST90001698	lymphocyte count	IPF
CD45RA+ CD28- CD8+ T cell Absolute	GCST90001698	lymphocyte count	IPF
CD45RA+ CD28- CD8+ T cell Absolute	GCST90001698	lymphocyte count	IPF
CD45RA+ CD28- CD8+ T cell Absolute	GCST90001698	lymphocyte count	IPF
CD45RA+ CD28- CD8+ T cell Absolute	GCST90001698	lymphocyte count	IPF
CD45RA+ CD28- CD8+ T cell Absolute	GCST90001698	lymphocyte count	IPF
CD45RA+ CD28- CD8+ T cell Absolute	GCST90001698	lymphocyte count	IPF
CD45RA+ CD28- CD8+ T cell Absolute	GCST90001698	lymphocyte count	IPF
CD45RA+ CD28- CD8+ T cell Absolute	GCST90001698	lymphocyte count	IPF
CD45RA+ CD28- CD8+ T cell Absolute	GCST90001698	lymphocyte count	IPF
CD45RA+ CD28- CD8+ T cell Absolute	GCST90001698	lymphocyte count	IPF
CD45RA+ CD28- CD8+ T cell Absolute	GCST90001698	lymphocyte count	IPF
CD45RA+ CD28- CD8+ T cell Absolute	GCST90001698	lymphocyte count	IPF
CD45RA+ CD28- CD8+ T cell Absolute	GCST90001698	lymphocyte count	IPF
CD45RA+ CD28- CD8+ T cell Absolute	GCST90001698	lymphocyte count	IPF
CD45RA+ CD28- CD8+ T cell Absolute	GCST90001698	lymphocyte count	IPF
CD45RA+ CD28- CD8+ T cell Absolute	GCST90001698	lymphocyte count	IPF
CD45RA+ CD28- CD8+ T cell Absolute	GCST90001698	lymphocyte count	IPF
CD45RA+ CD28- CD8+ T cell Absolute	GCST90001698	lymphocyte count	IPF
CD45RA+ CD28- CD8+ T cell Absolute	GCST90001698	lymphocyte count	IPF

CD45RA+ CD28- CD8+ T cell Absolute	GCST90001698	lymphocyte count	IPF
CD45RA+ CD28- CD8+ T cell Absolute	GCST90001698	lymphocyte count	IPF
CD45RA+ CD28- CD8+ T cell Absolute	GCST90001698	lymphocyte count	IPF
CD45RA+ CD28- CD8+ T cell Absolute	GCST90001698	lymphocyte count	IPF
CD45RA+ CD28- CD8+ T cell Absolute	GCST90001698	lymphocyte count	IPF
CD45RA+ CD28- CD8+ T cell Absolute	GCST90001698	lymphocyte count	IPF
CD45RA+ CD28- CD8+ T cell Absolute	GCST90001698	lymphocyte count	IPF
CD45RA+ CD28- CD8+ T cell Absolute	GCST90001698	lymphocyte count	IPF
CD45RA+ CD28- CD8+ T cell Absolute	GCST90001698	lymphocyte count	IPF
CD45RA+ CD28- CD8+ T cell Absolute	GCST90001698	lymphocyte count	IPF
CD45RA+ CD28- CD8+ T cell Absolute	GCST90001698	lymphocyte count	IPF
CD45RA+ CD28- CD8+ T cell Absolute	GCST90001698	lymphocyte count	IPF
CD45RA+ CD28- CD8+ T cell Absolute	GCST90001698	lymphocyte count	IPF
CD45RA+ CD28- CD8+ T cell Absolute	GCST90001698	lymphocyte count	IPF
CD45RA+ CD28- CD8+ T cell Absolute	GCST90001698	lymphocyte count	IPF
CD45RA+ CD28- CD8+ T cell Absolute	GCST90001698	lymphocyte count	IPF
CD45RA+ CD28- CD8+ T cell Absolute	GCST90001698	lymphocyte count	IPF
CD45RA+ CD28- CD8+ T cell Absolute	GCST90001698	lymphocyte count	IPF
CD45RA+ CD28- CD8+ T cell Absolute	GCST90001698	lymphocyte count	IPF
CD45RA+ CD28- CD8+ T cell Absolute	GCST90001698	lymphocyte count	IPF
CD45RA+ CD28- CD8+ T cell Absolute	GCST90001698	lymphocyte count	IPF
CD45RA+ CD28- CD8+ T cell Absolute	GCST90001698	lymphocyte count	IPF
CD45RA+ CD28- CD8+ T cell Absolute	GCST90001698	lymphocyte count	IPF
CD45RA+ CD28- CD8+ T cell Absolute	GCST90001698	lymphocyte count	IPF
CD45RA+ CD28- CD8+ T cell Absolute	GCST90001698	lymphocyte count	IPF
CD45RA+ CD28- CD8+ T cell Absolute	GCST90001698	lymphocyte count	IPF
CD45RA+ CD28- CD8+ T cell Absolute	GCST90001698	lymphocyte count	IPF
CD45RA+ CD28- CD8+ T cell Absolute	GCST90001698	lymphocyte count	IPF
CD45RA+ CD28- CD8+ T cell Absolute	GCST90001698	lymphocyte count	IPF
CD45RA+ CD28- CD8+ T cell Absolute	GCST90001698	lymphocyte count	IPF
CD45RA+ CD28- CD8+ T cell Absolute	GCST90001698	lymphocyte count	IPF
CD45RA+ CD28- CD8+ T cell Absolute	GCST90001698	lymphocyte count	IPF
CD45RA+ CD28- CD8+ T cell Absolute	GCST90001698	lymphocyte count	IPF
CD45RA+ CD28- CD8+ T cell Absolute	GCST90001698	lymphocyte count	IPF
CD45RA+ CD28- CD8+ T cell Absolute	GCST90001698	lymphocyte count	IPF
CD45RA+ CD28- CD8+ T cell Absolute	GCST90001698	lymphocyte count	IPF
CD45RA+ CD28- CD8+ T cell Absolute	GCST90001698	lymphocyte count	IPF
CD45RA+ CD28- CD8+ T cell Absolute	GCST90001698	lymphocyte count	IPF
CD45RA+ CD28- CD8+ T cell Absolute	GCST90001698	lymphocyte count	IPF
CD45RA+ CD28- CD8+ T cell Absolute	GCST90001698	lymphocyte count	IPF
CD45RA+ CD28- CD8+ T cell Absolute	GCST90001698	lymphocyte count	IPF
CD45RA+ CD28- CD8+ T cell Absolute	GCST90001698	lymphocyte count	IPF
CD45RA+ CD28- CD8+ T cell Absolute	GCST90001698	lymphocyte count	IPF
CD45RA+ CD28- CD8+ T cell Absolute	GCST90001698	lymphocyte count	IPF
CD45RA+ CD28- CD8+ T cell Absolute	GCST90001698	lymphocyte count	IPF
CD45RA+ CD28- CD8+ T cell Absolute	GCST90001698	lymphocyte count	IPF
CD45RA+ CD28- CD8+ T cell Absolute	GCST90001698	lymphocyte count	IPF
CD45RA+ CD28- CD8+ T cell Absolute	GCST90001698	lymphocyte count	IPF
CD45RA+ CD28- CD8+ T cell Absolute	GCST90001698	lymphocyte count	IPF
CD45RA+ CD28- CD8+ T cell Absolute	GCST90001698	lymphocyte count	IPF
CD45RA+ CD28- CD8+ T cell Absolute	GCST90001698	lymphocyte count	IPF
CD45RA+ CD28- CD8+ T cell Absolute	GCST90001698	lymphocyte count	IPF
CD45RA+ CD28- CD8+ T cell Absolute	GCST90001698	lymphocyte count	IPF
CD45RA+ CD28- CD8+ T cell Absolute	GCST90001698	lymphocyte count	IPF
CD45RA+ CD28- CD8+ T cell Absolute	GCST90001698	lymphocyte count	IPF
CD45RA+ CD28- CD8+ T cell Absolute	GCST90001698	lymphocyte count	IPF
CD45RA+ CD28- CD8+ T cell Absolute	GCST90001698	lymphocyte count	IPF
CD45RA+ CD28- CD8+ T cell Absolute	GCST90001698	lymphocyte count	IPF

CD45RA+ CD28- CD8+ T cell Absolute	GCST90001698	lymphocyte count	IPF
CD45RA+ CD28- CD8+ T cell Absolute	GCST90001698	lymphocyte count	IPF
CD45RA+ CD28- CD8+ T cell Absolute	GCST90001698	lymphocyte count	IPF
CD45RA+ CD28- CD8+ T cell Absolute	GCST90001698	lymphocyte count	IPF
CD45RA+ CD28- CD8+ T cell Absolute	GCST90001698	lymphocyte count	IPF
CD45RA+ CD28- CD8+ T cell Absolute	GCST90001698	lymphocyte count	IPF
CD45RA+ CD28- CD8+ T cell Absolute	GCST90001698	lymphocyte count	IPF
CD45RA+ CD28- CD8+ T cell Absolute	GCST90001698	lymphocyte count	IPF
CD45RA+ CD28- CD8+ T cell Absolute	GCST90001698	lymphocyte count	IPF
CD45RA+ CD28- CD8+ T cell Absolute	GCST90001698	lymphocyte count	IPF
CD45RA+ CD28- CD8+ T cell Absolute	GCST90001698	lymphocyte count	IPF
CD45RA+ CD28- CD8+ T cell Absolute	GCST90001698	lymphocyte count	IPF
CD45RA+ CD28- CD8+ T cell Absolute	GCST90001698	lymphocyte count	IPF
CD45RA+ CD28- CD8+ T cell Absolute	GCST90001698	lymphocyte count	IPF
CD45RA+ CD28- CD8+ T cell Absolute	GCST90001698	lymphocyte count	IPF
CD45RA+ CD28- CD8+ T cell Absolute	GCST90001698	lymphocyte count	IPF
CD45RA+ CD28- CD8+ T cell Absolute	GCST90001698	lymphocyte count	IPF
CD45RA+ CD28- CD8+ T cell Absolute	GCST90001698	lymphocyte count	IPF
CD45RA+ CD28- CD8+ T cell Absolute	GCST90001698	lymphocyte count	IPF
CD45RA+ CD28- CD8+ T cell Absolute	GCST90001698	lymphocyte count	IPF
CD45RA+ CD28- CD8+ T cell Absolute	GCST90001698	lymphocyte count	IPF
CD45RA+ CD28- CD8+ T cell Absolute	GCST90001698	lymphocyte count	IPF
CD45RA+ CD28- CD8+ T cell Absolute	GCST90001698	lymphocyte count	IPF
CD45RA+ CD28- CD8+ T cell Absolute	GCST90001698	lymphocyte count	IPF
CD45RA+ CD28- CD8+ T cell Absolute	GCST90001698	lymphocyte count	IPF
CD45RA+ CD28- CD8+ T cell Absolute	GCST90001698	lymphocyte count	IPF
CD45RA+ CD28- CD8+ T cell Absolute	GCST90001698	lymphocyte count	IPF
CD45RA+ CD28- CD8+ T cell Absolute	GCST90001698	lymphocyte count	IPF
CD45RA+ CD28- CD8+ T cell Absolute	GCST90001698	lymphocyte count	IPF
CD45RA+ CD28- CD8+ T cell Absolute	GCST90001698	lymphocyte count	IPF
CD45RA+ CD28- CD8+ T cell Absolute	GCST90001698	lymphocyte count	IPF
CD45RA+ CD28- CD8+ T cell Absolute	GCST90001698	lymphocyte count	IPF
CD45RA+ CD28- CD8+ T cell Absolute	GCST90001698	lymphocyte count	IPF
CD45RA+ CD28- CD8+ T cell Absolute	GCST90001698	lymphocyte count	IPF
CD45RA+ CD28- CD8+ T cell Absolute	GCST90001698	lymphocyte count	IPF
CD45RA+ CD28- CD8+ T cell Absolute	GCST90001698	lymphocyte count	IPF
CD45RA+ CD28- CD8+ T cell Absolute	GCST90001698	lymphocyte count	IPF
CD45RA+ CD28- CD8+ T cell Absolute	GCST90001698	lymphocyte count	IPF
CD45RA+ CD28- CD8+ T cell Absolute	GCST90001698	lymphocyte count	IPF
CD45RA+ CD28- CD8+ T cell Absolute	GCST90001698	lymphocyte count	IPF
CD45RA+ CD28- CD8+ T cell Absolute	GCST90001698	lymphocyte count	IPF
CD45RA+ CD28- CD8+ T cell Absolute	GCST90001698	lymphocyte count	IPF
CD45RA+ CD28- CD8+ T cell Absolute	GCST90001698	lymphocyte count	IPF
CD45RA+ CD28- CD8+ T cell Absolute	GCST90001698	lymphocyte count	IPF
CD45RA+ CD28- CD8+ T cell Absolute	GCST90001698	lymphocyte count	IPF
CD45RA+ CD28- CD8+ T cell Absolute	GCST90001698	lymphocyte count	IPF
CD45RA+ CD28- CD8+ T cell Absolute	GCST90001698	lymphocyte count	IPF
CD45RA+ CD28- CD8+ T cell Absolute	GCST90001698	lymphocyte count	IPF
CD45RA+ CD28- CD8+ T cell Absolute	GCST90001698	lymphocyte count	IPF
CD45RA+ CD28- CD8+ T cell Absolute	GCST90001698	lymphocyte count	IPF
CD45RA+ CD28- CD8+ T cell Absolute	GCST90001698	lymphocyte count	IPF
CD45RA+ CD28- CD8+ T cell Absolute	GCST90001698	lymphocyte count	IPF
CD45RA+ CD28- CD8+ T cell Absolute	GCST90001698	lymphocyte count	IPF
CD45RA+ CD28- CD8+ T cell Absolute	GCST90001698	lymphocyte count	IPF
CD45RA+ CD28- CD8+ T cell Absolute	GCST90001698	lymphocyte count	IPF
CD45RA+ CD28- CD8+ T cell Absolute	GCST90001698	lymphocyte count	IPF
CD45RA+ CD28- CD8+ T cell Absolute	GCST90001698	lymphocyte count	IPF
CD45RA+ CD28- CD8+ T cell Absolute	GCST90001698	lymphocyte count	IPF

CD45RA+ CD28- CD8+ T cell Absolute	GCST90001698	lymphocyte count	IPF
CD45RA+ CD28- CD8+ T cell Absolute	GCST90001698	lymphocyte count	IPF
CD45RA+ CD28- CD8+ T cell Absolute	GCST90001698	lymphocyte count	IPF
CD45RA+ CD28- CD8+ T cell Absolute	GCST90001698	lymphocyte count	IPF
CD45RA+ CD28- CD8+ T cell Absolute	GCST90001698	lymphocyte count	IPF
CD45RA+ CD28- CD8+ T cell Absolute	GCST90001698	lymphocyte count	IPF
CD45RA+ CD28- CD8+ T cell Absolute	GCST90001698	lymphocyte count	IPF
CD45RA+ CD28- CD8+ T cell Absolute	GCST90001698	lymphocyte count	IPF
CD45RA+ CD28- CD8+ T cell Absolute	GCST90001698	lymphocyte count	IPF
CD45RA+ CD28- CD8+ T cell Absolute	GCST90001698	lymphocyte count	IPF
CD45RA+ CD28- CD8+ T cell Absolute	GCST90001698	lymphocyte count	IPF
CD45RA+ CD28- CD8+ T cell Absolute	GCST90001698	lymphocyte count	IPF
CD20 on switched memory B cell	GCST90001761	blood protein measurement	IPF
CD20 on switched memory B cell	GCST90001761	blood protein measurement	IPF
CD20 on switched memory B cell	GCST90001761	blood protein measurement	IPF
CD20 on switched memory B cell	GCST90001761	blood protein measurement	IPF
CD20 on switched memory B cell	GCST90001761	blood protein measurement	IPF
CD20 on switched memory B cell	GCST90001761	blood protein measurement	IPF
CD8 on CD8+ T cell	GCST90002058	blood protein measurement	IPF
CD8 on CD8+ T cell	GCST90002058	blood protein measurement	IPF
Natural Killer %lymphocyte	GCST90001647	leukocyte count	IPF
Natural Killer %lymphocyte	GCST90001647	leukocyte count	IPF
Natural Killer %lymphocyte	GCST90001647	leukocyte count	IPF
CD3- lymphocyte %lymphocyte	GCST90001640	leukocyte count	IPF
CD3- lymphocyte %lymphocyte	GCST90001640	leukocyte count	IPF
T cell %lymphocyte	GCST90001604	leukocyte count	IPF
T cell %lymphocyte	GCST90001604	leukocyte count	IPF
CD28+ CD45RA- CD8+ T cell %CD8+ T	GCST90001692	lymphocyte count	IPF
CD28+ CD45RA- CD8+ T cell %CD8+ T	GCST90001692	lymphocyte count	IPF
Naive CD8+ T cell %CD8+ T cell	GCST90001552	lymphocyte count	IPF
CD4 on CD4+ T cell	GCST90002022	blood protein measurement	IPF
CD4 on CD4+ T cell	GCST90002022	blood protein measurement	IPF
CD39+ activated CD4 regulatory T cell A	GCST90001489	lymphocyte count	IPF
CD39+ activated CD4 regulatory T cell A	GCST90001489	lymphocyte count	IPF
CD39+ activated CD4 regulatory T cell A	GCST90001489	lymphocyte count	IPF
CD39+ activated CD4 regulatory T cell A	GCST90001489	lymphocyte count	IPF
CD39+ activated CD4 regulatory T cell A	GCST90001489	lymphocyte count	IPF
CD39+ activated CD4 regulatory T cell %	GCST90001491	lymphocyte count	IPF
CD39+ activated CD4 regulatory T cell %	GCST90001491	lymphocyte count	IPF
CD39+ activated CD4 regulatory T cell %	GCST90001491	lymphocyte count	IPF
CD39+ activated CD4 regulatory T cell %	GCST90001491	lymphocyte count	IPF
CD39+ activated CD4 regulatory T cell %	GCST90001491	lymphocyte count	IPF
CD39+ activated CD4 regulatory T cell %	GCST90001491	lymphocyte count	IPF
CD39+ CD4+ T cell Absolute Count	GCST90001660	lymphocyte count	IPF
CD39+ CD4+ T cell Absolute Count	GCST90001660	lymphocyte count	IPF
CD39+ CD4+ T cell Absolute Count	GCST90001660	lymphocyte count	IPF
CD39+ CD4+ T cell Absolute Count	GCST90001660	lymphocyte count	IPF
CD39+ CD4+ T cell Absolute Count	GCST90001660	lymphocyte count	IPF
CD39+ CD4+ T cell Absolute Count	GCST90001660	lymphocyte count	IPF
CD39+ secreting CD4 regulatory T cell %	GCST90001497	lymphocyte count	IPF
CD39+ secreting CD4 regulatory T cell %	GCST90001497	lymphocyte count	IPF
CD39+ secreting CD4 regulatory T cell %	GCST90001497	lymphocyte count	IPF
CD39+ secreting CD4 regulatory T cell %	GCST90001497	lymphocyte count	IPF
CD39+ secreting CD4 regulatory T cell %	GCST90001497	lymphocyte count	IPF
CD39+ secreting CD4 regulatory T cell %	GCST90001497	lymphocyte count	IPF
CD4 on CD39+ CD4+ T cell	GCST90002061	blood protein measurement	IPF
CD4 on CD39+ CD4+ T cell	GCST90002061	blood protein measurement	IPF
CD4 on CD39+ CD4+ T cell	GCST90002061	blood protein measurement	IPF

CD4 on CD39+ CD4+ T cell	GCST90002061	blood protein measurement	IPF
CD4 on CD39+ CD4+ T cell	GCST90002061	blood protein measurement	IPF
CD4 on CD39+ CD4+ T cell	GCST90002061	blood protein measurement	IPF
BAFF-R on IgD+ CD38- naive B cell	GCST90001706	blood protein measurement	IPF
BAFF-R on IgD+ CD38- naive B cell	GCST90001706	blood protein measurement	IPF
BAFF-R on IgD+ CD38- naive B cell	GCST90001706	blood protein measurement	IPF
BAFF-R on IgD+ CD38- naive B cell	GCST90001706	blood protein measurement	IPF
BAFF-R on IgD+ CD38- naive B cell	GCST90001706	blood protein measurement	IPF
BAFF-R on IgD+ CD38- naive B cell	GCST90001706	blood protein measurement	IPF
BAFF-R on IgD+ CD38- naive B cell	GCST90001706	blood protein measurement	IPF
BAFF-R on IgD+ B cell	GCST90001719	blood protein measurement	IPF
BAFF-R on IgD+ B cell	GCST90001719	blood protein measurement	IPF
BAFF-R on IgD+ B cell	GCST90001719	blood protein measurement	IPF
BAFF-R on IgD+ B cell	GCST90001719	blood protein measurement	IPF
BAFF-R on IgD+ B cell	GCST90001719	blood protein measurement	IPF
BAFF-R on IgD+ B cell	GCST90001719	blood protein measurement	IPF
BAFF-R on IgD+ B cell	GCST90001719	blood protein measurement	IPF
BAFF-R on IgD+ B cell	GCST90001719	blood protein measurement	IPF
BAFF-R on B cell	GCST90001829	blood protein measurement	IPF
BAFF-R on B cell	GCST90001829	blood protein measurement	IPF
BAFF-R on B cell	GCST90001829	blood protein measurement	IPF
BAFF-R on B cell	GCST90001829	blood protein measurement	IPF
BAFF-R on B cell	GCST90001829	blood protein measurement	IPF
BAFF-R on B cell	GCST90001829	blood protein measurement	IPF
BAFF-R on B cell	GCST90001829	blood protein measurement	IPF
BAFF-R on B cell	GCST90001829	blood protein measurement	IPF
BAFF-R on B cell	GCST90001829	blood protein measurement	IPF
BAFF-R on IgD+ CD38+ B cell	GCST90001708	blood protein measurement	IPF
BAFF-R on IgD+ CD38+ B cell	GCST90001708	blood protein measurement	IPF
BAFF-R on IgD+ CD38+ B cell	GCST90001708	blood protein measurement	IPF
BAFF-R on IgD+ CD38+ B cell	GCST90001708	blood protein measurement	IPF
BAFF-R on IgD+ CD38+ B cell	GCST90001708	blood protein measurement	IPF
BAFF-R on IgD+ CD38+ B cell	GCST90001708	blood protein measurement	IPF
BAFF-R on IgD+ CD38+ B cell	GCST90001708	blood protein measurement	IPF
BAFF-R on IgD+ CD38+ B cell	GCST90001708	blood protein measurement	IPF
BAFF-R on IgD+ CD38+ B cell	GCST90001708	blood protein measurement	IPF
BAFF-R on transitional B cell	GCST90001720	blood protein measurement	IPF
BAFF-R on transitional B cell	GCST90001720	blood protein measurement	IPF
BAFF-R on transitional B cell	GCST90001720	blood protein measurement	IPF
BAFF-R on transitional B cell	GCST90001720	blood protein measurement	IPF
BAFF-R on transitional B cell	GCST90001720	blood protein measurement	IPF
BAFF-R on transitional B cell	GCST90001720	blood protein measurement	IPF
BAFF-R on transitional B cell	GCST90001720	blood protein measurement	IPF
BAFF-R on transitional B cell	GCST90001720	blood protein measurement	IPF
BAFF-R on IgD+ CD38dim B cell	GCST90001709	blood protein measurement	IPF
BAFF-R on IgD+ CD38dim B cell	GCST90001709	blood protein measurement	IPF
BAFF-R on IgD+ CD38dim B cell	GCST90001709	blood protein measurement	IPF
BAFF-R on IgD+ CD38dim B cell	GCST90001709	blood protein measurement	IPF
BAFF-R on IgD+ CD38dim B cell	GCST90001709	blood protein measurement	IPF
BAFF-R on IgD+ CD38dim B cell	GCST90001709	blood protein measurement	IPF
BAFF-R on IgD+ CD38dim B cell	GCST90001709	blood protein measurement	IPF
BAFF-R on IgD+ CD38dim B cell	GCST90001709	blood protein measurement	IPF
BAFF-R on IgD+ CD24- B cell	GCST90001704	blood protein measurement	IPF
BAFF-R on IgD+ CD24- B cell	GCST90001704	blood protein measurement	IPF
BAFF-R on IgD+ CD24- B cell	GCST90001704	blood protein measurement	IPF
BAFF-R on IgD+ CD24- B cell	GCST90001704	blood protein measurement	IPF
BAFF-R on IgD+ CD24- B cell	GCST90001704	blood protein measurement	IPF
BAFF-R on IgD+ CD24- B cell	GCST90001704	blood protein measurement	IPF

BAFF-R on IgD+ CD24- B cell	GCST90001704	blood protein measurement	IPF
BAFF-R on IgD+ CD24- B cell	GCST90001704	blood protein measurement	IPF
BAFF-R on IgD+ CD24- B cell	GCST90001704	blood protein measurement	IPF
BAFF-R on naive-mature B cell	GCST90001716	blood protein measurement	IPF
BAFF-R on naive-mature B cell	GCST90001716	blood protein measurement	IPF
BAFF-R on naive-mature B cell	GCST90001716	blood protein measurement	IPF
BAFF-R on naive-mature B cell	GCST90001716	blood protein measurement	IPF
BAFF-R on naive-mature B cell	GCST90001716	blood protein measurement	IPF
BAFF-R on naive-mature B cell	GCST90001716	blood protein measurement	IPF
BAFF-R on naive-mature B cell	GCST90001716	blood protein measurement	IPF
BAFF-R on naive-mature B cell	GCST90001716	blood protein measurement	IPF
BAFF-R on IgD+ CD38- B cell	GCST90001705	blood protein measurement	IPF
BAFF-R on IgD+ CD38- B cell	GCST90001705	blood protein measurement	IPF
BAFF-R on IgD+ CD38- B cell	GCST90001705	blood protein measurement	IPF
BAFF-R on IgD+ CD38- B cell	GCST90001705	blood protein measurement	IPF
BAFF-R on IgD+ CD38- B cell	GCST90001705	blood protein measurement	IPF
BAFF-R on IgD+ CD38- B cell	GCST90001705	blood protein measurement	IPF
BAFF-R on IgD+ CD38- B cell	GCST90001705	blood protein measurement	IPF
HLA DR+ T cell%T cell	GCST90001619	leukocyte count	IPF
HLA DR+ T cell%T cell	GCST90001619	leukocyte count	IPF
HLA DR+ T cell%T cell	GCST90001619	leukocyte count	IPF
CD39+ secreting CD4 regulatory T cell A	GCST90001495	lymphocyte count	IPF
CD39+ secreting CD4 regulatory T cell A	GCST90001495	lymphocyte count	IPF
CD39+ secreting CD4 regulatory T cell A	GCST90001495	lymphocyte count	IPF
CD39+ secreting CD4 regulatory T cell A	GCST90001495	lymphocyte count	IPF
CD39+ secreting CD4 regulatory T cell A	GCST90001495	lymphocyte count	IPF
CD39+ secreting CD4 regulatory T cell A	GCST90001495	lymphocyte count	IPF
CD39+ secreting CD4 regulatory T cell A	GCST90001495	lymphocyte count	IPF
CD39+ secreting CD4 regulatory T cell %	GCST90001496	lymphocyte count	IPF
CD39+ secreting CD4 regulatory T cell %	GCST90001496	lymphocyte count	IPF
CD39+ secreting CD4 regulatory T cell %	GCST90001496	lymphocyte count	IPF
CD39+ secreting CD4 regulatory T cell %	GCST90001496	lymphocyte count	IPF
CD39+ secreting CD4 regulatory T cell %	GCST90001496	lymphocyte count	IPF
CD39+ secreting CD4 regulatory T cell %	GCST90001496	lymphocyte count	IPF
CD39+ activated CD4 regulatory T cell %	GCST90001490	lymphocyte count	IPF
CD39+ activated CD4 regulatory T cell %	GCST90001490	lymphocyte count	IPF
CD39+ activated CD4 regulatory T cell %	GCST90001490	lymphocyte count	IPF
CD39+ activated CD4 regulatory T cell %	GCST90001490	lymphocyte count	IPF
CD39+ activated CD4 regulatory T cell %	GCST90001490	lymphocyte count	IPF
CD39+ activated CD4 regulatory T cell %	GCST90001490	lymphocyte count	IPF
CD39+ activated CD4 regulatory T cell %	GCST90001490	lymphocyte count	IPF
CD39+ activated CD4 regulatory T cell %	GCST90001490	lymphocyte count	IPF
CD39+ CD4+ T cell %T cell	GCST90001658	lymphocyte count	IPF
CD39+ CD4+ T cell %T cell	GCST90001658	lymphocyte count	IPF
CD39+ CD4+ T cell %T cell	GCST90001658	lymphocyte count	IPF
CD39+ CD4+ T cell %T cell	GCST90001658	lymphocyte count	IPF
CD39+ CD4+ T cell %T cell	GCST90001658	lymphocyte count	IPF
CD39+ CD4+ T cell %T cell	GCST90001658	lymphocyte count	IPF
CD4 on CD39+ secreting CD4 regulatory	GCST90002069	blood protein measurement	IPF
CD4 on CD39+ secreting CD4 regulatory	GCST90002069	blood protein measurement	IPF
CD3 on CD39+ activated CD4 regulatory	GCST90001854	blood protein measurement	IPF
CD3 on CD39+ activated CD4 regulatory	GCST90001854	blood protein measurement	IPF
CD3 on CD39+ activated CD4 regulatory	GCST90001854	blood protein measurement	IPF
CD39 on CD39+ secreting CD4 regulator	GCST90002031	blood protein measurement	IPF
CD39 on CD39+ secreting CD4 regulator	GCST90002031	blood protein measurement	IPF
CD39 on CD39+ secreting CD4 regulator	GCST90002031	blood protein measurement	IPF
CD39 on CD39+ secreting CD4 regulator	GCST90002031	blood protein measurement	IPF
CD39 on CD39+ secreting CD4 regulator	GCST90002031	blood protein measurement	IPF

CD28 on CD39+ CD4+ T cell	GCST90001892	blood protein measurement	IPF
CD28 on CD39+ CD4+ T cell	GCST90001892	blood protein measurement	IPF
CD28 on CD39+ CD4+ T cell	GCST90001892	blood protein measurement	IPF
CD28 on CD39+ CD4+ T cell	GCST90001892	blood protein measurement	IPF
CD28 on CD39+ CD4+ T cell	GCST90001892	blood protein measurement	IPF
CD39 on CD39+ CD4+ T cell	GCST90002032	blood protein measurement	IPF
CD39 on CD39+ CD4+ T cell	GCST90002032	blood protein measurement	IPF
CD39 on CD39+ CD4+ T cell	GCST90002032	blood protein measurement	IPF
CD39 on CD39+ CD4+ T cell	GCST90002032	blood protein measurement	IPF
CD39 on CD39+ CD8+ T cell	GCST90002029	blood protein measurement	IPF
CD39 on CD39+ CD8+ T cell	GCST90002029	blood protein measurement	IPF
CD39 on CD39+ activated CD4 regulator	GCST90002030	blood protein measurement	IPF
CD39 on CD39+ activated CD4 regulator	GCST90002030	blood protein measurement	IPF
CD39 on CD39+ activated CD4 regulator	GCST90002030	blood protein measurement	IPF
CD39 on CD39+ activated CD4 regulator	GCST90002030	blood protein measurement	IPF
CD39 on CD39+ activated CD4 regulator	GCST90002030	blood protein measurement	IPF
CD39 on CD39+ activated CD4 regulator	GCST90002030	blood protein measurement	IPF
CD39+ CD4+ T cell %CD4+ T cell	GCST90001659	lymphocyte count	IPF
CD39+ CD4+ T cell %CD4+ T cell	GCST90001659	lymphocyte count	IPF
CD39+ CD4+ T cell %CD4+ T cell	GCST90001659	lymphocyte count	IPF
CD39+ CD4+ T cell %CD4+ T cell	GCST90001659	lymphocyte count	IPF
CD39+ CD4+ T cell %CD4+ T cell	GCST90001659	lymphocyte count	IPF
CD40 on monocytes	GCST90001985	blood protein measurement	IPF
CD40 on monocytes	GCST90001985	blood protein measurement	IPF
CD40 on monocytes	GCST90001985	blood protein measurement	IPF
CD40 on monocytes	GCST90001985	blood protein measurement	IPF
CD25 on IgD+ CD38- unswitched memor	GCST90001782	blood protein measurement	IPF
CD25++ CD45RA+ CD4 not regulatory '	GCST90001509	lymphocyte count	IPF
CD25++ CD45RA+ CD4 not regulatory '	GCST90001509	lymphocyte count	IPF
CD25++ CD45RA+ CD4 not regulatory '	GCST90001509	lymphocyte count	IPF
CD25++ CD45RA+ CD4 not regulatory '	GCST90001509	lymphocyte count	IPF
CD25 on CD45RA+ CD4 not regulatory '	GCST90001934	blood protein measurement	IPF
CD25++ CD45RA+ CD4 not regulatory '	GCST90001508	lymphocyte count	IPF
CD25++ CD45RA+ CD4 not regulatory '	GCST90001508	lymphocyte count	IPF
CD25++ CD45RA+ CD4 not regulatory '	GCST90001508	lymphocyte count	IPF
CD25++ CD45RA+ CD4 not regulatory '	GCST90001508	lymphocyte count	IPF
CD45 on CD33- HLA DR-	GCST90002045	blood protein measurement	IPF
CD45 on CD33- HLA DR-	GCST90002045	blood protein measurement	IPF
CD16-CD56 on Natural Killer	GCST90001884	blood protein measurement	IPF
CD16-CD56 on Natural Killer	GCST90001884	blood protein measurement	IPF
CD16-CD56 on Natural Killer	GCST90001884	blood protein measurement	IPF
CD16-CD56 on Natural Killer	GCST90001884	blood protein measurement	IPF
CD16-CD56 on Natural Killer	GCST90001884	blood protein measurement	IPF
CD16-CD56 on Natural Killer	GCST90001884	blood protein measurement	IPF
CD16-CD56 on Natural Killer	GCST90001884	blood protein measurement	IPF
CD4+CD8+ T cell Absolute Count	GCST90001594	leukocyte count	IPF
CD4+CD8+ T cell Absolute Count	GCST90001594	leukocyte count	IPF
CD27 on IgD- CD38- B cell	GCST90001802	blood protein measurement	IPF
CD27 on IgD- CD38- B cell	GCST90001802	blood protein measurement	IPF
CD27 on IgD- CD38- B cell	GCST90001802	blood protein measurement	IPF
CD27 on IgD- CD38- B cell	GCST90001802	blood protein measurement	IPF
CD27 on IgD- CD38- B cell	GCST90001802	blood protein measurement	IPF
CD27 on IgD- CD38- B cell	GCST90001802	blood protein measurement	IPF
CD27 on IgD- CD38- B cell	GCST90001802	blood protein measurement	IPF
CD64 on CD14+ CD16+ monocyte	GCST90002011	blood protein measurement	IPF
CD64 on CD14+ CD16+ monocyte	GCST90002011	blood protein measurement	IPF
CD64 on CD14+ CD16+ monocyte	GCST90002011	blood protein measurement	IPF
HLA DR on HLA DR+ CD4+ T cell	GCST90002114	blood protein measurement	IPF

HLA DR on HLA DR+ T cell	GCST90002113	blood protein measurement	IPF
HLA DR on CD33dim HLA DR+ CD11b	GCST90002110	blood protein measurement	IPF
HLA DR on CD33dim HLA DR+ CD11b	GCST90002110	blood protein measurement	IPF
HLA DR on CD33dim HLA DR+ CD11b	GCST90002111	blood protein measurement	IPF
HLA DR on CD33dim HLA DR+ CD11b	GCST90002111	blood protein measurement	IPF
HLA DR on CD33dim HLA DR+ CD11b	GCST90002111	blood protein measurement	IPF
CD8+ and CD8dim T cell %leukocyte	GCST90001614	leukocyte count	IPF
CD8+ and CD8dim T cell %leukocyte	GCST90001614	leukocyte count	IPF
CD27 on CD24+ CD27+ B cell	GCST90001798	blood protein measurement	IPF
CD27 on CD24+ CD27+ B cell	GCST90001798	blood protein measurement	IPF
CD27 on CD24+ CD27+ B cell	GCST90001798	blood protein measurement	IPF
CD27 on CD24+ CD27+ B cell	GCST90001798	blood protein measurement	IPF
CD27 on CD24+ CD27+ B cell	GCST90001798	blood protein measurement	IPF
CD27 on CD24+ CD27+ B cell	GCST90001798	blood protein measurement	IPF
CD27 on CD24+ CD27+ B cell	GCST90001798	blood protein measurement	IPF
CD27 on CD24+ CD27+ B cell	GCST90001798	blood protein measurement	IPF
CD27 on CD24+ CD27+ B cell	GCST90001798	blood protein measurement	IPF
CD27 on CD24+ CD27+ B cell	GCST90001798	blood protein measurement	IPF
CD27 on memory B cell	GCST90001805	blood protein measurement	IPF
CD27 on memory B cell	GCST90001805	blood protein measurement	IPF
CD27 on memory B cell	GCST90001805	blood protein measurement	IPF
CD27 on memory B cell	GCST90001805	blood protein measurement	IPF
CD27 on memory B cell	GCST90001805	blood protein measurement	IPF
CD27 on memory B cell	GCST90001805	blood protein measurement	IPF
CD27 on memory B cell	GCST90001805	blood protein measurement	IPF
CD27 on memory B cell	GCST90001805	blood protein measurement	IPF
CD27 on switched memory B cell	GCST90001808	blood protein measurement	IPF
CD27 on switched memory B cell	GCST90001808	blood protein measurement	IPF
CD27 on switched memory B cell	GCST90001808	blood protein measurement	IPF
CD27 on switched memory B cell	GCST90001808	blood protein measurement	IPF
CD27 on switched memory B cell	GCST90001808	blood protein measurement	IPF
CD27 on switched memory B cell	GCST90001808	blood protein measurement	IPF
CD27 on switched memory B cell	GCST90001808	blood protein measurement	IPF
CD27 on switched memory B cell	GCST90001808	blood protein measurement	IPF
CD27 on switched memory B cell	GCST90001808	blood protein measurement	IPF
CD27 on unswitched memory B cell	GCST90001806	blood protein measurement	IPF
CD27 on unswitched memory B cell	GCST90001806	blood protein measurement	IPF
CD27 on unswitched memory B cell	GCST90001806	blood protein measurement	IPF
CD27 on unswitched memory B cell	GCST90001806	blood protein measurement	IPF
CD27 on unswitched memory B cell	GCST90001806	blood protein measurement	IPF
CD27 on unswitched memory B cell	GCST90001806	blood protein measurement	IPF
CD27 on unswitched memory B cell	GCST90001806	blood protein measurement	IPF
CD27 on unswitched memory B cell	GCST90001806	blood protein measurement	IPF
CD27 on unswitched memory B cell	GCST90001806	blood protein measurement	IPF
CD27 on IgD- CD38dim B cell	GCST90001804	blood protein measurement	IPF
CD27 on IgD- CD38dim B cell	GCST90001804	blood protein measurement	IPF
CD27 on IgD- CD38dim B cell	GCST90001804	blood protein measurement	IPF
CD27 on IgD- CD38dim B cell	GCST90001804	blood protein measurement	IPF
CD27 on IgD- CD38dim B cell	GCST90001804	blood protein measurement	IPF
CD27 on IgD- CD38dim B cell	GCST90001804	blood protein measurement	IPF
CD27 on IgD- CD38dim B cell	GCST90001804	blood protein measurement	IPF
CD27 on IgD- CD38dim B cell	GCST90001804	blood protein measurement	IPF
CD33+ HLA DR+ CD14dim %CD33+ H	GCST90001521	myeloid white cell count	IPF
CD45RA on resting CD4 regulatory T cel	GCST90002102	blood protein measurement	IPF
CD45RA on resting CD4 regulatory T cel	GCST90002102	blood protein measurement	IPF
CD45RA on resting CD4 regulatory T cel	GCST90002102	blood protein measurement	IPF
CD45RA on resting CD4 regulatory T cel	GCST90002102	blood protein measurement	IPF
CD45RA- CD4+ T cell %CD4+ T cell	GCST90001535	lymphocyte count	IPF

CD45RA- CD4+ T cell %CD4+ T cell	GCST90001535	lymphocyte count	IPF
CD45RA- CD4+ T cell %CD4+ T cell	GCST90001535	lymphocyte count	IPF
CD25++ CD45RA+ CD4 not regulatory T	GCST90001507	lymphocyte count	IPF
CD25++ CD45RA+ CD4 not regulatory T	GCST90001507	lymphocyte count	IPF
CD25++ CD45RA+ CD4 not regulatory T	GCST90001507	lymphocyte count	IPF
CD8 on CD39+ CD8+ T cell	GCST90002121	blood protein measurement	IPF
CD8 on CD39+ CD8+ T cell	GCST90002121	blood protein measurement	IPF
CD8 on CD39+ CD8+ T cell	GCST90002121	blood protein measurement	IPF
CD8 on CD39+ CD8+ T cell	GCST90002121	blood protein measurement	IPF
SSC-A on monocyte	GCST90002073	blood protein measurement	IPF
SSC-A on monocyte	GCST90002073	blood protein measurement	IPF
SSC-A on monocyte	GCST90002073	blood protein measurement	IPF
SSC-A on monocyte	GCST90002073	blood protein measurement	IPF
SSC-A on monocyte	GCST90002073	blood protein measurement	IPF
SSC-A on monocyte	GCST90002073	blood protein measurement	IPF
CD45RA- CD28- CD8+ T cell %T cell	GCST90001697	lymphocyte count	IPF
CD45RA- CD28- CD8+ T cell %T cell	GCST90001697	lymphocyte count	IPF
CD45RA- CD28- CD8+ T cell %T cell	GCST90001697	lymphocyte count	IPF
CD45RA- CD28- CD8+ T cell %T cell	GCST90001697	lymphocyte count	IPF
CD45RA- CD28- CD8+ T cell %T cell	GCST90001697	lymphocyte count	IPF
CD45RA- CD28- CD8+ T cell %T cell	GCST90001697	lymphocyte count	IPF
CD45RA- CD28- CD8+ T cell %T cell	GCST90001697	lymphocyte count	IPF
CD45RA- CD28- CD8+ T cell %T cell	GCST90001697	lymphocyte count	IPF
CD45RA- CD28- CD8+ T cell %T cell	GCST90001697	lymphocyte count	IPF
CD45RA- CD28- CD8+ T cell %T cell	GCST90001697	lymphocyte count	IPF
CD45RA- CD28- CD8+ T cell %T cell	GCST90001697	lymphocyte count	IPF
CD45RA- CD28- CD8+ T cell %T cell	GCST90001697	lymphocyte count	IPF
CD45RA- CD28- CD8+ T cell %T cell	GCST90001697	lymphocyte count	IPF
CD45RA- CD28- CD8+ T cell %T cell	GCST90001697	lymphocyte count	IPF
CD45RA- CD28- CD8+ T cell %T cell	GCST90001697	lymphocyte count	IPF
CD45RA- CD28- CD8+ T cell %T cell	GCST90001697	lymphocyte count	IPF
CD45RA- CD28- CD8+ T cell %T cell	GCST90001697	lymphocyte count	IPF
CD45RA- CD28- CD8+ T cell %T cell	GCST90001697	lymphocyte count	IPF
CD45RA- CD28- CD8+ T cell %T cell	GCST90001697	lymphocyte count	IPF
CD45RA- CD28- CD8+ T cell %T cell	GCST90001697	lymphocyte count	IPF
CD45RA- CD28- CD8+ T cell %T cell	GCST90001697	lymphocyte count	IPF
CD45RA- CD28- CD8+ T cell %T cell	GCST90001697	lymphocyte count	IPF
CD45RA- CD28- CD8+ T cell %T cell	GCST90001697	lymphocyte count	IPF
CD45RA- CD28- CD8+ T cell Absolute (	GCST90001695	lymphocyte count	IPF
CD45RA- CD28- CD8+ T cell Absolute (	GCST90001695	lymphocyte count	IPF
CD45RA- CD28- CD8+ T cell Absolute (	GCST90001695	lymphocyte count	IPF
CD45RA- CD28- CD8+ T cell Absolute (	GCST90001695	lymphocyte count	IPF
CD45RA- CD28- CD8+ T cell Absolute (	GCST90001695	lymphocyte count	IPF
CD45RA- CD28- CD8+ T cell Absolute (	GCST90001695	lymphocyte count	IPF
CD45RA- CD28- CD8+ T cell Absolute (	GCST90001695	lymphocyte count	IPF
CD45RA- CD28- CD8+ T cell Absolute (	GCST90001695	lymphocyte count	IPF
CD45RA- CD28- CD8+ T cell Absolute (	GCST90001695	lymphocyte count	IPF
CD45RA- CD28- CD8+ T cell Absolute (	GCST90001695	lymphocyte count	IPF
CD45RA- CD28- CD8+ T cell Absolute (	GCST90001695	lymphocyte count	IPF
CD45RA- CD28- CD8+ T cell Absolute (	GCST90001695	lymphocyte count	IPF
CD45RA- CD28- CD8+ T cell Absolute (	GCST90001695	lymphocyte count	IPF
CD45RA- CD28- CD8+ T cell Absolute (	GCST90001695	lymphocyte count	IPF
CD45RA- CD28- CD8+ T cell Absolute (	GCST90001695	lymphocyte count	IPF
CD45RA- CD28- CD8+ T cell Absolute (	GCST90001695	lymphocyte count	IPF
CD45RA- CD28- CD8+ T cell Absolute (	GCST90001695	lymphocyte count	IPF
CD45RA- CD28- CD8+ T cell Absolute (	GCST90001695	lymphocyte count	IPF
CD45RA- CD28- CD8+ T cell Absolute (	GCST90001695	lymphocyte count	IPF
CD45RA- CD28- CD8+ T cell Absolute (	GCST90001695	lymphocyte count	IPF

CD45RA- CD28- CD8+ T cell Absolute (	GCST90001695	lymphocyte count	IPF
CD45RA- CD28- CD8+ T cell Absolute (	GCST90001695	lymphocyte count	IPF
CD45RA- CD28- CD8+ T cell Absolute (	GCST90001695	lymphocyte count	IPF
CD45RA- CD28- CD8+ T cell Absolute (	GCST90001695	lymphocyte count	IPF
CD45RA- CD28- CD8+ T cell Absolute (	GCST90001695	lymphocyte count	IPF
CD45RA- CD28- CD8+ T cell Absolute (	GCST90001695	lymphocyte count	IPF
CD45RA- CD28- CD8+ T cell Absolute (	GCST90001695	lymphocyte count	IPF
CD45RA- CD28- CD8+ T cell Absolute (	GCST90001695	lymphocyte count	IPF
CD45RA- CD28- CD8+ T cell Absolute (	GCST90001695	lymphocyte count	IPF
CD45RA- CD28- CD8+ T cell Absolute (	GCST90001695	lymphocyte count	IPF
CD45RA- CD28- CD8+ T cell Absolute (	GCST90001695	lymphocyte count	IPF
CD45RA- CD28- CD8+ T cell Absolute (	GCST90001695	lymphocyte count	IPF
CD45RA- CD28- CD8+ T cell Absolute (	GCST90001695	lymphocyte count	IPF
CD45RA- CD28- CD8+ T cell Absolute (	GCST90001695	lymphocyte count	IPF
CD45RA- CD28- CD8+ T cell Absolute (	GCST90001695	lymphocyte count	IPF
CD45RA- CD28- CD8+ T cell Absolute (	GCST90001695	lymphocyte count	IPF
CD45RA- CD28- CD8+ T cell Absolute (	GCST90001695	lymphocyte count	IPF
CD45RA- CD28- CD8+ T cell Absolute (	GCST90001695	lymphocyte count	IPF
CD45RA- CD28- CD8+ T cell Absolute (	GCST90001695	lymphocyte count	IPF
CD45RA- CD28- CD8+ T cell Absolute (	GCST90001695	lymphocyte count	IPF
CD45RA- CD28- CD8+ T cell Absolute (	GCST90001695	lymphocyte count	IPF
CD45RA- CD28- CD8+ T cell Absolute (	GCST90001695	lymphocyte count	IPF
CD45RA- CD28- CD8+ T cell Absolute (	GCST90001695	lymphocyte count	IPF
CD45RA- CD28- CD8+ T cell Absolute (	GCST90001695	lymphocyte count	IPF
CD45RA- CD28- CD8+ T cell Absolute (	GCST90001695	lymphocyte count	IPF
CD45RA- CD28- CD8+ T cell Absolute (	GCST90001695	lymphocyte count	IPF
CD45RA- CD28- CD8+ T cell Absolute (	GCST90001695	lymphocyte count	IPF
CD45RA- CD28- CD8+ T cell Absolute (	GCST90001695	lymphocyte count	IPF
CD45RA- CD28- CD8+ T cell Absolute (	GCST90001695	lymphocyte count	IPF
CD45RA- CD28- CD8+ T cell Absolute (	GCST90001695	lymphocyte count	IPF
CD45RA- CD28- CD8+ T cell Absolute (	GCST90001695	lymphocyte count	IPF
CD45RA- CD28- CD8+ T cell Absolute (	GCST90001695	lymphocyte count	IPF
CD45RA- CD28- CD8+ T cell Absolute (	GCST90001695	lymphocyte count	IPF
CD45RA- CD28- CD8+ T cell Absolute (	GCST90001695	lymphocyte count	IPF
CD45RA- CD28- CD8+ T cell Absolute (	GCST90001695	lymphocyte count	IPF
CD45RA- CD28- CD8+ T cell Absolute (	GCST90001695	lymphocyte count	IPF
CD45RA- CD28- CD8+ T cell Absolute (	GCST90001695	lymphocyte count	IPF
CD45RA- CD28- CD8+ T cell Absolute (	GCST90001695	lymphocyte count	IPF
CD45RA- CD28- CD8+ T cell Absolute (	GCST90001695	lymphocyte count	IPF
CD45RA- CD28- CD8+ T cell Absolute (	GCST90001695	lymphocyte count	IPF
CD45RA- CD28- CD8+ T cell Absolute (	GCST90001695	lymphocyte count	IPF
CD45RA- CD28- CD8+ T cell Absolute (	GCST90001695	lymphocyte count	IPF
CD45RA- CD28- CD8+ T cell Absolute (	GCST90001695	lymphocyte count	IPF
CD45RA- CD28- CD8+ T cell Absolute (	GCST90001695	lymphocyte count	IPF
CD45RA- CD28- CD8+ T cell Absolute (	GCST90001695	lymphocyte count	IPF
CD45RA- CD28- CD8+ T cell Absolute (	GCST90001695	lymphocyte count	IPF
CD45RA- CD28- CD8+ T cell Absolute (	GCST90001695	lymphocyte count	IPF
CD45RA- CD28- CD8+ T cell Absolute (	GCST90001695	lymphocyte count	IPF
CD45RA- CD28- CD8+ T cell Absolute (	GCST90001695	lymphocyte count	IPF
CD45RA- CD28- CD8+ T cell Absolute (	GCST90001695	lymphocyte count	IPF
CD45RA- CD28- CD8+ T cell Absolute (	GCST90001695	lymphocyte count	IPF
CD45RA- CD28- CD8+ T cell Absolute (	GCST90001695	lymphocyte count	IPF
CD45RA- CD28- CD8+ T cell Absolute (	GCST90001695	lymphocyte count	IPF
CD45RA- CD28- CD8+ T cell Absolute (	GCST90001695	lymphocyte count	IPF
CD45RA- CD28- CD8+ T cell Absolute (	GCST90001695	lymphocyte count	IPF
CD45RA- CD28- CD8+ T cell Absolute (	GCST90001695	lymphocyte count	IPF
CD45RA- CD28- CD8+ T cell Absolute (	GCST90001695	lymphocyte count	IPF
CD45RA- CD28- CD8+ T cell Absolute (	GCST90001695	lymphocyte count	IPF

CD45RA- CD28- CD8+ T cell Absolute (	GCST90001695	lymphocyte count	IPF
CD45RA- CD28- CD8+ T cell Absolute (	GCST90001695	lymphocyte count	IPF
CD45RA- CD28- CD8+ T cell Absolute (	GCST90001695	lymphocyte count	IPF
CD40 on CD14- CD16+ monocyte	GCST90001989	blood protein measurement	IPF
CD40 on CD14- CD16+ monocyte	GCST90001989	blood protein measurement	IPF
CD40 on CD14- CD16+ monocyte	GCST90001989	blood protein measurement	IPF
CD40 on CD14- CD16+ monocyte	GCST90001989	blood protein measurement	IPF
CD40 on CD14- CD16+ monocyte	GCST90001989	blood protein measurement	IPF
CD40 on CD14- CD16+ monocyte	GCST90001989	blood protein measurement	IPF
CD40 on CD14- CD16+ monocyte	GCST90001989	blood protein measurement	IPF
CD40 on CD14- CD16+ monocyte	GCST90001989	blood protein measurement	IPF
CD4 on naive CD4+ T cell	GCST90002024	blood protein measurement	IPF
CD4 on naive CD4+ T cell	GCST90002024	blood protein measurement	IPF
CD4 on naive CD4+ T cell	GCST90002024	blood protein measurement	IPF
CD4 on naive CD4+ T cell	GCST90002024	blood protein measurement	IPF
CD33 on basophil	GCST90001954	blood protein measurement	IPF
CD33 on basophil	GCST90001954	blood protein measurement	IPF
CD33 on basophil	GCST90001954	blood protein measurement	IPF
CD33 on basophil	GCST90001954	blood protein measurement	IPF
CD33 on basophil	GCST90001954	blood protein measurement	IPF
CD33 on basophil	GCST90001954	blood protein measurement	IPF
CD33 on Immature Myeloid-Derived Sup	GCST90001955	blood protein measurement	IPF
CD33 on Immature Myeloid-Derived Sup	GCST90001955	blood protein measurement	IPF
CD33 on Immature Myeloid-Derived Sup	GCST90001955	blood protein measurement	IPF
CD33 on Immature Myeloid-Derived Sup	GCST90001955	blood protein measurement	IPF
CD33 on Immature Myeloid-Derived Sup	GCST90001955	blood protein measurement	IPF
CD45 on Immature Myeloid-Derived Sup	GCST90002052	blood protein measurement	IPF
CD45 on Immature Myeloid-Derived Sup	GCST90002052	blood protein measurement	IPF
CD3 on naive CD8+ T cell	GCST90001838	blood protein measurement	IPF
CD3 on naive CD8+ T cell	GCST90001838	blood protein measurement	IPF
CD3 on naive CD8+ T cell	GCST90001838	blood protein measurement	IPF
CD3 on naive CD8+ T cell	GCST90001838	blood protein measurement	IPF
CD3 on naive CD8+ T cell	GCST90001838	blood protein measurement	IPF
CD3 on naive CD8+ T cell	GCST90001838	blood protein measurement	IPF
CD3 on naive CD8+ T cell	GCST90001838	blood protein measurement	IPF
CD20 on CD24+ CD27+ B cell	GCST90001745	blood protein measurement	IPF
CD20 on CD24+ CD27+ B cell	GCST90001745	blood protein measurement	IPF
CD20 on CD24+ CD27+ B cell	GCST90001745	blood protein measurement	IPF
CD20 on CD24+ CD27+ B cell	GCST90001745	blood protein measurement	IPF
CD20 on CD24+ CD27+ B cell	GCST90001745	blood protein measurement	IPF
CD20 on IgD- CD38dim B cell	GCST90001757	blood protein measurement	IPF
CD20 on IgD- CD38dim B cell	GCST90001757	blood protein measurement	IPF
CD20 on IgD- CD38dim B cell	GCST90001757	blood protein measurement	IPF
CD20 on IgD- CD38dim B cell	GCST90001757	blood protein measurement	IPF
CD20 on IgD- CD38dim B cell	GCST90001757	blood protein measurement	IPF
CD20 on IgD- CD38dim B cell	GCST90001757	blood protein measurement	IPF
CD25 on CD39+ CD4+ T cell	GCST90001961	blood protein measurement	IPF
CD25 on CD39+ CD4+ T cell	GCST90001961	blood protein measurement	IPF
CD25 on CD39+ CD4+ T cell	GCST90001961	blood protein measurement	IPF
CD25 on CD39+ CD4+ T cell	GCST90001961	blood protein measurement	IPF
CD25 on CD39+ CD4+ T cell	GCST90001961	blood protein measurement	IPF
Effector Memory CD4-CD8- T cell Abso	GCST90001569	lymphocyte count	IPF
Effector Memory CD4-CD8- T cell Abso	GCST90001569	lymphocyte count	IPF
Effector Memory CD4-CD8- T cell Abso	GCST90001569	lymphocyte count	IPF
Effector Memory CD4-CD8- T cell Abso	GCST90001569	lymphocyte count	IPF
Effector Memory CD4-CD8- T cell %CD	GCST90001570	lymphocyte count	IPF
Effector Memory CD4-CD8- T cell %CD	GCST90001570	lymphocyte count	IPF
Terminally Differentiated CD4-CD8- T c	GCST90001574	lymphocyte count	IPF

Terminally Differentiated CD4-CD8- T c	GCST90001574	lymphocyte count	IPF
HLA DR on B cell	GCST90002116	blood protein measurement	IPF
HLA DR on B cell	GCST90002116	blood protein measurement	IPF
HLA DR on B cell	GCST90002116	blood protein measurement	IPF
HLA DR on B cell	GCST90002116	blood protein measurement	IPF
HLA DR on B cell	GCST90002116	blood protein measurement	IPF
HLA DR on B cell	GCST90002116	blood protein measurement	IPF
HLA DR on B cell	GCST90002116	blood protein measurement	IPF
CD16-CD56 on Natural Killer T	GCST90001883	blood protein measurement	IPF
CD16-CD56 on Natural Killer T	GCST90001883	blood protein measurement	IPF
CD16-CD56 on Natural Killer T	GCST90001883	blood protein measurement	IPF
CD16-CD56 on Natural Killer T	GCST90001883	blood protein measurement	IPF
CD28+ CD45RA+ CD8+ T cell %T cell	GCST90001688	lymphocyte count	IPF
CD28+ CD45RA+ CD8+ T cell %T cell	GCST90001688	lymphocyte count	IPF
CD28+ CD45RA+ CD8+ T cell %T cell	GCST90001688	lymphocyte count	IPF
CD28+ CD45RA+ CD8+ T cell %T cell	GCST90001688	lymphocyte count	IPF
CD28+ CD45RA+ CD8+ T cell %T cell	GCST90001688	lymphocyte count	IPF
CD28+ CD45RA+ CD8+ T cell %T cell	GCST90001688	lymphocyte count	IPF
CD28+ CD45RA+ CD8+ T cell %T cell	GCST90001688	lymphocyte count	IPF
CD28+ CD45RA+ CD8+ T cell %T cell	GCST90001688	lymphocyte count	IPF
CD28+ CD45RA+ CD8+ T cell %T cell	GCST90001688	lymphocyte count	IPF
CD28+ CD45RA+ CD8+ T cell %T cell	GCST90001688	lymphocyte count	IPF
CD28+ CD45RA+ CD8+ T cell %T cell	GCST90001688	lymphocyte count	IPF
CD28+ CD45RA+ CD8+ T cell %T cell	GCST90001688	lymphocyte count	IPF
CD28+ CD45RA+ CD8+ T cell %T cell	GCST90001688	lymphocyte count	IPF
CD28+ CD45RA+ CD8+ T cell %T cell	GCST90001688	lymphocyte count	IPF
CD28+ CD45RA+ CD8+ T cell %T cell	GCST90001688	lymphocyte count	IPF
CD28+ CD45RA+ CD8+ T cell %T cell	GCST90001688	lymphocyte count	IPF
CD28+ CD45RA+ CD8+ T cell %T cell	GCST90001688	lymphocyte count	IPF
CD28+ CD45RA+ CD8+ T cell %T cell	GCST90001688	lymphocyte count	IPF
CD20 on unswitched memory B cell	GCST90001760	blood protein measurement	IPF
CD20 on unswitched memory B cell	GCST90001760	blood protein measurement	IPF
CD20 on unswitched memory B cell	GCST90001760	blood protein measurement	IPF
CD20 on unswitched memory B cell	GCST90001760	blood protein measurement	IPF
CD19 on IgD- CD38dim B cell	GCST90001735	blood protein measurement	IPF
CD19 on IgD- CD38dim B cell	GCST90001735	blood protein measurement	IPF
CD19 on IgD+ CD38dim B cell	GCST90001730	blood protein measurement	IPF
CD19 on IgD+ CD38dim B cell	GCST90001730	blood protein measurement	IPF
CD19 on IgD+ CD38dim B cell	GCST90001730	blood protein measurement	IPF
IgD on IgD+ CD24+ B cell	GCST90001820	blood protein measurement	IPF
IgD on IgD+ CD24+ B cell	GCST90001820	blood protein measurement	IPF
IgD on IgD+ CD24+ B cell	GCST90001820	blood protein measurement	IPF
CD62L- monocyte Absolute Count	GCST90001450	leukocyte count	IPF
CD62L- monocyte Absolute Count	GCST90001450	leukocyte count	IPF
Activated & secreting CD4 regulatory T c	GCST90001503	lymphocyte count	IPF
Activated & secreting CD4 regulatory T c	GCST90001503	lymphocyte count	IPF
Activated & secreting CD4 regulatory T c	GCST90001503	lymphocyte count	IPF
Activated & secreting CD4 regulatory T c	GCST90001503	lymphocyte count	IPF
Activated & secreting CD4 regulatory T c	GCST90001503	lymphocyte count	IPF
Activated & secreting CD4 regulatory T c	GCST90001503	lymphocyte count	IPF
CD28 on CD28+ CD45RA+ CD8+ T cell	GCST90001896	blood protein measurement	IPF
CD28 on CD28+ CD45RA+ CD8+ T cell	GCST90001896	blood protein measurement	IPF
CD28+ CD45RA+ CD8dim T cell Absolu	GCST90001666	lymphocyte count	IPF
CD28+ CD45RA+ CD8dim T cell Absolu	GCST90001666	lymphocyte count	IPF
CD28+ CD45RA+ CD8dim T cell Absolu	GCST90001666	lymphocyte count	IPF
CD28+ CD45RA+ CD8dim T cell Absolu	GCST90001666	lymphocyte count	IPF
CD28+ CD45RA+ CD8dim T cell Absolu	GCST90001666	lymphocyte count	IPF
CD25 on activated & secreting CD4 regu	GCST90001943	blood protein measurement	IPF

CD25 on CD39+ secreting CD4 regulator	GCST90001942	blood protein measurement	IPF
SSC-A on myeloid Dendritic Cell	GCST90002071	blood protein measurement	IPF
SSC-A on myeloid Dendritic Cell	GCST90002071	blood protein measurement	IPF
SSC-A on myeloid Dendritic Cell	GCST90002071	blood protein measurement	IPF
SSC-A on myeloid Dendritic Cell	GCST90002071	blood protein measurement	IPF
IgD+ CD38- B cell %B cell	GCST90001395	lymphocyte count	IPF
Activated CD4 regulatory T cell Absolute	GCST90001486	lymphocyte count	IPF
Activated CD4 regulatory T cell Absolute	GCST90001486	lymphocyte count	IPF
Central Memory CD8+ T cell Absolute C	GCST90001548	lymphocyte count	IPF
Effector Memory CD4-CD8- T cell %T c	GCST90001571	lymphocyte count	IPF
Effector Memory CD4-CD8- T cell %T c	GCST90001571	lymphocyte count	IPF
Effector Memory CD4-CD8- T cell %T c	GCST90001571	lymphocyte count	IPF
BAFF-R on CD20- B cell	GCST90001830	blood protein measurement	IPF
CD19 on IgD+ CD38- unswitched memor	GCST90001728	blood protein measurement	IPF
CD19 on IgD+ CD38- unswitched memor	GCST90001728	blood protein measurement	IPF
CD19 on IgD+ CD38- unswitched memor	GCST90001728	blood protein measurement	IPF
CD19 on transitional B cell	GCST90001742	blood protein measurement	IPF
CD19 on transitional B cell	GCST90001742	blood protein measurement	IPF
CD19 on transitional B cell	GCST90001742	blood protein measurement	IPF
CD19 on unswitched memory B cell	GCST90001738	blood protein measurement	IPF
CD19 on unswitched memory B cell	GCST90001738	blood protein measurement	IPF
CD19 on unswitched memory B cell	GCST90001738	blood protein measurement	IPF
CD19 on IgD+ CD24- B cell	GCST90001725	blood protein measurement	IPF
CD19 on IgD+ CD24- B cell	GCST90001725	blood protein measurement	IPF
CD19 on IgD+ CD24- B cell	GCST90001725	blood protein measurement	IPF
CD19 on IgD+ B cell	GCST90001741	blood protein measurement	IPF
CD19 on IgD+ B cell	GCST90001741	blood protein measurement	IPF
CD19 on IgD+ CD38+ B cell	GCST90001729	blood protein measurement	IPF
CD19 on IgD+ CD38+ B cell	GCST90001729	blood protein measurement	IPF
CD19 on IgD+ CD38+ B cell	GCST90001729	blood protein measurement	IPF
CD19 on IgD+ CD24+ B cell	GCST90001724	blood protein measurement	IPF
CD19 on IgD+ CD24+ B cell	GCST90001724	blood protein measurement	IPF
CD19 on IgD+ CD24+ B cell	GCST90001724	blood protein measurement	IPF
Immature Myeloid-Derived Suppressor C	GCST90001516	myeloid white cell count	IPF
Immature Myeloid-Derived Suppressor C	GCST90001516	myeloid white cell count	IPF
CD45RA- CD28- CD8+ T cell %CD8+ T	GCST90001696	lymphocyte count	IPF
CD45RA- CD28- CD8+ T cell %CD8+ T	GCST90001696	lymphocyte count	IPF
CD45RA- CD28- CD8+ T cell %CD8+ T	GCST90001696	lymphocyte count	IPF
CD45RA- CD28- CD8+ T cell %CD8+ T	GCST90001696	lymphocyte count	IPF
CD45RA- CD28- CD8+ T cell %CD8+ T	GCST90001696	lymphocyte count	IPF
CD45RA- CD28- CD8+ T cell %CD8+ T	GCST90001696	lymphocyte count	IPF
CD45RA- CD28- CD8+ T cell %CD8+ T	GCST90001696	lymphocyte count	IPF
CD33dim HLA DR- Absolute Count	GCST90001531	myeloid white cell count	IPF
CD33dim HLA DR- Absolute Count	GCST90001531	myeloid white cell count	IPF
Basophil Absolute Count	GCST90001532	myeloid white cell count	IPF
Basophil Absolute Count	GCST90001532	myeloid white cell count	IPF
Basophil Absolute Count	GCST90001532	myeloid white cell count	IPF
CD3 on secreting CD4 regulatory T cell	GCST90001855	blood protein measurement	IPF
CD3 on secreting CD4 regulatory T cell	GCST90001855	blood protein measurement	IPF
CD3 on secreting CD4 regulatory T cell	GCST90001855	blood protein measurement	IPF
CD3 on CD4 regulatory T cell	GCST90001868	blood protein measurement	IPF
CD3 on CD4 regulatory T cell	GCST90001868	blood protein measurement	IPF
CD3 on CD4 regulatory T cell	GCST90001868	blood protein measurement	IPF
CD3 on activated & secreting CD4 regula	GCST90001857	blood protein measurement	IPF
CD3 on activated & secreting CD4 regula	GCST90001857	blood protein measurement	IPF
CD3 on activated & secreting CD4 regula	GCST90001857	blood protein measurement	IPF
CD3 on activated CD4 regulatory T cell	GCST90001853	blood protein measurement	IPF
CD3 on activated CD4 regulatory T cell	GCST90001853	blood protein measurement	IPF

CD3 on activated CD4 regulatory T cell	GCST90001853	blood protein measurement	IPF
CD45RA+ CD8+ T cell Absolute Count	GCST90001560	lymphocyte count	IPF
CD38 on IgD+ CD24- B cell	GCST90001811	blood protein measurement	IPF
CD38 on IgD+ CD24- B cell	GCST90001811	blood protein measurement	IPF
CD38 on naive-mature B cell	GCST90001816	blood protein measurement	IPF
CD38 on naive-mature B cell	GCST90001816	blood protein measurement	IPF
CD38 on naive-mature B cell	GCST90001816	blood protein measurement	IPF
CD38 on transitional B cell	GCST90001819	blood protein measurement	IPF
CD38 on transitional B cell	GCST90001819	blood protein measurement	IPF
CD38 on transitional B cell	GCST90001819	blood protein measurement	IPF
CD38 on transitional B cell	GCST90001819	blood protein measurement	IPF
CD38 on transitional B cell	GCST90001819	blood protein measurement	IPF
CD38 on IgD- CD38dim B cell	GCST90001815	blood protein measurement	IPF
CD38 on IgD- CD38dim B cell	GCST90001815	blood protein measurement	IPF
CD38 on IgD- CD38dim B cell	GCST90001815	blood protein measurement	IPF
CD38 on IgD+ CD38dim B cell	GCST90001813	blood protein measurement	IPF
CD38 on IgD+ CD38dim B cell	GCST90001813	blood protein measurement	IPF
CD38 on IgD+ CD38dim B cell	GCST90001813	blood protein measurement	IPF
CD38 on IgD+ CD38dim B cell	GCST90001813	blood protein measurement	IPF
CD14+ CD16- monocyte %monocyte	GCST90001586	monocyte count	IPF
CD14+ CD16- monocyte %monocyte	GCST90001586	monocyte count	IPF
CD14- CD16+ monocyte %monocyte	GCST90001584	monocyte count	IPF
CD14- CD16+ monocyte %monocyte	GCST90001584	monocyte count	IPF
CD14- CD16+ monocyte Absolute Count	GCST90001579	monocyte count	IPF
CCR2 on CD14+ CD16+ monocyte	GCST90001992	blood protein measurement	IPF
CCR2 on CD14+ CD16+ monocyte	GCST90001992	blood protein measurement	IPF
CD16+ monocyte %monocyte	GCST90001587	monocyte count	IPF
CD16+ monocyte %monocyte	GCST90001587	monocyte count	IPF
CD64 on CD14- CD16+ monocyte	GCST90001990	blood protein measurement	IPF
CD8dim Natural Killer T %lymphocyte	GCST90001635	leukocyte count	IPF
CD8dim Natural Killer T %lymphocyte	GCST90001635	leukocyte count	IPF
CD8dim Natural Killer T %lymphocyte	GCST90001635	leukocyte count	IPF
CD8dim Natural Killer T %lymphocyte	GCST90001635	leukocyte count	IPF
CD8dim Natural Killer T %lymphocyte	GCST90001635	leukocyte count	IPF
CD8dim Natural Killer T %lymphocyte	GCST90001635	leukocyte count	IPF
CD8dim Natural Killer T %T cell	GCST90001634	leukocyte count	IPF
CD8dim Natural Killer T %T cell	GCST90001634	leukocyte count	IPF
CD8dim Natural Killer T %T cell	GCST90001634	leukocyte count	IPF
CD8dim Natural Killer T %T cell	GCST90001634	leukocyte count	IPF
CD8dim Natural Killer T %T cell	GCST90001634	leukocyte count	IPF
CD25 on IgD- CD24- B cell	GCST90001785	blood protein measurement	IPF
CD25 on IgD- CD24- B cell	GCST90001785	blood protein measurement	IPF
CD4-CD8- Natural Killer T %lymphocyt	GCST90001638	leukocyte count	IPF
CD4-CD8- Natural Killer T %lymphocyt	GCST90001638	leukocyte count	IPF
CD4-CD8- Natural Killer T %lymphocyt	GCST90001638	leukocyte count	IPF
CD4-CD8- Natural Killer T %lymphocyt	GCST90001638	leukocyte count	IPF
CD4-CD8- Natural Killer T %lymphocyt	GCST90001638	leukocyte count	IPF
Natural Killer T %T cell	GCST90001622	leukocyte count	IPF
Natural Killer T %T cell	GCST90001622	leukocyte count	IPF
Natural Killer T %T cell	GCST90001622	leukocyte count	IPF
Natural Killer T %T cell	GCST90001622	leukocyte count	IPF
Natural Killer T %T cell	GCST90001622	leukocyte count	IPF
CD8dim Natural Killer T Absolute Count	GCST90001633	leukocyte count	IPF
CD8dim Natural Killer T Absolute Count	GCST90001633	leukocyte count	IPF
CD8dim Natural Killer T Absolute Count	GCST90001633	leukocyte count	IPF
CD8dim Natural Killer T Absolute Count	GCST90001633	leukocyte count	IPF
CD8dim Natural Killer T Absolute Count	GCST90001633	leukocyte count	IPF
Natural Killer T %lymphocyte	GCST90001623	leukocyte count	IPF

Natural Killer T %lymphocyte	GCST90001623	leukocyte count	IPF
Natural Killer T %lymphocyte	GCST90001623	leukocyte count	IPF
Natural Killer T %lymphocyte	GCST90001623	leukocyte count	IPF
Natural Killer T %lymphocyte	GCST90001623	leukocyte count	IPF
Natural Killer T %lymphocyte	GCST90001623	leukocyte count	IPF
Natural Killer T %lymphocyte	GCST90001623	leukocyte count	IPF
CD4-CD8- Natural Killer T Absolute Cou	GCST90001636	leukocyte count	IPF
CD4-CD8- Natural Killer T Absolute Cou	GCST90001636	leukocyte count	IPF
CD4-CD8- Natural Killer T Absolute Cou	GCST90001636	leukocyte count	IPF
CD4-CD8- Natural Killer T Absolute Cou	GCST90001636	leukocyte count	IPF
CD4-CD8- Natural Killer T Absolute Cou	GCST90001636	leukocyte count	IPF
CD4-CD8- Natural Killer T %T cell	GCST90001637	leukocyte count	IPF
CD4-CD8- Natural Killer T %T cell	GCST90001637	leukocyte count	IPF
CD4-CD8- Natural Killer T %T cell	GCST90001637	leukocyte count	IPF
CD4-CD8- Natural Killer T %T cell	GCST90001637	leukocyte count	IPF
SSC-A on CD14+ monocyte	GCST90002074	blood protein measurement	IPF
SSC-A on CD14+ monocyte	GCST90002074	blood protein measurement	IPF
SSC-A on CD14+ monocyte	GCST90002074	blood protein measurement	IPF
SSC-A on CD14+ monocyte	GCST90002074	blood protein measurement	IPF
CD86+ myeloid Dendritic Cell %Dendrit	GCST90001465	leukocyte count	IPF
CD86+ myeloid Dendritic Cell %Dendrit	GCST90001465	leukocyte count	IPF
CD86+ myeloid Dendritic Cell %Dendrit	GCST90001465	leukocyte count	IPF
CD127 on CD45RA+ CD4+ T cell	GCST90001932	blood protein measurement	IPF
CD127 on CD45RA+ CD4+ T cell	GCST90001932	blood protein measurement	IPF
SSC-A on T cell	GCST90002080	blood protein measurement	IPF
CD39+ resting CD4 regulatory T cell %re	GCST90001484	lymphocyte count	IPF
CD39+ resting CD4 regulatory T cell %re	GCST90001484	lymphocyte count	IPF
CD39+ resting CD4 regulatory T cell %re	GCST90001484	lymphocyte count	IPF
CD39+ resting CD4 regulatory T cell %re	GCST90001484	lymphocyte count	IPF
CD39+ resting CD4 regulatory T cell %re	GCST90001484	lymphocyte count	IPF
CD39+ resting CD4 regulatory T cell %re	GCST90001484	lymphocyte count	IPF
CD39+ resting CD4 regulatory T cell %re	GCST90001484	lymphocyte count	IPF
CD39+ resting CD4 regulatory T cell %re	GCST90001484	lymphocyte count	IPF
Secreting CD4 regulatory T cell %CD4+	GCST90001494	lymphocyte count	IPF
Secreting CD4 regulatory T cell %CD4+	GCST90001494	lymphocyte count	IPF
Secreting CD4 regulatory T cell %CD4+	GCST90001494	lymphocyte count	IPF
Secreting CD4 regulatory T cell %CD4+	GCST90001494	lymphocyte count	IPF
Secreting CD4 regulatory T cell %CD4+	GCST90001494	lymphocyte count	IPF
Secreting CD4 regulatory T cell %CD4+	GCST90001494	lymphocyte count	IPF
CD25++ CD45RA- CD4 not regulatory T	GCST90001512	lymphocyte count	IPF
CD25++ CD45RA- CD4 not regulatory T	GCST90001512	lymphocyte count	IPF
CD4RA on Terminally Differentiated CD	GCST90002099	blood protein measurement	IPF
CD4RA on Terminally Differentiated CD	GCST90002099	blood protein measurement	IPF
Resting CD4 regulatory T cell %CD4 reg	GCST90001481	lymphocyte count	IPF
Resting CD4 regulatory T cell %CD4 reg	GCST90001481	lymphocyte count	IPF
Resting CD4 regulatory T cell %CD4 reg	GCST90001481	lymphocyte count	IPF
Resting CD4 regulatory T cell %CD4 reg	GCST90001481	lymphocyte count	IPF
Resting CD4 regulatory T cell %CD4 reg	GCST90001481	lymphocyte count	IPF
CD39+ resting CD4 regulatory T cell %C	GCST90001485	lymphocyte count	IPF
CD39+ resting CD4 regulatory T cell %C	GCST90001485	lymphocyte count	IPF
CD39+ resting CD4 regulatory T cell %C	GCST90001485	lymphocyte count	IPF
CD39+ resting CD4 regulatory T cell %C	GCST90001485	lymphocyte count	IPF
CD39+ resting CD4 regulatory T cell %C	GCST90001485	lymphocyte count	IPF
CD39+ resting CD4 regulatory T cell %C	GCST90001485	lymphocyte count	IPF
CD39+ resting CD4 regulatory T cell %C	GCST90001485	lymphocyte count	IPF
CD39+ resting CD4 regulatory T cell %C	GCST90001485	lymphocyte count	IPF
CD39+ resting CD4 regulatory T cell %C	GCST90001485	lymphocyte count	IPF
CD39+ resting CD4 regulatory T cell %C	GCST90001485	lymphocyte count	IPF

CD39+ resting CD4 regulatory T cell %C	GCST90001485	lymphocyte count	IPF
CD28+ CD45RA+ CD8dim T cell %T ce	GCST90001664	lymphocyte count	IPF
CD28+ CD45RA+ CD8dim T cell %T ce	GCST90001664	lymphocyte count	IPF
CD28+ CD45RA+ CD8dim T cell %T ce	GCST90001664	lymphocyte count	IPF
CD28+ CD45RA+ CD8dim T cell %T ce	GCST90001664	lymphocyte count	IPF
Activated & resting CD4 regulatory T cel	GCST90001500	lymphocyte count	IPF
Activated & resting CD4 regulatory T cel	GCST90001500	lymphocyte count	IPF
Activated & resting CD4 regulatory T cel	GCST90001500	lymphocyte count	IPF
Activated & secreting CD4 regulatory T c	GCST90001502	lymphocyte count	IPF
Activated & secreting CD4 regulatory T c	GCST90001502	lymphocyte count	IPF
Activated & secreting CD4 regulatory T c	GCST90001502	lymphocyte count	IPF
Activated & secreting CD4 regulatory T c	GCST90001502	lymphocyte count	IPF
Activated & secreting CD4 regulatory T c	GCST90001502	lymphocyte count	IPF
Activated & secreting CD4 regulatory T c	GCST90001502	lymphocyte count	IPF
Secreting CD4 regulatory T cell Absolute	GCST90001492	lymphocyte count	IPF
Secreting CD4 regulatory T cell Absolute	GCST90001492	lymphocyte count	IPF
Secreting CD4 regulatory T cell Absolute	GCST90001492	lymphocyte count	IPF
CD28+ CD45RA+ CD8dim T cell %CD8	GCST90001665	lymphocyte count	IPF
CD28+ CD45RA+ CD8dim T cell %CD8	GCST90001665	lymphocyte count	IPF
CD28+ CD45RA+ CD8dim T cell %CD8	GCST90001665	lymphocyte count	IPF
Activated & resting CD4 regulatory T cel	GCST90001499	lymphocyte count	IPF
Activated & resting CD4 regulatory T cel	GCST90001499	lymphocyte count	IPF
Activated & resting CD4 regulatory T cel	GCST90001499	lymphocyte count	IPF
CD28+ CD45RA- CD8dim T cell Absolu	GCST90001669	lymphocyte count	IPF
CD28+ CD45RA- CD8dim T cell Absolu	GCST90001669	lymphocyte count	IPF
CD28+ CD45RA- CD8+ T cell Absolute	GCST90001693	lymphocyte count	IPF
CD39+ resting CD4 regulatory T cell Abs	GCST90001483	lymphocyte count	IPF
CD39+ resting CD4 regulatory T cell Abs	GCST90001483	lymphocyte count	IPF
CD39+ resting CD4 regulatory T cell Abs	GCST90001483	lymphocyte count	IPF
CD39+ resting CD4 regulatory T cell Abs	GCST90001483	lymphocyte count	IPF
CD39+ resting CD4 regulatory T cell Abs	GCST90001483	lymphocyte count	IPF
CD39+ resting CD4 regulatory T cell Abs	GCST90001483	lymphocyte count	IPF
CD39+ resting CD4 regulatory T cell Abs	GCST90001483	lymphocyte count	IPF
CD39+ resting CD4 regulatory T cell Abs	GCST90001483	lymphocyte count	IPF
CD39+ resting CD4 regulatory T cell Abs	GCST90001483	lymphocyte count	IPF
CD39+ resting CD4 regulatory T cell Abs	GCST90001483	lymphocyte count	IPF
Resting CD4 regulatory T cell %CD4+ T	GCST90001482	lymphocyte count	IPF
Resting CD4 regulatory T cell %CD4+ T	GCST90001482	lymphocyte count	IPF
Resting CD4 regulatory T cell %CD4+ T	GCST90001482	lymphocyte count	IPF
Resting CD4 regulatory T cell %CD4+ T	GCST90001482	lymphocyte count	IPF
CD28+ CD45RA- CD8dim T cell %T cel	GCST90001667	lymphocyte count	IPF
CD28+ CD45RA- CD8dim T cell %T cel	GCST90001667	lymphocyte count	IPF
Secreting CD4 regulatory T cell %CD4 re	GCST90001493	lymphocyte count	IPF
Secreting CD4 regulatory T cell %CD4 re	GCST90001493	lymphocyte count	IPF
Secreting CD4 regulatory T cell %CD4 re	GCST90001493	lymphocyte count	IPF
CD28+ CD45RA- CD8dim T cell %CD8	GCST90001668	lymphocyte count	IPF
Activated & secreting CD4 regulatory T c	GCST90001501	lymphocyte count	IPF
Activated & secreting CD4 regulatory T c	GCST90001501	lymphocyte count	IPF
Activated & secreting CD4 regulatory T c	GCST90001501	lymphocyte count	IPF
CD45RA on naive CD4+ T cell	GCST90002098	blood protein measurement	IPF
CD45RA on naive CD4+ T cell	GCST90002098	blood protein measurement	IPF
CD45RA on naive CD4+ T cell	GCST90002098	blood protein measurement	IPF
CD45RA on naive CD4+ T cell	GCST90002098	blood protein measurement	IPF
CD45RA on naive CD4+ T cell	GCST90002098	blood protein measurement	IPF
CD45RA on naive CD4+ T cell	GCST90002098	blood protein measurement	IPF
CD45RA on naive CD4+ T cell	GCST90002098	blood protein measurement	IPF
CD45RA on naive CD4+ T cell	GCST90002098	blood protein measurement	IPF
CD25 on CD24+ CD27+ B cell	GCST90001777	blood protein measurement	IPF

CD25 on CD24+ CD27+ B cell	GCST90001777	blood protein measurement	IPF
CD25 on CD24+ CD27+ B cell	GCST90001777	blood protein measurement	IPF
CD25 on memory B cell	GCST90001790	blood protein measurement	IPF
CD25 on memory B cell	GCST90001790	blood protein measurement	IPF
CD25 on memory B cell	GCST90001790	blood protein measurement	IPF
CD25 on unswitched memory B cell	GCST90001792	blood protein measurement	IPF
CD25 on unswitched memory B cell	GCST90001792	blood protein measurement	IPF
CD25 on unswitched memory B cell	GCST90001792	blood protein measurement	IPF
CD25 on IgD+ CD24+ B cell	GCST90001778	blood protein measurement	IPF
CD25 on IgD+ CD24+ B cell	GCST90001778	blood protein measurement	IPF
CD45 on CD8+ T cell	GCST90001917	blood protein measurement	IPF
CD45 on CD8+ T cell	GCST90001917	blood protein measurement	IPF
CD4 on CD28+ CD4+ T cell	GCST90002062	blood protein measurement	IPF
CD4 on CD28+ CD4+ T cell	GCST90002062	blood protein measurement	IPF
CD4 on CD39+ activated CD4 regulatory	GCST90002067	blood protein measurement	IPF
CD4 on CD39+ activated CD4 regulatory	GCST90002067	blood protein measurement	IPF
CD4 on CD39+ activated CD4 regulatory	GCST90002067	blood protein measurement	IPF
CD4 on secreting CD4 regulatory T cell	GCST90002068	blood protein measurement	IPF
CD4 on activated & secreting CD4 regula	GCST90002070	blood protein measurement	IPF
CD4 on activated & secreting CD4 regula	GCST90002070	blood protein measurement	IPF
CD4 on CD45RA+ CD4+ T cell	GCST90002027	blood protein measurement	IPF
CD4 on CD4 regulatory T cell	GCST90002063	blood protein measurement	IPF
CD4 on Central Memory CD4+ T cell	GCST90002023	blood protein measurement	IPF
CD4 on Effector Memory CD4+ T cell	GCST90002025	blood protein measurement	IPF
CD4 on resting CD4 regulatory T cell	GCST90002064	blood protein measurement	IPF
CD4 on activated CD4 regulatory T cell	GCST90002066	blood protein measurement	IPF
CD4 on activated CD4 regulatory T cell	GCST90002066	blood protein measurement	IPF
CD64 on CD14+ CD16- monocyte	GCST90001987	blood protein measurement	IPF
CD64 on CD14+ CD16- monocyte	GCST90001987	blood protein measurement	IPF
CD64 on CD14+ CD16- monocyte	GCST90001987	blood protein measurement	IPF
CD64 on CD14+ CD16- monocyte	GCST90001987	blood protein measurement	IPF
CD64 on monocyte	GCST90002006	blood protein measurement	IPF
CD64 on monocyte	GCST90002006	blood protein measurement	IPF
CD64 on monocyte	GCST90002006	blood protein measurement	IPF
CD64 on monocyte	GCST90002006	blood protein measurement	IPF
HLA DR+ CD4+ T cell %T cell	GCST90001625	leukocyte count	IPF
CD127 on CD28+ CD45RA- CD8+ T cel	GCST90001928	blood protein measurement	IPF
CD19 on CD24+ CD27+ B cell	GCST90001723	blood protein measurement	IPF
CD19 on CD24+ CD27+ B cell	GCST90001723	blood protein measurement	IPF
CD19 on memory B cell	GCST90001736	blood protein measurement	IPF
CD19 on memory B cell	GCST90001736	blood protein measurement	IPF
HLA DR on plasmacytoid Dendritic Cell	GCST90002105	blood protein measurement	IPF
HLA DR on plasmacytoid Dendritic Cell	GCST90002105	blood protein measurement	IPF
HLA DR on plasmacytoid Dendritic Cell	GCST90002105	blood protein measurement	IPF
CD45RA on naive CD8+ T cell	GCST90002100	blood protein measurement	IPF
CD45RA on naive CD8+ T cell	GCST90002100	blood protein measurement	IPF
Memory B cell %lymphocyte	GCST90001436	lymphocyte count	IPF
Memory B cell %lymphocyte	GCST90001436	lymphocyte count	IPF
Switched memory B cell %lymphocyte	GCST90001434	lymphocyte count	IPF
Switched memory B cell %lymphocyte	GCST90001434	lymphocyte count	IPF
Switched memory B cell %lymphocyte	GCST90001434	lymphocyte count	IPF
IgD+ CD24+ B cell %lymphocyte	GCST90001439	lymphocyte count	IPF
IgD+ CD24+ B cell %lymphocyte	GCST90001439	lymphocyte count	IPF
IgD+ B cell %Lymphocyte	GCST90001424	lymphocyte count	IPF
IgD+ B cell %Lymphocyte	GCST90001424	lymphocyte count	IPF
CD24+ CD27+ B cell %lymphocyte	GCST90001442	lymphocyte count	IPF
CD24+ CD27+ B cell %lymphocyte	GCST90001442	lymphocyte count	IPF
Memory B cell %B cell	GCST90001406	lymphocyte count	IPF

IgD- CD38dim B cell %lymphocyte	GCST90001426	lymphocyte count	IPF
IgD- CD38dim B cell %lymphocyte	GCST90001426	lymphocyte count	IPF
FSC-A on plasmacytoid Dendritic Cell	GCST90001964	blood protein measurement	IPF
FSC-A on plasmacytoid Dendritic Cell	GCST90001964	blood protein measurement	IPF
FSC-A on plasmacytoid Dendritic Cell	GCST90001964	blood protein measurement	IPF
BAFF-R on CD24+ CD27+ B cell	GCST90001702	blood protein measurement	IPF
BAFF-R on CD24+ CD27+ B cell	GCST90001702	blood protein measurement	IPF
BAFF-R on CD24+ CD27+ B cell	GCST90001702	blood protein measurement	IPF
BAFF-R on CD24+ CD27+ B cell	GCST90001702	blood protein measurement	IPF
BAFF-R on CD24+ CD27+ B cell	GCST90001702	blood protein measurement	IPF
BAFF-R on CD24+ CD27+ B cell	GCST90001702	blood protein measurement	IPF
BAFF-R on CD24+ CD27+ B cell	GCST90001702	blood protein measurement	IPF
BAFF-R on memory B cell	GCST90001715	blood protein measurement	IPF
BAFF-R on memory B cell	GCST90001715	blood protein measurement	IPF
BAFF-R on memory B cell	GCST90001715	blood protein measurement	IPF
BAFF-R on memory B cell	GCST90001715	blood protein measurement	IPF
BAFF-R on memory B cell	GCST90001715	blood protein measurement	IPF
BAFF-R on IgD- CD38- B cell	GCST90001712	blood protein measurement	IPF
BAFF-R on IgD- CD38- B cell	GCST90001712	blood protein measurement	IPF
BAFF-R on IgD- CD38- B cell	GCST90001712	blood protein measurement	IPF
BAFF-R on IgD- CD38- B cell	GCST90001712	blood protein measurement	IPF
BAFF-R on IgD- CD38- B cell	GCST90001712	blood protein measurement	IPF
BAFF-R on unswitched memory B cell	GCST90001717	blood protein measurement	IPF
BAFF-R on unswitched memory B cell	GCST90001717	blood protein measurement	IPF
BAFF-R on unswitched memory B cell	GCST90001717	blood protein measurement	IPF
BAFF-R on unswitched memory B cell	GCST90001717	blood protein measurement	IPF
BAFF-R on unswitched memory B cell	GCST90001717	blood protein measurement	IPF
BAFF-R on switched memory B cell	GCST90001718	blood protein measurement	IPF
BAFF-R on switched memory B cell	GCST90001718	blood protein measurement	IPF
BAFF-R on switched memory B cell	GCST90001718	blood protein measurement	IPF
BAFF-R on switched memory B cell	GCST90001718	blood protein measurement	IPF
BAFF-R on switched memory B cell	GCST90001718	blood protein measurement	IPF
BAFF-R on switched memory B cell	GCST90001718	blood protein measurement	IPF
BAFF-R on switched memory B cell	GCST90001718	blood protein measurement	IPF
BAFF-R on switched memory B cell	GCST90001718	blood protein measurement	IPF
BAFF-R on IgD- CD38dim B cell	GCST90001714	blood protein measurement	IPF
BAFF-R on IgD- CD38dim B cell	GCST90001714	blood protein measurement	IPF
BAFF-R on IgD- CD38dim B cell	GCST90001714	blood protein measurement	IPF
BAFF-R on IgD- CD38dim B cell	GCST90001714	blood protein measurement	IPF
BAFF-R on IgD- CD38dim B cell	GCST90001714	blood protein measurement	IPF
BAFF-R on IgD- CD38dim B cell	GCST90001714	blood protein measurement	IPF
CD28- CD8dim T cell %T cell	GCST90001661	lymphocyte count	IPF
CD28- CD8dim T cell %T cell	GCST90001661	lymphocyte count	IPF
CD28- CD8dim T cell %T cell	GCST90001661	lymphocyte count	IPF
CD40 on CD14+ CD16- monocyte	GCST90001980	blood protein measurement	IPF
CD40 on CD14+ CD16- monocyte	GCST90001980	blood protein measurement	IPF
CD40 on CD14+ CD16- monocyte	GCST90001980	blood protein measurement	IPF
CD40 on CD14+ CD16- monocyte	GCST90001980	blood protein measurement	IPF
CD20- CD38- B cell %B cell	GCST90001422	lymphocyte count	IPF
CD19 on CD20- B cell	GCST90001721	blood protein measurement	IPF
CD20 on IgD- CD38- B cell	GCST90001755	blood protein measurement	IPF
CD20 on IgD- CD38- B cell	GCST90001755	blood protein measurement	IPF
CD20 on IgD- CD38- B cell	GCST90001755	blood protein measurement	IPF
CD20 on IgD- CD38- B cell	GCST90001755	blood protein measurement	IPF
CD28 on activated & secreting CD4 regu	GCST90001889	blood protein measurement	IPF
CD28 on CD4 regulatory T cell	GCST90001899	blood protein measurement	IPF
CD28 on CD39+ resting CD4 regulatory	GCST90001901	blood protein measurement	IPF
CD28 on CD39+ resting CD4 regulatory	GCST90001901	blood protein measurement	IPF

BAFF-R on IgD+ CD24+ B cell	GCST90001703	blood protein measurement	IPF
BAFF-R on IgD+ CD24+ B cell	GCST90001703	blood protein measurement	IPF
BAFF-R on IgD+ CD24+ B cell	GCST90001703	blood protein measurement	IPF
BAFF-R on IgD+ CD24+ B cell	GCST90001703	blood protein measurement	IPF
CD3 on Natural Killer T	GCST90001848	blood protein measurement	IPF
CD3 on Natural Killer T	GCST90001848	blood protein measurement	IPF
CD39+ CD8+ T cell %T cell	GCST90001670	lymphocyte count	IPF
CD39+ CD8+ T cell %T cell	GCST90001670	lymphocyte count	IPF
CD39+ CD8+ T cell %T cell	GCST90001670	lymphocyte count	IPF
CD39+ CD8+ T cell %T cell	GCST90001670	lymphocyte count	IPF
CD39+ CD8+ T cell %T cell	GCST90001670	lymphocyte count	IPF
CD39+ CD8+ T cell %CD8+ T cell	GCST90001671	lymphocyte count	IPF
CD39+ CD8+ T cell %CD8+ T cell	GCST90001671	lymphocyte count	IPF
CD39+ CD8+ T cell %CD8+ T cell	GCST90001671	lymphocyte count	IPF
CD39+ CD8+ T cell %CD8+ T cell	GCST90001671	lymphocyte count	IPF
CD39+ CD8+ T cell %CD8+ T cell	GCST90001671	lymphocyte count	IPF
CD39+ CD8+ T cell %CD8+ T cell	GCST90001671	lymphocyte count	IPF
CD39+ CD8+ T cell Absolute Count	GCST90001672	lymphocyte count	IPF
CD39+ CD8+ T cell Absolute Count	GCST90001672	lymphocyte count	IPF
CD39+ CD8+ T cell Absolute Count	GCST90001672	lymphocyte count	IPF
CD39+ CD8+ T cell Absolute Count	GCST90001672	lymphocyte count	IPF
CD39+ CD8+ T cell Absolute Count	GCST90001672	lymphocyte count	IPF
CD39+ CD8+ T cell Absolute Count	GCST90001672	lymphocyte count	IPF
FSC-A on HLA DR+ Natural Killer	GCST90001970	blood protein measurement	IPF
FSC-A on HLA DR+ Natural Killer	GCST90001970	blood protein measurement	IPF
Effector Memory CD8+ T cell %CD8+ T	GCST90001555	lymphocyte count	IPF
Effector Memory CD8+ T cell %CD8+ T	GCST90001555	lymphocyte count	IPF
CD45RA+ CD8+ T cell %CD8+ T cell	GCST90001561	lymphocyte count	IPF
CD45RA+ CD8+ T cell %CD8+ T cell	GCST90001561	lymphocyte count	IPF
CD45RA+ CD8+ T cell %CD8+ T cell	GCST90001561	lymphocyte count	IPF
CD28+ CD45RA+ CD8+ T cell Absolute	GCST90001690	lymphocyte count	IPF
CD28+ CD45RA+ CD8+ T cell Absolute	GCST90001690	lymphocyte count	IPF
B cell %CD3- lymphocyte	GCST90001643	leukocyte count	IPF
B cell %CD3- lymphocyte	GCST90001643	leukocyte count	IPF
B cell %CD3- lymphocyte	GCST90001643	leukocyte count	IPF
Natural Killer %CD3- lymphocyte	GCST90001646	leukocyte count	IPF
Natural Killer %CD3- lymphocyte	GCST90001646	leukocyte count	IPF
Natural Killer %CD3- lymphocyte	GCST90001646	leukocyte count	IPF
Natural Killer %CD3- lymphocyte	GCST90001646	leukocyte count	IPF
Natural Killer %CD3- lymphocyte	GCST90001646	leukocyte count	IPF
HLA DR on CD14+ monocyte	GCST90001991	blood protein measurement	IPF
HLA DR on CD14+ monocyte	GCST90001991	blood protein measurement	IPF
Monocytic Myeloid-Derived Suppressor (	GCST90001530	myeloid white cell count	IPF
Monocytic Myeloid-Derived Suppressor (	GCST90001530	myeloid white cell count	IPF
Monocytic Myeloid-Derived Suppressor (	GCST90001530	myeloid white cell count	IPF
HLA DR on CD14+ CD16- monocyte	GCST90001988	blood protein measurement	IPF
HLA DR on CD14+ CD16- monocyte	GCST90001988	blood protein measurement	IPF
CD62L on CD62L+ myeloid Dendritic C	GCST90001831	blood protein measurement	IPF
CD62L on CD62L+ myeloid Dendritic C	GCST90001831	blood protein measurement	IPF
CD45 on Natural Killer T	GCST90001919	blood protein measurement	IPF
CD3 on resting CD4 regulatory T cell	GCST90001869	blood protein measurement	IPF
CD3 on resting CD4 regulatory T cell	GCST90001869	blood protein measurement	IPF
CD3 on resting CD4 regulatory T cell	GCST90001869	blood protein measurement	IPF
CD3 on resting CD4 regulatory T cell	GCST90001869	blood protein measurement	IPF
CD3 on resting CD4 regulatory T cell	GCST90001869	blood protein measurement	IPF
CD3 on CD4+ T cell	GCST90001867	blood protein measurement	IPF
CD3 on CD4+ T cell	GCST90001867	blood protein measurement	IPF
CD24 on transitional B cell	GCST90001774	blood protein measurement	IPF

FSC-A on CD4+ T cell	GCST90001973	blood protein measurement	IPF
CD20 on B cell	GCST90001743	blood protein measurement	IPF
CD20 on B cell	GCST90001743	blood protein measurement	IPF
CD20 on B cell	GCST90001743	blood protein measurement	IPF
CD20 on IgD+ B cell	GCST90001762	blood protein measurement	IPF
CD20 on IgD+ B cell	GCST90001762	blood protein measurement	IPF
CD20 on IgD+ B cell	GCST90001762	blood protein measurement	IPF
CD20 on IgD+ B cell	GCST90001762	blood protein measurement	IPF
CD25 on IgD- CD38- B cell	GCST90001787	blood protein measurement	IPF
CD25 on IgD- CD38- B cell	GCST90001787	blood protein measurement	IPF
CD25 on IgD- CD38- B cell	GCST90001787	blood protein measurement	IPF
Monocyte Absolute Count	GCST90001583	monocyte count	IPF
CD33 on CD66b++ myeloid cell	GCST90001951	blood protein measurement	IPF
CD33 on CD66b++ myeloid cell	GCST90001951	blood protein measurement	IPF
CD33 on CD66b++ myeloid cell	GCST90001951	blood protein measurement	IPF
CD33 on CD66b++ myeloid cell	GCST90001951	blood protein measurement	IPF
CD20 on IgD+ CD38- B cell	GCST90001748	blood protein measurement	IPF
CD20 on IgD+ CD38- B cell	GCST90001748	blood protein measurement	IPF
CD20 on IgD+ CD38- B cell	GCST90001748	blood protein measurement	IPF
CD20 on IgD+ CD38- B cell	GCST90001748	blood protein measurement	IPF
CD3 on HLA DR+ T cell	GCST90001847	blood protein measurement	IPF
CD3 on HLA DR+ T cell	GCST90001847	blood protein measurement	IPF
HLA DR on HLA DR+ Natural Killer	GCST90002117	blood protein measurement	IPF
HLA DR on HLA DR+ Natural Killer	GCST90002117	blood protein measurement	IPF
HLA DR on HLA DR+ Natural Killer	GCST90002117	blood protein measurement	IPF
HLA DR on HLA DR+ Natural Killer	GCST90002117	blood protein measurement	IPF
HLA DR on HLA DR+ Natural Killer	GCST90002117	blood protein measurement	IPF
CD8 on CD28+ CD45RA+ CD8+ T cell	GCST90002119	blood protein measurement	IPF
CD8 on CD28+ CD45RA+ CD8+ T cell	GCST90002119	blood protein measurement	IPF
CD8 on CD28+ CD45RA+ CD8+ T cell	GCST90002119	blood protein measurement	IPF
CD8 on CD28+ CD45RA+ CD8+ T cell	GCST90002119	blood protein measurement	IPF
CD8 on CD28+ CD45RA+ CD8+ T cell	GCST90002119	blood protein measurement	IPF
CD8 on CD28+ CD45RA+ CD8+ T cell	GCST90002119	blood protein measurement	IPF
CD8 on CD28+ CD45RA+ CD8+ T cell	GCST90002119	blood protein measurement	IPF
CD4+ CD8dim T cell %lymphocyte	GCST90001610	leukocyte count	IPF
CD4+ CD8dim T cell %lymphocyte	GCST90001610	leukocyte count	IPF
CD80 on granulocyte	GCST90002040	blood protein measurement	IPF
CD80 on granulocyte	GCST90002040	blood protein measurement	IPF
CD80 on granulocyte	GCST90002040	blood protein measurement	IPF
CD86 on granulocyte	GCST90001906	blood protein measurement	IPF
CD86 on granulocyte	GCST90001906	blood protein measurement	IPF
CD45RA- CD4+ T cell %T cell	GCST90001536	lymphocyte count	IPF
Terminally Differentiated CD4-CD8- T c	GCST90001573	lymphocyte count	IPF
Terminally Differentiated CD4+ T cell A	GCST90001545	lymphocyte count	IPF
Naive CD4+ T cell %CD4+ T cell	GCST90001541	lymphocyte count	IPF
CD25 on resting CD4 regulatory T cell	GCST90001937	blood protein measurement	IPF
CD28 on CD39+ secreting CD4 regulator	GCST90001888	blood protein measurement	IPF
CD28 on CD39+ secreting CD4 regulator	GCST90001888	blood protein measurement	IPF
B cell Absolute Count	GCST90001642	leukocyte count	IPF
IgD+ CD24+ B cell Absolute Count	GCST90001412	lymphocyte count	IPF
IgD+ CD38- B cell %lymphocyte	GCST90001431	lymphocyte count	IPF
Unswitched memory B cell %lymphocyte	GCST90001432	lymphocyte count	IPF
Unswitched memory B cell %lymphocyte	GCST90001432	lymphocyte count	IPF
T/B cell	GCST90001588	leukocyte count	IPF
T/B cell	GCST90001588	leukocyte count	IPF
CD24+ CD27+ B cell Absolute Count	GCST90001418	lymphocyte count	IPF
Memory B cell Absolute Count	GCST90001407	lymphocyte count	IPF
B cell %lymphocyte	GCST90001644	leukocyte count	IPF

B cell %lymphocyte	GCST90001644	leukocyte count	IPF
Unswitched memory B cell Absolute Cou	GCST90001398	lymphocyte count	IPF
CD3 on CD45RA+ CD4+ T cell	GCST90001858	blood protein measurement	IPF
CD3 on CD45RA+ CD4+ T cell	GCST90001858	blood protein measurement	IPF
CD3 on CD45RA+ CD4+ T cell	GCST90001858	blood protein measurement	IPF
CD3 on CD45RA+ CD4+ T cell	GCST90001858	blood protein measurement	IPF
CD3 on CD45RA+ CD4+ T cell	GCST90001858	blood protein measurement	IPF
CD28- CD8dim T cell Absolute Count	GCST90001663	lymphocyte count	IPF
CD28- CD8dim T cell Absolute Count	GCST90001663	lymphocyte count	IPF
CD8 on CD28+ CD45RA- CD8+ T cell	GCST90002118	blood protein measurement	IPF
Terminally Differentiated CD8+ T cell %	GCST90001558	lymphocyte count	IPF
Terminally Differentiated CD8+ T cell %	GCST90001558	lymphocyte count	IPF
Terminally Differentiated CD8+ T cell %	GCST90001558	lymphocyte count	IPF
BAFF-R on IgD- CD27- B cell	GCST90001711	blood protein measurement	IPF
BAFF-R on IgD- CD27- B cell	GCST90001711	blood protein measurement	IPF
BAFF-R on IgD- CD27- B cell	GCST90001711	blood protein measurement	IPF
BAFF-R on IgD- CD27- B cell	GCST90001711	blood protein measurement	IPF
BAFF-R on IgD- CD27- B cell	GCST90001711	blood protein measurement	IPF
BAFF-R on IgD- CD27- B cell	GCST90001711	blood protein measurement	IPF
BAFF-R on IgD- CD27- B cell	GCST90001711	blood protein measurement	IPF
BAFF-R on IgD- CD24- B cell	GCST90001710	blood protein measurement	IPF
BAFF-R on IgD- CD24- B cell	GCST90001710	blood protein measurement	IPF
BAFF-R on IgD- CD24- B cell	GCST90001710	blood protein measurement	IPF
BAFF-R on IgD- CD24- B cell	GCST90001710	blood protein measurement	IPF
BAFF-R on IgD- CD24- B cell	GCST90001710	blood protein measurement	IPF
BAFF-R on IgD- CD24- B cell	GCST90001710	blood protein measurement	IPF
BAFF-R on IgD- CD24- B cell	GCST90001710	blood protein measurement	IPF
CD127 on CD28+ CD4+ T cell	GCST90001924	blood protein measurement	IPF
CD127 on CD28+ CD4+ T cell	GCST90001924	blood protein measurement	IPF
CD127 on CD28+ CD4+ T cell	GCST90001924	blood protein measurement	IPF
CD4+ T cell %leukocyte	GCST90001606	leukocyte count	IPF
HLA DR+ CD4+ T cell Absolute Count	GCST90001624	leukocyte count	IPF
HLA DR+ CD4+ T cell Absolute Count	GCST90001624	leukocyte count	IPF
CD28 on CD45RA- CD4 not regulatory T	GCST90001891	blood protein measurement	IPF
CD28 on CD45RA- CD4 not regulatory T	GCST90001891	blood protein measurement	IPF
CD14+ CD16- monocyte Absolute Count	GCST90001582	monocyte count	IPF
CD14+ CD16- monocyte Absolute Count	GCST90001582	monocyte count	IPF
CD14+ CD16- monocyte Absolute Count	GCST90001582	monocyte count	IPF
Activated CD4 regulatory T cell %CD4 r	GCST90001487	lymphocyte count	IPF
Activated CD4 regulatory T cell %CD4 r	GCST90001487	lymphocyte count	IPF
Activated CD4 regulatory T cell %CD4 r	GCST90001487	lymphocyte count	IPF
IgD on unswitched memory B cell	GCST90001826	blood protein measurement	IPF
IgD on unswitched memory B cell	GCST90001826	blood protein measurement	IPF
IgD on unswitched memory B cell	GCST90001826	blood protein measurement	IPF
IgD on unswitched memory B cell	GCST90001826	blood protein measurement	IPF
CD28 on CD28+ CD4-CD8- T cell	GCST90001895	blood protein measurement	IPF
CD28 on CD28+ CD4-CD8- T cell	GCST90001895	blood protein measurement	IPF
CD28 on CD28+ CD4-CD8- T cell	GCST90001895	blood protein measurement	IPF
CD28- CD4-CD8- T cell %T cell	GCST90001694	lymphocyte count	IPF
CD28- CD4-CD8- T cell %T cell	GCST90001694	lymphocyte count	IPF
CD28- CD4-CD8- T cell %T cell	GCST90001694	lymphocyte count	IPF
CD4-CD8- T cell %leukocyte	GCST90001613	leukocyte count	IPF
CD4-CD8- T cell %leukocyte	GCST90001613	leukocyte count	IPF
CD4-CD8- T cell %leukocyte	GCST90001613	leukocyte count	IPF
CD8dim T cell %leukocyte	GCST90001612	leukocyte count	IPF
CD8dim T cell %leukocyte	GCST90001612	leukocyte count	IPF
CD4-CD8- T cell %T cell	GCST90001599	leukocyte count	IPF
CD4-CD8- T cell %T cell	GCST90001599	leukocyte count	IPF

Natural Killer T Absolute Count	GCST90001621	leukocyte count	IPF
Natural Killer T Absolute Count	GCST90001621	leukocyte count	IPF
Natural Killer T Absolute Count	GCST90001621	leukocyte count	IPF
Natural Killer T Absolute Count	GCST90001621	leukocyte count	IPF
Natural Killer T Absolute Count	GCST90001621	leukocyte count	IPF
CD8dim T cell Absolute Count	GCST90001596	leukocyte count	IPF
CD8dim T cell Absolute Count	GCST90001596	leukocyte count	IPF
CD8dim T cell Absolute Count	GCST90001596	leukocyte count	IPF
CD8dim T cell %T cell	GCST90001597	leukocyte count	IPF
CD8dim T cell %T cell	GCST90001597	leukocyte count	IPF
IgD+ CD38+ B cell Absolute Count	GCST90001392	lymphocyte count	IPF
IgD+ CD38+ B cell Absolute Count	GCST90001392	lymphocyte count	IPF
BAFF-R on IgD+ CD38- unswitched mer	GCST90001707	blood protein measurement	IPF
BAFF-R on IgD+ CD38- unswitched mer	GCST90001707	blood protein measurement	IPF
BAFF-R on IgD+ CD38- unswitched mer	GCST90001707	blood protein measurement	IPF
BAFF-R on IgD+ CD38- unswitched mer	GCST90001707	blood protein measurement	IPF
BAFF-R on IgD+ CD38- unswitched mer	GCST90001707	blood protein measurement	IPF
CD34 on Hematopoietic Stem Cell	GCST90001870	blood protein measurement	IPF
CD34 on Hematopoietic Stem Cell	GCST90001870	blood protein measurement	IPF
CD34 on Hematopoietic Stem Cell	GCST90001870	blood protein measurement	IPF
CD34 on Hematopoietic Stem Cell	GCST90001870	blood protein measurement	IPF
CD80 on CD62L+ myeloid Dendritic Cel	GCST90002036	blood protein measurement	IPF
CD80 on CD62L+ myeloid Dendritic Cel	GCST90002036	blood protein measurement	IPF
CD80 on CD62L+ myeloid Dendritic Cel	GCST90002036	blood protein measurement	IPF
CD80 on monocyte	GCST90002039	blood protein measurement	IPF
CD80 on monocyte	GCST90002039	blood protein measurement	IPF
CD80 on monocyte	GCST90002039	blood protein measurement	IPF
CD80 on monocyte	GCST90002039	blood protein measurement	IPF
CD80 on monocyte	GCST90002039	blood protein measurement	IPF
CD80 on myeloid Dendritic Cell	GCST90002035	blood protein measurement	IPF
CD80 on myeloid Dendritic Cell	GCST90002035	blood protein measurement	IPF
CD80 on myeloid Dendritic Cell	GCST90002035	blood protein measurement	IPF
CD80 on myeloid Dendritic Cell	GCST90002035	blood protein measurement	IPF
IgD+ CD38+ B cell %lymphocyte	GCST90001429	lymphocyte count	IPF
CD25++ CD8+ T cell Absolute Count	GCST90001681	lymphocyte count	IPF
CD25++ CD8+ T cell Absolute Count	GCST90001681	lymphocyte count	IPF
CD25++ CD8+ T cell Absolute Count	GCST90001681	lymphocyte count	IPF
FSC-A on CD14+ monocyte	GCST90001967	blood protein measurement	IPF
SSC-A on plasmacytoid Dendritic Cell	GCST90002072	blood protein measurement	IPF
SSC-A on plasmacytoid Dendritic Cell	GCST90002072	blood protein measurement	IPF
SSC-A on plasmacytoid Dendritic Cell	GCST90002072	blood protein measurement	IPF
SSC-A on plasmacytoid Dendritic Cell	GCST90002072	blood protein measurement	IPF
CD62L- myeloid Dendritic Cell Absolute	GCST90001468	leukocyte count	IPF
CD62L- myeloid Dendritic Cell Absolute	GCST90001468	leukocyte count	IPF
CD62L- myeloid Dendritic Cell Absolute	GCST90001468	leukocyte count	IPF
CD62L- myeloid Dendritic Cell Absolute	GCST90001468	leukocyte count	IPF
CCR2 on CD62L+ plasmacytoid Dendriti	GCST90002016	blood protein measurement	IPF
CCR2 on CD62L+ plasmacytoid Dendriti	GCST90002016	blood protein measurement	IPF
CCR2 on plasmacytoid Dendritic Cell	GCST90002015	blood protein measurement	IPF
CCR2 on plasmacytoid Dendritic Cell	GCST90002015	blood protein measurement	IPF
CD14- CD16- Absolute Count	GCST90001581	monocyte count	IPF
CD14- CD16- Absolute Count	GCST90001581	monocyte count	IPF
CX3CR1 on CD14- CD16-	GCST90001994	blood protein measurement	IPF
HLA DR on CD14+ CD16+ monocyte	GCST90002007	blood protein measurement	IPF
HLA DR on CD14+ CD16+ monocyte	GCST90002007	blood protein measurement	IPF
FSC-A on Natural Killer T	GCST90001976	blood protein measurement	IPF
FSC-A on Natural Killer T	GCST90001976	blood protein measurement	IPF
CD25 on B cell	GCST90001775	blood protein measurement	IPF

CD25 on B cell	GCST90001775	blood protein measurement	IPF
CD25 on B cell	GCST90001775	blood protein measurement	IPF
CD25 on IgD+ CD38dim B cell	GCST90001784	blood protein measurement	IPF
CD25 on IgD+ CD38dim B cell	GCST90001784	blood protein measurement	IPF
CD25 on IgD+ CD38dim B cell	GCST90001784	blood protein measurement	IPF
CD25 on IgD+ B cell	GCST90001794	blood protein measurement	IPF
CD25 on IgD+ B cell	GCST90001794	blood protein measurement	IPF
CD25 on naive-mature B cell	GCST90001791	blood protein measurement	IPF
CD25 on naive-mature B cell	GCST90001791	blood protein measurement	IPF
CD25 on naive-mature B cell	GCST90001791	blood protein measurement	IPF
CD25 on naive-mature B cell	GCST90001791	blood protein measurement	IPF
CD25 on IgD+ CD24- B cell	GCST90001779	blood protein measurement	IPF
CD25 on IgD+ CD24- B cell	GCST90001779	blood protein measurement	IPF
CD25 on IgD+ CD24- B cell	GCST90001779	blood protein measurement	IPF
CD25 on IgD+ CD24- B cell	GCST90001779	blood protein measurement	IPF
CD16 on CD14+ CD16+ monocyte	GCST90002005	blood protein measurement	IPF
CD16 on CD14+ CD16+ monocyte	GCST90002005	blood protein measurement	IPF
CD16 on CD14+ CD16+ monocyte	GCST90002005	blood protein measurement	IPF
CD4 on CD39+ resting CD4 regulatory T	GCST90002065	blood protein measurement	IPF
CD127 on granulocyte	GCST90001926	blood protein measurement	IPF
CD127 on granulocyte	GCST90001926	blood protein measurement	IPF
CD127 on granulocyte	GCST90001926	blood protein measurement	IPF
SSC-A on CD4+ T cell	GCST90002081	blood protein measurement	IPF
CD62L- myeloid Dendritic Cell %Dendri	GCST90001469	leukocyte count	IPF
CD62L- myeloid Dendritic Cell %Dendri	GCST90001469	leukocyte count	IPF
CD62L- myeloid Dendritic Cell %Dendri	GCST90001469	leukocyte count	IPF
CD62L- myeloid Dendritic Cell %Dendri	GCST90001469	leukocyte count	IPF
BAFF-R on IgD- CD38+ B cell	GCST90001713	blood protein measurement	IPF
BAFF-R on IgD- CD38+ B cell	GCST90001713	blood protein measurement	IPF
BAFF-R on IgD- CD38+ B cell	GCST90001713	blood protein measurement	IPF
CD20 on IgD+ CD24+ B cell	GCST90001746	blood protein measurement	IPF
CD20 on IgD+ CD24+ B cell	GCST90001746	blood protein measurement	IPF
CD20 on memory B cell	GCST90001758	blood protein measurement	IPF
CD20 on memory B cell	GCST90001758	blood protein measurement	IPF
CD20 on memory B cell	GCST90001758	blood protein measurement	IPF
CD20 on memory B cell	GCST90001758	blood protein measurement	IPF
CD4 regulatory T cell %CD4+ T cell	GCST90001478	lymphocyte count	IPF
CD4 regulatory T cell %CD4+ T cell	GCST90001478	lymphocyte count	IPF
CD4 regulatory T cell %T cell	GCST90001479	lymphocyte count	IPF
CCR7 on naive CD4+ T cell	GCST90001907	blood protein measurement	IPF
CCR7 on naive CD4+ T cell	GCST90001907	blood protein measurement	IPF
CCR7 on naive CD4+ T cell	GCST90001907	blood protein measurement	IPF
CCR7 on naive CD4+ T cell	GCST90001907	blood protein measurement	IPF
CCR7 on naive CD8+ T cell	GCST90001908	blood protein measurement	IPF
CCR7 on naive CD8+ T cell	GCST90001908	blood protein measurement	IPF
CD127 on CD4+ T cell	GCST90001931	blood protein measurement	IPF
CD33- HLA DR+ Absolute Count	GCST90001523	myeloid white cell count	IPF
Granulocytic Myeloid-Derived Suppresso	GCST90001524	myeloid white cell count	IPF
Granulocytic Myeloid-Derived Suppresso	GCST90001524	myeloid white cell count	IPF
CD8 on Effector Memory CD8+ T cell	GCST90002056	blood protein measurement	IPF
CD8 on Effector Memory CD8+ T cell	GCST90002056	blood protein measurement	IPF
CD8 on Effector Memory CD8+ T cell	GCST90002056	blood protein measurement	IPF
CD3 on CD39+ resting CD4 regulatory T	GCST90001852	blood protein measurement	IPF
CD3 on HLA DR+ CD4+ T cell	GCST90001849	blood protein measurement	IPF
CD3 on Central Memory CD8+ T cell	GCST90001846	blood protein measurement	IPF
CD3 on Central Memory CD8+ T cell	GCST90001846	blood protein measurement	IPF
CD3 on Central Memory CD8+ T cell	GCST90001846	blood protein measurement	IPF
CD3 on CD28+ CD45RA- CD8+ T cell	GCST90001863	blood protein measurement	IPF

CD3 on CD28+ CD45RA- CD8+ T cell	GCST90001863	blood protein measurement	IPF
CD3 on CD28+ CD45RA- CD8+ T cell	GCST90001863	blood protein measurement	IPF
CD3 on CD39+ CD8+ T cell	GCST90001866	blood protein measurement	IPF
CD3 on Terminally Differentiated CD4+	GCST90001844	blood protein measurement	IPF
CD3 on CD8+ T cell	GCST90001859	blood protein measurement	IPF
CD3 on CD8+ T cell	GCST90001859	blood protein measurement	IPF
CD45 on CD33- HLA DR+	GCST90002046	blood protein measurement	IPF
Resting CD4 regulatory T cell Absolute (	GCST90001480	lymphocyte count	IPF
Resting CD4 regulatory T cell Absolute (	GCST90001480	lymphocyte count	IPF
Resting CD4 regulatory T cell Absolute (	GCST90001480	lymphocyte count	IPF
Effector Memory CD8+ T cell %T cell	GCST90001556	lymphocyte count	IPF
Effector Memory CD8+ T cell %T cell	GCST90001556	lymphocyte count	IPF
Effector Memory CD8+ T cell Absolute (	GCST90001554	lymphocyte count	IPF
Effector Memory CD8+ T cell Absolute (	GCST90001554	lymphocyte count	IPF
CD25++ CD45RA- CD4 not regulatory T	GCST90001511	lymphocyte count	IPF
CD25++ CD45RA- CD4 not regulatory T	GCST90001511	lymphocyte count	IPF
CD28+ CD45RA- CD8+ T cell %T cell	GCST90001691	lymphocyte count	IPF
Effector Memory CD4+ T cell %CD4+ T	GCST90001543	lymphocyte count	IPF
CD45RA+ CD8+ T cell %T cell	GCST90001562	lymphocyte count	IPF
CD45RA+ CD8+ T cell %T cell	GCST90001562	lymphocyte count	IPF
CD45RA- CD4+ T cell Absolute Count	GCST90001534	lymphocyte count	IPF
Terminally Differentiated CD4+ T cell %	GCST90001546	lymphocyte count	IPF
Terminally Differentiated CD4+ T cell %	GCST90001547	lymphocyte count	IPF
Terminally Differentiated CD4+ T cell %	GCST90001547	lymphocyte count	IPF
CD25++ CD45RA- CD4 not regulatory T	GCST90001510	lymphocyte count	IPF
CD3 on Effector Memory CD8+ T cell	GCST90001839	blood protein measurement	IPF
CD3 on Effector Memory CD8+ T cell	GCST90001839	blood protein measurement	IPF
CD3 on Effector Memory CD8+ T cell	GCST90001839	blood protein measurement	IPF
CD3 on HLA DR+ CD8+ T cell	GCST90001850	blood protein measurement	IPF
CD3- lymphocyte Absolute Count	GCST90001639	leukocyte count	IPF
CD24 on IgD+ CD38+ B cell	GCST90001768	blood protein measurement	IPF
CD24 on IgD+ CD38+ B cell	GCST90001768	blood protein measurement	IPF
HLA DR on monocyte	GCST90002010	blood protein measurement	IPF
HLA DR on monocyte	GCST90002010	blood protein measurement	IPF
FSC-A on monocyte	GCST90001965	blood protein measurement	IPF
FSC-A on monocyte	GCST90001965	blood protein measurement	IPF
PDL-1 on CD14- CD16-	GCST90002000	blood protein measurement	IPF
CCR2 on CD14- CD16-	GCST90002003	blood protein measurement	IPF
CCR2 on CD14- CD16-	GCST90002003	blood protein measurement	IPF
CCR2 on CD14- CD16-	GCST90002003	blood protein measurement	IPF
CD33dim HLA DR+ CD11b+ Absolute (	GCST90001525	myeloid white cell count	IPF
CCR2 on monocyte	GCST90002017	blood protein measurement	IPF
CCR2 on monocyte	GCST90002017	blood protein measurement	IPF
CD14 on CD33dim HLA DR+ CD11b+	GCST90002021	blood protein measurement	IPF
CD27 on T cell	GCST90001799	blood protein measurement	IPF
CX3CR1 on CD14+ CD16+ monocyte	GCST90001996	blood protein measurement	IPF
CX3CR1 on CD14+ CD16+ monocyte	GCST90001996	blood protein measurement	IPF
CX3CR1 on monocyte	GCST90001995	blood protein measurement	IPF
CX3CR1 on monocyte	GCST90001995	blood protein measurement	IPF
CD11c on monocyte	GCST90002089	blood protein measurement	IPF
CD33 on CD33dim HLA DR+ CD11b-	GCST90001949	blood protein measurement	IPF
CD33 on CD33dim HLA DR+ CD11b-	GCST90001949	blood protein measurement	IPF
CD33 on CD33dim HLA DR+ CD11b-	GCST90001949	blood protein measurement	IPF
CD33 on CD33dim HLA DR+ CD11b-	GCST90001949	blood protein measurement	IPF
CD33 on CD33dim HLA DR+ CD11b-	GCST90001949	blood protein measurement	IPF
CD40 on CD14+ CD16+ monocyte	GCST90001981	blood protein measurement	IPF
CD40 on CD14+ CD16+ monocyte	GCST90001981	blood protein measurement	IPF
CD40 on CD14+ CD16+ monocyte	GCST90001981	blood protein measurement	IPF

CD40 on CD14+ CD16+ monocyte	GCST90001981	blood protein measurement	IPF
CD33 on CD33dim HLA DR+ CD11b+	GCST90001948	blood protein measurement	IPF
CD33 on CD33dim HLA DR+ CD11b+	GCST90001948	blood protein measurement	IPF
CD33 on CD33dim HLA DR+ CD11b+	GCST90001948	blood protein measurement	IPF
CD33 on CD33dim HLA DR+ CD11b+	GCST90001948	blood protein measurement	IPF
CD33 on CD33dim HLA DR+ CD11b+	GCST90001948	blood protein measurement	IPF
CD33 on CD33dim HLA DR+ CD11b+	GCST90001948	blood protein measurement	IPF
CX3CR1 on CD14- CD16+ monocyte	GCST90002012	blood protein measurement	IPF
CD11b on CD33+ HLA DR+ CD14dim	GCST90002097	blood protein measurement	IPF
CD11b on Monocytic Myeloid-Derived S	GCST90002094	blood protein measurement	IPF
PDL-1 on CD14+ CD16+ monocyte	GCST90001998	blood protein measurement	IPF
CD33dim HLA DR+ CD11b- %CD33dim	GCST90001528	myeloid white cell count	IPF
CD33dim HLA DR+ CD11b- %CD33dim	GCST90001528	myeloid white cell count	IPF
CD33dim HLA DR+ CD11b- %CD33dim	GCST90001528	myeloid white cell count	IPF
CD11c on granulocyte	GCST90002090	blood protein measurement	IPF
CD11c on granulocyte	GCST90002090	blood protein measurement	IPF
CD11b on CD14+ monocyte	GCST90002091	blood protein measurement	IPF
CX3CR1 on CD14+ CD16- monocyte	GCST90001997	blood protein measurement	IPF
CX3CR1 on CD14+ CD16- monocyte	GCST90001997	blood protein measurement	IPF
CD33dim HLA DR+ CD11b+ %CD33dim	GCST90001526	myeloid white cell count	IPF
CD33dim HLA DR+ CD11b+ %CD33dim	GCST90001526	myeloid white cell count	IPF
CD33dim HLA DR+ CD11b+ %CD33dim	GCST90001526	myeloid white cell count	IPF
CD25 on CD45RA- CD4 not regulatory T	GCST90001933	blood protein measurement	IPF
Natural Killer Absolute Count	GCST90001645	leukocyte count	IPF
Natural Killer Absolute Count	GCST90001645	leukocyte count	IPF
Natural Killer Absolute Count	GCST90001645	leukocyte count	IPF
CD45 on Natural Killer	GCST90001911	blood protein measurement	IPF
CD45 on Natural Killer	GCST90001911	blood protein measurement	IPF
CD33dim HLA DR+ CD11b- Absolute C	GCST90001527	myeloid white cell count	IPF
CD33dim HLA DR+ CD11b- Absolute C	GCST90001527	myeloid white cell count	IPF
CD25 on IgD- CD27- B cell	GCST90001786	blood protein measurement	IPF
CD25 on IgD- CD27- B cell	GCST90001786	blood protein measurement	IPF
CD27 on IgD- CD38+ B cell	GCST90001803	blood protein measurement	IPF
CD27 on IgD- CD38+ B cell	GCST90001803	blood protein measurement	IPF
CD27 on IgD+ CD24+ B cell	GCST90001800	blood protein measurement	IPF
CD27 on IgD+ CD24+ B cell	GCST90001800	blood protein measurement	IPF
CD27 on IgD+ CD24+ B cell	GCST90001800	blood protein measurement	IPF
CD27 on IgD+ CD24+ B cell	GCST90001800	blood protein measurement	IPF
CD27 on IgD+ CD24+ B cell	GCST90001800	blood protein measurement	IPF
CD27 on IgD+ CD24+ B cell	GCST90001800	blood protein measurement	IPF
CD20 on IgD- CD27- B cell	GCST90001754	blood protein measurement	IPF
CD20 on IgD- CD27- B cell	GCST90001754	blood protein measurement	IPF
CD20 on IgD- CD27- B cell	GCST90001754	blood protein measurement	IPF
CD20 on IgD- CD27- B cell	GCST90001754	blood protein measurement	IPF
HVEM on Central Memory CD8+ T cell	GCST90001882	blood protein measurement	IPF
HVEM on CD45RA- CD4+ T cell	GCST90001880	blood protein measurement	IPF
HVEM on CD4+ T cell	GCST90001875	blood protein measurement	IPF
HVEM on Effector Memory CD8+ T cell	GCST90001873	blood protein measurement	IPF
HVEM on T cell	GCST90001871	blood protein measurement	IPF
HVEM on Central Memory CD4+ T cell	GCST90001876	blood protein measurement	IPF
CD62L- Dendritic Cell Absolute Count	GCST90001462	leukocyte count	IPF
CD62L- Dendritic Cell Absolute Count	GCST90001462	leukocyte count	IPF
CD62L- Dendritic Cell Absolute Count	GCST90001462	leukocyte count	IPF
CD86+ myeloid Dendritic Cell Absolute	GCST90001464	leukocyte count	IPF
CD86+ myeloid Dendritic Cell Absolute	GCST90001464	leukocyte count	IPF
CD86+ myeloid Dendritic Cell Absolute	GCST90001464	leukocyte count	IPF
CD62L- CD86+ myeloid Dendritic Cell A	GCST90001472	leukocyte count	IPF
CD62L- CD86+ myeloid Dendritic Cell A	GCST90001472	leukocyte count	IPF

CD62L- CD86+ myeloid Dendritic Cell A	GCST90001472	leukocyte count	IPF
CD20 on naive-mature B cell	GCST90001759	blood protein measurement	IPF
CD20 on naive-mature B cell	GCST90001759	blood protein measurement	IPF
CD20 on naive-mature B cell	GCST90001759	blood protein measurement	IPF
CD20 on naive-mature B cell	GCST90001759	blood protein measurement	IPF
CD28 on CD28+ CD4+ T cell	GCST90001894	blood protein measurement	IPF
CD28 on CD28+ CD4+ T cell	GCST90001894	blood protein measurement	IPF
CD28 on CD4+ T cell	GCST90001898	blood protein measurement	IPF
CD28 on CD4+ T cell	GCST90001898	blood protein measurement	IPF
CD3 on Naive CD4+ T cell	GCST90001842	blood protein measurement	IPF
CD3 on Naive CD4+ T cell	GCST90001842	blood protein measurement	IPF
CD3 on Naive CD4+ T cell	GCST90001842	blood protein measurement	IPF
CD3 on Naive CD4+ T cell	GCST90001842	blood protein measurement	IPF
IgD on IgD+ CD38+ B cell	GCST90001824	blood protein measurement	IPF
IgD on IgD+ CD38+ B cell	GCST90001824	blood protein measurement	IPF
CD20 on IgD+ CD24- B cell	GCST90001747	blood protein measurement	IPF
CD20 on IgD+ CD24- B cell	GCST90001747	blood protein measurement	IPF
CD20 on IgD+ CD24- B cell	GCST90001747	blood protein measurement	IPF
CD20 on IgD+ CD38+ B cell	GCST90001751	blood protein measurement	IPF
CD20 on IgD+ CD38+ B cell	GCST90001751	blood protein measurement	IPF
CD20 on IgD+ CD38+ B cell	GCST90001751	blood protein measurement	IPF
CD20 on IgD+ CD38+ B cell	GCST90001751	blood protein measurement	IPF
CD45 on granulocyte	GCST90001913	blood protein measurement	IPF
CD45 on HLA DR+ CD4+ T cell	GCST90001920	blood protein measurement	IPF
CD45 on HLA DR+ CD4+ T cell	GCST90001920	blood protein measurement	IPF
Plasmacytoid Dendritic Cell Absolute Co	GCST90001460	leukocyte count	IPF
HLA DR+ CD8+ T cell Absolute Count	GCST90001627	leukocyte count	IPF
HLA DR+ CD8+ T cell Absolute Count	GCST90001627	leukocyte count	IPF
HLA DR+ CD8+ T cell Absolute Count	GCST90001627	leukocyte count	IPF
HLA DR+ T cell Absolute Count	GCST90001618	leukocyte count	IPF
HLA DR+ T cell Absolute Count	GCST90001618	leukocyte count	IPF
HLA DR+ T cell Absolute Count	GCST90001618	leukocyte count	IPF
HVEM on Effector Memory CD4+ T cell	GCST90001878	blood protein measurement	IPF
HVEM on CD8+ T cell	GCST90001881	blood protein measurement	IPF
HVEM on Terminally Differentiated CD4	GCST90001879	blood protein measurement	IPF
HVEM on naive CD8+ T cell	GCST90001872	blood protein measurement	IPF
HVEM on naive CD8+ T cell	GCST90001872	blood protein measurement	IPF
HVEM on naive CD4+ T cell	GCST90001877	blood protein measurement	IPF
CD24 on IgD+ CD38- unswitched memor	GCST90001767	blood protein measurement	IPF
CD24 on IgD+ CD38- unswitched memor	GCST90001767	blood protein measurement	IPF
CD24 on CD24+ CD27+ B cell	GCST90001764	blood protein measurement	IPF
CD24 on CD24+ CD27+ B cell	GCST90001764	blood protein measurement	IPF
CD24 on CD24+ CD27+ B cell	GCST90001764	blood protein measurement	IPF
CD24 on IgD+ CD38- B cell	GCST90001766	blood protein measurement	IPF
CD24 on IgD+ CD38- B cell	GCST90001766	blood protein measurement	IPF
CD24 on IgD- CD38dim B cell	GCST90001770	blood protein measurement	IPF
CD24 on IgD- CD38dim B cell	GCST90001770	blood protein measurement	IPF
CD24 on memory B cell	GCST90001771	blood protein measurement	IPF
CD24 on memory B cell	GCST90001771	blood protein measurement	IPF
CD24 on unswitched memory B cell	GCST90001772	blood protein measurement	IPF
CD24 on unswitched memory B cell	GCST90001772	blood protein measurement	IPF
CD24 on switched memory B cell	GCST90001773	blood protein measurement	IPF
CD24 on switched memory B cell	GCST90001773	blood protein measurement	IPF
CD24 on IgD+ CD24+ B cell	GCST90001765	blood protein measurement	IPF
CD24 on IgD+ CD24+ B cell	GCST90001765	blood protein measurement	IPF
CD24 on IgD- CD38- B cell	GCST90001769	blood protein measurement	IPF
CD24 on IgD- CD38- B cell	GCST90001769	blood protein measurement	IPF
SSC-A on granulocyte	GCST90002078	blood protein measurement	IPF

SSC-A on granulocyte	GCST90002078	blood protein measurement	IPF
IgD- CD27- B cell %B cell	GCST90001399	lymphocyte count	IPF
IgD- CD27- B cell %B cell	GCST90001399	lymphocyte count	IPF
IgD- CD27- B cell %B cell	GCST90001399	lymphocyte count	IPF
IgD- CD27- B cell Absolute Count	GCST90001401	lymphocyte count	IPF
IgD- CD27- B cell Absolute Count	GCST90001401	lymphocyte count	IPF
CD25 on IgD+ CD38- naive B cell	GCST90001781	blood protein measurement	IPF
CD25 on IgD+ CD38- naive B cell	GCST90001781	blood protein measurement	IPF
CD39 on monocyte	GCST90002034	blood protein measurement	IPF
CD39 on monocyte	GCST90002034	blood protein measurement	IPF
Immature Myeloid-Derived Suppressor C	GCST90001515	myeloid white cell count	IPF
Immature Myeloid-Derived Suppressor C	GCST90001515	myeloid white cell count	IPF
PDL-1 on CD14- CD16+ monocyte	GCST90001999	blood protein measurement	IPF
PDL-1 on CD14- CD16+ monocyte	GCST90001999	blood protein measurement	IPF
CD45 on B cell	GCST90001910	blood protein measurement	IPF
CD45 on B cell	GCST90001910	blood protein measurement	IPF
Naive CD4-CD8- T cell %T cell	GCST90001568	lymphocyte count	IPF
CD8 on HLA DR+ CD8+ T cell	GCST90002060	blood protein measurement	IPF
CD8 on HLA DR+ CD8+ T cell	GCST90002060	blood protein measurement	IPF
CD8 on Terminally Differentiated CD8+	GCST90002057	blood protein measurement	IPF
CD8 on Terminally Differentiated CD8+	GCST90002057	blood protein measurement	IPF
CD27 on IgD+ CD38- unswitched memoı	GCST90001801	blood protein measurement	IPF
CD27 on IgD+ CD38- unswitched memoı	GCST90001801	blood protein measurement	IPF
CD27 on IgD+ CD38- unswitched memoı	GCST90001801	blood protein measurement	IPF
CD27 on IgD+ CD38- unswitched memoı	GCST90001801	blood protein measurement	IPF
CD27 on IgD+ CD38- unswitched memoı	GCST90001801	blood protein measurement	IPF
CD14 on CD14+ CD16+ monocyte	GCST90001983	blood protein measurement	IPF
CD14 on CD14+ CD16+ monocyte	GCST90001983	blood protein measurement	IPF
IgD- CD38+ B cell %B cell	GCST90001410	lymphocyte count	IPF
FSC-A on myeloid Dendritic Cell	GCST90001963	blood protein measurement	IPF
FSC-A on myeloid Dendritic Cell	GCST90001963	blood protein measurement	IPF
FSC-A on myeloid Dendritic Cell	GCST90001963	blood protein measurement	IPF
CD8 on Central Memory CD8+ T cell	GCST90002054	blood protein measurement	IPF
CD8 on Central Memory CD8+ T cell	GCST90002054	blood protein measurement	IPF
CD4+CD8+ T cell %leukocyte	GCST90001608	leukocyte count	IPF
CD4+CD8+ T cell %T cell	GCST90001595	leukocyte count	IPF
IgD on IgD+ CD24- B cell	GCST90001821	blood protein measurement	IPF
IgD on IgD+ CD24- B cell	GCST90001821	blood protein measurement	IPF
IgD on IgD+ CD24- B cell	GCST90001821	blood protein measurement	IPF
IgD on IgD+ B cell	GCST90001827	blood protein measurement	IPF
IgD on IgD+ CD38dim B cell	GCST90001825	blood protein measurement	IPF
IgD on IgD+ CD38dim B cell	GCST90001825	blood protein measurement	IPF
IgD on IgD+ CD38dim B cell	GCST90001825	blood protein measurement	IPF
HLA DR on myeloid Dendritic Cell	GCST90002104	blood protein measurement	IPF
HLA DR on myeloid Dendritic Cell	GCST90002104	blood protein measurement	IPF
CD3 on Central Memory CD4+ T cell	GCST90001841	blood protein measurement	IPF
CD3 on Central Memory CD4+ T cell	GCST90001841	blood protein measurement	IPF
CD3 on Central Memory CD4+ T cell	GCST90001841	blood protein measurement	IPF
CD3 on Central Memory CD4+ T cell	GCST90001841	blood protein measurement	IPF
CD3 on CD39+ secreting CD4 regulatory	GCST90001856	blood protein measurement	IPF
CD3 on CD39+ secreting CD4 regulatory	GCST90001856	blood protein measurement	IPF
CD3 on CD39+ secreting CD4 regulatory	GCST90001856	blood protein measurement	IPF
CD3 on CD39+ CD4+ T cell	GCST90001860	blood protein measurement	IPF
CD3 on CD39+ CD4+ T cell	GCST90001860	blood protein measurement	IPF
CD3 on CD45RA- CD4+ T cell	GCST90001845	blood protein measurement	IPF
CD3 on CD45RA- CD4+ T cell	GCST90001845	blood protein measurement	IPF
CD3 on CD45RA- CD4+ T cell	GCST90001845	blood protein measurement	IPF
CD3 on Terminally Differentiated CD8+	GCST90001840	blood protein measurement	IPF

CD3 on CD28+ CD4+ T cell	GCST90001861	blood protein measurement	IPF
CD3 on CD28+ CD4+ T cell	GCST90001861	blood protein measurement	IPF
CD3 on CD28- CD8+ T cell	GCST90001865	blood protein measurement	IPF
CD3 on Effector Memory CD4+ T cell	GCST90001843	blood protein measurement	IPF
CD3 on CD28+ CD45RA+ CD8+ T cell	GCST90001864	blood protein measurement	IPF
CD3 on CD28+ CD45RA+ CD8+ T cell	GCST90001864	blood protein measurement	IPF
CD3 on CD28+ CD45RA+ CD8+ T cell	GCST90001864	blood protein measurement	IPF
HLA DR+ CD8+ T cell %T cell	GCST90001628	leukocyte count	IPF
HLA DR+ CD8+ T cell %T cell	GCST90001628	leukocyte count	IPF
HLA DR+ CD8+ T cell %T cell	GCST90001628	leukocyte count	IPF
HLA DR+ T cell%lymphocyte	GCST90001620	leukocyte count	IPF
HLA DR+ T cell%lymphocyte	GCST90001620	leukocyte count	IPF
HLA DR+ T cell%lymphocyte	GCST90001620	leukocyte count	IPF
HLA DR+ CD8+ T cell %lymphocyte	GCST90001629	leukocyte count	IPF
HLA DR+ CD8+ T cell %lymphocyte	GCST90001629	leukocyte count	IPF
HLA DR+ CD4+ T cell %lymphocyte	GCST90001626	leukocyte count	IPF
HLA DR+ CD4+ T cell %lymphocyte	GCST90001626	leukocyte count	IPF
CD28 on CD39+ CD8+ T cell	GCST90001897	blood protein measurement	IPF
CD28 on CD39+ CD8+ T cell	GCST90001897	blood protein measurement	IPF
CD28 on CD39+ activated CD4 regulator	GCST90001886	blood protein measurement	IPF
Naive CD4+ T cell Absolute Count	GCST90001540	lymphocyte count	IPF
CD127- CD8+ T cell Absolute Count	GCST90001684	lymphocyte count	IPF
CD8+ T cell Absolute Count	GCST90001592	leukocyte count	IPF
CD8+ T cell %leukocyte	GCST90001607	leukocyte count	IPF
CD28- CD8+ T cell Absolute Count	GCST90001687	lymphocyte count	IPF
CD28- CD8+ T cell Absolute Count	GCST90001687	lymphocyte count	IPF
Central Memory CD4+ T cell Absolute C	GCST90001537	lymphocyte count	IPF
CD4+ T cell Absolute Count	GCST90001590	leukocyte count	IPF
T cell Absolute Count	GCST90001603	leukocyte count	IPF
CD62L on CD62L+ plasmacytoid Dendri	GCST90001832	blood protein measurement	IPF
CD28 on secreting CD4 regulatory T cell	GCST90001887	blood protein measurement	IPF
CD28 on secreting CD4 regulatory T cell	GCST90001887	blood protein measurement	IPF
CD28 on secreting CD4 regulatory T cell	GCST90001887	blood protein measurement	IPF
CD28+ CD4-CD8- T cell %T cell	GCST90001655	lymphocyte count	IPF
CD4+ CD8dim T cell %leukocyte	GCST90001611	leukocyte count	IPF
CD4+ CD8dim T cell Absolute Count	GCST90001609	leukocyte count	IPF
CD20 on IgD- CD24- B cell	GCST90001753	blood protein measurement	IPF
IgD- CD27- B cell %lymphocyte	GCST90001433	lymphocyte count	IPF
IgD- CD27- B cell %lymphocyte	GCST90001433	lymphocyte count	IPF
CCR2 on CD62L+ myeloid Dendritic Cel	GCST90002014	blood protein measurement	IPF
CCR2 on CD62L+ myeloid Dendritic Cel	GCST90002014	blood protein measurement	IPF
CCR2 on myeloid Dendritic Cell	GCST90002013	blood protein measurement	IPF
CCR2 on myeloid Dendritic Cell	GCST90002013	blood protein measurement	IPF
HLA DR on Dendritic Cell	GCST90002106	blood protein measurement	IPF
HLA DR on Dendritic Cell	GCST90002106	blood protein measurement	IPF
HLA DR on Dendritic Cell	GCST90002106	blood protein measurement	IPF
HLA DR on Dendritic Cell	GCST90002106	blood protein measurement	IPF
CD25 on IgD+ CD38- B cell	GCST90001780	blood protein measurement	IPF
CD25 on IgD+ CD38- B cell	GCST90001780	blood protein measurement	IPF
CD25 on IgD+ CD38+ B cell	GCST90001783	blood protein measurement	IPF
CD25 on IgD+ CD38+ B cell	GCST90001783	blood protein measurement	IPF
CD25 on IgD- CD38dim B cell	GCST90001789	blood protein measurement	IPF
CD25 on IgD- CD38dim B cell	GCST90001789	blood protein measurement	IPF
CD25 on IgD- CD38dim B cell	GCST90001789	blood protein measurement	IPF
CD14+ CD16+ monocyte Absolute Coun	GCST90001580	monocyte count	IPF
CD14+ CD16+ monocyte Absolute Coun	GCST90001580	monocyte count	IPF
CD14+ CD16+ monocyte %monocyte	GCST90001585	monocyte count	IPF
CD14+ CD16+ monocyte %monocyte	GCST90001585	monocyte count	IPF

CD16 on CD14- CD16+ monocyte	GCST90001979	blood protein measurement	IPF
CD16 on CD14- CD16+ monocyte	GCST90001979	blood protein measurement	IPF
CD16 on CD14- CD16+ monocyte	GCST90001979	blood protein measurement	IPF
CD16 on CD14- CD16+ monocyte	GCST90001979	blood protein measurement	IPF
CD33 on CD33+ HLA DR+ CD14-	GCST90001957	blood protein measurement	IPF
CD33 on CD33+ HLA DR+ CD14-	GCST90001957	blood protein measurement	IPF
CD33 on CD33+ HLA DR+ CD14-	GCST90001957	blood protein measurement	IPF
CD33 on Monocytic Myeloid-Derived Su	GCST90001952	blood protein measurement	IPF
CD33 on Monocytic Myeloid-Derived Su	GCST90001952	blood protein measurement	IPF
CD33 on Monocytic Myeloid-Derived Su	GCST90001952	blood protein measurement	IPF
Basophil %CD33dim HLA DR- CD66b-	GCST90001533	myeloid white cell count	IPF
Basophil %CD33dim HLA DR- CD66b-	GCST90001533	myeloid white cell count	IPF
Basophil %CD33dim HLA DR- CD66b-	GCST90001533	myeloid white cell count	IPF
CD33 on CD14+ monocyte	GCST90001946	blood protein measurement	IPF
CD33 on CD14+ monocyte	GCST90001946	blood protein measurement	IPF
CD33 on CD14+ monocyte	GCST90001946	blood protein measurement	IPF
CD33 on CD14+ monocyte	GCST90001946	blood protein measurement	IPF
CD33 on CD14+ monocyte	GCST90001946	blood protein measurement	IPF
CD33+ HLA DR+ CD14dim Absolute Co	GCST90001520	myeloid white cell count	IPF
CD33+ HLA DR+ CD14dim Absolute Co	GCST90001520	myeloid white cell count	IPF
CD33 on CD33+ HLA DR+ CD14dim	GCST90001947	blood protein measurement	IPF
CD33 on CD33+ HLA DR+ CD14dim	GCST90001947	blood protein measurement	IPF
CD33 on CD33+ HLA DR+ CD14dim	GCST90001947	blood protein measurement	IPF
CD33 on CD33+ HLA DR+	GCST90001956	blood protein measurement	IPF
CD33 on CD33+ HLA DR+	GCST90001956	blood protein measurement	IPF
CD33 on CD33+ HLA DR+	GCST90001956	blood protein measurement	IPF
CD33+ HLA DR+ CD14- Absolute Coun	GCST90001518	myeloid white cell count	IPF
CD33 on CD33dim HLA DR-	GCST90001953	blood protein measurement	IPF
CD33 on CD33dim HLA DR-	GCST90001953	blood protein measurement	IPF
CD33 on CD33dim HLA DR-	GCST90001953	blood protein measurement	IPF
CD33 on CD33dim HLA DR-	GCST90001953	blood protein measurement	IPF
CD33+ HLA DR+ Absolute Count	GCST90001517	myeloid white cell count	IPF
CD62L on CD62L+ Dendritic Cell	GCST90001833	blood protein measurement	IPF
CD62L on CD62L+ Dendritic Cell	GCST90001833	blood protein measurement	IPF
CD19 on switched memory B cell	GCST90001740	blood protein measurement	IPF
CD19 on switched memory B cell	GCST90001740	blood protein measurement	IPF
HLA DR on CD14- CD16-	GCST90002009	blood protein measurement	IPF
HLA DR on CD14- CD16+ monocyte	GCST90001984	blood protein measurement	IPF
HLA DR on CD14- CD16+ monocyte	GCST90001984	blood protein measurement	IPF
HLA DR on CD14- CD16+ monocyte	GCST90001984	blood protein measurement	IPF
CD25 on switched memory B cell	GCST90001793	blood protein measurement	IPF
CD25 on switched memory B cell	GCST90001793	blood protein measurement	IPF
FSC-A on granulocyte	GCST90001966	blood protein measurement	IPF
CCR2 on granulocyte	GCST90002018	blood protein measurement	IPF
CD4 on HLA DR+ CD4+ T cell	GCST90001959	blood protein measurement	IPF
CD4 on HLA DR+ CD4+ T cell	GCST90001959	blood protein measurement	IPF
CD39 on granulocyte	GCST90002033	blood protein measurement	IPF
CD11c on CD62L+ myeloid Dendritic Ce	GCST90002088	blood protein measurement	IPF
CD127 on CD28+ CD45RA+ CD8+ T ce	GCST90001929	blood protein measurement	IPF
CD62L- monocyte %monocyte	GCST90001451	leukocyte count	IPF
CD62L- monocyte %monocyte	GCST90001451	leukocyte count	IPF
CD62L- monocyte %monocyte	GCST90001451	leukocyte count	IPF
CD24+ CD27+ B cell %B cell	GCST90001417	lymphocyte count	IPF
CD24+ CD27+ B cell %B cell	GCST90001417	lymphocyte count	IPF
CD27 on Plasma Blast-Plasma Cell	GCST90001807	blood protein measurement	IPF
CD3 on T cell	GCST90001851	blood protein measurement	IPF
CCR2 on CD14+ CD16- monocyte	GCST90002004	blood protein measurement	IPF
CCR2 on CD14+ CD16- monocyte	GCST90002004	blood protein measurement	IPF

CCR2 on monocyte	GCST90002008	blood protein measurement	IPF
CCR2 on monocyte	GCST90002008	blood protein measurement	IPF
Plasma Blast-Plasma Cell %lymphocyte	GCST90001435	lymphocyte count	IPF
CD3- lymphocyte %leukocyte	GCST90001641	leukocyte count	IPF
CD28+ CD4-CD8- T cell %CD4-CD8- T	GCST90001656	lymphocyte count	IPF
CD28- CD4-CD8- T cell %CD4-CD8- T	GCST90001653	lymphocyte count	IPF
CD28- CD8+ T cell %CD8+ T cell	GCST90001686	lymphocyte count	IPF
CD28- CD8+ T cell %CD8+ T cell	GCST90001686	lymphocyte count	IPF
CD28- CD8+ T cell %CD8+ T cell	GCST90001686	lymphocyte count	IPF
CD28- CD8dim T cell %CD8dim T cell	GCST90001662	lymphocyte count	IPF
Naive CD8+ T cell Absolute Count	GCST90001551	lymphocyte count	IPF
CD62L on monocyte	GCST90001834	blood protein measurement	IPF
CD38 on CD3- CD19-	GCST90001810	blood protein measurement	IPF
CD11c+ CD62L- monocyte Absolute Co	GCST90001452	leukocyte count	IPF
Dendritic Cell Absolute Count	GCST90001461	leukocyte count	IPF
Dendritic Cell Absolute Count	GCST90001461	leukocyte count	IPF
CD28 on CD28+ CD45RA- CD8+ T cell	GCST90001893	blood protein measurement	IPF
CD8 on CD28- CD8+ T cell	GCST90002120	blood protein measurement	IPF
CD8 on CD28- CD8+ T cell	GCST90002120	blood protein measurement	IPF
SSC-A on HLA DR+ Natural Killer	GCST90002077	blood protein measurement	IPF
SSC-A on HLA DR+ Natural Killer	GCST90002077	blood protein measurement	IPF
HLA DR+ Natural Killer %CD3- lymph	GCST90001650	leukocyte count	IPF
HLA DR+ Natural Killer %CD3- lymph	GCST90001650	leukocyte count	IPF
HLA DR+ Natural Killer %Natural Kille	GCST90001649	leukocyte count	IPF
HLA DR+ Natural Killer %Natural Kille	GCST90001649	leukocyte count	IPF
HLA DR+ Natural Killer %Natural Kille	GCST90001649	leukocyte count	IPF
HLA DR+ Natural Killer %Natural Kille	GCST90001649	leukocyte count	IPF
HLA DR+ Natural Killer %Natural Kille	GCST90001649	leukocyte count	IPF
Myeloid Dendritic Cell Absolute Count	GCST90001458	leukocyte count	IPF
Myeloid Dendritic Cell Absolute Count	GCST90001458	leukocyte count	IPF
CD14 on CD14+ CD16- monocyte	GCST90001986	blood protein measurement	IPF
CD11c on myeloid Dendritic Cell	GCST90002087	blood protein measurement	IPF
CD11c on myeloid Dendritic Cell	GCST90002087	blood protein measurement	IPF
CD11c on myeloid Dendritic Cell	GCST90002087	blood protein measurement	IPF
CD11c on myeloid Dendritic Cell	GCST90002087	blood protein measurement	IPF
Lymphocyte Absolute Count	GCST90001601	leukocyte count	IPF
CD45RA on Terminally Differentiated C	GCST90002101	blood protein measurement	IPF
HLA DR on CD33+ HLA DR+ CD14dim	GCST90002109	blood protein measurement	IPF
CD25++ CD4+ T cell %CD4+ T cell	GCST90001505	lymphocyte count	IPF
CD25++ CD8+ T cell %T cell	GCST90001679	lymphocyte count	IPF
CD25++ CD8+ T cell %T cell	GCST90001679	lymphocyte count	IPF
CD25++ CD4+ T cell %T cell	GCST90001506	lymphocyte count	IPF
CD25 on CD4+ T cell	GCST90001960	blood protein measurement	IPF
CD25++ CD4+ T cell Absolute Count	GCST90001504	lymphocyte count	IPF
CD25 on CD28+ CD4+ T cell	GCST90001962	blood protein measurement	IPF
CD28 on activated CD4 regulatory T cell	GCST90001902	blood protein measurement	IPF
CD28 on resting CD4 regulatory T cell	GCST90001900	blood protein measurement	IPF
CD28 on resting CD4 regulatory T cell	GCST90001900	blood protein measurement	IPF
CD28 on CD45RA+ CD4+ T cell	GCST90001890	blood protein measurement	IPF
CD28 on CD45RA+ CD4+ T cell	GCST90001890	blood protein measurement	IPF
HLA DR++ monocyte %monocyte	GCST90001475	leukocyte count	IPF
HLA DR++ monocyte %monocyte	GCST90001475	leukocyte count	IPF
CD123 on plasmacytoid Dendritic Cell	GCST90001944	blood protein measurement	IPF
CD123 on plasmacytoid Dendritic Cell	GCST90001944	blood protein measurement	IPF
CD80 on plasmacytoid Dendritic Cell	GCST90002037	blood protein measurement	IPF
CD80 on plasmacytoid Dendritic Cell	GCST90002037	blood protein measurement	IPF
CD123 on CD62L+ plasmacytoid Dendri	GCST90001945	blood protein measurement	IPF
CD123 on CD62L+ plasmacytoid Dendri	GCST90001945	blood protein measurement	IPF

CD80 on CD62L+ plasmacytoid Dendriti	GCST90002038	blood protein measurement	IPF
CD80 on CD62L+ plasmacytoid Dendriti	GCST90002038	blood protein measurement	IPF
CD25 on transitional B cell	GCST90001795	blood protein measurement	IPF
CD14 on Monocytic Myeloid-Derived Su	GCST90002019	blood protein measurement	IPF
CD62L- plasmacytoid Dendritic Cell %D	GCST90001471	leukocyte count	IPF
CD62L- plasmacytoid Dendritic Cell Abs	GCST90001470	leukocyte count	IPF
CD62L- Dendritic Cell %Dendritic Cell	GCST90001463	leukocyte count	IPF
CD62L- Dendritic Cell %Dendritic Cell	GCST90001463	leukocyte count	IPF
CD62L- Dendritic Cell %Dendritic Cell	GCST90001463	leukocyte count	IPF
CD25 on CD39+ activated CD4 regulator	GCST90001940	blood protein measurement	IPF
CD25 on CD39+ CD4 regulatory T cell	GCST90001935	blood protein measurement	IPF
CD25 on CD39+ resting CD4 regulatory	GCST90001938	blood protein measurement	IPF
Terminally Differentiated CD8+ T cell %	GCST90001559	lymphocyte count	IPF
Terminally Differentiated CD8+ T cell A	GCST90001557	lymphocyte count	IPF
CD28- CD4-CD8- T cell Absolute Count	GCST90001654	lymphocyte count	IPF
CD28- CD4-CD8- T cell Absolute Count	GCST90001654	lymphocyte count	IPF
Central Memory CD4+ T cell %CD4+ T	GCST90001538	lymphocyte count	IPF
Activated & resting CD4 regulatory T cel	GCST90001498	lymphocyte count	IPF
CD4-CD8- T cell Absolute Count	GCST90001598	leukocyte count	IPF
CD4-CD8- T cell Absolute Count	GCST90001598	leukocyte count	IPF
CD11c+ HLA DR++ monocyte %monocy	GCST90001457	leukocyte count	IPF
CD86 on myeloid Dendritic Cell	GCST90001903	blood protein measurement	IPF
Myeloid Dendritic Cell %Dendritic Cell	GCST90001459	leukocyte count	IPF
CD62L- CD86+ myeloid Dendritic Cell %	GCST90001473	leukocyte count	IPF
CD62L- CD86+ myeloid Dendritic Cell %	GCST90001473	leukocyte count	IPF
CD62L- CD86+ myeloid Dendritic Cell %	GCST90001473	leukocyte count	IPF
Plasmacytoid Dendritic Cell %Dendritic (	GCST90001474	leukocyte count	IPF
Plasmacytoid Dendritic Cell %Dendritic (	GCST90001474	leukocyte count	IPF
HLA DR+ Natural Killer Absolute Count	GCST90001648	leukocyte count	IPF
HLA DR+ Natural Killer Absolute Count	GCST90001648	leukocyte count	IPF
CD19 on IgD- CD38- B cell	GCST90001733	blood protein measurement	IPF
CD33 on Granulocytic Myeloid-Derived	GCST90001950	blood protein measurement	IPF
CD33 on Granulocytic Myeloid-Derived	GCST90001950	blood protein measurement	IPF
CD33 on Granulocytic Myeloid-Derived	GCST90001950	blood protein measurement	IPF
CD33+ HLA DR+ CD14- %CD33+ HLA	GCST90001519	myeloid white cell count	IPF
CD45 on CD33+ HLA DR+ CD14-	GCST90002042	blood protein measurement	IPF
CD25++ CD8+ T cell %CD8+ T cell	GCST90001680	lymphocyte count	IPF
CD4 on monocyte	GCST90001958	blood protein measurement	IPF
BAFF-R on CD20- CD38- B cell	GCST90001701	blood protein measurement	IPF
CD4+/CD8+ T cell	GCST90001589	leukocyte count	IPF
CD8+ T cell %T cell	GCST90001593	leukocyte count	IPF
CD45RA on CD39+ resting CD4 regulato	GCST90002103	blood protein measurement	IPF
Hematopoietic Stem Cell Absolute Count	GCST90001514	myeloid white cell count	IPF
CD19 on naive-mature B cell	GCST90001737	blood protein measurement	IPF
CD19 on naive-mature B cell	GCST90001737	blood protein measurement	IPF
CD45RA+ CD28- CD8+ T cell %CD8+ T	GCST90001699	lymphocyte count	IPF
IgD+ CD24- B cell %B cell	GCST90001415	lymphocyte count	IPF
CD11b on CD33dim HLA DR-	GCST90002095	blood protein measurement	IPF
CD11b on basophil	GCST90002096	blood protein measurement	IPF
CD38 on IgD+ CD38+ B cell	GCST90001812	blood protein measurement	IPF
CD38 on IgD+ CD38+ B cell	GCST90001812	blood protein measurement	IPF
CD38 on IgD+ B cell	GCST90001818	blood protein measurement	IPF
CD8+ Natural Killer T %T cell	GCST90001631	leukocyte count	IPF
Lymphocyte %leukocyte	GCST90001602	leukocyte count	IPF
Granulocyte %leukocyte	GCST90001652	leukocyte count	IPF
FSC-A on CD8+ T cell	GCST90001974	blood protein measurement	IPF
PDL-1 on CD14+ CD16- monocyte	GCST90001993	blood protein measurement	IPF
CD127 on CD8+ T cell	GCST90001927	blood protein measurement	IPF

CD8+ Natural Killer T Absolute Count	GCST90001630	leukocyte count	IPF
CD8+ Natural Killer T %lymphocyte	GCST90001632	leukocyte count	IPF
CD16-CD56 on HLA DR+ Natural Killer	GCST90001885	blood protein measurement	IPF
CD20 on transitional B cell	GCST90001763	blood protein measurement	IPF
HLA DR on CD33- HLA DR+	GCST90002112	blood protein measurement	IPF
SSC-A on lymphocyte	GCST90002079	blood protein measurement	IPF
CD33- HLA DR- Absolute Count	GCST90001522	myeloid white cell count	IPF

SNP	Chr	Position	EA.exposure	NEA.exposure	EA.outcome	NEA.outcome
rs1006368	10	126346603	T	C	T	C
rs10919543	1	161508617	G	A	G	A
rs9912354	17	33805730	T	C	T	C
rs1006368	10	126346603	T	C	T	C
rs1007337	22	39781585	T	C	T	C
rs2066399	6	15880540	A	G	A	G
rs34486765	19	49915911	A	G	A	G
rs4939384	11	60273642	G	A	G	A
rs72836542	6	14091682	T	C	T	C
rs7404890	16	11007193	A	G	A	G
rs10824158	10	53915868	T	G	T	G
rs111747460	3	74016318	A	G	A	G
rs114230155	1	102796337	A	G	A	G
rs114581990	2	235962017	G	A	G	A
rs116399750	4	176754667	A	G	A	G
rs116581718	5	29413612	C	T	C	T
rs116915023	18	62442252	T	C	T	C
rs116916598	11	121746998	G	A	G	A
rs117173207	11	24544063	A	G	A	G
rs117478414	8	56081028	T	C	T	C
rs117692894	7	39587008	T	G	T	G
rs117867588	8	15699657	A	G	A	G
rs12279408	11	84269972	T	C	T	C
rs12501785	4	180565400	A	G	A	G
rs137942933	20	33104122	T	G	T	G
rs138327786	6	1730982	A	G	A	G
rs138937927	16	51095561	T	C	T	C
rs143395500	10	69536834	T	C	T	C
rs145604620	6	11491250	G	A	G	A
rs147093663	15	81422902	T	G	T	G
rs149208831	6	10017676	C	A	C	A
rs16864501	3	152385976	A	G	A	G
rs17791728	20	17556587	C	T	C	T
rs185587352	3	117384981	A	G	A	G
rs62321187	4	120605817	T	C	T	C
rs72758450	15	69811049	C	T	C	T
rs72931475	2	159316835	G	A	G	A
rs75993384	4	55305871	C	T	C	T
rs76552842	11	83433494	A	G	A	G
rs77113660	12	83249816	G	A	G	A
rs78069205	10	9668124	G	A	G	A
rs78088167	4	130098084	G	A	G	A
rs78750136	14	26308944	C	T	C	T
rs79027575	2	146371302	C	T	C	T
rs79851411	8	13716390	A	G	A	G
rs80331554	15	59013643	G	A	G	A
rs10183338	2	111610816	T	G	T	G
rs116562707	6	31360602	G	A	G	A
rs143809235	2	167484735	G	T	G	T
rs1037633	3	23750521	G	T	G	T
rs200474	6	27767640	A	G	A	G
rs2018404	14	105695446	T	C	T	C
rs10422871	19	8789227	T	C	T	C
rs10765790	11	95852734	C	T	C	T
rs10426452	19	49907690	A	G	A	G
rs10484068	14	99653123	T	C	T	C

rs10824158	10	53915868	T	G	T	G
rs11060236	12	129773605	G	A	G	A
rs1106305	11	12065087	T	C	T	C
rs11112760	12	106241673	T	C	T	C
rs111648746	13	28485345	A	G	A	G
rs111867376	6	159962229	A	G	A	G
rs112121527	12	119037578	C	T	C	T
rs112137766	2	41540149	C	T	C	T
rs112517700	10	91013835	A	G	A	G
rs112529428	12	129340399	T	C	T	C
rs112785730	2	127831527	A	G	A	G
rs112880431	6	130091902	A	G	A	G
rs112973846	5	128365521	G	A	G	A
rs113369914	20	21420694	T	C	T	C
rs113698814	1	207678128	G	A	G	A
rs114230155	1	102796337	A	G	A	G
rs114425563	5	176911575	T	C	T	C
rs114507999	1	21892266	A	G	A	G
rs114619811	4	71875684	G	A	G	A
rs114685875	4	138349095	A	G	A	G
rs114791501	1	81668305	T	C	T	C
rs114871166	5	24165437	G	T	G	T
rs115040341	3	64543612	T	C	T	C
rs115135290	3	105859785	A	G	A	G
rs115212782	3	74527776	T	C	T	C
rs115221944	7	56724250	G	T	G	T
rs11538695	20	50004560	A	G	A	G
rs115517310	2	170436614	C	T	C	T
rs115660429	3	195531004	A	C	A	C
rs115677012	4	167555345	T	C	T	C
rs115755474	5	168914162	T	C	T	C
rs11576563	1	6118693	T	C	T	C
rs115821332	3	115341107	G	A	G	A
rs115945729	2	199934892	A	G	A	G
rs116399750	4	176754667	A	G	A	G
rs116431337	6	66416572	C	T	C	T
rs116498781	4	183314518	A	G	A	G
rs116510159	1	195636177	C	A	C	A
rs116581718	5	29413612	C	T	C	T
rs116589647	3	122070556	T	G	T	G
rs11661882	18	70504468	A	G	A	G
rs116719160	3	130201546	T	G	T	G
rs116916598	11	121746998	G	A	G	A
rs116921833	7	153895185	T	C	T	C
rs117003718	10	124406240	A	G	A	G
rs117177255	13	77081029	T	C	T	C
rs117185099	6	119514715	T	C	T	C
rs117270364	11	60694375	C	A	C	A
rs117293367	12	52437438	T	C	T	C
rs117433099	7	6934892	A	G	A	G
rs117448261	8	82400020	C	T	C	T
rs117469911	7	79791344	A	C	A	C
rs117555426	16	13128176	C	A	C	A
rs117702997	9	83435830	G	A	G	A
rs117741105	8	112234506	A	G	A	G
rs117785622	12	132283582	T	G	T	G
rs117801457	15	29421113	T	C	T	C
rs117808048	8	73079210	A	G	A	G

rs117822024	6	86346609	C	T	C	T
rs117840149	22	36506460	A	C	A	C
rs117922572	21	27289871	T	C	T	C
rs117922931	17	30028367	G	A	G	A
rs117983267	8	124174485	A	C	A	C
rs118076041	8	144562245	A	G	A	G
rs12130486	1	81455274	C	T	C	T
rs12279408	11	84269972	T	C	T	C
rs12332976	6	40616989	A	G	A	G
rs12378893	9	79057643	A	G	A	G
rs12501785	4	180565400	A	G	A	G
rs12750174	1	184765000	A	G	A	G
rs13010105	2	42107980	G	A	G	A
rs132751	22	36610967	T	C	T	C
rs137942933	20	33104122	T	G	T	G
rs137997289	7	115706613	C	T	C	T
rs138327786	6	1730982	A	G	A	G
rs138401052	3	166256176	A	G	A	G
rs138822361	1	5236853	A	G	A	G
rs138871895	3	162179186	C	T	C	T
rs138937927	16	51095561	T	C	T	C
rs138948609	10	132596864	A	G	A	G
rs138954494	4	32184694	C	T	C	T
rs139075636	12	102082782	G	A	G	A
rs140322068	10	1765793	A	G	A	G
rs140367496	4	131786765	C	T	C	T
rs140800343	19	50001170	T	C	T	C
rs140929582	2	169297185	T	C	T	C
rs141644068	1	220781210	A	G	A	G
rs142076427	16	5628078	A	G	A	G
rs142686680	12	29095512	C	T	C	T
rs142869493	11	87291175	T	C	T	C
rs142922198	5	145713781	C	A	C	A
rs143395500	10	69536834	T	C	T	C
rs143435133	12	129545747	A	G	A	G
rs143772909	10	101494118	T	C	T	C
rs143871291	19	1191667	G	A	G	A
rs143940797	1	52644883	A	G	A	G
rs144104650	17	5023557	A	G	A	G
rs144258173	8	2859400	G	A	G	A
rs144275943	14	34065832	G	T	G	T
rs144613377	5	81311763	C	T	C	T
rs144682449	12	53402638	A	G	A	G
rs144942838	1	111842326	T	C	T	C
rs145021762	10	22411454	A	G	A	G
rs145336406	6	78745175	T	C	T	C
rs145563511	9	106801353	G	A	G	A
rs145604620	6	11491250	G	A	G	A
rs145720280	13	43404037	C	T	C	T
rs145978106	18	7839215	A	C	A	C
rs146135726	5	154015729	T	C	T	C
rs146217030	2	41343706	C	T	C	T
rs146661200	12	100494123	T	C	T	C
rs147026949	4	100588891	T	C	T	C
rs147563624	10	5377215	T	C	T	C
rs147591823	1	47595103	G	A	G	A
rs147681963	7	37590076	T	C	T	C
rs147959386	8	124197307	A	G	A	G

rs147968748	10	54704473	G	A	G	A
rs148380088	9	28759554	T	C	T	C
rs148816938	5	71107850	G	A	G	A
rs148939501	5	12480500	G	A	G	A
rs148947489	2	10326418	T	C	T	C
rs149208831	6	10017676	C	A	C	A
rs149649979	9	10889621	T	C	T	C
rs149801155	6	162457335	G	A	G	A
rs149828559	6	84898123	T	C	T	C
rs149844272	4	87144762	A	G	A	G
rs150053624	17	80299376	T	C	T	C
rs150055480	14	55295865	A	G	A	G
rs150476409	13	113289664	A	G	A	G
rs1508064	2	72979172	T	C	T	C
rs151250363	2	240644273	T	C	T	C
rs16864501	3	152385976	A	G	A	G
rs17642570	5	148699825	C	T	C	T
rs17791728	20	17556587	C	T	C	T
rs17878699	3	3132754	A	G	A	G
rs17882143	17	41836082	T	C	T	C
rs183280627	8	30627573	A	G	A	G
rs183895386	2	22576943	T	C	T	C
rs184184520	14	45055780	T	G	T	G
rs184401135	7	83368658	T	C	T	C
rs185587352	3	117384981	A	G	A	G
rs186251573	7	13558580	C	A	C	A
rs187990734	3	144835290	G	A	G	A
rs188074116	12	19311236	A	G	A	G
rs188292706	2	162960872	T	C	T	C
rs191325818	10	120573449	C	A	C	A
rs192397067	6	81984275	T	C	T	C
rs2384564	10	36442592	G	A	G	A
rs2496150	6	13374132	G	A	G	A
rs34000508	8	25574426	T	C	T	C
rs34772471	1	112269677	T	C	T	C
rs35275693	12	88561479	G	A	G	A
rs35706199	7	131191874	T	C	T	C
rs35730839	7	109248384	T	C	T	C
rs35948774	2	118664032	A	G	A	G
rs459056	20	15614140	G	T	G	T
rs536434085	18	63832285	T	C	T	C
rs55787444	3	185766414	T	C	T	C
rs55856613	17	79094162	A	G	A	G
rs58243030	5	180495180	G	A	G	A
rs58817739	12	46009960	G	A	G	A
rs59745713	19	34075835	A	G	A	G
rs62247421	3	64390467	A	G	A	G
rs62321187	4	120605817	T	C	T	C
rs62337106	5	1957403	G	T	G	T
rs62424171	6	118769058	A	G	A	G
rs6517382	21	38156170	C	T	C	T
rs7130130	11	59336329	A	G	A	G
rs71466029	12	82065434	T	C	T	C
rs7254864	19	9246656	A	G	A	G
rs72704856	9	10774031	G	A	G	A
rs72723889	4	146773005	C	T	C	T
rs72768736	9	137149408	A	G	A	G
rs72931475	2	159316835	G	A	G	A

rs72996438	2	211172214	C	T	C	T
rs73034498	19	44937435	T	C	T	C
rs73116391	7	46417626	G	T	G	T
rs73206236	7	113679246	A	G	A	G
rs73780868	6	152137377	C	T	C	T
rs7426047	2	107184424	T	C	T	C
rs74322543	10	85916001	G	A	G	A
rs74336249	9	135625608	T	C	T	C
rs74540476	11	29902098	A	G	A	G
rs74565504	14	89207147	T	G	T	G
rs74842782	7	131413341	G	T	G	T
rs75157411	11	95180478	A	G	A	G
rs75361870	8	9202609	A	G	A	G
rs75510626	6	64401131	C	T	C	T
rs75550713	13	112088993	T	C	T	C
rs75551427	3	168644376	G	A	G	A
rs75561723	2	199542954	T	C	T	C
rs75592465	13	105831838	T	G	T	G
rs75600477	20	40197928	G	T	G	T
rs75660425	3	129232254	A	G	A	G
rs75760272	5	10850589	A	G	A	G
rs7597139	2	107560107	A	G	A	G
rs76385522	11	10620991	T	C	T	C
rs76543451	9	122201008	A	G	A	G
rs76552842	11	83433494	A	G	A	G
rs76568972	2	1592272	T	C	T	C
rs76636736	1	99542806	A	C	A	C
rs76679968	4	129754843	T	C	T	C
rs76736944	1	180881632	C	T	C	T
rs76808079	18	22735939	C	A	C	A
rs77009235	8	4499026	T	C	T	C
rs77156603	2	104590904	A	C	A	C
rs77370289	8	57917248	C	T	C	T
rs77390603	10	4688192	A	C	A	C
rs77434296	4	22140311	A	G	A	G
rs77485746	11	129187484	A	C	A	C
rs77538429	1	110742985	G	A	G	A
rs77577050	12	116077199	T	G	T	G
rs77587134	5	3635021	A	G	A	G
rs77689186	4	176614955	A	C	A	C
rs77725246	17	55639212	T	C	T	C
rs77814979	4	131041099	C	A	C	A
rs78085398	11	33121166	A	G	A	G
rs78100215	17	8298873	A	G	A	G
rs78275221	6	90900544	A	G	A	G
rs78313301	3	11075184	T	C	T	C
rs78470553	7	158334355	A	G	A	G
rs7850783	9	100968226	G	A	G	A
rs78622624	10	33359425	C	T	C	T
rs78656354	1	166905650	A	G	A	G
rs78703636	19	33953595	C	T	C	T
rs78750136	14	26308944	C	T	C	T
rs78773852	12	71181930	C	T	C	T
rs78881177	11	127881478	C	A	C	A
rs78888262	16	88701572	A	C	A	C
rs78922116	11	25534610	T	C	T	C
rs79027575	2	146371302	C	T	C	T
rs79418578	3	141886501	A	C	A	C

rs79548047	16	11614838	C	T	C	T
rs79725470	5	94705605	C	T	C	T
rs79741386	11	109660227	A	G	A	G
rs79757812	17	65896989	A	G	A	G
rs79851411	8	13716390	A	G	A	G
rs79888370	4	180201786	T	G	T	G
rs80018878	7	46985321	T	C	T	C
rs80305889	7	34681601	G	T	G	T
rs80331575	4	109566866	T	C	T	C
rs80334718	14	33998519	G	A	G	A
rs9471016	6	39196392	T	C	T	C
rs9479093	6	151992984	G	A	G	A
rs10501383	11	60164527	T	C	T	C
rs11751198	6	31753526	A	G	A	G
rs1569590	6	15898884	G	A	G	A
rs35039517	10	94522438	A	G	A	G
rs55809481	19	49872521	C	T	C	T
rs61731791	19	50940778	T	C	T	C
rs1050420	6	31239518	T	C	T	C
rs3020726	2	87016506	G	A	G	A
rs10512469	17	33770802	T	C	T	C
rs183053322	1	25076059	T	C	T	C
rs9916257	17	33797371	T	G	T	G
rs10512469	17	33770802	T	C	T	C
rs9916257	17	33797371	T	G	T	G
rs10512469	17	33770802	T	C	T	C
rs9916257	17	33797371	T	G	T	G
rs1051488	6	31322911	T	C	T	C
rs11579717	1	199999121	A	G	A	G
rs1064086	2	237034061	A	G	A	G
rs10774625	12	111910219	G	A	G	A
rs11615628	12	6903631	A	G	A	G
rs10786199	10	97069472	A	G	A	G
rs10882701	10	97741071	C	A	C	A
rs11188757	10	98205222	C	A	C	A
rs7088584	10	97607644	T	G	T	G
rs72809762	10	97294154	T	C	T	C
rs10786199	10	97069472	A	G	A	G
rs10882701	10	97741071	C	A	C	A
rs12268044	10	98794615	T	C	T	C
rs141018090	10	98169221	T	G	T	G
rs7094484	10	97490998	G	A	G	A
rs72809762	10	97294154	T	C	T	C
rs10786199	10	97069472	A	G	A	G
rs10882701	10	97741071	C	A	C	A
rs12268044	10	98794615	T	C	T	C
rs141018090	10	98169221	T	G	T	G
rs5014871	10	97522795	G	A	G	A
rs72809762	10	97294154	T	C	T	C
rs10786199	10	97069472	A	G	A	G
rs10882701	10	97741071	C	A	C	A
rs12268044	10	98794615	T	C	T	C
rs6584022	10	97509641	G	T	G	T
rs72809762	10	97294154	T	C	T	C
rs7918541	10	98168559	G	A	G	A
rs10786281	10	98116936	C	T	C	T
rs117458836	10	96773382	A	G	A	G
rs12778618	10	95633120	C	T	C	T

rs4293064	10	107757380	A	C	A	C
rs7086986	10	97507312	G	A	G	A
rs9264532	6	31234381	T	C	T	C
rs1081003	22	42525756	A	G	A	G
rs117705111	22	41957510	A	G	A	G
rs149806587	22	40774205	G	A	G	A
rs1894588	22	40629980	T	C	T	C
rs2018340	22	43390319	A	G	A	G
rs5757678	22	39843409	T	C	T	C
rs76146864	22	40246055	T	C	T	C
rs1081003	22	42525756	A	G	A	G
rs117705111	22	41957510	A	G	A	G
rs149806587	22	40774205	G	A	G	A
rs1801274	1	161479745	G	A	G	A
rs1894588	22	40629980	T	C	T	C
rs5757678	22	39843409	T	C	T	C
rs5759079	22	43393437	A	G	A	G
rs76146864	22	40246055	T	C	T	C
rs1081003	22	42525756	A	G	A	G
rs117705111	22	41957510	A	G	A	G
rs144886236	22	42326166	C	T	C	T
rs149806587	22	40774205	G	A	G	A
rs1801274	1	161479745	G	A	G	A
rs1894588	22	40629980	T	C	T	C
rs5757678	22	39843409	T	C	T	C
rs5759079	22	43393437	A	G	A	G
rs76146864	22	40246055	T	C	T	C
rs1081003	22	42525756	A	G	A	G
rs117705111	22	41957510	A	G	A	G
rs149806587	22	40774205	G	A	G	A
rs1894588	22	40629980	T	C	T	C
rs4657041	1	161478859	C	T	C	T
rs5757678	22	39843409	T	C	T	C
rs5759079	22	43393437	A	G	A	G
rs5996087	22	42321591	G	A	G	A
rs76146864	22	40246055	T	C	T	C
rs1081003	22	42525756	A	G	A	G
rs117705111	22	41957510	A	G	A	G
rs149806587	22	40774205	G	A	G	A
rs1894588	22	40629980	T	C	T	C
rs5757678	22	39843409	T	C	T	C
rs5759079	22	43393437	A	G	A	G
rs73165129	22	42315371	T	C	T	C
rs76146864	22	40246055	T	C	T	C
rs1081003	22	42525756	A	G	A	G
rs117705111	22	41957510	A	G	A	G
rs149806587	22	40774205	G	A	G	A
rs1801274	1	161479745	G	A	G	A
rs1894588	22	40629980	T	C	T	C
rs5757678	22	39843409	T	C	T	C
rs5759079	22	43393437	A	G	A	G
rs76146864	22	40246055	T	C	T	C
rs1081003	22	42525756	A	G	A	G
rs117705111	22	41957510	A	G	A	G
rs144886236	22	42326166	C	T	C	T
rs149806587	22	40774205	G	A	G	A
rs1801274	1	161479745	G	A	G	A
rs1894588	22	40629980	T	C	T	C

rs5757678	22	39843409	T	C	T	C
rs5759079	22	43393437	A	G	A	G
rs76146864	22	40246055	T	C	T	C
rs1081003	22	42525756	A	G	A	G
rs117705111	22	41957510	A	G	A	G
rs149806587	22	40774205	G	A	G	A
rs1801274	1	161479745	G	A	G	A
rs1894588	22	40629980	T	C	T	C
rs5757678	22	39843409	T	C	T	C
rs5759079	22	43393437	A	G	A	G
rs76146864	22	40246055	T	C	T	C
rs1081003	22	42525756	A	G	A	G
rs117705111	22	41957510	A	G	A	G
rs149806587	22	40774205	G	A	G	A
rs1894588	22	40629980	T	C	T	C
rs5757678	22	39843409	T	C	T	C
rs5759079	22	43393437	A	G	A	G
rs76146864	22	40246055	T	C	T	C
rs1084560	16	10824872	G	A	G	A
rs3087456	16	10970902	A	G	A	G
rs7032773	9	117645562	T	C	T	C
rs10882573	10	97009657	A	G	A	G
rs10882701	10	97741071	C	A	C	A
rs10883093	10	100183774	G	A	G	A
rs11188757	10	98205222	C	A	C	A
rs1410600	10	97070694	T	C	T	C
rs2805590	10	98812166	T	C	T	C
rs4918962	10	97475574	C	A	C	A
rs10882573	10	97009657	A	G	A	G
rs10882701	10	97741071	C	A	C	A
rs141018090	10	98169221	T	G	T	G
rs3814159	10	97515137	A	G	A	G
rs3824789	10	98800099	A	G	A	G
rs72809757	10	97285715	G	A	G	A
rs10882573	10	97009657	A	G	A	G
rs10882701	10	97741071	C	A	C	A
rs10883093	10	100183774	G	A	G	A
rs141018090	10	98169221	T	G	T	G
rs2805590	10	98812166	T	C	T	C
rs61854874	10	89173298	A	G	A	G
rs6584018	10	97480752	G	A	G	A
rs72809757	10	97285715	G	A	G	A
rs10882573	10	97009657	A	G	A	G
rs10882701	10	97741071	C	A	C	A
rs11188465	10	97506400	T	C	T	C
rs1410600	10	97070694	T	C	T	C
rs145557266	10	95850055	G	A	G	A
rs7918541	10	98168559	G	A	G	A
rs10882655	10	97474371	A	G	A	G
rs9263475	6	31050348	G	A	G	A
rs10882659	10	97510704	C	T	C	T
rs2949661	1	167424924	T	C	T	C
rs35055340	1	165314063	T	C	T	C
rs10882701	10	97741071	C	A	C	A
rs11188465	10	97506400	T	C	T	C
rs12778797	10	96998688	C	T	C	T
rs1410600	10	97070694	T	C	T	C
rs9663948	10	98238236	C	T	C	T

rs10882701	10	97741071	C	A	C	A
rs117458836	10	96773382	A	G	A	G
rs12778618	10	95633120	C	T	C	T
rs1539433	10	97519645	T	C	T	C
rs7426056	2	204612058	G	A	G	A
rs10882701	10	97741071	C	A	C	A
rs1410600	10	97070694	T	C	T	C
rs2901833	10	96998591	A	G	A	G
rs6584017	10	97480462	A	G	A	G
rs10882701	10	97741071	C	A	C	A
rs2901833	10	96998591	A	G	A	G
rs10882701	10	97741071	C	A	C	A
rs12778797	10	96998688	C	T	C	T
rs1410600	10	97070694	T	C	T	C
rs145557266	10	95850055	G	A	G	A
rs1539433	10	97519645	T	C	T	C
rs1923692	10	98173715	G	T	G	T
rs10882701	10	97741071	C	A	C	A
rs141018090	10	98169221	T	G	T	G
rs3824789	10	98800099	A	G	A	G
rs4336961	10	97497563	C	A	C	A
rs72809762	10	97294154	T	C	T	C
rs113063605	1	161246613	G	A	G	A
rs1801274	1	161479745	G	A	G	A
rs34658867	20	44977184	C	A	C	A
rs745307	20	44747086	A	G	A	G
rs10905716	10	6114010	T	C	T	C
rs10905719	10	6115171	A	G	A	G
rs11124653	2	39112166	A	C	A	C
rs11579717	1	199999121	A	G	A	G
rs61836370	1	188691469	G	A	G	A
rs10905719	10	6115171	A	G	A	G
rs10905719	10	6115171	A	G	A	G
rs11124653	2	39112166	A	C	A	C
rs11579717	1	199999121	A	G	A	G
rs61836370	1	188691469	G	A	G	A
rs10919544	1	161508763	C	T	C	T
rs12614599	2	232290640	A	G	A	G
rs10919544	1	161508763	C	T	C	T
rs111579151	11	111848073	T	C	T	C
rs11214436	11	112830782	T	G	T	G
rs117533070	11	112710664	T	C	T	C
rs148849191	11	113153223	T	C	T	C
rs17504675	11	110737507	A	G	A	G
rs77291736	11	112749800	T	C	T	C
rs11025036	11	19175633	A	G	A	G
rs2857229	12	6900616	G	A	G	A
rs11086984	20	44511627	T	C	T	C
rs162076	20	46785499	A	G	A	G
rs1801274	1	161479745	G	A	G	A
rs1883832	20	44746982	C	T	C	T
rs2250246	12	6553016	G	A	G	A
rs35574803	3	32533246	C	T	C	T
rs75918255	3	32945761	A	G	A	G
rs11065979	12	112059557	T	C	T	C
rs116275309	1	150863650	A	G	A	G
rs1801274	1	161479745	G	A	G	A
rs11074932	16	10968336	C	T	C	T

rs11074932	16	10968336	C	T	C	T
rs11074934	16	10979440	T	C	T	C
rs61742525	6	33072522	A	G	A	G
rs11074934	16	10979440	T	C	T	C
rs1632994	6	29926597	C	T	C	T
rs61742525	6	33072522	A	G	A	G
rs11080350	17	33774635	T	C	T	C
rs73402222	6	31388449	C	T	C	T
rs11086984	20	44511627	T	C	T	C
rs147714617	12	6605299	T	C	T	C
rs162076	20	46785499	A	G	A	G
rs1801274	1	161479745	G	A	G	A
rs1883832	20	44746982	C	T	C	T
rs25680	12	6554628	A	G	A	G
rs35574803	3	32533246	C	T	C	T
rs75918255	3	32945761	A	G	A	G
rs7621492	3	32302456	A	G	A	G
rs9989924	20	42019380	C	T	C	T
rs11086984	20	44511627	T	C	T	C
rs147714617	12	6605299	T	C	T	C
rs1883832	20	44746982	C	T	C	T
rs25680	12	6554628	A	G	A	G
rs35574803	3	32533246	C	T	C	T
rs56237381	3	32924804	A	G	A	G
rs72485816	20	45314435	C	T	C	T
rs75918255	3	32945761	A	G	A	G
rs11086984	20	44511627	T	C	T	C
rs144642376	3	36349691	G	A	G	A
rs147714617	12	6605299	T	C	T	C
rs162076	20	46785499	A	G	A	G
rs1801274	1	161479745	G	A	G	A
rs1883832	20	44746982	C	T	C	T
rs25680	12	6554628	A	G	A	G
rs35574803	3	32533246	C	T	C	T
rs75918255	3	32945761	A	G	A	G
rs11086984	20	44511627	T	C	T	C
rs144642376	3	36349691	G	A	G	A
rs147714617	12	6605299	T	C	T	C
rs1801274	1	161479745	G	A	G	A
rs25680	12	6554628	A	G	A	G
rs35574803	3	32533246	C	T	C	T
rs4810485	20	44747947	G	T	G	T
rs56237381	3	32924804	A	G	A	G
rs75918255	3	32945761	A	G	A	G
rs11086984	20	44511627	T	C	T	C
rs147714617	12	6605299	T	C	T	C
rs162076	20	46785499	A	G	A	G
rs1801274	1	161479745	G	A	G	A
rs1883832	20	44746982	C	T	C	T
rs25680	12	6554628	A	G	A	G
rs35574803	3	32533246	C	T	C	T
rs75918255	3	32945761	A	G	A	G
rs111231787	5	112408670	T	C	T	C
rs11124653	2	39112166	A	C	A	C
rs11579717	1	199999121	A	G	A	G
rs138915779	1	190079170	A	C	A	C
rs78689347	2	40773763	C	A	C	A
rs11124653	2	39112166	A	C	A	C

rs11579717	1	199999121	A	G	A	G
rs12712610	2	38897249	G	A	G	A
rs11124653	2	39112166	A	C	A	C
rs11579717	1	199999121	A	G	A	G
rs12712610	2	38897249	G	A	G	A
rs117519014	10	97403248	A	C	A	C
rs17190839	6	31138707	A	G	A	G
rs72847706	2	87055981	C	T	C	T
rs9357097	6	30285121	T	C	T	C
rs11136335	8	145003777	A	G	A	G
rs11554159	19	18285944	A	G	A	G
rs1800973	12	69744014	A	C	A	C
rs445	7	92408370	T	C	T	C
rs75994780	12	70949085	A	G	A	G
rs80216229	12	67839087	C	T	C	T
rs111388649	12	28246470	A	G	A	G
rs11233425	11	82732689	A	G	A	G
rs114135956	2	8930530	C	T	C	T
rs114216104	5	140131486	T	C	T	C
rs115954619	2	174896997	A	G	A	G
rs117428784	19	43422957	A	C	A	C
rs12656604	5	72679056	A	C	A	C
rs130071	6	31116210	A	G	A	G
rs143930177	10	70543706	A	G	A	G
rs147264423	15	78644995	G	A	G	A
rs149487182	8	131518018	T	C	T	C
rs149716976	16	75062625	G	A	G	A
rs149994650	3	18550864	A	C	A	C
rs190272290	2	9944052	A	G	A	G
rs55881385	1	34724192	T	G	T	G
rs55971473	1	117061778	T	C	T	C
rs58905133	2	38887964	G	A	G	A
rs61772921	1	80897562	C	T	C	T
rs62021713	15	50365389	T	C	T	C
rs62409210	4	25412773	A	G	A	G
rs74707627	15	86484743	C	T	C	T
rs76506268	4	187981624	G	A	G	A
rs76938777	7	31473901	A	G	A	G
rs111417549	11	36584481	T	C	T	C
rs111953852	14	84194540	G	A	G	A
rs111978771	1	21025910	C	T	C	T
rs112568609	15	27818525	T	C	T	C
rs113432575	21	30128360	T	C	T	C
rs114216104	5	140131486	T	C	T	C
rs114507999	1	21892266	A	G	A	G
rs114801643	6	66316172	A	C	A	C
rs115379408	6	18617129	A	G	A	G
rs115954619	2	174896997	A	G	A	G
rs116581718	5	29413612	C	T	C	T
rs116959045	8	53929771	G	T	G	T
rs117036387	20	3521072	G	A	G	A
rs11716645	3	29402908	G	A	G	A
rs117196114	9	83853182	A	G	A	G
rs117271894	8	112881674	A	G	A	G
rs117413806	11	16290338	C	T	C	T
rs117428784	19	43422957	A	C	A	C
rs117431224	11	44356396	G	A	G	A
rs117469911	7	79791344	A	C	A	C

rs117514047	21	26316207	C	A	C	A
rs117785622	12	132283582	T	G	T	G
rs117804407	16	5944116	G	A	G	A
rs12464465	2	176542288	A	G	A	G
rs130071	6	31116210	A	G	A	G
rs13044559	20	36073660	A	G	A	G
rs137978465	2	228411157	A	G	A	G
rs138010330	1	161526187	T	C	T	C
rs138799468	2	9441279	T	C	T	C
rs138962348	2	146534594	C	T	C	T
rs139457426	12	47679643	T	C	T	C
rs140134394	16	17341866	C	T	C	T
rs141023610	3	15790899	A	G	A	G
rs142470414	13	73498369	T	C	T	C
rs142522853	7	1084031	T	C	T	C
rs143825022	8	28561116	T	C	T	C
rs144571871	1	65133062	G	A	G	A
rs145336406	6	78745175	T	C	T	C
rs146164275	17	76005958	G	A	G	A
rs146940094	15	94946993	T	G	T	G
rs149716976	16	75062625	G	A	G	A
rs149994650	3	18550864	A	C	A	C
rs151120803	9	96711406	A	G	A	G
rs151145927	18	38375388	A	G	A	G
rs16864501	3	152385976	A	G	A	G
rs183382760	4	116470715	C	A	C	A
rs186595909	15	78554710	A	G	A	G
rs2615702	16	81462243	T	C	T	C
rs34592474	7	57279055	C	T	C	T
rs55830085	4	106862213	T	C	T	C
rs55881385	1	34724192	T	G	T	G
rs58905133	2	38887964	G	A	G	A
rs61772921	1	80897562	C	T	C	T
rs61864030	10	133431823	C	T	C	T
rs61988500	14	86640070	C	T	C	T
rs62021713	15	50365389	T	C	T	C
rs62369101	5	97959184	A	G	A	G
rs62409210	4	25412773	A	G	A	G
rs62513410	8	142607179	A	G	A	G
rs72728362	8	127054086	C	T	C	T
rs72815709	2	80415786	C	T	C	T
rs72929459	2	200181613	T	C	T	C
rs74603821	12	101714936	C	A	C	A
rs74902462	12	59066617	T	C	T	C
rs75346347	18	7862061	T	C	T	C
rs75828981	2	144233823	A	G	A	G
rs75884646	15	95776793	A	G	A	G
rs76506268	4	187981624	G	A	G	A
rs76595423	1	102745011	G	T	G	T
rs76732756	1	221000197	A	G	A	G
rs77152285	4	93935039	G	A	G	A
rs77164608	7	21673701	A	G	A	G
rs77243672	14	76846837	G	T	G	T
rs77937827	20	20799344	C	T	C	T
rs78656354	1	166905650	A	G	A	G
rs78939697	6	67374008	C	A	C	A
rs79407975	10	28109090	A	C	A	C
rs79870119	4	64142694	A	G	A	G

rs79888820	15	96888705	C	T	C	T
rs80012883	13	94341740	T	G	T	G
rs80347872	6	562203	A	G	A	G
rs111436326	20	42701487	T	C	T	C
rs139376787	1	161847528	A	G	A	G
rs148765873	1	161221713	C	T	C	T
rs1801274	1	161479745	G	A	G	A
rs34658867	20	44977184	C	A	C	A
rs6032211	20	44092301	A	G	A	G
rs6687275	1	161515638	G	T	G	T
rs745307	20	44747086	A	G	A	G
rs11158593	14	65859984	A	G	A	G
rs2844608	6	31247414	T	C	T	C
rs3184504	12	111884608	C	T	C	T
rs951818	12	6896055	A	C	A	C
rs111695695	19	51897760	A	C	A	C
rs56043070	1	247719769	A	G	A	G
rs58483275	19	51868443	T	C	T	C
rs7245846	19	51731176	A	G	A	G
rs7351079	19	51574038	G	A	G	A
rs75773078	19	51742145	A	G	A	G
rs111695695	19	51897760	A	C	A	C
rs58483275	19	51868443	T	C	T	C
rs7245846	19	51731176	A	G	A	G
rs7351079	19	51574038	G	A	G	A
rs75773078	19	51742145	A	G	A	G
rs111695695	19	51897760	A	C	A	C
rs273640	19	51725824	T	G	T	G
rs111780443	11	132210362	T	C	T	C
rs12921174	16	430304	C	T	C	T
rs2995089	1	167431193	A	G	A	G
rs4657649	1	167173140	C	T	C	T
rs4657706	1	167728447	T	C	T	C
rs61814886	1	167136405	A	G	A	G
rs78132817	1	165191695	G	T	G	T
rs11187166	10	94516008	T	C	T	C
rs2304938	11	60167744	T	C	T	C
rs55809481	19	49872521	C	T	C	T
rs6459417	6	15858996	T	C	T	C
rs9461557	6	29765925	T	C	T	C
rs11187166	10	94516008	T	C	T	C
rs2070770	11	60230531	T	C	T	C
rs55809481	19	49872521	C	T	C	T
rs61731791	19	50940778	T	C	T	C
rs909846	6	15897003	G	T	G	T
rs9469053	6	31755776	G	A	G	A
rs11187515	10	95327429	T	C	T	C
rs12778618	10	95633120	C	T	C	T
rs35025323	10	97089991	C	T	C	T
rs4075310	10	97574689	T	C	T	C
rs7079761	10	97842115	A	G	A	G
rs111902980	1	157954450	A	G	A	G
rs139249541	6	102294761	G	A	G	A
rs6710361	2	87047145	A	G	A	G
rs9896155	17	33794697	G	A	G	A
rs111902980	1	157954450	A	G	A	G
rs116877116	8	135490341	A	G	A	G
rs111983490	6	32205324	A	G	A	G

rs6755452	2	87035076	G	T	G	T
rs111983490	6	32205324	A	G	A	G
rs143305120	6	13991643	C	T	C	T
rs147210938	6	13892767	A	G	A	G
rs2286975	16	11114006	A	G	A	G
rs3869145	6	31851354	A	G	A	G
rs7192768	16	11004905	C	T	C	T
rs74405933	6	14095755	A	G	A	G
rs11214436	11	112830782	T	G	T	G
rs117147408	6	101980039	G	A	G	A
rs77738700	11	112751407	A	G	A	G
rs79251210	6	100468602	A	G	A	G
rs112258643	12	55672838	T	C	T	C
rs112571466	7	111704231	G	A	G	A
rs113447229	9	105346840	C	T	C	T
rs115077170	2	53872294	G	T	G	T
rs115218435	4	67836131	G	T	G	T
rs116562707	6	31360602	G	A	G	A
rs117475733	6	148775706	T	C	T	C
rs118016388	12	28662288	C	T	C	T
rs12755474	1	216352525	G	A	G	A
rs142208889	19	51829114	T	C	T	C
rs142530179	1	62672889	G	A	G	A
rs143498795	8	140342260	A	C	A	C
rs149023182	1	232754676	G	A	G	A
rs189801254	10	68336237	A	C	A	C
rs73127385	4	31396800	G	A	G	A
rs76325389	9	122676137	G	A	G	A
rs77935824	18	68968361	G	A	G	A
rs80194890	10	91332621	G	T	G	T
rs112405902	6	31809504	A	G	A	G
rs4447195	11	60249734	T	C	T	C
rs6459417	6	15858996	T	C	T	C
rs6509428	19	49853881	T	C	T	C
rs112417310	1	206855483	A	G	A	G
rs11643297	16	28973268	G	A	G	A
rs112417310	1	206855483	A	G	A	G
rs113812519	1	207276336	G	A	G	A
rs144743804	1	207018206	A	G	A	G
rs112421400	6	28205826	T	C	T	C
rs139084170	6	43351686	A	C	A	C
rs1794511	6	32671207	G	T	G	T
rs11249570	5	177033794	A	G	A	G
rs4987369	1	169662742	T	G	T	G
rs11579717	1	199999121	A	G	A	G
rs12712610	2	38897249	G	A	G	A
rs138915779	1	190079170	A	C	A	C
rs138960714	4	123422719	C	A	C	A
rs61839660	10	6094697	T	C	T	C
rs74830391	7	37427289	T	C	T	C
rs112581826	2	204650296	A	G	A	G
rs72928038	6	90976768	A	G	A	G
rs112637846	6	149114553	A	C	A	C
rs11579717	1	199999121	A	G	A	G
rs12712610	2	38897249	G	A	G	A
rs61038907	2	87061070	A	C	A	C
rs74577787	3	84799491	T	C	T	C
rs113116201	1	198830942	C	T	C	T

rs113116201	1	198830942	C	T	C	T
rs139529340	1	161563554	G	A	G	A
rs1800973	12	69744014	A	C	A	C
rs4910742	11	5306509	A	G	A	G
rs80297185	11	5488177	T	C	T	C
rs113398446	6	161871755	C	A	C	A
rs113435341	1	198151688	G	A	G	A
rs138915779	1	190079170	A	C	A	C
rs113435341	1	198151688	G	A	G	A
rs113500908	6	100774499	T	C	T	C
rs13210358	6	102272234	T	C	T	C
rs34546581	2	87021659	A	G	A	G
rs5996087	22	42321591	G	A	G	A
rs113812519	1	207276336	G	A	G	A
rs144743804	1	207018206	A	G	A	G
rs55712453	1	206902437	G	A	G	A
rs113812519	1	207276336	G	A	G	A
rs144743804	1	207018206	A	G	A	G
rs55712453	1	206902437	G	A	G	A
rs113812519	1	207276336	G	A	G	A
rs144743804	1	207018206	A	G	A	G
rs55712453	1	206902437	G	A	G	A
rs113812519	1	207276336	G	A	G	A
rs78760135	1	206909820	G	A	G	A
rs79856590	1	205241642	A	G	A	G
rs113812519	1	207276336	G	A	G	A
rs3934748	1	206879659	T	G	T	G
rs113812519	1	207276336	G	A	G	A
rs144743804	1	207018206	A	G	A	G
rs3934748	1	206879659	T	G	T	G
rs113812519	1	207276336	G	A	G	A
rs11643297	16	28973268	G	A	G	A
rs55712453	1	206902437	G	A	G	A
rs113865045	2	176969703	A	G	A	G
rs2455069	19	51728641	G	A	G	A
rs114135956	2	8930530	C	T	C	T
rs130071	6	31116210	A	G	A	G
rs140385926	15	69903187	G	A	G	A
rs1736913	6	29704400	C	T	C	T
rs450175	2	38400812	C	A	C	A
rs58905133	2	38887964	G	A	G	A
rs72815709	2	80415786	C	T	C	T
rs114590598	1	185852065	T	C	T	C
rs78744187	19	33754548	T	C	T	C
rs114590598	1	185852065	T	C	T	C
rs3865444	19	51727962	A	C	A	C
rs78744187	19	33754548	T	C	T	C
rs114672530	1	168306279	A	G	A	G
rs2949661	1	167424924	T	C	T	C
rs4657649	1	167173140	C	T	C	T
rs114672530	1	168306279	A	G	A	G
rs2949661	1	167424924	T	C	T	C
rs35055340	1	165314063	T	C	T	C
rs114672530	1	168306279	A	G	A	G
rs2949661	1	167424924	T	C	T	C
rs35055340	1	165314063	T	C	T	C
rs114672530	1	168306279	A	G	A	G
rs2949661	1	167424924	T	C	T	C

rs35055340	1	165314063	T	C	T	C
rs114968045	6	32666960	C	T	C	T
rs115062208	4	15956227	G	A	G	A
rs76112278	4	15915069	A	G	A	G
rs115062208	4	15956227	G	A	G	A
rs76112278	4	15915069	A	G	A	G
rs76354299	4	15037408	A	G	A	G
rs115062208	4	15956227	G	A	G	A
rs149852991	4	15044338	T	G	T	G
rs197429	1	112267500	T	C	T	C
rs572542	4	14029370	T	C	T	C
rs76112278	4	15915069	A	G	A	G
rs115062208	4	15956227	G	A	G	A
rs76112278	4	15915069	A	G	A	G
rs76354299	4	15037408	A	G	A	G
rs115062208	4	15956227	G	A	G	A
rs572542	4	14029370	T	C	T	C
rs76112278	4	15915069	A	G	A	G
rs76354299	4	15037408	A	G	A	G
rs115383270	1	161531340	A	G	A	G
rs74975395	1	166026114	T	C	T	C
rs115383270	1	161531340	A	G	A	G
rs3826007	15	80263217	T	C	T	C
rs115383270	1	161531340	A	G	A	G
rs115383270	1	161531340	A	G	A	G
rs77640671	16	58607804	T	C	T	C
rs115383270	1	161531340	A	G	A	G
rs74975395	1	166026114	T	C	T	C
rs115383270	1	161531340	A	G	A	G
rs11544989	8	145057868	G	A	G	A
rs115536607	6	101417409	T	C	T	C
rs117147408	6	101980039	G	A	G	A
rs77738700	11	112751407	A	G	A	G
rs79251210	6	100468602	A	G	A	G
rs9916257	17	33797371	T	G	T	G
rs11544989	8	145057868	G	A	G	A
rs115536607	6	101417409	T	C	T	C
rs117147408	6	101980039	G	A	G	A
rs77738700	11	112751407	A	G	A	G
rs79251210	6	100468602	A	G	A	G
rs11548656	16	81916912	G	A	G	A
rs41294937	10	6106621	C	T	C	T
rs115536607	6	101417409	T	C	T	C
rs117147408	6	101980039	G	A	G	A
rs77738700	11	112751407	A	G	A	G
rs79251210	6	100468602	A	G	A	G
rs9916629	17	33796260	C	T	C	T
rs115536607	6	101417409	T	C	T	C
rs117147408	6	101980039	G	A	G	A
rs77738700	11	112751407	A	G	A	G
rs7819099	8	144992862	G	A	G	A
rs79251210	6	100468602	A	G	A	G
rs115536607	6	101417409	T	C	T	C
rs117147408	6	101980039	G	A	G	A
rs77738700	11	112751407	A	G	A	G
rs79251210	6	100468602	A	G	A	G
rs9916257	17	33797371	T	G	T	G
rs115536607	6	101417409	T	C	T	C

rs117147408	6	101980039	G	A	G	A
rs7218011	17	33773022	G	A	G	A
rs77738700	11	112751407	A	G	A	G
rs7819099	8	144992862	G	A	G	A
rs79251210	6	100468602	A	G	A	G
rs9912354	17	33805730	T	C	T	C
rs115536607	6	101417409	T	C	T	C
rs117147408	6	101980039	G	A	G	A
rs77291736	11	112749800	T	C	T	C
rs79251210	6	100468602	A	G	A	G
rs9916629	17	33796260	C	T	C	T
rs115536607	6	101417409	T	C	T	C
rs117147408	6	101980039	G	A	G	A
rs77738700	11	112751407	A	G	A	G
rs79251210	6	100468602	A	G	A	G
rs11554159	19	18285944	A	G	A	G
rs1800973	12	69744014	A	C	A	C
rs445	7	92408370	T	C	T	C
rs75994780	12	70949085	A	G	A	G
rs115624142	1	161701708	A	C	A	C
rs71632979	1	161526397	G	A	G	A
rs71639910	1	161937774	A	G	A	G
rs1573735	16	423696	T	C	T	C
rs3129891	6	32415080	A	G	A	G
rs115752428	1	205560814	A	G	A	G
rs11579717	1	199999121	A	G	A	G
rs11818051	10	97972294	T	C	T	C
rs143132810	10	96890004	C	T	C	T
rs17111341	10	97881031	C	T	C	T
rs185474383	10	96311553	T	G	T	G
rs3740373	10	95256200	A	C	A	C
rs4918969	10	97562938	A	G	A	G
rs77520132	10	98160835	A	G	A	G
rs11579717	1	199999121	A	G	A	G
rs12712610	2	38897249	G	A	G	A
rs138915779	1	190079170	A	C	A	C
rs138960714	4	123422719	C	A	C	A
rs74830391	7	37427289	T	C	T	C
rs7893324	10	6111075	C	T	C	T
rs11579717	1	199999121	A	G	A	G
rs61839660	10	6094697	T	C	T	C
rs11579717	1	199999121	A	G	A	G
rs7559619	2	38892207	G	A	G	A
rs11579717	1	199999121	A	G	A	G
rs138915779	1	190079170	A	C	A	C
rs17607399	2	41884212	T	C	T	C
rs6751481	2	38897810	C	T	C	T
rs7530076	1	195825276	A	G	A	G
rs11579717	1	199999121	A	G	A	G
rs11818051	10	97972294	T	C	T	C
rs11890666	2	38745822	A	G	A	G
rs12712610	2	38897249	G	A	G	A
rs1410600	10	97070694	T	C	T	C
rs17111341	10	97881031	C	T	C	T
rs182702572	10	96658384	A	C	A	C
rs34645541	1	195875605	A	C	A	C
rs35909109	10	98014850	T	C	T	C
rs4918969	10	97562938	A	G	A	G

rs77520132	10	98160835	A	G	A	G
rs11579717	1	199999121	A	G	A	G
rs12712610	2	38897249	G	A	G	A
rs4832054	2	87058580	A	G	A	G
rs79827501	2	38431267	T	G	T	G
rs11579717	1	199999121	A	G	A	G
rs408686	2	39177320	A	C	A	C
rs6751481	2	38897810	C	T	C	T
rs11579717	1	199999121	A	G	A	G
rs138915779	1	190079170	A	C	A	C
rs17607399	2	41884212	T	C	T	C
rs34645541	1	195875605	A	C	A	C
rs6751481	2	38897810	C	T	C	T
rs7530076	1	195825276	A	G	A	G
rs11579717	1	199999121	A	G	A	G
rs138915779	1	190079170	A	C	A	C
rs61839660	10	6094697	T	C	T	C
rs11579717	1	199999121	A	G	A	G
rs12712610	2	38897249	G	A	G	A
rs2367376	2	87009742	C	T	C	T
rs11579717	1	199999121	A	G	A	G
rs17607399	2	41884212	T	C	T	C
rs6751481	2	38897810	C	T	C	T
rs11579717	1	199999121	A	G	A	G
rs138915779	1	190079170	A	C	A	C
rs11579717	1	199999121	A	G	A	G
rs11579717	1	199999121	A	G	A	G
rs11890666	2	38745822	A	G	A	G
rs12712610	2	38897249	G	A	G	A
rs143132810	10	96890004	C	T	C	T
rs185474383	10	96311553	T	G	T	G
rs34645541	1	195875605	A	C	A	C
rs35909109	10	98014850	T	C	T	C
rs7079761	10	97842115	A	G	A	G
rs72809762	10	97294154	T	C	T	C
rs77520132	10	98160835	A	G	A	G
rs11579717	1	199999121	A	G	A	G
rs138915779	1	190079170	A	C	A	C
rs408686	2	39177320	A	C	A	C
rs6751481	2	38897810	C	T	C	T
rs11579717	1	199999121	A	G	A	G
rs116759767	1	189981116	A	G	A	G
rs11579717	1	199999121	A	G	A	G
rs17607399	2	41884212	T	C	T	C
rs6751481	2	38897810	C	T	C	T
rs11579717	1	199999121	A	G	A	G
rs11579717	1	199999121	A	G	A	G
rs138915779	1	190079170	A	C	A	C
rs61839660	10	6094697	T	C	T	C
rs11579717	1	199999121	A	G	A	G
rs138915779	1	190079170	A	C	A	C
rs145947216	2	38596674	T	C	T	C
rs147692482	2	39864242	T	C	T	C
rs34558770	2	38933040	T	C	T	C
rs7605193	2	38698112	G	T	G	T
rs79747818	2	41757305	T	C	T	C
rs917116	7	28172739	G	T	G	T
rs11598494	10	6178941	C	T	C	T

rs1887027	10	6113782	T	C	T	C
rs3793662	9	95886051	T	C	T	C
rs11598494	10	6178941	C	T	C	T
rs1887027	10	6113782	T	C	T	C
rs3793662	9	95886051	T	C	T	C
rs11598494	10	6178941	C	T	C	T
rs1887027	10	6113782	T	C	T	C
rs3793662	9	95886051	T	C	T	C
rs11598494	10	6178941	C	T	C	T
rs1887027	10	6113782	T	C	T	C
rs116030815	1	42691989	C	T	C	T
rs7559619	2	38892207	G	A	G	A
rs11615628	12	6903631	A	G	A	G
rs2844608	6	31247414	T	C	T	C
rs11615628	12	6903631	A	G	A	G
rs2253487	6	31249127	A	G	A	G
rs9263475	6	31050348	G	A	G	A
rs11615628	12	6903631	A	G	A	G
rs11615628	12	6903631	A	G	A	G
rs9264532	6	31234381	T	C	T	C
rs11615628	12	6903631	A	G	A	G
rs11615628	12	6903631	A	G	A	G
rs11615628	12	6903631	A	G	A	G
rs11615628	12	6903631	A	G	A	G
rs11615628	12	6903631	A	G	A	G
rs11615628	12	6903631	A	G	A	G
rs9264532	6	31234381	T	C	T	C
rs116275309	1	150863650	A	G	A	G
rs145828126	1	120233380	A	G	A	G
rs1801274	1	161479745	G	A	G	A
rs80178684	8	59726631	G	A	G	A
rs116275309	1	150863650	A	G	A	G
rs145828126	1	120233380	A	G	A	G
rs1801274	1	161479745	G	A	G	A
rs80178684	8	59726631	G	A	G	A
rs11641185	16	10986596	T	C	T	C
rs116423144	5	174310054	A	G	A	G
rs11643297	16	28973268	G	A	G	A
rs75968672	1	206890212	G	A	G	A
rs11643297	16	28973268	G	A	G	A
rs75968672	1	206890212	G	A	G	A
rs11644019	16	10863414	T	C	T	C
rs7748976	6	33890451	A	G	A	G
rs9296042	6	32736005	T	C	T	C
rs116517602	1	198922068	T	C	T	C
rs12712613	2	38948860	T	G	T	G
rs11653761	17	2716535	T	G	T	G
rs12874404	13	108993494	G	A	G	A
rs11653761	17	2716535	T	G	T	G
rs11777835	8	144986103	C	T	C	T
rs9520836	13	108957063	G	A	G	A
rs11657118	17	2714118	C	T	C	T
rs12874404	13	108993494	G	A	G	A
rs11657531	17	2731370	T	C	T	C
rs12874404	13	108993494	G	A	G	A
rs11657759	17	2715961	C	A	C	A
rs12874404	13	108993494	G	A	G	A
rs116844769	14	65713895	A	G	A	G

rs11687597	2	136926146	G	A	G	A
rs7840395	8	144984956	G	A	G	A
rs117067704	17	62128083	G	A	G	A
rs62045817	16	89045233	T	C	T	C
rs722926	17	8677215	T	C	T	C
rs117086217	22	39876838	G	A	G	A
rs13058590	22	39641412	T	C	T	C
rs143944768	22	44378670	T	C	T	C
rs144886236	22	42326166	C	T	C	T
rs146069723	22	43339331	T	C	T	C
rs2142827	22	42874781	A	G	A	G
rs5758649	22	42605548	T	C	T	C
rs117086217	22	39876838	G	A	G	A
rs13058590	22	39641412	T	C	T	C
rs146069723	22	43339331	T	C	T	C
rs2142827	22	42874781	A	G	A	G
rs5758649	22	42605548	T	C	T	C
rs117086217	22	39876838	G	A	G	A
rs142780018	22	44395926	A	G	A	G
rs2142827	22	42874781	A	G	A	G
rs5758649	22	42605548	T	C	T	C
rs75056525	22	43349884	A	G	A	G
rs117086217	22	39876838	G	A	G	A
rs13058590	22	39641412	T	C	T	C
rs146069723	22	43339331	T	C	T	C
rs2142827	22	42874781	A	G	A	G
rs5758649	22	42605548	T	C	T	C
rs117086217	22	39876838	G	A	G	A
rs13058590	22	39641412	T	C	T	C
rs143944768	22	44378670	T	C	T	C
rs146069723	22	43339331	T	C	T	C
rs2142827	22	42874781	A	G	A	G
rs4657041	1	161478859	C	T	C	T
rs5758649	22	42605548	T	C	T	C
rs77657071	22	42324463	A	G	A	G
rs117086217	22	39876838	G	A	G	A
rs142780018	22	44395926	A	G	A	G
rs2142827	22	42874781	A	G	A	G
rs4657041	1	161478859	C	T	C	T
rs5758649	22	42605548	T	C	T	C
rs75056525	22	43349884	A	G	A	G
rs117147408	6	101980039	G	A	G	A
rs12986962	2	111808558	G	A	G	A
rs4796089	17	33814758	C	T	C	T
rs117295081	20	45333273	G	A	G	A
rs1801274	1	161479745	G	A	G	A
rs60629714	9	95889160	C	T	C	T
rs745307	20	44747086	A	G	A	G
rs117308259	14	31993501	C	T	C	T
rs11748905	5	80934634	C	T	C	T
rs11751198	6	31753526	A	G	A	G
rs55809481	19	49872521	C	T	C	T
rs61731791	19	50940778	T	C	T	C
rs78287249	11	60157281	T	G	T	G
rs62183989	2	204609402	T	C	T	C
rs56209416	2	204627500	C	T	C	T
rs117653391	10	97551952	A	G	A	G
rs4707609	6	90946479	C	T	C	T

rs117705111	22	41957510	A	G	A	G
rs12170003	22	37748690	T	C	T	C
rs148289768	22	43472311	C	A	C	A
rs5996088	22	42323913	T	C	T	C
rs117913408	6	102173117	G	A	G	A
rs79086781	6	100526652	T	C	T	C
rs11818051	10	97972294	T	C	T	C
rs12778618	10	95633120	C	T	C	T
rs17111341	10	97881031	C	T	C	T
rs7083411	10	97606602	T	C	T	C
rs72812685	10	95845989	A	G	A	G
rs11818051	10	97972294	T	C	T	C
rs12778618	10	95633120	C	T	C	T
rs17111341	10	97881031	C	T	C	T
rs2901833	10	96998591	A	G	A	G
rs4381306	10	97484131	T	C	T	C
rs72812685	10	95845989	A	G	A	G
rs11818051	10	97972294	T	C	T	C
rs12778618	10	95633120	C	T	C	T
rs12778797	10	96998688	C	T	C	T
rs17111341	10	97881031	C	T	C	T
rs72812685	10	95845989	A	G	A	G
rs7913082	10	97561886	C	T	C	T
rs569911	1	160798209	C	T	C	T
rs71632979	1	161526397	G	A	G	A
rs17583875	1	197924770	A	G	A	G
rs2463509	2	103672986	T	C	T	C
rs150956189	1	214625781	C	T	C	T
rs17583875	1	197924770	A	G	A	G
rs2463509	2	103672986	T	C	T	C
rs12901644	15	36456429	T	C	T	C
rs3131627	6	31415065	G	A	G	A
rs11954223	5	67413499	A	G	A	G
rs9520836	13	108957063	G	A	G	A
rs9916257	17	33797371	T	G	T	G
rs11954223	5	67413499	A	G	A	G
rs12874404	13	108993494	G	A	G	A
rs183053322	1	25076059	T	C	T	C
rs7218011	17	33773022	G	A	G	A
rs9916257	17	33797371	T	G	T	G
rs11961777	6	32379535	C	T	C	T
rs1800973	12	69744014	A	C	A	C
rs11961777	6	32379535	C	T	C	T
rs1354106	19	51737991	G	T	G	T
rs2071473	6	32782605	T	C	T	C
rs11961777	6	32379535	C	T	C	T
rs1800973	12	69744014	A	C	A	C
rs12075684	1	169620673	G	A	G	A
rs6427213	1	169713146	G	A	G	A
rs12145132	1	198636736	G	A	G	A
rs12752576	1	168605042	G	A	G	A
rs61814886	1	167136405	A	G	A	G
rs6658665	1	166460696	G	A	G	A
rs6668517	1	26556870	C	A	C	A
rs74620952	11	117753136	C	T	C	T
rs41289614	3	45972492	T	C	T	C
rs4657649	1	167173140	C	T	C	T
rs12206866	6	107159822	T	C	T	C

rs7745305	6	30913475	C	T	C	T
rs12212931	6	15913304	G	T	G	T
rs1941018	11	60268994	G	A	G	A
rs354026	19	49841054	T	G	T	G
rs12212931	6	15913304	G	T	G	T
rs30003	19	49846398	C	T	C	T
rs4939384	11	60273642	G	A	G	A
rs72836542	6	14091682	T	C	T	C
rs12342539	9	4327960	T	C	T	C
rs12722531	10	6076076	A	G	A	G
rs706779	10	6098824	C	T	C	T
rs12346499	9	113902881	T	C	T	C
rs12459503	19	52161035	C	T	C	T
rs3865444	19	51727962	A	C	A	C
rs7351079	19	51574038	G	A	G	A
rs78949787	19	51591138	T	C	T	C
rs12554596	9	37002142	G	A	G	A
rs2066399	6	15880540	A	G	A	G
rs2070770	11	60230531	T	C	T	C
rs30003	19	49846398	C	T	C	T
rs12596540	16	10970476	G	A	G	A
rs1723018	1	167433420	G	A	G	A
rs12598451	16	10982607	A	G	A	G
rs71632979	1	161526397	G	A	G	A
rs805288	6	31678028	T	C	T	C
rs885947	6	29696656	A	G	A	G
rs9268628	6	32405865	C	A	C	A
rs1264570	6	30365210	T	C	T	C
rs12660712	6	29734634	A	C	A	C
rs130065	6	31122500	A	G	A	G
rs13024436	2	86534521	A	G	A	G
rs1356625	2	86022747	C	T	C	T
rs6903896	6	31168029	A	G	A	G
rs938487	2	87009488	G	A	G	A
rs1264580	6	30301230	A	G	A	G
rs9461744	6	32388317	G	A	G	A
rs12675296	8	6828795	A	G	A	G
rs1886654	1	236105910	C	T	C	T
rs2981398	8	6801712	G	A	G	A
rs12675298	8	6828813	A	G	A	G
rs445	7	92408370	T	C	T	C
rs12712610	2	38897249	G	A	G	A
rs12712610	2	38897249	G	A	G	A
rs12712610	2	38897249	G	A	G	A
rs12712610	2	38897249	G	A	G	A
rs12722600	10	6054765	T	C	T	C
rs12778618	10	95633120	C	T	C	T
rs3181132	10	97601894	G	A	G	A
rs12865434	13	108970631	G	A	G	A
rs12874404	13	108993494	G	A	G	A
rs12874404	13	108993494	G	A	G	A
rs12874404	13	108993494	G	A	G	A
rs6032660	20	44730245	A	G	A	G
rs12874404	13	108993494	G	A	G	A
rs9892810	17	2716155	C	T	C	T
rs12874404	13	108993494	G	A	G	A
rs12874404	13	108993494	G	A	G	A
rs12874404	13	108993494	G	A	G	A

rs66777340	17	2716327	T	C	T	C
rs12874404	13	108993494	G	A	G	A
rs12921174	16	430304	C	T	C	T
rs2949661	1	167424924	T	C	T	C
rs4657649	1	167173140	C	T	C	T
rs61814872	1	167113055	T	C	T	C
rs79179998	3	45947066	A	G	A	G
rs12986962	2	111808558	G	A	G	A
rs58745116	17	33803592	A	G	A	G
rs56262516	6	29930939	A	G	A	G
rs13023673	2	38594483	C	T	C	T
rs6707421	2	38897781	G	A	G	A
rs79827501	2	38431267	T	G	T	G
rs13058590	22	39641412	T	C	T	C
rs142780018	22	44395926	A	G	A	G
rs146069723	22	43339331	T	C	T	C
rs150612106	22	40175781	A	G	A	G
rs4657041	1	161478859	C	T	C	T
rs5758649	22	42605548	T	C	T	C
rs73165129	22	42315371	T	C	T	C
rs13058590	22	39641412	T	C	T	C
rs142780018	22	44395926	A	G	A	G
rs146069723	22	43339331	T	C	T	C
rs150612106	22	40175781	A	G	A	G
rs4657041	1	161478859	C	T	C	T
rs5758649	22	42605548	T	C	T	C
rs73165129	22	42315371	T	C	T	C
rs13172065	5	35887454	G	A	G	A
rs1573734	16	423619	T	C	T	C
rs28680958	1	173848808	A	G	A	G
rs13210358	6	102272234	T	C	T	C
rs1322067	9	117660933	G	A	G	A
rs3087456	16	10970902	A	G	A	G
rs13390252	2	204596729	G	A	G	A
rs59639671	2	39003961	T	C	T	C
rs1375493	2	182323766	A	G	A	G
rs2418135	9	113901309	A	G	A	G
rs445	7	92408370	T	C	T	C
rs138915779	1	190079170	A	C	A	C
rs17583875	1	197924770	A	G	A	G
rs7530076	1	195825276	A	G	A	G
rs139084170	6	43351686	A	C	A	C
rs241407	6	32862740	T	C	T	C
rs9267517	6	31573110	A	G	A	G
rs9276702	6	32756741	G	A	G	A
rs139249541	6	102294761	G	A	G	A
rs62184019	2	204633784	T	C	T	C
rs79086781	6	100526652	T	C	T	C
rs139249541	6	102294761	G	A	G	A
rs6755452	2	87035076	G	T	G	T
rs9896155	17	33794697	G	A	G	A
rs139249541	6	102294761	G	A	G	A
rs79251210	6	100468602	A	G	A	G
rs9896155	17	33794697	G	A	G	A
rs139249541	6	102294761	G	A	G	A
rs79086781	6	100526652	T	C	T	C
rs139249541	6	102294761	G	A	G	A
rs79251210	6	100468602	A	G	A	G

rs139249541	6	102294761	G	A	G	A
rs75494211	6	101911859	A	G	A	G
rs7819099	8	144992862	G	A	G	A
rs78268116	11	112705025	T	C	T	C
rs9916629	17	33796260	C	T	C	T
rs139249541	6	102294761	G	A	G	A
rs79251210	6	100468602	A	G	A	G
rs9916629	17	33796260	C	T	C	T
rs139249541	6	102294761	G	A	G	A
rs79086781	6	100526652	T	C	T	C
rs139260757	2	105578959	T	C	T	C
rs80330977	5	158276610	A	G	A	G
rs139290596	22	44349795	G	A	G	A
rs146069723	22	43339331	T	C	T	C
rs150612106	22	40175781	A	G	A	G
rs5758649	22	42605548	T	C	T	C
rs73165129	22	42315371	T	C	T	C
rs139489968	1	208017826	A	G	A	G
rs36102158	15	50363583	A	G	A	G
rs4406271	6	109598213	A	G	A	G
rs586030	1	208170487	T	C	T	C
rs139795227	1	92842367	C	A	C	A
rs2325259	6	90850164	C	T	C	T
rs6445484	3	52503800	G	A	G	A
rs139795227	1	92842367	C	A	C	A
rs141264358	1	90530314	C	T	C	T
rs758800	3	52529266	C	T	C	T
rs76064946	1	93926697	G	T	G	T
rs79015439	1	94803124	T	C	T	C
rs139795227	1	92842367	C	A	C	A
rs2325259	6	90850164	C	T	C	T
rs6445506	3	52528292	C	T	C	T
rs71632979	1	161526397	G	A	G	A
rs140088532	2	105581937	C	T	C	T
rs1403755	18	30263492	T	C	T	C
rs2875404	3	76674553	T	C	T	C
rs78667279	2	54957709	A	G	A	G
rs142456232	11	4960640	T	C	T	C
rs142456232	11	4960640	T	C	T	C
rs17552904	7	50318308	T	G	T	G
rs722926	17	8677215	T	C	T	C
rs78736654	11	5544477	G	T	G	T
rs143140559	1	160972255	T	C	T	C
rs148765873	1	161221713	C	T	C	T
rs71632979	1	161526397	G	A	G	A
rs71639910	1	161937774	A	G	A	G
rs143407337	7	1095701	T	C	T	C
rs3092963	3	46396938	G	A	G	A
rs143407337	7	1095701	T	C	T	C
rs3092963	3	46396938	G	A	G	A
rs1800973	12	69744014	A	C	A	C
rs445	7	92408370	T	C	T	C
rs876036	7	50307710	C	T	C	T
rs62395272	6	31394424	T	C	T	C
rs6934244	6	33024032	A	C	A	C
rs146765347	11	11413255	T	C	T	C
rs60499622	10	126551228	T	C	T	C
rs147487472	10	6191376	A	C	A	C

rs3793662	9	95886051	T	C	T	C
rs41294937	10	6106621	C	T	C	T
rs147487472	10	6191376	A	C	A	C
rs149662107	7	47871972	A	G	A	G
rs62626323	10	6115678	A	C	A	C
rs147487472	10	6191376	A	C	A	C
rs41294937	10	6106621	C	T	C	T
rs147487472	10	6191376	A	C	A	C
rs17403348	10	5827901	C	T	C	T
rs3793662	9	95886051	T	C	T	C
rs41294937	10	6106621	C	T	C	T
rs147487472	10	6191376	A	C	A	C
rs17403348	10	5827901	C	T	C	T
rs3793662	9	95886051	T	C	T	C
rs62626323	10	6115678	A	C	A	C
rs147678401	1	161035780	A	G	A	G
rs71632979	1	161526397	G	A	G	A
rs71639910	1	161937774	A	G	A	G
rs147763768	10	97548303	A	G	A	G
rs147850744	17	56374364	T	C	T	C
rs445	7	92408370	T	C	T	C
rs77771777	17	55927801	T	C	T	C
rs148031710	6	31148873	C	T	C	T
rs148765873	1	161221713	C	T	C	T
rs191217950	1	160940671	A	G	A	G
rs71632979	1	161526397	G	A	G	A
rs71639910	1	161937774	A	G	A	G
rs149192849	22	42270864	C	T	C	T
rs4822134	22	42867332	C	T	C	T
rs73165129	22	42315371	T	C	T	C
rs1569590	6	15898884	G	A	G	A
rs55809481	19	49872521	C	T	C	T
rs1569590	6	15898884	G	A	G	A
rs2070770	11	60230531	T	C	T	C
rs30003	19	49846398	C	T	C	T
rs9461562	6	29778271	G	A	G	A
rs1571025	10	6134869	G	A	G	A
rs6602392	10	6078079	A	C	A	C
rs1571026	10	6135025	G	T	G	T
rs1573732	16	423420	T	C	T	C
rs2228015	17	38715186	C	T	C	T
rs61968809	13	106399307	A	G	A	G
rs75983909	17	38723925	T	C	T	C
rs1573732	16	423420	T	C	T	C
rs2228015	17	38715186	C	T	C	T
rs1573734	16	423619	T	C	T	C
rs876036	7	50307710	C	T	C	T
rs16988872	19	51580161	T	C	T	C
rs3865444	19	51727962	A	C	A	C
rs17201560	6	32047268	C	T	C	T
rs2571390	6	29923554	C	T	C	T
rs3020726	2	87016506	G	A	G	A
rs1723016	1	167433056	C	T	C	T
rs1723018	1	167433420	G	A	G	A
rs1723018	1	167433420	G	A	G	A
rs61814886	1	167136405	A	G	A	G
rs858545	1	167461391	C	A	C	A
rs1723018	1	167433420	G	A	G	A

rs62028293	16	50264054	C	T	C	T
rs858545	1	167461391	C	A	C	A
rs1723018	1	167433420	G	A	G	A
rs1723018	1	167433420	G	A	G	A
rs1723018	1	167433420	G	A	G	A
rs858545	1	167461391	C	A	C	A
rs17552904	7	50318308	T	G	T	G
rs17583875	1	197924770	A	G	A	G
rs408686	2	39177320	A	C	A	C
rs6751481	2	38897810	C	T	C	T
rs17583875	1	197924770	A	G	A	G
rs58905133	2	38887964	G	A	G	A
rs17583875	1	197924770	A	G	A	G
rs3130685	6	31206206	T	C	T	C
rs17583875	1	197924770	A	G	A	G
rs61839660	10	6094697	T	C	T	C
rs17583875	1	197924770	A	G	A	G
rs4959028	6	32383138	G	A	G	A
rs2596459	6	31417540	T	G	T	G
rs6707421	2	38897781	G	A	G	A
rs653178	12	112007756	T	C	T	C
rs62400929	6	31159546	G	A	G	A
rs62400929	6	31159546	G	A	G	A
rs9501107	6	31392047	T	G	T	G
rs61839660	10	6094697	T	C	T	C
rs17664049	18	48685445	T	C	T	C
rs2480679	1	167407517	G	A	G	A
rs2988279	1	167419839	A	G	A	G
rs1773542	1	167432949	C	T	C	T
rs17824379	3	5810961	G	A	G	A
rs1799693	6	106992464	G	A	G	A
rs9398107	6	107155687	G	A	G	A
rs1800973	12	69744014	A	C	A	C
rs62395272	6	31394424	T	C	T	C
rs1800973	12	69744014	A	C	A	C
rs77762937	2	203467470	T	G	T	G
rs1800973	12	69744014	A	C	A	C
rs1800973	12	69744014	A	C	A	C
rs762789	3	46402627	A	G	A	G
rs77465361	5	159009803	T	G	T	G
rs1801274	1	161479745	G	A	G	A
rs1801274	1	161479745	G	A	G	A
rs4683184	3	46187707	A	G	A	G
rs1801274	1	161479745	G	A	G	A
rs1801274	1	161479745	G	A	G	A
rs1801274	1	161479745	G	A	G	A
rs2649752	3	39351805	A	C	A	C
rs1801274	1	161479745	G	A	G	A
rs72865957	3	39355919	A	G	A	G
rs1801274	1	161479745	G	A	G	A
rs1801274	1	161479745	G	A	G	A
rs3865444	19	51727962	A	C	A	C
rs73051934	19	48582916	G	A	G	A
rs7351079	19	51574038	G	A	G	A
rs78949787	19	51591138	T	C	T	C
rs1801274	1	161479745	G	A	G	A
rs2664523	20	45318042	A	G	A	G
rs6687275	1	161515638	G	T	G	T

rs745307	20	44747086	A	G	A	G
rs1801274	1	161479745	G	A	G	A
rs3865444	19	51727962	A	C	A	C
rs4802858	19	52253150	C	T	C	T
rs58483275	19	51868443	T	C	T	C
rs7351079	19	51574038	G	A	G	A
rs78949787	19	51591138	T	C	T	C
rs1801274	1	161479745	G	A	G	A
rs1801274	1	161479745	G	A	G	A
rs1801274	1	161479745	G	A	G	A
rs1801274	1	161479745	G	A	G	A
rs1801274	1	161479745	G	A	G	A
rs3820097	1	161049358	A	G	A	G
rs3826007	15	80263217	T	C	T	C
rs1801274	1	161479745	G	A	G	A
rs8046381	16	31379355	C	T	C	T
rs1801274	1	161479745	G	A	G	A
rs1801274	1	161479745	G	A	G	A
rs72865957	3	39355919	A	G	A	G
rs1801274	1	161479745	G	A	G	A
rs3820097	1	161049358	A	G	A	G
rs3826007	15	80263217	T	C	T	C
rs61839660	10	6094697	T	C	T	C
rs183053322	1	25076059	T	C	T	C
rs7216096	17	33772954	A	G	A	G
rs9916257	17	33797371	T	G	T	G
rs183053322	1	25076059	T	C	T	C
rs6721978	2	232290157	C	T	C	T
rs185048341	9	107953420	A	G	A	G
rs67760360	15	80260872	A	G	A	G
rs1883832	20	44746982	C	T	C	T
rs41295093	10	6120173	A	G	A	G
rs1883832	20	44746982	C	T	C	T
rs2250246	12	6553016	G	A	G	A
rs1883832	20	44746982	C	T	C	T
rs25680	12	6554628	A	G	A	G
rs35574803	3	32533246	C	T	C	T
rs75918255	3	32945761	A	G	A	G
rs7621492	3	32302456	A	G	A	G
rs76929865	3	31134534	C	A	C	A
rs1883832	20	44746982	C	T	C	T
rs30006	19	49847916	C	T	C	T
rs4716010	6	15898371	A	G	A	G
rs61731791	19	50940778	T	C	T	C
rs1886730	1	2488608	C	T	C	T
rs1886730	1	2488608	C	T	C	T
rs1886730	1	2488608	C	T	C	T
rs1886730	1	2488608	C	T	C	T
rs1886730	1	2488608	C	T	C	T
rs1886730	1	2488608	C	T	C	T
rs191217950	1	160940671	A	G	A	G
rs71632979	1	161526397	G	A	G	A
rs71639910	1	161937774	A	G	A	G
rs191217950	1	160940671	A	G	A	G
rs71632979	1	161526397	G	A	G	A
rs71639910	1	161937774	A	G	A	G
rs191217950	1	160940671	A	G	A	G
rs71632979	1	161526397	G	A	G	A

rs71639910	1	161937774	A	G	A	G
rs1941018	11	60268994	G	A	G	A
rs30003	19	49846398	C	T	C	T
rs388354	7	40190449	C	A	C	A
rs72836542	6	14091682	T	C	T	C
rs1973872	2	204631016	G	T	G	T
rs72928038	6	90976768	A	G	A	G
rs1973872	2	204631016	G	T	G	T
rs72928038	6	90976768	A	G	A	G
rs28607988	3	45913338	C	A	C	A
rs2949661	1	167424924	T	C	T	C
rs4657649	1	167173140	C	T	C	T
rs61814872	1	167113055	T	C	T	C
rs2018404	14	105695446	T	C	T	C
rs28669075	15	40856521	T	C	T	C
rs2066399	6	15880540	A	G	A	G
rs4939384	11	60273642	G	A	G	A
rs72836542	6	14091682	T	C	T	C
rs2066399	6	15880540	A	G	A	G
rs34486765	19	49915911	A	G	A	G
rs72836542	6	14091682	T	C	T	C
rs7939177	11	60266070	T	C	T	C
rs2070901	1	161185058	T	G	T	G
rs2092305	6	33100189	G	A	G	A
rs72770017	16	10973612	A	G	A	G
rs2164210	2	136580287	C	T	C	T
rs2181036	9	117649136	C	T	C	T
rs3087456	16	10970902	A	G	A	G
rs72779785	16	10569039	G	A	G	A
rs2181036	9	117649136	C	T	C	T
rs3087456	16	10970902	A	G	A	G
rs72779785	16	10569039	G	A	G	A
rs2182176	1	2499254	A	G	A	G
rs2227313	1	2487766	C	T	C	T
rs2227313	1	2487766	C	T	C	T
rs2234161	1	2491205	T	C	T	C
rs2844594	6	31258393	A	G	A	G
rs2234161	1	2491205	T	C	T	C
rs2236073	6	106898421	A	G	A	G
rs9400058	6	107164076	G	A	G	A
rs2236073	6	106898421	A	G	A	G
rs7246669	19	16442023	G	A	G	A
rs72948545	6	107168477	G	A	G	A
rs2236073	6	106898421	A	G	A	G
rs9400058	6	107164076	G	A	G	A
rs2236073	6	106898421	A	G	A	G
rs72948545	6	107168477	G	A	G	A
rs2236073	6	106898421	A	G	A	G
rs72948545	6	107168477	G	A	G	A
rs2236073	6	106898421	A	G	A	G
rs9400058	6	107164076	G	A	G	A
rs2236073	6	106898421	A	G	A	G
rs72948545	6	107168477	G	A	G	A
rs2236073	6	106898421	A	G	A	G
rs9400058	6	107164076	G	A	G	A
rs2236073	6	106898421	A	G	A	G
rs9400058	6	107164076	G	A	G	A
rs2236947	3	50371432	A	C	A	C

rs445	7	92408370	T	C	T	C
rs2250246	12	6553016	G	A	G	A
rs35574803	3	32533246	C	T	C	T
rs4810485	20	44747947	G	T	G	T
rs2250246	12	6553016	G	A	G	A
rs4239702	20	44749251	C	T	C	T
rs2275848	9	95887320	T	G	T	G
rs41294937	10	6106621	C	T	C	T
rs2337147	18	46483810	A	G	A	G
rs77346511	11	5268498	T	C	T	C
rs2455069	19	51728641	G	A	G	A
rs4499342	19	8786913	T	C	T	C
rs2490437	1	161937517	C	T	C	T
rs71632989	1	161533055	G	T	G	T
rs2517515	6	31029258	T	C	T	C
rs5750829	22	39838018	G	A	G	A
rs2523614	6	31320654	C	T	C	T
rs2523882	6	31042217	T	C	T	C
rs3020726	2	87016506	G	A	G	A
rs2523887	6	31040725	G	A	G	A
rs2571390	6	29923554	C	T	C	T
rs25680	12	6554628	A	G	A	G
rs35574803	3	32533246	C	T	C	T
rs4810485	20	44747947	G	T	G	T
rs56237381	3	32924804	A	G	A	G
rs75918255	3	32945761	A	G	A	G
rs2569177	5	139992162	T	G	T	G
rs6671847	1	161478810	A	G	A	G
rs2792563	1	213100225	A	G	A	G
rs28424875	4	42664508	C	A	C	A
rs55971447	1	161515326	T	C	T	C
rs72926952	2	203485086	T	G	T	G
rs2844804	6	29942126	G	T	G	T
rs3020726	2	87016506	G	A	G	A
rs2857229	12	6900616	G	A	G	A
rs2857229	12	6900616	G	A	G	A
rs28634695	15	40856113	A	G	A	G
rs35501848	17	62021003	T	G	T	G
rs9639820	7	40094641	G	A	G	A
rs28669075	15	40856521	T	C	T	C
rs28669075	15	40856521	T	C	T	C
rs35501848	17	62021003	T	G	T	G
rs9639820	7	40094641	G	A	G	A
rs55971447	1	161515326	T	C	T	C
rs6925683	6	33894292	G	T	G	T
rs2949661	1	167424924	T	C	T	C
rs4657649	1	167173140	C	T	C	T
rs57319220	3	45919992	A	G	A	G
rs61814872	1	167113055	T	C	T	C
rs2949661	1	167424924	T	C	T	C
rs61814872	1	167113055	T	C	T	C
rs76540213	1	166035343	T	C	T	C
rs2949661	1	167424924	T	C	T	C
rs858496	1	167500918	G	T	G	T
rs2949661	1	167424924	T	C	T	C
rs4657649	1	167173140	C	T	C	T
rs61814872	1	167113055	T	C	T	C
rs2949669	1	167417884	T	C	T	C

rs2988277	1	167431352	T	C	T	C
rs4657649	1	167173140	C	T	C	T
rs2988279	1	167419839	A	G	A	G
rs2995089	1	167431193	A	G	A	G
rs2995089	1	167431193	A	G	A	G
rs4657649	1	167173140	C	T	C	T
rs7519927	1	167100361	A	G	A	G
rs3087456	16	10970902	A	G	A	G
rs542160	11	107676543	T	C	T	C
rs72779785	16	10569039	G	A	G	A
rs3087456	16	10970902	A	G	A	G
rs62029480	16	10651993	T	C	T	C
rs7032773	9	117645562	T	C	T	C
rs3087456	16	10970902	A	G	A	G
rs72779785	16	10569039	G	A	G	A
rs3087456	16	10970902	A	G	A	G
rs7032773	9	117645562	T	C	T	C
rs3116493	2	204589281	T	G	T	G
rs75380201	18	39778311	T	C	T	C
rs3116493	2	204589281	T	G	T	G
rs3129891	6	32415080	A	G	A	G
rs3130685	6	31206206	T	C	T	C
rs3130685	6	31206206	T	C	T	C
rs3130685	6	31206206	T	C	T	C
rs3130685	6	31206206	T	C	T	C
rs4796089	17	33814758	C	T	C	T
rs3184504	12	111884608	C	T	C	T
rs3184504	12	111884608	C	T	C	T
rs3184504	12	111884608	C	T	C	T
rs4987358	1	169665551	T	G	T	G
rs34460712	2	38935236	A	G	A	G
rs3757247	6	90957463	T	C	T	C
rs62183989	2	204609402	T	C	T	C
rs34546581	2	87021659	A	G	A	G
rs9461744	6	32388317	G	A	G	A
rs9461744	6	32388317	G	A	G	A
rs354020	19	49838891	A	C	A	C
rs35574803	3	32533246	C	T	C	T
rs4810485	20	44747947	G	T	G	T
rs35587265	3	46259296	G	A	G	A
rs6687275	1	161515638	G	T	G	T
rs35587265	3	46259296	G	A	G	A
rs55971447	1	161515326	T	C	T	C
rs362522	6	29545208	T	C	T	C
rs55971447	1	161515326	T	C	T	C
rs7748976	6	33890451	A	G	A	G
rs9296042	6	32736005	T	C	T	C
rs3793662	9	95886051	T	C	T	C
rs41295093	10	6120173	A	G	A	G
rs3793662	9	95886051	T	C	T	C
rs62626323	10	6115678	A	C	A	C
rs3793662	9	95886051	T	C	T	C
rs41294937	10	6106621	C	T	C	T
rs7078614	10	6075831	T	G	T	G
rs71632979	1	161526397	G	A	G	A
rs71639910	1	161937774	A	G	A	G
rs71632979	1	161526397	G	A	G	A
rs71639910	1	161937774	A	G	A	G

rs3856266	1	157973889	G	T	G	T
rs4656984	1	161078909	C	T	C	T
rs77418963	1	162197834	G	A	G	A
rs78569595	1	162032153	T	G	T	G
rs3865444	19	51727962	A	C	A	C
rs7351079	19	51574038	G	A	G	A
rs78949787	19	51591138	T	C	T	C
rs3865444	19	51727962	A	C	A	C
rs62114147	19	51588018	T	C	T	C
rs7351079	19	51574038	G	A	G	A
rs3865444	19	51727962	A	C	A	C
rs4499342	19	8786913	T	C	T	C
rs7351079	19	51574038	G	A	G	A
rs3865444	19	51727962	A	C	A	C
rs4802858	19	52253150	C	T	C	T
rs58483275	19	51868443	T	C	T	C
rs7351079	19	51574038	G	A	G	A
rs78949787	19	51591138	T	C	T	C
rs3865444	19	51727962	A	C	A	C
rs74253876	18	5858361	T	C	T	C
rs3865444	19	51727962	A	C	A	C
rs7351079	19	51574038	G	A	G	A
rs78949787	19	51591138	T	C	T	C
rs3865444	19	51727962	A	C	A	C
rs7351079	19	51574038	G	A	G	A
rs78949787	19	51591138	T	C	T	C
rs3865444	19	51727962	A	C	A	C
rs3865444	19	51727962	A	C	A	C
rs56043070	1	247719769	A	G	A	G
rs7351079	19	51574038	G	A	G	A
rs75773078	19	51742145	A	G	A	G
rs3865444	19	51727962	A	C	A	C
rs3917768	1	169572947	G	T	G	T
rs7526645	1	169728916	T	C	T	C
rs3934748	1	206879659	T	G	T	G
rs7189927	16	28913787	C	T	C	T
rs394199	6	33553580	T	C	T	C
rs61742525	6	33072522	A	G	A	G
rs6808893	3	188133439	T	C	T	C
rs73499473	16	10978885	T	C	T	C
rs41294937	10	6106621	C	T	C	T
rs7078614	10	6075831	T	G	T	G
rs445	7	92408370	T	C	T	C
rs445	7	92408370	T	C	T	C
rs4371876	6	31067550	A	G	A	G
rs9357097	6	30285121	T	C	T	C
rs445	7	92408370	T	C	T	C
rs4493054	16	31361988	A	G	A	G
rs4521502	5	35842748	G	T	G	T
rs4987360	1	169664867	G	A	G	A
rs7139370	13	109976273	T	C	T	C
rs9859349	3	31661754	A	G	A	G
rs45608038	2	73679013	G	A	G	A
rs75766272	1	8009985	T	C	T	C
rs4657041	1	161478859	C	T	C	T
rs4657649	1	167173140	C	T	C	T
rs4683184	3	46187707	A	G	A	G
rs79641389	3	46882338	C	A	C	A

rs4683184	3	46187707	A	G	A	G
rs79825014	3	46880193	T	G	T	G
rs4785074	16	59885737	T	C	T	C
rs4796089	17	33814758	C	T	C	T
rs4796089	17	33814758	C	T	C	T
rs4796089	17	33814758	C	T	C	T
rs4796089	17	33814758	C	T	C	T
rs6904670	6	31192797	G	A	G	A
rs7728865	5	59819941	A	G	A	G
rs4796089	17	33814758	C	T	C	T
rs4849134	2	111615015	G	A	G	A
rs4987369	1	169662742	T	G	T	G
rs548316	11	128077683	A	G	A	G
rs55971447	1	161515326	T	C	T	C
rs55971447	1	161515326	T	C	T	C
rs75072970	1	161058351	C	T	C	T
rs56216383	2	204631712	G	A	G	A
rs56262516	6	29930939	A	G	A	G
rs7425430	2	87056480	G	A	G	A
rs569911	1	160798209	C	T	C	T
rs71632979	1	161526397	G	A	G	A
rs569911	1	160798209	C	T	C	T
rs71632979	1	161526397	G	A	G	A
rs569911	1	160798209	C	T	C	T
rs71632989	1	161533055	G	T	G	T
rs7180804	15	80259074	A	G	A	G
rs865239	1	156896164	G	A	G	A
rs9916257	17	33797371	T	G	T	G
rs569911	1	160798209	C	T	C	T
rs71632979	1	161526397	G	A	G	A
rs58027502	5	139978241	T	C	T	C
rs58408589	16	31362741	G	A	G	A
rs75841521	2	59592691	A	G	A	G
rs78520150	1	19786101	A	C	A	C
rs79298699	16	31402042	T	C	T	C
rs597808	12	111973358	G	A	G	A
rs61141933	2	39388479	T	C	T	C
rs613165	6	31834398	A	G	A	G
rs61839660	10	6094697	T	C	T	C
rs61839660	10	6094697	T	C	T	C
rs78667279	2	54957709	A	G	A	G
rs61839660	10	6094697	T	C	T	C
rs61839660	10	6094697	T	C	T	C
rs61839660	10	6094697	T	C	T	C
rs61839660	10	6094697	T	C	T	C
rs62183989	2	204609402	T	C	T	C
rs62184016	2	204623481	C	T	C	T
rs72928038	6	90976768	A	G	A	G
rs62184016	2	204623481	C	T	C	T
rs72928038	6	90976768	A	G	A	G
rs62395272	6	31394424	T	C	T	C
rs9296042	6	32736005	T	C	T	C
rs62447171	7	50309890	A	G	A	G
rs7658518	4	84160279	A	G	A	G
rs62447171	7	50309890	A	G	A	G
rs7658518	4	84160279	A	G	A	G
rs62447171	7	50309890	A	G	A	G
rs7658518	4	84160279	A	G	A	G

rs62447171	7	50309890	A	G	A	G
rs7658518	4	84160279	A	G	A	G
rs62626323	10	6115678	A	C	A	C
rs6453485	5	79151752	T	C	T	C
rs6535445	4	84151427	C	T	C	T
rs6535445	4	84151427	C	T	C	T
rs6537835	1	115108914	A	G	A	G
rs71632989	1	161533055	G	T	G	T
rs71639910	1	161937774	A	G	A	G
rs7078614	10	6075831	T	G	T	G
rs7078614	10	6075831	T	G	T	G
rs6681289	1	119421209	A	G	A	G
rs6707421	2	38897781	G	A	G	A
rs6707421	2	38897781	G	A	G	A
rs6710361	2	87047145	A	G	A	G
rs9896155	17	33794697	G	A	G	A
rs6751481	2	38897810	C	T	C	T
rs6751481	2	38897810	C	T	C	T
rs6755452	2	87035076	G	T	G	T
rs9896155	17	33794697	G	A	G	A
rs6808893	3	188133439	T	C	T	C
rs71632979	1	161526397	G	A	G	A
rs71632979	1	161526397	G	A	G	A
rs71632979	1	161526397	G	A	G	A
rs71639910	1	161937774	A	G	A	G
rs78600824	1	160841013	G	A	G	A
rs71632989	1	161533055	G	T	G	T
rs876036	7	50307710	C	T	C	T
rs71632989	1	161533055	G	T	G	T
rs71639910	1	161937774	A	G	A	G
rs7189927	16	28913787	C	T	C	T
rs7245846	19	51731176	A	G	A	G
rs7351079	19	51574038	G	A	G	A
rs78949787	19	51591138	T	C	T	C
rs7245846	19	51731176	A	G	A	G
rs72677097	1	61134076	G	A	G	A
rs72915529	2	54970478	A	G	A	G
rs9302635	16	72144174	C	T	C	T
rs73165129	22	42315371	T	C	T	C
rs73402222	6	31388449	C	T	C	T
rs73402222	6	31388449	C	T	C	T
rs73735764	6	46441386	T	C	T	C
rs74529033	2	137747326	G	A	G	A
rs75320486	1	206842241	A	G	A	G
rs79856590	1	205241642	A	G	A	G
rs7559619	2	38892207	G	A	G	A
rs75766272	1	8009985	T	C	T	C
rs760462	21	46328099	C	T	C	T
rs760462	21	46328099	C	T	C	T
rs76112278	4	15915069	A	G	A	G
rs807784	4	13906853	T	G	T	G
rs76112278	4	15915069	A	G	A	G
rs76161425	11	112704995	T	C	T	C
rs77650974	18	12394263	A	G	A	G
rs77650974	18	12394263	A	G	A	G
rs77789294	14	29178686	C	T	C	T
rs778582	5	140004816	T	C	T	C
rs7801342	7	31163003	C	T	C	T

rs78268116	11	112705025	T	C	T	C
rs78268116	11	112705025	T	C	T	C
rs78358948	11	112751974	A	G	A	G
rs879299	6	15897007	G	T	G	T
rs9268498	6	32375431	C	T	C	T
rs9268832	6	32427789	C	T	C	T
rs9916257	17	33797371	T	G	T	G

EAF.exposure	EAF.outcome	BETA.exposure	BETA.outcome	SE.exposure	SE.outcome
0.1224	0.8889362	0.389	0.0092	0.04003	0.04310441
0.5475	0.332246216	-0.1509	0.0551	0.02717	0.028666013
0.5254	0.5819941	0.1606	-0.0194	0.02681	0.02761212
0.1224	0.8889362	0.4144	0.0092	0.04052	0.04310441
0.6024	0.6754331	-0.1389	0.009	0.02509	0.02887679
0.821	0.8228947	0.2375	0.0247	0.03213	0.03920499
0.3094	0.7911125	0.167	-0.0081	0.02642	0.03319777
0.6088	0.4717883	-0.236	0.0168	0.02474	0.02719056
0.3161	0.7159645	0.1752	0.0285	0.0268	0.03024686
0.8208	0.7666319	0.1788	-0.0873	0.03256	0.04036428
0.0019	0.96419689	9.768	0.1314	1.725	0.07155964
0.0003	0.9871039	26.82	0.029	4.296	0.1372175
0.0023	0.987676059	9.265	0.0902	1.585	0.128470109
0.0022	0.01415165	8.874	0.056	1.564	0.1230952
0.0006	0.9866611	17.96	0.2719	3.205	0.1138209
0.0006	0.02026086	22.4	-0.0884	3.301	0.1006472
0.0016	0.97909603	10.65	0.1129	1.782	0.09790708
0.0006	0.01336841	19.07	-0.031	3.407	0.1241491
0.0004	0.9795834	18.77	0.0397	3.342	0.1091838
0.0001	0.98304394	32.87	-0.1083	5.796	0.1119239
0.0001	0.97515327	33.95	0.1941	5.804	0.08367946
0.0001	0.9739926	36.56	0.2132	6.272	0.08694655
0.0007	0.98877191	16	0.2952	2.92	0.1360582
0.0013	0.98156476	10.8	0.1065	1.881	0.1007526
0.0006	0.9645273	23.37	0.0477	3.154	0.08083394
0.0003	0.98607117	21.5	0.2214	3.886	0.1133994
0.0003	0.97577714	23.94	0.093	3.906	0.09021363
0.0001	0.9837365	51.17	-0.0102	8.845	0.1111862
0.0003	0.01714996	34.45	-0.0405	4.832	0.1083407
0.0001	0.98187071	42.63	-0.0299	7.413	0.1145587
0.0003	0.02310309	25.69	0.0502	4.095	0.0935861
0.0001	0.98181837	33.31	-0.064	5.744	0.1041251
0.0006	0.03324949	15.79	-0.079	2.811	0.07524828
0.0006	0.98327753	18.17	-0.0866	3.072	0.1172988
0.0001	0.97758993	49	0.0325	5.703	0.09211064
0.0001	0.01306243	33.05	0.0514	5.644	0.1303671
0.0003	0.01199841	31.04	0.1773	4.31	0.1282593
0.0001	0.02740286	38.22	0.0796	4.737	0.0853657
0.0001	0.98281062	49.64	0.0712	5.557	0.1043359
0.0001	0.01424236	52.91	-0.0836	5.64	0.1254138
0.0001	0.01128583	46.68	0.0474	7.967	0.1435408
0.0006	0.02180299	19.81	-0.0027	3.224	0.09653701
0	0.01886418	100.8	0.0307	11.59	0.1093946
0.0022	0.01071407	9.036	0.2055	1.652	0.1322641
0.0001	0.98919462	35.15	-0.1931	5.793	0.1513397
0.0001	0.01544379	44.6	0.081	7.703	0.1157179
0.1892	0.7034485	0.1206	0.029	0.02163	0.02971991
0.0191	0.02907878	0.3952	-0.1512	0.06626	0.08578726
0	0.01609922	-12.04	0.1998	2.063	0.1064437
0.124	0.06602777	0.234	0.0111	0.03837	0.05490806
0.3117	0.8077573	-0.1677	0.0326	0.02967	0.03477862
0.1325	0.8378662	-0.2444	0.0026	0.0376	0.03867804
0.0888	0.91798137	-0.2765	0.0188	0.04672	0.04984935
0.5524	0.4572018	0.1554	-0.0282	0.02741	0.02719056
0.3815	0.7085882	-0.1391	-0.0099	0.02491	0.0298253
0.0103	0.92049029	118.5	-0.0163	17.59	0.04984935

0.0019	0.96419689	407.5	0.1314	40.68	0.07155964
0.0031	0.04033336	216.9	0.0583	30.85	0.06934646
0.0173	0.96841416	95.49	0.0226	13.66	0.07767225
0.0173	0.98555397	82	0.0629	13.96	0.1143479
0.0015	0.97568433	308.6	-0.1452	43.15	0.09021363
0.0026	0.96205689	206.9	-0.0544	34.55	0.07261354
0.0019	0.01377006	249.4	-0.0134	42.57	0.1144533
0.0019	0.02082668	292.3	0.0329	40.49	0.09759091
0.0114	0.96262871	95.85	0.048	16.51	0.0699788
0.0015	0.96746908	345.5	0.0779	42.75	0.08473336
0.0026	0.98285181	206.8	0.2128	32.42	0.1063383
0.0015	0.98485096	312.7	-0.0474	46.04	0.1078137
0.0012	0.01082456	386.6	0.0774	51.06	0.1448055
0.0021	0.97563359	214.8	0.0498	37.5	0.09611545
0.0037	0.02348297	189.8	0.0309	29.08	0.09727474
0.0023	0.987676059	324.4	0.0902	37.63	0.128470109
0.0038	0.98431097	172.3	-0.0798	28.61	0.1307887
0.0022	0.982179784	234.6	-0.0223	39.65	0.105600532
0.0006	0.01103262	725.8	0.0724	66.51	0.1356366
0.0004	0.988121	662.4	-0.1119	82.11	0.1394306
0.0059	0.982094194	139.3	-0.0644	25.24	0.107075989
0.0019	0.02489514	239.1	0.0241	39.88	0.08705194
0.0054	0.98520068	160.1	0.0325	23.53	0.1124509
0.0003	0.98838633	623.6	0.0323	88.02	0.1325803
0.0018	0.98539682	274.8	0.183	40.08	0.1156126
0.0041	0.0124802	192.2	-0.1399	28.52	0.145754
0.0023	0.98043859	221.5	0.0501	34.4	0.1021227
0.0034	0.0327613	177.5	-0.0683	30.9	0.07746147
0.0023	0.98209737	207.9	0.0726	36.33	0.1140317
0.0016	0.98341378	315.7	0.0382	41.99	0.117088
0.0003	0.97456223	771.6	-0.108	91.49	0.09042441
0.0006	0.977252932	794.2	-0.1597	70	0.094218439
0.0006	0.01415715	759.4	0.1028	70.06	0.117088
0.0003	0.98909167	896.2	0.014	97.49	0.1477564
0.0006	0.9866611	889.9	0.2719	73.93	0.1138209
0.0043	0.01128752	159.2	-0.1112	28.32	0.1465971
0.0015	0.96743738	268.3	-0.0619	44.19	0.0781992
0.0022	0.02510031	297	-0.1267	38.79	0.09200525
0.0006	0.02026086	1006	-0.0884	78.94	0.1006472
0.0023	0.97523846	212.9	0.0354	34.36	0.09759091
0.9974	0.98823728	-233.6	-0.1129	40.25	0.1244653
0.0012	0.98672996	420	0.2269	62.49	0.131948
0.0006	0.01336841	834.4	-0.031	80.31	0.1241491
0.0065	0.98815331	126.2	-0.1952	21.64	0.1387983
0.0035	0.9856401	194.3	-0.0968	29.94	0.1194066
0.0028	0.98131625	255.1	-0.0426	33.32	0.1043359
0.0028	0.98235331	208.3	-0.146	32.03	0.1074975
0.0032	0.0116044	186	-0.0286	31.2	0.1340558
0.0006	0.97525802	437.7	0.0254	69.06	0.08778966
0.0019	0.97958602	264.9	0.0668	39.21	0.1059167
0.0022	0.0123869	196.1	-0.0642	35.66	0.1243599
0.0035	0.98548787	272.5	-0.0651	27.65	0.1163503
0.001	0.02914985	414.6	-0.1389	53.64	0.08589265
0.0021	0.01319819	215.2	0.1873	39.24	0.1177204
0.0021	0.97482968	260.9	-0.0836	40.57	0.09653701
0.001	0.98735438	573	0.0437	57.77	0.139536
0.0034	0.9737687	224.8	0.1031	31.22	0.09284837
0.0026	0.98808371	206.4	0.1019	37.47	0.1322641

0.0079	0.0136695	141	-0.024	20.2	0.1171934
0.0021	0.98474473	246.8	-0.1021	37.81	0.1144533
0.0032	0.98261055	172.1	-0.0004	31.46	0.104652
0.0006	0.02497234	785.2	-0.0242	70.89	0.08800044
0.0079	0.97330471	111	-0.0845	19.94	0.0853657
0.0022	0.98554605	248.5	0.0877	37.09	0.1149802
0.0251	0.103848041	76.42	-0.016	11.29	0.045106814
0.0007	0.98877191	699.1	0.2952	69.55	0.1360582
0.0057	0.98403866	169.6	0.0028	23.32	0.108973
0.0025	0.9697458	245.4	-0.0034	36.99	0.08283635
0.0013	0.98156476	620.5	0.1065	43.09	0.1007526
0.0035	0.96653244	166.6	-0.0795	29.17	0.07809381
0.0113	0.01219264	98.72	-0.0864	17.57	0.1209874
0.0012	0.97867044	417.3	-0.0856	51.4	0.09495617
0.0006	0.9645273	1000	0.0477	72.99	0.08083394
0.0028	0.02405701	219.9	-0.2065	33.8	0.09179447
0.0003	0.98607117	1285	0.2214	88.96	0.1133994
0.0054	0.98867435	139.5	0.1377	25.13	0.1341612
0.0034	0.982567529	200.7	0.0511	32.7	0.108129886
0.0006	0.01310247	570.9	0.2159	70.25	0.1191958
0.0003	0.97577714	842.4	0.093	89.95	0.09021363
0.0318	0.96796895	59.98	-0.0362	10.31	0.08009621
0.0004	0.01869668	836.1	0.0101	73.46	0.1023334
0.001	0.01535632	516.8	0.0824	57.96	0.120882
0.0032	0.98751415	204.4	0.0367	36.08	0.1281539
0.0012	0.01347037	460.1	0.1009	50.43	0.1191958
0.0211	0.98473183	69.9	-0.0319	12.06	0.121409
0.0026	0.97113734	210.6	0.0112	33.76	0.08115011
0.0009	0.98857458	636.7	0.156	65.61	0.1340558
0.0029	0.9838868	181.9	-0.0997	32.71	0.111397
0.0007	0.01333926	440.2	0.033	64.38	0.1236222
0.0013	0.98772699	394.5	-0.1751	51.12	0.1423816
0.0072	0.0192333	127.1	0.0921	21.37	0.1037035
0.0001	0.9837365	1232	-0.0102	203.7	0.1111862
0.0021	0.98043422	279.2	0.0867	36.97	0.1004364
0.001	0.98681758	328.1	-0.0299	52.32	0.1164557
0.0092	0.02196956	111.8	-0.1926	18.01	0.1056005
0.0565	0.983126233	43.94	0.0736	7.987	0.103703517
0.0006	0.98295575	591.4	0.0326	73.04	0.1090784
0.0007	0.01578429	369.2	0.2467	61.96	0.1061275
0.0003	0.01862886	730	-0.1288	96.39	0.1082353
0.01	0.02203397	159.5	0.0207	18.29	0.09274298
0.0062	0.97993006	144.1	-0.0653	25.27	0.09885559
0.0025	0.983073198	210.7	-0.0367	35.78	0.104546635
0.0207	0.96998417	77.03	-0.0208	13.23	0.08652499
0.0026	0.98310219	186.1	0.0563	33.82	0.106549
0.0031	0.01340742	185.9	0.0426	33.18	0.1227791
0.0003	0.01714996	1994	-0.0405	108.9	0.1083407
0.0031	0.01329828	202.4	0.1809	32.76	0.1274162
0.006	0.98410214	207	0.0144	22.8	0.1093946
0.0016	0.98647639	235.3	-0.0519	42.7	0.121409
0.005	0.01072233	143.9	-0.0001	26.11	0.1351097
0.0056	0.98084517	152.8	-0.076	23.81	0.1054951
0.0032	0.98289946	181.2	-0.0339	33.13	0.1088676
0.0009	0.9885705	549.6	-0.0416	57.08	0.1460702
0.0016	0.013485144	301	-0.0588	43.5	0.118985031
0.0094	0.98746893	114.2	0.1236	18.22	0.1187743
0.0079	0.98877646	123.5	0.0422	20	0.1273108

0.0057	0.01385253	183.9	0.3099	22.31	0.1140317
0.0044	0.98332186	149.3	0.1083	26.38	0.107076
0.0007	0.01001303	438.9	0.1138	64.07	0.1674643
0.0013	0.01669789	266.7	-0.0502	46.83	0.1033873
0.0022	0.96910044	257.4	0.1108	36.09	0.07904231
0.0003	0.02310309	1445	0.0502	93.44	0.0935861
0.0007	0.98769911	708.1	-0.1791	63.75	0.1403792
0.0028	0.03440742	203.9	-0.0262	36.54	0.07703991
0.0022	0.98275093	339.6	-0.0613	33.81	0.1060221
0.0019	0.98173442	240.6	-0.1084	38.16	0.09906637
0.0009	0.98208131	496.1	-0.0133	55.44	0.1125563
0.0025	0.97574899	262.4	0.0492	37.13	0.08894895
0.0012	0.98145415	432.3	0.1186	52.29	0.1066544
0.0073	0.94613854	163.3	-0.0705	20.92	0.06449853
0.0012	0.98219229	320.1	0.1667	47.71	0.1101323
0.0001	0.98181837	2073	-0.064	128.5	0.1041251
0.0091	0.02619594	150.2	0.0908	18.37	0.08937051
0.0006	0.03324949	751.8	-0.079	64.6	0.07524828
0.0028	0.98532138	235.6	-0.0253	38.25	0.1172988
0.007	0.98311021	121.9	0.156	21.11	0.1129778
0.0021	0.98859344	253.3	-0.124	38.53	0.1344773
0.0026	0.98216767	230.6	0.0048	33.78	0.1103431
0.0013	0.98185971	306.1	-0.1376	48.59	0.1021227
0.0032	0.97849558	181.1	-0.1321	30.9	0.09558851
0.0006	0.98327753	887.3	-0.0866	70.63	0.1172988
0.0007	0.02400834	750.9	0.033	68.52	0.08810583
0.0009	0.02135305	428.3	0.1883	59.76	0.09474539
0.0001	0.9882903	1200	-0.2103	109.5	0.1278378
0.0007	0.97531989	413.3	-0.0531	62.46	0.09232142
0.004	0.01219474	177.1	0.1851	31.66	0.1203551
0.0009	0.98832722	436.5	-0.1834	60.77	0.1299456
0.0066	0.03844981	156.1	-0.061	22.14	0.07219198
0.9988	0.03954309	-388.2	0.1042	50.03	0.07208659
0.0066	0.97987692	164.3	-0.0579	21.61	0.09853942
0.0018	0.987338319	270.7	-0.0814	41.43	0.123306011
0.001	0.01906996	542.5	-0.256	54.89	0.1166665
0.0009	0.98267978	336.7	0.033	53.43	0.1052844
0.001	0.97214318	475	-0.0416	55.24	0.08884356
0.0035	0.97338396	158.9	0.0033	28.76	0.08620882
0.0034	0.01584373	197.3	-0.1385	29.42	0.1091838
0.0025	0.98864644	286.8	-0.1086	38.31	0.1380606
0.0022	0.9849388	212.6	-0.0084	36.32	0.1117131
0.0007	0.98368665	330.6	-0.016	59.91	0.1110808
0.0037	0.01326142	160.9	-0.2425	28.33	0.1204605
0.0007	0.01729143	398.3	0.1422	60.2	0.1041251
0.0069	0.98550463	149.1	-0.1418	21.18	0.1177204
0.0032	0.98770375	255.1	-0.1127	29.73	0.1378498
0.0001	0.97758993	1757	0.0325	129.6	0.09211064
0.0022	0.01504849	235.6	0.1061	37	0.1138209
0.0016	0.98410962	263	0.0042	43.72	0.1095
0.999	0.03531652	-548.4	-0.0412	57.56	0.0760914
0.0015	0.95540653	342.1	0.0666	46.48	0.07271893
0.0015	0.9885306	397	-0.0216	48.27	0.1225683
0.0006	0.98292753	643.8	-0.0691	80.36	0.1118185
0.0013	0.02277337	424.9	-0.014	49.05	0.09938254
0.0012	0.0309615	381.2	-0.0075	58.11	0.0771453
0.0007	0.97536527	520	0.0413	59.93	0.09748552
0.0003	0.01199841	1003	0.1773	98.17	0.1282593

0.0004	0.02533842	568.9	0.082	78.84	0.08473336
0.0016	0.96759374	250	0.047	43.75	0.07809381
0.004	0.01959568	204.6	-0.0663	27.04	0.09790708
0.0116	0.96879091	130.7	0.1882	17.31	0.08157167
0.0029	0.01820527	218.4	0.001	33.18	0.1025442
0.0041	0.98457324	166.4	-0.1342	27.44	0.1137155
0.0004	0.02200173	658.4	0.0464	82.87	0.08989746
0.0076	0.97704832	125.6	-0.116	19.43	0.09611545
0.0122	0.98326187	90.23	-0.0966	16.28	0.1069706
0.001	0.97241604	442.9	0.1298	54.79	0.08410102
0.0032	0.02680152	184.9	0.1269	31.03	0.0853657
0.0022	0.97463833	212.8	-0.0876	37.83	0.08873817
0.0015	0.98581446	269.8	-0.1265	44.42	0.1198281
0.0022	0.01115302	239.6	0.0964	36.44	0.1353204
0.0029	0.98709176	207	0.051	33.71	0.1194066
0.0009	0.010292	358.8	0.1049	54.73	0.1551337
0.0013	0.98179744	353	0.0473	46.68	0.1035981
0.0006	0.9840287	559.9	0.1182	69.63	0.1028604
0.0009	0.02122599	559.6	-0.1032	56.91	0.09305915
0.0004	0.9809955	632.9	0.0986	75.09	0.1031766
0.0016	0.98255561	348.1	0.0707	45.47	0.1012796
0.9869	0.9872728	-87.22	0.053	15.38	0.1210928
0.0016	0.9668513	335	-0.0996	44.28	0.07703991
0.0079	0.98413772	130.9	-0.0089	20.78	0.1082353
0.0001	0.98281062	2721	0.0712	123.4	0.1043359
0.0029	0.98546138	203	-0.1113	35.7	0.1163503
0.0088	0.979274382	106.8	-0.1068	19.44	0.092532203
0.0004	0.97865667	634.9	-0.0282	80.9	0.1024388
0.0063	0.01150237	134.6	0.1684	22.06	0.1293132
0.001	0.03549027	364.4	0.0131	54.53	0.07872615
0.0066	0.95834935	116.6	-0.0386	21.02	0.06903029
0.0018	0.98567904	278.8	-0.0438	42.64	0.1207767
0.0007	0.01419689	404.4	-0.1716	62.01	0.1216198
0.0015	0.97570031	336.9	0.0668	45.79	0.08884356
0.0012	0.98907429	438.9	-0.0429	52.07	0.1512343
0.0015	0.97788482	287.4	0.054	43.92	0.0915837
0.0018	0.024439875	264	-0.1987	40.5	0.09790708
0.0016	0.96774718	322	-0.0546	41.59	0.07883154
0.0018	0.96761029	308.9	0.1102	44.43	0.07935848
0.0019	0.98660028	326.1	0.1684	38.46	0.1172988
0.0016	0.9850556	268.4	-0.1873	41.66	0.1156126
0.0018	0.01522108	338.9	-0.0128	40.47	0.1234114
0.0094	0.98441518	105.3	0.0487	18.42	0.1128724
0.0015	0.98231232	352	-0.1353	44.95	0.1071814
0.0103	0.97894049	101.4	0.1559	17.52	0.1001203
0.0018	0.98587311	225.9	0.0633	40.82	0.1175096
0.0097	0.97549097	132.5	-0.0522	17.32	0.09316454
0.0079	0.03022528	143.3	-0.044	20.28	0.08030699
0.0128	0.02292249	97.68	-0.1219	15.15	0.09453461
0.0013	0.984637461	287	0.2675	50.66	0.108340666
0.0001	0.02424251	1057	0.0367	134.1	0.08852739
0	0.01886418	3549	0.0307	263.7	0.1093946
0.0016	0.02192245	303.6	-0.0567	45.76	0.09221603
0.0016	0.01167217	297	0.0628	43.55	0.1351097
0.0006	0.97419497	434.2	-0.0649	63.13	0.08989746
0.0029	0.9847622	263.6	-0.0157	31.58	0.1269947
0.0022	0.01071407	331.8	0.2055	38.87	0.1322641
0.0003	0.97626379	961.3	0.0501	93.43	0.09390227

0.0053	0.04828693	163.6	-0.0011	28.37	0.06934646
0.0004	0.02243307	1117	0.1659	84.15	0.09442922
0.0073	0.98453632	141.4	0.2243	20.64	0.1232006
0.0116	0.98081326	99.34	0.2628	16.98	0.1102377
0.0001	0.98919462	1826	-0.1931	132.8	0.1513397
0.0433	0.98403694	47.59	0.1204	8.691	0.1069706
0.0004	0.98443843	774.8	-0.1946	85.45	0.1117131
0.0007	0.01779188	404.7	0.0393	62.22	0.1052844
0.0043	0.97337307	168.6	0.1023	26.99	0.08494414
0.0012	0.02245681	391.1	0.0159	49.95	0.09263759
0.0161	0.97217755	85.79	-0.2378	14.27	0.08620882
0.0031	0.02390009	193.7	-0.0244	32.62	0.08589265
0.0971	0.91276956	-0.2991	-0.0693	0.04099	0.04890085
0.1839	0.95956166	0.2182	-0.0805	0.03712	0.07429978
0.8748	0.07937202	0.207	0.0581	0.03723	0.05100864
0.203	0.834726	0.1894	0.0475	0.0315	0.03962655
0.2425	0.2448006	-0.3215	-0.0138	0.02865	0.03161693
0.0972	0.95894409	0.2358	0.0999	0.04238	0.06860873
0.5787	0.6203122	0.2317	-0.0648	0.02892	0.03045764
0.1412	0.1588857	-0.5196	0.0342	0.03725	0.03688641
0.1639	0.8232942	0.249	0.061	0.03122	0.03562174
0.0354	0.988271966	-0.408	-0.1162	0.06431	0.130894073
0.5685	0.5594995	-0.2323	-0.0028	0.02306	0.02740134
0.1639	0.8232942	0.2345	0.061	0.03229	0.03562174
0.5685	0.5594995	-0.1918	-0.0028	0.02391	0.02740134
0.1639	0.8232942	-0.2345	0.061	0.03229	0.03562174
0.5685	0.5594995	0.1918	-0.0028	0.02391	0.02740134
0.7693	0.6926367	0.2395	-0.047	0.03338	0.03172232
0.0044	0.9524443	-1.249	0.0334	0.188	0.06397158
0.328	0.615129	-0.1062	0.0206	0.01937	0.02803367
0.5294	0.4947278	-0.1633	-0.0646	0.02863	0.02708517
0.2464	0.6983108	-0.2753	0.0017	0.03163	0.02961452
0.2894	0.5997991	-0.189	0.0087	0.02875	0.02782289
0.514	0.4115997	-0.3631	0.0066	0.02528	0.0277175
0.2213	0.3083588	-0.2494	-0.0195	0.03146	0.02961452
0.4687	0.5454015	0.8768	0.0122	0.02116	0.02719056
0.0675	0.9628155	0.3373	-0.0594	0.0511	0.07229737
0.2888	0.5997991	-0.1864	0.0087	0.02836	0.02782289
0.5141	0.4115997	-0.3538	0.0066	0.02489	0.0277175
0.3439	0.5965761	-0.1886	-0.0094	0.02672	0.02761212
0.0528	0.98522253	0.4456	-0.0419	0.05532	0.1119239
0.4686	0.4548862	0.9156	0.0121	0.02033	0.02719056
0.0675	0.9628155	0.3615	-0.0594	0.05014	0.07229737
0.2895	0.5997991	-0.2188	0.0087	0.02869	0.02782289
0.5139	0.4115997	-0.4118	0.0066	0.0249	0.0277175
0.3446	0.5965761	-0.1846	-0.0094	0.02711	0.02761212
0.0524	0.98522253	0.5248	-0.0419	0.05593	0.1119239
0.4682	0.4548643	1.011	0.013	0.01936	0.02719056
0.0675	0.9628155	0.4486	-0.0594	0.05056	0.07229737
0.2888	0.5997991	-0.1827	0.0087	0.02838	0.02782289
0.5141	0.4115997	-0.3962	0.0066	0.02472	0.0277175
0.3439	0.5965761	-0.1937	-0.0094	0.02673	0.02761212
0.4687	0.4549361	1.014	0.0114	0.01892	0.02719056
0.0675	0.9628155	0.4759	-0.0594	0.0499	0.07229737
0.9472	0.01481844	-0.5171	0.0582	0.0553	0.1117131
0.6527	0.2266671	0.1739	-0.0007	0.02896	0.03277621
0.0187	0.98374399	0.8979	-0.0521	0.1053	0.1145587
0.0457	0.01499557	0.5908	0.1274	0.06675	0.1160341

0.0884	0.93086251	0.2681	0.0177	0.04886	0.05374877
0.4728	0.4549336	0.4907	0.0114	0.0262	0.02719056
0.5897	0.6909228	0.185	-0.0499	0.03057	0.0319331
0.0887	0.97541987	-0.5202	-0.1282	0.05995	0.09780169
0.0346	0.96480425	-0.7129	0.0568	0.09169	0.07440517
0.0517	0.01403027	-0.5047	-0.0838	0.07979	0.1172988
0.061	0.96862688	-0.607	-0.0636	0.07005	0.08009621
0.1375	0.92517759	-0.472	0.0138	0.04978	0.05195715
0.6486	0.7404184	0.2286	0.0144	0.03604	0.03098459
0.025	0.97486188	-0.7454	0.083	0.1101	0.0894759
0.0875	0.97541987	-0.5131	-0.1282	0.04473	0.09780169
0.0365	0.96480425	-0.7498	0.0568	0.06509	0.07440517
0.0526	0.01403027	-0.5364	-0.0838	0.05734	0.1172988
0.325	0.480596621	-0.1694	0.0078	0.02694	0.027295946
0.0622	0.96862688	-0.5248	-0.0636	0.05105	0.08009621
0.6419	0.7404184	0.218	0.0144	0.0259	0.03098459
0.1407	0.92520665	-0.4367	0.0139	0.036	0.05195715
0.0264	0.97486188	-0.7095	0.083	0.07772	0.0894759
0.0874	0.97541987	-0.5326	-0.1282	0.04476	0.09780169
0.0365	0.96480425	-0.7406	0.0568	0.06518	0.07440517
0.099	0.07958666	-1.496	0.0485	0.03385	0.05058708
0.0526	0.01403027	-0.5338	-0.0838	0.05744	0.1172988
0.3253	0.480596621	-0.1724	0.0078	0.02698	0.027295946
0.0622	0.96862688	-0.5142	-0.0636	0.05113	0.08009621
0.6419	0.7404184	0.2136	0.0144	0.02594	0.03098459
0.1407	0.92520665	-0.4435	0.0139	0.03602	0.05195715
0.0264	0.97486188	-0.7056	0.083	0.07783	0.0894759
0.0874	0.97541987	-0.4922	-0.1282	0.04486	0.09780169
0.0365	0.96480425	-0.7389	0.0568	0.0653	0.07440517
0.0527	0.01403027	-0.4826	-0.0838	0.05757	0.1172988
0.0622	0.96862688	-0.5142	-0.0636	0.05119	0.08009621
0.3342	0.47856234	-0.1522	0.0065	0.02702	0.029614521
0.6418	0.7404184	0.2246	0.0144	0.02597	0.03098459
0.1406	0.92520665	-0.4284	0.0139	0.03608	0.05195715
0.0996	0.08022913	-1.434	0.0391	0.03455	0.05027091
0.0264	0.97486188	-0.7005	0.083	0.0779	0.0894759
0.0873	0.97541987	-0.4717	-0.1282	0.0449	0.09780169
0.0365	0.96480425	-0.6734	0.0568	0.06556	0.07440517
0.0525	0.01403027	-0.4771	-0.0838	0.05763	0.1172988
0.0622	0.96862688	-0.4832	-0.0636	0.05131	0.08009621
0.6419	0.7404184	0.2113	0.0144	0.026	0.03098459
0.1406	0.92520665	-0.4046	0.0139	0.03616	0.05195715
0.099	0.92084717	-1.385	0.0455	0.03524	0.05069247
0.0264	0.97486188	-0.6664	0.083	0.07803	0.0894759
0.0874	0.97541987	-0.5028	-0.1282	0.04481	0.09780169
0.0365	0.96480425	-0.7505	0.0568	0.06509	0.07440517
0.0526	0.01403027	-0.5223	-0.0838	0.05739	0.1172988
0.325	0.480596621	-0.1765	0.0078	0.02694	0.027295946
0.0622	0.96862688	-0.5308	-0.0636	0.05104	0.08009621
0.6418	0.7404184	0.2184	0.0144	0.02591	0.03098459
0.1406	0.92520665	-0.4356	0.0139	0.03602	0.05195715
0.0264	0.97486188	-0.7149	0.083	0.07772	0.0894759
0.0874	0.97541987	-0.4975	-0.1282	0.0448	0.09780169
0.0365	0.96480425	-0.763	0.0568	0.065	0.07440517
0.099	0.07958666	-1.509	0.0485	0.03362	0.05058708
0.0526	0.01403027	-0.5384	-0.0838	0.05731	0.1172988
0.325	0.480596621	-0.1805	0.0078	0.02691	0.027295946
0.0622	0.96862688	-0.532	-0.0636	0.051	0.08009621

0.6418	0.7404184	0.2191	0.0144	0.0259	0.03098459
0.1406	0.92520665	-0.4296	0.0139	0.03601	0.05195715
0.0264	0.97486188	-0.7283	0.083	0.07763	0.0894759
0.0874	0.97541987	-0.5029	-0.1282	0.04491	0.09780169
0.0365	0.96480425	-0.7531	0.0568	0.06516	0.07440517
0.0526	0.01403027	-0.5248	-0.0838	0.05747	0.1172988
0.325	0.480596621	-0.1866	0.0078	0.02696	0.027295946
0.0622	0.96862688	-0.5235	-0.0636	0.05113	0.08009621
0.6418	0.7404184	0.2161	0.0144	0.02596	0.03098459
0.1406	0.92520665	-0.4336	0.0139	0.03609	0.05195715
0.0264	0.97486188	-0.7259	0.083	0.0778	0.0894759
0.0872	0.97541987	-0.5412	-0.1282	0.04473	0.09780169
0.0364	0.96480425	-0.7379	0.0568	0.06516	0.07440517
0.0527	0.01403027	-0.5355	-0.0838	0.05733	0.1172988
0.0621	0.96862688	-0.5121	-0.0636	0.05103	0.08009621
0.6417	0.7404184	0.2107	0.0144	0.02589	0.03098459
0.1407	0.92520665	-0.4487	0.0139	0.03597	0.05195715
0.0263	0.97486188	-0.6949	0.083	0.07785	0.0894759
0.5726	0.3374182	-0.1328	0.0217	0.02309	0.03140615
0.7661	0.742607	-0.4611	-0.0272	0.02546	0.0308792
0.4182	0.5706917	-0.1471	0.0227	0.02426	0.02740134
0.1007	0.8023709	0.4553	0.0139	0.04256	0.03425167
0.514	0.4115997	-0.4069	0.0066	0.02528	0.0277175
0.9692	0.01600679	-0.4298	-0.183	0.07576	0.1078137
0.2213	0.3083588	-0.2549	-0.0195	0.03174	0.02961452
0.4073	0.5029423	-0.2387	0.004	0.02661	0.02719056
0.6348	0.5804156	0.1594	0.0232	0.02725	0.02761212
0.574	0.4948719	-0.9707	-0.0089	0.02069	0.02697978
0.1007	0.8023709	0.4987	0.0139	0.04195	0.03425167
0.5141	0.4115997	-0.4034	0.0066	0.02502	0.0277175
0.0528	0.98522253	0.5578	-0.0419	0.05578	0.1119239
0.4262	0.5052813	1.039	0.008	0.01935	0.02697978
0.3	0.6881858	-0.1833	-0.0352	0.02842	0.02929835
0.0588	0.02573456	0.562	-0.0992	0.05417	0.08821122
0.1007	0.8023709	0.5396	0.0139	0.04083	0.03425167
0.5141	0.4115997	-0.4485	0.0066	0.02417	0.0277175
0.9693	0.01600679	-0.4197	-0.183	0.07322	0.1078137
0.0528	0.98522253	0.6399	-0.0419	0.0542	0.1119239
0.6359	0.5804156	0.2158	0.0232	0.0262	0.02761212
0.0716	0.96151999	0.269	-0.0469	0.0485	0.07040035
0.5738	0.4948475	-1.111	-0.0084	0.01733	0.02697978
0.0588	0.02573456	0.6144	-0.0992	0.05272	0.08821122
0.1009	0.8023709	0.4441	0.0139	0.04007	0.03425167
0.5141	0.4115997	-0.3969	0.0066	0.02378	0.0277175
0.4259	0.5052349	0.9616	0.007	0.01893	0.02697978
0.4068	0.5029423	-0.2419	0.004	0.0251	0.02719056
0.0211	0.01943345	0.6746	-0.0582	0.08639	0.09959332
0.9472	0.01481844	-0.5209	0.0582	0.0533	0.1117131
0.5276	0.5448902	-0.2945	-0.013	0.02665	0.02719056
0.4865	0.2053527	0.2076	-0.0532	0.02905	0.03625407
0.4269	0.4947586	-0.2226	0.0073	0.02713	0.02697978
0.4466	0.605326605	0.5797	0.0108	0.02517	0.027822895
0.0324	0.93209821	-0.4505	-0.0165	0.07604	0.053748774
0.5156	0.4115997	-0.3988	0.0066	0.02725	0.0277175
0.4288	0.5052349	1.015	0.007	0.02097	0.02697978
0.3854	0.3931844	0.2849	0.0076	0.02836	0.03024686
0.4053	0.5029423	-0.2442	0.004	0.02817	0.02719056
0.8479	0.2239949	-0.22	-0.0445	0.03859	0.03256543

0.5156	0.4115997	-0.1597	0.0066	0.02758	0.0277175
0.0187	0.98374399	0.6927	-0.0521	0.1041	0.1145587
0.0457	0.01499557	0.5196	0.1274	0.06591	0.1160341
0.4719	0.5474985	0.5445	0.0168	0.02537	0.02950913
0.5717	0.2393961	0.3005	0.0084	0.02793	0.03172232
0.5156	0.4115997	-0.3837	0.0066	0.0273	0.0277175
0.4053	0.5029423	-0.2329	0.004	0.02819	0.02719056
0.3848	0.6068208	0.2703	0.007	0.02839	0.03024686
0.5709	0.5051715	-1.026	-0.0084	0.02079	0.02697978
0.5153	0.4115997	-0.1936	0.0066	0.02737	0.0277175
0.3851	0.6068208	0.1839	0.007	0.02761	0.03024686
0.5156	0.4115997	-0.4542	0.0066	0.02621	0.0277175
0.3854	0.3931844	0.3052	0.0076	0.02778	0.03024686
0.4053	0.5029423	-0.2642	0.004	0.02755	0.02719056
0.0211	0.01943345	0.569	-0.0582	0.09629	0.09959332
0.4719	0.5474985	1.175	0.0168	0.01665	0.02950913
0.9466	0.01974258	-0.4413	0.0496	0.05908	0.09685318
0.5141	0.4115997	-0.3997	0.0066	0.02377	0.0277175
0.0528	0.98522253	0.5572	-0.0419	0.05308	0.1119239
0.2999	0.6881858	-0.2053	-0.0352	0.02702	0.02929835
0.4686	0.4548874	1.021	0.0123	0.01764	0.02719056
0.0674	0.9628155	0.4246	-0.0594	0.04825	0.07229737
0.1106	0.135951218	0.2178	-0.052	0.03952	0.040048106
0.3258	0.480596621	0.5089	0.0078	0.02554	0.027295946
0.0455	0.01871598	0.3665	-0.0397	0.06056	0.1042305
0.146	0.7315893	0.715	0.0431	0.0334	0.03056303
0.2439	0.7849069	-0.4537	-0.0132	0.03721	0.03267082
0.2082	0.6878651	0.1734	0.0168	0.03172	0.02950913
0.5911	0.6088145	-0.1952	-0.0323	0.02627	0.0277175
0.0044	0.9524443	1.391	0.0334	0.1912	0.06397158
0.0145	0.06456783	0.6084	-0.0294	0.1074	0.05627813
0.2083	0.6878651	0.2281	0.0168	0.02982	0.02950913
0.2082	0.6878651	0.1841	0.0168	0.03172	0.02950913
0.5911	0.6088145	-0.2138	-0.0323	0.02624	0.0277175
0.0044	0.9524443	1.418	0.0334	0.1913	0.06397158
0.0145	0.06456783	0.6318	-0.0294	0.1074	0.05627813
0.552	0.332185099	0.238	0.0556	0.03646	0.028666013
0.3428	0.7678453	0.2608	0.0052	0.03665	0.03477862
0.5475	0.332185099	-0.2555	0.0556	0.02745	0.028666013
0.0323	0.96141674	-0.5632	0.0179	0.07427	0.07788303
0.3055	0.6139303	0.3221	-0.0079	0.02878	0.02792828
0.0816	0.97718644	0.2856	-0.0427	0.04932	0.09548312
0.0124	0.98311064	-0.7822	-0.1058	0.1208	0.1091838
0.0308	0.97488021	-0.4696	0.165	0.07632	0.08620882
0.1086	0.96980236	-1.076	0.013	0.03832	0.07904231
0.022	0.96320915	-0.4567	0.0146	0.08253	0.07261354
0.2581	0.3227183	0.273	0.0324	0.02764	0.02898218
0.1514	0.8949306	0.2106	0.0358	0.03486	0.0442637
0.555	0.5682089	-0.143	-0.0168	0.02522	0.02750673
0.325	0.480596621	-0.2111	0.0078	0.02661	0.027295946
0.6318	0.2481714	-0.4707	-0.0551	0.02507	0.03130076
0.5339	0.4012964	-0.2059	0.0136	0.02456	0.0277175
0.0562	0.02701209	0.6071	-0.0586	0.05356	0.08273096
0.035	0.98073243	0.4158	0.0596	0.06821	0.1021227
0.469	0.5566083	0.1497	0.0549	0.02574	0.02729595
0.0133	0.963834118	0.6796	-0.1283	0.109	0.075459063
0.3268	0.480596621	0.3834	0.0078	0.02582	0.027295946
0.3064	0.2916925	-0.1674	0.0084	0.02775	0.0298253

0.3064	0.2916925	-0.2433	0.0084	0.02779	0.0298253
0.2194	0.7256658	-0.3589	0.0084	0.04157	0.03045764
0.0918	0.98911045	0.4117	0.0849	0.06776	0.1322641
0.2193	0.7256658	-0.4126	0.0084	0.04076	0.03045764
0.7156	0.4798355	-0.2424	0.03	0.04306	0.02961452
0.0917	0.98911045	0.3935	0.0849	0.06679	0.1322641
0.4017	0.7138743	-0.1548	0.029	0.0252	0.02993069
0.3458	0.06052395	-0.1904	0.0311	0.03241	0.0596506
0.1514	0.8949306	0.256	0.0358	0.03444	0.0442637
0.1224	0.95767674	0.2579	-0.0321	0.03725	0.07113808
0.555	0.5682089	-0.1453	-0.0168	0.02494	0.02750673
0.325	0.480596621	-0.2208	0.0078	0.02632	0.027295946
0.6318	0.2481714	-0.5841	-0.0551	0.02417	0.03130076
0.1671	0.7631517	-0.2616	0.0099	0.03299	0.03203848
0.0562	0.02701209	0.7589	-0.0586	0.05246	0.08273096
0.035	0.98073243	0.5011	0.0596	0.06717	0.1021227
0.1136	0.95896223	0.2196	0.0549	0.03939	0.06713327
0.6669	0.2582425	0.1525	0.0121	0.02627	0.03098459
0.1514	0.8949306	0.199	0.0358	0.03488	0.0442637
0.1224	0.95767674	0.2563	-0.0321	0.03761	0.07113808
0.6318	0.2481714	-0.5071	-0.0551	0.0249	0.03130076
0.1671	0.7631517	-0.213	0.0099	0.03344	0.03203848
0.0562	0.02701209	0.6708	-0.0586	0.05333	0.08273096
0.0272	0.9634158	0.4286	0.0998	0.07713	0.07271893
0.0304	0.03540675	-0.4093	0.1531	0.0725	0.07177042
0.035	0.98073243	0.4366	0.0596	0.06803	0.1021227
0.1514	0.8949306	0.2761	0.0358	0.03467	0.0442637
0.0718	0.02406367	0.2781	0.0013	0.0492	0.08873817
0.1224	0.95767674	0.299	-0.0321	0.03747	0.07113808
0.555	0.5682089	-0.1635	-0.0168	0.02512	0.02750673
0.325	0.480596621	-0.2933	0.0078	0.02635	0.027295946
0.6318	0.2481714	-0.5853	-0.0551	0.02439	0.03130076
0.1671	0.7631517	-0.3147	0.0099	0.03313	0.03203848
0.0562	0.02701209	0.6995	-0.0586	0.05313	0.08273096
0.035	0.98073243	0.478	0.0596	0.06777	0.1021227
0.1513	0.8949306	0.1988	0.0358	0.03485	0.0442637
0.0718	0.02406367	0.2712	0.0013	0.04922	0.08873817
0.1224	0.95767674	0.2484	-0.0321	0.03759	0.07113808
0.3251	0.480596621	-0.1586	0.0078	0.02671	0.027295946
0.167	0.7631517	-0.2612	0.0099	0.03338	0.03203848
0.0561	0.02701209	0.7465	-0.0586	0.05306	0.08273096
0.6313	0.2482242	-0.4783	-0.0536	0.02498	0.03130076
0.0272	0.9634158	0.4379	0.0998	0.077	0.07271893
0.035	0.98073243	0.5086	0.0596	0.06804	0.1021227
0.1514	0.8949306	0.2742	0.0358	0.03426	0.0442637
0.1224	0.95767674	0.264	-0.0321	0.03709	0.07113808
0.555	0.5682089	-0.1634	-0.0168	0.02479	0.02750673
0.325	0.480596621	-0.2036	0.0078	0.02626	0.027295946
0.6318	0.2481714	-0.6404	-0.0551	0.02368	0.03130076
0.1671	0.7631517	-0.2122	0.0099	0.03291	0.03203848
0.0562	0.02701209	0.6579	-0.0586	0.05259	0.08273096
0.035	0.98073243	0.4177	0.0596	0.06693	0.1021227
0.0057	0.98063408	1.244	-0.0876	0.2236	0.1009634
0.5925	0.6088145	-0.2555	-0.0323	0.02636	0.0277175
0.0041	0.9524443	1.499	0.0334	0.1979	0.06397158
0.0033	0.97848138	1.586	0.0964	0.2396	0.1012796
0.0149	0.03293785	0.698	0.0715	0.1104	0.07588062
0.5909	0.6088145	0.1345	-0.0323	0.02443	0.0277175

0.0044	0.9524443	-1.081	0.0334	0.1759	0.06397158
0.2969	0.1744765	-0.1902	0.0787	0.02599	0.03541096
0.5907	0.6088145	-0.1861	-0.0323	0.0267	0.0277175
0.0044	0.9524443	1.342	0.0334	0.1929	0.06397158
0.2972	0.1744765	0.2347	0.0787	0.02843	0.03541096
0.0427	0.95329406	0.4352	0.0264	0.06661	0.06355002
0.1266	0.97237729	-0.2839	-0.0557	0.04341	0.09105675
0.228	0.3246714	-0.2551	0.0332	0.03189	0.02919296
0.4762	0.754849	0.1925	-0.0197	0.02804	0.03456784
0.4853	0.5866563	-0.1653	0.0224	0.02755	0.02750673
0.2982	0.7284708	-0.3745	-0.0296	0.02938	0.03045764
0.0844	0.93606052	-1.218	-0.0655	0.04471	0.05480267
0.1975	0.90110612	0.2088	-0.0062	0.03441	0.04942779
0.0637	0.97695845	-0.4161	-0.1528	0.05614	0.09095136
0.0212	0.0153733	-0.5777	0.0456	0.0981	0.1230952
0.0001	0.97968015	26.26	0.0756	4.684	0.1048628
0.0003	0.98514891	23.77	-0.1534	3.62	0.1358474
0.0003	0.03656503	32	-0.0975	3.541	0.08041238
0.0001	0.98489401	30.16	0.0588	4.486	0.1143479
0.0015	0.98731482	9.757	0.0086	1.738	0.1210928
0.0004	0.98279228	24.71	0.0192	3.261	0.1112916
0.0047	0.97838495	5.388	0.0563	0.8953	0.09211064
0.2185	0.704696	1.086	-0.0388	0.1686	0.03246004
0.0072	0.95716267	4.44	0.0664	0.7441	0.06860873
0.0003	0.01711402	19.07	0.0973	3.318	0.105179
0.0004	0.98426147	20.95	0.2047	2.979	0.1110808
0.0057	0.02114181	4.577	0.2215	0.79	0.09896098
0.0003	0.98493582	22.84	0.1169	3.766	0.1210928
0.0003	0.98381268	24.63	-0.028	3.673	0.1136102
0.0001	0.986435563	25.81	-0.0434	4.27	0.118247303
0.001	0.985159205	11.36	-0.0279	1.879	0.113293984
0.3523	0.3818072	1.152	-0.0222	0.1309	0.0277175
0.0001	0.01549095	31.35	-0.1516	4.91	0.114031713
0.0001	0.97660591	52.08	0.1346	8.178	0.09010824
0.0001	0.98036846	29.68	-0.0075	4.936	0.100858
0.0003	0.01996814	20.97	0.1057	3.305	0.09969871
0.0032	0.04135303	6.353	0.1118	1.049	0.07040035
0.0003	0.98489215	18.92	0.139	3.324	0.108973
0.0006	0.97908569	303.3	-0.0439	53.88	0.09390227
0.0025	0.02127788	137.6	-0.0058	24.38	0.09590468
0.0043	0.015378928	123.8	-0.048	19.64	0.120565877
0.0076	0.97699058	87.63	0.0311	15.24	0.09031902
0.0006	0.98861784	345.8	-0.0222	57.03	0.133318
0.0001	0.98489401	591	0.0588	96.85	0.1143479
0.0022	0.982179784	176.7	-0.0223	29.35	0.105600532
0.0003	0.97598111	436	0.0444	75.02	0.0976963
0.0003	0.98817053	450.6	-0.0604	77.86	0.135742
0.0015	0.98731482	213	0.0086	36.73	0.1210928
0.0006	0.02026086	384.3	-0.0884	58.05	0.1006472
0.0022	0.01099178	172.7	0.1607	28.26	0.1316318
0.0075	0.02343719	84.27	0.1624	15.32	0.09042441
0.0025	0.02881138	159.7	-0.0131	26.69	0.08157167
0.0018	0.95529191	201.5	-0.018	32.14	0.06639554
0.0022	0.98266658	192.8	-0.0712	34.06	0.1043359
0.0004	0.0247793	364.8	0.0384	66.04	0.09622084
0.0004	0.98279228	472.2	0.0192	70.2	0.1112916
0.0004	0.01144458	385.7	-0.1674	68.87	0.1301563
0.0035	0.98548787	132.8	-0.0651	20.58	0.1163503

0.0003	0.01358241	462.3	-0.0879	82.97	0.1193012
0.001	0.98735438	232.5	0.0437	42.11	0.139536
0	0.01465189	2185	0.1756	391	0.1122401
0.0015	0.97671832	189.7	0.0245	34.49	0.09653701
0.2188	0.704696	22.81	-0.0388	3.563	0.03246004
0	0.98346473	1678	-0.0058	304.6	0.1099215
0.0004	0.98278539	641.1	-0.0951	67.08	0.1096053
0.0048	0.980979747	111	-0.1716	19.7	0.116034118
0.0001	0.98688365	791.9	0.2462	98.43	0.1428031
0.0007	0.01876459	259.1	-0.0195	45.06	0.103282
0.0147	0.98308721	71.36	0.0321	10.98	0.1031766
0.0028	0.01335061	151.8	0.1092	24.76	0.1234114
0.0004	0.9894948	556.4	0.3367	84.21	0.1426977
0.0009	0.98300647	259.6	0.0479	43.23	0.1061275
0.0003	0.97751619	595.1	-0.1756	71.5	0.09706396
0.001	0.97850794	251.2	0.0999	41.16	0.09590468
0.0001	0.012060681	725.9	0.0626	100.6	0.135952781
0.0026	0.98310219	148.2	0.0563	25.16	0.106549
0.0009	0.01292423	262.3	0.0993	44.92	0.1330019
0.0007	0.98809857	285.3	-0.0303	47.21	0.127627
0.0057	0.02114181	118.8	0.2215	16.87	0.09896098
0.0003	0.98493582	473.4	0.1169	81.22	0.1210928
0.0004	0.9856037	402.2	-0.1604	60.78	0.1120293
0.0001	0.98548952	613.8	0.0572	90.65	0.1168772
0.0001	0.98181837	760.5	-0.064	100.6	0.1041251
0.0003	0.01442422	1005	-0.134	130	0.1283647
0.0003	0.98625613	402.2	0.1004	71.9	0.1230952
0.9991	0.98796374	-397.5	0.0882	42.74	0.1475457
0.0009	0.01377977	256.7	-0.1786	45.57	0.152499
0.0004	0.97847424	548.7	-0.1433	98.8	0.1062329
0.0001	0.986435563	721.2	-0.0434	91.57	0.118247303
0.3526	0.3818072	20.08	-0.0222	2.814	0.0277175
0.0001	0.01549095	644.1	-0.1516	106	0.114031713
0.0006	0.02221245	284.3	0.0989	51.52	0.1020173
0.0007	0.01394672	296.2	0.034	50.18	0.118985
0.0001	0.97660591	1065	0.1346	176.6	0.09010824
0.001	0.98381856	251.6	0.0248	42.22	0.1217252
0.0001	0.98036846	610.4	-0.0075	106.6	0.100858
0.0098	0.96869065	75.05	-0.0412	12.99	0.08620882
0.0007	0.01695547	375.7	0.0911	59.27	0.1080245
0.0022	0.04187151	185.1	0.0645	26.88	0.07113808
0.0003	0.98914133	485.7	-0.0836	72.98	0.1468079
0.0001	0.01575078	591.3	-0.0446	99.63	0.1084461
0.004	0.97202549	123.2	-0.0131	21.68	0.08209862
0.0073	0.9863678	88.88	0.1155	15.94	0.1178257
0.0007	0.98813605	283	-0.2663	46.74	0.1360582
0.0022	0.97767933	186.4	-0.0248	27.77	0.1013849
0.0032	0.04135303	154.5	0.1118	22.53	0.07040035
0.0035	0.042610028	147.3	-0.0659	23.48	0.068608729
0.0016	0.98656904	187	0.0392	33.11	0.1193012
0.0006	0.01118989	285.1	-0.155	50.77	0.1498642
0.0006	0.98538135	379.6	0.146	61.08	0.1115024
0.0072	0.01082888	109	-0.0877	15.71	0.1432247
0.0004	0.02876123	358.8	-0.1535	56.41	0.08958129
0.0013	0.984637461	263.3	0.2675	37.24	0.108340666
0.0125	0.01346555	74.28	-0.0355	11.52	0.1169826
0.0003	0.98398986	389.2	0.0278	70.79	0.1163503
0.0004	0.97215177	397.6	0.0064	71.92	0.08389024

0.0003	0.01949902	514.7	0.0627	82.05	0.1072868
0.0004	0.98680291	368.8	0.0444	66.09	0.1245707
0	0.98530474	1621	-0.2039	252.5	0.1162449
0.034	0.98300627	0.4119	0.1365	0.06902	0.1023334
0.0758	0.97539085	-0.3125	-0.0906	0.04775	0.097169352
0.0453	0.011696572	-0.3497	0.0388	0.06275	0.126994652
0.3264	0.480596621	0.5292	0.0078	0.02548	0.027295946
0.0456	0.01871598	0.4042	-0.0397	0.06064	0.1042305
0.1838	0.8915075	0.189	0.0119	0.0324	0.04753078
0.2737	0.178094739	0.339	0.0253	0.02812	0.038678039
0.1464	0.7315893	0.7089	0.0431	0.03346	0.03056303
0.479	0.529565	0.1981	0.0215	0.02746	0.02708517
0.4574	0.622394	0.1993	-0.0673	0.03174	0.03035225
0.5296	0.4881992	-0.1576	-0.0661	0.02879	0.02708517
0.4856	0.6080001	-0.1549	0.0025	0.02715	0.03509479
0.0772	0.97365801	-0.5454	0.0433	0.06799	0.0894759
0.0566	0.93029537	0.4881	-0.0175	0.07845	0.05417033
0.0733	0.98906866	0.5176	-0.0653	0.07238	0.1501804
0.1939	0.6507084	-1.131	-0.0173	0.03861	0.02856062
0.2579	0.1764607	-0.3642	-0.0004	0.04217	0.03551635
0.0707	0.92082395	-0.4918	0.0123	0.07051	0.05058708
0.0772	0.97365801	-0.5128	0.0433	0.06836	0.0894759
0.0734	0.98906866	0.5046	-0.0653	0.07256	0.1501804
0.1934	0.6507084	-1.115	-0.0173	0.0393	0.02856062
0.258	0.1764607	-0.3351	-0.0004	0.0424	0.03551635
0.0705	0.92082395	-0.5252	0.0123	0.07057	0.05058708
0.0772	0.97365801	0.3963	0.0433	0.06945	0.0894759
0.5441	0.573417	-0.5579	0.0236	0.03593	0.0277175
0.045	0.9834009	-0.3659	0.0375	0.06602	0.1063383
0.2041	0.4449728	-0.1937	0.0005	0.03404	0.02740134
0.4459	0.608089804	0.6185	0.0038	0.02534	0.027822895
0.051	0.100653834	0.4823	-0.0309	0.06189	0.04679305
0.7893	0.686264144	0.1921	-0.0447	0.03327	0.029614521
0.106	0.879144205	0.3184	0.0358	0.04432	0.041945122
0.0296	0.023620203	-0.4992	-0.0093	0.08145	0.090845967
0.1993	0.834777	0.1756	0.0503	0.03165	0.03962655
0.0917	0.91692666	-0.2878	-0.0738	0.04216	0.05016552
0.2425	0.2448006	-0.283	-0.0138	0.0286	0.03161693
0.8737	0.91247	0.2138	0.036	0.03693	0.04900624
0.1124	0.97028055	0.2943	-0.1921	0.05167	0.08937051
0.1993	0.834777	0.1757	0.0503	0.03163	0.03962655
0.0965	0.91845283	-0.2657	-0.0485	0.0417	0.0503763
0.2425	0.2448006	-0.3192	-0.0138	0.02843	0.03161693
0.0972	0.95894409	0.2688	0.0999	0.042	0.06860873
0.8744	0.07925644	0.2058	0.0611	0.03711	0.05100864
0.184	0.04429664	0.2372	-0.0696	0.03691	0.07082191
0.1491	0.8814485	-0.2152	-0.0124	0.03869	0.04183973
0.0457	0.01499557	-0.6486	0.1274	0.06633	0.1160341
0.2147	0.1240099	-0.2986	-0.0213	0.03345	0.04089122
0.4729	0.5452511	-0.7175	0.0122	0.02414	0.02719056
0.0557	0.98403512	-0.4818	-0.2116	0.06227	0.1076029
0.0193	0.971911457	0.4732	0.0845	0.08644	0.082204007
0.0314	0.01522292	0.3984	-0.0498	0.07064	0.1332126
0.1985	0.7401346	0.177	0.0303	0.03027	0.03077381
0.6739	0.4695682	0.1522	-0.0222	0.02556	0.02729595
0.0193	0.971911457	0.5134	0.0845	0.09207	0.082204007
0.1008	0.97891384	0.2343	0.1317	0.04232	0.09337532
0.2107	0.95045462	-0.2166	0.0096	0.03665	0.06449853

0.1886	0.2517236	0.1903	0.0304	0.03241	0.03108998
0.2111	0.95045462	-0.3007	0.0096	0.03814	0.06449853
0.0359	0.02908859	-0.3957	-0.0999	0.07188	0.08252018
0.0332	0.98623927	-0.4296	-0.0587	0.07489	0.115191
0.3234	0.7170177	0.1738	0.0564	0.02982	0.0298253
0.0817	0.96761985	-0.3894	0.063	0.0561	0.08030699
0.8124	0.2339563	-0.4789	-0.0787	0.03416	0.04025889
0.0729	0.9527966	-0.7731	0.1008	0.05178	0.06312846
0.3055	0.6139303	0.2713	-0.0079	0.02873	0.02792828
0.0276	0.02307243	0.6523	-0.0632	0.08226	0.09211064
0.1066	0.96977141	-0.7478	0.0172	0.04098	0.0791477
0.0385	0.96396148	0.4508	-0.0595	0.07013	0.07240276
0.0001	0.98337037	3.436	-0.0833	0.6021	0.1091838
0.0001	0.01170785	3.787	-0.0642	0.6773	0.1281539
0.0001	0.01718125	4.765	0.1456	0.8459	0.102755
0	0.01211493	7.992	0.014	1.404	0.1375336
0.0001	0.01111673	4.272	-0.1049	0.7543	0.1372175
0.0192	0.02907878	0.4024	-0.1512	0.06819	0.08578726
0.0004	0.9766971	1.818	0.0289	0.2979	0.09074058
0	0.02084062	7.82	-0.0261	1.424	0.09632623
0.0001	0.01114357	6.643	-0.1204	1.199	0.1390091
0.0001	0.98145344	3.814	0.0523	0.6612	0.1048628
0.0001	0.012295369	3.783	0.2779	0.6683	0.133001868
0.0001	0.98641582	3.717	0.0325	0.67	0.1217252
0.0001	0.01268386	4.52	0.033	0.7897	0.125203
0.0001	0.9851344	4.11	0.0516	0.7256	0.1157179
0.0001	0.01862649	3.855	-0.1035	0.6805	0.1020173
0.0001	0.01083516	4.877	-0.0446	0.8599	0.1428031
0.0001	0.01052958	3.755	-0.0472	0.6644	0.1406953
0.0001	0.01197512	4.68	-0.0272	0.8111	0.1243599
0.1069	0.97239935	0.2604	-0.0353	0.0461	0.09021363
0.0982	0.91684888	-0.2525	-0.0427	0.04163	0.04974396
0.8737	0.91247	0.2282	0.036	0.03694	0.04900624
0.2323	0.7524537	-0.2404	-0.0091	0.02912	0.03151154
0.0201	0.98853127	0.5373	-0.1768	0.08896	0.1305779
0.611	0.304194	0.167	0.0119	0.02538	0.02971991
0.0201	0.98853127	0.5594	-0.1768	0.089	0.1305779
0.0185	0.01781957	0.5583	0.0207	0.09163	0.1082353
0.0197	0.98875462	0.5079	-0.0336	0.0892	0.1346881
0.2101	0.94805868	-0.1982	0.0004	0.03505	0.06059911
0.052	0.98540978	0.3593	0.0115	0.05776	0.1126616
0.2594	0.1400056	-0.2439	-0.0754	0.03383	0.04405292
0.4581	0.5840964	-0.1525	0.0027	0.02632	0.02750673
0.2774	0.677216374	0.2378	-0.0215	0.03006	0.029192962
0.0044	0.9524443	-1.573	0.0334	0.1855	0.06397158
0.2975	0.1744765	-0.1572	0.0787	0.02752	0.03541096
0.0035	0.97848138	-1.565	0.0964	0.2272	0.1012796
0.0006	0.01047694	-2.923	0.1039	0.4989	0.1404845
0.0543	0.90385129	0.4097	-0.0393	0.05516	0.04616071
0.1525	0.8928413	0.1992	-0.0497	0.03616	0.04394753
0.1281	0.95469981	-0.2422	-0.062	0.04219	0.06470931
0.199	0.8235794	0.5885	-0.0489	0.03315	0.03583252
0.0003	0.97382295	3.334	0.0638	0.5986	0.08294174
0.0044	0.9524443	1.176	0.0334	0.1662	0.06397158
0.2974	0.1744765	0.1631	0.0787	0.02442	0.03541096
0.2664	0.6069461	0.1398	0.0397	0.02509	0.03035225
0.0003	0.98383776	3.341	-0.0953	0.5986	0.1110808
0.0044	0.01937848	1.068	-0.0296	0.1846	0.106549

0.0044	0.01937848	1.189	-0.0296	0.186	0.106549
0.2308	0.128257917	0.2016	0.0678	0.03334	0.043736747
0.0841	0.93606052	-0.3487	-0.0655	0.0505	0.05480267
0.9244	0.95188003	0.3805	-0.0365	0.05405	0.0648147
0.0425	0.98719058	-0.4081	-0.0773	0.07152	0.1202497
0.1687	0.2153354	0.1793	0.0493	0.03256	0.03298699
0.0032	0.01859489	-1.414	-0.1534	0.2277	0.1082353
0.0034	0.97848138	-1.288	0.0964	0.2345	0.1012796
0.0032	0.01859489	-1.226	-0.1534	0.2117	0.1082353
0.0449	0.96366853	0.3476	0.0715	0.05835	0.07229737
0.0312	0.9791012	0.4707	-0.041	0.07114	0.103282
0.1923	0.7476587	0.2019	0.03	0.03063	0.03119537
0.0998	0.08022913	-0.4215	0.0391	0.0399	0.05027091
0.0215	0.01781957	0.7181	0.0207	0.116	0.1082353
0.0224	0.98875462	0.6567	-0.0336	0.1158	0.1346881
0.0205	0.01148618	0.7788	-0.1527	0.1195	0.1301563
0.0185	0.01781957	0.5863	0.0207	0.09251	0.1082353
0.0197	0.98875462	0.539	-0.0336	0.09004	0.1346881
0.0201	0.01148618	0.5582	-0.1527	0.0907	0.1301563
0.0185	0.01781957	0.5404	0.0207	0.0918	0.1082353
0.0197	0.98875462	0.4919	-0.0336	0.08935	0.1346881
0.0201	0.01148618	0.5735	-0.1527	0.08992	0.1301563
0.0185	0.01781957	0.5314	0.0207	0.09132	0.1082353
0.0201	0.01147284	0.5237	-0.164	0.08866	0.1304725
0.0609	0.95486603	0.281	0.0146	0.05096	0.06744944
0.0185	0.01781957	0.5095	0.0207	0.09165	0.1082353
0.0201	0.98854203	0.5195	-0.1458	0.08901	0.1303671
0.0185	0.01781957	0.6788	0.0207	0.09244	0.1082353
0.0197	0.98875462	0.6138	-0.0336	0.09002	0.1346881
0.0201	0.98854203	0.6504	-0.1458	0.08983	0.1303671
0.0185	0.01781957	0.5546	0.0207	0.09212	0.1082353
0.6109	0.304194	0.148	0.0119	0.02557	0.02971991
0.0201	0.01148618	0.5738	-0.1527	0.09026	0.1301563
0.0307	0.93812853	-0.5378	-0.0187	0.09656	0.05796436
0.5197	0.4267604	0.2522	0.0233	0.03266	0.0277175
0.0003	0.03656503	53.93	-0.0975	8.721	0.08041238
0.2185	0.704696	2.545	-0.0388	0.4129	0.03246004
0.0009	0.01294799	28.75	-0.0299	5.206	0.1226737
0.6474	0.3264086	2.095	0.0172	0.3672	0.03140615
0.541	0.4776394	-1.704	0.0393	0.3071	0.02971991
0.3523	0.3818072	3.541	-0.0222	0.3183	0.0277175
0.0022	0.04187151	18.44	0.0645	3.073	0.07113808
0.0016	0.97067529	2.431	0.0864	0.4216	0.08062316
0.0673	0.91676436	-0.5108	0.0616	0.06739	0.04911162
0.0016	0.97067529	2.421	0.0864	0.4204	0.08062316
0.2132	0.6806864	-0.4855	-0.0232	0.03861	0.02919296
0.0673	0.91676436	-0.5045	0.0616	0.06722	0.04911162
0.0173	0.97256145	-0.6777	-0.0651	0.105	0.086524987
0.4466	0.605326605	0.6149	0.0108	0.02529	0.027822895
0.0512	0.100653834	0.3533	-0.0309	0.062	0.04679305
0.0173	0.97256145	-0.6683	-0.0651	0.1039	0.086524987
0.4466	0.605326605	0.6285	0.0108	0.02484	0.027822895
0.0324	0.93209821	-0.4838	-0.0165	0.07619	0.053748774
0.0173	0.97256145	-0.6462	-0.0651	0.1049	0.086524987
0.4466	0.605326605	0.6324	0.0108	0.02507	0.027822895
0.0324	0.93209821	-0.4665	-0.0165	0.07691	0.053748774
0.0173	0.97256145	-0.6177	-0.0651	0.1046	0.086524987
0.4466	0.605326605	0.6184	0.0108	0.02511	0.027822895

0.0324	0.93209821	-0.4927	-0.0165	0.07664	0.053748774
0.2996	0.07723095	-0.1586	-0.1135	0.02793	0.05258949
0.0115	0.01844077	-0.6992	0.2738	0.1209	0.09896098
0.0194	0.98573486	-0.7826	0.0585	0.08987	0.1147694
0.0115	0.01844077	-0.7874	0.2738	0.1197	0.09896098
0.0194	0.98573486	-0.8747	0.0585	0.08908	0.1147694
0.0321	0.96426846	-0.4181	0.004	0.06918	0.07377283
0.0115	0.01844077	-0.912	0.2738	0.1184	0.09896098
0.0321	0.96405962	-0.5334	0	0.06859	0.07366744
0.233	0.75063287	0.1669	0.0117	0.02931	0.031195367
0.0288	0.97381613	-0.4513	-0.0711	0.07246	0.08663038
0.0194	0.98573486	-1.046	0.0585	0.08813	0.1147694
0.0115	0.01844077	-0.8942	0.2738	0.1174	0.09896098
0.0194	0.98573486	-0.9249	0.0585	0.08708	0.1147694
0.0321	0.96426846	-0.4635	0.004	0.06776	0.07377283
0.0115	0.01844077	-0.8972	0.2738	0.1213	0.09896098
0.0289	0.97381613	-0.4696	-0.0711	0.07316	0.08663038
0.0194	0.98573486	-1.065	0.0585	0.08937	0.1147694
0.0322	0.96426846	-0.502	0.004	0.06983	0.07377283
0.1714	0.921286668	0.5518	0.032	0.03171	0.054908061
0.081	0.976083308	-0.254	-0.024	0.04499	0.089897459
0.1714	0.921286668	-0.4468	0.032	0.03195	0.054908061
0.2136	0.7421583	-0.1739	0.0386	0.02973	0.03098459
0.1714	0.921286668	-0.4624	0.032	0.03231	0.054908061
0.1689	0.921286668	0.2574	0.032	0.03268	0.054908061
0.0401	0.96656008	0.3476	-0.0272	0.06205	0.07640757
0.1714	0.921286668	-0.5431	0.032	0.03176	0.054908061
0.081	0.976083308	0.2556	-0.024	0.045	0.089897459
0.1699	0.921286668	0.2927	0.032	0.0332	0.054908061
0.6125	0.428884	0.1407	-0.0144	0.02521	0.02729595
0.0545	0.95433669	0.3865	0.0607	0.05433	0.06565782
0.0275	0.02307243	0.7078	-0.0632	0.07572	0.09211064
0.1067	0.96977141	-0.3205	0.0172	0.03937	0.0791477
0.0378	0.96396148	0.5857	-0.0595	0.06479	0.07240276
0.5684	0.5594995	0.1437	-0.0028	0.02463	0.02740134
0.6125	0.428884	0.1399	-0.0144	0.02533	0.02729595
0.0545	0.95433669	0.3978	0.0607	0.05455	0.06565782
0.0275	0.02307243	0.7354	-0.0632	0.07599	0.09211064
0.1067	0.96977141	-0.3265	0.0172	0.03954	0.0791477
0.0378	0.96396148	0.5985	-0.0595	0.06505	0.07240276
0.1287	0.03671273	-0.2363	0.0194	0.03676	0.07166503
0.1073	0.05372035	0.3718	0.0556	0.04036	0.05954521
0.0545	0.95433669	0.3576	0.0607	0.05416	0.06565782
0.0275	0.02307243	0.675	-0.0632	0.07557	0.09211064
0.1067	0.96977141	-0.3774	0.0172	0.03905	0.0791477
0.0378	0.96396148	0.5505	-0.0595	0.06465	0.07240276
0.5123	0.3279215	0.1378	-0.0449	0.02431	0.03151154
0.0545	0.95433669	0.3253	0.0607	0.05512	0.06565782
0.0275	0.02307243	0.6063	-0.0632	0.07722	0.09211064
0.1067	0.96977141	-0.435	0.0172	0.03955	0.0791477
0.5112	0.4325118	0.148	0.0175	0.02495	0.02740134
0.0377	0.96396148	0.4184	-0.0595	0.06614	0.07240276
0.0548	0.95433669	0.3487	0.0607	0.05419	0.06565782
0.0277	0.02307243	0.6679	-0.0632	0.0755	0.09211064
0.1069	0.96977141	-0.3231	0.0172	0.03929	0.0791477
0.0379	0.96396148	0.5556	-0.0595	0.0646	0.07240276
0.5685	0.5594995	0.1472	-0.0028	0.02455	0.02740134
0.0545	0.95433669	0.3109	0.0607	0.05547	0.06565782

0.0275	0.02307243	0.5685	-0.0632	0.07776	0.09211064
0.1634	0.1747017	-0.1866	0.0649	0.03387	0.03572713
0.1067	0.96977141	-0.4214	0.0172	0.03983	0.0791477
0.5112	0.4325118	0.1488	0.0175	0.0251	0.02740134
0.0377	0.96396148	0.4014	-0.0595	0.06656	0.07240276
0.5271	0.5819941	0.1443	-0.0194	0.02505	0.02761212
0.0548	0.95433669	0.316	0.0607	0.05379	0.06565782
0.0277	0.02307243	0.6264	-0.0632	0.07504	0.09211064
0.1094	0.96980236	-0.3692	0.013	0.0383	0.07904231
0.0379	0.96396148	0.5148	-0.0595	0.06419	0.07240276
0.5122	0.3279215	0.1432	-0.0449	0.02412	0.03151154
0.0545	0.95433669	0.3694	0.0607	0.05436	0.06565782
0.0275	0.02307243	0.7037	-0.0632	0.07581	0.09211064
0.1067	0.96977141	-0.3832	0.0172	0.0392	0.0791477
0.0378	0.96396148	0.564	-0.0595	0.06488	0.07240276
0.2995	0.7284708	-0.2988	-0.0296	0.0284	0.03045764
0.0845	0.93606052	-1.048	-0.0655	0.04423	0.05480267
0.1968	0.90110612	0.229	-0.0062	0.03302	0.04942779
0.0625	0.97695845	-0.3607	-0.1528	0.05457	0.09095136
0.0381	0.977076489	-0.3742	-0.0282	0.06789	0.09369149
0.2447	0.123361277	0.8089	0.0572	0.02792	0.044579865
0.0688	0.976189635	0.3612	0.0148	0.0521	0.087789664
0.3262	0.566992	-0.2069	0.0099	0.02839	0.02750673
0.2223	0.7918039	-0.2373	-0.0234	0.03434	0.03646485
0.0226	0.98456778	0.482	0.0124	0.08487	0.1128724
0.0044	0.9524443	1.128	0.0334	0.1851	0.06397158
0.0317	0.98960096	0.7676	-0.3571	0.07181	0.1570307
0.0263	0.01876155	0.7376	-0.1956	0.07787	0.1021227
0.0666	0.02559553	0.7229	-0.138	0.05006	0.08589265
0.0209	0.97942471	0.4997	0.035	0.08601	0.09263759
0.0653	0.97796134	0.2846	-0.0223	0.0507	0.09421844
0.4267	0.5053871	0.8891	0.0087	0.02025	0.02697978
0.0464	0.98687268	0.6555	-0.0562	0.05812	0.1172988
0.0044	0.9524443	-1.625	0.0334	0.1865	0.06397158
0.2975	0.1744765	-0.1818	0.0787	0.02763	0.03541096
0.0035	0.97848138	-1.514	0.0964	0.2286	0.1012796
0.0006	0.01047694	-2.942	0.1039	0.5018	0.1404845
0.1525	0.8928413	0.1999	-0.0497	0.03637	0.04394753
0.1562	0.1485174	0.2816	0.0037	0.03509	0.03815109
0.0044	0.9524443	-1.078	0.0334	0.1911	0.06397158
0.0543	0.90385129	0.5758	-0.0393	0.05532	0.04616071
0.0045	0.9524443	1.19	0.0334	0.202	0.06397158
0.3455	0.376821	-0.2144	-0.0192	0.02871	0.02782289
0.0044	0.9524443	1.864	0.0334	0.1678	0.06397158
0.0035	0.97848138	1.793	0.0964	0.2061	0.1012796
0.1084	0.92139333	0.209	-0.0315	0.03723	0.05111403
0.4709	0.4787451	0.2946	0.0462	0.02252	0.02697978
0.9748	0.97111705	-0.463	0.0023	0.07456	0.08420641
0.0044	0.9524443	1.718	0.0334	0.1835	0.06397158
0.0317	0.98960096	0.6998	-0.3571	0.0719	0.1570307
0.2866	0.7817408	-0.1612	-0.0078	0.02787	0.0328816
0.2975	0.1744765	0.201	0.0787	0.02719	0.03541096
0.4069	0.5029423	-0.2216	0.004	0.02536	0.02719056
0.0666	0.02559553	0.6853	-0.138	0.05013	0.08589265
0.0295	0.98914066	0.5464	-0.1965	0.07402	0.136269
0.0095	0.98020646	0.7843	0.0874	0.1289	0.1031766
0.0464	0.9735335	0.3811	0.0377	0.05993	0.08705194
0.4267	0.5053871	0.8426	0.0087	0.02078	0.02697978

0.0464	0.98687268	0.6067	-0.0562	0.05819	0.1172988
0.0044	0.9524443	1.303	0.0334	0.1744	0.06397158
0.2977	0.1744765	0.1724	0.0787	0.02569	0.03541096
0.243	0.6731526	0.1726	0.0319	0.02712	0.02908757
0.0731	0.95701402	-0.267	-0.0604	0.04503	0.06818717
0.0044	0.9524443	1.174	0.0334	0.1853	0.06397158
0.8142	0.91833056	-0.2228	-0.04	0.03216	0.04953318
0.4709	0.4787451	0.2086	0.0462	0.02476	0.02697978
0.0044	0.9524443	-1.821	0.0334	0.1663	0.06397158
0.0035	0.97848138	-1.784	0.0964	0.2042	0.1012796
0.1084	0.92139333	-0.2077	-0.0315	0.03673	0.05111403
0.0095	0.98020646	-0.6555	0.0874	0.1174	0.1031766
0.4709	0.4787451	-0.2903	0.0462	0.02227	0.02697978
0.9748	0.97111705	0.4611	0.0023	0.07373	0.08420641
0.0044	0.9524443	-1.201	0.0334	0.1934	0.06397158
0.0034	0.97848138	-1.455	0.0964	0.2371	0.1012796
0.0542	0.90385129	0.3436	-0.0393	0.05734	0.04616071
0.0044	0.9524443	1.307	0.0334	0.1858	0.06397158
0.2977	0.1744765	0.1611	0.0787	0.02726	0.03541096
0.2464	0.4337771	0.1716	0.0408	0.02881	0.02740134
0.0044	0.9524443	1.815	0.0334	0.1783	0.06397158
0.1084	0.92139333	0.218	-0.0315	0.03915	0.05111403
0.4709	0.4787451	0.2886	0.0462	0.02387	0.02697978
0.0044	0.9524443	-1.125	0.0334	0.169	0.06397158
0.0034	0.97848138	-1.228	0.0964	0.2078	0.1012796
0.0044	0.9524443	-1.253	0.0334	0.1905	0.06397158
0.0044	0.9524443	1.608	0.0334	0.1845	0.06397158
0.2875	0.7817408	-0.1566	-0.0078	0.02815	0.0328816
0.2972	0.1744765	0.1912	0.0787	0.02749	0.03541096
0.0263	0.01876155	0.6358	-0.1956	0.0789	0.1021227
0.021	0.97942471	0.5292	0.035	0.08658	0.09263759
0.0095	0.98020646	0.7461	0.0874	0.1294	0.1031766
0.0464	0.9735335	0.3601	0.0377	0.06066	0.08705194
0.0554	0.98403512	0.7212	-0.2116	0.05595	0.1076029
0.0675	0.9628155	0.4336	-0.0594	0.04951	0.07229737
0.046	0.98687268	0.5828	-0.0562	0.05899	0.1172988
0.0044	0.9524443	1.52	0.0334	0.1739	0.06397158
0.0035	0.97848138	1.439	0.0964	0.2131	0.1012796
0.8142	0.91833056	-0.2655	-0.04	0.03044	0.04953318
0.4709	0.4787451	0.2762	0.0462	0.0232	0.02697978
0.0044	0.9524443	-1.201	0.0334	0.1771	0.06397158
0.0049	0.96728229	-0.9923	0.0602	0.1747	0.08157167
0.0044	0.9524443	-1.776	0.0334	0.1774	0.06397158
0.1084	0.92139333	-0.2213	-0.0315	0.03894	0.05111403
0.4709	0.4787451	-0.2879	0.0462	0.02374	0.02697978
0.0044	0.9524443	-1.217	0.0334	0.1857	0.06397158
0.0044	0.9524443	-1.179	0.0334	0.1933	0.06397158
0.0034	0.97848138	-1.518	0.0964	0.2369	0.1012796
0.0542	0.90385129	0.3316	-0.0393	0.05733	0.04616071
0.0045	0.9524443	1.394	0.0334	0.191	0.06397158
0.0036	0.97848138	1.33	0.0964	0.2281	0.1012796
0.0375	0.97944285	0.7147	0.1167	0.07055	0.09527234
0.0246	0.98794316	0.6089	-0.0257	0.08426	0.1389037
0.1871	0.92153759	0.4963	0.1047	0.03241	0.05016552
0.0643	0.05328005	-0.3245	-0.0806	0.05318	0.06186378
0.0689	0.98142263	0.3184	0.0904	0.0526	0.09748552
0.2548	0.216538	0.1762	-0.0475	0.0299	0.0328816
0.5587	0.3493569	-0.1762	0.0136	0.02445	0.02834984

0.2454	0.7849887	-0.4244	-0.014	0.0273	0.03277621
0.7759	0.8096676	-0.1594	-0.043	0.0289	0.03425167
0.5587	0.3493569	-0.1768	0.0136	0.02448	0.02834984
0.2454	0.7849887	-0.4203	-0.014	0.02736	0.03277621
0.7759	0.8096676	-0.1626	-0.043	0.02894	0.03425167
0.5587	0.3493569	-0.1584	0.0136	0.02453	0.02834984
0.2454	0.7849887	-0.4047	-0.014	0.02745	0.03277621
0.7759	0.8096676	-0.167	-0.043	0.02895	0.03425167
0.5587	0.3493569	-0.1524	0.0136	0.02465	0.02834984
0.2454	0.7849887	-0.3962	-0.014	0.0276	0.03277621
0.0005	0.013547986	-3.125	-0.1913	0.5674	0.12383296
0.345	0.376821	0.1424	-0.0192	0.02589	0.02782289
0.2466	0.6983108	-0.2032	0.0017	0.03171	0.02961452
0.4572	0.622394	0.1884	-0.0673	0.03065	0.03035225
0.2466	0.6983108	-0.2062	0.0017	0.03133	0.02961452
0.4719	0.593635	0.2209	-0.0429	0.02974	0.02993069
0.4865	0.2053527	0.2029	-0.0532	0.02967	0.03625407
0.2466	0.6983108	-0.2046	0.0017	0.03153	0.02961452
0.2466	0.6983108	-0.2367	0.0017	0.03149	0.02961452
0.5897	0.6909228	0.1686	-0.0499	0.03009	0.0319331
0.2465	0.6983108	-0.3264	0.0017	0.03137	0.02961452
0.2466	0.6983108	-0.2417	0.0017	0.03133	0.02961452
0.2464	0.6983108	-0.3032	0.0017	0.03145	0.02961452
0.2464	0.6983108	-0.3219	0.0017	0.03112	0.02961452
0.2467	0.6983108	-0.1847	0.0017	0.03074	0.02961452
0.2466	0.6983108	-0.2479	0.0017	0.03131	0.02961452
0.5897	0.6909228	0.188	-0.0499	0.02994	0.0319331
0.0133	0.963834118	0.8426	-0.1283	0.1098	0.075459063
0.0478	0.924670903	-0.3359	0.0724	0.05863	0.05121942
0.3259	0.480596621	0.429	0.0078	0.02588	0.027295946
0.011	0.02117985	-0.6409	-0.1602	0.1167	0.09906637
0.0133	0.963834118	0.8229	-0.1283	0.1104	0.075459063
0.0478	0.924670903	-0.3227	0.0724	0.05896	0.05121942
0.3259	0.480596621	0.3944	0.0078	0.02617	0.027295946
0.011	0.02117985	-0.6492	-0.1602	0.1176	0.09906637
0.7744	0.7342165	-0.2989	0.0022	0.02511	0.03077381
0.0077	0.97445843	0.8507	0.1022	0.1494	0.09063519
0.6109	0.304194	0.1545	0.0119	0.02548	0.02971991
0.0201	0.01148703	0.5474	-0.1461	0.08918	0.1303671
0.611	0.304194	0.1475	0.0119	0.02551	0.02971991
0.0201	0.01148703	0.5116	-0.1461	0.08936	0.1303671
0.1398	0.7761381	-0.291	-0.006	0.03898	0.03267082
0.1367	0.7371554	0.2325	0.0349	0.04076	0.03077381
0.5014	0.6106737	-0.294	-0.0275	0.03041	0.03014147
0.0041	0.98508444	1.147	-0.03	0.201	0.1301563
0.4847	0.5324209	-0.1917	-0.0088	0.02672	0.02719056
0.592	0.6146481	-0.1359	0.0178	0.02451	0.02792828
0.3256	0.06073939	0.2221	-0.0146	0.0262	0.05564579
0.592	0.6146481	-0.1491	0.0178	0.02489	0.02792828
0.5715	0.4758682	-0.1437	0.0157	0.02517	0.02729595
0.5044	0.2386445	0.1567	0.0016	0.02479	0.03172232
0.475	0.4846681	-0.1428	0.0237	0.02416	0.02719056
0.3256	0.06073939	0.2335	-0.0146	0.02632	0.05564579
0.4882	0.5293066	-0.1468	0.0303	0.0236	0.02719056
0.3257	0.06073939	0.1898	-0.0146	0.02599	0.05564579
0.4903	0.4884768	-0.1352	0.034	0.02397	0.02719056
0.3256	0.06073939	0.2114	-0.0146	0.02614	0.05564579
0.0662	0.95753497	0.2839	-0.0255	0.05066	0.07324588

0.2069	0.2598781	0.1644	-0.0525	0.03007	0.03151154
0.5715	0.4761836	-0.1408	0.0222	0.02438	0.02856062
0.0232	0.01654435	0.6413	0.049	0.08914	0.1148748
0.1737	0.8492428	-0.2143	0.0577	0.0366	0.03836187
0.4102	0.6033231	0.1525	0.0306	0.02762	0.02782289
0.0343	0.02714758	-0.5014	0.0055	0.06723	0.08515492
0.0468	0.97288166	-0.3738	-0.0227	0.05926	0.08736811
0.0463	0.98448718	-0.3468	0.0587	0.0588	0.1138209
0.099	0.07958666	-1.492	0.0485	0.0339	0.05058708
0.0756	0.98609609	-0.574	-0.0128	0.04773	0.1188796
0.1416	0.7905467	-0.3402	0.0076	0.03585	0.04268285
0.0871	0.9756463	-0.6147	-0.1068	0.04478	0.09632623
0.0343	0.02714758	-0.5069	0.0055	0.06712	0.08515492
0.0468	0.97288166	-0.3667	-0.0227	0.0592	0.08736811
0.0756	0.98609609	-0.5694	-0.0128	0.04769	0.1188796
0.1416	0.7905467	-0.3357	0.0076	0.03582	0.04268285
0.0871	0.9756463	-0.6016	-0.1068	0.04478	0.09632623
0.0343	0.02714758	-0.4887	0.0055	0.06701	0.08515492
0.0463	0.98441591	-0.3333	0.0354	0.05866	0.1246761
0.1416	0.7905467	-0.3562	0.0076	0.03572	0.04268285
0.0871	0.9756463	-0.6648	-0.1068	0.04459	0.09632623
0.0762	0.97227924	-0.6176	0.0513	0.04729	0.08273096
0.0343	0.02714758	-0.5	0.0055	0.06715	0.08515492
0.0468	0.97288166	-0.3847	-0.0227	0.05918	0.08736811
0.0756	0.98609609	-0.5628	-0.0128	0.04773	0.1188796
0.1416	0.7905467	-0.3298	0.0076	0.03584	0.04268285
0.0871	0.9756463	-0.6019	-0.1068	0.04479	0.09632623
0.0343	0.02714758	-0.4893	0.0055	0.06729	0.08515492
0.0468	0.97288166	-0.3673	-0.0227	0.05933	0.08736811
0.0463	0.98448718	-0.3347	0.0587	0.05887	0.1138209
0.0756	0.98609609	-0.5915	-0.0128	0.04776	0.1188796
0.1416	0.7905467	-0.3514	0.0076	0.03586	0.04268285
0.3344	0.47856234	-0.1521	0.0065	0.02702	0.029614521
0.0871	0.9756463	-0.6396	-0.1068	0.04477	0.09632623
0.099	0.92082617	-1.465	0.0444	0.03426	0.05069247
0.0343	0.02714758	-0.503	0.0055	0.06729	0.08515492
0.0463	0.98441591	-0.3452	0.0354	0.05888	0.1246761
0.1416	0.7905467	-0.3391	0.0076	0.0359	0.04268285
0.3344	0.47856234	-0.1739	0.0065	0.02699	0.029614521
0.0871	0.9756463	-0.6019	-0.1068	0.04492	0.09632623
0.0762	0.97227924	-0.5742	0.0513	0.04755	0.08273096
0.0273	0.02307243	0.4819	-0.0632	0.07946	0.09211064
0.2827	0.371674	-0.1604	-0.0233	0.02829	0.02824445
0.1343	0.08687124	-0.2225	0.0862	0.0377	0.04763617
0.0175	0.03282322	0.5503	0.1435	0.09653	0.07493211
0.3258	0.480596621	0.4371	0.0078	0.02569	0.027295946
0.1835	0.1982917	0.1864	0.0275	0.03172	0.0339355
0.146	0.7315893	0.6636	0.0431	0.03341	0.03056303
0.0066	0.0134038	-0.8348	-0.1384	0.1494	0.1203551
0.0048	0.02385227	-1.009	-0.1832	0.1845	0.09147831
0.1839	0.95956166	0.1995	-0.0805	0.03634	0.07429978
0.2425	0.2448006	-0.2424	-0.0138	0.02879	0.03161693
0.0972	0.95894409	0.2328	0.0999	0.04226	0.06860873
0.0971	0.91284558	-0.255	-0.0654	0.04098	0.04890085
0.1983	0.8238965	-0.4077	-0.0218	0.03293	0.03562174
0.1976	0.176266	-0.3545	-0.0188	0.0323	0.03562174
0.0449	0.95304762	0.4455	0.0225	0.06505	0.06333924
0.4387	0.3615601	0.1619	0.0248	0.0273	0.02845523

0.0365	0.96480425	-0.731	0.0568	0.06518	0.07440517
0.0648	0.96858726	-0.322	0.1968	0.05089	0.0760914
0.0409	0.03361877	-0.3511	-0.0773	0.06406	0.07862076
0.1053	0.91926911	-1.396	0.0444	0.03364	0.05006013
0.0321	0.02077387	0.4285	0.0454	0.07737	0.1038089
0.0387	0.96043839	0.4289	-0.0805	0.06851	0.06966263
0.0317	0.98960096	0.6769	-0.3571	0.07397	0.1570307
0.0462	0.01499557	0.8332	0.1274	0.06081	0.1160341
0.0667	0.02559553	0.6237	-0.138	0.0518	0.08589265
0.4688	0.5454178	1.01	0.0122	0.01915	0.02719056
0.0483	0.9261867	0.6459	-0.1307	0.05873	0.05332721
0.0317	0.98960096	0.6977	-0.3571	0.07403	0.1570307
0.0462	0.01499557	0.8186	0.1274	0.06103	0.1160341
0.0667	0.02559553	0.6402	-0.138	0.05184	0.08589265
0.383	0.6068208	0.2205	0.007	0.02662	0.03024686
0.5314	0.5450437	-0.9998	-0.0119	0.01941	0.02719056
0.0483	0.9261867	0.6238	-0.1307	0.05887	0.05332721
0.0317	0.98960096	0.7156	-0.3571	0.07506	0.1570307
0.0461	0.01499557	0.8528	0.1274	0.06178	0.1160341
0.3841	0.3931844	0.2118	0.0076	0.0271	0.03024686
0.0668	0.02559553	0.6424	-0.138	0.05263	0.08589265
0.0481	0.9261867	0.6785	-0.1307	0.05954	0.05332721
0.4689	0.4548542	1.01	0.0116	0.01969	0.02719056
0.4305	0.446061027	-0.1551	0.0049	0.02782	0.027401336
0.2402	0.123361277	-0.5493	0.0572	0.03044	0.044579865
0.0029	0.97951509	-1.619	0.0393	0.1943	0.09832864
0.9971	0.96582227	1.154	-0.1257	0.2032	0.07398361
0.0003	0.01968076	3.098	0.0941	0.5579	0.09811786
0.0029	0.97951509	1.755	0.0393	0.1946	0.09832864
0.9971	0.96582227	-1.139	-0.1257	0.2039	0.07398361
0.0929	0.91409653	-0.1685	0.0412	0.03056	0.04774156
0.5109	0.4110804	0.1259	-0.0288	0.021	0.02971991
0.001	0.97735715	2.135	0.0406	0.3629	0.09453461
0.4999	0.2386445	0.1363	0.0016	0.02361	0.03172232
0.5685	0.5594995	0.1693	-0.0028	0.0237	0.02740134
0.001	0.97735715	-2.093	0.0406	0.3644	0.09453461
0.319	0.06073939	-0.1456	-0.0146	0.02572	0.05564579
0.0354	0.988271966	-0.4163	-0.1162	0.06609	0.130894073
0.1634	0.1747017	0.1822	0.0649	0.03225	0.03572713
0.5685	0.5594995	-0.1909	-0.0028	0.02375	0.02740134
0.0704	0.01536781	0.4454	-0.165	0.05597	0.1146641
0.0798	0.93606052	-0.315	-0.0655	0.04587	0.05480267
0.072	0.01536781	-0.4695	-0.165	0.0721	0.1146641
0.2237	0.3500874	-0.4306	-0.0154	0.03825	0.02845523
0.3892	0.6642629	-0.2405	-0.0366	0.03772	0.03098459
0.0704	0.01536781	0.4573	-0.165	0.0559	0.1146641
0.0798	0.93606052	-0.3123	-0.0655	0.04579	0.05480267
0.2561	0.275401506	0.1927	0.0308	0.02975	0.030457639
0.1758	0.141248699	0.3455	-0.0676	0.03444	0.039204988
0.1462	0.1524209	0.209	0.0439	0.03794	0.04152356
0.0154	0.045457836	-0.7279	-0.0047	0.1101	0.065657816
0.1057	0.879144205	0.3619	0.0358	0.04392	0.041945122
0.5197	0.340822483	0.1538	0.0409	0.02746	0.036043295
0.1227	0.105340973	-0.2306	0.0009	0.04135	0.043525968
0.0031	0.02920255	-1.355	0.0084	0.247	0.08304713
0.1961	0.90839607	0.1883	-0.0102	0.03418	0.04679305
0.0512	0.100653834	0.4334	-0.0309	0.06029	0.04679305
0.0559	0.94388769	-0.4459	-0.0357	0.05365	0.0596506

0.1635	0.05202141	0.2065	-0.0018	0.03765	0.06492009
0.8197	0.1720222	0.2121	-0.0017	0.03182	0.0360433
0.6079	0.4734739	-0.1808	0.0146	0.02489	0.02950913
0.7669	0.7522896	0.1996	0.0104	0.0293	0.03151154
0.8197	0.1720222	0.2233	-0.0017	0.03173	0.0360433
0.2303	0.246139	-0.1786	-0.0117	0.02943	0.03435706
0.6088	0.4717883	-0.2011	0.0168	0.02485	0.02719056
0.3162	0.7159645	0.1521	0.0285	0.02688	0.03024686
0.2216	0.811438	-0.1657	0.0433	0.02956	0.0349894
0.074	0.97668284	0.4479	0.1062	0.04714	0.08873817
0.6084	0.4680248	-0.293	-0.0163	0.02511	0.02729595
0.5233	0.5199509	-0.1378	0.0069	0.0244	0.02719056
0.7148	0.3600622	0.2704	-0.012	0.04205	0.02824445
0.2094	0.6806864	-0.9497	-0.0232	0.0394	0.02919296
0.2702	0.1764607	-0.3103	-0.0004	0.04316	0.03551635
0.5574	0.5366913	0.2681	0.003	0.03799	0.02961452
0.3892	0.2056222	-0.1558	-0.0138	0.02531	0.03361933
0.8211	0.8228947	0.187	0.0247	0.03209	0.03920499
0.0967	0.91845283	-0.2571	-0.0485	0.04166	0.0503763
0.2303	0.246139	-0.1948	-0.0117	0.02926	0.03435706
0.2521	0.2474684	0.2057	0.014	0.03039	0.03140615
0.4556	0.403856826	0.3893	-0.0046	0.02598	0.027717505
0.2279	0.735701	-0.378	-0.0008	0.03133	0.03077381
0.2402	0.123361277	-0.4762	0.0572	0.03104	0.044579865
0.5436	0.7418202	-0.1801	0.0499	0.02926	0.03330316
0.1067	0.7385435	0.2608	0.0155	0.04744	0.0339355
0.6062	0.1451289	-0.2999	-0.0519	0.02977	0.04078583
0.7204	0.6046474	0.1984	-0.0253	0.03266	0.03014147
0.1152	0.96980573	-0.2799	-0.186	0.04891	0.08852739
0.1642	0.765104	-0.3127	0.0241	0.04043	0.03509479
0.3759	0.6019412	0.1591	0.0257	0.02774	0.02761212
0.463	0.4818525	0.192	-0.0416	0.02696	0.02729595
0.1269	0.97379986	-0.3411	0.0387	0.04595	0.09274298
0.2476	0.4369519	-0.5168	0.0328	0.02954	0.02740134
0.2255	0.91055998	0.1861	-0.0062	0.03366	0.05079786
0.2125	0.01349708	0.3015	0.017	0.03386	0.1150856
0.0277	0.98025025	-0.6824	0.0742	0.08527	0.09906637
0.8374	0.1088978	-0.2078	0.0717	0.03753	0.04405292
0.3354	0.3898166	-0.2231	0.0012	0.02895	0.0277175
0.0277	0.97949554	-0.5258	0.0496	0.08507	0.09853942
0.1973	0.90110612	0.2079	-0.0062	0.0346	0.04942779
0.2969	0.1744765	-0.1632	0.0787	0.02646	0.03541096
0.2969	0.1744765	0.1603	0.0787	0.0281	0.03541096
0.2966	0.1744765	0.183	0.0787	0.02834	0.03541096
0.2969	0.1744765	0.1457	0.0787	0.02548	0.03541096
0.0705	0.9775227	0.3212	0.1242	0.04962	0.09084597
0.0457	0.01499557	0.4215	0.1274	0.06475	0.1160341
0.4733	0.4546282	0.4156	0.0115	0.02562	0.02719056
0.4777	0.2284913	0.1537	-0.0153	0.02404	0.03214387
0.3256	0.06073939	0.1963	-0.0146	0.02601	0.05564579
0.3256	0.06073939	0.1714	-0.0146	0.02663	0.05564579
0.3256	0.06073939	0.1827	-0.0146	0.02625	0.05564579
0.6359	0.7526132	-0.1525	-0.0579	0.02629	0.03140615
0.3195	0.06073939	-0.2201	-0.0146	0.02651	0.05564579
0.597	0.3611831	0.1572	0.0186	0.02469	0.02834984
0.3256	0.06073939	0.1691	-0.0146	0.02653	0.05564579
0.3256	0.06073939	0.173	-0.0146	0.02661	0.05564579
0.319	0.06073939	0.2272	-0.0146	0.02633	0.05564579

0.4842	0.5124104	-0.1493	0.0375	0.02419	0.02961452
0.3256	0.06073939	0.1523	-0.0146	0.02639	0.05564579
0.2038	0.4449728	-0.2044	0.0005	0.03371	0.02740134
0.4466	0.605326605	0.6441	0.0108	0.02485	0.027822895
0.0512	0.100653834	0.5386	-0.0309	0.06087	0.04679305
0.1024	0.893214404	0.3132	0.0252	0.04432	0.044263696
0.2093	0.8914494	0.2304	-0.0409	0.03386	0.04352597
0.2826	0.371674	-0.1568	-0.0233	0.0284	0.02824445
0.5219	0.6096652	0.1662	-0.0174	0.02565	0.02782289
0.2757	0.9303338	0.2531	-0.0624	0.03508	0.05828053
0.1792	0.1684372	0.1837	0.0286	0.03302	0.03646485
0.3011	0.1687823	0.246	0.09	0.02696	0.03583252
0.0732	0.95701402	-0.3142	-0.0604	0.04765	0.06818717
0.0468	0.97288166	-0.3797	-0.0227	0.05894	0.08736811
0.0463	0.98441591	-0.3313	0.0354	0.05854	0.1246761
0.0756	0.98609609	-0.5638	-0.0128	0.04748	0.1188796
0.0261	0.97891546	-0.725	0.1455	0.07793	0.09716935
0.3344	0.47856234	-0.1503	0.0065	0.02684	0.029614521
0.0871	0.9756463	-0.5779	-0.1068	0.04464	0.09632623
0.099	0.92084717	-1.439	0.0455	0.03429	0.05069247
0.0468	0.97288166	-0.355	-0.0227	0.05912	0.08736811
0.0463	0.98441591	-0.3245	0.0354	0.05867	0.1246761
0.0756	0.98609609	-0.5779	-0.0128	0.0476	0.1188796
0.0261	0.97891546	-0.7252	0.1455	0.07811	0.09716935
0.3344	0.47856234	-0.1479	0.0065	0.02692	0.029614521
0.0871	0.9756463	-0.6004	-0.1068	0.04473	0.09632623
0.099	0.92084717	-1.427	0.0455	0.03453	0.05069247
0.1101	0.1408396	-0.2835	0.045	0.04291	0.03920499
0.3265	0.5669823	-0.182	0.0098	0.02858	0.02750673
0.2366	0.784688702	0.1855	0.0081	0.03217	0.033197772
0.0315	0.9791012	-0.3968	-0.041	0.07123	0.103282
0.4173	0.4304709	-0.1511	0.0224	0.02575	0.02729595
0.766	0.742607	-0.2926	-0.0272	0.02777	0.0308792
0.199	0.1739004	-0.4805	-0.0212	0.03356	0.03583252
0.1779	0.9290425	0.1962	0.0486	0.03583	0.05258949
0.3781	0.5521445	0.1517	0.0274	0.02476	0.02750673
0.5236	0.5199127	-0.1487	0.0066	0.02438	0.02719056
0.1941	0.90110612	-0.1822	-0.0062	0.02999	0.04942779
0.0035	0.97848138	-1.347	0.0964	0.2241	0.1012796
0.0029	0.97951509	-1.532	0.0393	0.2241	0.09832864
0.9748	0.97111705	0.4628	0.0023	0.08043	0.08420641
0.0523	0.98540978	0.3794	0.0115	0.05758	0.1126616
0.223	0.90611356	-0.201	-0.0677	0.03417	0.05006013
0.2446	0.98567273	-0.2671	-0.0953	0.03579	0.1222521
0.7804	0.1392854	0.2205	-0.1895	0.03527	0.04226129
0.0315	0.01522292	-0.4403	-0.0498	0.07983	0.1332126
0.1973	0.8255325	-0.4347	-0.0091	0.03405	0.03878343
0.0389	0.96043839	-0.4661	-0.0805	0.07094	0.06966263
0.0312	0.01522292	0.4291	-0.0498	0.07557	0.1332126
0.189	0.2517236	0.2159	0.0304	0.03275	0.03108998
0.6741	0.4695682	0.1701	-0.0222	0.02733	0.02729595
0.0315	0.01522292	0.415	-0.0498	0.06958	0.1332126
0.0378	0.96396148	0.3543	-0.0595	0.06302	0.07240276
0.6738	0.4695682	0.1408	-0.0222	0.02517	0.02729595
0.0315	0.01522292	0.5228	-0.0498	0.07237	0.1332126
0.0378	0.96043839	0.471	-0.0805	0.06515	0.06966263
0.0315	0.01522292	0.4569	-0.0498	0.0704	0.1332126
0.0378	0.96396148	0.3685	-0.0595	0.0638	0.07240276

0.0316	0.01522292	0.4624	-0.0498	0.07284	0.1332126
0.0328	0.96442635	0.4706	0.0361	0.07011	0.07482672
0.5116	0.4325118	0.1387	0.0175	0.02493	0.02740134
0.1096	0.96878866	-0.4086	0.0429	0.03904	0.07735608
0.5123	0.3279215	0.1457	-0.0449	0.02472	0.03151154
0.0316	0.01522292	0.4806	-0.0498	0.07016	0.1332126
0.0379	0.96396148	0.425	-0.0595	0.06368	0.07240276
0.5122	0.3279215	0.1339	-0.0449	0.02395	0.03151154
0.0315	0.01522292	0.5664	-0.0498	0.07147	0.1332126
0.0378	0.96043839	0.4888	-0.0805	0.06448	0.06966263
0.0368	0.98573072	-0.3567	0.0554	0.06295	0.1174042
0.0814	0.92393594	-0.2576	0.0352	0.04422	0.05100864
0.0482	0.01544514	-0.4589	0.0338	0.07926	0.1154018
0.0762	0.98609609	-0.5625	-0.0128	0.06459	0.1188796
0.025	0.97891546	-0.6961	0.1455	0.1109	0.09716935
0.0887	0.9756463	-0.6208	-0.1068	0.05961	0.09632623
0.0944	0.92084717	-1.495	0.0455	0.04742	0.05069247
0.1016	0.98660943	0.5509	0.059	0.05917	0.1261515
0.1622	0.7860275	0.2745	0.0343	0.04858	0.03340855
0.4036	0.5125463	-0.2587	-0.0244	0.036	0.02961452
0.5545	0.6013043	0.2107	0.027	0.03686	0.03014147
0.0479	0.015563395	-0.5867	-0.0777	0.06432	0.114453272
0.4422	0.35907	0.2265	0.0111	0.02793	0.02824445
0.8964	0.05442347	0.4659	0.003	0.04526	0.05975599
0.0481	0.015563395	-1.147	-0.0777	0.06195	0.114453272
0.016	0.026128823	-0.5868	0.0519	0.1067	0.084417192
0.8956	0.05496869	0.6619	0.0114	0.04462	0.05954521
0.1204	0.045166011	-0.35	-0.0945	0.04248	0.070294965
0.0126	0.98194135	-0.7914	0.0507	0.1209	0.100752604
0.0479	0.015563395	-0.5725	-0.0777	0.0642	0.114453272
0.4422	0.35907	0.1995	0.0111	0.02793	0.02824445
0.8955	0.05965993	0.4055	0.0014	0.04516	0.05733203
0.2457	0.123361277	0.3323	0.0572	0.0314	0.044579865
0.0368	0.01445622	-0.3597	0.0742	0.06402	0.1161395
0.0864	0.94092196	0.2696	0.0554	0.04674	0.05891287
0.657	0.7883355	-0.1537	0.0037	0.02772	0.03330316
0.0096	0.95561488	-0.7492	-0.0869	0.1315	0.06650093
0.0572	0.97626834	-0.3287	0.1203	0.05796	0.08863278
0.0573	0.97626834	-0.3341	0.1203	0.05919	0.08863278
0.245	0.7425922	-0.1851	0.0127	0.03237	0.03108998
0.4102	0.6033231	0.1642	0.0306	0.02772	0.02782289
0.0378	0.01050977	-0.4036	0.1083	0.07353	0.1268893
0.0094	0.986539893	0.8211	0.2271	0.1303	0.111186189
0.0438	0.011696572	-0.3791	0.0388	0.06661	0.126994652
0.2449	0.123361277	0.8094	0.0572	0.02787	0.044579865
0.0686	0.976189635	0.401	0.0148	0.05202	0.087789664
0.2142	0.95372751	0.2427	-0.0485	0.03394	0.07271893
0.4183	0.3115299	-0.4395	-0.0062	0.0274	0.02940374
0.2142	0.95372751	0.2439	-0.0485	0.03399	0.07271893
0.4183	0.3115299	-0.4405	-0.0062	0.02745	0.02940374
0.0798	0.93606052	0.3084	-0.0655	0.0448	0.05480267
0.1941	0.90110612	-0.1786	-0.0062	0.03068	0.04942779
0.2589	0.3216039	0.1655	0.0217	0.02824	0.02908757
0.2848	0.90704723	-0.2623	-0.0268	0.03321	0.05111403
0.1551	0.97088922	0.4143	-0.0649	0.039	0.08452258
0.0495	0.96806773	-0.3331	0.062	0.06077	0.07524828
0.1078	0.8953978	0.2272	-0.0005	0.04123	0.04447448
0.0415	0.95205874	0.4239	-0.0813	0.06144	0.06439314

0.776	0.8096676	-0.1654	-0.043	0.0295	0.03425167
0.1077	0.05372035	0.4838	0.0556	0.04036	0.05954521
0.0414	0.95205874	0.4191	-0.0813	0.06162	0.06439314
0.0062	0.98765616	-0.9269	0.0017	0.1592	0.1286809
0.1075	0.94504495	0.5433	0.0502	0.04021	0.05891287
0.0416	0.95205874	0.3795	-0.0813	0.0615	0.06439314
0.1076	0.05372035	0.476	0.0556	0.04039	0.05954521
0.0414	0.95205874	0.4465	-0.0813	0.0617	0.06439314
0.0997	0.03251896	0.2314	0.0069	0.04201	0.07893692
0.7759	0.8096676	-0.1953	-0.043	0.02956	0.03425167
0.1075	0.05372035	0.5626	0.0556	0.04021	0.05954521
0.0414	0.95205874	0.4264	-0.0813	0.06126	0.06439314
0.0997	0.03251896	0.2334	0.0069	0.04168	0.07893692
0.7759	0.8096676	-0.1898	-0.043	0.02934	0.03425167
0.1075	0.94504495	0.5847	0.0502	0.03976	0.05891287
0.0405	0.928591483	-0.3505	0.0408	0.06354	0.052589487
0.2456	0.123361277	-0.8542	0.0572	0.02593	0.044579865
0.0702	0.976189635	-0.3755	0.0148	0.0488	0.087789664
0.0434	0.95306675	0.3722	0.0222	0.06802	0.06333924
0.0277	0.982385	-0.7524	-0.1114	0.08106	0.1028604
0.1974	0.90110612	0.3156	-0.0062	0.03411	0.04942779
0.0188	0.96625957	-0.6814	0.1079	0.1013	0.07240276
0.1078	0.01592119	0.2974	-0.0561	0.04937	0.123833
0.0437	0.011696572	-0.3975	0.0388	0.06549	0.126994652
0.0095	0.986995687	0.8012	0.2067	0.1303	0.112556256
0.2447	0.123361277	0.8232	0.0572	0.02725	0.044579865
0.0688	0.976189635	0.4169	0.0148	0.05126	0.087789664
0.0536	0.02831551	-0.3114	-0.0705	0.0547	0.08157167
0.4057	0.4135887	0.1467	-0.0045	0.02525	0.02750673
0.0991	0.92084717	-0.5288	0.0455	0.04055	0.05069247
0.8748	0.07937202	0.2082	0.0581	0.03692	0.05100864
0.2425	0.2448006	-0.2125	-0.0138	0.0287	0.03161693
0.8748	0.07937202	0.2257	0.0581	0.03693	0.05100864
0.0965	0.91845283	-0.2717	-0.0485	0.04169	0.0503763
0.2304	0.246139	-0.2717	-0.0117	0.02911	0.03435706
0.1025	0.02699745	0.2896	-0.1839	0.05014	0.09369149
0.1766	0.1340879	0.2422	-0.0213	0.0339	0.03983733
0.1155	0.8991515	0.2173	0.0261	0.03926	0.04468526
0.1766	0.1342725	0.2054	-0.0208	0.03486	0.03973194
0.3258	0.5704662	-0.2745	0.0071	0.02859	0.03003608
0.0407	0.03775643	0.683	0.0803	0.06832	0.07082191
0.0979	0.92212349	0.2565	0.0218	0.04671	0.05027091
0.017	0.97209325	0.5993	-0.069	0.1066	0.08557648
0.3258	0.5704662	-0.209	0.0071	0.02852	0.03003608
0.0407	0.03775643	0.3833	0.0803	0.06868	0.07082191
0.3265	0.5669823	-0.1614	0.0098	0.02865	0.02750673
0.2635	0.3216039	-0.2533	0.0217	0.03729	0.02908757
0.2659	0.8319775	-0.2007	0.0325	0.03572	0.0360433
0.2128	0.6806864	-0.2687	-0.0232	0.03761	0.02919296
0.029	0.03967256	-0.5186	0.0051	0.08634	0.07588062
0.8223	0.4004856	0.3643	-0.0152	0.03754	0.03024686
0.1384	0.1588857	-0.2979	0.0342	0.03961	0.03688641
0.4579	0.404382487	0.3265	-0.0035	0.02759	0.027717505
0.4556	0.403856826	0.4384	-0.0046	0.02585	0.027717505
0.4562	0.403856826	0.4235	-0.0046	0.02586	0.027717505
0.1062	0.879144205	0.254	0.0358	0.04309	0.041945122
0.5771	0.383552659	-0.1676	-0.0573	0.02695	0.030352249
0.4567	0.403856826	0.3668	-0.0046	0.0263	0.027717505

0.0735	0.01475094	0.2839	-0.3388	0.05153	0.1261515
0.5766	0.383552659	-0.1489	-0.0573	0.02709	0.030352249
0.4569	0.403856826	0.3496	-0.0046	0.02643	0.027717505
0.4559	0.403856826	0.2737	-0.0046	0.02723	0.027717505
0.4568	0.403856826	0.4112	-0.0046	0.02631	0.027717505
0.5764	0.383552659	-0.1746	-0.0573	0.02729	0.030352249
0.255	0.7425922	-0.2523	0.0127	0.04091	0.03108998
0.0029	0.97951509	1.651	0.0393	0.2111	0.09832864
0.814	0.91833056	-0.2089	-0.04	0.03078	0.04953318
0.4699	0.4787451	0.2177	0.0462	0.02348	0.02697978
0.0029	0.97951509	-1.502	0.0393	0.2221	0.09832864
0.3528	0.3818072	0.1775	-0.0222	0.02609	0.0277175
0.0029	0.97951509	-1.51	0.0393	0.2336	0.09832864
0.3439	0.5614494	0.1803	-0.0042	0.03185	0.02971991
0.0029	0.97951509	-1.737	0.0393	0.2311	0.09832864
0.0543	0.90385129	0.6146	-0.0393	0.05523	0.04616071
0.0029	0.97951509	-2.154	0.0393	0.2288	0.09832864
0.2288	0.049622	0.2111	0.0189	0.03351	0.06418236
0.5099	0.5889065	0.1499	-0.0291	0.02649	0.02971991
0.3011	0.1687823	0.1592	0.09	0.02442	0.03583252
0.5287	0.511173	-0.1619	-0.0678	0.02833	0.02708517
0.1087	0.01592168	0.307	-0.0561	0.04769	0.123833
0.1087	0.01592168	0.3169	-0.0561	0.04734	0.123833
0.1503	0.94072813	0.2185	-0.0929	0.03884	0.06165301
0.0542	0.90385129	0.5547	-0.0393	0.05665	0.04616071
0.013	0.97481682	-0.6608	0.1429	0.12	0.08852739
0.8652	0.110879532	0.2289	0.0088	0.04072	0.043315188
0.4845	0.580520179	0.3371	0.005	0.02703	0.027612115
0.4552	0.404401427	0.2805	-0.0037	0.0264	0.027717505
0.0027	0.01432312	-1.353	0.0059	0.2268	0.1171934
0.8286	0.0710573	-0.2018	-0.0458	0.03253	0.05311644
0.0555	0.05616582	-0.4386	-0.0374	0.0546	0.05954521
0.0798	0.93606052	-0.2562	-0.0655	0.04587	0.05480267
0.2849	0.90704723	-0.2546	-0.0268	0.03302	0.05111403
0.0844	0.93606052	-0.2824	-0.0655	0.04984	0.05480267
0.5089	0.7122266	0.1733	0.0574	0.02875	0.02993069
0.0798	0.93606052	-0.2797	-0.0655	0.04402	0.05480267
0.0798	0.93606052	-0.2492	-0.0655	0.04479	0.05480267
0.447	0.6673901	-0.1859	-0.0177	0.02487	0.02887679
0.0236	0.98670342	0.4573	-0.1599	0.07909	0.1209874
0.3334	0.480596621	0.2617	0.0078	0.03479	0.027295946
0.3209	0.480596621	0.2581	0.0078	0.02951	0.027295946
0.6884	0.6387578	0.3253	0.0178	0.02975	0.02824445
0.3302	0.480596621	-0.3379	0.0078	0.03798	0.027295946
0.3166	0.480596621	-0.3112	0.0078	0.03296	0.027295946
0.327	0.480596621	-0.4628	0.0078	0.02573	0.027295946
0.2677	0.662003	0.1581	0.006	0.02862	0.02856062
0.3262	0.480596621	-0.41	0.0078	0.02603	0.027295946
0.2681	0.6616651	0.2391	0.007	0.02846	0.02856062
0.3209	0.480596621	0.2755	0.0078	0.02934	0.027295946
0.3299	0.480596621	-0.261	0.0078	0.03857	0.027295946
0.2121	0.6806864	-1.119	-0.0232	0.03392	0.02919296
0.0278	0.01802522	-0.6094	-0.0065	0.105	0.1093946
0.2708	0.1764607	-0.3832	-0.0004	0.03992	0.03551635
0.5535	0.5366913	0.2977	0.003	0.03561	0.02961452
0.3267	0.480596621	0.4646	0.0078	0.02562	0.027295946
0.0427	0.9282526	0.3595	0.1071	0.06265	0.05164098
0.2729	0.178094739	0.1985	0.0253	0.02838	0.038678039

0.1461	0.7315893	0.6978	0.0431	0.03329	0.03056303
0.3298	0.480596621	-0.2575	0.0078	0.0387	0.027295946
0.2122	0.6806864	-1.139	-0.0232	0.03358	0.02919296
0.5707	0.4477274	-0.2061	-0.0058	0.03609	0.0298253
0.0704	0.98906866	0.4941	-0.0653	0.07133	0.1501804
0.271	0.1764607	-0.3895	-0.0004	0.04	0.03551635
0.5533	0.5366913	0.2922	0.003	0.03574	0.02961452
0.3268	0.480596621	-0.2388	0.0078	0.02599	0.027295946
0.3305	0.480596621	0.4941	0.0078	0.03702	0.027295946
0.3309	0.480596621	0.461	0.0078	0.03828	0.027295946
0.3267	0.480596621	0.1446	0.0078	0.02624	0.027295946
0.3334	0.480596621	-0.5517	0.0078	0.03439	0.027295946
0.9459	0.890610494	0.4291	-0.0305	0.07422	0.043736747
0.2209	0.7421583	-0.3541	0.0386	0.04034	0.03098459
0.3209	0.480596621	0.248	0.0078	0.02982	0.027295946
0.8668	0.06731554	0.4684	-0.0155	0.04088	0.05511884
0.3303	0.480596621	0.624	0.0078	0.03633	0.027295946
0.3262	0.480596621	-0.4504	0.0078	0.02605	0.027295946
0.2681	0.6616651	0.2834	0.007	0.02853	0.02856062
0.3334	0.480596621	0.5511	0.0078	0.03437	0.027295946
0.9459	0.890610494	-0.4313	-0.0305	0.07416	0.043736747
0.2209	0.7421583	0.361	0.0386	0.04027	0.03098459
0.0544	0.90385129	0.6113	-0.0393	0.05534	0.04616071
0.0356	0.988271966	-0.3926	-0.1162	0.06648	0.130894073
0.1622	0.8260896	0.2022	0.0596	0.03265	0.03583252
0.5686	0.5594995	-0.2005	-0.0028	0.02398	0.02740134
0.0357	0.988271966	0.5102	-0.1162	0.07408	0.130894073
0.3466	0.2330231	0.2237	-0.0264	0.02785	0.03214387
0.0175	0.9868665	-0.6561	0.1577	0.1188	0.1286809
0.2209	0.7447375	-0.3414	0.0427	0.0375	0.03108998
0.6316	0.2481714	0.2306	-0.0551	0.02558	0.03130076
0.1067	0.9468934	0.3186	0.0378	0.0403	0.06565782
0.6323	0.2481714	-0.1431	-0.0551	0.02594	0.03130076
0.534	0.4012964	-0.1679	0.0136	0.02462	0.0277175
0.6317	0.2481714	-0.4972	-0.0551	0.02506	0.03130076
0.167	0.7631517	-0.1925	0.0099	0.03366	0.03203848
0.0561	0.02701209	0.7463	-0.0586	0.05335	0.08273096
0.035	0.98073243	0.4946	0.0596	0.06834	0.1021227
0.1136	0.95896223	0.2435	0.0549	0.03994	0.06713327
0.0223	0.01729453	0.5735	0.0766	0.08703	0.1193012
0.6318	0.2481714	-0.1869	-0.0551	0.02576	0.03130076
0.2335	0.2474873	-0.1907	-0.0086	0.02929	0.03151154
0.8194	0.8256369	0.1924	0.0206	0.0321	0.0360433
0.0972	0.95894409	0.2363	0.0999	0.04232	0.06860873
0.5778	0.495913938	0.3092	-0.0551	0.03847	0.029509131
0.5778	0.495913938	0.3004	-0.0551	0.03886	0.029509131
0.5778	0.495913938	0.2928	-0.0551	0.0385	0.029509131
0.5778	0.495913938	0.2857	-0.0551	0.03952	0.029509131
0.5778	0.495913938	0.2864	-0.0551	0.03948	0.029509131
0.5778	0.495913938	0.2857	-0.0551	0.03841	0.029509131
0.0096	0.986995687	0.7543	0.2067	0.1293	0.112556256
0.2449	0.123361277	0.7524	0.0572	0.02735	0.044579865
0.0686	0.976189635	0.3586	0.0148	0.05081	0.087789664
0.0096	0.986995687	0.7582	0.2067	0.1312	0.112556256
0.2449	0.123361277	0.8088	0.0572	0.02785	0.044579865
0.0686	0.976189635	0.3667	0.0148	0.05205	0.087789664
0.0096	0.986995687	0.7724	0.2067	0.1306	0.112556256
0.2449	0.123361277	0.9207	0.0572	0.02691	0.044579865

0.0686	0.976189635	0.425	0.0148	0.05195	0.087789664
0.6081	0.4734739	-0.1965	0.0146	0.02481	0.02950913
0.2304	0.246139	-0.1667	-0.0117	0.02948	0.03435706
0.5205	0.4073034	-0.1343	0.0038	0.0244	0.03024686
0.3161	0.7159645	0.1572	0.0285	0.02688	0.03024686
0.601	0.2667026	0.3858	0.0135	0.02783	0.03077381
0.199	0.8235794	0.2512	-0.0489	0.03397	0.03583252
0.601	0.2667026	0.3661	0.0135	0.02791	0.03077381
0.199	0.8235794	0.233	-0.0489	0.03402	0.03583252
0.1704	0.0787949	0.2334	-0.0625	0.03629	0.05058708
0.446	0.605326605	0.6864	0.0108	0.02442	0.027822895
0.051	0.100653834	0.5489	-0.0309	0.06086	0.04679305
0.1024	0.893214404	0.3354	0.0252	0.04422	0.044263696
0.1325	0.8378662	-0.2711	0.0026	0.03734	0.03867804
0.4074	0.5478163	-0.1665	-0.1275	0.02485	0.02750673
0.821	0.8228947	0.2255	0.0247	0.03219	0.03920499
0.6088	0.4717883	-0.2046	0.0168	0.02482	0.02719056
0.3161	0.7159645	0.1645	0.0285	0.02685	0.03024686
0.821	0.8228947	0.3353	0.0247	0.03177	0.03920499
0.3094	0.7911125	0.1684	-0.0081	0.02631	0.03319777
0.3161	0.7159645	0.1494	0.0285	0.02674	0.03024686
0.6102	0.5273167	-0.1785	0.0162	0.02475	0.02719056
0.2834	0.730585995	0.173	0.0036	0.02971	0.030563028
0.265	0.1308152	-0.182	0.0122	0.03168	0.04405292
0.7709	0.7444521	-0.1919	-0.0312	0.03092	0.03098459
0.3244	0.2322854	0.1937	0.0053	0.02628	0.04078583
0.4167	0.4315696	-0.1446	0.0212	0.02608	0.02740134
0.766	0.742607	-0.4286	-0.0272	0.0278	0.0308792
0.1636	0.05045666	-0.2122	0.0226	0.03296	0.06734405
0.4168	0.4315696	-0.1628	0.0212	0.02568	0.02740134
0.7659	0.742607	-0.4355	-0.0272	0.02736	0.0308792
0.1635	0.05045666	-0.2015	0.0226	0.03253	0.06734405
0.5882	0.521369452	0.2882	-0.0535	0.03927	0.029509131
0.5754	0.493399851	0.2584	-0.0629	0.03896	0.029614521
0.5747	0.493399851	0.2382	-0.0629	0.03976	0.029614521
0.593	0.504110418	0.2305	-0.0526	0.03896	0.029509131
0.3841	0.8015662	-0.276	-0.0394	0.04325	0.03730797
0.593	0.504110418	0.2484	-0.0526	0.03809	0.029509131
0.5961	0.5685548	0.2114	0.0358	0.03543	0.02740134
0.0617	0.0711945	-0.8239	-0.026	0.07087	0.05364338
0.5998	0.5685548	0.1789	0.0358	0.02478	0.02740134
0.7284	0.2469821	0.1513	0.0191	0.0275	0.03435706
0.0584	0.05979309	-0.858	-0.0259	0.05093	0.05849131
0.5998	0.5685548	0.1396	0.0358	0.02534	0.02740134
0.0651	0.0711945	-0.432	-0.026	0.05081	0.05364338
0.5995	0.5685548	0.1758	0.0358	0.02511	0.02740134
0.0586	0.05979309	-0.7439	-0.0259	0.05193	0.05849131
0.5996	0.5685548	0.1799	0.0358	0.0249	0.02740134
0.0586	0.05979309	-0.7352	-0.0259	0.05156	0.05849131
0.5997	0.5685548	0.1623	0.0358	0.02499	0.02740134
0.065	0.0711945	-0.6739	-0.026	0.04955	0.05364338
0.5997	0.5685548	0.1709	0.0358	0.02524	0.02740134
0.0586	0.05979309	-0.7073	-0.0259	0.05233	0.05849131
0.5997	0.5685548	0.1681	0.0358	0.02484	0.02740134
0.065	0.0711945	-0.7377	-0.026	0.04904	0.05364338
0.5996	0.5685548	0.1798	0.0358	0.02528	0.02740134
0.0651	0.0711945	-0.5307	-0.026	0.05059	0.05364338
0.6173	0.5197658	0.1811	0.023	0.02845	0.02708517

0.1965	0.90110612	0.4371	-0.0062	0.03249	0.04942779
0.5339	0.4012964	0.1466	0.0136	0.02442	0.0277175
0.0562	0.02701209	-0.3526	-0.0586	0.05371	0.08273096
0.6314	0.2482242	0.3898	-0.0536	0.02585	0.03130076
0.5341	0.4012964	0.1359	0.0136	0.02451	0.0277175
0.6002	0.2757794	0.2841	-0.0467	0.02593	0.03035225
0.7711	0.8085377	-0.2187	-0.0429	0.0399	0.03414628
0.1068	0.05372035	0.4909	0.0556	0.05533	0.05954521
0.3544	0.7992945	-0.165	0.0167	0.02926	0.03383011
0.0756	0.96765703	-0.2936	-0.0326	0.05364	0.07999082
0.5197	0.4267604	0.3938	0.0233	0.03247	0.0277175
0.1527	0.8454784	-0.2816	0.0595	0.04685	0.03741336
0.3672	0.375473785	-0.145	-0.0119	0.02605	0.028139064
0.2461	0.123329992	-0.5526	0.0562	0.02805	0.044579865
0.4243	0.843785	-0.1602	-0.0177	0.02905	0.0401535
0.5967	0.2629509	-0.1537	0.0358	0.02608	0.03340855
0.2642	0.275708	-0.1522	-0.003	0.02611	0.03298699
0.5685	0.739911	0.1657	-0.0412	0.0284	0.03351394
0.1412	0.1588857	-0.3008	0.0342	0.03802	0.03688641
0.5359	0.2331346	0.1639	-0.0486	0.02894	0.03467323
0.8222	0.4004856	0.3571	-0.0152	0.0369	0.03024686
0.1672	0.7631517	-0.2854	0.0099	0.04636	0.03203848
0.0564	0.02701209	0.9344	-0.0586	0.07016	0.08273096
0.6302	0.2482242	-0.5719	-0.0536	0.03295	0.03130076
0.0294	0.9634158	0.5453	0.0998	0.09922	0.07271893
0.0376	0.98073243	0.5783	0.0596	0.0916	0.1021227
0.3057	0.7105329	0.1645	-0.0449	0.02658	0.03246004
0.2995	0.512842806	0.1769	0.0143	0.02681	0.027295946
0.567	0.5578927	-0.1407	0.0064	0.02479	0.02729595
0.0627	0.05587868	-0.3193	-0.0057	0.05783	0.05975599
0.2371	0.876309667	-0.2761	0.0472	0.03208	0.040891224
0.4991	0.7011108	0.3426	0.0741	0.0281	0.02961452
0.6009	0.4480597	0.1877	0.0143	0.03056	0.02971991
0.1385	0.1588857	-0.4218	0.0342	0.03923	0.03688641
0.2578	0.3227183	0.2919	0.0324	0.02741	0.02898218
0.2578	0.3227183	0.299	0.0324	0.0274	0.02898218
0.408	0.5471778	-0.2027	-0.1252	0.02492	0.02750673
0.4878	0.650616	-0.1633	0.0078	0.02494	0.02856062
0.3917	0.3568843	0.1554	0.0215	0.02512	0.02824445
0.4074	0.5478163	-0.1749	-0.1275	0.02521	0.02750673
0.4075	0.5478163	-0.2111	-0.1275	0.02508	0.02750673
0.4878	0.650616	-0.1647	0.0078	0.02506	0.02856062
0.3917	0.3568843	0.1551	0.0215	0.02525	0.02824445
0.2371	0.876309667	-0.4802	0.0472	0.03135	0.040891224
0.1238	0.2268457	0.2917	0.0345	0.04331	0.03246004
0.4463	0.605326605	0.567	0.0108	0.0258	0.027822895
0.0512	0.100653834	0.4895	-0.0309	0.06186	0.04679305
0.1715	0.91857449	0.2019	-0.0743	0.03657	0.04963857
0.1027	0.893214404	0.2885	0.0252	0.04484	0.044263696
0.4466	0.605326605	0.5477	0.0108	0.02564	0.027822895
0.1024	0.893214404	0.2777	0.0252	0.0445	0.044263696
0.0214	0.989015672	0.5264	0.0821	0.09617	0.147440264
0.4466	0.605326605	0.644	0.0108	0.02499	0.027822895
0.6622	0.182408031	0.1732	-0.0035	0.02866	0.036886413
0.4462	0.605326605	0.521	0.0108	0.02584	0.027822895
0.051	0.100653834	0.4147	-0.0309	0.06162	0.04679305
0.1025	0.893214404	0.2716	0.0252	0.0445	0.044263696
0.472	0.600484792	0.2073	0.009	0.02706	0.027717505

0.4462	0.60793966	0.4749	0.0028	0.02583	0.027822895
0.0512	0.100653834	0.4331	-0.0309	0.0606	0.04679305
0.4849	0.580520179	0.2396	0.005	0.02713	0.027612115
0.4461	0.608089804	0.4829	0.0038	0.02629	0.027822895
0.4466	0.608089804	0.6021	0.0038	0.02516	0.027822895
0.0512	0.100653834	0.447	-0.0309	0.06122	0.04679305
0.5978	0.559980812	-0.1899	0.0023	0.02764	0.027295946
0.7662	0.742607	-0.4526	-0.0272	0.02659	0.0308792
0.5089	0.542335	-0.1295	0.0032	0.02364	0.02719056
0.1634	0.05045666	-0.1852	0.0226	0.03176	0.06734405
0.7661	0.742607	-0.4689	-0.0272	0.02616	0.0308792
0.0236	0.97244648	0.4397	-0.1535	0.07676	0.09442922
0.4182	0.5706917	-0.1552	0.0227	0.0249	0.02740134
0.7662	0.742607	-0.4525	-0.0272	0.027	0.0308792
0.1634	0.05045666	-0.1929	0.0226	0.03221	0.06734405
0.7662	0.742607	-0.3114	-0.0272	0.02551	0.0308792
0.4182	0.5706917	-0.1334	0.0227	0.02345	0.02740134
0.5635	0.7424431	0.1976	0.0303	0.02744	0.03108998
0.0022	0.94034804	-1.668	0.0302	0.2802	0.05764819
0.5639	0.7424431	0.303	0.0303	0.02642	0.03108998
0.2239	0.7918039	-0.2185	-0.0234	0.03362	0.03646485
0.3449	0.5614494	0.1905	-0.0042	0.03102	0.02971991
0.3428	0.5614494	0.1887	-0.0042	0.0284	0.02971991
0.3428	0.5614494	0.1801	-0.0042	0.02952	0.02971991
0.3439	0.5614494	0.1894	-0.0042	0.02987	0.02971991
0.1339	0.08687124	-0.2103	0.0862	0.03733	0.04763617
0.5292	0.4881992	-0.1609	-0.0661	0.02705	0.02708517
0.5305	0.4881992	-0.1838	-0.0661	0.02676	0.02708517
0.5304	0.4881992	-0.1468	-0.0661	0.02633	0.02708517
0.2441	0.731635994	0.3259	-0.033	0.02935	0.030879198
0.1712	0.92402453	0.1995	0.105	0.03596	0.05490806
0.5264	0.5238236	0.1526	0.0124	0.02725	0.02950913
0.1983	0.8238965	-0.4161	-0.0218	0.03368	0.03562174
0.1927	0.7476587	0.1659	0.03	0.02878	0.03119537
0.2125	0.01349708	0.2827	0.017	0.03403	0.1150856
0.2128	0.01349708	0.2434	0.017	0.03534	0.1150856
0.7665	0.7524722	0.196	0.01	0.02941	0.03151154
0.0562	0.02701209	-0.3044	-0.0586	0.05397	0.08273096
0.6313	0.2482242	0.317	-0.0536	0.02622	0.03130076
0.0624	0.07756574	-0.4732	-0.042	0.05907	0.05058708
0.2742	0.178094739	0.2327	0.0253	0.03137	0.038678039
0.0624	0.07756574	-0.4223	-0.042	0.05867	0.05058708
0.2369	0.876309667	-0.4101	0.0472	0.03167	0.040891224
0.1553	0.768573	0.3225	-0.0416	0.04073	0.03488401
0.2372	0.876309667	-0.3418	0.0472	0.03192	0.040891224
0.1365	0.7371554	0.2659	0.0349	0.04133	0.03077381
0.5014	0.6106737	-0.3191	-0.0275	0.03045	0.03014147
0.7759	0.8096676	-0.1749	-0.043	0.02923	0.03425167
0.1068	0.9468934	0.4244	0.0378	0.0402	0.06565782
0.7758	0.8096676	-0.1813	-0.043	0.02898	0.03425167
0.1074	0.94504495	0.349	0.0502	0.03994	0.05891287
0.7759	0.8096676	-0.1698	-0.043	0.02871	0.03425167
0.1075	0.05372035	0.5734	0.0556	0.03893	0.05954521
0.35	0.6045065	0.2184	-0.0345	0.02531	0.0277175
0.2479	0.123361277	-0.6399	0.0572	0.02726	0.044579865
0.0704	0.976189635	-0.3061	0.0148	0.04812	0.087789664
0.2479	0.123361277	-0.7013	0.0572	0.02722	0.044579865
0.0704	0.976189635	-0.2863	0.0148	0.04872	0.087789664

0.1482	0.251945786	-0.2278	0.0102	0.03508	0.03414628
0.5946	0.389494173	0.2065	0.0239	0.02568	0.027612115
0.0247	0.028211035	-0.507	-0.0068	0.08113	0.082941735
0.0272	0.977534109	0.4617	0.0787	0.0778	0.091267526
0.1936	0.6806864	-1.223	-0.0232	0.0362	0.02919296
0.2617	0.1764607	-0.3413	-0.0004	0.04213	0.03551635
0.5596	0.5366913	0.2935	0.003	0.03701	0.02961452
0.1896	0.6806864	-1.151	-0.0232	0.03884	0.02919296
0.5586	0.5322823	0.2625	0.0016	0.03721	0.02729595
0.2604	0.1764607	-0.3677	-0.0004	0.04232	0.03551635
0.2132	0.6806864	-0.5183	-0.0232	0.03788	0.02919296
0.1527	0.8454784	0.2541	0.0595	0.04602	0.03741336
0.2722	0.1764607	-0.2756	-0.0004	0.03696	0.03551635
0.2121	0.6806864	-1.143	-0.0232	0.03378	0.02919296
0.571	0.4477274	-0.2031	-0.0058	0.03621	0.0298253
0.0704	0.98906866	0.4387	-0.0653	0.07202	0.1501804
0.2708	0.1764607	-0.4007	-0.0004	0.04013	0.03551635
0.5535	0.5366913	0.2828	0.003	0.03593	0.02961452
0.2131	0.6806864	-0.3278	-0.0232	0.03915	0.02919296
0.2177	0.849878	-0.2252	-0.0118	0.03955	0.04173434
0.1934	0.6806864	-1.214	-0.0232	0.03655	0.02919296
0.2613	0.1764607	-0.326	-0.0004	0.04234	0.03551635
0.5593	0.5366913	0.2876	0.003	0.03706	0.02961452
0.1938	0.6806864	-1.219	-0.0232	0.03627	0.02919296
0.2619	0.1764607	-0.3366	-0.0004	0.04212	0.03551635
0.5592	0.5366913	0.2938	0.003	0.03696	0.02961452
0.2131	0.6806864	-0.3767	-0.0232	0.03917	0.02919296
0.1845	0.6806864	-1.138	-0.0232	0.03974	0.02919296
0.0565	0.93029537	0.5006	-0.0175	0.0784	0.05417033
0.258	0.1764607	-0.3645	-0.0004	0.04209	0.03551635
0.0709	0.92082395	-0.4918	0.0123	0.07038	0.05058708
0.2131	0.6806864	-0.3696	-0.0232	0.03875	0.02919296
0.3642	0.439263237	0.1797	-0.0492	0.02746	0.027295946
0.4599	0.569179938	0.1721	-0.0621	0.02612	0.027506725
0.0201	0.98854203	0.4952	-0.1458	0.0895	0.1303671
0.5982	0.3530986	0.167	-0.0007	0.02504	0.02845523
0.481	0.5123808	-0.1722	-0.0634	0.02583	0.02697978
0.0888	0.98911045	0.313	0.0849	0.04821	0.1322641
0.5719	0.5117309	-0.2043	0.032	0.02491	0.02729595
0.776	0.7241723	0.3801	-0.0102	0.02898	0.03035225
0.1075	0.05372035	0.5555	0.0556	0.03963	0.05954521
0.35	0.6045065	0.2383	-0.0345	0.02566	0.0277175
0.1975	0.90110612	0.4475	-0.0062	0.03413	0.04942779
0.1975	0.90110612	0.2826	-0.0062	0.03426	0.04942779
0.4923	0.6525941	0.1831	-0.0296	0.02869	0.03098459
0.476	0.754849	0.1669	-0.0197	0.02793	0.03456784
0.1974	0.90110612	0.2094	-0.0062	0.03421	0.04942779
0.7366	0.686247	0.3611	0.0029	0.02998	0.02950913
0.1051	0.1349908	-0.3173	0.0421	0.04155	0.03952116
0.2763	0.308565431	0.3359	-0.0254	0.02969	0.029509131
0.0676	0.8930332	0.2947	-0.0476	0.0539	0.04436909
0.1205	0.90719217	0.227	0.0143	0.04151	0.05058708
0.0421	0.01583341	0.3527	-0.0175	0.0624	0.1240437
0.0201	0.976143807	-0.4865	-0.0115	0.08656	0.093586101
0.3347	0.47856234	-0.1484	0.0065	0.02581	0.029614521
0.0512	0.100653834	0.3622	-0.0309	0.06121	0.04679305
0.6904	0.6387578	0.2454	0.0178	0.02618	0.02824445
0.0336	0.04173253	-0.4165	-0.1194	0.06803	0.06913568

0.6904	0.6387578	0.2224	0.0178	0.0265	0.02824445
0.0336	0.95828728	-0.4084	-0.1181	0.06877	0.06924107
0.6349	0.7338248	-0.1403	0.01	0.02562	0.03108998
0.134	0.08687124	0.2194	0.0862	0.0369	0.04763617
0.1343	0.08687124	0.2435	0.0862	0.0371	0.04763617
0.1343	0.08687124	-0.2435	0.0862	0.0371	0.04763617
0.1343	0.08687124	-0.2034	0.0862	0.03346	0.04763617
0.6414	0.4306812	-0.1458	-0.03	0.02634	0.02971991
0.1019	0.8685341	-0.2169	-0.0077	0.03774	0.03994272
0.1343	0.08687124	-0.2283	0.0862	0.03646	0.04763617
0.6455	0.2852371	0.1004	-0.0431	0.0178	0.03003608
0.279	0.677216374	-0.4474	-0.0215	0.02945	0.029192962
0.5251	0.7070426	-0.1994	-0.0119	0.03417	0.02971991
0.231	0.876309667	-0.1809	0.0472	0.03084	0.040891224
0.2358	0.876309667	0.5889	0.0472	0.02846	0.040891224
0.0532	0.043457713	0.3483	0.119	0.05681	0.066184765
0.1976	0.1744217	-0.4779	-0.0102	0.03389	0.03878343
0.2757	0.9303338	0.23	-0.0624	0.03381	0.05828053
0.2317	0.3256302	-0.2188	0.0276	0.03213	0.03161693
0.4305	0.446061027	-0.1723	0.0049	0.02756	0.027401336
0.2402	0.123361277	-0.5652	0.0572	0.0301	0.044579865
0.4331	0.446061027	-0.1534	0.0049	0.02565	0.027401336
0.2474	0.123361277	-0.7124	0.0572	0.02686	0.044579865
0.4331	0.446061027	-0.1436	0.0049	0.02619	0.027401336
0.2474	0.123329992	-0.6848	0.0562	0.02772	0.044579865
0.2162	0.7446472	-0.2525	0.0422	0.03019	0.03108998
0.5004	0.474756961	-0.1371	0.0221	0.02509	0.027190556
0.5698	0.5594995	0.1457	-0.0028	0.02506	0.02740134
0.4349	0.446061027	0.1529	0.0049	0.02614	0.027401336
0.2449	0.123361277	0.6546	0.0572	0.02797	0.044579865
0.1375	0.7783377	-0.2591	-0.0453	0.03497	0.0328816
0.3505	0.1615462	0.3276	-0.0018	0.02803	0.03635946
0.028	0.98708127	-0.442	-0.3146	0.08067	0.1244653
0.0376	0.976453893	0.3947	-0.023	0.07143	0.090319018
0.0825	0.93411417	-0.279	-0.206	0.04807	0.05817514
0.5363	0.4887289	-0.1543	-0.0689	0.02682	0.02708517
0.1242	0.91741931	0.2582	0.033	0.04145	0.04911162
0.3188	0.8494761	-0.2784	0.0454	0.04395	0.04068044
0.0543	0.90385129	0.592	-0.0393	0.05509	0.04616071
0.0541	0.90385129	0.3212	-0.0393	0.05805	0.04616071
0.0096	0.95561488	-0.7401	-0.0869	0.13	0.06650093
0.0543	0.90385129	0.5156	-0.0393	0.05562	0.04616071
0.0575	0.90385129	0.386	-0.0393	0.0569	0.04616071
0.0542	0.90385129	0.4625	-0.0393	0.05656	0.04616071
0.0575	0.90385129	0.4562	-0.0393	0.05531	0.04616071
0.1983	0.8238965	-0.3589	-0.0218	0.03258	0.03562174
0.1987	0.176446	-0.5184	-0.0247	0.03354	0.03562174
0.199	0.8235794	0.5273	-0.0489	0.03289	0.03583252
0.1986	0.176446	-0.4171	-0.0247	0.03444	0.03562174
0.199	0.8235794	0.3653	-0.0489	0.03405	0.03583252
0.2819	0.90704723	-0.2295	-0.0268	0.03657	0.05111403
0.5013	0.6106737	-0.1923	-0.0275	0.02928	0.03014147
0.2429	0.7415999	-0.2192	0.0126	0.03318	0.03108998
0.294	0.7108824	-0.3048	0.0264	0.03055	0.02993069
0.2426	0.7415999	-0.2093	0.0126	0.03307	0.03108998
0.2941	0.7108824	-0.2855	0.0264	0.03051	0.02993069
0.2429	0.7415999	-0.2184	0.0126	0.03318	0.03108998
0.294	0.7108824	-0.3012	0.0264	0.03056	0.02993069

0.2426	0.7415999	-0.2089	0.0126	0.03307	0.03108998
0.2941	0.7108824	-0.2821	0.0264	0.03052	0.02993069
0.1075	0.94504495	0.2969	0.0502	0.04016	0.05891287
0.0997	0.7653477	0.3322	-0.0138	0.06048	0.03224926
0.344	0.3459092	-0.3029	0.0085	0.02564	0.02845523
0.3435	0.3459092	-0.2794	0.0085	0.02525	0.02845523
0.8488	0.922622738	-0.2072	0.0245	0.03711	0.050060133
0.2447	0.123329992	0.7582	0.0562	0.02825	0.044579865
0.0688	0.976189635	0.3617	0.0148	0.05191	0.087789664
0.3413	0.6045065	0.1714	-0.0345	0.0264	0.0277175
0.3414	0.6045065	0.182	-0.0345	0.02618	0.0277175
0.1884	0.862196494	-0.186	0.0559	0.03337	0.039837327
0.3011	0.1687823	0.1972	0.09	0.02757	0.03583252
0.3009	0.1687823	0.1912	0.09	0.02822	0.03583252
0.1991	0.7401346	0.1921	0.0303	0.03166	0.03077381
0.6743	0.4695682	0.1857	-0.0222	0.02686	0.02729595
0.4704	0.4787451	-0.1726	0.0462	0.0256	0.02697978
0.4699	0.4787451	0.1507	0.0462	0.02494	0.02697978
0.1925	0.2517236	0.2134	0.0304	0.02962	0.03108998
0.6737	0.4695682	0.1433	-0.0222	0.02475	0.02729595
0.5717	0.5117309	-0.1582	0.032	0.02739	0.02729595
0.2454	0.123361277	0.5196	0.0572	0.031	0.044579865
0.2447	0.123361277	0.6258	0.0572	0.0287	0.044579865
0.2447	0.123361277	0.929	0.0572	0.0268	0.044579865
0.0688	0.976189635	0.4245	0.0148	0.05172	0.087789664
0.1104	0.127086456	-0.2319	-0.0102	0.04179	0.040680445
0.2447	0.123329992	-0.5889	0.0562	0.02815	0.044579865
0.2645	0.3216039	-0.1835	0.0217	0.02885	0.02908757
0.2473	0.123329992	-0.6984	0.0562	0.02689	0.044579865
0.0686	0.976189635	-0.3019	0.0148	0.04947	0.087789664
0.5982	0.3530986	0.1434	-0.0007	0.025	0.02845523
0.1853	0.6507084	-0.8358	-0.0173	0.04903	0.02856062
0.2544	0.1764607	-0.2821	-0.0004	0.048	0.03551635
0.5691	0.5366913	0.2277	0.003	0.04091	0.02961452
0.2236	0.6507084	-0.3128	-0.0173	0.03922	0.02856062
0.0861	0.058766079	0.3805	0.081	0.06635	0.056699687
0.0103	0.95475878	-0.7447	-0.0968	0.1252	0.06660632
0.1984	0.178909	0.2953	0.0736	0.03367	0.0349894
0.0987	0.92084717	-0.5191	0.0455	0.04044	0.05069247
0.3456	0.06052395	0.1905	0.0311	0.03406	0.0596506
0.3458	0.06052395	-0.1934	0.0311	0.03245	0.0596506
0.0413	0.96767917	-0.383	0.0192	0.06981	0.0760914
0.0081	0.0161378	1.453	0.0375	0.1814	0.1147694
0.0246	0.98997111	0.4393	-0.0431	0.07886	0.1498642
0.0609	0.95486603	0.292	0.0146	0.05091	0.06744944
0.3508	0.376821	-2.745	-0.0192	0.372	0.02782289
0.0201	0.976143807	0.483	-0.0115	0.08768	0.093586101
0.8262	0.1804233	0.4384	0.0041	0.04722	0.03562174
0.8258	0.1804233	0.4516	0.0041	0.04677	0.03562174
0.0194	0.98573486	-0.7731	0.0585	0.089	0.1147694
0.9403	0.94745022	0.3174	0.1257	0.05158	0.06133684
0.0194	0.98573486	-0.6069	0.0585	0.09018	0.1147694
0.1088	0.96940732	-0.406	0.0462	0.03854	0.07798842
0.3227	0.8117048	-0.1502	-0.0071	0.02624	0.03467323
0.3227	0.8117048	0.1512	-0.0071	0.0265	0.03467323
0.0957	0.04141161	0.2186	0.0809	0.03992	0.06660632
0.3067	0.7113613	0.1479	-0.053	0.02672	0.03003608
0.2342	0.3262434	0.1834	-0.0287	0.03032	0.02866601

0.1097	0.96878866	-0.3964	0.0429	0.0389	0.07735608
0.1095	0.96878866	-0.3961	0.0429	0.03859	0.07735608
0.1096	0.96977877	-0.3396	0.0168	0.04218	0.0791477
0.8198	0.1742978	0.287	0.0222	0.03212	0.0360433
0.2911	0.0658253	-0.4511	-0.0101	0.04468	0.05849131
0.7326	0.395538	-0.1677	-0.072	0.03001	0.03024686
0.5737	0.5594995	-0.1904	-0.0028	0.03354	0.02740134

Pval.exposure	Pval.outcome	Samplesize.exposure	Samplesize.outcome	F-statistics
5.08E-22	0.8309875	3113	24589	94.433921
3.03E-08	0.054588538	3113	24589	30.8460247
2.35E-09	0.4823112	3113	24589	35.8837196
3.61E-24	0.8309875	3113	24589	104.592518
3.34E-08	0.7552921	3657	24589	30.6480727
1.75E-13	0.5286803	3657	24589	54.6393818
2.90E-10	0.8072368	3657	24589	39.9546486
2.50E-21	0.5366666	3657	24589	90.9964847
7.20E-11	0.3460664	3657	24589	42.7364669
4.23E-08	0.03055629	3657	24589	30.1554794
1.62E-08	0.06632357	3425	24589	32.0651372
4.81E-10	0.8326193	3425	24589	38.9752583
5.47E-09	0.482611285	3425	24589	34.1690036
1.51E-08	0.6491579	3425	24589	32.1932892
2.27E-08	0.01690166	3425	24589	31.4019485
1.35E-11	0.3797726	3425	24589	46.0473867
2.50E-09	0.2488553	3425	24589	35.7177272
2.33E-08	0.8028196	3425	24589	31.3297637
2.10E-08	0.7161514	3425	24589	31.5439194
1.53E-08	0.3332334	3425	24589	32.1619709
5.41E-09	0.02036439	3425	24589	34.2156464
6.06E-09	0.01420326	3425	24589	33.9781927
4.60E-08	0.0300326	3425	24589	30.0243948
1.00E-08	0.2904912	3425	24589	32.9662782
1.58E-13	0.5551245	3425	24589	54.9027435
3.38E-08	0.05089171	3425	24589	30.6105637
9.81E-10	0.3025941	3425	24589	37.5650364
7.92E-09	0.9269062	3425	24589	33.4684161
1.23E-12	0.7085376	3425	24589	50.8305257
9.65E-09	0.7940912	3425	24589	33.070645
3.97E-10	0.5916791	3425	24589	39.3568559
7.31E-09	0.5387889	3425	24589	33.6294994
2.10E-08	0.2937835	3425	24589	31.5531389
3.62E-09	0.460341	3425	24589	34.9838363
1.26E-17	0.724211	3425	24589	73.8219337
5.19E-09	0.6933808	3425	24589	34.2901131
7.31E-13	0.1668625	3425	24589	51.866732
9.74E-16	0.3510995	3425	24589	65.0990418
6.60E-19	0.4949787	3425	24589	79.7962996
1.16E-20	0.505032	3425	24589	88.0070199
5.07E-09	0.741234	3425	24589	34.3298622
8.99E-10	0.9776872	3425	24589	37.7553834
5.21E-18	0.77899	3425	24589	75.6404654
4.84E-08	0.1202538	3425	24589	29.9179863
1.44E-09	0.2019776	3425	24589	36.8165934
7.70E-09	0.4839411	3425	24589	33.5235438
2.66E-08	0.3291751	3427	24589	31.0871978
2.71E-09	0.07798494	3427	24589	35.5738614
5.88E-09	0.06051131	3427	24589	34.0607744
1.19E-09	0.8397947	3657	24589	37.1918787
1.72E-08	0.3485747	3657	24589	31.9470699
9.14E-11	0.9464053	3657	24589	42.25
3.60E-09	0.7060723	3113	24589	35.0255209
1.55E-08	0.2996777	3113	24589	32.1428186
2.51E-08	0.7399399	3595	24589	31.1822037
1.86E-11	0.7436791	3408	24589	45.3842353

2.65E-23	0.06632357	3408	24589	100.344446
2.49E-12	0.4005125	3408	24589	49.4320666
3.28E-12	0.7710772	3408	24589	48.8668548
4.67E-09	0.5822674	3408	24589	34.5030008
1.04E-12	0.1075042	3408	24589	51.1481869
2.34E-09	0.4537538	3408	24589	35.8612049
5.14E-09	0.906798	3408	24589	34.3230283
6.43E-13	0.7360253	3408	24589	52.1149202
7.06E-09	0.4927623	3408	24589	33.7046707
8.74E-16	0.3579102	3408	24589	65.3166444
2.03E-10	0.04537502	3408	24589	40.6888156
1.30E-11	0.6601926	3408	24589	46.1301782
4.77E-14	0.5929887	3408	24589	57.327378
1.11E-08	0.6043698	3408	24589	32.809984
7.67E-11	0.7507452	3408	24589	42.5994127
1.01E-17	0.482611285	3408	24589	74.3178701
1.93E-09	0.5417661	3408	24589	36.268938
3.61E-09	0.83275214	3408	24589	35.008188
2.81E-27	0.5934943	3408	24589	119.085815
9.85E-16	0.4222351	3408	24589	65.0801497
3.67E-08	0.547545612	3408	24589	30.4595534
2.24E-09	0.7818982	3408	24589	35.9458579
1.21E-11	0.7725698	3408	24589	46.2955033
1.69E-12	0.8075206	3408	24589	50.1937317
8.40E-12	0.1134504	3408	24589	47.0086773
1.88E-11	0.3371376	3408	24589	45.415879
1.36E-10	0.623719	3408	24589	41.460122
1.01E-08	0.3779235	3408	24589	32.9974026
1.15E-08	0.5243431	3408	24589	32.7475024
7.06E-14	0.744235	3408	24589	56.5271923
4.87E-17	0.2323341	3408	24589	71.1274408
2.61E-29	0.090075932	3408	24589	128.725233
6.21E-27	0.3799589	3408	24589	117.490004
6.49E-20	0.924513	3408	24589	84.5064267
1.04E-32	0.01690166	3408	24589	144.890864
2.05E-08	0.448127	3408	24589	31.6009129
1.40E-09	0.4286125	3408	24589	36.8632263
2.47E-14	0.1684828	3408	24589	58.6237154
2.29E-36	0.3797726	3408	24589	162.405868
6.51E-10	0.7168001	3408	24589	38.392378
7.08E-09	0.3643644	3408	24589	33.6832437
2.10E-11	0.08550205	3408	24589	45.1728542
6.49E-25	0.8028196	3408	24589	107.946692
5.99E-09	0.1596181	3408	24589	34.0098093
9.89E-11	0.417552	3408	24589	42.1155047
2.52E-14	0.6830558	3408	24589	58.6152919
8.96E-11	0.1744096	3408	24589	42.292627
2.73E-09	0.8310586	3408	24589	35.5399408
2.64E-10	0.7723305	3408	24589	40.1698255
1.68E-11	0.528247	3408	24589	45.6425849
4.08E-08	0.6056843	3408	24589	30.2407466
1.31E-22	0.5758087	3408	24589	97.1276189
1.41E-14	0.1058492	3408	24589	59.7421788
4.46E-08	0.1115964	3408	24589	30.0764474
1.44E-10	0.3864961	3408	24589	41.3559634
7.11E-23	0.7541433	3408	24589	98.3794769
7.31E-13	0.2668213	3408	24589	51.8473801
3.86E-08	0.441046	3408	24589	30.3425447

3.50E-12	0.8377364	3408	24589	48.7231644
7.63E-11	0.372357	3408	24589	42.6066076
4.77E-08	0.9969503	3408	24589	29.925699
4.89E-28	0.7833173	3408	24589	122.684763
2.77E-08	0.3222431	3408	24589	30.98815
2.41E-11	0.4456185	3408	24589	44.8889162
1.55E-11	0.722804169	3408	24589	45.8169508
1.90E-23	0.0300326	3408	24589	101.037906
4.38E-13	0.9795011	3408	24589	52.892562
3.79E-11	0.9672602	3408	24589	44.0129475
1.11E-45	0.2904912	3408	24589	207.362673
1.22E-08	0.3086749	3408	24589	32.6194877
2.08E-08	0.4751507	3408	24589	31.5694322
6.59E-16	0.3673392	3408	24589	65.9129169
1.18E-41	0.5551245	3408	24589	187.70389
8.78E-11	0.02447494	3408	24589	42.3269581
5.98E-46	0.05089171	3408	24589	208.649192
3.08E-08	0.3047136	3408	24589	30.8150898
9.35E-10	0.636512997	3408	24589	37.6703139
6.12E-16	0.07009361	3408	24589	66.0430967
1.34E-20	0.3025941	3408	24589	87.7070252
6.47E-09	0.6513004	3408	24589	33.8450837
1.75E-29	0.9213789	3408	24589	129.543193
7.67E-19	0.4954564	3408	24589	79.5038676
1.58E-08	0.7745914	3408	24589	32.0943604
1.21E-19	0.3972704	3408	24589	83.2389379
7.48E-09	0.7927446	3408	24589	33.5938467
4.98E-10	0.8902279	3408	24589	38.9145361
5.51E-22	0.2445477	3408	24589	94.1736979
2.90E-08	0.3707885	3408	24589	30.9246156
9.46E-12	0.7895135	3408	24589	46.751778
1.55E-14	0.2187742	3408	24589	59.5541904
3.00E-09	0.3744823	3408	24589	35.3738278
1.63E-09	0.9269062	3408	24589	36.5796342
5.42E-14	0.3880096	3408	24589	57.0337499
4.02E-10	0.7973717	3408	24589	39.3257478
6.12E-10	0.06817408	3408	24589	38.5350726
4.03E-08	0.477880538	3408	24589	30.2658403
7.80E-16	0.7650411	3408	24589	65.560327
2.79E-09	0.02009535	3408	24589	35.5059033
4.65E-14	0.2340463	3408	24589	57.3563835
4.20E-18	0.8233818	3408	24589	76.0490799
1.30E-08	0.5088948	3408	24589	32.5175246
4.25E-09	0.725558704	3408	24589	34.6775494
6.37E-09	0.8100256	3408	24589	33.9000319
4.06E-08	0.5972251	3408	24589	30.2792797
2.28E-08	0.7286178	3408	24589	31.3910625
1.48E-71	0.7085376	3408	24589	335.269887
7.31E-10	0.1556786	3408	24589	38.170973
1.80E-19	0.8952741	3408	24589	82.4272853
3.85E-08	0.6690293	3408	24589	30.3660361
3.78E-08	0.9994095	3408	24589	30.3744095
1.58E-10	0.4712712	3408	24589	41.1839424
4.82E-08	0.7555062	3408	24589	29.9139329
1.16E-21	0.7758015	3408	24589	92.7097733
5.38E-12	0.621179197	3408	24589	47.880037
4.11E-10	0.2980475	3408	24589	39.2857874
7.32E-10	0.7402878	3408	24589	38.130625

2.32E-16	0.006574436	3408	24589	67.9460662
1.64E-08	0.3118101	3408	24589	32.0309657
8.69E-12	0.4967909	3408	24589	46.9268836
1.34E-08	0.6272844	3408	24589	32.4337911
1.20E-12	0.1609807	3408	24589	50.8678429
3.23E-52	0.5916791	3408	24589	239.149807
3.47E-28	0.2020154	3408	24589	123.375467
2.60E-08	0.7337937	3408	24589	31.1384769
2.09E-23	0.5631417	3408	24589	100.88928
3.26E-10	0.2738603	3408	24589	39.7534215
5.81E-19	0.9059384	3408	24589	80.0740898
1.91E-12	0.580177	3408	24589	49.9433607
1.92E-16	0.266137	3408	24589	68.3491609
7.76E-15	0.2743726	3408	24589	60.9325017
2.29E-11	0.1301186	3408	24589	45.0145088
1.74E-56	0.5387889	3408	24589	260.250965
4.15E-16	0.3096317	3408	24589	66.8531004
1.00E-30	0.2937835	3408	24589	135.437711
8.14E-10	0.8292306	3408	24589	37.9391584
8.32E-09	0.1673398	3408	24589	33.3450171
5.64E-11	0.3564824	3408	24589	43.2187659
1.02E-11	0.9653024	3408	24589	46.6014377
3.39E-10	0.1778517	3408	24589	39.6855918
5.04E-09	0.1669823	3408	24589	34.3494622
2.03E-35	0.460341	3408	24589	157.820186
1.71E-27	0.7079955	3408	24589	120.096265
9.36E-13	0.04687456	3408	24589	51.3659082
1.70E-27	0.09995873	3408	24589	120.097579
4.25E-11	0.5651801	3408	24589	43.7851509
2.42E-08	0.1240611	3408	24589	31.2907016
8.35E-13	0.1581384	3408	24589	51.5929099
2.15E-12	0.398128	3408	24589	49.7107795
1.11E-14	0.148322	3408	24589	60.2074254
3.69E-14	0.5568128	3408	24589	57.805025
7.32E-11	0.509159985	3408	24589	42.6920142
9.78E-23	0.02821459	3408	24589	97.6816582
3.31E-10	0.7539488	3408	24589	39.7114628
1.21E-17	0.6396138	3408	24589	73.9400738
3.53E-08	0.9694651	3408	24589	30.5260092
2.34E-11	0.2046182	3408	24589	44.9747578
9.00E-14	0.4315095	3408	24589	56.0446279
5.30E-09	0.9400615	3408	24589	34.2637459
3.68E-08	0.8854694	3408	24589	30.4513856
1.47E-08	0.04410324	3408	24589	32.256626
4.25E-11	0.172044	3408	24589	43.7751487
2.36E-12	0.2283772	3408	24589	49.5568137
1.40E-17	0.4136105	3408	24589	73.6259817
8.20E-41	0.724211	3408	24589	183.794927
2.19E-10	0.3512508	3408	24589	40.5459167
1.99E-09	0.9694037	3408	24589	36.1868842
2.95E-21	0.5881946	3408	24589	90.7722962
2.29E-13	0.3597429	3408	24589	54.1718802
2.74E-16	0.8601146	3408	24589	67.6435538
1.54E-15	0.536598	3408	24589	64.1833068
7.02E-18	0.8879728	3408	24589	75.0404516
6.19E-11	0.9225524	3408	24589	43.0332388
6.19E-18	0.6718191	3408	24589	75.2866776
3.69E-24	0.1668625	3408	24589	104.386487

6.60E-13	0.3331734	3408	24589	52.068928
1.20E-08	0.5472804	3408	24589	32.6530612
4.93E-14	0.4982964	3408	24589	57.252952
5.64E-14	0.02104514	3408	24589	57.0107874
5.41E-11	0.9922192	3408	24589	43.32639
1.46E-09	0.2379455	3408	24589	36.7737932
2.61E-15	0.6057541	3408	24589	63.1226127
1.16E-10	0.2274776	3408	24589	41.7862769
3.21E-08	0.3664984	3408	24589	30.7180126
8.69E-16	0.1227381	3408	24589	65.3444579
2.77E-09	0.1371349	3408	24589	35.5066968
2.00E-08	0.3235574	3408	24589	31.6424837
1.39E-09	0.2911151	3408	24589	36.8915398
5.54E-11	0.4762276	3408	24589	43.2331511
9.17E-10	0.6692977	3408	24589	37.7071046
6.40E-11	0.4989195	3408	24589	42.978769
5.07E-14	0.6479787	3408	24589	57.1857361
1.22E-15	0.2505023	3408	24589	64.6588751
1.61E-22	0.2674422	3408	24589	96.6892609
5.08E-17	0.3392525	3408	24589	71.0404956
2.50E-14	0.4851348	3408	24589	58.6081669
1.55E-08	0.6616177	3408	24589	32.1602557
4.92E-14	0.1960676	3408	24589	57.2366741
3.35E-10	0.9344652	3408	24589	39.6815115
8.41E-101	0.4949787	3408	24589	486.213221
1.41E-08	0.3387722	3408	24589	32.3337178
4.27E-08	0.248421145	3408	24589	30.1821369
5.64E-15	0.7830964	3408	24589	61.5904831
1.16E-09	0.192826	3408	24589	37.2288056
2.74E-11	0.8678425	3408	24589	44.6566089
3.19E-08	0.5760424	3408	24589	30.7702962
7.23E-11	0.7168642	3408	24589	42.7514793
7.99E-11	0.158258	3408	24589	42.5303377
2.36E-13	0.4521212	3408	24589	54.1328304
5.06E-17	0.7766665	3408	24589	71.0486788
6.96E-11	0.5554424	3408	24589	42.8202171
8.16E-11	0.042409904	3408	24589	42.4910837
1.29E-14	0.4885504	3408	24589	59.942369
4.29E-12	0.1649436	3408	24589	48.3373893
3.32E-17	0.151102	3408	24589	71.8924092
1.35E-10	0.1052181	3408	24589	41.5074919
7.89E-17	0.917393	3408	24589	70.1256224
1.18E-08	0.6661335	3408	24589	32.6796571
6.36E-15	0.2068243	3408	24589	61.3233589
7.68E-09	0.1194403	3408	24589	33.4971383
3.37E-08	0.590108	3408	24589	30.6257308
2.61E-14	0.5752755	3408	24589	58.5242668
1.89E-12	0.5837623	3408	24589	49.9294152
1.30E-10	0.1972331	3408	24589	41.5705754
1.60E-08	0.013546712	3408	24589	32.0947075
4.18E-15	0.6784633	3408	24589	62.1287218
2.85E-40	0.77899	3408	24589	181.130564
3.79E-11	0.5386468	3408	24589	44.0181213
1.07E-11	0.6420692	3408	24589	46.5089548
7.20E-12	0.4703353	3408	24589	47.3051103
1.02E-16	0.9016104	3408	24589	69.6733288
2.06E-17	0.1202538	3408	24589	72.8657907
1.80E-24	0.5936644	3408	24589	105.863241

8.71E-09	0.9873442	3408	24589	33.2543089
3.23E-39	0.07893972	3408	24589	176.196836
8.60E-12	0.06866651	3408	24589	46.9331208
5.33E-09	0.0171284	3408	24589	34.2273235
6.61E-42	0.2019776	3408	24589	189.0625
4.67E-08	0.260359	3408	24589	29.9841616
2.03E-19	0.08151516	3408	24589	82.2157627
8.89E-11	0.7089439	3408	24589	42.3064316
4.71E-10	0.2284656	3408	24589	39.0219862
6.43E-15	0.8637232	3408	24589	61.3062352
2.00E-09	0.005808178	3408	24589	36.1430992
3.18E-09	0.7763525	3408	24589	35.2607323
3.61E-13	0.1564381	3657	24589	53.2447742
4.52E-09	0.2786093	3657	24589	34.5536204
2.88E-08	0.2546937	3657	24589	30.9139584
2.00E-09	0.230648	3657	24589	36.1525422
9.31E-29	0.6624921	3657	24589	125.925215
2.83E-08	0.1453697	3657	24589	30.9574868
1.58E-15	0.03337482	3113	24589	64.1882433
6.09E-43	0.3538379	3113	24589	194.574414
1.98E-15	0.08681606	3669	24589	63.6110987
2.51E-10	0.374680231	3669	24589	40.2497611
1.51E-23	0.91861	3669	24589	101.479849
4.61E-13	0.08681606	3669	24589	52.7411511
1.41E-15	0.91861	3669	24589	64.3484445
4.61E-13	0.08681606	3669	24589	52.7411511
1.41E-15	0.91861	3669	24589	64.3484445
8.82E-13	0.1384449	3440	24589	51.4799802
3.55E-11	0.6015959	3440	24589	44.1376471
4.56E-08	0.4624427	3427	24589	30.0600565
1.29E-08	0.0170759	2912	24589	32.5334165
5.31E-18	0.9542232	2912	24589	75.7554773
5.63E-11	0.7545146	3405	24589	43.2163327
1.80E-45	0.8117906	3405	24589	206.299587
3.06E-15	0.5102421	3405	24589	62.8456846
3.74E-304	0.6536588	3405	24589	1716.99787
4.74E-11	0.4113008	3405	24589	43.5703333
5.61E-11	0.7545146	3437	24589	43.1995639
1.32E-44	0.8117906	3437	24589	202.053261
2.05E-12	0.7335325	3437	24589	49.8207739
1.08E-15	0.7081354	3437	24589	64.8822678
0.00E+00	0.6563144	3437	24589	2028.32158
6.86E-13	0.4113008	3437	24589	51.9813966
3.14E-14	0.7545146	3408	24589	58.1612242
3.85E-59	0.8117906	3408	24589	273.510492
1.18E-11	0.7335325	3408	24589	46.3665047
1.14E-20	0.7081354	3408	24589	88.043645
0.00E+00	0.6325739	3408	24589	2727.04061
1.12E-18	0.4113008	3408	24589	78.7235025
1.39E-10	0.7545146	3437	24589	41.4431075
8.03E-56	0.8117906	3437	24589	256.881016
5.32E-13	0.7335325	3437	24589	52.5123343
0.00E+00	0.6750238	3437	24589	2872.32545
2.70E-21	0.4113008	3437	24589	90.9557833
1.53E-20	0.6023826	3437	24589	87.4377177
2.18E-09	0.9829609	2920	24589	36.0580344
2.34E-17	0.6492612	2920	24589	72.7108465
1.47E-18	0.272225	2920	24589	78.3390739

4.45E-08	0.7419215	2920	24589	30.1083324
5.68E-74	0.6750238	2920	24589	350.775727
1.62E-09	0.1181368	2920	24589	36.6228872
8.86E-18	0.18992	1837	24589	75.2943383
1.25E-14	0.4452321	1837	24589	60.4523633
3.18E-10	0.4749707	1837	24589	40.0101039
9.72E-18	0.4271692	1837	24589	75.0863689
7.45E-21	0.7905446	1837	24589	89.9030059
2.82E-10	0.6421125	1837	24589	40.2330436
1.74E-11	0.3536026	1837	24589	45.8357286
5.92E-30	0.18992	3658	24589	131.584954
3.48E-30	0.4452321	3658	24589	132.697374
1.41E-20	0.4749707	3658	24589	87.5109022
3.64E-10	0.775064459	3658	24589	39.5395415
1.87E-24	0.4271692	3658	24589	105.680816
5.51E-17	0.6421125	3658	24589	70.845694
3.06E-33	0.7890625	3658	24589	147.150378
1.11E-19	0.3536026	3658	24589	83.3372597
4.65E-32	0.18992	3660	24589	141.586605
1.97E-29	0.4452321	3660	24589	129.103704
0.00E+00	0.3376883	3660	24589	1953.19607
2.50E-20	0.4749707	3660	24589	86.3631106
1.86E-10	0.775064459	3660	24589	40.8310579
1.71E-23	0.4271692	3660	24589	101.13758
2.44E-16	0.6421125	3660	24589	67.80513
3.75E-34	0.7890625	3660	24589	151.60022
1.97E-19	0.3536026	3660	24589	82.1907765
1.40E-27	0.18992	3656	24589	120.382866
3.37E-29	0.4452321	3656	24589	128.039795
7.25E-17	0.4749707	3656	24589	70.2719777
1.95E-23	0.4271692	3656	24589	100.900632
1.93E-08	0.826270765	3656	24589	31.729163
7.67E-18	0.6421125	3656	24589	74.7955232
6.31E-32	0.7890625	3656	24589	140.982714
0.00E+00	0.4366953	3656	24589	1722.67043
3.78E-19	0.3536026	3656	24589	80.86142
1.86E-25	0.18992	3655	24589	110.366958
2.04E-24	0.4452321	3655	24589	105.503856
1.73E-16	0.4749707	3655	24589	68.5364482
7.95E-21	0.4271692	3655	24589	88.6849288
5.84E-16	0.6421125	3655	24589	66.0468787
1.32E-28	0.7890625	3655	24589	125.197286
3.43E-282	0.3694153	3655	24589	1544.64167
1.94E-17	0.3536026	3655	24589	72.9368097
9.61E-29	0.18992	3657	24589	125.904325
3.09E-30	0.4452321	3657	24589	132.945258
1.44E-19	0.4749707	3657	24589	82.8261743
6.47E-11	0.775064459	3657	24589	42.923412
5.44E-25	0.4271692	3657	24589	108.15348
5.08E-17	0.6421125	3657	24589	71.0510405
4.86E-33	0.7890625	3657	24589	146.247458
5.94E-20	0.3536026	3657	24589	84.6106459
3.32E-28	0.18992	3657	24589	123.319042
2.97E-31	0.4452321	3657	24589	137.791479
0.00E+00	0.3376883	3657	24589	2014.57324
9.77E-21	0.4749707	3657	24589	88.2570253
2.29E-11	0.775064459	3657	24589	44.991142
3.97E-25	0.4271692	3657	24589	108.813533

3.82E-17	0.6421125	3657	24589	71.5624543
3.27E-32	0.7890625	3657	24589	142.325364
1.10E-20	0.3536026	3657	24589	88.0159635
1.23E-28	0.18992	3657	24589	125.394117
2.27E-30	0.4452321	3657	24589	133.580524
1.09E-19	0.4749707	3657	24589	83.3883266
5.25E-12	0.775064459	3657	24589	47.9052933
2.84E-24	0.4271692	3657	24589	104.829083
1.16E-16	0.6421125	3657	24589	69.2947221
1.22E-32	0.7890625	3657	24589	144.34601
1.78E-20	0.3536026	3657	24589	87.0551361
4.59E-33	0.18992	3645	24589	146.392147
3.00E-29	0.4452321	3645	24589	128.242764
1.61E-20	0.4749707	3645	24589	87.2479169
2.15E-23	0.4271692	3645	24589	100.706712
5.47E-16	0.6421125	3645	24589	66.2315579
5.11E-35	0.7890625	3645	24589	155.607764
6.86E-19	0.3536026	3645	24589	79.6759755
9.71E-09	0.4895985	3596	24589	33.0786886
3.08E-70	0.378398	3596	24589	327.999699
1.46E-09	0.4074289	3596	24589	36.7657773
2.76E-26	0.6848751	3405	24589	114.443745
3.19E-56	0.8117906	3405	24589	259.072455
1.52E-08	0.08962633	3405	24589	32.1849538
1.33E-15	0.5102421	3405	24589	64.4949672
4.74E-19	0.8830453	3405	24589	80.4664958
5.38E-09	0.4007902	3405	24589	34.2171332
0.00E+00	0.7414931	3405	24589	2201.14716
5.73E-32	0.6848751	3437	24589	141.323637
2.04E-56	0.8117906	3437	24589	259.954403
3.17E-23	0.7081354	3437	24589	100
0.00E+00	0.7668342	3437	24589	2883.16274
1.28E-10	0.2295834	3437	24589	41.5984149
7.59E-25	0.2607698	3437	24589	107.635358
6.27E-39	0.6848751	3437	24589	174.656648
2.50E-73	0.8117906	3437	24589	344.327408
1.08E-08	0.08962633	3437	24589	32.8562874
1.49E-31	0.7081354	3437	24589	139.388084
2.44E-16	0.4007902	3437	24589	67.8422586
3.13E-08	0.5052898	3437	24589	30.7624615
0.00E+00	0.7555389	3437	24589	4109.89752
8.25E-31	0.2607698	3437	24589	135.816211
4.50E-28	0.6848751	3440	24589	122.835207
3.17E-60	0.8117906	3440	24589	278.572664
0.00E+00	0.7952853	3440	24589	2580.40385
1.06E-21	0.8830453	3440	24589	92.8804463
7.61E-15	0.558967	3440	24589	60.9769539
2.86E-22	0.6023826	3440	24589	95.511199
7.84E-28	0.6325739	2920	24589	122.117013
1.11E-12	0.1422615	2920	24589	51.0695963
3.43E-16	0.7867194	2919	24589	67.321027
5.30E-108	0.69789058	2919	24589	530.444767
3.51E-09	0.758855744	2919	24589	35.0998592
7.28E-47	0.8117906	2920	24589	214.179197
0.00E+00	0.7952853	2920	24589	2342.80004
2.24E-23	0.8016086	2920	24589	100.918885
6.97E-18	0.8830453	2920	24589	75.1480344
1.32E-08	0.1717885	2920	24589	32.5009312

7.66E-09	0.8117906	2920	24589	33.5290546
3.45E-11	0.6492612	2920	24589	44.2780938
4.44E-15	0.272225	2920	24589	62.149217
5.03E-95	0.5691422	2920	24589	460.632773
1.67E-26	0.7911652	2920	24589	115.756952
1.78E-43	0.8117906	2920	24589	197.54148
2.19E-16	0.8830453	2920	24589	68.257256
3.43E-21	0.8169815	2920	24589	90.6486428
0.00E+00	0.7555389	2920	24589	2435.48659
1.87E-12	0.8117906	2915	24589	50.0335261
3.22E-11	0.8169815	2915	24589	44.3639947
4.05E-64	0.8117906	2920	24589	300.303378
1.54E-27	0.8016086	2920	24589	120.699251
1.82E-21	0.8830453	2920	24589	91.9649672
3.84E-09	0.558967	2920	24589	34.9190291
0.00E+00	0.5691422	2920	24589	4980.20543
1.06E-13	0.6085703	2920	24589	55.7939208
4.54E-61	0.8117906	3440	24589	282.754765
2.14E-25	0.7081354	3440	24589	110.194619
3.88E-14	0.2295834	3440	24589	57.7307513
0.00E+00	0.6510075	3440	24589	3350.0734
2.12E-18	0.4113008	3440	24589	77.44
3.83E-08	0.19413672	3629	24589	30.3725936
7.00E-84	0.775064459	3629	24589	397.02981
1.58E-09	0.7032873	3629	24589	36.6248812
7.30E-96	0.1584805	3629	24589	458.267597
6.15E-33	0.6861905	1837	24589	148.668246
4.89E-08	0.5691422	3437	24589	29.8835253
1.36E-13	0.243886	3437	24589	55.212763
4.29E-13	0.6015959	3437	24589	52.927147
1.58E-08	0.6013881	3437	24589	32.0900097
2.56E-14	0.5691422	3435	24589	58.5106998
7.06E-09	0.5691422	3437	24589	33.6853621
5.23E-16	0.243886	3437	24589	66.387718
1.55E-13	0.6015959	3437	24589	54.9442844
4.42E-09	0.6013881	3437	24589	34.6059424
8.84E-11	0.05243086	1635	24589	42.6108881
1.68E-12	0.8811456	1635	24589	50.6369124
2.40E-20	0.05243086	3113	24589	86.6357444
4.45E-14	0.8182224	3113	24589	57.5040515
1.59E-28	0.7772785	3113	24589	125.256384
7.71E-09	0.654731	3113	24589	33.5328349
1.11E-10	0.332541	3113	24589	41.9277691
8.59E-10	0.05562561	3113	24589	37.8598992
6.93E-155	0.8693621	3113	24589	788.448882
3.35E-08	0.8406482	3652	24589	30.6223371
1.03E-22	0.2635976	3652	24589	97.5549289
1.67E-09	0.4186364	3657	24589	36.4974034
1.54E-08	0.5413587	3657	24589	32.1500691
2.77E-15	0.775064459	3657	24589	62.9342002
3.83E-75	0.07835003	3657	24589	352.516727
7.42E-17	0.6236636	3657	24589	70.2839188
2.74E-29	0.4787465	3657	24589	128.481125
1.20E-09	0.5594815	3657	24589	37.1597575
6.46E-09	0.04429508	3611	24589	33.8241207
5.10E-10	0.089082097	3611	24589	38.873509
1.86E-48	0.775064459	3611	24589	220.491444
1.83E-09	0.7782195	3060	24589	36.3902411

3.30E-18	0.7782195	3060	24589	76.6491114
1.37E-17	0.7827071	1634	24589	74.5395674
1.54E-09	0.5209398	1634	24589	36.9160257
2.14E-23	0.7827071	1635	24589	102.468433
2.12E-08	0.3110522	1635	24589	31.6896308
4.65E-09	0.5209398	1635	24589	34.7109569
9.08E-10	0.3325925	3668	24589	37.7346939
4.59E-09	0.6021094	3668	24589	34.5124528
1.30E-13	0.4186364	3657	24589	55.2527178
5.23E-12	0.6518201	3657	24589	47.9347129
6.20E-09	0.5413587	3657	24589	33.9420704
7.07E-17	0.775064459	3657	24589	70.3762899
8.47E-120	0.07835003	3657	24589	584.011114
2.87E-15	0.757319	3657	24589	62.8797561
3.63E-46	0.4787465	3657	24589	209.272679
1.07E-13	0.5594815	3657	24589	55.6542211
2.67E-08	0.4134849	3657	24589	31.0808373
7.02E-09	0.6961545	3657	24589	33.6991962
1.25E-08	0.4186364	3657	24589	32.5501654
1.11E-11	0.6518201	3657	24589	46.4398219
1.88E-87	0.07835003	3657	24589	414.752036
2.13E-10	0.757319	3657	24589	40.5720236
1.49E-35	0.4787465	3657	24589	158.213282
2.95E-08	0.1699366	3657	24589	30.8786049
1.77E-08	0.03290908	3657	24589	31.8718649
1.57E-10	0.5594815	3657	24589	41.1876033
2.23E-15	0.4186364	3657	24589	63.4198382
1.69E-08	0.9883115	3657	24589	31.9500669
1.93E-15	0.6518201	3657	24589	63.6758851
8.52E-11	0.5413587	3657	24589	42.3639302
2.48E-28	0.775064459	3657	24589	123.897598
2.53E-118	0.07835003	3657	24589	575.881925
3.76E-21	0.757319	3657	24589	90.2299287
1.02E-38	0.4787465	3657	24589	173.338808
2.09E-12	0.5594815	3657	24589	49.7485952
1.25E-08	0.4186364	3656	24589	32.5407228
3.82E-08	0.9883115	3656	24589	30.3596074
4.48E-11	0.6518201	3656	24589	43.6675204
3.13E-09	0.775064459	3656	24589	35.2580744
6.60E-15	0.757319	3656	24589	61.2313283
8.02E-44	0.4787465	3656	24589	197.936156
5.93E-78	0.08681954	3656	24589	366.619781
1.39E-08	0.1699366	3656	24589	32.3421167
9.62E-14	0.5594815	3656	24589	55.8758447
1.59E-15	0.4186364	3657	24589	64.0560543
1.33E-12	0.6518201	3657	24589	50.6633831
5.03E-11	0.5413587	3657	24589	43.4461253
1.14E-14	0.775064459	3657	24589	60.1126818
6.21E-147	0.07835003	3657	24589	731.37355
1.29E-10	0.757319	3657	24589	41.575262
3.42E-35	0.4787465	3657	24589	156.499672
4.84E-10	0.5594815	3657	24589	38.9481918
3.04E-08	0.3855907	1858	24589	30.9526019
6.84E-22	0.243886	2919	24589	93.948748
4.74E-14	0.6015959	2919	24589	57.373544
4.28E-11	0.3411878	2919	24589	43.8160011
3.00E-10	0.3460547	2919	24589	39.9735481
3.92E-08	0.243886	3427	24589	30.3108165

8.87E-10	0.6015959	3427	24589	37.7676279
3.13E-13	0.02625116	3427	24589	53.5560411
3.74E-12	0.243886	3405	24589	48.5814221
4.23E-12	0.6015959	3405	24589	48.3994667
2.15E-16	0.02625116	3405	24589	68.1510356
7.57E-11	0.6778339	2915	24589	42.6873216
7.21E-11	0.540732	2915	24589	42.7711782
1.80E-15	0.2554294	2915	24589	63.9899659
8.08E-12	0.5687497	2915	24589	47.1308692
2.20E-09	0.4154466	2850	24589	36
3.14E-36	0.3311294	2850	24589	162.480056
1.72E-145	0.23201	2850	24589	742.138969
1.45E-09	0.9001788	2850	24589	36.8206663
1.64E-13	0.09295363	2850	24589	54.935188
4.35E-09	0.711051	2850	24589	34.6790123
2.22E-08	0.4709452	3425	24589	31.430817
5.93E-11	0.25881	3425	24589	43.1162739
2.61E-19	0.2253211	3425	24589	81.6672822
2.09E-11	0.607098	3425	24589	45.2005934
2.15E-08	0.9433819	3425	24589	31.5161432
4.54E-14	0.8630289	3425	24589	57.417449
1.95E-09	0.541053	3425	24589	36.2174613
1.37E-10	0.2319637	3425	24589	41.4901027
2.66E-09	0.3331408	3425	24589	35.6043721
9.78E-09	0.3549192	3425	24589	33.033046
2.46E-12	0.06535782	3425	24589	49.4569179
7.53E-09	0.02520437	3425	24589	33.5666223
1.47E-09	0.334357	3425	24589	36.7816805
2.35E-11	0.8053286	3425	24589	44.9662807
1.66E-09	0.71359858	3425	24589	36.5359378
1.67E-09	0.805479445	3425	24589	36.5513231
2.08E-18	0.4231676	3425	24589	77.4507567
1.95E-10	0.183697991	3425	24589	40.7673147
2.17E-10	0.1352386	3425	24589	40.5553112
2.03E-09	0.9407224	3425	24589	36.1557597
2.50E-10	0.2890562	3425	24589	40.2581611
1.52E-09	0.1122727	3425	24589	36.6780919
1.37E-08	0.2021163	3425	24589	32.3981516
1.95E-08	0.6401369	3408	24589	31.6876218
1.82E-08	0.951776	3408	24589	31.8544026
3.21E-10	0.690539819	3408	24589	39.7336372
9.60E-09	0.7305944	3408	24589	33.0625
1.48E-09	0.8677485	3408	24589	36.7657335
1.16E-09	0.607098	3408	24589	37.237088
1.92E-09	0.83275214	3408	24589	36.2457331
6.72E-09	0.6494909	3408	24589	33.7768277
7.79E-09	0.6563474	3408	24589	33.4929635
7.23E-09	0.9433819	3408	24589	33.629263
4.14E-11	0.3797726	3408	24589	43.8264527
1.12E-09	0.2221505	3408	24589	37.345679
4.04E-08	0.07249845	3408	24589	30.2571806
2.42E-09	0.8724124	3408	24589	35.8024449
4.11E-10	0.7863122	3408	24589	39.3059553
1.63E-08	0.4949787	3408	24589	32.0423804
3.57E-08	0.6898328	3408	24589	30.5137462
2.03E-11	0.8630289	3408	24589	45.2457448
2.31E-08	0.1983923	3408	24589	31.3645537
1.24E-10	0.5758087	3408	24589	41.6394907

2.71E-08	0.4612497	3408	24589	31.0459967
3.63E-08	0.7541433	3408	24589	30.4842442
2.46E-08	0.1176994	3408	24589	31.2283737
4.09E-08	0.7996588	3408	24589	30.2515947
1.74E-10	0.2319637	3408	24589	40.9844325
3.88E-08	0.9579192	3408	24589	30.3475835
2.24E-21	0.3855813	3408	24589	91.3409335
1.89E-08	0.139173625	3408	24589	31.7477905
1.17E-15	0.08469845	3408	24589	64.7270353
9.70E-09	0.8502468	3408	24589	33.0637761
9.24E-11	0.7557116	3408	24589	42.2381611
9.76E-10	0.3762401	3408	24589	37.587398
4.51E-11	0.01829795	3408	24589	43.6562983
2.11E-09	0.6517416	3408	24589	36.0610946
1.23E-16	0.07043293	3408	24589	69.2736095
1.15E-09	0.2975696	3408	24589	37.2467632
6.76E-13	0.645190392	3408	24589	52.0664097
4.22E-09	0.5972251	3408	24589	34.6956584
5.71E-09	0.4553014	3408	24589	34.0970719
1.66E-09	0.8123383	3408	24589	36.5204014
2.27E-12	0.02520437	3408	24589	49.5909829
6.10E-09	0.334357	3408	24589	33.972737
4.24E-11	0.1522102	3408	24589	43.7887695
1.50E-11	0.6245568	3408	24589	45.8477631
5.25E-14	0.5387889	3408	24589	57.1481894
1.39E-14	0.2965314	3408	24589	59.7647929
2.39E-08	0.4147125	3408	24589	31.2914978
2.40E-20	0.549986	3408	24589	86.4978336
1.91E-08	0.2415363	3408	24589	31.7317249
3.01E-08	0.1773616	3408	24589	30.8429586
4.50E-15	0.71359858	3408	24589	62.0304608
1.18E-12	0.4231676	3408	24589	50.9189255
1.37E-09	0.183697991	3408	24589	36.9228204
3.69E-08	0.3323239	3408	24589	30.4510317
3.93E-09	0.7750694	3408	24589	34.8424587
1.78E-09	0.1352386	3408	24589	36.3678659
2.78E-09	0.8385585	3408	24589	35.5127918
1.11E-08	0.9407224	3408	24589	32.7879784
8.29E-09	0.6327147	3408	24589	33.3797509
2.64E-10	0.3990455	3408	24589	40.1802408
6.76E-12	0.3645722	3408	24589	47.4192094
3.29E-11	0.5690497	3408	24589	44.2923261
3.23E-09	0.6808787	3408	24589	35.2237425
1.43E-08	0.8732244	3408	24589	32.2925886
2.66E-08	0.3269572	3408	24589	31.0907686
1.55E-09	0.05031796	3408	24589	36.6602528
2.26E-11	0.8067567	3408	24589	45.0546953
8.23E-12	0.1122727	3408	24589	47.0256258
3.93E-10	0.336793984	3408	24589	39.3558511
1.74E-08	0.7424731	3408	24589	31.8981027
2.11E-08	0.3010102	3408	24589	31.5340758
5.76E-10	0.1904025	3408	24589	38.6237423
4.64E-12	0.540323	3408	24589	48.1393948
2.28E-10	0.08661555	3408	24589	40.4569063
1.90E-12	0.013546712	3408	24589	49.9899122
1.29E-10	0.7615364	3408	24589	41.5756293
4.14E-08	0.811157	3408	24589	30.2274728
3.50E-08	0.9391883	3408	24589	30.5628179

3.97E-10	0.5589411	3408	24589	39.3505825
2.59E-08	0.721523	3408	24589	31.1394058
1.55E-10	0.079421	3408	24589	41.2138575
2.63E-09	0.1822449	3621	24589	35.6150595
6.85E-11	0.351133587	3621	24589	42.8305145
2.69E-08	0.759966579	3621	24589	31.0573077
1.29E-90	0.775064459	3621	24589	431.360947
3.04E-11	0.7032873	3621	24589	44.4297872
5.95E-09	0.8023058	3621	24589	34.0277778
7.57E-33	0.513035814	3621	24589	145.334514
5.03E-94	0.1584805	3621	24589	448.867415
6.89E-13	0.4273163	2910	24589	52.0436665
3.90E-10	0.02660269	2910	24589	39.4276373
4.81E-08	0.01466897	2910	24589	29.9660222
1.29E-08	0.9432102	2910	24589	32.5509125
2.04E-15	0.6284361	1555	24589	64.3487604
6.30E-10	0.7466535	1555	24589	38.7107619
1.33E-12	0.6637	1555	24589	51.1388995
1.81E-150	0.5446947	1555	24589	858.075707
1.40E-17	0.9910141	1555	24589	74.5886387
4.53E-12	0.807893	1555	24589	48.6491906
1.06E-13	0.6284361	1554	24589	56.2719448
5.21E-12	0.6637	1554	24589	48.3615202
6.08E-143	0.5446947	1554	24589	804.942084
5.07E-15	0.9910141	1554	24589	62.4621807
1.64E-13	0.807893	1554	24589	55.3871731
1.38E-08	0.6284361	1555	24589	32.5614431
1.22E-50	0.3945208	1555	24589	241.100598
3.25E-08	0.7243522	2910	24589	30.716645
1.40E-08	0.9854416	2910	24589	32.3802456
6.62E-120	0.891364234	2910	24589	595.75293
9.11E-15	0.509026381	2910	24589	60.7286404
8.64E-09	0.13119795	2910	24589	33.3387358
8.58E-13	0.393384219	2910	24589	51.6115159
1.00E-09	0.918462136	2910	24589	37.56358
3.07E-08	0.2043159	3657	24589	30.7823374
1.03E-11	0.141256	3657	24589	46.5994054
8.18E-23	0.6624921	3657	24589	97.9131009
7.67E-09	0.4625829	3657	24589	33.5163554
1.32E-08	0.03159648	3657	24589	32.4416998
2.97E-08	0.2043159	3657	24589	30.8563917
2.09E-10	0.3356709	3657	24589	40.5985945
9.01E-29	0.6624921	3657	24589	126.058474
1.75E-10	0.1453697	3657	24589	40.96
3.14E-08	0.2309808	3657	24589	30.7545114
1.47E-10	0.3257321	3657	24589	41.2991658
2.90E-08	0.7669483	2920	24589	30.9376324
3.04E-22	0.272225	2920	24589	95.6167315
7.71E-19	0.6024405	2920	24589	79.686979
7.97E-170	0.6536588	2920	24589	883.424174
1.39E-14	0.04924214	2920	24589	59.865396
4.72E-08	0.303982557	3395	24589	29.9681515
1.85E-08	0.7085246	3395	24589	31.8080671
5.45E-09	0.3248189	3395	24589	34.1917785
2.84E-09	0.4160417	3395	24589	35.4574587
2.65E-08	0.303982557	3427	24589	31.0939175
3.32E-08	0.1584107	3427	24589	30.6516077
3.74E-09	0.8816794	3427	24589	34.9276162

4.75E-09	0.3281697	3427	24589	34.4762098
4.36E-15	0.8816794	3060	24589	62.1592089
4.01E-08	0.2260438	3060	24589	30.3051169
1.06E-08	0.6103398	3060	24589	32.9064388
6.18E-09	0.05862274	3060	24589	33.9691176
4.73E-12	0.4327529	3060	24589	48.1799308
2.47E-43	0.05060102	3060	24589	196.541357
1.06E-48	0.1103228	3060	24589	222.919086
7.05E-21	0.7772785	3113	24589	89.1719647
3.03E-15	0.4926303	3113	24589	62.880697
9.39E-71	0.8279627	3113	24589	332.986797
1.49E-10	0.4111954	3113	24589	41.3199833
1.25E-08	0.4455029	3440	24589	32.5663477
2.44E-08	0.6163993	3440	24589	31.2628422
1.92E-08	0.1564939	3440	24589	31.7312923
1.37E-08	0.9189208	3440	24589	32.4023669
1.60E-08	0.4445813	3440	24589	32.0755619
3.97E-09	0.07798494	3440	24589	34.823672
1.17E-09	0.750113	3440	24589	37.2431796
4.27E-08	0.7864262	3440	24589	30.1573744
3.22E-08	0.3864186	3440	24589	30.6965904
8.68E-09	0.6179577	3440	24589	33.2732856
1.63E-08	0.036667744	3440	24589	32.0427484
3.11E-08	0.7894731	3440	24589	30.7776543
1.13E-08	0.7921098	3440	24589	32.7606563
1.60E-08	0.655661	3440	24589	32.0840434
1.59E-08	0.310328	3440	24589	32.0916839
1.53E-08	0.7547988	3440	24589	32.1669299
1.72E-08	0.7372655	3440	24589	31.9418919
8.65E-09	0.8268682	3440	24589	33.2922313
1.75E-08	0.6955804	3657	24589	31.9065692
1.45E-09	0.390674	3657	24589	36.788319
7.25E-10	0.4625829	3657	24589	38.1625598
2.09E-16	0.7727473	3657	24589	68.1531895
1.70E-09	0.1757424	3657	24589	36.4791015
5.48E-11	0.6888583	3657	24589	43.2961926
3.65E-10	0.1757424	3657	24589	39.5061684
1.22E-09	0.8483297	3657	24589	37.1244375
1.34E-08	0.8030009	3657	24589	32.4210232
1.70E-08	0.9947334	3656	24589	31.9765243
5.51E-10	0.9186967	3656	24589	38.6954646
6.87E-13	0.08697448	3656	24589	51.9780048
7.66E-09	0.921807	3019	24589	33.5712813
3.61E-15	0.461439308	3019	24589	62.5814682
3.35E-17	0.6015959	3437	24589	71.9067429
1.21E-08	0.02625116	3437	24589	32.6293351
6.75E-12	0.3411878	3437	24589	47.4473575
5.13E-09	0.459553	3437	24589	34.3265868
1.38E-13	0.394562	3437	24589	55.1675139
3.87E-08	0.2580997	3437	24589	30.3474234
1.04E-08	0.3379967	2920	24589	32.9556003
5.13E-67	0.1723529	2920	24589	315.15621
2.76E-08	0.4417659	3408	24589	31.0211409
1.77E-12	0.6015959	3408	24589	50.0671193
2.82E-11	0.02625116	3408	24589	44.6083955
2.72E-08	0.1908816	3408	24589	31.0465266
2.58E-08	0.3909289	3408	24589	31.1515404
8.02E-09	0.781161	3435	24589	33.4718034

1.82E-10	0.781161	3435	24589	40.8637126
1.68E-09	0.121097392	2872	24589	36.5636771
6.15E-12	0.23201	2872	24589	47.6783413
2.39E-12	0.5733369	2872	24589	49.5584741
1.27E-08	0.5203351	2872	24589	32.5595338
3.91E-08	0.1350375	3657	24589	30.32437
5.88E-10	0.1564002	3405	24589	38.5631727
4.23E-08	0.3411878	3405	24589	30.1679661
7.49E-09	0.1564002	3395	24589	33.5381594
2.84E-09	0.3226773	3427	24589	35.4876969
4.24E-11	0.6913884	3427	24589	43.7784796
5.06E-11	0.3362097	3427	24589	43.4488858
1.03E-25	0.4366953	3648	24589	111.59619
7.39E-10	0.8483297	1833	24589	38.3225037
1.65E-08	0.8030009	1833	24589	32.1600647
9.18E-11	0.2407138	1833	24589	42.4733068
2.61E-10	0.8483297	3657	24589	40.1663554
2.36E-09	0.8030009	3657	24589	35.8349298
8.34E-10	0.2407138	3657	24589	37.8760857
4.29E-09	0.8483297	3656	24589	34.6533574
3.95E-08	0.8030009	3656	24589	30.3085062
2.02E-10	0.2407138	3656	24589	40.6774995
6.42E-09	0.8483297	3656	24589	33.8618978
3.80E-09	0.2087646	3656	24589	34.8907009
3.76E-08	0.8286304	3656	24589	30.4056156
2.91E-08	0.8483297	3658	24589	30.9046221
5.79E-09	0.2634047	3658	24589	34.063832
2.55E-13	0.8483297	3657	24589	53.921727
1.07E-11	0.8030009	3657	24589	46.4917347
5.43E-13	0.2634047	3657	24589	52.4225649
1.92E-09	0.8483297	3656	24589	36.2453145
7.82E-09	0.6888583	3656	24589	33.5013243
2.31E-10	0.2407138	3656	24589	40.413869
2.92E-08	0.7469891	1857	24589	31.0203777
1.86E-14	0.4005586	1857	24589	59.6290381
6.98E-10	0.2253211	3425	24589	38.2409143
7.93E-10	0.2319637	3425	24589	37.9914446
3.59E-08	0.8074352	3425	24589	30.4977198
1.26E-08	0.583923	3425	24589	32.5508782
3.08E-08	0.186053	3425	24589	30.7878632
2.91E-28	0.4231676	3425	24589	123.759509
2.16E-09	0.3645722	3425	24589	36.0078104
9.41E-09	0.2838762	1856	24589	33.248244
5.44E-14	0.2097382	1856	24589	57.4527557
9.84E-09	0.2838762	1857	24589	33.1637906
7.51E-35	0.426781	1857	24589	158.117109
9.46E-14	0.2097382	1857	24589	56.3281289
1.29E-10	0.451820276	2919	24589	41.6578041
4.33E-119	0.69789058	2919	24589	591.168558
1.33E-08	0.509026381	2919	24589	32.4716155
1.46E-10	0.451820276	2919	24589	41.372506
6.96E-128	0.69789058	2919	24589	640.187782
2.49E-10	0.758855744	2919	24589	40.3214165
8.19E-10	0.451820276	2919	24589	37.9474791
3.46E-127	0.69789058	2919	24589	636.31924
1.48E-09	0.758855744	2919	24589	36.790668
3.99E-09	0.451820276	2919	24589	34.8732027
7.26E-122	0.69789058	2919	24589	606.520573

1.50E-10	0.758855744	2919	24589	41.3289272
1.48E-08	0.03091026	3395	24589	32.2451569
7.97E-09	0.005661866	3656	24589	33.4464661
4.58E-18	0.6102493	3656	24589	75.8315973
5.48E-11	0.005661866	3656	24589	43.2715568
1.76E-22	0.6102493	3656	24589	96.4179373
1.66E-09	0.9567595	3656	24589	36.5257565
1.69E-14	0.005661866	3657	24589	59.3316289
9.69E-15	1	3657	24589	60.4761892
1.35E-08	0.707619046	3657	24589	32.4250785
5.24E-10	0.4118011	3657	24589	38.7912708
6.27E-32	0.6102493	3657	24589	140.869129
3.34E-14	0.005661866	3657	24589	58.0140436
5.62E-26	0.6102493	3657	24589	112.81133
9.22E-12	0.9567595	3657	24589	46.7899609
1.71E-13	0.005661866	3654	24589	54.7087671
1.54E-10	0.4118011	3654	24589	41.2010973
3.63E-32	0.6102493	3654	24589	142.008945
7.84E-13	0.9567595	3654	24589	51.6801006
3.21E-65	0.560033071	3629	24589	302.810485
1.77E-08	0.789491614	3629	24589	31.8739177
2.52E-43	0.560033071	3629	24589	195.56206
5.41E-09	0.2128449	3629	24589	34.2144329
3.10E-45	0.560033071	3629	24589	204.814988
4.42E-15	0.560033071	3618	24589	62.0373145
2.29E-08	0.7218513	3618	24589	31.3816637
4.06E-63	0.560033071	3629	24589	292.414307
1.45E-08	0.789491614	3629	24589	32.2624
1.81E-18	0.560033071	3614	24589	77.7265296
2.55E-08	0.5978111	3668	24589	31.1488849
1.35E-12	0.3552312	3668	24589	50.608048
1.51E-20	0.4926303	3668	24589	87.3775586
5.38E-16	0.8279627	3668	24589	66.2712616
2.47E-19	0.4111954	3668	24589	81.7211535
5.79E-09	0.91861	3668	24589	34.0396207
3.56E-08	0.5978111	3668	24589	30.50458
3.73E-13	0.3552312	3668	24589	53.1789855
6.79E-22	0.4926303	3668	24589	93.6557319
2.06E-16	0.8279627	3668	24589	68.1856589
5.81E-20	0.4111954	3668	24589	84.6513148
1.47E-10	0.7866188	3649	24589	41.3215342
5.23E-20	0.3504358	3649	24589	84.8626239
4.64E-11	0.3552312	3668	24589	43.5951031
6.50E-19	0.4926303	3668	24589	79.7826948
7.85E-22	0.8279627	3668	24589	93.4031914
2.39E-17	0.4111954	3668	24589	72.506608
1.54E-08	0.1541933	3668	24589	32.1313163
3.91E-09	0.3552312	3669	24589	34.829698
5.37E-15	0.4926303	3669	24589	61.6475103
1.04E-27	0.8279627	3669	24589	120.972189
3.25E-09	0.5230474	3669	24589	35.1870073
2.83E-10	0.4111954	3669	24589	40.0179623
1.40E-10	0.3552312	3652	24589	41.4062207
1.38E-18	0.4926303	3652	24589	78.2580431
2.69E-16	0.8279627	3652	24589	67.6254018
1.16E-17	0.4111954	3652	24589	73.9706505
2.20E-09	0.91861	3652	24589	35.9511368
2.25E-08	0.3552312	3669	24589	31.4141355

3.23E-13	0.4926303	3669	24589	53.4500892
3.82E-08	0.06928652	3669	24589	30.3523894
8.64E-26	0.8279627	3669	24589	111.935656
3.34E-09	0.5230474	3669	24589	35.144585
1.79E-09	0.4111954	3669	24589	36.3687278
9.10E-09	0.4823112	3669	24589	33.1831188
4.64E-09	0.3552312	3652	24589	34.5120754
9.75E-17	0.4926303	3652	24589	69.6815572
9.86E-22	0.8693621	3652	24589	92.9235594
1.41E-15	0.4111954	3652	24589	64.3194504
3.16E-09	0.1541933	3652	24589	35.2477524
1.25E-11	0.3552312	3668	24589	46.1779752
2.76E-20	0.4926303	3668	24589	86.1632573
2.68E-22	0.8279627	3668	24589	95.5605998
5.29E-18	0.4111954	3668	24589	75.5677559
1.82E-25	0.3311294	3112	24589	110.694108
3.30E-114	0.23201	3112	24589	561.421043
4.94E-12	0.9001788	3112	24589	48.0968713
4.48E-11	0.09295363	3112	24589	43.6902346
3.80E-08	0.763423761	3410	24589	30.3805722
4.21E-165	0.199460386	3410	24589	839.380437
4.90E-12	0.866123264	3410	24589	48.0640139
4.02E-13	0.718913	2920	24589	53.1116992
5.88E-12	0.521059	2920	24589	47.752366
1.47E-08	0.9125216	3113	24589	32.2541553
1.23E-09	0.6015959	3437	24589	37.1368755
2.94E-26	0.02296135	3437	24589	114.261541
4.91E-21	0.0554487	3437	24589	89.7225208
6.09E-46	0.1081297	3437	24589	208.532985
6.83E-09	0.705567	3437	24589	33.7536547
2.14E-08	0.8129019	3437	24589	31.5103969
0.00E+00	0.7471014	3437	24589	1927.75201
5.35E-29	0.6318545	3437	24589	127.202071
4.49E-18	0.6015959	3437	24589	75.9187517
5.43E-11	0.02625116	3437	24589	43.2938281
4.11E-11	0.3411878	3437	24589	43.8630976
4.97E-09	0.459553	3437	24589	34.3735212
4.14E-08	0.2580997	3437	24589	30.2091834
1.37E-15	0.92274	3437	24589	64.4018828
1.83E-08	0.6015959	3437	24589	31.8211703
5.32E-25	0.394562	3437	24589	108.337542
4.31E-09	0.6015959	2903	24589	34.7049309
1.09E-13	0.4901444	2903	24589	55.767757
3.37E-28	0.6015959	3437	24589	123.397938
4.93E-18	0.3411878	3437	24589	75.6840904
2.14E-08	0.5377169	3437	24589	31.5142154
3.40E-38	0.08682414	3437	24589	171.13101
5.96E-10	0.9782094	3437	24589	38.5611683
1.35E-20	0.6015959	3437	24589	87.6544929
4.24E-22	0.02296135	3437	24589	94.730558
7.94E-09	0.8124902	3437	24589	33.4546228
1.83E-13	0.02625116	3437	24589	54.6479279
3.59E-18	0.8830453	3437	24589	76.355621
1.86E-41	0.1081297	3437	24589	186.881389
1.95E-13	0.149302	3437	24589	54.4908045
1.29E-09	0.3969441	3437	24589	37.0218908
2.30E-10	0.6649604	3437	24589	40.4379697
4.32E-294	0.7471014	3437	24589	1644.18932

4.44E-25	0.6318545	3437	24589	108.705439
1.02E-13	0.6015959	3440	24589	55.8207761
2.27E-11	0.02625116	3440	24589	45.0346005
2.21E-10	0.2727777	3440	24589	40.5044008
3.34E-09	0.3757269	3440	24589	35.1575521
2.66E-10	0.6015959	3437	24589	40.1407382
5.06E-12	0.4193558	3437	24589	47.9952538
5.19E-17	0.08682414	3437	24589	70.9785834
1.87E-27	0.6015959	3437	24589	119.904475
3.70E-18	0.3411878	3437	24589	76.3270056
1.70E-08	0.5377169	3437	24589	31.9765155
2.55E-08	0.3969441	3437	24589	31.1751964
6.29E-38	0.08682414	3437	24589	169.923575
4.50E-10	0.9782094	3437	24589	39.1112572
5.94E-10	0.6015959	3405	24589	38.5632009
9.40E-10	0.3411878	3405	24589	37.658488
2.27E-09	0.394562	3405	24589	35.9079766
2.37E-12	0.6015959	3440	24589	49.4834255
3.80E-09	0.02625116	3440	24589	34.9252383
2.85E-09	0.136493	3440	24589	35.477095
5.33E-24	0.6015959	3437	24589	103.621666
2.77E-08	0.5377169	3437	24589	31.006265
5.49E-33	0.08682414	3437	24589	146.179952
3.18E-11	0.6015959	3408	24589	44.3130493
3.74E-09	0.3411878	3408	24589	34.9225258
5.53E-11	0.6015959	3408	24589	43.2625567
4.41E-18	0.6015959	3405	24589	75.9590191
2.88E-08	0.8124902	3405	24589	30.9475816
4.17E-12	0.02625116	3405	24589	48.3755924
1.07E-15	0.0554487	3405	24589	64.9362254
1.10E-09	0.705567	3405	24589	37.359797
8.93E-09	0.3969441	3405	24589	33.244938
3.22E-09	0.6649604	3405	24589	35.2404488
3.75E-37	0.04924214	3405	24589	166.154173
3.04E-18	0.4113008	3405	24589	76.6995285
1.03E-22	0.6318545	3405	24589	97.6072987
3.57E-18	0.6015959	3437	24589	76.3990594
1.72E-11	0.3411878	3437	24589	45.5989338
4.22E-18	0.4193558	3437	24589	76.0746135
4.78E-32	0.08682414	3437	24589	141.73313
1.42E-11	0.6015959	3440	24589	45.9884627
1.45E-08	0.4605136	3440	24589	32.2626601
2.93E-23	0.6015959	3437	24589	100.225606
1.44E-08	0.5377169	3437	24589	32.2976507
3.53E-33	0.08682414	3437	24589	147.069258
6.36E-11	0.6015959	3440	24589	42.9494245
1.18E-09	0.6015959	3405	24589	37.2017956
1.67E-10	0.3411878	3405	24589	41.0594778
7.93E-09	0.394562	3405	24589	33.4553178
3.79E-13	0.6015959	2910	24589	53.2670705
6.19E-09	0.3411878	2910	24589	33.9979485
9.98E-24	0.2206093	2910	24589	102.625085
6.32E-13	0.8532131	2910	24589	52.2214635
5.75E-51	0.03687993	2910	24589	234.493327
1.19E-09	0.1926217	2910	24589	37.2334035
1.59E-09	0.3537619	2910	24589	36.6416169
4.26E-09	0.1485768	2910	24589	34.7271731
6.79E-13	0.6314262	3657	24589	51.934276

8.06E-53	0.6692781	3657	24589	241.67151
3.75E-08	0.2093288	3657	24589	30.4215227
6.09E-13	0.6314262	3657	24589	52.1604938
1.16E-51	0.6692781	3657	24589	235.986193
2.05E-08	0.2093288	3657	24589	31.5677791
1.20E-10	0.6314262	3657	24589	41.6980032
7.76E-48	0.6692781	3657	24589	217.361044
8.76E-09	0.2093288	3657	24589	33.2763594
6.90E-10	0.6314262	3657	24589	38.223996
1.71E-45	0.6692781	3657	24589	206.068105
3.93E-08	0.12238909	3113	24589	30.3334201
4.05E-08	0.4901444	3113	24589	30.2521244
1.71E-10	0.9542232	2920	24589	41.0634017
9.04E-10	0.02660269	2920	24589	37.7833829
5.49E-11	0.9542232	2920	24589	43.3168166
1.42E-13	0.1517689	2920	24589	55.1708282
9.61E-12	0.1422615	2920	24589	46.7658682
1.00E-10	0.9542232	2920	24589	42.1078742
7.48E-14	0.9542232	2920	24589	56.5003573
2.29E-08	0.1181368	2920	24589	31.395743
6.46E-25	0.9542232	2911	24589	108.260808
1.66E-14	0.9542232	2920	24589	59.5158323
1.12E-21	0.9542232	2912	24589	92.9430873
1.21E-24	0.9542232	2912	24589	106.994826
2.11E-09	0.9542232	2919	24589	36.101568
3.36E-15	0.9542232	2920	24589	62.6883614
3.87E-10	0.1181368	2920	24589	39.4286681
2.16E-14	0.089082097	3622	24589	58.8895491
1.09E-08	0.157501017	3622	24589	32.8231471
1.45E-59	0.775064459	3622	24589	274.780583
4.21E-08	0.1058572	3622	24589	30.1605204
1.15E-13	0.089082097	3622	24589	55.5591993
4.73E-08	0.157501017	3622	24589	29.955942
8.17E-50	0.775064459	3622	24589	227.125746
3.65E-08	0.1058572	3622	24589	30.4749063
4.52E-32	0.9430083	3595	24589	141.696266
1.37E-08	0.25949	2920	24589	32.4228861
1.46E-09	0.6888583	3656	24589	36.7669937
9.28E-10	0.2624235	3656	24589	37.6768556
7.89E-09	0.6888583	3657	24589	33.4320589
1.12E-08	0.2624235	3657	24589	32.7774189
1.10E-13	0.854288	2872	24589	55.7317023
1.28E-08	0.2567604	2872	24589	32.5370042
8.65E-22	0.361577	2872	24589	93.4677569
1.29E-08	0.8177095	2910	24589	32.5637732
9.13E-13	0.7462093	2910	24589	51.4720325
3.18E-08	0.523899	3656	24589	30.743428
3.31E-17	0.7930332	3656	24589	71.8612115
2.30E-09	0.523899	3656	24589	35.8843839
1.21E-08	0.5651716	3656	24589	32.5947088
2.91E-10	0.9597736	3656	24589	39.95627
3.70E-09	0.3834124	3656	24589	34.9351454
1.09E-18	0.7930332	3656	24589	78.7049414
5.53E-10	0.2651259	3657	24589	38.6926171
3.43E-13	0.7930332	3657	24589	53.331016
1.81E-08	0.211141	3656	24589	31.8139296
8.09E-16	0.7930332	3656	24589	65.4031701
2.26E-08	0.7277332	3657	24589	31.4051169

4.86E-08	0.09570216	3656	24589	29.8907471
8.25E-09	0.4369854	3656	24589	33.3532307
7.97E-13	0.6697062	2872	24589	51.7579683
5.27E-09	0.1325562	2872	24589	34.2832647
3.66E-08	0.2714133	2872	24589	30.485431
1.09E-13	0.9485019	3657	24589	55.6214667
3.16E-10	0.7950025	3657	24589	39.7882923
4.03E-09	0.6060483	3657	24589	34.7859225
0.00E+00	0.3376883	3657	24589	1937.03849
1.06E-32	0.9142559	3657	24589	144.624182
4.12E-21	0.8586778	3657	24589	90.0513646
7.31E-42	0.2675457	3657	24589	188.433558
5.39E-14	0.9485019	3657	24589	57.0349003
6.49E-10	0.7950025	3657	24589	38.3688168
2.95E-32	0.9142559	3657	24589	142.554287
1.22E-20	0.8586778	3657	24589	87.8317467
3.37E-40	0.2675457	3657	24589	180.487644
3.73E-13	0.9485019	3657	24589	53.1869936
1.44E-08	0.7764596	3657	24589	32.2839384
3.98E-23	0.8586778	3657	24589	99.4408733
7.62E-49	0.2675457	3657	24589	222.283423
3.88E-38	0.5352036	3657	24589	170.559513
1.20E-13	0.9485019	3657	24589	55.4431599
9.08E-11	0.7950025	3657	24589	42.2565903
1.58E-31	0.9142559	3657	24589	139.035374
5.84E-20	0.8586778	3657	24589	84.6769683
3.19E-40	0.2675457	3657	24589	180.587033
4.34E-13	0.9485019	3657	24589	52.8748776
6.66E-10	0.7950025	3657	24589	38.3259704
1.41E-08	0.6060483	3657	24589	32.3238701
1.54E-34	0.9142559	3657	24589	153.384283
2.13E-22	0.8586778	3657	24589	96.0246966
1.93E-08	0.826270765	3657	24589	31.6874826
4.41E-45	0.2675457	3657	24589	204.099867
0.00E+00	0.3811009	3657	24589	1828.52344
9.69E-14	0.9485019	3657	24589	55.8772358
4.98E-09	0.7764596	3657	24589	34.3720925
6.20E-21	0.8586778	3657	24589	89.220917
1.31E-10	0.826270765	3657	24589	41.5138866
5.24E-40	0.2675457	3657	24589	179.543295
5.97E-33	0.5352036	3657	24589	145.822767
1.47E-09	0.4926303	3440	24589	36.780424
1.54E-08	0.4094052	3440	24589	32.1471764
3.91E-09	0.07036569	3440	24589	34.8319133
1.29E-08	0.05548399	3629	24589	32.4993304
1.62E-62	0.775064459	3629	24589	289.489892
4.55E-09	0.4177328	3629	24589	34.5322963
2.07E-83	0.1584805	3629	24589	394.511624
2.48E-08	0.2501725	3659	24589	31.2222133
4.78E-08	0.04521373	3656	24589	29.9081528
4.32E-08	0.2786093	3657	24589	30.1381059
5.44E-17	0.6624921	3657	24589	70.889498
3.86E-08	0.1453697	3657	24589	30.3463852
5.41E-10	0.181092	3657	24589	38.7200984
2.33E-34	0.5405471	2920	24589	153.284401
1.68E-27	0.5976606	2920	24589	120.455722
9.29E-12	0.7224173	2650	24589	46.9030209
3.44E-09	0.383457	2650	24589	35.1696789

1.02E-28	0.4452321	3657	24589	125.778393
2.80E-10	0.009699467	3657	24589	40.0356461
4.53E-08	0.3255086	3657	24589	30.0391604
0.00E+00	0.3751148	3657	24589	1722.10197
3.31E-08	0.6618633	3113	24589	30.6730144
4.36E-10	0.2478573	3113	24589	39.1926074
9.35E-20	0.02296135	3440	24589	83.7409321
1.23E-41	0.272225	3440	24589	187.736413
9.80E-33	0.1081297	3440	24589	144.974617
0.00E+00	0.6536588	3440	24589	2781.66734
1.13E-27	0.01424971	3440	24589	120.951307
7.75E-21	0.02296135	3440	24589	88.8222862
5.07E-40	0.272225	3440	24589	179.910595
2.59E-34	0.1081297	3440	24589	152.511065
1.73E-16	0.8169815	3440	24589	68.612159
0.00E+00	0.6616386	3440	24589	2653.2318
7.75E-26	0.01424971	3440	24589	112.280069
2.79E-21	0.02296135	3408	24589	90.8915571
3.23E-42	0.272225	3408	24589	190.545447
7.19E-15	0.8016086	3408	24589	61.0820114
1.45E-33	0.1081297	3408	24589	148.98561
1.49E-29	0.01424971	3408	24589	129.861987
0.00E+00	0.6696571	3408	24589	2631.18458
2.70E-08	0.858076371	2971	24589	31.0820278
3.58E-69	0.199460386	2971	24589	325.634118
1.14E-16	0.6893922	3427	24589	69.4301488
1.46E-08	0.08931447	3427	24589	32.2525614
3.03E-08	0.3375332	3427	24589	30.8354367
3.06E-19	0.6893922	3427	24589	81.333333
2.52E-08	0.08931447	3427	24589	31.204196
3.80E-08	0.3881486	3408	24589	30.4013669
2.27E-09	0.3325216	3408	24589	35.9428798
4.36E-09	0.6675795	3669	24589	34.6116117
8.45E-09	0.9597736	3669	24589	33.3272698
1.08E-12	0.91861	3669	24589	51.0290196
9.93E-09	0.6675795	3669	24589	32.9899548
1.60E-08	0.7930332	3669	24589	32.046516
3.35E-10	0.374680231	3669	24589	39.6772275
1.74E-08	0.06928652	3669	24589	31.9181203
1.21E-15	0.91861	3669	24589	64.6077518
2.31E-15	0.1501544	3629	24589	63.3271308
7.74E-12	0.23201	3629	24589	47.1588958
9.55E-11	0.1501544	1855	24589	42.4033983
1.72E-28	0.5883691	1855	24589	126.731674
2.30E-10	0.2375104	1855	24589	40.6524565
3.84E-16	0.1501544	3629	24589	66.9235218
1.06E-11	0.23201	3629	24589	46.5160342
1.09E-10	0.311901299	2872	24589	41.9555568
2.69E-23	0.084659042	2872	24589	100.639812
3.88E-08	0.2904061	3113	24589	30.345753
4.57E-11	0.942933569	2918	24589	43.7087917
2.58E-16	0.393384219	2918	24589	67.8972998
2.35E-08	0.256481587	2918	24589	31.3697895
2.66E-08	0.983503068	2918	24589	31.1005148
4.46E-08	0.9194335	2918	24589	30.0943303
3.94E-08	0.8274438	2919	24589	30.3498482
8.31E-13	0.509026381	2919	24589	51.6758045
1.32E-16	0.5495162	3656	24589	69.0773219

4.51E-08	0.9778804	3113	24589	30.0822386
3.02E-11	0.9623813	3660	24589	44.4304781
4.63E-13	0.620768	3660	24589	52.7651357
1.12E-11	0.7413714	3660	24589	46.4072499
2.33E-12	0.9623813	3658	24589	49.5264617
1.42E-09	0.7334489	3658	24589	36.8283604
7.90E-16	0.5366666	3658	24589	65.4894518
1.64E-08	0.3460664	3658	24589	32.0184202
2.25E-08	0.2158949	3657	24589	31.4221688
3.61E-21	0.2313926	3657	24589	90.278216
6.64E-31	0.5504017	3657	24589	136.157577
1.77E-08	0.7996774	3629	24589	31.8947192
1.74E-10	0.6709372	1464	24589	41.350558
2.39E-108	0.426781	1464	24589	581.005753
1.04E-12	0.9910141	1464	24589	51.6893034
2.62E-12	0.919311	1464	24589	49.8029469
8.43E-10	0.6814552	3645	24589	37.8922694
6.08E-09	0.5286803	3645	24589	33.958131
7.49E-10	0.3356709	3645	24589	38.0860227
3.22E-11	0.7334489	3645	24589	44.3230116
1.56E-11	0.6557615	3060	24589	45.8149464
4.92E-49	0.868188347	3060	24589	224.538343
9.38E-33	0.9792604	2971	24589	145.566959
3.47E-51	0.199460386	2971	24589	235.361464
8.44E-10	0.1340407	2971	24589	37.8860024
4.17E-08	0.6478516	2971	24589	30.2221818
1.72E-23	0.2031953	2971	24589	101.483459
1.42E-09	0.4012588	2920	24589	36.9020909
1.15E-08	0.03563705	2920	24589	32.7499372
1.42E-14	0.4922644	2920	24589	59.8202577
1.08E-08	0.3519825	2920	24589	32.8948148
1.37E-12	0.1274998	2920	24589	50.7180657
1.49E-13	0.676472	2920	24589	55.1051777
2.91E-65	0.231298	2920	24589	306.072301
3.47E-08	0.9028575	3668	24589	30.5678239
8.28E-19	0.8825668	3668	24589	79.2867699
1.76E-15	0.4538615	2850	24589	64.0450413
3.36E-08	0.1036123	2850	24589	30.6573038
1.77E-14	0.9654672	2850	24589	59.3884519
7.25E-10	0.6147169	2848	24589	38.2021933
2.11E-09	0.9001788	2848	24589	36.1041214
7.83E-10	0.02625116	3427	24589	38.0417624
1.26E-08	0.02625116	3427	24589	32.5427616
1.22E-10	0.02625116	3395	24589	41.6967771
1.18E-08	0.02625116	3427	24589	32.6979297
1.09E-10	0.1715785	3434	24589	41.9022702
8.83E-11	0.272225	2920	24589	42.3755758
9.56E-57	0.6723383	2920	24589	263.143609
1.83E-10	0.6340851	3653	24589	40.8769805
5.62E-14	0.7930332	3656	24589	56.9586772
1.39E-10	0.7930332	3656	24589	41.4266011
4.02E-12	0.7930332	3656	24589	48.4416
7.20E-09	0.06524318	3656	24589	33.6479427
1.43E-16	0.7930332	3653	24589	68.9319604
2.18E-10	0.5117673	3653	24589	40.5380546
2.04E-10	0.7930332	3656	24589	40.6268093
9.14E-11	0.7930332	3656	24589	42.2671006
9.00E-18	0.7930332	3669	24589	74.4586119

7.63E-10	0.2054162	3669	24589	38.093237
8.62E-09	0.7930332	3656	24589	33.3058887
1.49E-09	0.9854416	2919	24589	36.7658218
1.66E-133	0.69789058	2919	24589	671.821367
1.51E-18	0.509026381	2919	24589	78.2935697
1.98E-12	0.569142177	2919	24589	49.9394786
1.23E-11	0.3473874	2919	24589	46.3010715
3.59E-08	0.4094052	3408	24589	30.4828407
1.05E-10	0.5317189	3408	24589	41.9843371
6.75E-13	0.2843118	2920	24589	52.05532
2.87E-08	0.4328542	3427	24589	30.950251
1.21E-19	0.01201562	3427	24589	83.2588558
4.97E-11	0.3757269	3427	24589	43.4797013
1.33E-10	0.7950025	3657	24589	41.5012265
1.63E-08	0.7764596	3657	24589	32.0285616
6.25E-32	0.9142559	3657	24589	141.00312
2.29E-20	0.1342929	3657	24589	86.549918
2.29E-08	0.826270765	3657	24589	31.3583309
1.63E-37	0.2675457	3657	24589	167.593441
1.76399999983	0.3694153	3657	24589	1761.11055
2.10E-09	0.7950025	3657	24589	36.056856
3.41E-08	0.7764596	3657	24589	30.5912502
2.82E-33	0.9142559	3657	24589	147.397964
2.74E-20	0.1342929	3657	24589	86.1990174
4.18E-08	0.826270765	3657	24589	30.1846564
3.89E-40	0.2675457	3657	24589	180.170453
1.85E-306	0.3694153	3657	24589	1707.86992
4.67E-11	0.2510457	2920	24589	43.6504874
2.20E-10	0.7216335	2920	24589	40.5525648
8.94E-09	0.807236802	2920	24589	33.249545
2.73E-08	0.6913884	3668	24589	31.0325482
4.75E-09	0.4118544	3579	24589	34.4329682
1.40E-25	0.378398	3579	24589	111.018891
5.58E-45	0.5540907	2920	24589	204.994817
4.70E-08	0.3554139	2920	24589	29.9850232
9.92E-10	0.3191918	3629	24589	37.537892
1.16E-09	0.8082135	3629	24589	37.2009932
1.36E-09	0.9001788	3629	24589	36.9099803
2.06E-09	0.3411878	3437	24589	36.1286287
9.63E-12	0.6893922	3437	24589	46.7340929
9.52E-09	0.9782094	3437	24589	33.109343
5.09E-11	0.9186967	3636	24589	43.4161523
4.39E-09	0.1762558	3636	24589	34.6020761
1.06E-13	0.4356637	3636	24589	55.6960479
4.55E-10	7.32536E-06	3636	24589	39.0846537
3.78E-08	0.7085246	2919	24589	30.4204135
2.31E-36	0.814491	2919	24589	162.984028
5.94E-11	0.2478573	2919	24589	43.1693826
1.48E-08	0.7085246	3440	24589	32.2417187
5.01E-11	0.3281697	3440	24589	43.4592949
5.51E-10	0.4160417	3440	24589	38.7373015
2.69E-09	0.7085246	3668	24589	35.5735613
2.02E-08	0.4111954	3668	24589	31.6071623
2.39E-08	0.4160417	3668	24589	31.2924004
6.10E-13	0.7085246	3668	24589	52.1860003
5.86E-13	0.2478573	3668	24589	52.265243
9.72E-11	0.7085246	3668	24589	42.1208375
8.33E-09	0.4111954	3668	24589	33.3605826

2.44E-10	0.7085246	3653	24589	40.2991398
2.22E-11	0.6294878	3653	24589	45.0550952
2.84E-08	0.5230474	3653	24589	30.9534004
2.79E-25	0.5791832	3653	24589	109.541102
4.13E-09	0.1541933	3653	24589	34.7393886
8.63E-12	0.7085246	3652	24589	46.9232811
2.87E-11	0.4111954	3652	24589	44.5422089
2.41E-08	0.1541933	3652	24589	31.2572034
2.99E-15	0.7085246	3668	24589	62.8056804
4.31E-14	0.2478573	3668	24589	57.4661811
1.57E-08	0.6370166	3656	24589	32.1081106
6.16E-09	0.4901444	3656	24589	33.9354991
8.25E-09	0.7696064	1837	24589	33.5218501
6.78E-18	0.9142559	1837	24589	75.8428221
4.23E-10	0.1342929	1837	24589	39.3985442
1.02E-24	0.2675457	1837	24589	108.458894
9.85E-175	0.3694153	1837	24589	993.93798
3.99E-20	0.6400052	1634	24589	86.6846912
1.89E-08	0.3045696	1634	24589	31.9278502
1.01E-12	0.4099849	1634	24589	51.6401929
1.29E-08	0.3703725	1634	24589	32.6752117
1.36E-19	0.497214104	2871	24589	83.2032144
7.54E-16	0.6943213	2871	24589	65.7649571
1.96E-24	0.9599597	2871	24589	105.963507
2.34E-72	0.497214104	2850	24589	342.802685
4.18E-08	0.538684381	2850	24589	30.2448456
5.12E-48	0.8481721	2850	24589	220.052157
2.61E-16	0.178839999	2850	24589	67.8839449
6.94E-11	0.614814665	2850	24589	42.8488815
8.24E-19	0.497214104	2871	24589	79.5208339
1.17E-12	0.6943213	2871	24589	51.0204082
4.79E-19	0.9805183	2871	24589	80.6257654
1.06E-25	0.199460386	2871	24589	111.99571
2.07E-08	0.5228966	3656	24589	31.5681794
8.82E-09	0.3470269	3130	24589	33.2707323
3.16E-08	0.9115366	3130	24589	30.7440645
1.32E-08	0.1912981	3130	24589	32.4596649
1.55E-08	0.1746905	3112	24589	32.1619709
1.81E-08	0.1746905	2872	24589	31.8607703
1.20E-08	0.6829127	2872	24589	32.6984705
3.49E-09	0.2714133	2872	24589	35.0881001
4.41E-08	0.393382	2872	24589	30.1281998
3.34E-10	0.041099991	3387	24589	39.7103096
1.36E-08	0.759966579	3387	24589	32.3913241
9.85E-166	0.199460386	3387	24589	843.436639
1.66E-14	0.866123264	3387	24589	59.4221073
1.10E-12	0.5048031	2871	24589	51.1347194
1.56E-55	0.8329985	2871	24589	257.286283
9.17E-13	0.5048031	2871	24589	51.4898079
1.45E-55	0.8329985	2871	24589	257.517726
6.79E-12	0.23201	3629	24589	47.3884726
6.35E-09	0.9001788	3629	24589	33.8884885
5.07E-09	0.4556535	3590	24589	34.3452444
3.77E-15	0.6000575	3618	24589	62.3819333
5.66E-26	0.442581	3618	24589	112.849763
4.55E-08	0.4099742	3113	24589	30.0449021
3.87E-08	0.99103	3113	24589	30.3661676
6.12E-12	0.2067485	3660	24589	47.6019144

2.19E-08	0.2093288	3660	24589	31.4359782
1.68E-32	0.3504358	3660	24589	143.690949
1.20E-11	0.2067485	3657	24589	46.2585413
6.27E-09	0.9894595	3657	24589	33.8984341
1.25E-40	0.3941554	3657	24589	182.562369
7.55E-10	0.2067485	3658	24589	38.0779298
1.74E-31	0.3504358	3658	24589	138.888472
5.60E-13	0.2067485	3657	24589	52.3687971
3.86E-08	0.9303444	3657	24589	30.3404031
4.56E-11	0.2093288	3657	24589	43.6511437
2.21E-43	0.3504358	3657	24589	195.763314
4.00E-12	0.2067485	3657	24589	48.448508
2.29E-08	0.9303444	3657	24589	31.3578503
1.11E-10	0.2093288	3657	24589	41.8477582
1.32E-47	0.3941554	3657	24589	216.258624
3.72E-08	0.437854999	3617	24589	30.4285756
2.60E-208	0.199460386	3617	24589	1085.21078
1.83E-14	0.866123264	3617	24589	59.2079792
4.86E-08	0.7259683	2650	24589	29.9419087
3.17E-20	0.278799	2920	24589	86.1557713
4.02E-20	0.9001788	2920	24589	85.6072508
2.06E-11	0.136152	2920	24589	45.2465393
1.91E-09	0.6505274	3113	24589	36.2873851
1.43E-09	0.759966579	3410	24589	36.8403956
8.71E-10	0.066296299	3410	24589	37.8088136
8.53E-178	0.199460386	3410	24589	912.594213
5.86E-16	0.866123264	3410	24589	66.1464569
1.35E-08	0.3874392	3659	24589	32.4087711
6.78E-09	0.8700489	3659	24589	33.7549495
4.96E-38	0.3694153	3659	24589	170.059609
1.85E-08	0.2546937	3657	24589	31.8008015
1.61E-13	0.6624921	3657	24589	54.821899
1.10E-09	0.2546937	3657	24589	37.3511952
8.13E-11	0.3356709	3657	24589	42.4732493
1.71E-20	0.7334489	3657	24589	87.1153861
8.25E-09	0.04966651	3657	24589	33.3601854
1.11E-12	0.592875	3437	24589	51.0444914
3.34E-08	0.5591629	3437	24589	30.6350689
4.17E-09	0.6006206	3437	24589	34.7173136
1.65E-21	0.813136	2910	24589	92.1841779
3.72E-23	0.2568659	2910	24589	99.9414606
4.36E-08	0.6645423	2910	24589	30.1546995
2.10E-08	0.420072	2910	24589	31.6063355
3.01E-13	0.813136	2910	24589	53.7023796
2.61E-08	0.2568659	2910	24589	31.1470604
1.94E-08	0.7216335	2920	24589	31.7364107
1.49E-11	0.4556535	1858	24589	46.1408523
2.20E-08	0.3672198	1856	24589	31.569801
1.29E-12	0.426781	1856	24589	51.0421155
2.13E-09	0.9464139	2912	24589	36.0778739
6.16E-22	0.6152937	2912	24589	94.1737373
7.15E-14	0.3538379	2912	24589	56.5628549
1.57E-31	0.899515043	2649	24589	140.043505
1.01E-61	0.868188347	3060	24589	287.620605
9.84E-58	0.868188347	2910	24589	268.194451
4.21E-09	0.393384219	2910	24589	34.7467704
5.71E-10	0.059048271	2910	24589	38.6750149
7.25E-43	0.868188347	2919	24589	194.512339

3.92E-08	0.007238693	2919	24589	30.3536205
4.23E-08	0.059048271	2919	24589	30.2114372
7.82E-39	0.868188347	2914	24589	174.963929
2.21E-23	0.868188347	2903	24589	101.030921
6.14E-53	0.868188347	2918	24589	244.266841
1.83E-10	0.059048271	2918	24589	40.9337382
8.80E-10	0.6829127	1635	24589	38.03431
6.94E-15	0.6893922	3405	24589	61.1670703
1.33E-11	0.4193558	3405	24589	46.0616668
3.10E-20	0.08682414	3405	24589	85.9648031
1.56E-11	0.6893922	3427	24589	45.734375
1.19E-11	0.4231676	3427	24589	46.2858834
1.15E-10	0.6893922	3395	24589	41.7838273
1.63E-08	0.8876176	3395	24589	32.0459079
7.08E-14	0.6893922	3437	24589	56.4936644
2.78E-28	0.394562	3437	24589	123.83261
8.60E-21	0.6893922	3440	24589	88.629716
3.35E-10	0.7683962	3427	24589	39.6851121
1.65E-08	0.32751	3427	24589	32.0213287
8.11E-11	0.01201562	3427	24589	42.5005752
1.18E-08	0.01230707	3395	24589	32.6588245
1.37E-10	0.6505274	3427	24589	41.4402251
2.55E-11	0.6505274	3427	24589	44.8113442
2.00E-08	0.1318567	3427	24589	31.6478667
2.40E-22	0.394562	3405	24589	95.8774563
3.96E-08	0.1064863	2912	24589	30.3233778
2.08E-08	0.839008419	2912	24589	31.599197
8.20E-35	0.856304835	2912	24589	155.534026
6.52E-26	0.89380615	3060	24589	112.890625
2.63E-09	0.9598482	3653	24589	35.5884844
6.12E-10	0.3885463	3655	24589	38.4834676
1.28E-15	0.5299422	3655	24589	64.5285594
2.51E-08	0.23201	3629	24589	31.1961335
1.61E-14	0.6000575	3629	24589	59.4514787
1.61E-08	0.23201	2850	24589	32.1050475
1.89E-09	0.05514094	2850	24589	36.3346873
2.37E-10	0.23201	3629	24589	40.3724268
2.82E-08	0.23201	3629	24589	30.955224
9.48E-14	0.5399101	3629	24589	55.8736714
8.02E-09	0.1862931	3629	24589	33.4317609
8.34E-14	0.775064459	1858	24589	56.584644
3.69E-18	0.775064459	2850	24589	76.4957999
2.69E-27	0.5285557	2850	24589	119.56228
1.47E-18	0.775064459	1634	24589	79.1528329
1.06E-20	0.775064459	1838	24589	89.1465972
2.62E-69	0.775064459	3579	24589	323.524465
3.51E-08	0.8336057	3579	24589	30.5157804
4.45E-54	0.775064459	3590	24589	248.09578
6.28E-17	0.8063847	3590	24589	70.5812525
1.20E-20	0.775064459	2850	24589	88.1704358
1.84E-11	0.775064459	1634	24589	45.7911697
2.40E-183	0.426781	1634	24589	1088.29964
7.89E-09	0.9526192	1634	24589	33.684205
2.91E-21	0.9910141	1634	24589	92.1446099
1.33E-16	0.919311	1634	24589	69.8897878
2.06E-70	0.775064459	3618	24589	328.851752
1.03E-08	0.03808541	3618	24589	32.9272635
3.17E-12	0.513035814	3618	24589	48.921103

3.78E-92	0.1584805	3618	24589	439.373986
3.93E-11	0.775064459	1633	24589	44.2723461
2.39E-191	0.426781	1633	24589	1150.49852
1.33E-08	0.8458112	1633	24589	32.6123594
6.18E-12	0.6637	1633	24589	47.9827638
8.18E-22	0.9910141	1633	24589	94.8189063
5.83E-16	0.919311	1633	24589	66.842291
6.57E-20	0.775064459	3582	24589	84.4220873
1.43E-38	0.775064459	1578	24589	178.138126
5.24E-32	0.775064459	1570	24589	145.030049
3.85E-08	0.775064459	3618	24589	30.3674919
2.53E-54	0.775064459	1858	24589	257.360337
8.70E-09	0.485581221	1858	24589	33.4252858
3.71E-18	0.2128449	1858	24589	77.0513169
1.36E-16	0.775064459	2850	24589	69.1652711
9.63E-30	0.7785489	2850	24589	131.28406
6.27E-61	0.775064459	1635	24589	295.011118
2.10E-64	0.775064459	3590	24589	298.938127
6.00E-23	0.8063847	3590	24589	98.6725038
2.77E-54	0.775064459	1858	24589	257.099811
7.11E-09	0.485581221	1858	24589	33.8235723
7.44E-19	0.2128449	1858	24589	80.3620755
6.70E-28	0.394562	3435	24589	122.019849
3.83E-09	0.374680231	3653	24589	34.8753495
6.55E-10	0.09625365	3653	24589	38.3526989
8.82E-17	0.91861	3653	24589	69.9085664
6.88E-12	0.374680231	3113	24589	47.4328217
1.34E-15	0.4114712	3113	24589	64.5181
3.83E-08	0.2203823	1858	24589	30.5005165
2.20E-19	0.1696178	1858	24589	82.882816
3.11E-19	0.07835003	3651	24589	81.2676171
3.50E-15	0.5648098	3651	24589	62.5002063
3.69E-08	0.07835003	3642	24589	30.4326187
1.07E-11	0.6236636	3642	24589	46.5077463
2.98E-83	0.07835003	3656	24589	393.640801
1.15E-08	0.757319	3656	24589	32.7064377
2.35E-43	0.4787465	3656	24589	195.685225
5.54E-13	0.5594815	3656	24589	52.3791293
1.18E-09	0.4134849	3656	24589	37.1690799
5.04E-11	0.5208262	3656	24589	43.4239052
4.90E-13	0.07835003	3657	24589	52.6413339
8.55E-11	0.7849178	3657	24589	42.3899245
2.24E-09	0.5676371	3657	24589	35.9252725
2.51E-08	0.1453697	3657	24589	31.1771294
2.10E-15	0.061871057	1247	24589	64.6003094
2.17E-14	0.061871057	1247	24589	59.7577537
5.64E-14	0.061871057	1247	24589	57.838988
8.55E-13	0.061871057	1247	24589	52.2620702
7.00E-13	0.061871057	1247	24589	52.6249552
1.91E-13	0.061871057	1247	24589	55.3263305
5.90E-09	0.066296299	3387	24589	34.0322894
1.40E-150	0.199460386	3387	24589	756.803118
2.05E-12	0.866123264	3387	24589	49.8106467
8.13E-09	0.066296299	3387	24589	33.3964181
9.71E-166	0.199460386	3387	24589	843.396678
2.26E-12	0.866123264	3387	24589	49.634123
3.62E-09	0.066296299	3387	24589	34.978258
1.34E-220	0.199460386	3387	24589	1170.601

3.96E-16	0.866123264	3387	24589	66.9278319
3.16E-15	0.620768	3657	24589	62.7294646
1.67E-08	0.7334489	3657	24589	31.9754188
3.93E-08	0.9000225	3657	24589	30.2950988
5.42E-09	0.3460664	3657	24589	34.2016103
2.24E-42	0.66089	2920	24589	192.175506
1.84E-13	0.1723529	2920	24589	54.6824767
3.15E-38	0.66089	2920	24589	172.059947
9.00E-12	0.1723529	2920	24589	46.9076011
1.46E-10	0.216647	2910	24589	41.3644989
5.66E-154	0.69789058	2910	24589	790.065741
3.34E-19	0.509026381	2910	24589	81.3434458
4.48E-14	0.569142177	2910	24589	57.5292404
4.69E-13	0.9464053	3656	24589	52.7121109
2.44E-11	3.56541E-06	3656	24589	44.8926962
2.95E-12	0.5286803	3657	24589	49.0739639
2.29E-16	0.5366666	3657	24589	67.9528544
9.87E-10	0.3460664	3657	24589	37.5355881
1.13E-25	0.5286803	3657	24589	111.386531
1.75E-10	0.8072368	3657	24589	40.9677845
2.45E-08	0.3460664	3657	24589	31.2160817
6.73E-13	0.5513121	3657	24589	52.0146924
6.37E-09	0.906234549	3112	24589	33.9068077
1.00E-08	0.7818264	3060	24589	33.0044447
6.18E-10	0.3139577	3060	24589	38.518643
2.12E-13	0.8966083	3374	24589	54.3260989
3.17E-08	0.4391171	3579	24589	30.7412421
5.57E-52	0.378398	3579	24589	237.692097
1.36E-10	0.73718	3579	24589	41.4492191
2.59E-10	0.4391171	3580	24589	40.19007
3.62E-55	0.378398	3580	24589	253.363548
6.46E-10	0.73718	3580	24589	38.369132
3.86E-13	0.069832104	1247	24589	53.8599754
4.90E-11	0.03367365	1247	24589	43.9893072
2.74E-09	0.03367365	1245	24589	35.8914301
4.25E-09	0.074667873	1247	24589	35.0028948
2.44E-10	0.2909342	1247	24589	40.7235791
1.02E-10	0.074667873	1247	24589	42.5286148
2.92E-09	0.1913807	1832	24589	35.6014479
3.42E-30	0.6279017	1832	24589	135.152518
6.38E-13	0.1913807	3654	24589	52.1216406
4.04E-08	0.5782611	3654	24589	30.2700033
2.16E-61	0.6579098	3654	24589	283.809693
3.86E-08	0.1913807	3638	24589	30.3499245
2.66E-17	0.6279017	3638	24589	72.2884818
2.96E-12	0.1913807	3649	24589	49.0167278
2.52E-45	0.6579098	3649	24589	205.207142
6.00E-13	0.1913807	3653	24589	52.1991742
6.21E-45	0.6579098	3653	24589	203.322378
9.31E-11	0.1913807	3655	24589	42.1798011
3.93E-41	0.6279017	3655	24589	184.970979
1.46E-11	0.1913807	3652	24589	45.8464195
1.21E-40	0.6579098	3652	24589	182.686244
1.54E-11	0.1913807	3655	24589	45.7964953
1.16E-49	0.6279017	3655	24589	226.286499
1.36E-12	0.1913807	3648	24589	50.5854055
2.21E-25	0.6279017	3648	24589	110.04462
2.24E-10	0.3957849	3112	24589	40.5202727

3.84E-40	0.9001788	3112	24589	180.993172
2.13E-09	0.6236636	3657	24589	36.0393228
5.92E-11	0.4787465	3657	24589	43.0977216
7.20E-50	0.08681954	3657	24589	227.385399
3.13E-08	0.6236636	3656	24589	30.743428
1.64E-27	0.1239017	3656	24589	120.043164
4.81E-08	0.2089857	1835	24589	30.0435864
1.67E-18	0.3504358	1835	24589	78.7163093
1.86E-08	0.6215583	2909	24589	31.7994234
4.79E-08	0.6836065	2909	24589	29.9595002
1.25E-32	0.4005586	1857	24589	147.091253
2.20E-09	0.111758	1857	24589	36.1281822
2.77E-08	0.672368486	3621	24589	30.9827918
3.82E-82	0.207431801	3621	24589	388.11107
3.74E-08	0.6593517	3112	24589	30.4111435
4.14E-09	0.2839079	3112	24589	34.7322214
6.10E-09	0.9275363	3427	24589	33.9793887
5.97E-09	0.2189447	3060	24589	34.0414724
3.53E-15	0.3538379	3060	24589	62.5938174
1.63E-08	0.1610175	2911	24589	32.0745708
8.13E-22	0.6152937	2911	24589	93.6541374
9.11E-10	0.757319	1836	24589	37.8984181
1.08E-38	0.4787465	1836	24589	177.372586
1.33E-62	0.08681954	1836	24589	301.251595
4.44E-08	0.1699366	1836	24589	30.2045625
3.41E-10	0.5594815	1836	24589	39.857994
6.78E-10	0.1665916	3618	24589	38.3020352
4.70E-11	0.600356914	3618	24589	43.5373547
1.49E-08	0.8146224	3657	24589	32.2132944
3.67E-08	0.9240067	2872	24589	30.4853687
1.22E-17	0.248384613	2872	24589	74.0737095
2.21E-33	0.01234413	2872	24589	148.649029
9.29E-10	0.6304036	2910	24589	37.7243569
1.84E-26	0.3538379	2910	24589	115.604978
4.29E-26	0.2635976	3668	24589	113.409678
2.66E-27	0.2635976	3668	24589	119.080665
5.70E-16	5.32359E-06	3656	24589	66.162426
6.58E-11	0.7847738	3656	24589	42.8725654
6.86E-10	0.4465308	3656	24589	38.2703786
4.71E-12	3.56541E-06	3657	24589	48.1320022
5.47E-17	3.56541E-06	3656	24589	70.8469898
5.73E-11	0.7847738	3656	24589	43.1941632
8.85E-10	0.4465308	3656	24589	37.7312185
5.94E-51	0.248384613	2872	24589	234.622264
1.95E-11	0.2878522	2872	24589	45.3624488
3.45E-99	0.69789058	2912	24589	482.977285
3.56E-15	0.509026381	2912	24589	62.6160346
3.70E-08	0.1344402	2912	24589	30.4805442
1.46E-10	0.569142177	2912	24589	41.3961953
3.54E-94	0.69789058	2919	24589	456.298919
5.02E-10	0.569142177	2919	24589	38.9432092
4.79E-08	0.577639703	2919	24589	29.9607395
3.76E-132	0.69789058	2919	24589	664.108781
1.69E-09	0.924405468	2919	24589	36.5210624
1.05E-84	0.69789058	2911	24589	406.527967
2.05E-11	0.509026381	2911	24589	45.2923321
1.18E-09	0.569142177	2911	24589	37.251135
2.49E-14	0.74540469	2911	24589	58.68715

1.61E-71	0.919838985	2919	24589	338.030219
1.12E-12	0.509026381	2919	24589	51.0776748
1.75E-18	0.856304835	2919	24589	77.996307
2.05E-71	0.891364234	2912	24589	337.390802
1.10E-115	0.891364234	2919	24589	572.685228
3.64E-13	0.509026381	2919	24589	53.3124199
7.71E-12	0.932848445	2919	24589	47.203462
1.47E-62	0.378398	3595	24589	289.729305
4.62E-08	0.9063149	3595	24589	30.0085211
5.96E-09	0.73718	3595	24589	34.003293
7.09E-69	0.378398	3596	24589	321.280958
1.10E-08	0.1040441	3596	24589	32.8127779
5.15E-10	0.4074289	3596	24589	38.8494379
9.23E-61	0.378398	3595	24589	280.872771
2.31E-09	0.73718	3595	24589	35.8660051
1.34E-33	0.378398	3595	24589	149.010303
1.39E-08	0.4074289	3595	24589	32.3613004
7.57E-13	0.3297634	2914	24589	51.8567944
2.96E-09	0.6003709	2914	24589	35.4369088
8.44E-30	0.3297634	2920	24589	131.528428
9.33E-11	0.521059	3395	24589	42.2384006
9.17E-10	0.8876176	3371	24589	37.7143279
3.52E-11	0.8876176	3652	24589	44.1476022
1.16E-09	0.8876176	3668	24589	37.2215727
2.56E-10	0.8876176	3408	24589	40.2058737
1.90E-08	0.07036569	3408	24589	31.7367584
2.99E-09	0.01466897	3395	24589	35.3816066
7.66E-12	0.01466897	3652	24589	47.1757481
2.65E-08	0.01466897	3653	24589	31.0849657
4.42E-28	0.285213607	2872	24589	123.297
3.17E-08	0.05583933	2920	24589	30.778428
2.34E-08	0.6743328	2920	24589	31.36
3.31E-34	0.5405471	2920	24589	152.633993
8.92E-09	0.3362097	3440	24589	33.2285349
1.38E-16	0.8825668	3668	24589	69.0124934
6.70E-12	0.8825668	3652	24589	47.4359997
3.03E-11	0.7509825	3657	24589	44.4142255
1.83E-08	0.4787465	3656	24589	31.8115229
4.98E-33	0.08681954	3656	24589	146.168284
1.64E-15	0.4063966	2870	24589	64.173471
1.56E-13	0.513035814	2870	24589	55.025466
7.75E-13	0.4063966	2870	24589	51.8095698
2.59E-37	0.248384613	2870	24589	167.680829
3.42E-15	0.233056	2871	24589	62.6946696
2.91E-26	0.248384613	2871	24589	114.661693
1.44E-10	0.2567604	2871	24589	41.3909948
3.03E-25	0.361577	2871	24589	109.819387
2.38E-09	0.2093288	3642	24589	35.8032118
1.11E-25	0.5648098	3642	24589	111.454766
4.38E-10	0.2093288	3656	24589	39.1380196
3.54E-18	0.3941554	3656	24589	76.3545168
3.69E-09	0.2093288	3657	24589	34.9791287
9.24E-48	0.3504358	3657	24589	216.943464
9.24E-18	0.2132418	3657	24589	74.4596478
1.56E-113	0.199460386	3629	24589	551.02654
2.25E-10	0.866123264	3629	24589	40.4646159
1.41E-134	0.199460386	3629	24589	663.791117
4.58E-09	0.866123264	3629	24589	34.5325092

9.59E-11	0.765157645	3620	24589	42.1685114
1.19E-15	0.386730356	3620	24589	64.6621399
4.60E-10	0.934658432	3620	24589	39.052871
3.22E-09	0.388522378	3620	24589	35.2176648
9.19E-189	0.426781	1578	24589	1141.39449
1.08E-15	0.9910141	1578	24589	65.6280739
4.12E-15	0.919311	1578	24589	62.8894854
1.71E-153	0.426781	1569	24589	878.197517
2.56E-12	0.9532574	1569	24589	49.7667495
9.03E-18	0.9910141	1569	24589	75.4911327
1.14E-40	0.426781	1857	24589	187.215791
3.85E-08	0.111758	1857	24589	30.487099
1.37E-13	0.9910141	1857	24589	55.6025234
1.05E-190	0.426781	1634	24589	1144.9148
2.39E-08	0.8458112	1634	24589	31.4602955
1.39E-09	0.6637	1634	24589	37.1047094
7.98E-23	0.9910141	1634	24589	99.7011954
6.32E-15	0.919311	1634	24589	61.9504372
1.09E-16	0.426781	1858	24589	70.1058672
1.45E-08	0.7773756	1858	24589	32.4222983
7.84E-184	0.426781	1577	24589	1103.22123
2.43E-14	0.9910141	1577	24589	59.2834532
1.51E-14	0.919311	1577	24589	60.2236306
2.80E-187	0.426781	1579	24589	1129.56779
2.54E-15	0.9910141	1579	24589	63.8633209
3.54E-15	0.919311	1579	24589	63.1887345
2.13E-21	0.426781	1858	24589	92.4877251
2.60E-145	0.426781	1558	24589	820.028221
2.26E-10	0.7466535	1558	24589	40.7708312
1.15E-17	0.9910141	1558	24589	74.9958469
4.12E-12	0.807893	1558	24589	48.829078
4.36E-21	0.426781	1858	24589	90.9746747
7.06E-11	0.071472203	2871	24589	42.8247749
5.33E-11	0.023968756	2871	24589	43.4125599
3.38E-08	0.2634047	3657	24589	30.6136563
2.98E-11	0.980374	3657	24589	44.4799503
3.02E-11	0.01877804	3629	24589	44.4444444
9.64E-11	0.5209398	3621	24589	42.1516338
3.27E-16	0.2410625	3621	24589	67.2650204
1.91E-38	0.73683	3621	24589	172.027935
1.57E-43	0.3504358	3657	24589	196.480739
2.59E-20	0.2132418	3657	24589	86.2451696
3.45E-38	0.9001788	2850	24589	171.914894
2.41E-16	0.9001788	2850	24589	68.0408292
1.99E-10	0.3394191	3060	24589	40.7301109
2.57E-09	0.5687497	3060	24589	35.7084338
1.06E-09	0.9001788	2920	24589	37.4668521
1.22E-32	0.9217142	2872	24589	145.074713
3.00E-14	0.2867618	2920	24589	58.3173912
4.37E-29	0.389375456	3042	24589	127.996947
4.96E-08	0.2833523	3042	24589	29.8939113
4.89E-08	0.7774215	3042	24589	29.9051681
1.71E-08	0.8878073	3657	24589	31.9478576
2.05E-08	0.902200939	3657	24589	31.5886593
9.69E-09	0.826270765	3651	24589	33.0591584
3.66E-09	0.509026381	2919	24589	35.0148389
1.17E-20	0.5285557	3629	24589	87.8637697
1.02E-09	0.08416069	3629	24589	37.4825449

6.59E-17	0.5285557	3629	24589	70.4332645
3.13E-09	0.08807603	3629	24589	35.2673979
4.62E-08	0.7477201	3657	24589	29.9886621
2.99E-09	0.07036569	3669	24589	35.3525312
6.13E-11	0.07036569	3440	24589	43.0774624
6.13E-11	0.07036569	3440	24589	43.0774624
1.35E-09	0.07036569	3440	24589	36.9530274
3.34E-08	0.3127712	3440	24589	30.6396293
9.95E-09	0.8471343	3440	24589	33.0305125
4.32E-10	0.07036569	3440	24589	39.2083435
1.86E-08	0.1513039	3395	24589	31.8146699
3.51E-50	0.461439308	2848	24589	230.792324
6.38E-09	0.6888583	1834	24589	34.0533899
4.95E-09	0.248384613	3017	24589	34.4071258
1.20E-89	0.248384613	3374	24589	428.167123
9.74E-10	0.072177504	3374	24589	37.5887056
9.69E-44	0.7925514	2920	24589	198.85244
1.24E-11	0.2843118	2920	24589	46.2770142
1.19E-11	0.3826903	2920	24589	46.3738533
4.62E-10	0.858076371	2971	24589	39.0851811
1.98E-74	0.199460386	2971	24589	352.591075
2.47E-09	0.858076371	3596	24589	35.7664619
9.97E-142	0.199460386	3596	24589	703.454164
4.42E-08	0.858076371	3596	24589	30.0633853
1.30E-124	0.207431801	3596	24589	610.296742
8.74E-17	0.1746698	3596	24589	69.9514206
4.97E-08	0.416342945	3596	24589	29.8588851
6.72E-09	0.91861	3596	24589	33.8031342
5.43E-09	0.858076371	3387	24589	34.2139963
2.29E-112	0.199460386	3387	24589	547.730682
1.57E-13	0.1683052	3629	24589	54.8963616
7.19E-31	0.9605163	2872	24589	136.597135
4.65E-08	0.01148411	2872	24589	30.0206731
3.57E-08	0.798991197	2872	24589	30.5332443
7.22E-09	0.000398566	2872	24589	33.6868313
9.39E-09	0.01096451	3653	24589	33.0989567
5.39E-10	0.5016227	2911	24589	38.8028304
3.10E-10	0.2644156	1578	24589	40.1255227
1.64E-26	0.394562	3437	24589	115.477632
3.41E-08	0.394562	3162	24589	30.6158714
1.37E-08	0.1912981	3162	24589	32.4111249
3.21E-20	0.394562	3437	24589	85.9337667
1.42E-11	0.394562	2920	24589	46.0203669
4.05E-16	0.394562	3405	24589	66.8658966
2.38E-16	0.394562	2920	24589	68.0304345
1.13E-27	0.5405471	2920	24589	121.35139
7.38E-52	0.4880605	2919	24589	238.893245
1.61E-55	0.1723529	2919	24589	257.032355
5.77E-33	0.4880605	2920	24589	146.674324
2.26E-26	0.1723529	2920	24589	115.097294
3.91E-10	0.6000575	3595	24589	39.3836043
5.86E-11	0.361577	3595	24589	43.1336712
4.68E-11	0.6852752	2872	24589	43.6443808
4.48E-23	0.3777564	2872	24589	99.5422599
2.85E-10	0.6852752	2871	24589	40.0562295
1.58E-20	0.3777564	2871	24589	87.5644471
5.49E-11	0.6852752	2872	24589	43.32639
1.44E-22	0.3777564	2872	24589	97.1411488

3.09E-10	0.6852752	2871	24589	39.9032703
4.45E-20	0.3777564	2871	24589	85.4352538
1.78E-13	0.3941554	3657	24589	54.6553887
4.64E-08	0.6687117	1570	24589	30.1700265
1.35E-31	0.7651576	3410	24589	139.560496
5.63E-28	0.7651576	3374	24589	122.441894
2.54E-08	0.624550461	3410	24589	31.1743634
4.02E-144	0.207431801	3410	24589	720.328596
3.87E-12	0.866123264	3410	24589	48.5506401
9.59E-11	0.2132418	3435	24589	42.1515725
4.31E-12	0.2132418	3434	24589	48.3285195
2.73E-08	0.160555237	3118	24589	31.0680128
1.03E-12	0.01201562	3427	24589	51.1611489
1.45E-11	0.01201562	3395	24589	45.9051866
1.45E-09	0.3248189	3408	24589	36.815687
5.64E-12	0.4160417	3408	24589	47.7982274
1.82E-11	0.08682414	3427	24589	45.4570923
1.68E-09	0.08682414	3405	24589	36.5118305
7.04E-13	0.3281697	3652	24589	51.9061386
7.59E-09	0.4160417	3652	24589	33.5229303
8.47E-09	0.2410625	3042	24589	33.3602191
3.13E-60	0.199460386	2871	24589	280.940853
8.46E-99	0.199460386	3410	24589	475.452707
1.03E-225	0.199460386	3410	24589	1201.60531
3.16E-16	0.866123264	3410	24589	67.3656271
3.09E-08	0.802019213	3410	24589	30.793337
1.63E-91	0.207431801	3410	24589	437.649373
2.25E-10	0.4556535	3410	24589	40.4557729
2.05E-136	0.207431801	3580	24589	674.569742
1.15E-09	0.866123264	3580	24589	37.2428069
1.07E-08	0.980374	3657	24589	32.901696
1.02E-58	0.5446947	1244	24589	290.590189
5.38E-09	0.9910141	1244	24589	34.5401085
3.22E-08	0.919311	1244	24589	30.9789791
2.63E-15	0.5446947	1858	24589	63.6089622
1.16E-08	0.153125183	1579	24589	32.887243
3.01E-09	0.1461362	3162	24589	35.3796922
2.87E-18	0.03542258	3112	24589	76.9202454
6.75E-37	0.3694153	3592	24589	164.770615
2.39E-08	0.6021094	3652	24589	31.2824438
2.76E-09	0.6021094	3668	24589	35.5208653
4.50E-08	0.8007875	2650	24589	30.0997073
2.00E-15	0.743863	1858	24589	64.1588636
2.72E-08	0.7736578	3653	24589	31.0319353
1.05E-08	0.8286304	3653	24589	32.8972435
1.96E-13	0.4901444	3425	24589	54.4501171
3.86E-08	0.902200939	3657	24589	30.3454218
5.30E-20	0.9083672	1559	24589	86.1963995
1.85E-21	0.9083672	1556	24589	93.2337444
5.54E-18	0.6102493	3652	24589	75.4555751
8.42E-10	0.04042893	3652	24589	37.8661516
1.97E-11	0.6102493	3658	24589	45.2911985
1.41E-25	0.5535857	3668	24589	110.975893
1.12E-08	0.8377527	3669	24589	32.7651533
1.25E-08	0.8377527	3669	24589	32.5545603
4.71E-08	0.2245189	3113	24589	29.9860493
3.34E-08	0.07764034	3629	24589	30.6382137
1.63E-09	0.3167371	2920	24589	36.5881347

4.65E-24	0.5791832	3652	24589	103.840815
2.18E-24	0.5791832	3668	24589	105.356207
1.17E-15	0.8319031	2971	24589	64.821968
6.36E-19	0.5379434	3657	24589	79.8385646
2.71E-23	0.8629067	1635	24589	101.934061
2.50E-08	0.01729327	3113	24589	31.2272783
1.59E-08	0.91861	1858	24589	32.2261016

R2
0.00651714
0.00490968
0.00574865
0.00721821
0.00401457
0.00439144
0.00466895
0.01185231
0.00505267
0.00242575
3.5508E-05
6.8257E-06
4.5786E-05
4.1267E-05
1.0996E-05
1.6124E-05
3.3318E-05
1.097E-05
7.365E-06
1.8779E-06
1.9978E-06
1.9839E-06
1.2264E-05
2.4993E-05
1.9224E-05
5.3608E-06
6.5788E-06
1.9542E-06
8.9019E-06
1.9309E-06
6.8926E-06
1.9636E-06
1.1048E-05
1.225E-05
4.3103E-06
2.0021E-06
9.0834E-06
3.801E-06
4.6592E-06
5.1386E-06
2.0045E-06
1.322E-05
0
3.835E-05
2.1497E-06
1.9574E-06
0.00278312
0.00038896
0
0.00220942
0.00374844
0.00265593
0.0018208
0.00510597
0.00409329
0.0002715

0.00011167
8.965E-05
0.00048754
0.00034423
4.4957E-05
5.4576E-05
3.8198E-05
5.7999E-05
0.00022292
5.7411E-05
6.1922E-05
4.0547E-05
4.0323E-05
4.035E-05
9.2156E-05
0.00010008
8.0574E-05
4.5099E-05
4.1906E-05
1.5271E-05
0.00010484
4.0004E-05
0.00014592
8.8343E-06
4.9568E-05
0.00010883
5.5833E-05
6.5616E-05
4.41E-05
5.2992E-05
1.2519E-05
4.5299E-05
4.1345E-05
1.4873E-05
5.0987E-05
7.9401E-05
3.2401E-05
7.5521E-05
5.7151E-05
5.1702E-05
5.1261E-05
3.1774E-05
3.7987E-05
0.00012889
8.6202E-05
9.6047E-05
6.93E-05
6.6528E-05
1.4136E-05
5.0796E-05
3.8957E-05
0.0001988
3.5025E-05
3.6988E-05
5.086E-05
5.7677E-05
0.0001031
4.6177E-05

0.0002241
5.2398E-05
5.6019E-05
4.3173E-05
0.00014253
5.7828E-05
0.00065795
4.1477E-05
0.00017592
6.4412E-05
0.00015799
6.6766E-05
0.00020699
4.6362E-05
6.6053E-05
6.9357E-05
3.6723E-05
9.7126E-05
7.4908E-05
2.3241E-05
1.5437E-05
0.00061153
3.0397E-05
4.6611E-05
6.0078E-05
5.8549E-05
0.0004072
5.9222E-05
4.9695E-05
5.2477E-05
1.9192E-05
4.5375E-05
0.00014839
2.1465E-06
7.014E-05
2.3055E-05
0.00020614
0.00094683
2.3071E-05
1.4576E-05
1.0095E-05
0.00044183
0.00011758
5.0749E-05
0.00040329
4.6081E-05
5.6931E-05
5.9009E-05
6.9227E-05
0.0002885
2.8467E-05
8.8681E-05
0.00013459
5.5997E-05
4.8922E-05
4.4886E-05
0.00021468
0.00017538

0.00022599
8.2345E-05
1.9264E-05
2.4712E-05
6.553E-05
4.2091E-05
5.0647E-05
5.1023E-05
0.00012997
4.4242E-05
4.2255E-05
7.3091E-05
4.8075E-05
0.00025913
3.1662E-05
1.5271E-05
0.00035377
4.7661E-05
6.2167E-05
0.00013602
5.3151E-05
7.0921E-05
3.0237E-05
6.43E-05
5.5537E-05
4.9301E-05
2.7105E-05
7.0473E-06
1.7974E-05
7.3159E-05
2.7225E-05
0.00019127
4.2349E-05
0.00022242
4.5016E-05
5.7268E-05
2.0955E-05
4.3349E-05
6.2481E-05
8.9433E-05
8.202E-05
4.414E-05
1.2501E-05
6.9782E-05
1.797E-05
0.00019929
0.00013782
1.0785E-05
5.2233E-05
3.3924E-05
5.3217E-05
4.7615E-05
5.9456E-05
2.2586E-05
5.7175E-05
3.0269E-05
3.0906E-05
1.8372E-05

1.2218E-05
3.0611E-05
0.00013386
0.0003836
7.3522E-05
8.8119E-05
1.4812E-05
0.00018495
0.00021725
3.8309E-05
6.6466E-05
4.0763E-05
3.2426E-05
5.5695E-05
6.3987E-05
2.268E-05
4.3571E-05
2.2754E-05
5.1022E-05
1.667E-05
5.4943E-05
0.000244
5.3657E-05
0.00018252
2.8531E-05
5.4868E-05
0.0001545
1.4452E-05
0.00013677
2.6181E-05
0.00011839
4.5079E-05
1.7459E-05
4.7581E-05
4.9974E-05
3.7637E-05
4.4804E-05
5.6194E-05
5.0969E-05
8.0009E-05
3.8912E-05
7.3943E-05
0.00017858
5.3901E-05
0.00020039
3.2293E-05
0.00032992
0.00022965
0.00030827
2.4454E-05
3.6457E-06
0
4.1265E-05
4.3601E-05
1.6647E-05
0.00011823
9.3869E-05
1.8632E-05

0.00010288
4.1344E-05
0.0001996
0.0002303
1.1094E-05
0.00072893
1.9292E-05
1.7367E-05
9.8048E-05
4.3122E-05
0.00033599
6.3949E-05
0.00255294
0.00283611
0.00185171
0.00319888
0.01265063
0.00148569
0.01005429
0.01515875
0.00475174
0.0007492
0.0135698
0.00393975
0.00860462
0.00393975
0.00860462
0.00531194
0.00011241
0.00386677
0.00556678
0.00966127
0.00522017
0.03026987
0.00636121
0.25114088
0.00161085
0.0051632
0.02937047
0.00654129
0.00188822
0.2939078
0.00190393
0.00702064
0.0400967
0.00614549
0.00256558
0.39847562
0.00290795
0.00495327
0.03734023
0.00689468
0.4162161
0.00333145
0.00254463
0.00559845
0.00091388
0.00234006

0.00166184
0.05988658
0.00606921
0.00662626
0.00219845
0.00213563
0.0046825
0.01160799
0.00998349
0.00121638
0.00574425
0.00255149
0.00238434
0.00474248
0.00337041
0.00890372
0.00972717
0.00117114
0.00617111
0.00248103
0.0952039
0.00235178
0.00489705
0.00322376
0.00851693
0.01001585
0.0011544
0.00525267
0.00246328
0.00191913
0.00321972
0.00386218
0.00940643
0.00931902
0.08451248
0.00113697
0.00481198
0.00203028
0.00186553
0.00283069
0.00830743
0.00827786
0.07539288
0.00102583
0.00549209
0.00255695
0.00225731
0.00514975
0.00345021
0.00893307
0.00966438
0.00118936
0.00537932
0.00265016
0.09827614
0.00240532
0.00539783
0.00347127

0.00899737
0.0094052
0.00123723
0.00546983
0.00256917
0.00227263
0.00574746
0.00334416
0.00871225
0.00953873
0.00122373
0.00639355
0.0024681
0.00238993
0.00321839
0.00835557
0.01032292
0.00111954
0.0045024
0.03268883
0.00497521
0.0060875
0.03801312
0.00056433
0.00652815
0.01140978
0.00465934
0.31614291
0.00744731
0.03778698
0.00291022
0.41029253
0.00508331
0.00346629
0.00920385
0.05005144
0.00056894
0.0040565
0.0091403
0.00118993
0.58486475
0.00437382
0.00647879
0.04045801
0.3668212
0.01303101
0.00073225
0.00277716
0.02084673
0.00873842
0.01128504
0.08982432
0.00075395
0.03663882
0.39302968
0.01637283
0.01240622
0.00287089

0.00573569
0.00055652
0.00185645
0.07862635
0.0194138
0.03379267
0.01126861
0.01469805
0.40864994
0.00857404
0.00720776
0.05137176
0.01958195
0.01518253
0.000494
0.85008146
0.00193171
0.04106539
0.00320411
0.00704718
0.4850089
0.00283004
0.00164656
0.04806246
0.00087661
0.03149002
0.02984902
0.00286668
0.00776547
0.00013492
0.00026684
0.00561809
0.00323138
0.00933719
0.00014006
0.00028776
0.01288991
0.01395461
0.01378957
0.00115476
0.01707396
0.00161452
0.00032988
0.0007261
0.04903734
0.00036083
0.01023015
0.00256446
0.0043425
0.00755056
0.04484853
0.00956533
0.00372701
0.00068639
0.00466548
0.00028255
0.02686708
0.00505465

0.01064666
0.01562536
0.00376719
0.02145977
0.00788913
0.00353653
0.00494495
0.00425708
0.00388229
0.002816
0.00458455
0.00844342
0.07430013
0.00478614
0.00607063
0.00102801
0.00171161
0.00409411
0.00228711
0.00272818
0.05276634
0.00308817
0.00458949
0.00044684
0.00051378
0.00076079
0.00445614
0.00116451
0.00374074
0.00572208
0.01486466
0.0732659
0.00686792
0.00502825
0.00091893
0.00228583
0.00110684
0.00256602
0.00423193
0.00465971
0.00573374
0.04668191
0.00046815
0.00103239
0.00450085
0.0029763
0.00586826
0.00721204
0.09304814
0.00316453
0.00453978
0.00071943
0.00018883
0.01554186
0.00016051
9.8743E-05
0.00040201
0.00427619

9.6555E-05
0.00652457
0.00689909
0.00012453
0.00836115
0.0011972
0.00324481
0.00772779
0.00806588
0.00631033
0.02386196
0.04024562
0.00409533
0.00229927
0.00050499
1.8352E-06
7.5509E-06
1.4302E-05
2.6392E-06
2.7564E-05
1.3406E-05
9.8933E-05
0.00413708
0.00014862
5.7851E-06
1.1547E-05
0.00011109
6.4416E-06
7.8749E-06
2.1333E-06
2.1322E-05
0.01032005
2.3803E-06
2.368E-06
2.1111E-06
7.0504E-06
6.8318E-05
5.6739E-06
1.1151E-05
4.6618E-05
9.9836E-05
0.00014634
1.2938E-05
2.1851E-06
4.6693E-05
5.9448E-06
5.8949E-06
2.9559E-05
1.5423E-05
4.811E-05
0.00013218
5.2396E-05
4.1446E-05
4.1278E-05
7.16E-06
1.0617E-05
7.3596E-06
8.5228E-05

5.4642E-06
1.7872E-05
0
2.659E-05
0.00411111
0
2.1433E-05
8.9001E-05
3.7982E-06
1.3573E-05
0.00035902
6.159E-05
1.0244E-05
1.9029E-05
1.2192E-05
2.1837E-05
3.0552E-06
5.2802E-05
1.7993E-05
1.4992E-05
0.00016494
5.9793E-06
1.0275E-05
2.6903E-06
3.3534E-06
1.0519E-05
5.5074E-06
4.5644E-05
1.6745E-05
7.2372E-06
3.6399E-06
0.00682126
2.1666E-06
1.0716E-05
1.4303E-05
2.1341E-06
2.082E-05
1.924E-06
0.00019009
1.6494E-05
6.1087E-05
7.7956E-06
2.0669E-06
7.5501E-05
0.00013222
1.5049E-05
5.8041E-05
8.8028E-05
8.0554E-05
2.9903E-05
1.1097E-05
1.3592E-05
0.00020194
9.4931E-06
3.8088E-05
0.00030117
5.3201E-06
7.1715E-06

6.9258E-06
7.3068E-06
0
0.00064609
0.00165726
0.00074187
0.0523835
0.001068
0.00281953
0.01595736
0.0309824
0.00892644
0.00672533
0.00513076
0.0055883
0.0058961
0.00265854
0.0044678
0.17250113
0.01836057
0.00411103
0.00515936
0.00423319
0.16160631
0.01538933
0.00467118
0.00298351
0.07692123
0.00090725
0.00361509
0.10116466
0.00202007
0.00381059
0.00336145
0.00074156
0.00268648
0.00212268
0.00983649
0.00202268
0.00177008
0.00269294
0.00193585
0.01266402
0.00196573
0.0018472
0.00339121
0.00268838
0.00285616
0.00920242
0.15082688
0.00215669
0.00033415
0.0005699
0.00320462
0.00459033
0.00034347
0.00162138
0.00338994

0.00307902
0.00676588
0.00068555
0.00069034
0.00485808
0.00236255
0.01957786
0.00984714
0.01215522
0.00108423
0.02037422
0.0009827
1.8932E-06
1.8174E-06
1.8447E-06
0
1.8647E-06
0.00038127
8.6577E-06
0
1.7845E-06
1.9343E-06
1.8628E-06
1.7892E-06
1.9045E-06
1.8652E-06
1.8656E-06
1.87E-06
1.8569E-06
1.9354E-06
0.00166595
0.00178171
0.00230308
0.0066471
0.00039294
0.00562789
0.00042555
0.00036866
0.00034242
0.00290304
0.00104351
0.00546257
0.00552096
0.00831031
0.0001833
0.00396819
9.6296E-05
1.1978E-05
0.00164849
0.00228235
0.00252111
0.03440793
5.4598E-06
0.00012871
0.00547011
0.00356071
5.4828E-06
8.5373E-05

0.00010423
0.00452033
0.00255747
0.00241182
0.00092268
0.00232579
7.2251E-05
6.0042E-05
6.3021E-05
0.00088816
0.00077226
0.00393844
0.0054966
0.00087967
0.00076841
0.00093055
0.00039887
0.00037847
0.00040799
0.00034422
0.00032019
0.00043828
0.00033635
0.00037593
0.00095128
0.00030681
0.00036682
0.00053546
0.00049103
0.00056468
0.00036003
0.00435629
0.00043544
0.00099417
0.01603028
6.6971E-06
0.00378822
1.6014E-05
0.00433897
0.00446436
0.01649053
4.6156E-05
5.7233E-05
0.00388615
5.7057E-05
0.02856594
0.00380803
0.00048524
0.10010715
0.0010808
0.00048192
0.10840796
0.00086611
0.00044202
0.10775287
0.00079027
0.00040621
0.10270683

0.00088775
0.00398605
0.00020799
0.00078916
0.00026909
0.0010034
0.00062081
0.00036886
0.0010276
0.00316911
0.00059339
0.0014656
0.00036067
0.00117368
0.00079505
0.0003404
0.0006329
0.00147867
0.00088151
0.02370116
0.00130761
0.01530676
0.00316736
0.016031
0.0048139
0.00066774
0.02288745
0.00132355
0.00606645
0.00403108
0.00142193
0.00127416
0.00344419
0.00162066
0.00455324
0.00394769
0.00149417
0.00136571
0.00354369
0.00167877
0.00253968
0.00445531
0.00122489
0.00116341
0.00485427
0.00143792
0.0043773
0.00097834
0.00089871
0.00628535
0.00479277
0.00079139
0.00117454
0.00115427
0.00353579
0.00147713
0.00482973
0.0008824

0.00077921
0.00226175
0.00581584
0.00478699
0.00071922
0.00450881
0.00097898
0.00102777
0.00495821
0.0012844
0.00482294
0.00129746
0.00125645
0.0049664
0.00149863
0.01492519
0.02791221
0.00488603
0.00164523
0.00065302
0.0909888
0.00180604
0.00799562
0.00565449
0.00045774
9.4666E-05
0.00204089
0.001337
0.0075434
0.00040192
0.00111915
0.27441397
0.00327513
0.00019353
0.00526514
8.9022E-05
1.1994E-05
0.00227195
0.00493935
8.1116E-05
0.0032373
0.00010711
0.00868808
0.00031455
0.0001536
0.00177238
0.02481108
0.00055121
0.00022344
0.00169204
0.0039803
0.00664596
0.01072277
0.00676018
0.0009078
0.00020272
0.00104118
0.23404904

0.00279889
0.00014217
0.00547418
0.00433188
0.00138497
0.00010232
0.00422499
0.01029068
0.00030565
0.00015491
0.00179838
0.0001707
0.02463602
0.00055907
9.9226E-05
7.4951E-05
0.00108119
0.00012603
0.00424534
0.00383002
0.00026414
0.00174381
0.0211936
0.00011392
6.9444E-05
0.00011122
0.00019545
0.00372359
0.00593499
0.00097674
0.00045115
0.00018375
0.00091588
0.00510719
0.00283569
0.00251595
0.00019475
9.2544E-05
0.0066968
0.02054889
0.00011713
9.1461E-05
0.00025549
0.00181644
0.02132253
0.00010939
9.5723E-05
8.172E-05
0.00100734
0.000164
8.3816E-05
0.00254579
0.0008612
0.02451198
0.00153963
0.00161558
0.00453188
0.0070028

0.02447495
0.0028929
0.0070333
0.02389918
0.0030019
0.00562254
0.02201294
0.00316438
0.00515411
0.02086927
9.7392E-06
0.00439205
0.00522541
0.00642235
0.00551217
0.00941722
0.00800202
0.00535833
0.0071898
0.00520296
0.01381527
0.00757353
0.01185325
0.0136453
0.00459684
0.00797724
0.00653419
0.00042673
0.00082493
0.03333314
0.00018118
0.0004026
0.00075287
0.02755222
0.00018307
0.0137719
0.00016968
0.00478094
0.00040595
0.00434569
0.00035307
0.00466718
0.00267395
0.01627211
9.1384E-05
0.00883571
0.00406217
0.00863218
0.00474145
0.00436655
0.00546406
0.00476584
0.00945426
0.00528727
0.00640554
0.00434928
0.00785642
0.00106174

0.00268318
0.00446816
0.0008168
0.00342661
0.00513616
0.00100759
0.00097071
0.00084004
0.09449379
0.00552749
0.00598616
0.00819416
0.00103319
0.00093608
0.00544838
0.00583861
0.00784863
0.00096349
0.00077962
0.00661033
0.00966615
0.0065662
0.00100436
0.00103093
0.00531388
0.0056289
0.00785295
0.00095784
0.00093504
0.00078059
0.00586229
0.00638324
0.0038572
0.00887542
0.08920014
0.00101222
0.00083005
0.00593096
0.00505333
0.00780756
0.00561388
0.00056785
0.00379002
0.00235447
0.00030796
0.03504421
0.00285142
0.027109
0.00011189
7.8156E-05
0.00247369
0.00712166
0.00145636
0.00185652
0.0166909
0.01308135
0.00151803
0.00653605

0.00241911
0.00132688
0.00064444
0.08872984
0.00061227
0.00093675
0.00149444
0.00480972
0.00524699
0.40273782
0.00323243
0.00158512
0.00460922
0.00551976
0.00942663
0.38412324
0.00300069
0.00163728
0.00491737
0.00848005
0.00545037
0.00348938
0.38453711
0.00512984
0.04000641
0.00011717
5.4427E-05
5.3971E-06
0.00013725
5.2658E-05
0.00150347
0.0052708
1.8848E-05
0.00454174
0.00682356
1.7965E-05
0.0037949
0.00073854
0.00237843
0.00863929
0.00228403
0.0019085
0.00305469
0.02372831
0.01041945
0.00241374
0.00188248
0.0055662
0.01015469
0.00243362
0.00045425
0.00439901
0.00536688
0.00229459
6.3744E-05
0.00327817
0.00172
0.00199429

0.00264329
0.00358823
0.00687266
0.00453331
0.00400197
0.00356931
0.00852768
0.0037851
0.00296424
0.00338322
0.01774103
0.00438488
0.01151607
0.13140226
0.01392447
0.01678504
0.00494259
0.00273705
0.00182539
0.00431099
0.00564589
0.03639996
0.01724281
0.02891579
0.00632749
0.00193917
0.01630851
0.00509107
0.00228642
0.00562304
0.00528568
0.00863704
0.00418182
0.03905446
0.00291094
0.00723454
0.00121046
0.00292936
0.00928989
0.00072253
0.00401538
0.00463451
0.00396458
0.00512467
0.00398349
0.00159921
0.0012658
0.04493035
0.00558386
0.00684204
0.00497628
0.00581894
0.00426179
0.0082054
0.00533977
0.00488021
0.00507725
0.00881729

0.00518605
0.0040008
0.00408758
0.11376472
0.00260595
0.00314501
0.0052501
0.00362676
0.00614786
0.0071198
0.00265678
0.01022521
0.00172147
0.0010125
0.00077346
0.00538909
0.00120317
0.00381714
0.00728792
0.08591157
0.00087968
0.00073875
0.0056335
0.00119829
0.00367427
0.00783484
0.08331435
0.00292931
0.00610782
0.00411339
0.00051621
0.00467882
0.01112013
0.0223808
0.00300367
0.00486452
0.0051141
0.00318195
7.3324E-05
7.8636E-05
0.00047328
0.00118367
0.00329787
0.00566062
0.00368436
0.00063587
0.01768566
0.00110583
0.0005666
0.00387291
0.00494777
0.00059175
0.00062682
0.0037502
0.00086809
0.0010365
0.00070066
0.00066159

0.00067518
0.00078255
0.00423443
0.00585266
0.00475203
0.00078637
0.00088947
0.00427692
0.00104474
0.00113965
0.00062259
0.00138813
0.00167433
0.00581257
0.00104555
0.00954489
0.09250997
0.00968465
0.00531054
0.01521439
0.00987974
0.00264335
0.01130026
0.00685511
0.01101452
0.00033416
0.01443861
0.00504503
0.0003741
0.00252636
0.00876674
0.00525597
0.01445931
0.00061212
0.0016781
0.00442697
0.0001972
0.00111468
0.00119848
0.00421198
0.00591161
0.00076309
0.00021835
0.00080106
0.09210019
0.00224194
0.00599576
0.04361143
0.00603739
0.04365066
0.00191779
0.00292147
0.00367123
0.00702405
0.00817484
0.00090819
0.00187638
0.0010347

0.00298597
0.00754579
0.001004
0.00011423
0.00957928
0.00083004
0.00729163
0.00113662
0.00148939
0.00415096
0.01027195
0.00105153
0.00153934
0.00397947
0.01134737
0.00065383
0.11117973
0.00213692
0.00093817
0.00158932
0.00928975
0.00057167
0.00224227
0.00090297
0.00020866
0.09892516
0.0024855
0.00089861
0.00444852
0.00829887
0.00190483
0.00550749
0.00223729
0.00202524
0.00844787
0.00167839
0.00431918
0.00182116
0.00293764
0.0139166
0.00268183
0.00183033
0.00036301
0.00810719
0.0008358
0.00477998
0.00963881
0.00664045
0.00921377
0.00069775
0.00945118
0.00463246
0.02624588
0.04662624
0.0457279
0.00226682
0.00648718
0.03306845

0.00141625
0.00505351
0.02979819
0.01726576
0.04154273
0.00685024
0.00883862
0.00010389
0.00409628
0.01257757
7.7178E-05
0.00616782
7.1176E-05
0.00425956
9.5058E-05
0.00370032
0.000149
0.00408664
0.00467009
0.00521959
0.00479399
0.0023431
0.00253371
0.00235877
0.00288687
0.00026722
0.00253117
0.02668004
0.01829809
5.2466E-05
0.0029907
0.00185093
0.00126249
0.00667521
0.00174103
0.00637249
0.00163385
0.00125274
0.00761172
0.00042456
0.01353673
0.01169839
0.01799773
0.02142726
0.0209882
0.03978673
0.00334295
0.03037875
0.00771567
0.01348378
0.01239029
0.22260668
0.00111431
0.02227118
0.02114125
0.03998705
0.00074403
0.00536606

0.03030072
0.01198482
0.23555441
0.00978577
0.0038459
0.02294223
0.02023353
0.01037019
0.04995764
0.04090498
0.00369256
0.06156825
0.0018412
0.01427425
0.01057734
0.01063703
0.0798251
0.03660428
0.01078649
0.06150592
0.00186314
0.01488759
0.0036546
0.00065555
0.00285343
0.00938853
0.00104908
0.00938729
0.0005645
0.01535457
0.01035851
0.00326333
0.00388549
0.00635539
0.05009986
0.00248896
0.00566853
0.00096778
0.00204745
0.00051792
0.00669723
0.00414922
0.00290749
0.00149623
0.02527516
0.02338049
0.02262976
0.02044777
0.02058975
0.02164667
0.00019107
0.08264013
0.00187931
0.0001875
0.09209582
0.00187265
0.00019638
0.12782534

0.00252512
0.00817574
0.00310076
0.00413511
0.0040436
0.03156404
0.00597009
0.02826014
0.00512125
0.00401886
0.13416674
0.0027058
0.0036342
0.00331451
0.00592901
0.00394414
0.00885088
0.00443778
0.00895229
0.00478734
0.00369063
0.0067662
0.00442541
0.0042016
0.00444634
0.00705771
0.00417547
0.02380826
0.00316944
0.00545772
0.02537842
0.00293165
0.02092382
0.01723695
0.0140925
0.01354929
0.01545126
0.01646243
0.00935761
0.00854192
0.00684799
0.00327773
0.00854217
0.00400505
0.0024187
0.00645048
0.00620469
0.00686119
0.00614097
0.00554073
0.00615136
0.00602732
0.00551921
0.00601582
0.00752534
0.00665819
0.00367189
0.00615202

0.01836543
0.00490479
0.00125019
0.02894193
0.00418496
0.01575796
0.00577966
0.00818424
0.00500222
0.00143947
0.03954306
0.00503432
0.00397641
0.0397725
0.00477411
0.00537164
0.003855
0.00545793
0.00496097
0.0054808
0.00940641
0.00574851
0.01028279
0.0764772
0.0009389
0.00157114
0.00449393
0.00504927
0.00432525
0.00124762
0.00933058
0.02587893
0.00621789
0.00948022
0.0118319
0.01242355
0.00874213
0.00585983
0.00498836
0.00635509
0.00935753
0.00590378
0.00491808
0.02955384
0.00342662
0.08197223
0.00208915
0.00297453
0.00262004
0.07726863
0.00245251
0.0004299
0.11245868
0.00559742
0.06901774
0.00150608
0.00235443
0.01004863

0.05723134
0.00170009
0.01334792
0.0572579
0.09697723
0.00177447
0.00777621
0.02887421
0.00417232
0.00258596
0.03201924
0.00042053
0.00525718
0.02799158
0.00272762
0.01485026
0.00438041
0.00875436
5.339E-05
0.02215414
0.00432384
0.00505567
0.00544684
0.00457229
0.0053238
0.00215994
0.00519307
0.00643486
0.00423899
0.01584274
0.00299121
0.00535489
0.01662008
0.00300538
0.00629706
0.00435175
0.00434737
0.00092305
0.01861169
0.00261641
0.00763126
0.00211232
0.02112409
0.0057293
0.01445242
0.00339858
0.01912548
0.00341869
0.0058386
0.00372399
0.00400424
0.0033263
0.0113833
0.00926419
0.05661971
0.00145944
0.06820663
0.00124549

0.002941
0.00861153
0.00051977
0.00051484
0.22584716
0.01607123
0.01964382
0.1720034
0.01564154
0.01853279
0.033823
0.00424825
0.0118635
0.23418705
0.00943266
0.00297218
0.02409759
0.01873965
0.0126544
0.00594374
0.21826073
0.0145124
0.01882579
0.22354076
0.01563688
0.0197286
0.01669442
0.15838413
0.00278999
0.01842992
0.00412905
0.01642131
0.006908
0.0075119
0.00032976
0.0058469
0.00611467
0.00188383
0.00909612
0.0165162
0.0103096
0.01073053
0.01912101
0.00756775
0.00665367
0.00582127
0.00406575
0.0196013
0.00375684
0.01682712
0.0012388
0.00208372
0.00070461
0.00034026
0.00403258
0.00116545
0.01035034
0.00067076

0.00829703
0.00063112
0.00380171
0.00223628
0.00291182
0.00291182
0.00249784
0.00409727
0.00175746
0.00265029
0.00428874
0.0326025
0.00926052
0.00405174
0.04573504
0.00112231
0.02159514
0.00632948
0.00565428
0.00645069
0.04331825
0.00488406
0.07284672
0.00410528
0.06319974
0.00659277
0.00415168
0.00460851
0.00496516
0.05981018
0.00358797
0.02165481
0.00056897
0.00076942
0.00177568
0.0045065
0.00289986
0.01104426
0.00345066
0.00099096
0.00019491
0.00256784
0.00170823
0.00201334
0.00252522
0.01321376
0.0260611
0.02807174
0.01598931
0.01256602
0.00443534
0.00599908
0.00558926
0.01438815
0.00512723
0.01266376
0.00554854
0.01404109

0.00510765
0.01235584
0.00286784
0.00344976
0.01847143
0.01636728
0.00234655
0.07808358
0.00182432
0.00551748
0.00632876
0.00304713
0.00628322
0.00568871
0.00344519
0.00616046
0.00660896
0.00534207
0.00441867
0.00403576
0.00537052
0.03624124
0.05153905
0.13025395
0.00253131
0.00177377
0.04744117
0.00461599
0.07014874
0.00132938
0.00432494
0.07052835
0.0105331
0.01221355
0.01188666
0.00327776
0.00022812
0.00786195
0.00816131
0.00387451
0.00438147
0.00089945
0.00055487
0.00040767
0.00103008
0.00724113
0.00032687
0.01587844
0.01723918
0.00078611
0.0011641
0.00047108
0.00586723
0.00390368
0.00387859
0.00166723
0.00359039
0.00449459

0.00555406
0.00560156
0.00425839
0.00645031
0.02573112
0.00393019
0.00848384

Supplementary Table 3

Id.exposure	Outcome	Exposure
GCST90001392	IPF	IgD+ CD38+ B cell Absolute Count
GCST90001424	IPF	IgD+ B cell %Lymphocyte
GCST90001426	IPF	IgD- CD38dim B cell %lymphocyte
GCST90001436	IPF	Memory B cell %lymphocyte
GCST90001458	IPF	Myeloid Dendritic Cell Absolute Count
GCST90001465	IPF	CD86+ myeloid Dendritic Cell %Dendritic Cell
GCST90001465	IPF	CD86+ myeloid Dendritic Cell %Dendritic Cell
GCST90001465	IPF	CD86+ myeloid Dendritic Cell %Dendritic Cell
GCST90001465	IPF	CD86+ myeloid Dendritic Cell %Dendritic Cell
GCST90001465	IPF	CD86+ myeloid Dendritic Cell %Dendritic Cell
GCST90001473	IPF	CD62L- CD86+ myeloid Dendritic Cell %Dendritic Cell
GCST90001473	IPF	CD62L- CD86+ myeloid Dendritic Cell %Dendritic Cell
GCST90001473	IPF	CD62L- CD86+ myeloid Dendritic Cell %Dendritic Cell
GCST90001473	IPF	CD62L- CD86+ myeloid Dendritic Cell %Dendritic Cell
GCST90001473	IPF	CD62L- CD86+ myeloid Dendritic Cell %Dendritic Cell
GCST90001474	IPF	Plasmacytoid Dendritic Cell %Dendritic Cell
GCST90001475	IPF	HLA DR++ monocyte %monocyte
GCST90001520	IPF	CD33+ HLA DR+ CD14dim Absolute Count
GCST90001527	IPF	CD33dim HLA DR+ CD11b- Absolute Count
GCST90001547	IPF	Terminally Differentiated CD4+ T cell %T cell
GCST90001554	IPF	Effector Memory CD8+ T cell Absolute Count
GCST90001558	IPF	Terminally Differentiated CD8+ T cell %CD8+ T cell
GCST90001558	IPF	Terminally Differentiated CD8+ T cell %CD8+ T cell
GCST90001558	IPF	Terminally Differentiated CD8+ T cell %CD8+ T cell
GCST90001558	IPF	Terminally Differentiated CD8+ T cell %CD8+ T cell
GCST90001558	IPF	Terminally Differentiated CD8+ T cell %CD8+ T cell
GCST90001580	IPF	CD14+ CD16+ monocyte Absolute Count
GCST90001585	IPF	CD14+ CD16+ monocyte %monocyte
GCST90001586	IPF	CD14+ CD16- monocyte %monocyte
GCST90001587	IPF	CD16+ monocyte %monocyte
GCST90001588	IPF	T/B cell
GCST90001596	IPF	CD8dim T cell Absolute Count
GCST90001596	IPF	CD8dim T cell Absolute Count
GCST90001596	IPF	CD8dim T cell Absolute Count
GCST90001596	IPF	CD8dim T cell Absolute Count
GCST90001596	IPF	CD8dim T cell Absolute Count
GCST90001597	IPF	CD8dim T cell %T cell
GCST90001599	IPF	CD4-CD8- T cell %T cell
GCST90001612	IPF	CD8dim T cell %leukocyte
GCST90001613	IPF	CD4-CD8- T cell %leukocyte
GCST90001613	IPF	CD4-CD8- T cell %leukocyte
GCST90001613	IPF	CD4-CD8- T cell %leukocyte
GCST90001613	IPF	CD4-CD8- T cell %leukocyte
GCST90001613	IPF	CD4-CD8- T cell %leukocyte
GCST90001614	IPF	CD8+ and CD8dim T cell %leukocyte
GCST90001648	IPF	HLA DR+ Natural Killer Absolute Count
GCST90001649	IPF	HLA DR+ Natural Killer %Natural Killer
GCST90001649	IPF	HLA DR+ Natural Killer %Natural Killer
GCST90001649	IPF	HLA DR+ Natural Killer %Natural Killer
GCST90001649	IPF	HLA DR+ Natural Killer %Natural Killer
GCST90001649	IPF	HLA DR+ Natural Killer %Natural Killer
GCST90001650	IPF	HLA DR+ Natural Killer %CD3- lymphocyte
GCST90001690	IPF	CD28+ CD45RA+ CD8+ T cell Absolute Count
GCST90001748	IPF	CD20 on IgD+ CD38- B cell
GCST90001748	IPF	CD20 on IgD+ CD38- B cell
GCST90001748	IPF	CD20 on IgD+ CD38- B cell

GCST90001748	IPF	CD20 on IgD+ CD38- B cell
GCST90001748	IPF	CD20 on IgD+ CD38- B cell
GCST90001781	IPF	CD25 on IgD+ CD38- naive B cell
GCST90001783	IPF	CD25 on IgD+ CD38+ B cell
GCST90001827	IPF	IgD on IgD+ B cell
GCST90001833	IPF	CD62L on CD62L+ Dendritic Cell
GCST90001867	IPF	CD3 on CD4+ T cell
GCST90001870	IPF	CD34 on Hematopoietic Stem Cell
GCST90001870	IPF	CD34 on Hematopoietic Stem Cell
GCST90001870	IPF	CD34 on Hematopoietic Stem Cell
GCST90001870	IPF	CD34 on Hematopoietic Stem Cell
GCST90001870	IPF	CD34 on Hematopoietic Stem Cell
GCST90001911	IPF	CD45 on Natural Killer
GCST90001944	IPF	CD123 on plasmacytoid Dendritic Cell
GCST90001945	IPF	CD123 on CD62L+ plasmacytoid Dendritic Cell
GCST90001947	IPF	CD33 on CD33+ HLA DR+ CD14dim
GCST90001947	IPF	CD33 on CD33+ HLA DR+ CD14dim
GCST90001947	IPF	CD33 on CD33+ HLA DR+ CD14dim
GCST90001947	IPF	CD33 on CD33+ HLA DR+ CD14dim
GCST90001947	IPF	CD33 on CD33+ HLA DR+ CD14dim
GCST90001950	IPF	CD33 on Granulocytic Myeloid-Derived Suppressor Cells
GCST90001950	IPF	CD33 on Granulocytic Myeloid-Derived Suppressor Cells
GCST90001950	IPF	CD33 on Granulocytic Myeloid-Derived Suppressor Cells
GCST90001950	IPF	CD33 on Granulocytic Myeloid-Derived Suppressor Cells
GCST90001950	IPF	CD33 on Granulocytic Myeloid-Derived Suppressor Cells
GCST90001952	IPF	CD33 on Monocytic Myeloid-Derived Suppressor Cells
GCST90001952	IPF	CD33 on Monocytic Myeloid-Derived Suppressor Cells
GCST90001952	IPF	CD33 on Monocytic Myeloid-Derived Suppressor Cells
GCST90001952	IPF	CD33 on Monocytic Myeloid-Derived Suppressor Cells
GCST90001952	IPF	CD33 on Monocytic Myeloid-Derived Suppressor Cells
GCST90001956	IPF	CD33 on CD33+ HLA DR+
GCST90001956	IPF	CD33 on CD33+ HLA DR+
GCST90001956	IPF	CD33 on CD33+ HLA DR+
GCST90001956	IPF	CD33 on CD33+ HLA DR+
GCST90001956	IPF	CD33 on CD33+ HLA DR+
GCST90001957	IPF	CD33 on CD33+ HLA DR+ CD14-
GCST90001957	IPF	CD33 on CD33+ HLA DR+ CD14-
GCST90001957	IPF	CD33 on CD33+ HLA DR+ CD14-
GCST90001957	IPF	CD33 on CD33+ HLA DR+ CD14-
GCST90001957	IPF	CD33 on CD33+ HLA DR+ CD14-
GCST90001959	IPF	CD4 on HLA DR+ CD4+ T cell
GCST90001965	IPF	FSC-A on monocyte
GCST90001970	IPF	FSC-A on HLA DR+ Natural Killer
GCST90002005	IPF	CD16 on CD14+ CD16+ monocyte
GCST90002005	IPF	CD16 on CD14+ CD16+ monocyte
GCST90002005	IPF	CD16 on CD14+ CD16+ monocyte
GCST90002005	IPF	CD16 on CD14+ CD16+ monocyte
GCST90002005	IPF	CD16 on CD14+ CD16+ monocyte
GCST90002014	IPF	CCR2 on CD62L+ myeloid Dendritic Cell
GCST90002017	IPF	CCR2 on monocyte
GCST90002037	IPF	CD80 on plasmacytoid Dendritic Cell
GCST90002038	IPF	CD80 on CD62L+ plasmacytoid Dendritic Cell
GCST90002077	IPF	SSC-A on HLA DR+ Natural Killer

Method	nsnp	β coefficient (95%CI)	SE	OR (95%CI)
IVW-mre	2	-0.142 (-0.158 - -0.125)	0.008245665	0.868 (0.854 - 0.882)
IVW-mre	2	-0.169 (-0.284 - -0.055)	0.058465806	0.844 (0.753 - 0.947)
IVW-mre	2	-0.243 (-0.401 - -0.085)	0.080706827	0.784 (0.669 - 0.919)
IVW-mre	2	-0.105 (-0.167 - -0.042)	0.031991103	0.901 (0.846 - 0.959)
IVW-mre	2	0.08 (0.044 - 0.116)	0.018374394	1.084 (1.045 - 1.123)
MR Egger	3	0.082 (-0.264 - 0.428)	0.176698386	1.086 (0.768 - 1.535)
IVW-mre	3	0.07 (0.061 - 0.078)	0.00452157	1.072 (1.063 - 1.082)
Weighted median	3	0.071 (-0.035 - 0.176)	0.05373131	1.073 (0.966 - 1.192)
Weighted mode	3	0.071 (-0.032 - 0.173)	0.052295288	1.074 (0.969 - 1.189)
Simple mode	3	0.073 (-0.109 - 0.255)	0.092933586	1.076 (0.897 - 1.291)
MR Egger	3	0.068 (-0.1 - 0.237)	0.085999731	1.071 (0.905 - 1.267)
IVW-mre	3	0.059 (0.049 - 0.069)	0.004928393	1.061 (1.051 - 1.071)
Weighted median	3	0.059 (-0.033 - 0.152)	0.047289049	1.061 (0.967 - 1.164)
Weighted mode	3	0.061 (-0.034 - 0.157)	0.048708357	1.063 (0.966 - 1.17)
Simple mode	3	0.041 (-0.11 - 0.192)	0.077075634	1.042 (0.896 - 1.212)
IVW-mre	2	-0.1 (-0.117 - -0.082)	0.008875584	0.905 (0.89 - 0.921)
IVW-mre	2	0.134 (0.11 - 0.159)	0.012345365	1.144 (1.116 - 1.172)
IVW-mre	2	0.067 (0.053 - 0.081)	0.007174369	1.07 (1.055 - 1.085)
IVW-mre	2	-0.146 (-0.232 - -0.059)	0.044035671	0.865 (0.793 - 0.943)
IVW-mre	2	-0.34 (-0.571 - -0.109)	0.117773391	0.712 (0.565 - 0.896)
IVW-mre	2	-0.026 (-0.027 - -0.024)	0.00093357	0.975 (0.973 - 0.976)
MR Egger	3	0.443 (-0.632 - 1.518)	0.548322852	1.558 (0.532 - 4.563)
IVW-mre	3	0.27 (0.134 - 0.405)	0.069016942	1.309 (1.144 - 1.499)
Weighted median	3	0.243 (0.008 - 0.478)	0.119967281	1.275 (1.008 - 1.613)
Weighted mode	3	0.366 (0.086 - 0.645)	0.142485194	1.442 (1.09 - 1.906)
Simple mode	3	0.174 (-0.138 - 0.486)	0.159099081	1.19 (0.871 - 1.626)
IVW-mre	2	-0.087 (-0.106 - -0.069)	0.009413313	0.917 (0.9 - 0.934)
IVW-mre	2	-0.08 (-0.092 - -0.069)	0.005936851	0.923 (0.912 - 0.934)
IVW-mre	2	0.061 (0.042 - 0.079)	0.009509285	1.063 (1.043 - 1.083)
IVW-mre	2	-0.062 (-0.08 - -0.043)	0.009286585	0.94 (0.923 - 0.958)
IVW-mre	2	0.101 (0.053 - 0.149)	0.024577725	1.106 (1.054 - 1.161)
IVW-mre	3	-0.187 (-0.315 - -0.058)	0.065591053	0.83 (0.73 - 0.944)
MR Egger	3	-0.039 (-0.496 - 0.417)	0.232839059	0.962 (0.609 - 1.518)
Weighted median	3	-0.155 (-0.429 - 0.118)	0.139446422	0.856 (0.651 - 1.125)
Weighted mode	3	-0.132 (-0.455 - 0.192)	0.164940846	0.877 (0.635 - 1.211)
Simple mode	3	-0.122 (-0.461 - 0.218)	0.173306898	0.886 (0.631 - 1.244)
IVW-mre	2	-0.144 (-0.211 - -0.077)	0.034023742	0.866 (0.81 - 0.926)
IVW-mre	2	-0.145 (-0.193 - -0.097)	0.024316394	0.865 (0.825 - 0.907)
IVW-mre	2	-0.152 (-0.216 - -0.087)	0.032847923	0.859 (0.806 - 0.916)
MR Egger	3	-0.15 (-0.742 - 0.443)	0.302250024	0.861 (0.476 - 1.557)
IVW-mre	3	-0.156 (-0.178 - -0.133)	0.011543082	0.856 (0.837 - 0.875)
Weighted median	3	-0.161 (-0.453 - 0.131)	0.148973115	0.852 (0.636 - 1.14)
Weighted mode	3	-0.162 (-0.509 - 0.184)	0.176690721	0.85 (0.601 - 1.202)
Simple mode	3	-0.163 (-0.496 - 0.17)	0.169963292	0.85 (0.609 - 1.186)
IVW-mre	2	-0.181 (-0.202 - -0.16)	0.010725509	0.835 (0.817 - 0.852)
IVW-mre	2	-0.079 (-0.092 - -0.066)	0.006585561	0.924 (0.912 - 0.936)
MR Egger	5	-0.091 (-0.261 - 0.078)	0.086434935	0.913 (0.77 - 1.081)
IVW-mre	5	-0.094 (-0.137 - -0.05)	0.022236862	0.91 (0.872 - 0.951)
Weighted median	5	-0.092 (-0.205 - 0.02)	0.057457799	0.912 (0.815 - 1.021)
Weighted mode	5	-0.082 (-0.2 - 0.036)	0.060078611	0.921 (0.819 - 1.036)
Simple mode	5	-0.046 (-0.212 - 0.12)	0.084603266	0.955 (0.809 - 1.127)
IVW-mre	2	-0.075 (-0.105 - -0.045)	0.015086385	0.928 (0.901 - 0.956)
IVW-mre	2	-0.235 (-0.25 - -0.22)	0.007749243	0.79 (0.778 - 0.803)
MR Egger	4	0.317 (-0.847 - 1.482)	0.594088869	1.374 (0.429 - 4.401)
IVW-mre	4	0.115 (0.058 - 0.172)	0.029084434	1.122 (1.059 - 1.187)
Weighted median	4	0.104 (-0.124 - 0.332)	0.116336427	1.11 (0.884 - 1.394)

Weighted mode	4 0.083 (-0.208 - 0.373)	0.148025507	1.086 (0.813 - 1.452)
Simple mode	4 0.091 (-0.196 - 0.379)	0.146557704	1.096 (0.822 - 1.46)
IVW-mre	2 0.144 (0.066 - 0.223)	0.040162208	1.155 (1.068 - 1.25)
IVW-mre	2 0.185 (0.094 - 0.276)	0.046373941	1.204 (1.099 - 1.318)
Wald ratio	1 0.729 (0.421 - 1.037)	0.157271184	2.073 (1.523 - 2.821)
IVW-mre	2 -0.315 (-0.4 - -0.23)	0.043467205	0.73 (0.67 - 0.795)
IVW-mre	2 -0.069 (-0.081 - -0.056)	0.006259907	0.934 (0.922 - 0.945)
MR Egger	4 0.091 (-0.602 - 0.784)	0.353647122	1.095 (0.548 - 2.19)
IVW-mre	4 0.113 (0.096 - 0.13)	0.008706325	1.12 (1.101 - 1.139)
Weighted median	4 0.115 (-0.043 - 0.273)	0.080687928	1.122 (0.958 - 1.314)
Weighted mode	4 0.125 (-0.055 - 0.306)	0.092021135	1.133 (0.946 - 1.357)
Simple mode	4 0.124 (-0.073 - 0.32)	0.100380687	1.132 (0.929 - 1.378)
IVW-mre	2 -0.144 (-0.236 - -0.053)	0.046786242	0.866 (0.79 - 0.949)
IVW-mre	2 -0.077 (-0.104 - -0.05)	0.013634224	0.926 (0.901 - 0.951)
IVW-mre	2 -0.078 (-0.105 - -0.05)	0.014060406	0.925 (0.9 - 0.951)
MR Egger	3 0.023 (-0.056 - 0.103)	0.040615491	1.024 (0.945 - 1.108)
IVW-mre	3 0.018 (0.012 - 0.023)	0.002860169	1.018 (1.012 - 1.024)
Weighted median	3 0.018 (-0.025 - 0.062)	0.022286768	1.018 (0.975 - 1.064)
Weighted mode	3 0.019 (-0.028 - 0.066)	0.023928955	1.019 (0.972 - 1.068)
Simple mode	3 0.011 (-0.065 - 0.087)	0.03873708	1.011 (0.937 - 1.091)
MR Egger	3 0.026 (-0.096 - 0.148)	0.062160763	1.026 (0.908 - 1.159)
IVW-mre	3 0.019 (0.012 - 0.026)	0.003500288	1.019 (1.012 - 1.026)
Weighted median	3 0.02 (-0.043 - 0.082)	0.03197553	1.02 (0.958 - 1.086)
Weighted mode	3 0.02 (-0.048 - 0.088)	0.034634653	1.021 (0.954 - 1.092)
Simple mode	3 0.015 (-0.084 - 0.114)	0.050493895	1.015 (0.92 - 1.121)
MR Egger	3 0.026 (-0.058 - 0.109)	0.042567569	1.026 (0.944 - 1.115)
IVW-mre	3 0.018 (0.011 - 0.026)	0.003808321	1.018 (1.011 - 1.026)
Weighted median	3 0.018 (-0.028 - 0.065)	0.023755438	1.019 (0.972 - 1.067)
Weighted mode	3 0.02 (-0.028 - 0.068)	0.024646352	1.02 (0.972 - 1.071)
Simple mode	3 0.004 (-0.078 - 0.085)	0.041462157	1.004 (0.925 - 1.089)
MR Egger	3 0.023 (-0.057 - 0.103)	0.040744134	1.024 (0.945 - 1.109)
IVW-mre	3 0.018 (0.012 - 0.023)	0.002926748	1.018 (1.012 - 1.024)
Weighted median	3 0.018 (-0.028 - 0.064)	0.023341293	1.018 (0.973 - 1.066)
Weighted mode	3 0.019 (-0.025 - 0.063)	0.022420008	1.019 (0.975 - 1.065)
Simple mode	3 0.01 (-0.069 - 0.089)	0.04029696	1.01 (0.934 - 1.093)
MR Egger	3 0.023 (-0.056 - 0.103)	0.040636442	1.024 (0.945 - 1.108)
IVW-mre	3 0.018 (0.012 - 0.023)	0.002936248	1.018 (1.012 - 1.024)
Weighted median	3 0.018 (-0.027 - 0.063)	0.023122879	1.018 (0.973 - 1.065)
Weighted mode	3 0.019 (-0.027 - 0.065)	0.02340915	1.019 (0.973 - 1.067)
Simple mode	3 0.011 (-0.069 - 0.09)	0.040408462	1.011 (0.934 - 1.094)
IVW-mre	2 -0.144 (-0.186 - -0.102)	0.021374886	0.866 (0.83 - 0.903)
IVW-mre	2 0.287 (0.191 - 0.384)	0.049303247	1.333 (1.21 - 1.468)
IVW-mre	2 -0.091 (-0.145 - -0.038)	0.027516484	0.913 (0.865 - 0.963)
MR Egger	3 -0.046 (-0.296 - 0.204)	0.127527452	0.955 (0.744 - 1.227)
IVW-mre	3 -0.071 (-0.094 - -0.048)	0.011606186	0.932 (0.911 - 0.953)
Weighted median	3 -0.07 (-0.165 - 0.025)	0.048464468	0.932 (0.848 - 1.025)
Weighted mode	3 -0.068 (-0.168 - 0.033)	0.051313381	0.845 (1.034 - 0.319)
Simple mode	3 -0.058 (-0.207 - 0.09)	0.07586317	0.813 (1.094 - 0.522)
IVW-mre	2 0.095 (0.077 - 0.112)	0.009083978	1.099 (1.08 - 1.119)
IVW-mre	2 0.045 (0.021 - 0.068)	0.012014472	1.046 (1.022 - 1.071)
IVW-mre	2 -0.082 (-0.112 - -0.052)	0.015201943	0.922 (0.894 - 0.949)
IVW-mre	2 -0.082 (-0.113 - -0.052)	0.015724918	0.921 (0.893 - 0.95)
IVW-mre	2 -0.087 (-0.144 - -0.03)	0.028963937	0.917 (0.866 - 0.97)

Pval	FDR	Cochrane Q	Q pval	Egger Intercept	Pleiotropy pval
4.20E-66	8.10E-64	0.002361637	0.961241	NA	NA
0.003749	0.042560119	0.139404592	0.708874	NA	NA
2.60E-03	3.07E-02	0.335593805	0.562384	NA	NA
0.00106	0.013063667	0.040536977	0.840434	NA	NA
1.21E-05	1.95E-04	0.083307125	0.772865	NA	NA
0.723016	1	0.009228643	0.923468	NA	NA
2.34E-53	2.71E-51	0.014806775	0.992624	-0.009189377	0.952540957
0.188701	1	NA	NA	NA	NA
3.08E-01	1.00E+00	NA	NA	NA	NA
0.51409	1	NA	NA	NA	NA
5.73E-01	1.00E+00	0.008058771	0.928469	NA	NA
3.63E-33	2.1E-31	0.023810142	0.988166	-0.006793561	0.920516958
2.09E-01	1.00E+00	NA	NA	NA	NA
0.336076	1	NA	NA	NA	NA
6.47E-01	1.00E+00	NA	NA	NA	NA
2.91E-29	1.53E-27	0.016881831	0.896622	NA	NA
1.44E-27	6.39E-26	0.00927602	0.923273	NA	NA
6.33E-21	2.44E-19	0.007988474	0.928781	NA	NA
9.51E-04	1.20E-02	0.284237905	0.593937	NA	NA
0.003877	0.043169353	0.265053936	0.606669	NA	NA
2.77E-166	8.03E-164	0.000237613	0.987701	NA	NA
0.567256	1	0.789198759	0.374343	NA	NA
9.35E-05	1.29E-03	0.893063576	0.639843	-0.040947058	0.801520813
0.042767	1	NA	NA	NA	NA
1.24E-01	1.00E+00	NA	NA	NA	NA
0.387999	1	NA	NA	NA	NA
2.18E-20	7.89E-19	0.019334368	0.889412	NA	NA
1.02E-41	9.89E-40	0.009097388	0.924013	NA	NA
1.78E-10	5.14E-09	0.009854326	0.920925	NA	NA
3.31E-11	1.01E-09	0.009134391	0.923859	NA	NA
4.12E-05	6.11E-04	0.028023807	0.867053	NA	NA
0.004444	0.048544687	0.563829163	0.754338	-0.039743653	0.591658282
8.94E-01	1.00E+00	0.006184738	0.937316	NA	NA
0.265523	1	NA	NA	NA	NA
5.09E-01	1.00E+00	NA	NA	NA	NA
0.555723	1	NA	NA	NA	NA
2.29E-05	3.59E-04	0.077921326	0.780134	NA	NA
2.43E-09	5.62E-08	0.022272472	0.881365	NA	NA
3.79E-06	6.27E-05	0.065942631	0.797339	NA	NA
0.70717	1	0.01558575	0.900648	NA	NA
1.86E-41	1.54E-39	0.016058139	0.992003	-0.00127917	0.986165556
0.281229	1	NA	NA	NA	NA
4.55E-01	1.00E+00	NA	NA	NA	NA
0.439203	1	NA	NA	NA	NA
1.06E-63	1.53E-61	0.004249152	0.948026	NA	NA
3.57E-33	2.1E-31	0.011157176	0.915878	NA	NA
3.68E-01	1.00E+00	0.756361869	0.859874	NA	NA
2.42E-05	0.000368575	0.757713481	0.944026	-0.000834565	0.972982503
1.08E-01	1.00E+00	NA	NA	NA	NA
0.243513	1	NA	NA	NA	NA
6.15E-01	1.00E+00	NA	NA	NA	NA
6.63E-07	0.000012	0.065255103	0.798375	NA	NA
2.33E-202	1.35E-199	0.001825684	0.965918	NA	NA
0.646589	1	0.140226559	0.932288	NA	NA
7.98E-05	1.13E-03	0.259915628	0.967382	-0.039474748	0.762375528
0.369975	1	NA	NA	NA	NA

6.16E-01	1.00E+00	NA	NA	NA	NA
0.577023	1	NA	NA	NA	NA
3.22E-04	4.24E-03	0.175797214	0.67501	NA	NA
6.46E-05	0.000935481	0.13572392	0.71257	NA	NA
3.57E-06	6.07E-05	NA	NA	NA	NA
4.19E-13	1.43E-11	0.155850354	0.693006	NA	NA
6.29E-28	3.04E-26	0.003996199	0.949595	NA	NA
0.821285	1	0.044058765	0.978211	NA	NA
1.51E-38	1.09E-36	0.048153432	0.99723	0.00582159	0.954798779
0.153794	1	NA	NA	NA	NA
2.67E-01	1.00E+00	NA	NA	NA	NA
0.306059	1	NA	NA	NA	NA
2.05E-03	2.48E-02	0.139272689	0.709005	NA	NA
1.51E-08	0.000000324	0.028518413	0.865896	NA	NA
3.10E-08	6.41E-07	0.029776111	0.862999	NA	NA
0.669024	1	0.005711362	0.939758	NA	NA
3.96E-10	1.09E-08	0.031215519	0.984513	-0.005083331	0.899183021
0.4126	1	NA	NA	NA	NA
5.11E-01	1.00E+00	NA	NA	NA	NA
0.801227	1	NA	NA	NA	NA
7.49E-01	1.00E+00	0.007542961	0.930791	NA	NA
5.68E-08	0.00000113	0.023979451	0.988082	-0.004435603	0.918825054
5.38E-01	1.00E+00	NA	NA	NA	NA
0.615987	1	NA	NA	NA	NA
7.90E-01	1.00E+00	NA	NA	NA	NA
0.655526	1	0.007621659	0.930431	NA	NA
1.65E-06	2.90E-05	0.050882843	0.974879	-0.006447061	0.869448758
0.437341	1	NA	NA	NA	NA
5.00E-01	1.00E+00	NA	NA	NA	NA
0.936143	1	NA	NA	NA	NA
6.69E-01	1.00E+00	0.006061356	0.937944	NA	NA
1.31E-09	3.31E-08	0.033096053	0.983588	-0.005283068	0.896253774
4.37E-01	1.00E+00	NA	NA	NA	NA
0.490128	1	NA	NA	NA	NA
8.21E-01	1.00E+00	NA	NA	NA	NA
0.669333	1	0.006450126	0.935989	NA	NA
1.72E-09	4.14E-08	0.033548987	0.983365	-0.005294143	0.896132902
0.434527	1	NA	NA	NA	NA
5.08E-01	1.00E+00	NA	NA	NA	NA
0.818165	1	NA	NA	NA	NA
1.52E-11	4.89E-10	0.026605495	0.87043	NA	NA
5.61E-09	0.000000125	0.146039089	0.70235	NA	NA
8.84E-04	1.14E-02	0.139213537	0.709064	NA	NA
0.780723	1	0.070421119	0.790725	NA	NA
1.00E-09	2.64E-08	0.115808258	0.94374	-0.017862037	0.866370664
0.148582	1	NA	NA	NA	NA
1.00E+00	NA	NA	NA	NA	NA
1	NA	NA	NA	NA	NA
2.14E-25	8.86E-24	0.010207288	0.919526	NA	NA
0.000189	0.002543635	0.032053386	0.857911	NA	NA
7.58E-08	1.46E-06	0.031500576	0.859128	NA	NA
1.62E-07	0.00000303	0.033129742	0.85557	NA	NA
2.72E-03	3.15E-02	0.168016974	0.68188	NA	NA

Supplementary Table 4

Id.exposure	Outcome	Exposure
GCST90001473	IPF	CD62L- CD86+ myeloid Dendritic Cell %Dendritic Cell
GCST90001957	IPF	CD33 on CD33+ HLA DR+ CD14-
GCST90001956	IPF	CD33 on CD33+ HLA DR+
GCST90001947	IPF	CD33 on CD33+ HLA DR+ CD14dim
GCST90002005	IPF	CD16 on CD14+ CD16+ monocyte
GCST90001952	IPF	CD33 on Monocytic Myeloid-Derived Suppressor Cells
GCST90001465	IPF	CD86+ myeloid Dendritic Cell %Dendritic Cell
GCST90001650	IPF	HLA DR+ Natural Killer %CD3- lymphocyte
GCST90001648	IPF	HLA DR+ Natural Killer Absolute Count
GCST90001585	IPF	CD14+ CD16+ monocyte %monocyte
GCST90001649	IPF	HLA DR+ Natural Killer %Natural Killer
GCST90001458	IPF	Myeloid Dendritic Cell Absolute Count
GCST90001580	IPF	CD14+ CD16+ monocyte Absolute Count
GCST90001474	IPF	Plasmacytoid Dendritic Cell %Dendritic Cell
GCST90002077	IPF	SSC-A on HLA DR+ Natural Killer
GCST90001970	IPF	FSC-A on HLA DR+ Natural Killer
GCST90001950	IPF	CD33 on Granulocytic Myeloid-Derived Suppressor Cells
GCST90001586	IPF	CD14+ CD16- monocyte %monocyte
GCST90001587	IPF	CD16+ monocyte %monocyte
GCST90002017	IPF	CCR2 on monocyte
GCST90001944	IPF	CD123 on plasmacytoid Dendritic Cell
GCST90001945	IPF	CD123 on CD62L+ plasmacytoid Dendritic Cell
GCST90001870	IPF	CD34 on Hematopoietic Stem Cell
GCST90002037	IPF	CD80 on plasmacytoid Dendritic Cell
GCST90002038	IPF	CD80 on CD62L+ plasmacytoid Dendritic Cell
GCST90001952	IPF	CD33 on Monocytic Myeloid-Derived Suppressor Cells
GCST90001527	IPF	CD33dim HLA DR+ CD11b- Absolute Count
GCST90001781	IPF	CD25 on IgD+ CD38- naive B cell
GCST90002017	IPF	CCR2 on monocyte
GCST90001783	IPF	CD25 on IgD+ CD38+ B cell
GCST90001520	IPF	CD33+ HLA DR+ CD14dim Absolute Count
GCST90001957	IPF	CD33 on CD33+ HLA DR+ CD14-
GCST90001436	IPF	Memory B cell %lymphocyte
GCST90001956	IPF	CD33 on CD33+ HLA DR+
GCST90001558	IPF	Terminally Differentiated CD8+ T cell %CD8+ T cell
GCST90001956	IPF	CD33 on CD33+ HLA DR+
GCST90001588	IPF	T/B cell
GCST90001957	IPF	CD33 on CD33+ HLA DR+ CD14-
GCST90001473	IPF	CD62L- CD86+ myeloid Dendritic Cell %Dendritic Cell
GCST90001947	IPF	CD33 on CD33+ HLA DR+ CD14dim
GCST90001947	IPF	CD33 on CD33+ HLA DR+ CD14dim
GCST90001649	IPF	HLA DR+ Natural Killer %Natural Killer
GCST90002014	IPF	CCR2 on CD62L+ myeloid Dendritic Cell
GCST90001911	IPF	CD45 on Natural Killer
GCST90001597	IPF	CD8dim T cell %T cell
GCST90002005	IPF	CD16 on CD14+ CD16+ monocyte
GCST90001952	IPF	CD33 on Monocytic Myeloid-Derived Suppressor Cells
GCST90002014	IPF	CCR2 on CD62L+ myeloid Dendritic Cell
GCST90001870	IPF	CD34 on Hematopoietic Stem Cell
GCST90001424	IPF	IgD+ B cell %Lymphocyte
GCST90001597	IPF	CD8dim T cell %T cell
GCST90001612	IPF	CD8dim T cell %leukocyte
GCST90001867	IPF	CD3 on CD4+ T cell
GCST90001465	IPF	CD86+ myeloid Dendritic Cell %Dendritic Cell
GCST90001612	IPF	CD8dim T cell %leukocyte
GCST90001596	IPF	CD8dim T cell Absolute Count

GCST90001911	IPF	CD45 on Natural Killer
GCST90001944	IPF	CD123 on plasmacytoid Dendritic Cell
GCST90001547	IPF	Terminally Differentiated CD4+ T cell %T cell
GCST90001945	IPF	CD123 on CD62L+ plasmacytoid Dendritic Cell
GCST90001748	IPF	CD20 on IgD+ CD38- B cell
GCST90001596	IPF	CD8dim T cell Absolute Count
GCST90001599	IPF	CD4-CD8- T cell %T cell
GCST90001554	IPF	Effector Memory CD8+ T cell Absolute Count
GCST90001580	IPF	CD14+ CD16+ monocyte Absolute Count
GCST90001558	IPF	Terminally Differentiated CD8+ T cell %CD8+ T cell
GCST90002037	IPF	CD80 on plasmacytoid Dendritic Cell
GCST90002077	IPF	SSC-A on HLA DR+ Natural Killer
GCST90002038	IPF	CD80 on CD62L+ plasmacytoid Dendritic Cell
GCST90001475	IPF	HLA DR++ monocyte %monocyte
GCST90001950	IPF	CD33 on Granulocytic Myeloid-Derived Suppressor Cells
GCST90001588	IPF	T/B cell
GCST90001474	IPF	Plasmacytoid Dendritic Cell %Dendritic Cell
GCST90001959	IPF	CD4 on HLA DR+ CD4+ T cell
GCST90001475	IPF	HLA DR++ monocyte %monocyte
GCST90001648	IPF	HLA DR+ Natural Killer Absolute Count
GCST90001833	IPF	CD62L on CD62L+ Dendritic Cell
GCST90001748	IPF	CD20 on IgD+ CD38- B cell
GCST90001650	IPF	HLA DR+ Natural Killer %CD3- lymphocyte
GCST90001833	IPF	CD62L on CD62L+ Dendritic Cell
GCST90001959	IPF	CD4 on HLA DR+ CD4+ T cell
GCST90001613	IPF	CD4-CD8- T cell %leukocyte
GCST90001585	IPF	CD14+ CD16+ monocyte %monocyte
GCST90001458	IPF	Myeloid Dendritic Cell Absolute Count
GCST90001748	IPF	CD20 on IgD+ CD38- B cell
GCST90001520	IPF	CD33+ HLA DR+ CD14dim Absolute Count
GCST90001424	IPF	IgD+ B cell %Lymphocyte
GCST90001649	IPF	HLA DR+ Natural Killer %Natural Killer
GCST90001950	IPF	CD33 on Granulocytic Myeloid-Derived Suppressor Cells
GCST90001783	IPF	CD25 on IgD+ CD38+ B cell
GCST90001614	IPF	CD8+ and CD8dim T cell %leukocyte
GCST90001870	IPF	CD34 on Hematopoietic Stem Cell
GCST90001614	IPF	CD8+ and CD8dim T cell %leukocyte
GCST90001690	IPF	CD28+ CD45RA+ CD8+ T cell Absolute Count
GCST90001554	IPF	Effector Memory CD8+ T cell Absolute Count
GCST90001748	IPF	CD20 on IgD+ CD38- B cell
GCST90001587	IPF	CD16+ monocyte %monocyte
GCST90001970	IPF	FSC-A on HLA DR+ Natural Killer
GCST90001870	IPF	CD34 on Hematopoietic Stem Cell
GCST90001392	IPF	IgD+ CD38+ B cell Absolute Count
GCST90001586	IPF	CD14+ CD16- monocyte %monocyte
GCST90001867	IPF	CD3 on CD4+ T cell
GCST90001392	IPF	IgD+ CD38+ B cell Absolute Count
GCST90001473	IPF	CD62L- CD86+ myeloid Dendritic Cell %Dendritic Cell
GCST90001426	IPF	IgD- CD38dim B cell %lymphocyte
GCST90001599	IPF	CD4-CD8- T cell %T cell
GCST90001965	IPF	FSC-A on monocyte
GCST90001465	IPF	CD86+ myeloid Dendritic Cell %Dendritic Cell
GCST90001649	IPF	HLA DR+ Natural Killer %Natural Killer
GCST90001527	IPF	CD33dim HLA DR+ CD11b- Absolute Count
GCST90001965	IPF	FSC-A on monocyte
GCST90001781	IPF	CD25 on IgD+ CD38- naive B cell
GCST90001613	IPF	CD4-CD8- T cell %leukocyte
GCST90001436	IPF	Memory B cell %lymphocyte

GCST90001558	IPF	Terminally Differentiated CD8+ T cell %CD8+ T cell
GCST90001613	IPF	CD4-CD8- T cell %leukocyte
GCST90002005	IPF	CD16 on CD14+ CD16+ monocyte
GCST90001690	IPF	CD28+ CD45RA+ CD8+ T cell Absolute Count
GCST90001649	IPF	HLA DR+ Natural Killer %Natural Killer
GCST90001827	IPF	IgD on IgD+ B cell
GCST90001547	IPF	Terminally Differentiated CD4+ T cell %T cell
GCST90001596	IPF	CD8dim T cell Absolute Count
GCST90001426	IPF	IgD- CD38dim B cell %lymphocyte

Types	SNP	Samplesize.outcome	Samplesize.exposure
leukocyte count	rs71632979	24589	3410
blood protein measurement	rs3865444	24589	1578
blood protein measurement	rs3865444	24589	1579
blood protein measurement	rs3865444	24589	1577
blood protein measurement	rs71632979	24589	3617
blood protein measurement	rs3865444	24589	1569
leukocyte count	rs71632979	24589	3410
leukocyte count	rs71632979	24589	3596
leukocyte count	rs71632989	24589	3580
monocyte count	rs71632979	24589	3629
leukocyte count	rs71632989	24589	3596
leukocyte count	rs71632979	24589	3387
monocyte count	rs71632979	24589	3629
leukocyte count	rs71632989	24589	3410
blood protein measurement	rs71632979	24589	2971
blood protein measurement	rs71632979	24589	2971
blood protein measurement	rs7245846	24589	1244
monocyte count	rs115383270	24589	3629
monocyte count	rs115383270	24589	3629
blood protein measurement	rs4683184	24589	2850
blood protein measurement	rs7658518	24589	2872
blood protein measurement	rs7658518	24589	2872
blood protein measurement	rs139489968	24589	1634
blood protein measurement	rs7658518	24589	2871
blood protein measurement	rs7658518	24589	2871
blood protein measurement	rs7351079	24589	1569
myeloid white cell count	rs67760360	24589	1858
blood protein measurement	rs41294937	24589	1835
blood protein measurement	rs1801274	24589	2850
blood protein measurement	rs62626323	24589	3656
myeloid white cell count	rs3865444	24589	1858
blood protein measurement	rs7351079	24589	1578
lymphocyte count	rs12874404	24589	3656
blood protein measurement	rs7351079	24589	1579
lymphocyte count	rs6707421	24589	3427
blood protein measurement	rs78949787	24589	1579
leukocyte count	rs12874404	24589	3653
blood protein measurement	rs78949787	24589	1578
leukocyte count	rs71639910	24589	3410
blood protein measurement	rs78949787	24589	1577
blood protein measurement	rs7351079	24589	1577
leukocyte count	rs7180804	24589	3596
blood protein measurement	rs35587265	24589	2870
blood protein measurement	rs6721978	24589	3113
leukocyte count	rs139249541	24589	3668
blood protein measurement	rs71639910	24589	3617
blood protein measurement	rs62114147	24589	1569
blood protein measurement	rs6687275	24589	2870
blood protein measurement	rs4406271	24589	1634
lymphocyte count	rs12874404	24589	3657
leukocyte count	rs79086781	24589	3668
leukocyte count	rs139249541	24589	3668
blood protein measurement	rs4657649	24589	2919
leukocyte count	rs71639910	24589	3410
leukocyte count	rs79086781	24589	3668
leukocyte count	rs139249541	24589	3652

blood protein measurement	rs183053322	24589	3113
blood protein measurement	rs62447171	24589	2872
lymphocyte count	rs62400929	24589	3427
blood protein measurement	rs62447171	24589	2872
blood protein measurement	rs30003	24589	3645
leukocyte count	rs79251210	24589	3652
leukocyte count	rs139249541	24589	3668
lymphocyte count	rs17583875	24589	3395
monocyte count	rs71639910	24589	3629
lymphocyte count	rs79827501	24589	3427
blood protein measurement	rs62447171	24589	2871
blood protein measurement	rs569911	24589	2971
blood protein measurement	rs62447171	24589	2871
leukocyte count	rs9296042	24589	3595
blood protein measurement	rs7351079	24589	1244
leukocyte count	rs9892810	24589	3653
leukocyte count	rs876036	24589	3410
blood protein measurement	rs4371876	24589	3060
leukocyte count	rs62395272	24589	3595
leukocyte count	rs71639910	24589	3580
blood protein measurement	rs3917768	24589	2871
blood protein measurement	rs12554596	24589	3645
leukocyte count	rs569911	24589	3596
blood protein measurement	rs7526645	24589	2871
blood protein measurement	rs9357097	24589	3060
leukocyte count	rs139249541	24589	3668
monocyte count	rs71639910	24589	3629
leukocyte count	rs569911	24589	3387
blood protein measurement	rs2070770	24589	3645
myeloid white cell count	rs74253876	24589	1858
lymphocyte count	rs11657531	24589	3657
leukocyte count	rs9916257	24589	3596
blood protein measurement	rs78949787	24589	1244
blood protein measurement	rs3793662	24589	3656
leukocyte count	rs11080350	24589	3668
blood protein measurement	rs586030	24589	1634
leukocyte count	rs73402222	24589	3668
lymphocyte count	rs3131627	24589	3408
lymphocyte count	rs3130685	24589	3395
blood protein measurement	rs2066399	24589	3645
monocyte count	rs74975395	24589	3629
blood protein measurement	rs569911	24589	2971
blood protein measurement	rs36102158	24589	1634
lymphocyte count	rs80330977	24589	3656
monocyte count	rs74975395	24589	3629
blood protein measurement	rs41289614	24589	2919
lymphocyte count	rs139260757	24589	3656
leukocyte count	rs78600824	24589	3410
lymphocyte count	rs7840395	24589	3656
leukocyte count	rs79251210	24589	3668
blood protein measurement	rs77762937	24589	2850
leukocyte count	rs115624142	24589	3410
leukocyte count	rs569911	24589	3596
myeloid white cell count	rs185048341	24589	1858
blood protein measurement	rs1800973	24589	2850
blood protein measurement	rs2275848	24589	1835
leukocyte count	rs79251210	24589	3668
lymphocyte count	rs11653761	24589	3656

lymphocyte count	rs13023673	24589	3427
leukocyte count	rs9896155	24589	3668
blood protein measurement	rs147678401	24589	3617
lymphocyte count	rs12901644	24589	3408
leukocyte count	rs865239	24589	3596
blood protein measurement	rs28669075	24589	3657
lymphocyte count	rs9501107	24589	3427
leukocyte count	rs9916629	24589	3652
lymphocyte count	rs11687597	24589	3656

Steiger_dir	Steiger_pval	Steiger_FDR
TRUE	1.12E-202	1.38E-200
TRUE	2.36E-191	1.45E-189
TRUE	1.05E-189	4.30E-188
TRUE	5.73E-186	1.76E-184
TRUE	4.47E-184	1.10E-182
TRUE	4.05E-153	8.31E-152
TRUE	1.37E-145	2.41E-144
TRUE	8.14E-123	1.25E-121
TRUE	3.65E-118	4.99E-117
TRUE	3.76E-116	4.62E-115
TRUE	2.09E-107	2.34E-106
TRUE	2.29E-97	2.35E-96
TRUE	2.82E-97	2.67E-96
TRUE	1.43E-78	1.26E-77
TRUE	1.16E-64	9.51E-64
TRUE	6.59E-60	5.06E-59
TRUE	5.91E-57	4.28E-56
TRUE	2.43E-56	1.66E-55
TRUE	1.71E-54	1.11E-53
TRUE	7.08E-24	4.36E-23
TRUE	8.96E-20	5.25E-19
TRUE	2.51E-19	1.40E-18
TRUE	1.16E-18	6.18E-18
TRUE	1.54E-17	7.88E-17
TRUE	3.84E-17	1.89E-16
TRUE	6.67E-17	3.15E-16
TRUE	6.90E-17	3.15E-16
TRUE	1.57E-16	6.91E-16
TRUE	3.65E-16	1.55E-15
TRUE	5.38E-15	2.21E-14
TRUE	5.66E-15	2.25E-14
TRUE	6.46E-15	2.48E-14
TRUE	6.77E-15	2.52E-14
TRUE	1.47E-14	5.30E-14
TRUE	2.26E-14	7.93E-14
TRUE	2.38E-14	7.94E-14
TRUE	2.39E-14	7.94E-14
TRUE	2.73E-14	8.85E-14
TRUE	2.81E-14	8.85E-14
TRUE	9.41E-14	2.89E-13
TRUE	1.23E-13	3.70E-13
TRUE	2.85E-13	8.34E-13
TRUE	3.26E-13	9.31E-13
TRUE	3.71E-13	1.04E-12
TRUE	4.60E-13	1.26E-12
TRUE	1.21E-12	3.24E-12
TRUE	1.14E-11	2.97E-11
TRUE	1.16E-11	2.97E-11
TRUE	1.88E-11	4.71E-11
TRUE	2.05E-11	5.05E-11
TRUE	3.22E-11	7.76E-11
TRUE	4.47E-11	1.06E-10
TRUE	5.35E-11	1.24E-10
TRUE	1.34E-10	3.06E-10
TRUE	2.77E-10	6.19E-10
TRUE	4.29E-10	9.41E-10

TRUE	6.66E-10	1.44E-09
TRUE	1.04E-09	2.20E-09
TRUE	1.07E-09	2.23E-09
TRUE	1.19E-09	2.45E-09
TRUE	1.22E-09	2.46E-09
TRUE	3.24E-09	6.43E-09
TRUE	3.47E-09	6.69E-09
TRUE	3.48E-09	6.69E-09
TRUE	4.46E-09	8.44E-09
TRUE	4.79E-09	8.93E-09
TRUE	5.11E-09	9.39E-09
TRUE	5.45E-09	9.76E-09
TRUE	5.47E-09	9.76E-09
TRUE	6.79E-09	1.19E-08
TRUE	1.18E-08	2.04E-08
TRUE	1.24E-08	2.13E-08
TRUE	1.29E-08	2.15E-08
TRUE	1.29E-08	2.15E-08
TRUE	1.48E-08	2.43E-08
TRUE	1.79E-08	2.90E-08
TRUE	2.22E-08	3.55E-08
TRUE	2.31E-08	3.65E-08
TRUE	3.54E-08	5.51E-08
TRUE	4.05E-08	6.22E-08
TRUE	5.50E-08	8.36E-08
TRUE	5.97E-08	8.96E-08
TRUE	6.10E-08	9.03E-08
TRUE	6.24E-08	9.14E-08
TRUE	6.56E-08	9.49E-08
TRUE	6.72E-08	9.61E-08
TRUE	6.96E-08	9.84E-08
TRUE	7.24E-08	1.01E-07
TRUE	7.29E-08	1.01E-07
TRUE	7.67E-08	1.05E-07
TRUE	7.83E-08	1.06E-07
TRUE	1.21E-07	1.62E-07
TRUE	1.27E-07	1.68E-07
TRUE	1.36E-07	1.78E-07
TRUE	1.53E-07	1.99E-07
TRUE	1.96E-07	2.51E-07
TRUE	2.01E-07	2.52E-07
TRUE	2.02E-07	2.52E-07
TRUE	2.03E-07	2.52E-07
TRUE	2.24E-07	2.75E-07
TRUE	2.39E-07	2.91E-07
TRUE	2.93E-07	3.53E-07
TRUE	3.24E-07	3.87E-07
TRUE	3.32E-07	3.92E-07
TRUE	3.39E-07	3.97E-07
TRUE	3.71E-07	4.30E-07
TRUE	3.86E-07	4.44E-07
TRUE	4.37E-07	4.98E-07
TRUE	4.42E-07	4.99E-07
TRUE	6.20E-07	6.93E-07
TRUE	6.76E-07	7.49E-07
TRUE	7.77E-07	8.48E-07
TRUE	7.79E-07	8.48E-07
TRUE	7.95E-07	8.58E-07

TRUE	8.25E-07	8.82E-07
TRUE	8.77E-07	9.30E-07
TRUE	1.14E-06	1.20E-06
TRUE	1.19E-06	1.24E-06
TRUE	1.54E-06	1.59E-06
TRUE	1.66E-06	1.70E-06
TRUE	2.19E-06	2.23E-06
TRUE	2.64E-06	2.67E-06
TRUE	6.97E-06	6.97E-06

Supplementary Table 5

Exposure	Id.exposure	Types	Outcome
SSC-A on Natural Killer	GCST90002076	blood protein measurement	IPF
FSC-A on Natural Killer	GCST90001969	blood protein measurement	IPF
FSC-A on Natural Killer	GCST90001969	blood protein measurement	IPF
FSC-A on Natural Killer	GCST90001969	blood protein measurement	IPF
CD20 on IgD+ CD38dim B cell	GCST90001752	blood protein measurement	IPF
CD20 on IgD+ CD38dim B cell	GCST90001752	blood protein measurement	IPF
CD20 on IgD+ CD38dim B cell	GCST90001752	blood protein measurement	IPF
CD20 on IgD+ CD38dim B cell	GCST90001752	blood protein measurement	IPF
CD20 on IgD+ CD38dim B cell	GCST90001752	blood protein measurement	IPF
CD20 on IgD+ CD38dim B cell	GCST90001752	blood protein measurement	IPF
CD20 on IgD+ CD38dim B cell	GCST90001752	blood protein measurement	IPF
CD45RA+ CD28- CD8+ T cell Ab	GCST90001698	lymphocyte count	IPF
CD45RA+ CD28- CD8+ T cell Ab	GCST90001698	lymphocyte count	IPF
CD45RA+ CD28- CD8+ T cell Ab	GCST90001698	lymphocyte count	IPF
CD45RA+ CD28- CD8+ T cell Ab	GCST90001698	lymphocyte count	IPF
CD45RA+ CD28- CD8+ T cell Ab	GCST90001698	lymphocyte count	IPF
CD45RA+ CD28- CD8+ T cell Ab	GCST90001698	lymphocyte count	IPF
CD45RA+ CD28- CD8+ T cell Ab	GCST90001698	lymphocyte count	IPF
CD45RA+ CD28- CD8+ T cell Ab	GCST90001698	lymphocyte count	IPF
CD45RA+ CD28- CD8+ T cell Ab	GCST90001698	lymphocyte count	IPF
CD45RA+ CD28- CD8+ T cell Ab	GCST90001698	lymphocyte count	IPF
CD45RA+ CD28- CD8+ T cell Ab	GCST90001698	lymphocyte count	IPF
CD45RA+ CD28- CD8+ T cell Ab	GCST90001698	lymphocyte count	IPF
CD45RA+ CD28- CD8+ T cell Ab	GCST90001698	lymphocyte count	IPF
CD45RA+ CD28- CD8+ T cell Ab	GCST90001698	lymphocyte count	IPF
CD45RA+ CD28- CD8+ T cell Ab	GCST90001698	lymphocyte count	IPF
CD45RA+ CD28- CD8+ T cell Ab	GCST90001698	lymphocyte count	IPF
CD45RA+ CD28- CD8+ T cell Ab	GCST90001698	lymphocyte count	IPF
CD45RA+ CD28- CD8+ T cell Ab	GCST90001698	lymphocyte count	IPF
CD45RA+ CD28- CD8+ T cell Ab	GCST90001698	lymphocyte count	IPF
CD45RA+ CD28- CD8+ T cell Ab	GCST90001698	lymphocyte count	IPF
CD45RA+ CD28- CD8+ T cell Ab	GCST90001698	lymphocyte count	IPF
CD45RA+ CD28- CD8+ T cell Ab	GCST90001698	lymphocyte count	IPF
CD45RA+ CD28- CD8+ T cell Ab	GCST90001698	lymphocyte count	IPF
CD45RA+ CD28- CD8+ T cell Ab	GCST90001698	lymphocyte count	IPF
CD45RA+ CD28- CD8+ T cell Ab	GCST90001698	lymphocyte count	IPF
CD45RA+ CD28- CD8+ T cell Ab	GCST90001698	lymphocyte count	IPF
CD45RA+ CD28- CD8+ T cell Ab	GCST90001698	lymphocyte count	IPF
CD45RA+ CD28- CD8+ T cell Ab	GCST90001698	lymphocyte count	IPF
CD45RA+ CD28- CD8+ T cell Ab	GCST90001698	lymphocyte count	IPF
CD45RA+ CD28- CD8+ T cell Ab	GCST90001698	lymphocyte count	IPF
CD45RA+ CD28- CD8+ T cell Ab	GCST90001698	lymphocyte count	IPF
CD45RA+ CD28- CD8+ T cell Ab	GCST90001698	lymphocyte count	IPF
CD45RA+ CD28- CD8+ T cell Ab	GCST90001698	lymphocyte count	IPF
CD45RA+ CD28- CD8+ T cell Ab	GCST90001698	lymphocyte count	IPF
CD45RA+ CD28- CD8+ T cell Ab	GCST90001698	lymphocyte count	IPF
CD45RA+ CD28- CD8+ T cell Ab	GCST90001698	lymphocyte count	IPF
CD45RA+ CD28- CD8+ T cell Ab	GCST90001698	lymphocyte count	IPF
CD45RA+ CD28- CD8+ T cell Ab	GCST90001698	lymphocyte count	IPF
CD45RA+ CD28- CD8+ T cell Ab	GCST90001698	lymphocyte count	IPF
CD45RA+ CD28- CD8+ T cell Ab	GCST90001698	lymphocyte count	IPF
CD45RA+ CD28- CD8+ T cell Ab	GCST90001698	lymphocyte count	IPF
CD45RA+ CD28- CD8+ T cell Ab	GCST90001698	lymphocyte count	IPF
CD45RA+ CD28- CD8+ T cell Ab	GCST90001698	lymphocyte count	IPF
CD45RA+ CD28- CD8+ T cell Ab	GCST90001698	lymphocyte count	IPF
CD45RA+ CD28- CD8+ T cell Ab	GCST90001698	lymphocyte count	IPF

CD45RA+ CD28- CD8+ T cell Ab	GCST90001698	lymphocyte count	IPF
CD45RA+ CD28- CD8+ T cell Ab	GCST90001698	lymphocyte count	IPF
CD45RA+ CD28- CD8+ T cell Ab	GCST90001698	lymphocyte count	IPF
CD45RA+ CD28- CD8+ T cell Ab	GCST90001698	lymphocyte count	IPF
CD45RA+ CD28- CD8+ T cell Ab	GCST90001698	lymphocyte count	IPF
CD45RA+ CD28- CD8+ T cell Ab	GCST90001698	lymphocyte count	IPF
CD45RA+ CD28- CD8+ T cell Ab	GCST90001698	lymphocyte count	IPF
CD45RA+ CD28- CD8+ T cell Ab	GCST90001698	lymphocyte count	IPF
CD45RA+ CD28- CD8+ T cell Ab	GCST90001698	lymphocyte count	IPF
CD45RA+ CD28- CD8+ T cell Ab	GCST90001698	lymphocyte count	IPF
CD45RA+ CD28- CD8+ T cell Ab	GCST90001698	lymphocyte count	IPF
CD45RA+ CD28- CD8+ T cell Ab	GCST90001698	lymphocyte count	IPF
CD45RA+ CD28- CD8+ T cell Ab	GCST90001698	lymphocyte count	IPF
CD45RA+ CD28- CD8+ T cell Ab	GCST90001698	lymphocyte count	IPF
CD45RA+ CD28- CD8+ T cell Ab	GCST90001698	lymphocyte count	IPF
CD45RA+ CD28- CD8+ T cell Ab	GCST90001698	lymphocyte count	IPF
CD45RA+ CD28- CD8+ T cell Ab	GCST90001698	lymphocyte count	IPF
CD45RA+ CD28- CD8+ T cell Ab	GCST90001698	lymphocyte count	IPF
CD45RA+ CD28- CD8+ T cell Ab	GCST90001698	lymphocyte count	IPF
CD45RA+ CD28- CD8+ T cell Ab	GCST90001698	lymphocyte count	IPF
CD45RA+ CD28- CD8+ T cell Ab	GCST90001698	lymphocyte count	IPF
CD45RA+ CD28- CD8+ T cell Ab	GCST90001698	lymphocyte count	IPF
CD45RA+ CD28- CD8+ T cell Ab	GCST90001698	lymphocyte count	IPF
CD45RA+ CD28- CD8+ T cell Ab	GCST90001698	lymphocyte count	IPF
CD45RA+ CD28- CD8+ T cell Ab	GCST90001698	lymphocyte count	IPF
CD45RA+ CD28- CD8+ T cell Ab	GCST90001698	lymphocyte count	IPF
CD45RA+ CD28- CD8+ T cell Ab	GCST90001698	lymphocyte count	IPF
CD45RA+ CD28- CD8+ T cell Ab	GCST90001698	lymphocyte count	IPF
CD45RA+ CD28- CD8+ T cell Ab	GCST90001698	lymphocyte count	IPF
CD45RA+ CD28- CD8+ T cell Ab	GCST90001698	lymphocyte count	IPF
CD45RA+ CD28- CD8+ T cell Ab	GCST90001698	lymphocyte count	IPF
CD45RA+ CD28- CD8+ T cell Ab	GCST90001698	lymphocyte count	IPF
CD45RA+ CD28- CD8+ T cell Ab	GCST90001698	lymphocyte count	IPF
CD45RA+ CD28- CD8+ T cell Ab	GCST90001698	lymphocyte count	IPF
CD45RA+ CD28- CD8+ T cell Ab	GCST90001698	lymphocyte count	IPF
CD45RA+ CD28- CD8+ T cell Ab	GCST90001698	lymphocyte count	IPF
CD45RA+ CD28- CD8+ T cell Ab	GCST90001698	lymphocyte count	IPF
CD45RA+ CD28- CD8+ T cell Ab	GCST90001698	lymphocyte count	IPF
CD45RA+ CD28- CD8+ T cell Ab	GCST90001698	lymphocyte count	IPF
CD45RA+ CD28- CD8+ T cell Ab	GCST90001698	lymphocyte count	IPF
CD45RA+ CD28- CD8+ T cell Ab	GCST90001698	lymphocyte count	IPF
CD45RA+ CD28- CD8+ T cell Ab	GCST90001698	lymphocyte count	IPF
CD45RA+ CD28- CD8+ T cell Ab	GCST90001698	lymphocyte count	IPF
CD45RA+ CD28- CD8+ T cell Ab	GCST90001698	lymphocyte count	IPF
CD45RA+ CD28- CD8+ T cell Ab	GCST90001698	lymphocyte count	IPF
CD45RA+ CD28- CD8+ T cell Ab	GCST90001698	lymphocyte count	IPF
CD45RA+ CD28- CD8+ T cell Ab	GCST90001698	lymphocyte count	IPF
CD45RA+ CD28- CD8+ T cell Ab	GCST90001698	lymphocyte count	IPF
CD45RA+ CD28- CD8+ T cell Ab	GCST90001698	lymphocyte count	IPF
CD45RA+ CD28- CD8+ T cell Ab	GCST90001698	lymphocyte count	IPF
CD45RA+ CD28- CD8+ T cell Ab	GCST90001698	lymphocyte count	IPF
CD45RA+ CD28- CD8+ T cell Ab	GCST90001698	lymphocyte count	IPF
CD45RA+ CD28- CD8+ T cell Ab	GCST90001698	lymphocyte count	IPF
CD45RA+ CD28- CD8+ T cell Ab	GCST90001698	lymphocyte count	IPF
CD45RA+ CD28- CD8+ T cell Ab	GCST90001698	lymphocyte count	IPF
CD45RA+ CD28- CD8+ T cell Ab	GCST90001698	lymphocyte count	IPF
CD45RA+ CD28- CD8+ T cell Ab	GCST90001698	lymphocyte count	IPF
CD45RA+ CD28- CD8+ T cell Ab	GCST90001698	lymphocyte count	IPF

CD45RA+ CD28- CD8+ T cell Ab	GCST90001698	lymphocyte count	IPF
CD45RA+ CD28- CD8+ T cell Ab	GCST90001698	lymphocyte count	IPF
CD45RA+ CD28- CD8+ T cell Ab	GCST90001698	lymphocyte count	IPF
CD45RA+ CD28- CD8+ T cell Ab	GCST90001698	lymphocyte count	IPF
CD45RA+ CD28- CD8+ T cell Ab	GCST90001698	lymphocyte count	IPF
CD45RA+ CD28- CD8+ T cell Ab	GCST90001698	lymphocyte count	IPF
CD45RA+ CD28- CD8+ T cell Ab	GCST90001698	lymphocyte count	IPF
CD45RA+ CD28- CD8+ T cell Ab	GCST90001698	lymphocyte count	IPF
CD45RA+ CD28- CD8+ T cell Ab	GCST90001698	lymphocyte count	IPF
CD45RA+ CD28- CD8+ T cell Ab	GCST90001698	lymphocyte count	IPF
CD45RA+ CD28- CD8+ T cell Ab	GCST90001698	lymphocyte count	IPF
CD45RA+ CD28- CD8+ T cell Ab	GCST90001698	lymphocyte count	IPF
CD45RA+ CD28- CD8+ T cell Ab	GCST90001698	lymphocyte count	IPF
CD45RA+ CD28- CD8+ T cell Ab	GCST90001698	lymphocyte count	IPF
CD45RA+ CD28- CD8+ T cell Ab	GCST90001698	lymphocyte count	IPF
CD45RA+ CD28- CD8+ T cell Ab	GCST90001698	lymphocyte count	IPF
CD45RA+ CD28- CD8+ T cell Ab	GCST90001698	lymphocyte count	IPF
CD45RA+ CD28- CD8+ T cell Ab	GCST90001698	lymphocyte count	IPF
CD45RA+ CD28- CD8+ T cell Ab	GCST90001698	lymphocyte count	IPF
CD45RA+ CD28- CD8+ T cell Ab	GCST90001698	lymphocyte count	IPF
CD45RA+ CD28- CD8+ T cell Ab	GCST90001698	lymphocyte count	IPF
CD45RA+ CD28- CD8+ T cell Ab	GCST90001698	lymphocyte count	IPF
CD45RA+ CD28- CD8+ T cell Ab	GCST90001698	lymphocyte count	IPF
CD45RA+ CD28- CD8+ T cell Ab	GCST90001698	lymphocyte count	IPF
CD45RA+ CD28- CD8+ T cell Ab	GCST90001698	lymphocyte count	IPF
CD45RA+ CD28- CD8+ T cell Ab	GCST90001698	lymphocyte count	IPF
CD45RA+ CD28- CD8+ T cell Ab	GCST90001698	lymphocyte count	IPF
CD45RA+ CD28- CD8+ T cell Ab	GCST90001698	lymphocyte count	IPF
CD45RA+ CD28- CD8+ T cell Ab	GCST90001698	lymphocyte count	IPF
CD45RA+ CD28- CD8+ T cell Ab	GCST90001698	lymphocyte count	IPF
CD45RA+ CD28- CD8+ T cell Ab	GCST90001698	lymphocyte count	IPF
CD45RA+ CD28- CD8+ T cell Ab	GCST90001698	lymphocyte count	IPF
CD45RA+ CD28- CD8+ T cell Ab	GCST90001698	lymphocyte count	IPF
CD45RA+ CD28- CD8+ T cell Ab	GCST90001698	lymphocyte count	IPF
CD45RA+ CD28- CD8+ T cell Ab	GCST90001698	lymphocyte count	IPF
CD45RA+ CD28- CD8+ T cell Ab	GCST90001698	lymphocyte count	IPF
CD45RA+ CD28- CD8+ T cell Ab	GCST90001698	lymphocyte count	IPF
CD45RA+ CD28- CD8+ T cell Ab	GCST90001698	lymphocyte count	IPF
CD45RA+ CD28- CD8+ T cell Ab	GCST90001698	lymphocyte count	IPF
CD45RA+ CD28- CD8+ T cell Ab	GCST90001698	lymphocyte count	IPF
CD45RA+ CD28- CD8+ T cell Ab	GCST90001698	lymphocyte count	IPF
CD45RA+ CD28- CD8+ T cell Ab	GCST90001698	lymphocyte count	IPF
CD45RA+ CD28- CD8+ T cell Ab	GCST90001698	lymphocyte count	IPF
CD45RA+ CD28- CD8+ T cell Ab	GCST90001698	lymphocyte count	IPF
CD45RA+ CD28- CD8+ T cell Ab	GCST90001698	lymphocyte count	IPF
CD45RA+ CD28- CD8+ T cell Ab	GCST90001698	lymphocyte count	IPF
CD45RA+ CD28- CD8+ T cell Ab	GCST90001698	lymphocyte count	IPF
CD45RA+ CD28- CD8+ T cell Ab	GCST90001698	lymphocyte count	IPF
CD45RA+ CD28- CD8+ T cell Ab	GCST90001698	lymphocyte count	IPF
CD45RA+ CD28- CD8+ T cell Ab	GCST90001698	lymphocyte count	IPF
CD45RA+ CD28- CD8+ T cell Ab	GCST90001698	lymphocyte count	IPF
CD45RA+ CD28- CD8+ T cell Ab	GCST90001698	lymphocyte count	IPF
CD45RA+ CD28- CD8+ T cell Ab	GCST90001698	lymphocyte count	IPF
CD45RA+ CD28- CD8+ T cell Ab	GCST90001698	lymphocyte count	IPF
CD45RA+ CD28- CD8+ T cell Ab	GCST90001698	lymphocyte count	IPF
CD45RA+ CD28- CD8+ T cell Ab	GCST90001698	lymphocyte count	IPF
CD45RA+ CD28- CD8+ T cell Ab	GCST90001698	lymphocyte count	IPF
CD45RA+ CD28- CD8+ T cell Ab	GCST90001698	lymphocyte count	IPF

CD45RA+ CD28- CD8+ T cell Ab	GCST90001698	lymphocyte count	IPF
CD45RA+ CD28- CD8+ T cell Ab	GCST90001698	lymphocyte count	IPF
CD45RA+ CD28- CD8+ T cell Ab	GCST90001698	lymphocyte count	IPF
CD45RA+ CD28- CD8+ T cell Ab	GCST90001698	lymphocyte count	IPF
CD45RA+ CD28- CD8+ T cell Ab	GCST90001698	lymphocyte count	IPF
CD45RA+ CD28- CD8+ T cell Ab	GCST90001698	lymphocyte count	IPF
CD45RA+ CD28- CD8+ T cell Ab	GCST90001698	lymphocyte count	IPF
CD45RA+ CD28- CD8+ T cell Ab	GCST90001698	lymphocyte count	IPF
CD45RA+ CD28- CD8+ T cell Ab	GCST90001698	lymphocyte count	IPF
CD45RA+ CD28- CD8+ T cell Ab	GCST90001698	lymphocyte count	IPF
CD45RA+ CD28- CD8+ T cell Ab	GCST90001698	lymphocyte count	IPF
CD45RA+ CD28- CD8+ T cell Ab	GCST90001698	lymphocyte count	IPF
CD45RA+ CD28- CD8+ T cell Ab	GCST90001698	lymphocyte count	IPF
CD45RA+ CD28- CD8+ T cell Ab	GCST90001698	lymphocyte count	IPF
CD45RA+ CD28- CD8+ T cell Ab	GCST90001698	lymphocyte count	IPF
CD45RA+ CD28- CD8+ T cell Ab	GCST90001698	lymphocyte count	IPF
CD45RA+ CD28- CD8+ T cell Ab	GCST90001698	lymphocyte count	IPF
CD45RA+ CD28- CD8+ T cell Ab	GCST90001698	lymphocyte count	IPF
CD45RA+ CD28- CD8+ T cell Ab	GCST90001698	lymphocyte count	IPF
CD45RA+ CD28- CD8+ T cell Ab	GCST90001698	lymphocyte count	IPF
CD45RA+ CD28- CD8+ T cell Ab	GCST90001698	lymphocyte count	IPF
CD45RA+ CD28- CD8+ T cell Ab	GCST90001698	lymphocyte count	IPF
CD45RA+ CD28- CD8+ T cell Ab	GCST90001698	lymphocyte count	IPF
CD45RA+ CD28- CD8+ T cell Ab	GCST90001698	lymphocyte count	IPF
CD45RA+ CD28- CD8+ T cell Ab	GCST90001698	lymphocyte count	IPF
CD45RA+ CD28- CD8+ T cell Ab	GCST90001698	lymphocyte count	IPF
CD45RA+ CD28- CD8+ T cell Ab	GCST90001698	lymphocyte count	IPF
CD45RA+ CD28- CD8+ T cell Ab	GCST90001698	lymphocyte count	IPF
CD45RA+ CD28- CD8+ T cell Ab	GCST90001698	lymphocyte count	IPF
CD45RA+ CD28- CD8+ T cell Ab	GCST90001698	lymphocyte count	IPF
CD45RA+ CD28- CD8+ T cell Ab	GCST90001698	lymphocyte count	IPF
CD45RA+ CD28- CD8+ T cell Ab	GCST90001698	lymphocyte count	IPF
CD45RA+ CD28- CD8+ T cell Ab	GCST90001698	lymphocyte count	IPF
CD45RA+ CD28- CD8+ T cell Ab	GCST90001698	lymphocyte count	IPF
CD45RA+ CD28- CD8+ T cell Ab	GCST90001698	lymphocyte count	IPF
CD45RA+ CD28- CD8+ T cell Ab	GCST90001698	lymphocyte count	IPF
CD45RA+ CD28- CD8+ T cell Ab	GCST90001698	lymphocyte count	IPF
CD45RA+ CD28- CD8+ T cell Ab	GCST90001698	lymphocyte count	IPF
CD45RA+ CD28- CD8+ T cell Ab	GCST90001698	lymphocyte count	IPF
CD45RA+ CD28- CD8+ T cell Ab	GCST90001698	lymphocyte count	IPF
CD45RA+ CD28- CD8+ T cell Ab	GCST90001698	lymphocyte count	IPF
CD45RA+ CD28- CD8+ T cell Ab	GCST90001698	lymphocyte count	IPF
CD45RA+ CD28- CD8+ T cell Ab	GCST90001698	lymphocyte count	IPF
CD45RA+ CD28- CD8+ T cell Ab	GCST90001698	lymphocyte count	IPF
CD45RA+ CD28- CD8+ T cell Ab	GCST90001698	lymphocyte count	IPF
CD45RA+ CD28- CD8+ T cell Ab	GCST90001698	lymphocyte count	IPF
CD45RA+ CD28- CD8+ T cell Ab	GCST90001698	lymphocyte count	IPF
CD45RA+ CD28- CD8+ T cell Ab	GCST90001698	lymphocyte count	IPF
CD45RA+ CD28- CD8+ T cell Ab	GCST90001698	lymphocyte count	IPF
CD45RA+ CD28- CD8+ T cell Ab	GCST90001698	lymphocyte count	IPF
CD45RA+ CD28- CD8+ T cell Ab	GCST90001698	lymphocyte count	IPF
CD45RA+ CD28- CD8+ T cell Ab	GCST90001698	lymphocyte count	IPF
CD45RA+ CD28- CD8+ T cell Ab	GCST90001698	lymphocyte count	IPF
CD45RA+ CD28- CD8+ T cell Ab	GCST90001698	lymphocyte count	IPF
CD45RA+ CD28- CD8+ T cell Ab	GCST90001698	lymphocyte count	IPF
CD45RA+ CD28- CD8+ T cell Ab	GCST90001698	lymphocyte count	IPF
CD45RA+ CD28- CD8+ T cell Ab	GCST90001698	lymphocyte count	IPF
CD45RA+ CD28- CD8+ T cell Ab	GCST90001698	lymphocyte count	IPF

CD45RA+ CD28- CD8+ T cell Ab	GCST90001698	lymphocyte count	IPF
CD45RA+ CD28- CD8+ T cell Ab	GCST90001698	lymphocyte count	IPF
CD45RA+ CD28- CD8+ T cell Ab	GCST90001698	lymphocyte count	IPF
CD45RA+ CD28- CD8+ T cell Ab	GCST90001698	lymphocyte count	IPF
CD45RA+ CD28- CD8+ T cell Ab	GCST90001698	lymphocyte count	IPF
CD45RA+ CD28- CD8+ T cell Ab	GCST90001698	lymphocyte count	IPF
CD45RA+ CD28- CD8+ T cell Ab	GCST90001698	lymphocyte count	IPF
CD45RA+ CD28- CD8+ T cell Ab	GCST90001698	lymphocyte count	IPF
CD45RA+ CD28- CD8+ T cell Ab	GCST90001698	lymphocyte count	IPF
CD45RA+ CD28- CD8+ T cell Ab	GCST90001698	lymphocyte count	IPF
CD45RA+ CD28- CD8+ T cell Ab	GCST90001698	lymphocyte count	IPF
CD45RA+ CD28- CD8+ T cell Ab	GCST90001698	lymphocyte count	IPF
CD45RA+ CD28- CD8+ T cell Ab	GCST90001698	lymphocyte count	IPF
CD45RA+ CD28- CD8+ T cell Ab	GCST90001698	lymphocyte count	IPF
CD45RA+ CD28- CD8+ T cell Ab	GCST90001698	lymphocyte count	IPF
CD45RA+ CD28- CD8+ T cell Ab	GCST90001698	lymphocyte count	IPF
CD45RA+ CD28- CD8+ T cell Ab	GCST90001698	lymphocyte count	IPF
CD45RA+ CD28- CD8+ T cell Ab	GCST90001698	lymphocyte count	IPF
CD45RA+ CD28- CD8+ T cell Ab	GCST90001698	lymphocyte count	IPF
CD45RA+ CD28- CD8+ T cell Ab	GCST90001698	lymphocyte count	IPF
CD45RA+ CD28- CD8+ T cell Ab	GCST90001698	lymphocyte count	IPF
CD45RA+ CD28- CD8+ T cell Ab	GCST90001698	lymphocyte count	IPF
CD45RA+ CD28- CD8+ T cell Ab	GCST90001698	lymphocyte count	IPF
CD45RA+ CD28- CD8+ T cell Ab	GCST90001698	lymphocyte count	IPF
CD45RA+ CD28- CD8+ T cell Ab	GCST90001698	lymphocyte count	IPF
CD45RA+ CD28- CD8+ T cell Ab	GCST90001698	lymphocyte count	IPF
CD45RA+ CD28- CD8+ T cell Ab	GCST90001698	lymphocyte count	IPF
CD45RA+ CD28- CD8+ T cell Ab	GCST90001698	lymphocyte count	IPF
CD45RA+ CD28- CD8+ T cell Ab	GCST90001698	lymphocyte count	IPF
CD45RA+ CD28- CD8+ T cell Ab	GCST90001698	lymphocyte count	IPF
CD45RA+ CD28- CD8+ T cell Ab	GCST90001698	lymphocyte count	IPF
CD45RA+ CD28- CD8+ T cell Ab	GCST90001698	lymphocyte count	IPF
CD45RA+ CD28- CD8+ T cell Ab	GCST90001698	lymphocyte count	IPF
CD45RA+ CD28- CD8+ T cell Ab	GCST90001698	lymphocyte count	IPF
CD45RA+ CD28- CD8+ T cell Ab	GCST90001698	lymphocyte count	IPF
CD45RA+ CD28- CD8+ T cell Ab	GCST90001698	lymphocyte count	IPF
CD45RA+ CD28- CD8+ T cell Ab	GCST90001698	lymphocyte count	IPF
CD45RA+ CD28- CD8+ T cell Ab	GCST90001698	lymphocyte count	IPF
CD45RA+ CD28- CD8+ T cell Ab	GCST90001698	lymphocyte count	IPF
CD45RA+ CD28- CD8+ T cell Ab	GCST90001698	lymphocyte count	IPF
CD45RA+ CD28- CD8+ T cell Ab	GCST90001698	lymphocyte count	IPF
CD45RA+ CD28- CD8+ T cell Ab	GCST90001698	lymphocyte count	IPF
CD45RA+ CD28- CD8+ T cell Ab	GCST90001698	lymphocyte count	IPF
CD45RA+ CD28- CD8+ T cell Ab	GCST90001698	lymphocyte count	IPF
CD45RA+ CD28- CD8+ T cell Ab	GCST90001698	lymphocyte count	IPF
CD45RA+ CD28- CD8+ T cell Ab	GCST90001698	lymphocyte count	IPF
CD45RA+ CD28- CD8+ T cell Ab	GCST90001698	lymphocyte count	IPF
CD45RA+ CD28- CD8+ T cell Ab	GCST90001698	lymphocyte count	IPF
CD45RA+ CD28- CD8+ T cell Ab	GCST90001698	lymphocyte count	IPF
CD45RA+ CD28- CD8+ T cell Ab	GCST90001698	lymphocyte count	IPF
CD45RA+ CD28- CD8+ T cell Ab	GCST90001698	lymphocyte count	IPF
CD45RA+ CD28- CD8+ T cell Ab	GCST90001698	lymphocyte count	IPF
CD45RA+ CD28- CD8+ T cell Ab	GCST90001698	lymphocyte count	IPF
CD45RA+ CD28- CD8+ T cell Ab	GCST90001698	lymphocyte count	IPF
CD45RA+ CD28- CD8+ T cell Ab	GCST90001698	lymphocyte count	IPF
CD45RA+ CD28- CD8+ T cell Ab	GCST90001698	lymphocyte count	IPF
CD45RA+ CD28- CD8+ T cell Ab	GCST90001698	lymphocyte count	IPF
CD45RA+ CD28- CD8+ T cell Ab	GCST90001698	lymphocyte count	IPF

CD45RA+ CD28- CD8+ T cell Ab	GCST90001698	lymphocyte count	IPF
CD45RA+ CD28- CD8+ T cell Ab	GCST90001698	lymphocyte count	IPF
CD45RA+ CD28- CD8+ T cell Ab	GCST90001698	lymphocyte count	IPF
CD45RA+ CD28- CD8+ T cell Ab	GCST90001698	lymphocyte count	IPF
CD45RA+ CD28- CD8+ T cell Ab	GCST90001698	lymphocyte count	IPF
CD45RA+ CD28- CD8+ T cell Ab	GCST90001698	lymphocyte count	IPF
CD45RA+ CD28- CD8+ T cell Ab	GCST90001698	lymphocyte count	IPF
CD45RA+ CD28- CD8+ T cell Ab	GCST90001698	lymphocyte count	IPF
CD45RA+ CD28- CD8+ T cell Ab	GCST90001698	lymphocyte count	IPF
CD45RA+ CD28- CD8+ T cell Ab	GCST90001698	lymphocyte count	IPF
CD45RA+ CD28- CD8+ T cell Ab	GCST90001698	lymphocyte count	IPF
CD45RA+ CD28- CD8+ T cell Ab	GCST90001698	lymphocyte count	IPF
CD45RA+ CD28- CD8+ T cell Ab	GCST90001698	lymphocyte count	IPF
CD45RA+ CD28- CD8+ T cell Ab	GCST90001698	lymphocyte count	IPF
CD45RA+ CD28- CD8+ T cell Ab	GCST90001698	lymphocyte count	IPF
CD45RA+ CD28- CD8+ T cell Ab	GCST90001698	lymphocyte count	IPF
CD45RA+ CD28- CD8+ T cell Ab	GCST90001698	lymphocyte count	IPF
CD45RA+ CD28- CD8+ T cell Ab	GCST90001698	lymphocyte count	IPF
CD45RA+ CD28- CD8+ T cell Ab	GCST90001698	lymphocyte count	IPF
CD45RA+ CD28- CD8+ T cell Ab	GCST90001698	lymphocyte count	IPF
CD45RA+ CD28- CD8+ T cell Ab	GCST90001698	lymphocyte count	IPF
CD45RA+ CD28- CD8+ T cell Ab	GCST90001698	lymphocyte count	IPF
CD45RA+ CD28- CD8+ T cell Ab	GCST90001698	lymphocyte count	IPF
CD45RA+ CD28- CD8+ T cell Ab	GCST90001698	lymphocyte count	IPF
CD45RA+ CD28- CD8+ T cell Ab	GCST90001698	lymphocyte count	IPF
CD45RA+ CD28- CD8+ T cell Ab	GCST90001698	lymphocyte count	IPF
CD45RA+ CD28- CD8+ T cell Ab	GCST90001698	lymphocyte count	IPF
CD45RA+ CD28- CD8+ T cell Ab	GCST90001698	lymphocyte count	IPF
CD45RA+ CD28- CD8+ T cell Ab	GCST90001698	lymphocyte count	IPF
CD45RA+ CD28- CD8+ T cell Ab	GCST90001698	lymphocyte count	IPF
CD45RA+ CD28- CD8+ T cell Ab	GCST90001698	lymphocyte count	IPF
CD45RA+ CD28- CD8+ T cell Ab	GCST90001698	lymphocyte count	IPF
CD45RA+ CD28- CD8+ T cell Ab	GCST90001698	lymphocyte count	IPF
CD45RA+ CD28- CD8+ T cell Ab	GCST90001698	lymphocyte count	IPF
CD45RA+ CD28- CD8+ T cell Ab	GCST90001698	lymphocyte count	IPF
CD45RA+ CD28- CD8+ T cell Ab	GCST90001698	lymphocyte count	IPF
CD45RA+ CD28- CD8+ T cell Ab	GCST90001698	lymphocyte count	IPF
CD45RA+ CD28- CD8+ T cell Ab	GCST90001698	lymphocyte count	IPF
CD45RA+ CD28- CD8+ T cell Ab	GCST90001698	lymphocyte count	IPF
CD45RA+ CD28- CD8+ T cell Ab	GCST90001698	lymphocyte count	IPF
CD45RA+ CD28- CD8+ T cell Ab	GCST90001698	lymphocyte count	IPF
CD45RA+ CD28- CD8+ T cell Ab	GCST90001698	lymphocyte count	IPF
CD45RA+ CD28- CD8+ T cell Ab	GCST90001698	lymphocyte count	IPF
CD45RA+ CD28- CD8+ T cell Ab	GCST90001698	lymphocyte count	IPF
CD45RA+ CD28- CD8+ T cell Ab	GCST90001698	lymphocyte count	IPF
CD45RA+ CD28- CD8+ T cell Ab	GCST90001698	lymphocyte count	IPF
CD45RA+ CD28- CD8+ T cell Ab	GCST90001698	lymphocyte count	IPF
CD45RA+ CD28- CD8+ T cell Ab	GCST90001698	lymphocyte count	IPF
CD45RA+ CD28- CD8+ T cell Ab	GCST90001698	lymphocyte count	IPF
CD45RA+ CD28- CD8+ T cell Ab	GCST90001698	lymphocyte count	IPF
CD45RA+ CD28- CD8+ T cell Ab	GCST90001698	lymphocyte count	IPF
CD45RA+ CD28- CD8+ T cell Ab	GCST90001698	lymphocyte count	IPF
CD45RA+ CD28- CD8+ T cell Ab	GCST90001698	lymphocyte count	IPF
CD45RA+ CD28- CD8+ T cell Ab	GCST90001698	lymphocyte count	IPF
CD45RA+ CD28- CD8+ T cell Ab	GCST90001698	lymphocyte count	IPF
CD45RA+ CD28- CD8+ T cell Ab	GCST90001698	lymphocyte count	IPF
CD45RA+ CD28- CD8+ T cell Ab	GCST90001698	lymphocyte count	IPF
CD45RA+ CD28- CD8+ T cell Ab	GCST90001698	lymphocyte count	IPF

CD45RA+ CD28- CD8+ T cell Ab	GCST90001698	lymphocyte count	IPF
CD45RA+ CD28- CD8+ T cell Ab	GCST90001698	lymphocyte count	IPF
CD45RA+ CD28- CD8+ T cell Ab	GCST90001698	lymphocyte count	IPF
CD45RA+ CD28- CD8+ T cell Ab	GCST90001698	lymphocyte count	IPF
CD45RA+ CD28- CD8+ T cell Ab	GCST90001698	lymphocyte count	IPF
CD45RA+ CD28- CD8+ T cell Ab	GCST90001698	lymphocyte count	IPF
CD45RA+ CD28- CD8+ T cell Ab	GCST90001698	lymphocyte count	IPF
CD45RA+ CD28- CD8+ T cell Ab	GCST90001698	lymphocyte count	IPF
CD45RA+ CD28- CD8+ T cell Ab	GCST90001698	lymphocyte count	IPF
CD45RA+ CD28- CD8+ T cell Ab	GCST90001698	lymphocyte count	IPF
CD45RA+ CD28- CD8+ T cell Ab	GCST90001698	lymphocyte count	IPF
CD45RA+ CD28- CD8+ T cell Ab	GCST90001698	lymphocyte count	IPF
CD45RA+ CD28- CD8+ T cell Ab	GCST90001698	lymphocyte count	IPF
CD45RA+ CD28- CD8+ T cell Ab	GCST90001698	lymphocyte count	IPF
CD45RA+ CD28- CD8+ T cell Ab	GCST90001698	lymphocyte count	IPF
CD45RA+ CD28- CD8+ T cell Ab	GCST90001698	lymphocyte count	IPF
CD45RA+ CD28- CD8+ T cell Ab	GCST90001698	lymphocyte count	IPF
CD45RA+ CD28- CD8+ T cell Ab	GCST90001698	lymphocyte count	IPF
CD45RA+ CD28- CD8+ T cell Ab	GCST90001698	lymphocyte count	IPF
CD45RA+ CD28- CD8+ T cell Ab	GCST90001698	lymphocyte count	IPF
CD45RA+ CD28- CD8+ T cell Ab	GCST90001698	lymphocyte count	IPF
CD45RA+ CD28- CD8+ T cell Ab	GCST90001698	lymphocyte count	IPF
CD45RA+ CD28- CD8+ T cell Ab	GCST90001698	lymphocyte count	IPF
CD45RA+ CD28- CD8+ T cell Ab	GCST90001698	lymphocyte count	IPF
CD45RA+ CD28- CD8+ T cell Ab	GCST90001698	lymphocyte count	IPF
Naive CD8+ T cell %T cell	GCST90001553	lymphocyte count	IPF
Naive CD8+ T cell %T cell	GCST90001553	lymphocyte count	IPF
Naive CD8+ T cell %T cell	GCST90001553	lymphocyte count	IPF
CD8 on CD28+ CD45RA+ CD8+	GCST90002119	blood protein measurement	IPF
CD8 on CD28+ CD45RA+ CD8+	GCST90002119	blood protein measurement	IPF
CD8 on CD28+ CD45RA+ CD8+	GCST90002119	blood protein measurement	IPF
CD8 on CD28+ CD45RA+ CD8+	GCST90002119	blood protein measurement	IPF
CD8 on CD28+ CD45RA+ CD8+	GCST90002119	blood protein measurement	IPF
CD8 on CD28+ CD45RA+ CD8+	GCST90002119	blood protein measurement	IPF
CD8 on CD28+ CD45RA+ CD8+	GCST90002119	blood protein measurement	IPF
CD8 on CD28+ CD45RA+ CD8+	GCST90002119	blood protein measurement	IPF
IgD on transitional B cell	GCST90001828	blood protein measurement	IPF
IgD on transitional B cell	GCST90001828	blood protein measurement	IPF
IgD on transitional B cell	GCST90001828	blood protein measurement	IPF
IgD on transitional B cell	GCST90001828	blood protein measurement	IPF
SSC-A on Natural Killer T	GCST90002084	blood protein measurement	IPF
SSC-A on Natural Killer T	GCST90002084	blood protein measurement	IPF
CD19 on CD20- CD38- B cell	GCST90001722	blood protein measurement	IPF
CD20 on switched memory B cell	GCST90001761	blood protein measurement	IPF
CD20 on switched memory B cell	GCST90001761	blood protein measurement	IPF
CD20 on switched memory B cell	GCST90001761	blood protein measurement	IPF
CD20 on switched memory B cell	GCST90001761	blood protein measurement	IPF
CD20 on switched memory B cell	GCST90001761	blood protein measurement	IPF
CD20 on switched memory B cell	GCST90001761	blood protein measurement	IPF
CD20 on switched memory B cell	GCST90001761	blood protein measurement	IPF
CD45RA+ CD28- CD8+ T cell %	GCST90001700	lymphocyte count	IPF
CD45RA+ CD28- CD8+ T cell %	GCST90001700	lymphocyte count	IPF
CD45RA+ CD28- CD8+ T cell %	GCST90001700	lymphocyte count	IPF
CD45RA+ CD28- CD8+ T cell %	GCST90001700	lymphocyte count	IPF
CD45RA+ CD28- CD8+ T cell %	GCST90001700	lymphocyte count	IPF
CD45RA+ CD28- CD8+ T cell %	GCST90001700	lymphocyte count	IPF
CD45RA+ CD28- CD8+ T cell %	GCST90001700	lymphocyte count	IPF
CD45RA+ CD28- CD8+ T cell %	GCST90001700	lymphocyte count	IPF

CD45RA+ CD28- CD8+ T cell %T	GCST90001700	lymphocyte count	IPF
CD45RA+ CD28- CD8+ T cell %T	GCST90001700	lymphocyte count	IPF
CD45RA+ CD28- CD8+ T cell %T	GCST90001700	lymphocyte count	IPF
CD45RA+ CD28- CD8+ T cell %T	GCST90001700	lymphocyte count	IPF
CD45RA+ CD28- CD8+ T cell %T	GCST90001700	lymphocyte count	IPF
CD45RA+ CD28- CD8+ T cell %T	GCST90001700	lymphocyte count	IPF
CD45RA+ CD28- CD8+ T cell %T	GCST90001700	lymphocyte count	IPF
CD45RA+ CD28- CD8+ T cell %T	GCST90001700	lymphocyte count	IPF
CD45RA+ CD28- CD8+ T cell %T	GCST90001700	lymphocyte count	IPF
CD45RA+ CD28- CD8+ T cell %T	GCST90001700	lymphocyte count	IPF
CD45RA+ CD28- CD8+ T cell %T	GCST90001700	lymphocyte count	IPF
CD45RA+ CD28- CD8+ T cell %T	GCST90001700	lymphocyte count	IPF
CD45RA+ CD28- CD8+ T cell %T	GCST90001700	lymphocyte count	IPF
CD45RA+ CD28- CD8+ T cell %T	GCST90001700	lymphocyte count	IPF
CD45RA+ CD28- CD8+ T cell %T	GCST90001700	lymphocyte count	IPF
CD45RA+ CD28- CD8+ T cell %T	GCST90001700	lymphocyte count	IPF
CD45RA+ CD28- CD8+ T cell %T	GCST90001700	lymphocyte count	IPF
CD45RA+ CD28- CD8+ T cell %T	GCST90001700	lymphocyte count	IPF
CD45RA+ CD28- CD8+ T cell %T	GCST90001700	lymphocyte count	IPF
CD45RA+ CD28- CD8+ T cell %T	GCST90001700	lymphocyte count	IPF
CD45RA+ CD28- CD8+ T cell %T	GCST90001700	lymphocyte count	IPF
CD45RA+ CD28- CD8+ T cell %T	GCST90001700	lymphocyte count	IPF
CD45RA+ CD28- CD8+ T cell %T	GCST90001700	lymphocyte count	IPF
CD45RA+ CD28- CD8+ T cell %T	GCST90001700	lymphocyte count	IPF
CD45RA+ CD28- CD8+ T cell %T	GCST90001700	lymphocyte count	IPF
CD45RA+ CD28- CD8+ T cell %T	GCST90001700	lymphocyte count	IPF
CD45RA+ CD28- CD8+ T cell %T	GCST90001700	lymphocyte count	IPF
CD45RA+ CD28- CD8+ T cell %T	GCST90001700	lymphocyte count	IPF
CD45RA+ CD28- CD8+ T cell %T	GCST90001700	lymphocyte count	IPF
CD45RA+ CD28- CD8+ T cell %T	GCST90001700	lymphocyte count	IPF
CD45RA+ CD28- CD8+ T cell %T	GCST90001700	lymphocyte count	IPF
CD45RA+ CD28- CD8+ T cell %T	GCST90001700	lymphocyte count	IPF
CD45RA+ CD28- CD8+ T cell %T	GCST90001700	lymphocyte count	IPF
CD45RA+ CD28- CD8+ T cell %T	GCST90001700	lymphocyte count	IPF
CD45RA+ CD28- CD8+ T cell %T	GCST90001700	lymphocyte count	IPF
CD45RA+ CD28- CD8+ T cell %T	GCST90001700	lymphocyte count	IPF
CD45RA+ CD28- CD8+ T cell %T	GCST90001700	lymphocyte count	IPF
CD45RA+ CD28- CD8+ T cell %T	GCST90001700	lymphocyte count	IPF
CD45RA+ CD28- CD8+ T cell %T	GCST90001700	lymphocyte count	IPF
CD45RA+ CD28- CD8+ T cell %T	GCST90001700	lymphocyte count	IPF
CD45RA+ CD28- CD8+ T cell %T	GCST90001700	lymphocyte count	IPF
CD45RA+ CD28- CD8+ T cell %T	GCST90001700	lymphocyte count	IPF
CD45RA+ CD28- CD8+ T cell %T	GCST90001700	lymphocyte count	IPF
CD45RA+ CD28- CD8+ T cell %T	GCST90001700	lymphocyte count	IPF
CD45RA+ CD28- CD8+ T cell %T	GCST90001700	lymphocyte count	IPF
CD45RA+ CD28- CD8+ T cell %T	GCST90001700	lymphocyte count	IPF
CD45RA+ CD28- CD8+ T cell %T	GCST90001700	lymphocyte count	IPF
CD45RA+ CD28- CD8+ T cell %T	GCST90001700	lymphocyte count	IPF
CD45RA+ CD28- CD8+ T cell %T	GCST90001700	lymphocyte count	IPF
CD3- lymphocyte %lymphocyte	GCST90001640	leukocyte count	IPF
CD3- lymphocyte %lymphocyte	GCST90001640	leukocyte count	IPF
Natural Killer %lymphocyte	GCST90001647	leukocyte count	IPF
Natural Killer %lymphocyte	GCST90001647	leukocyte count	IPF
Natural Killer %lymphocyte	GCST90001647	leukocyte count	IPF
T cell %lymphocyte	GCST90001604	leukocyte count	IPF
T cell %lymphocyte	GCST90001604	leukocyte count	IPF
Naive CD8+ T cell %CD8+ T cell	GCST90001552	lymphocyte count	IPF
CD4 on CD4+ T cell	GCST90002022	blood protein measurement	IPF

CD4 on CD4+ T cell	GCST90002022	blood protein measurement	IPF
CD39+ CD4+ T cell Absolute Cou	GCST90001660	lymphocyte count	IPF
CD39+ CD4+ T cell Absolute Cou	GCST90001660	lymphocyte count	IPF
CD39+ CD4+ T cell Absolute Cou	GCST90001660	lymphocyte count	IPF
CD39+ CD4+ T cell Absolute Cou	GCST90001660	lymphocyte count	IPF
CD39+ CD4+ T cell Absolute Cou	GCST90001660	lymphocyte count	IPF
CD39+ CD4+ T cell Absolute Cou	GCST90001660	lymphocyte count	IPF
CD39+ secreting CD4 regulatory ꟷ	GCST90001497	lymphocyte count	IPF
CD39+ secreting CD4 regulatory ꟷ	GCST90001497	lymphocyte count	IPF
CD39+ secreting CD4 regulatory ꟷ	GCST90001497	lymphocyte count	IPF
CD39+ secreting CD4 regulatory ꟷ	GCST90001497	lymphocyte count	IPF
CD39+ secreting CD4 regulatory ꟷ	GCST90001497	lymphocyte count	IPF
CD39+ secreting CD4 regulatory ꟷ	GCST90001497	lymphocyte count	IPF
CD39+ activated CD4 regulatory ꟷ	GCST90001489	lymphocyte count	IPF
CD39+ activated CD4 regulatory ꟷ	GCST90001489	lymphocyte count	IPF
CD39+ activated CD4 regulatory ꟷ	GCST90001489	lymphocyte count	IPF
CD39+ activated CD4 regulatory ꟷ	GCST90001489	lymphocyte count	IPF
CD39+ activated CD4 regulatory ꟷ	GCST90001489	lymphocyte count	IPF
CD39+ activated CD4 regulatory ꟷ	GCST90001491	lymphocyte count	IPF
CD39+ activated CD4 regulatory ꟷ	GCST90001491	lymphocyte count	IPF
CD39+ activated CD4 regulatory ꟷ	GCST90001491	lymphocyte count	IPF
CD39+ activated CD4 regulatory ꟷ	GCST90001491	lymphocyte count	IPF
CD39+ activated CD4 regulatory ꟷ	GCST90001491	lymphocyte count	IPF
CD39+ activated CD4 regulatory ꟷ	GCST90001491	lymphocyte count	IPF
CD4 on CD39+ CD4+ T cell	GCST90002061	blood protein measurement	IPF
CD4 on CD39+ CD4+ T cell	GCST90002061	blood protein measurement	IPF
CD4 on CD39+ CD4+ T cell	GCST90002061	blood protein measurement	IPF
CD4 on CD39+ CD4+ T cell	GCST90002061	blood protein measurement	IPF
CD4 on CD39+ CD4+ T cell	GCST90002061	blood protein measurement	IPF
BAFF-R on IgD+ CD38- naive B c	GCST90001706	blood protein measurement	IPF
BAFF-R on IgD+ CD38- naive B c	GCST90001706	blood protein measurement	IPF
BAFF-R on IgD+ CD38- naive B c	GCST90001706	blood protein measurement	IPF
BAFF-R on IgD+ CD38- naive B c	GCST90001706	blood protein measurement	IPF
BAFF-R on IgD+ CD38- naive B c	GCST90001706	blood protein measurement	IPF
BAFF-R on IgD+ CD38- naive B c	GCST90001706	blood protein measurement	IPF
BAFF-R on IgD+ CD38- naive B c	GCST90001706	blood protein measurement	IPF
BAFF-R on IgD+ CD38- B cell	GCST90001705	blood protein measurement	IPF
BAFF-R on IgD+ CD38- B cell	GCST90001705	blood protein measurement	IPF
BAFF-R on IgD+ CD38- B cell	GCST90001705	blood protein measurement	IPF
BAFF-R on IgD+ CD38- B cell	GCST90001705	blood protein measurement	IPF
BAFF-R on IgD+ CD38- B cell	GCST90001705	blood protein measurement	IPF
BAFF-R on IgD+ CD38- B cell	GCST90001705	blood protein measurement	IPF
BAFF-R on IgD+ CD38- B cell	GCST90001705	blood protein measurement	IPF
BAFF-R on IgD+ CD38dim B cell	GCST90001709	blood protein measurement	IPF
BAFF-R on IgD+ CD38dim B cell	GCST90001709	blood protein measurement	IPF
BAFF-R on IgD+ CD38dim B cell	GCST90001709	blood protein measurement	IPF
BAFF-R on IgD+ CD38dim B cell	GCST90001709	blood protein measurement	IPF
BAFF-R on IgD+ CD38dim B cell	GCST90001709	blood protein measurement	IPF
BAFF-R on IgD+ CD38dim B cell	GCST90001709	blood protein measurement	IPF
BAFF-R on IgD+ CD38dim B cell	GCST90001709	blood protein measurement	IPF
BAFF-R on IgD+ CD38dim B cell	GCST90001709	blood protein measurement	IPF
BAFF-R on IgD+ B cell	GCST90001719	blood protein measurement	IPF
BAFF-R on IgD+ B cell	GCST90001719	blood protein measurement	IPF
BAFF-R on IgD+ B cell	GCST90001719	blood protein measurement	IPF
BAFF-R on IgD+ B cell	GCST90001719	blood protein measurement	IPF
BAFF-R on IgD+ B cell	GCST90001719	blood protein measurement	IPF
BAFF-R on IgD+ B cell	GCST90001719	blood protein measurement	IPF
BAFF-R on IgD+ B cell	GCST90001719	blood protein measurement	IPF

BAFF-R on IgD+ B cell	GCST90001719	blood protein measurement	IPF
BAFF-R on IgD+ CD24- B cell	GCST90001704	blood protein measurement	IPF
BAFF-R on IgD+ CD24- B cell	GCST90001704	blood protein measurement	IPF
BAFF-R on IgD+ CD24- B cell	GCST90001704	blood protein measurement	IPF
BAFF-R on IgD+ CD24- B cell	GCST90001704	blood protein measurement	IPF
BAFF-R on IgD+ CD24- B cell	GCST90001704	blood protein measurement	IPF
BAFF-R on IgD+ CD24- B cell	GCST90001704	blood protein measurement	IPF
BAFF-R on IgD+ CD24- B cell	GCST90001704	blood protein measurement	IPF
BAFF-R on IgD+ CD24- B cell	GCST90001704	blood protein measurement	IPF
BAFF-R on IgD+ CD24- B cell	GCST90001704	blood protein measurement	IPF
BAFF-R on naive-mature B cell	GCST90001716	blood protein measurement	IPF
BAFF-R on naive-mature B cell	GCST90001716	blood protein measurement	IPF
BAFF-R on naive-mature B cell	GCST90001716	blood protein measurement	IPF
BAFF-R on naive-mature B cell	GCST90001716	blood protein measurement	IPF
BAFF-R on naive-mature B cell	GCST90001716	blood protein measurement	IPF
BAFF-R on naive-mature B cell	GCST90001716	blood protein measurement	IPF
BAFF-R on naive-mature B cell	GCST90001716	blood protein measurement	IPF
BAFF-R on naive-mature B cell	GCST90001716	blood protein measurement	IPF
BAFF-R on B cell	GCST90001829	blood protein measurement	IPF
BAFF-R on B cell	GCST90001829	blood protein measurement	IPF
BAFF-R on B cell	GCST90001829	blood protein measurement	IPF
BAFF-R on B cell	GCST90001829	blood protein measurement	IPF
BAFF-R on B cell	GCST90001829	blood protein measurement	IPF
BAFF-R on B cell	GCST90001829	blood protein measurement	IPF
BAFF-R on B cell	GCST90001829	blood protein measurement	IPF
BAFF-R on B cell	GCST90001829	blood protein measurement	IPF
BAFF-R on B cell	GCST90001829	blood protein measurement	IPF
BAFF-R on transitional B cell	GCST90001720	blood protein measurement	IPF
BAFF-R on transitional B cell	GCST90001720	blood protein measurement	IPF
BAFF-R on transitional B cell	GCST90001720	blood protein measurement	IPF
BAFF-R on transitional B cell	GCST90001720	blood protein measurement	IPF
BAFF-R on transitional B cell	GCST90001720	blood protein measurement	IPF
BAFF-R on transitional B cell	GCST90001720	blood protein measurement	IPF
BAFF-R on transitional B cell	GCST90001720	blood protein measurement	IPF
BAFF-R on transitional B cell	GCST90001720	blood protein measurement	IPF
BAFF-R on IgD+ CD38+ B cell	GCST90001708	blood protein measurement	IPF
BAFF-R on IgD+ CD38+ B cell	GCST90001708	blood protein measurement	IPF
BAFF-R on IgD+ CD38+ B cell	GCST90001708	blood protein measurement	IPF
BAFF-R on IgD+ CD38+ B cell	GCST90001708	blood protein measurement	IPF
BAFF-R on IgD+ CD38+ B cell	GCST90001708	blood protein measurement	IPF
BAFF-R on IgD+ CD38+ B cell	GCST90001708	blood protein measurement	IPF
BAFF-R on IgD+ CD38+ B cell	GCST90001708	blood protein measurement	IPF
BAFF-R on IgD+ CD38+ B cell	GCST90001708	blood protein measurement	IPF
BAFF-R on IgD+ CD38+ B cell	GCST90001708	blood protein measurement	IPF
HLA DR+ T cell%T cell	GCST90001619	leukocyte count	IPF
HLA DR+ T cell%T cell	GCST90001619	leukocyte count	IPF
HLA DR+ T cell%T cell	GCST90001619	leukocyte count	IPF
HLA DR+ T cell%T cell	GCST90001619	leukocyte count	IPF
CD39+ secreting CD4 regulatory T	GCST90001496	lymphocyte count	IPF
CD39+ secreting CD4 regulatory T	GCST90001496	lymphocyte count	IPF
CD39+ secreting CD4 regulatory T	GCST90001496	lymphocyte count	IPF
CD39+ secreting CD4 regulatory T	GCST90001496	lymphocyte count	IPF
CD39+ secreting CD4 regulatory T	GCST90001496	lymphocyte count	IPF
CD39+ secreting CD4 regulatory T	GCST90001496	lymphocyte count	IPF
CD39+ secreting CD4 regulatory T	GCST90001495	lymphocyte count	IPF
CD39+ secreting CD4 regulatory T	GCST90001495	lymphocyte count	IPF
CD39+ secreting CD4 regulatory T	GCST90001495	lymphocyte count	IPF
CD39+ secreting CD4 regulatory T	GCST90001495	lymphocyte count	IPF

CD39+ secreting CD4 regulatory T	GCST90001495	lymphocyte count	IPF
CD39+ secreting CD4 regulatory T	GCST90001495	lymphocyte count	IPF
CD39+ secreting CD4 regulatory T	GCST90001495	lymphocyte count	IPF
CD39+ CD4+ T cell %T cell	GCST90001658	lymphocyte count	IPF
CD39+ CD4+ T cell %T cell	GCST90001658	lymphocyte count	IPF
CD39+ CD4+ T cell %T cell	GCST90001658	lymphocyte count	IPF
CD39+ CD4+ T cell %T cell	GCST90001658	lymphocyte count	IPF
CD39+ CD4+ T cell %T cell	GCST90001658	lymphocyte count	IPF
CD39+ CD4+ T cell %T cell	GCST90001658	lymphocyte count	IPF
CD39+ activated CD4 regulatory T	GCST90001490	lymphocyte count	IPF
CD39+ activated CD4 regulatory T	GCST90001490	lymphocyte count	IPF
CD39+ activated CD4 regulatory T	GCST90001490	lymphocyte count	IPF
CD39+ activated CD4 regulatory T	GCST90001490	lymphocyte count	IPF
CD39+ activated CD4 regulatory T	GCST90001490	lymphocyte count	IPF
CD39+ activated CD4 regulatory T	GCST90001490	lymphocyte count	IPF
CD39+ activated CD4 regulatory T	GCST90001490	lymphocyte count	IPF
CD39+ activated CD4 regulatory T	GCST90001490	lymphocyte count	IPF
CD4 on CD39+ secreting CD4 reg	GCST90002069	blood protein measurement	IPF
CD4 on CD39+ secreting CD4 reg	GCST90002069	blood protein measurement	IPF
CD4 on CD39+ secreting CD4 reg	GCST90002069	blood protein measurement	IPF
CD3 on CD39+ activated CD4 reg	GCST90001854	blood protein measurement	IPF
CD3 on CD39+ activated CD4 reg	GCST90001854	blood protein measurement	IPF
CD3 on CD39+ activated CD4 reg	GCST90001854	blood protein measurement	IPF
CD3 on CD39+ activated CD4 reg	GCST90001854	blood protein measurement	IPF
CD39 on CD39+ CD4+ T cell	GCST90002032	blood protein measurement	IPF
CD39 on CD39+ CD4+ T cell	GCST90002032	blood protein measurement	IPF
CD39 on CD39+ CD4+ T cell	GCST90002032	blood protein measurement	IPF
CD39 on CD39+ CD4+ T cell	GCST90002032	blood protein measurement	IPF
CD39 on CD39+ activated CD4 re	GCST90002030	blood protein measurement	IPF
CD39 on CD39+ activated CD4 re	GCST90002030	blood protein measurement	IPF
CD39 on CD39+ activated CD4 re	GCST90002030	blood protein measurement	IPF
CD39 on CD39+ activated CD4 re	GCST90002030	blood protein measurement	IPF
CD39 on CD39+ activated CD4 re	GCST90002030	blood protein measurement	IPF
CD39 on CD39+ activated CD4 re	GCST90002030	blood protein measurement	IPF
CD39 on CD39+ secreting CD4 re	GCST90002031	blood protein measurement	IPF
CD39 on CD39+ secreting CD4 re	GCST90002031	blood protein measurement	IPF
CD39 on CD39+ secreting CD4 re	GCST90002031	blood protein measurement	IPF
CD39 on CD39+ secreting CD4 re	GCST90002031	blood protein measurement	IPF
CD39 on CD39+ secreting CD4 re	GCST90002031	blood protein measurement	IPF
CD39 on CD39+ CD8+ T cell	GCST90002029	blood protein measurement	IPF
CD39 on CD39+ CD8+ T cell	GCST90002029	blood protein measurement	IPF
CD28 on CD39+ CD4+ T cell	GCST90001892	blood protein measurement	IPF
CD28 on CD39+ CD4+ T cell	GCST90001892	blood protein measurement	IPF
CD28 on CD39+ CD4+ T cell	GCST90001892	blood protein measurement	IPF
CD28 on CD39+ CD4+ T cell	GCST90001892	blood protein measurement	IPF
CD28 on CD39+ CD4+ T cell	GCST90001892	blood protein measurement	IPF
CD39+ CD4+ T cell %CD4+ T cel	GCST90001659	lymphocyte count	IPF
CD39+ CD4+ T cell %CD4+ T cel	GCST90001659	lymphocyte count	IPF
CD39+ CD4+ T cell %CD4+ T cel	GCST90001659	lymphocyte count	IPF
CD39+ CD4+ T cell %CD4+ T cel	GCST90001659	lymphocyte count	IPF
CD39+ CD4+ T cell %CD4+ T cel	GCST90001659	lymphocyte count	IPF
CD40 on monocytes	GCST90001985	blood protein measurement	IPF
CD40 on monocytes	GCST90001985	blood protein measurement	IPF
CD40 on monocytes	GCST90001985	blood protein measurement	IPF
CD40 on monocytes	GCST90001985	blood protein measurement	IPF
CD25 on IgD+ CD38- unswitched	GCST90001782	blood protein measurement	IPF
CD25++ CD45RA+ CD4 not regul	GCST90001508	lymphocyte count	IPF
CD25++ CD45RA+ CD4 not regul	GCST90001508	lymphocyte count	IPF

CD25++ CD45RA+ CD4 not regul	GCST90001508	lymphocyte count	IPF
CD25++ CD45RA+ CD4 not regul	GCST90001508	lymphocyte count	IPF
CD25++ CD45RA+ CD4 not regul	GCST90001509	lymphocyte count	IPF
CD25++ CD45RA+ CD4 not regul	GCST90001509	lymphocyte count	IPF
CD25++ CD45RA+ CD4 not regul	GCST90001509	lymphocyte count	IPF
CD25++ CD45RA+ CD4 not regul	GCST90001509	lymphocyte count	IPF
CD25 on CD45RA+ CD4 not regu	GCST90001934	blood protein measurement	IPF
CD16-CD56 on Natural Killer	GCST90001884	blood protein measurement	IPF
CD16-CD56 on Natural Killer	GCST90001884	blood protein measurement	IPF
CD16-CD56 on Natural Killer	GCST90001884	blood protein measurement	IPF
CD16-CD56 on Natural Killer	GCST90001884	blood protein measurement	IPF
CD16-CD56 on Natural Killer	GCST90001884	blood protein measurement	IPF
CD16-CD56 on Natural Killer	GCST90001884	blood protein measurement	IPF
CD16-CD56 on Natural Killer	GCST90001884	blood protein measurement	IPF
CD45 on CD33- HLA DR-	GCST90002045	blood protein measurement	IPF
CD45 on CD33- HLA DR-	GCST90002045	blood protein measurement	IPF
CD4+CD8+ T cell Absolute Count	GCST90001594	leukocyte count	IPF
CD4+CD8+ T cell Absolute Count	GCST90001594	leukocyte count	IPF
CD27 on IgD- CD38- B cell	GCST90001802	blood protein measurement	IPF
CD27 on IgD- CD38- B cell	GCST90001802	blood protein measurement	IPF
CD27 on IgD- CD38- B cell	GCST90001802	blood protein measurement	IPF
CD27 on IgD- CD38- B cell	GCST90001802	blood protein measurement	IPF
CD27 on IgD- CD38- B cell	GCST90001802	blood protein measurement	IPF
CD27 on IgD- CD38- B cell	GCST90001802	blood protein measurement	IPF
CD27 on IgD- CD38- B cell	GCST90001802	blood protein measurement	IPF
CD64 on CD14+ CD16+ monocyt	GCST90002011	blood protein measurement	IPF
CD64 on CD14+ CD16+ monocyt	GCST90002011	blood protein measurement	IPF
CD64 on CD14+ CD16+ monocyt	GCST90002011	blood protein measurement	IPF
CD64 on CD14+ CD16+ monocyt	GCST90002011	blood protein measurement	IPF
CD64 on CD14+ CD16+ monocyt	GCST90002011	blood protein measurement	IPF
CD64 on CD14+ CD16+ monocyt	GCST90002011	blood protein measurement	IPF
CD64 on CD14+ CD16+ monocyt	GCST90002011	blood protein measurement	IPF
HLA DR on HLA DR+ CD4+ T ce	GCST90002114	blood protein measurement	IPF
HLA DR on HLA DR+ T cell	GCST90002113	blood protein measurement	IPF
HLA DR on CD33dim HLA DR+	GCST90002110	blood protein measurement	IPF
HLA DR on CD33dim HLA DR+	GCST90002110	blood protein measurement	IPF
HLA DR on CD33dim HLA DR+	GCST90002110	blood protein measurement	IPF
HLA DR on CD33dim HLA DR+	GCST90002111	blood protein measurement	IPF
HLA DR on CD33dim HLA DR+	GCST90002111	blood protein measurement	IPF
HLA DR on CD33dim HLA DR+	GCST90002111	blood protein measurement	IPF
HLA DR on CD33dim HLA DR+	GCST90002111	blood protein measurement	IPF
CD8+ and CD8dim T cell %leuko	GCST90001614	leukocyte count	IPF
CD8+ and CD8dim T cell %leuko	GCST90001614	leukocyte count	IPF
CD27 on memory B cell	GCST90001805	blood protein measurement	IPF
CD27 on memory B cell	GCST90001805	blood protein measurement	IPF
CD27 on memory B cell	GCST90001805	blood protein measurement	IPF
CD27 on memory B cell	GCST90001805	blood protein measurement	IPF
CD27 on memory B cell	GCST90001805	blood protein measurement	IPF
CD27 on memory B cell	GCST90001805	blood protein measurement	IPF
CD27 on memory B cell	GCST90001805	blood protein measurement	IPF
CD27 on memory B cell	GCST90001805	blood protein measurement	IPF
CD27 on CD24+ CD27+ B cell	GCST90001798	blood protein measurement	IPF
CD27 on CD24+ CD27+ B cell	GCST90001798	blood protein measurement	IPF
CD27 on CD24+ CD27+ B cell	GCST90001798	blood protein measurement	IPF
CD27 on CD24+ CD27+ B cell	GCST90001798	blood protein measurement	IPF
CD27 on CD24+ CD27+ B cell	GCST90001798	blood protein measurement	IPF
CD27 on CD24+ CD27+ B cell	GCST90001798	blood protein measurement	IPF
CD27 on CD24+ CD27+ B cell	GCST90001798	blood protein measurement	IPF

CD27 on CD24+ CD27+ B cell	GCST90001798	blood protein measurement	IPF
CD27 on CD24+ CD27+ B cell	GCST90001798	blood protein measurement	IPF
CD27 on CD24+ CD27+ B cell	GCST90001798	blood protein measurement	IPF
CD27 on CD24+ CD27+ B cell	GCST90001798	blood protein measurement	IPF
CD27 on unswitched memory B ce	GCST90001806	blood protein measurement	IPF
CD27 on unswitched memory B ce	GCST90001806	blood protein measurement	IPF
CD27 on unswitched memory B ce	GCST90001806	blood protein measurement	IPF
CD27 on unswitched memory B ce	GCST90001806	blood protein measurement	IPF
CD27 on unswitched memory B ce	GCST90001806	blood protein measurement	IPF
CD27 on unswitched memory B ce	GCST90001806	blood protein measurement	IPF
CD27 on unswitched memory B ce	GCST90001806	blood protein measurement	IPF
CD27 on unswitched memory B ce	GCST90001806	blood protein measurement	IPF
CD27 on unswitched memory B ce	GCST90001806	blood protein measurement	IPF
CD27 on unswitched memory B ce	GCST90001806	blood protein measurement	IPF
CD27 on switched memory B cell	GCST90001808	blood protein measurement	IPF
CD27 on switched memory B cell	GCST90001808	blood protein measurement	IPF
CD27 on switched memory B cell	GCST90001808	blood protein measurement	IPF
CD27 on switched memory B cell	GCST90001808	blood protein measurement	IPF
CD27 on switched memory B cell	GCST90001808	blood protein measurement	IPF
CD27 on switched memory B cell	GCST90001808	blood protein measurement	IPF
CD27 on switched memory B cell	GCST90001808	blood protein measurement	IPF
CD27 on switched memory B cell	GCST90001808	blood protein measurement	IPF
CD27 on switched memory B cell	GCST90001808	blood protein measurement	IPF
CD27 on switched memory B cell	GCST90001808	blood protein measurement	IPF
CD27 on IgD- CD38dim B cell	GCST90001804	blood protein measurement	IPF
CD27 on IgD- CD38dim B cell	GCST90001804	blood protein measurement	IPF
CD27 on IgD- CD38dim B cell	GCST90001804	blood protein measurement	IPF
CD27 on IgD- CD38dim B cell	GCST90001804	blood protein measurement	IPF
CD27 on IgD- CD38dim B cell	GCST90001804	blood protein measurement	IPF
CD27 on IgD- CD38dim B cell	GCST90001804	blood protein measurement	IPF
CD27 on IgD- CD38dim B cell	GCST90001804	blood protein measurement	IPF
CD27 on IgD- CD38dim B cell	GCST90001804	blood protein measurement	IPF
CD27 on IgD- CD38dim B cell	GCST90001804	blood protein measurement	IPF
CD33+ HLA DR+ CD14dim %CD	GCST90001521	myeloid white cell count	IPF
CD45RA- CD4+ T cell %CD4+ T	GCST90001535	lymphocyte count	IPF
CD45RA- CD4+ T cell %CD4+ T	GCST90001535	lymphocyte count	IPF
CD45RA- CD4+ T cell %CD4+ T	GCST90001535	lymphocyte count	IPF
CD25++ CD45RA+ CD4 not regul	GCST90001507	lymphocyte count	IPF
CD25++ CD45RA+ CD4 not regul	GCST90001507	lymphocyte count	IPF
CD25++ CD45RA+ CD4 not regul	GCST90001507	lymphocyte count	IPF
CD45RA on resting CD4 regulator	GCST90002102	blood protein measurement	IPF
CD45RA on resting CD4 regulator	GCST90002102	blood protein measurement	IPF
CD45RA on resting CD4 regulator	GCST90002102	blood protein measurement	IPF
CD45RA on resting CD4 regulator	GCST90002102	blood protein measurement	IPF
CD20 on CD24+ CD27+ B cell	GCST90001745	blood protein measurement	IPF
CD20 on CD24+ CD27+ B cell	GCST90001745	blood protein measurement	IPF
CD20 on CD24+ CD27+ B cell	GCST90001745	blood protein measurement	IPF
CD20 on CD24+ CD27+ B cell	GCST90001745	blood protein measurement	IPF
CD20 on CD24+ CD27+ B cell	GCST90001745	blood protein measurement	IPF
CD20 on CD24+ CD27+ B cell	GCST90001745	blood protein measurement	IPF
CD28+ CD45RA+ CD8+ T cell %	GCST90001688	lymphocyte count	IPF
CD28+ CD45RA+ CD8+ T cell %	GCST90001688	lymphocyte count	IPF
CD28+ CD45RA+ CD8+ T cell %	GCST90001688	lymphocyte count	IPF
CD28+ CD45RA+ CD8+ T cell %	GCST90001688	lymphocyte count	IPF
CD28+ CD45RA+ CD8+ T cell %	GCST90001688	lymphocyte count	IPF
CD28+ CD45RA+ CD8+ T cell %	GCST90001688	lymphocyte count	IPF
CD28+ CD45RA+ CD8+ T cell %	GCST90001688	lymphocyte count	IPF
CD28+ CD45RA+ CD8+ T cell %	GCST90001688	lymphocyte count	IPF

CD28+ CD45RA+ CD8+ T cell %	GCST90001688	lymphocyte count	IPF
CD28+ CD45RA+ CD8+ T cell %	GCST90001688	lymphocyte count	IPF
CD28+ CD45RA+ CD8+ T cell %	GCST90001688	lymphocyte count	IPF
CD28+ CD45RA+ CD8+ T cell %	GCST90001688	lymphocyte count	IPF
CD28+ CD45RA+ CD8+ T cell %	GCST90001688	lymphocyte count	IPF
CD28+ CD45RA+ CD8+ T cell %	GCST90001688	lymphocyte count	IPF
CD28+ CD45RA+ CD8+ T cell %	GCST90001688	lymphocyte count	IPF
CD28+ CD45RA+ CD8+ T cell %	GCST90001688	lymphocyte count	IPF
CD28+ CD45RA+ CD8+ T cell %	GCST90001688	lymphocyte count	IPF
CD28+ CD45RA+ CD8+ T cell %	GCST90001688	lymphocyte count	IPF
CD28+ CD45RA+ CD8+ T cell %	GCST90001688	lymphocyte count	IPF
CD28+ CD45RA+ CD8+ T cell %	GCST90001688	lymphocyte count	IPF
CD28+ CD45RA+ CD8+ T cell %	GCST90001688	lymphocyte count	IPF
CD28+ CD45RA+ CD8+ T cell %	GCST90001688	lymphocyte count	IPF
CD28+ CD45RA+ CD8+ T cell %	GCST90001688	lymphocyte count	IPF
CD28+ CD45RA+ CD8+ T cell %	GCST90001688	lymphocyte count	IPF
CD28+ CD45RA+ CD8+ T cell %	GCST90001688	lymphocyte count	IPF
CD28+ CD45RA+ CD8+ T cell %	GCST90001688	lymphocyte count	IPF
CD28+ CD45RA+ CD8+ T cell %	GCST90001688	lymphocyte count	IPF
CD28+ CD45RA+ CD8+ T cell %	GCST90001688	lymphocyte count	IPF
CD28+ CD45RA+ CD8+ T cell %	GCST90001688	lymphocyte count	IPF
CD28+ CD45RA+ CD8+ T cell %	GCST90001688	lymphocyte count	IPF
CD28+ CD45RA+ CD8+ T cell %	GCST90001688	lymphocyte count	IPF
CD28+ CD45RA+ CD8+ T cell %	GCST90001688	lymphocyte count	IPF
CD28+ CD45RA+ CD8+ T cell %	GCST90001688	lymphocyte count	IPF
CD28+ CD45RA+ CD8+ T cell %	GCST90001688	lymphocyte count	IPF
CD8 on CD39+ CD8+ T cell	GCST90002121	blood protein measurement	IPF
CD8 on CD39+ CD8+ T cell	GCST90002121	blood protein measurement	IPF
CD8 on CD39+ CD8+ T cell	GCST90002121	blood protein measurement	IPF
CD8 on CD39+ CD8+ T cell	GCST90002121	blood protein measurement	IPF
SSC-A on monocyte	GCST90002073	blood protein measurement	IPF
SSC-A on monocyte	GCST90002073	blood protein measurement	IPF
SSC-A on monocyte	GCST90002073	blood protein measurement	IPF
SSC-A on monocyte	GCST90002073	blood protein measurement	IPF
SSC-A on monocyte	GCST90002073	blood protein measurement	IPF
SSC-A on monocyte	GCST90002073	blood protein measurement	IPF
CD45RA- CD28- CD8+ T cell %T	GCST90001697	lymphocyte count	IPF
CD45RA- CD28- CD8+ T cell %T	GCST90001697	lymphocyte count	IPF
CD45RA- CD28- CD8+ T cell %T	GCST90001697	lymphocyte count	IPF
CD45RA- CD28- CD8+ T cell %T	GCST90001697	lymphocyte count	IPF
CD45RA- CD28- CD8+ T cell %T	GCST90001697	lymphocyte count	IPF
CD45RA- CD28- CD8+ T cell %T	GCST90001697	lymphocyte count	IPF
CD45RA- CD28- CD8+ T cell %T	GCST90001697	lymphocyte count	IPF
CD45RA- CD28- CD8+ T cell %T	GCST90001697	lymphocyte count	IPF
CD45RA- CD28- CD8+ T cell %T	GCST90001697	lymphocyte count	IPF
CD45RA- CD28- CD8+ T cell %T	GCST90001697	lymphocyte count	IPF
CD45RA- CD28- CD8+ T cell %T	GCST90001697	lymphocyte count	IPF
CD45RA- CD28- CD8+ T cell %T	GCST90001697	lymphocyte count	IPF
CD45RA- CD28- CD8+ T cell %T	GCST90001697	lymphocyte count	IPF
CD45RA- CD28- CD8+ T cell %T	GCST90001697	lymphocyte count	IPF
CD45RA- CD28- CD8+ T cell %T	GCST90001697	lymphocyte count	IPF
CD45RA- CD28- CD8+ T cell %T	GCST90001697	lymphocyte count	IPF
CD45RA- CD28- CD8+ T cell %T	GCST90001697	lymphocyte count	IPF
CD45RA- CD28- CD8+ T cell %T	GCST90001697	lymphocyte count	IPF
CD45RA- CD28- CD8+ T cell %T	GCST90001697	lymphocyte count	IPF
CD45RA- CD28- CD8+ T cell %T	GCST90001697	lymphocyte count	IPF
CD45RA- CD28- CD8+ T cell %T	GCST90001697	lymphocyte count	IPF
CD45RA- CD28- CD8+ T cell %T	GCST90001697	lymphocyte count	IPF

CD45RA- CD28- CD8+ T cell %T	GCST90001697	lymphocyte count	IPF
CD45RA- CD28- CD8+ T cell %T	GCST90001697	lymphocyte count	IPF
CD45RA- CD28- CD8+ T cell %T	GCST90001697	lymphocyte count	IPF
CD45RA- CD28- CD8+ T cell %T	GCST90001697	lymphocyte count	IPF
CD45RA- CD28- CD8+ T cell %T	GCST90001697	lymphocyte count	IPF
CD45RA- CD28- CD8+ T cell %T	GCST90001697	lymphocyte count	IPF
CD45RA- CD28- CD8+ T cell %T	GCST90001697	lymphocyte count	IPF
CD45RA- CD28- CD8+ T cell %T	GCST90001697	lymphocyte count	IPF
CD45RA- CD28- CD8+ T cell %T	GCST90001697	lymphocyte count	IPF
CD45RA- CD28- CD8+ T cell %T	GCST90001697	lymphocyte count	IPF
CD45RA- CD28- CD8+ T cell %T	GCST90001697	lymphocyte count	IPF
CD45RA- CD28- CD8+ T cell %T	GCST90001697	lymphocyte count	IPF
CD45RA- CD28- CD8+ T cell %T	GCST90001697	lymphocyte count	IPF
CD45RA- CD28- CD8+ T cell %T	GCST90001697	lymphocyte count	IPF
CD45RA- CD28- CD8+ T cell Ab	GCST90001695	lymphocyte count	IPF
CD45RA- CD28- CD8+ T cell Ab	GCST90001695	lymphocyte count	IPF
CD45RA- CD28- CD8+ T cell Ab	GCST90001695	lymphocyte count	IPF
CD45RA- CD28- CD8+ T cell Ab	GCST90001695	lymphocyte count	IPF
CD45RA- CD28- CD8+ T cell Ab	GCST90001695	lymphocyte count	IPF
CD45RA- CD28- CD8+ T cell Ab	GCST90001695	lymphocyte count	IPF
CD45RA- CD28- CD8+ T cell Ab	GCST90001695	lymphocyte count	IPF
CD45RA- CD28- CD8+ T cell Ab	GCST90001695	lymphocyte count	IPF
CD45RA- CD28- CD8+ T cell Ab	GCST90001695	lymphocyte count	IPF
CD45RA- CD28- CD8+ T cell Ab	GCST90001695	lymphocyte count	IPF
CD45RA- CD28- CD8+ T cell Ab	GCST90001695	lymphocyte count	IPF
CD45RA- CD28- CD8+ T cell Ab	GCST90001695	lymphocyte count	IPF
CD45RA- CD28- CD8+ T cell Ab	GCST90001695	lymphocyte count	IPF
CD45RA- CD28- CD8+ T cell Ab	GCST90001695	lymphocyte count	IPF
CD45RA- CD28- CD8+ T cell Ab	GCST90001695	lymphocyte count	IPF
CD45RA- CD28- CD8+ T cell Ab	GCST90001695	lymphocyte count	IPF
CD45RA- CD28- CD8+ T cell Ab	GCST90001695	lymphocyte count	IPF
CD45RA- CD28- CD8+ T cell Ab	GCST90001695	lymphocyte count	IPF
CD45RA- CD28- CD8+ T cell Ab	GCST90001695	lymphocyte count	IPF
CD45RA- CD28- CD8+ T cell Ab	GCST90001695	lymphocyte count	IPF
CD45RA- CD28- CD8+ T cell Ab	GCST90001695	lymphocyte count	IPF
CD45RA- CD28- CD8+ T cell Ab	GCST90001695	lymphocyte count	IPF
CD45RA- CD28- CD8+ T cell Ab	GCST90001695	lymphocyte count	IPF
CD45RA- CD28- CD8+ T cell Ab	GCST90001695	lymphocyte count	IPF
CD45RA- CD28- CD8+ T cell Ab	GCST90001695	lymphocyte count	IPF
CD45RA- CD28- CD8+ T cell Ab	GCST90001695	lymphocyte count	IPF
CD45RA- CD28- CD8+ T cell Ab	GCST90001695	lymphocyte count	IPF
CD45RA- CD28- CD8+ T cell Ab	GCST90001695	lymphocyte count	IPF
CD45RA- CD28- CD8+ T cell Ab	GCST90001695	lymphocyte count	IPF
CD45RA- CD28- CD8+ T cell Ab	GCST90001695	lymphocyte count	IPF
CD45RA- CD28- CD8+ T cell Ab	GCST90001695	lymphocyte count	IPF
CD45RA- CD28- CD8+ T cell Ab	GCST90001695	lymphocyte count	IPF
CD45RA- CD28- CD8+ T cell Ab	GCST90001695	lymphocyte count	IPF
CD45RA- CD28- CD8+ T cell Ab	GCST90001695	lymphocyte count	IPF
CD45RA- CD28- CD8+ T cell Ab	GCST90001695	lymphocyte count	IPF
CD45RA- CD28- CD8+ T cell Ab	GCST90001695	lymphocyte count	IPF
CD45RA- CD28- CD8+ T cell Ab	GCST90001695	lymphocyte count	IPF
CD45RA- CD28- CD8+ T cell Ab	GCST90001695	lymphocyte count	IPF
CD45RA- CD28- CD8+ T cell Ab	GCST90001695	lymphocyte count	IPF
CD45RA- CD28- CD8+ T cell Ab	GCST90001695	lymphocyte count	IPF
CD45RA- CD28- CD8+ T cell Ab	GCST90001695	lymphocyte count	IPF
CD45RA- CD28- CD8+ T cell Ab	GCST90001695	lymphocyte count	IPF
CD45RA- CD28- CD8+ T cell Ab	GCST90001695	lymphocyte count	IPF
CD45RA- CD28- CD8+ T cell Ab	GCST90001695	lymphocyte count	IPF

CD45RA- CD28- CD8+ T cell Ab	GCST90001695	lymphocyte count	IPF
CD45RA- CD28- CD8+ T cell Ab	GCST90001695	lymphocyte count	IPF
CD45RA- CD28- CD8+ T cell Ab	GCST90001695	lymphocyte count	IPF
CD45RA- CD28- CD8+ T cell Ab	GCST90001695	lymphocyte count	IPF
CD45RA- CD28- CD8+ T cell Ab	GCST90001695	lymphocyte count	IPF
CD45RA- CD28- CD8+ T cell Ab	GCST90001695	lymphocyte count	IPF
CD45RA- CD28- CD8+ T cell Ab	GCST90001695	lymphocyte count	IPF
CD45RA- CD28- CD8+ T cell Ab	GCST90001695	lymphocyte count	IPF
CD45RA- CD28- CD8+ T cell Ab	GCST90001695	lymphocyte count	IPF
CD45RA- CD28- CD8+ T cell Ab	GCST90001695	lymphocyte count	IPF
CD45RA- CD28- CD8+ T cell Ab	GCST90001695	lymphocyte count	IPF
CD45RA- CD28- CD8+ T cell Ab	GCST90001695	lymphocyte count	IPF
CD45RA- CD28- CD8+ T cell Ab	GCST90001695	lymphocyte count	IPF
CD45RA- CD28- CD8+ T cell Ab	GCST90001695	lymphocyte count	IPF
CD45RA- CD28- CD8+ T cell Ab	GCST90001695	lymphocyte count	IPF
CD45RA- CD28- CD8+ T cell Ab	GCST90001695	lymphocyte count	IPF
CD45RA- CD28- CD8+ T cell Ab	GCST90001695	lymphocyte count	IPF
CD45RA- CD28- CD8+ T cell Ab	GCST90001695	lymphocyte count	IPF
CD45RA- CD28- CD8+ T cell Ab	GCST90001695	lymphocyte count	IPF
CD45RA- CD28- CD8+ T cell Ab	GCST90001695	lymphocyte count	IPF
CD45RA- CD28- CD8+ T cell Ab	GCST90001695	lymphocyte count	IPF
CD45RA- CD28- CD8+ T cell Ab	GCST90001695	lymphocyte count	IPF
CD45RA- CD28- CD8+ T cell Ab	GCST90001695	lymphocyte count	IPF
CD45RA- CD28- CD8+ T cell Ab	GCST90001695	lymphocyte count	IPF
CD45RA- CD28- CD8+ T cell Ab	GCST90001695	lymphocyte count	IPF
CD45RA- CD28- CD8+ T cell Ab	GCST90001695	lymphocyte count	IPF
CD45RA- CD28- CD8+ T cell Ab	GCST90001695	lymphocyte count	IPF
CD45RA- CD28- CD8+ T cell Ab	GCST90001695	lymphocyte count	IPF
CD45RA- CD28- CD8+ T cell Ab	GCST90001695	lymphocyte count	IPF
CD45RA- CD28- CD8+ T cell Ab	GCST90001695	lymphocyte count	IPF
CD45RA- CD28- CD8+ T cell Ab	GCST90001695	lymphocyte count	IPF
CD45RA- CD28- CD8+ T cell Ab	GCST90001695	lymphocyte count	IPF
CD45RA- CD28- CD8+ T cell Ab	GCST90001695	lymphocyte count	IPF
CD45RA- CD28- CD8+ T cell Ab	GCST90001695	lymphocyte count	IPF
CD45RA- CD28- CD8+ T cell Ab	GCST90001695	lymphocyte count	IPF
CD45RA- CD28- CD8+ T cell Ab	GCST90001695	lymphocyte count	IPF
CD45RA- CD28- CD8+ T cell Ab	GCST90001695	lymphocyte count	IPF
CD45RA- CD28- CD8+ T cell Ab	GCST90001695	lymphocyte count	IPF
CD45RA- CD28- CD8+ T cell Ab	GCST90001695	lymphocyte count	IPF
CD45RA- CD28- CD8+ T cell Ab	GCST90001695	lymphocyte count	IPF
CD45RA- CD28- CD8+ T cell Ab	GCST90001695	lymphocyte count	IPF
CD45RA- CD28- CD8+ T cell Ab	GCST90001695	lymphocyte count	IPF
CD45RA- CD28- CD8+ T cell Ab	GCST90001695	lymphocyte count	IPF
CD45RA- CD28- CD8+ T cell Ab	GCST90001695	lymphocyte count	IPF
CD45RA- CD28- CD8+ T cell Ab	GCST90001695	lymphocyte count	IPF
CD45RA- CD28- CD8+ T cell Ab	GCST90001695	lymphocyte count	IPF
CD45RA- CD28- CD8+ T cell Ab	GCST90001695	lymphocyte count	IPF
CD45RA- CD28- CD8+ T cell Ab	GCST90001695	lymphocyte count	IPF
CD45RA- CD28- CD8+ T cell Ab	GCST90001695	lymphocyte count	IPF
CD45RA- CD28- CD8+ T cell Ab	GCST90001695	lymphocyte count	IPF
CD45RA- CD28- CD8+ T cell Ab	GCST90001695	lymphocyte count	IPF
CD45RA- CD28- CD8+ T cell Ab	GCST90001695	lymphocyte count	IPF
CD45RA- CD28- CD8+ T cell Ab	GCST90001695	lymphocyte count	IPF
CD45RA- CD28- CD8+ T cell Ab	GCST90001695	lymphocyte count	IPF
CD45RA- CD28- CD8+ T cell Ab	GCST90001695	lymphocyte count	IPF
CD45RA- CD28- CD8+ T cell Ab	GCST90001695	lymphocyte count	IPF
CD45RA- CD28- CD8+ T cell Ab	GCST90001695	lymphocyte count	IPF
CD45RA- CD28- CD8+ T cell Ab	GCST90001695	lymphocyte count	IPF

CD45RA- CD28- CD8+ T cell Ab:	GCST90001695	lymphocyte count	IPF
CD45RA- CD28- CD8+ T cell Ab:	GCST90001695	lymphocyte count	IPF
CD45RA- CD28- CD8+ T cell Ab:	GCST90001695	lymphocyte count	IPF
CD45RA- CD28- CD8+ T cell Ab:	GCST90001695	lymphocyte count	IPF
CD45RA- CD28- CD8+ T cell Ab:	GCST90001695	lymphocyte count	IPF
CD45RA- CD28- CD8+ T cell Ab:	GCST90001695	lymphocyte count	IPF
CD45RA- CD28- CD8+ T cell Ab:	GCST90001695	lymphocyte count	IPF
CD45RA- CD28- CD8+ T cell Ab:	GCST90001695	lymphocyte count	IPF
CD45RA- CD28- CD8+ T cell Ab:	GCST90001695	lymphocyte count	IPF
CD45RA- CD28- CD8+ T cell Ab:	GCST90001695	lymphocyte count	IPF
CD45RA- CD28- CD8+ T cell Ab:	GCST90001695	lymphocyte count	IPF
CD45RA- CD28- CD8+ T cell Ab:	GCST90001695	lymphocyte count	IPF
CD45RA- CD28- CD8+ T cell Ab:	GCST90001695	lymphocyte count	IPF
CD45RA- CD28- CD8+ T cell Ab:	GCST90001695	lymphocyte count	IPF
CD45RA- CD28- CD8+ T cell Ab:	GCST90001695	lymphocyte count	IPF
CD45RA- CD28- CD8+ T cell Ab:	GCST90001695	lymphocyte count	IPF
CD45RA- CD28- CD8+ T cell Ab:	GCST90001695	lymphocyte count	IPF
CD45RA- CD28- CD8+ T cell Ab:	GCST90001695	lymphocyte count	IPF
CD45RA- CD28- CD8+ T cell Ab:	GCST90001695	lymphocyte count	IPF
CD45RA- CD28- CD8+ T cell Ab:	GCST90001695	lymphocyte count	IPF
CD40 on CD14- CD16+ monocyte	GCST90001989	blood protein measurement	IPF
CD40 on CD14- CD16+ monocyte	GCST90001989	blood protein measurement	IPF
CD40 on CD14- CD16+ monocyte	GCST90001989	blood protein measurement	IPF
CD40 on CD14- CD16+ monocyte	GCST90001989	blood protein measurement	IPF
CD40 on CD14- CD16+ monocyte	GCST90001989	blood protein measurement	IPF
CD40 on CD14- CD16+ monocyte	GCST90001989	blood protein measurement	IPF
CD40 on CD14- CD16+ monocyte	GCST90001989	blood protein measurement	IPF
CD40 on CD14- CD16+ monocyte	GCST90001989	blood protein measurement	IPF
CD39+ resting CD4 regulatory T c	GCST90001484	lymphocyte count	IPF
CD39+ resting CD4 regulatory T c	GCST90001484	lymphocyte count	IPF
CD39+ resting CD4 regulatory T c	GCST90001484	lymphocyte count	IPF
CD39+ resting CD4 regulatory T c	GCST90001484	lymphocyte count	IPF
CD39+ resting CD4 regulatory T c	GCST90001484	lymphocyte count	IPF
CD39+ resting CD4 regulatory T c	GCST90001484	lymphocyte count	IPF
CD39+ resting CD4 regulatory T c	GCST90001484	lymphocyte count	IPF
CD39+ resting CD4 regulatory T c	GCST90001484	lymphocyte count	IPF
CD39+ resting CD4 regulatory T c	GCST90001484	lymphocyte count	IPF
CD39+ CD8+ T cell %T cell	GCST90001670	lymphocyte count	IPF
CD39+ CD8+ T cell %T cell	GCST90001670	lymphocyte count	IPF
CD39+ CD8+ T cell %T cell	GCST90001670	lymphocyte count	IPF
CD39+ CD8+ T cell %T cell	GCST90001670	lymphocyte count	IPF
CD39+ CD8+ T cell %T cell	GCST90001670	lymphocyte count	IPF
CD39+ CD8+ T cell %T cell	GCST90001670	lymphocyte count	IPF
CD39+ CD8+ T cell %T cell	GCST90001670	lymphocyte count	IPF
CD39+ CD8+ T cell %T cell	GCST90001670	lymphocyte count	IPF
CD39+ CD8+ T cell Absolute Cou	GCST90001672	lymphocyte count	IPF
CD39+ CD8+ T cell Absolute Cou	GCST90001672	lymphocyte count	IPF
CD39+ CD8+ T cell Absolute Cou	GCST90001672	lymphocyte count	IPF
CD39+ CD8+ T cell Absolute Cou	GCST90001672	lymphocyte count	IPF
CD39+ CD8+ T cell Absolute Cou	GCST90001672	lymphocyte count	IPF
CD39+ CD8+ T cell Absolute Cou	GCST90001672	lymphocyte count	IPF
CD39+ CD8+ T cell Absolute Cou	GCST90001672	lymphocyte count	IPF
CD39+ resting CD4 regulatory T c	GCST90001485	lymphocyte count	IPF
CD39+ resting CD4 regulatory T c	GCST90001485	lymphocyte count	IPF
CD39+ resting CD4 regulatory T c	GCST90001485	lymphocyte count	IPF
CD39+ resting CD4 regulatory T c	GCST90001485	lymphocyte count	IPF
CD39+ resting CD4 regulatory T c	GCST90001485	lymphocyte count	IPF
CD39+ resting CD4 regulatory T c	GCST90001485	lymphocyte count	IPF

CD39+ resting CD4 regulatory T c	GCST90001485	lymphocyte count	IPF
CD39+ resting CD4 regulatory T c	GCST90001485	lymphocyte count	IPF
CD39+ resting CD4 regulatory T c	GCST90001485	lymphocyte count	IPF
CD39+ resting CD4 regulatory T c	GCST90001485	lymphocyte count	IPF
CD39+ resting CD4 regulatory T c	GCST90001485	lymphocyte count	IPF
CD39+ resting CD4 regulatory T c	GCST90001485	lymphocyte count	IPF
CD39+ resting CD4 regulatory T c	GCST90001485	lymphocyte count	IPF
CD39+ resting CD4 regulatory T c	GCST90001485	lymphocyte count	IPF
CD39+ resting CD4 regulatory T c	GCST90001483	lymphocyte count	IPF
CD39+ resting CD4 regulatory T c	GCST90001483	lymphocyte count	IPF
CD39+ resting CD4 regulatory T c	GCST90001483	lymphocyte count	IPF
CD39+ resting CD4 regulatory T c	GCST90001483	lymphocyte count	IPF
CD39+ resting CD4 regulatory T c	GCST90001483	lymphocyte count	IPF
CD39+ resting CD4 regulatory T c	GCST90001483	lymphocyte count	IPF
CD39+ resting CD4 regulatory T c	GCST90001483	lymphocyte count	IPF
CD39+ resting CD4 regulatory T c	GCST90001483	lymphocyte count	IPF
CD39+ resting CD4 regulatory T c	GCST90001483	lymphocyte count	IPF
CD39+ resting CD4 regulatory T c	GCST90001483	lymphocyte count	IPF
CD39+ resting CD4 regulatory T c	GCST90001483	lymphocyte count	IPF
CD39+ resting CD4 regulatory T c	GCST90001483	lymphocyte count	IPF
CD39+ CD8+ T cell %CD8+ T cel	GCST90001671	lymphocyte count	IPF
CD39+ CD8+ T cell %CD8+ T cel	GCST90001671	lymphocyte count	IPF
CD39+ CD8+ T cell %CD8+ T cel	GCST90001671	lymphocyte count	IPF
CD39+ CD8+ T cell %CD8+ T cel	GCST90001671	lymphocyte count	IPF
CD39+ CD8+ T cell %CD8+ T cel	GCST90001671	lymphocyte count	IPF
CD39+ CD8+ T cell %CD8+ T cel	GCST90001671	lymphocyte count	IPF
CD39+ CD8+ T cell %CD8+ T cel	GCST90001671	lymphocyte count	IPF
CD4 on naive CD4+ T cell	GCST90002024	blood protein measurement	IPF
CD4 on naive CD4+ T cell	GCST90002024	blood protein measurement	IPF
CD4 on naive CD4+ T cell	GCST90002024	blood protein measurement	IPF
CD4 on naive CD4+ T cell	GCST90002024	blood protein measurement	IPF
CD33 on basophil	GCST90001954	blood protein measurement	IPF
CD33 on basophil	GCST90001954	blood protein measurement	IPF
CD33 on basophil	GCST90001954	blood protein measurement	IPF
CD33 on basophil	GCST90001954	blood protein measurement	IPF
CD33 on basophil	GCST90001954	blood protein measurement	IPF
CD33 on basophil	GCST90001954	blood protein measurement	IPF
CD33 on basophil	GCST90001954	blood protein measurement	IPF
CD33 on Immature Myeloid-Deriv	GCST90001955	blood protein measurement	IPF
CD33 on Immature Myeloid-Deriv	GCST90001955	blood protein measurement	IPF
CD33 on Immature Myeloid-Deriv	GCST90001955	blood protein measurement	IPF
CD33 on Immature Myeloid-Deriv	GCST90001955	blood protein measurement	IPF
CD33 on Immature Myeloid-Deriv	GCST90001955	blood protein measurement	IPF
CD33 on Immature Myeloid-Deriv	GCST90001955	blood protein measurement	IPF
CD45 on Immature Myeloid-Deriv	GCST90002052	blood protein measurement	IPF
CD45 on Immature Myeloid-Deriv	GCST90002052	blood protein measurement	IPF
CD3 on naive CD8+ T cell	GCST90001838	blood protein measurement	IPF
CD3 on naive CD8+ T cell	GCST90001838	blood protein measurement	IPF
CD3 on naive CD8+ T cell	GCST90001838	blood protein measurement	IPF
CD3 on naive CD8+ T cell	GCST90001838	blood protein measurement	IPF
CD3 on naive CD8+ T cell	GCST90001838	blood protein measurement	IPF
CD3 on naive CD8+ T cell	GCST90001838	blood protein measurement	IPF
CD3 on naive CD8+ T cell	GCST90001838	blood protein measurement	IPF
CD20 on IgD- CD38dim B cell	GCST90001757	blood protein measurement	IPF
CD20 on IgD- CD38dim B cell	GCST90001757	blood protein measurement	IPF
CD20 on IgD- CD38dim B cell	GCST90001757	blood protein measurement	IPF
CD20 on IgD- CD38dim B cell	GCST90001757	blood protein measurement	IPF
CD20 on IgD- CD38dim B cell	GCST90001757	blood protein measurement	IPF

CD20 on IgD- CD38dim B cell	GCST90001757	blood protein measurement	IPF
CD20 on IgD- CD38dim B cell	GCST90001757	blood protein measurement	IPF
CD25 on CD39+ CD4+ T cell	GCST90001961	blood protein measurement	IPF
CD25 on CD39+ CD4+ T cell	GCST90001961	blood protein measurement	IPF
CD25 on CD39+ CD4+ T cell	GCST90001961	blood protein measurement	IPF
CD25 on CD39+ CD4+ T cell	GCST90001961	blood protein measurement	IPF
CD25 on CD39+ CD4+ T cell	GCST90001961	blood protein measurement	IPF
CD25 on CD39+ CD4+ T cell	GCST90001961	blood protein measurement	IPF
Effector Memory CD4-CD8- T cel	GCST90001570	lymphocyte count	IPF
Effector Memory CD4-CD8- T cel	GCST90001570	lymphocyte count	IPF
Effector Memory CD4-CD8- T cel	GCST90001569	lymphocyte count	IPF
Effector Memory CD4-CD8- T cel	GCST90001569	lymphocyte count	IPF
Effector Memory CD4-CD8- T cel	GCST90001569	lymphocyte count	IPF
Effector Memory CD4-CD8- T cel	GCST90001569	lymphocyte count	IPF
HLA DR on B cell	GCST90002116	blood protein measurement	IPF
HLA DR on B cell	GCST90002116	blood protein measurement	IPF
HLA DR on B cell	GCST90002116	blood protein measurement	IPF
HLA DR on B cell	GCST90002116	blood protein measurement	IPF
HLA DR on B cell	GCST90002116	blood protein measurement	IPF
HLA DR on B cell	GCST90002116	blood protein measurement	IPF
HLA DR on B cell	GCST90002116	blood protein measurement	IPF
HLA DR on B cell	GCST90002116	blood protein measurement	IPF
HLA DR on B cell	GCST90002116	blood protein measurement	IPF
HLA DR on B cell	GCST90002116	blood protein measurement	IPF
Terminally Differentiated CD4-CD	GCST90001574	lymphocyte count	IPF
Terminally Differentiated CD4-CD	GCST90001574	lymphocyte count	IPF
CD16-CD56 on Natural Killer T	GCST90001883	blood protein measurement	IPF
CD16-CD56 on Natural Killer T	GCST90001883	blood protein measurement	IPF
CD16-CD56 on Natural Killer T	GCST90001883	blood protein measurement	IPF
CD16-CD56 on Natural Killer T	GCST90001883	blood protein measurement	IPF
CD20 on unswitched memory B ce	GCST90001760	blood protein measurement	IPF
CD20 on unswitched memory B ce	GCST90001760	blood protein measurement	IPF
CD20 on unswitched memory B ce	GCST90001760	blood protein measurement	IPF
CD20 on unswitched memory B ce	GCST90001760	blood protein measurement	IPF
CD19 on IgD+ CD38dim B cell	GCST90001730	blood protein measurement	IPF
CD19 on IgD+ CD38dim B cell	GCST90001730	blood protein measurement	IPF
CD19 on IgD+ CD38dim B cell	GCST90001730	blood protein measurement	IPF
CD19 on IgD- CD38dim B cell	GCST90001735	blood protein measurement	IPF
CD19 on IgD- CD38dim B cell	GCST90001735	blood protein measurement	IPF
IgD on IgD+ CD24+ B cell	GCST90001820	blood protein measurement	IPF
IgD on IgD+ CD24+ B cell	GCST90001820	blood protein measurement	IPF
IgD on IgD+ CD24+ B cell	GCST90001820	blood protein measurement	IPF
IgD on IgD+ CD24+ B cell	GCST90001820	blood protein measurement	IPF
IgD on IgD+ CD24+ B cell	GCST90001820	blood protein measurement	IPF
CD62L- monocyte Absolute Count	GCST90001450	leukocyte count	IPF
CD62L- monocyte Absolute Count	GCST90001450	leukocyte count	IPF
Activated & secreting CD4 regulat	GCST90001503	lymphocyte count	IPF
Activated & secreting CD4 regulat	GCST90001503	lymphocyte count	IPF
Activated & secreting CD4 regulat	GCST90001503	lymphocyte count	IPF
Activated & secreting CD4 regulat	GCST90001503	lymphocyte count	IPF
Activated & secreting CD4 regulat	GCST90001503	lymphocyte count	IPF
Activated & secreting CD4 regulat	GCST90001503	lymphocyte count	IPF
Activated & secreting CD4 regulat	GCST90001503	lymphocyte count	IPF
CD28 on CD28+ CD45RA+ CD8+	GCST90001896	blood protein measurement	IPF
CD28 on CD28+ CD45RA+ CD8+	GCST90001896	blood protein measurement	IPF
CD28+ CD45RA+ CD8dim T cell	GCST90001666	lymphocyte count	IPF
CD28+ CD45RA+ CD8dim T cell	GCST90001666	lymphocyte count	IPF
CD28+ CD45RA+ CD8dim T cell	GCST90001666	lymphocyte count	IPF

CD28+ CD45RA+ CD8dim T cell	GCST90001666	lymphocyte count	IPF
CD28+ CD45RA+ CD8dim T cell	GCST90001666	lymphocyte count	IPF
CD28+ CD45RA+ CD8dim T cell	GCST90001666	lymphocyte count	IPF
CD28+ CD45RA+ CD8dim T cell	GCST90001666	lymphocyte count	IPF
CD64 on CD14+ CD16- monocyte	GCST90001987	blood protein measurement	IPF
CD64 on CD14+ CD16- monocyte	GCST90001987	blood protein measurement	IPF
CD64 on CD14+ CD16- monocyte	GCST90001987	blood protein measurement	IPF
CD64 on CD14+ CD16- monocyte	GCST90001987	blood protein measurement	IPF
CD64 on CD14+ CD16- monocyte	GCST90001987	blood protein measurement	IPF
CD64 on CD14+ CD16- monocyte	GCST90001987	blood protein measurement	IPF
CD64 on CD14+ CD16- monocyte	GCST90001987	blood protein measurement	IPF
CD64 on CD14+ CD16- monocyte	GCST90001987	blood protein measurement	IPF
CD64 on monocyte	GCST90002006	blood protein measurement	IPF
CD64 on monocyte	GCST90002006	blood protein measurement	IPF
CD64 on monocyte	GCST90002006	blood protein measurement	IPF
CD64 on monocyte	GCST90002006	blood protein measurement	IPF
CD64 on monocyte	GCST90002006	blood protein measurement	IPF
CD64 on monocyte	GCST90002006	blood protein measurement	IPF
CD64 on monocyte	GCST90002006	blood protein measurement	IPF
CD25 on CD39+ secreting CD4 re	GCST90001942	blood protein measurement	IPF
CD25 on activated & secreting CD	GCST90001943	blood protein measurement	IPF
SSC-A on myeloid Dendritic Cell	GCST90002071	blood protein measurement	IPF
SSC-A on myeloid Dendritic Cell	GCST90002071	blood protein measurement	IPF
SSC-A on myeloid Dendritic Cell	GCST90002071	blood protein measurement	IPF
SSC-A on myeloid Dendritic Cell	GCST90002071	blood protein measurement	IPF
SSC-A on myeloid Dendritic Cell	GCST90002071	blood protein measurement	IPF
SSC-A on myeloid Dendritic Cell	GCST90002071	blood protein measurement	IPF
Terminally Differentiated CD4+ T	GCST90001547	lymphocyte count	IPF
Terminally Differentiated CD4+ T	GCST90001547	lymphocyte count	IPF
Naive CD4+ T cell %CD4+ T cell	GCST90001541	lymphocyte count	IPF
Terminally Differentiated CD4+ T	GCST90001546	lymphocyte count	IPF
SSC-A on CD4+ T cell	GCST90002081	blood protein measurement	IPF
IgD+ CD38- B cell %B cell	GCST90001395	lymphocyte count	IPF
Central Memory CD8+ T cell Abs	GCST90001548	lymphocyte count	IPF
Activated CD4 regulatory T cell A	GCST90001486	lymphocyte count	IPF
Activated CD4 regulatory T cell A	GCST90001486	lymphocyte count	IPF
Effector Memory CD4-CD8- T cel	GCST90001571	lymphocyte count	IPF
Effector Memory CD4-CD8- T cel	GCST90001571	lymphocyte count	IPF
Effector Memory CD4-CD8- T cel	GCST90001571	lymphocyte count	IPF
BAFF-R on CD20- B cell	GCST90001830	blood protein measurement	IPF
CD19 on IgD+ CD38+ B cell	GCST90001729	blood protein measurement	IPF
CD19 on IgD+ CD38+ B cell	GCST90001729	blood protein measurement	IPF
CD19 on IgD+ CD38+ B cell	GCST90001729	blood protein measurement	IPF
CD19 on IgD+ B cell	GCST90001741	blood protein measurement	IPF
CD19 on IgD+ B cell	GCST90001741	blood protein measurement	IPF
CD19 on transitional B cell	GCST90001742	blood protein measurement	IPF
CD19 on transitional B cell	GCST90001742	blood protein measurement	IPF
CD19 on transitional B cell	GCST90001742	blood protein measurement	IPF
CD19 on IgD+ CD38- unswitched	GCST90001728	blood protein measurement	IPF
CD19 on IgD+ CD38- unswitched	GCST90001728	blood protein measurement	IPF
CD19 on IgD+ CD38- unswitched	GCST90001728	blood protein measurement	IPF
CD19 on IgD+ CD24- B cell	GCST90001725	blood protein measurement	IPF
CD19 on IgD+ CD24- B cell	GCST90001725	blood protein measurement	IPF
CD19 on IgD+ CD24- B cell	GCST90001725	blood protein measurement	IPF
CD19 on unswitched memory B ce	GCST90001738	blood protein measurement	IPF
CD19 on unswitched memory B ce	GCST90001738	blood protein measurement	IPF
CD19 on unswitched memory B ce	GCST90001738	blood protein measurement	IPF
CD19 on IgD+ CD24+ B cell	GCST90001724	blood protein measurement	IPF

CD19 on IgD+ CD24+ B cell	GCST90001724	blood protein measurement	IPF
CD19 on IgD+ CD24+ B cell	GCST90001724	blood protein measurement	IPF
Immature Myeloid-Derived Suppre	GCST90001516	myeloid white cell count	IPF
Immature Myeloid-Derived Suppre	GCST90001516	myeloid white cell count	IPF
HLA DR on CD33- HLA DR+	GCST90002112	blood protein measurement	IPF
HLA DR on CD33- HLA DR+	GCST90002112	blood protein measurement	IPF
HLA DR on CD33- HLA DR+	GCST90002112	blood protein measurement	IPF
CD45RA- CD28- CD8+ T cell %C	GCST90001696	lymphocyte count	IPF
CD45RA- CD28- CD8+ T cell %C	GCST90001696	lymphocyte count	IPF
CD45RA- CD28- CD8+ T cell %C	GCST90001696	lymphocyte count	IPF
CD45RA- CD28- CD8+ T cell %C	GCST90001696	lymphocyte count	IPF
CD45RA- CD28- CD8+ T cell %C	GCST90001696	lymphocyte count	IPF
CD45RA- CD28- CD8+ T cell %C	GCST90001696	lymphocyte count	IPF
CD45RA- CD28- CD8+ T cell %C	GCST90001696	lymphocyte count	IPF
CD45RA- CD28- CD8+ T cell %C	GCST90001696	lymphocyte count	IPF
CD45RA- CD28- CD8+ T cell %C	GCST90001696	lymphocyte count	IPF
Basophil Absolute Count	GCST90001532	myeloid white cell count	IPF
Basophil Absolute Count	GCST90001532	myeloid white cell count	IPF
Basophil Absolute Count	GCST90001532	myeloid white cell count	IPF
CD33dim HLA DR- Absolute Cou	GCST90001531	myeloid white cell count	IPF
CD33dim HLA DR- Absolute Cou	GCST90001531	myeloid white cell count	IPF
CD3 on secreting CD4 regulatory '	GCST90001855	blood protein measurement	IPF
CD3 on secreting CD4 regulatory '	GCST90001855	blood protein measurement	IPF
CD3 on secreting CD4 regulatory '	GCST90001855	blood protein measurement	IPF
CD3 on activated & secreting CD4	GCST90001857	blood protein measurement	IPF
CD3 on activated & secreting CD4	GCST90001857	blood protein measurement	IPF
CD3 on activated & secreting CD4	GCST90001857	blood protein measurement	IPF
CD3 on activated & secreting CD4	GCST90001857	blood protein measurement	IPF
CD3 on activated CD4 regulatory '	GCST90001853	blood protein measurement	IPF
CD3 on activated CD4 regulatory '	GCST90001853	blood protein measurement	IPF
CD3 on activated CD4 regulatory '	GCST90001853	blood protein measurement	IPF
CD3 on activated CD4 regulatory '	GCST90001853	blood protein measurement	IPF
CD3 on CD4 regulatory T cell	GCST90001868	blood protein measurement	IPF
CD3 on CD4 regulatory T cell	GCST90001868	blood protein measurement	IPF
CD3 on CD4 regulatory T cell	GCST90001868	blood protein measurement	IPF
CD45RA+ CD8+ T cell Absolute (	GCST90001560	lymphocyte count	IPF
CD45RA+ CD8+ T cell Absolute (	GCST90001560	lymphocyte count	IPF
CD38 on IgD+ CD38dim B cell	GCST90001813	blood protein measurement	IPF
CD38 on IgD+ CD38dim B cell	GCST90001813	blood protein measurement	IPF
CD38 on IgD+ CD38dim B cell	GCST90001813	blood protein measurement	IPF
CD38 on IgD+ CD38dim B cell	GCST90001813	blood protein measurement	IPF
CD38 on IgD+ CD24- B cell	GCST90001811	blood protein measurement	IPF
CD38 on IgD+ CD24- B cell	GCST90001811	blood protein measurement	IPF
CD38 on transitional B cell	GCST90001819	blood protein measurement	IPF
CD38 on transitional B cell	GCST90001819	blood protein measurement	IPF
CD38 on transitional B cell	GCST90001819	blood protein measurement	IPF
CD38 on transitional B cell	GCST90001819	blood protein measurement	IPF
CD38 on transitional B cell	GCST90001819	blood protein measurement	IPF
CD38 on naive-mature B cell	GCST90001816	blood protein measurement	IPF
CD38 on naive-mature B cell	GCST90001816	blood protein measurement	IPF
CD38 on naive-mature B cell	GCST90001816	blood protein measurement	IPF
CD38 on IgD- CD38dim B cell	GCST90001815	blood protein measurement	IPF
CD38 on IgD- CD38dim B cell	GCST90001815	blood protein measurement	IPF
CD38 on IgD- CD38dim B cell	GCST90001815	blood protein measurement	IPF
CCR2 on CD14+ CD16+ monocyt	GCST90001992	blood protein measurement	IPF
CCR2 on CD14+ CD16+ monocyt	GCST90001992	blood protein measurement	IPF
CD16+ monocyte %monocyte	GCST90001587	monocyte count	IPF
CD16+ monocyte %monocyte	GCST90001587	monocyte count	IPF

CD14- CD16+ monocyte Absolute	GCST90001579	monocyte count	IPF
CD64 on CD14- CD16+ monocyte	GCST90001990	blood protein measurement	IPF
CD64 on CD14- CD16+ monocyte	GCST90001990	blood protein measurement	IPF
CD14+ CD16- monocyte %monoc	GCST90001586	monocyte count	IPF
CD14+ CD16- monocyte %monoc	GCST90001586	monocyte count	IPF
CD14- CD16+ monocyte %monoc	GCST90001584	monocyte count	IPF
CD14- CD16+ monocyte %monoc	GCST90001584	monocyte count	IPF
CD8dim Natural Killer T %T cell	GCST90001634	leukocyte count	IPF
CD8dim Natural Killer T %T cell	GCST90001634	leukocyte count	IPF
CD8dim Natural Killer T %T cell	GCST90001634	leukocyte count	IPF
CD8dim Natural Killer T %T cell	GCST90001634	leukocyte count	IPF
CD8dim Natural Killer T %T cell	GCST90001634	leukocyte count	IPF
CD8dim Natural Killer T %lymph	GCST90001635	leukocyte count	IPF
CD8dim Natural Killer T %lymph	GCST90001635	leukocyte count	IPF
CD8dim Natural Killer T %lymph	GCST90001635	leukocyte count	IPF
CD8dim Natural Killer T %lymph	GCST90001635	leukocyte count	IPF
CD8dim Natural Killer T %lymph	GCST90001635	leukocyte count	IPF
CD8dim Natural Killer T %lymph	GCST90001635	leukocyte count	IPF
CD25 on IgD- CD24- B cell	GCST90001785	blood protein measurement	IPF
CD25 on IgD- CD24- B cell	GCST90001785	blood protein measurement	IPF
CD4-CD8- Natural Killer T %lym	GCST90001638	leukocyte count	IPF
CD4-CD8- Natural Killer T %lym	GCST90001638	leukocyte count	IPF
CD4-CD8- Natural Killer T %lym	GCST90001638	leukocyte count	IPF
CD4-CD8- Natural Killer T %lym	GCST90001638	leukocyte count	IPF
CD4-CD8- Natural Killer T %lym	GCST90001638	leukocyte count	IPF
CD4-CD8- Natural Killer T %T ce	GCST90001637	leukocyte count	IPF
CD4-CD8- Natural Killer T %T ce	GCST90001637	leukocyte count	IPF
CD4-CD8- Natural Killer T %T ce	GCST90001637	leukocyte count	IPF
CD4-CD8- Natural Killer T %T ce	GCST90001637	leukocyte count	IPF
CD4-CD8- Natural Killer T %T ce	GCST90001637	leukocyte count	IPF
Natural Killer T %lymphocyte	GCST90001623	leukocyte count	IPF
Natural Killer T %lymphocyte	GCST90001623	leukocyte count	IPF
Natural Killer T %lymphocyte	GCST90001623	leukocyte count	IPF
Natural Killer T %lymphocyte	GCST90001623	leukocyte count	IPF
Natural Killer T %lymphocyte	GCST90001623	leukocyte count	IPF
Natural Killer T %lymphocyte	GCST90001623	leukocyte count	IPF
Natural Killer T %lymphocyte	GCST90001623	leukocyte count	IPF
CD4-CD8- Natural Killer T Absol	GCST90001636	leukocyte count	IPF
CD4-CD8- Natural Killer T Absol	GCST90001636	leukocyte count	IPF
CD4-CD8- Natural Killer T Absol	GCST90001636	leukocyte count	IPF
CD4-CD8- Natural Killer T Absol	GCST90001636	leukocyte count	IPF
Natural Killer T %T cell	GCST90001622	leukocyte count	IPF
Natural Killer T %T cell	GCST90001622	leukocyte count	IPF
Natural Killer T %T cell	GCST90001622	leukocyte count	IPF
Natural Killer T %T cell	GCST90001622	leukocyte count	IPF
Natural Killer T %T cell	GCST90001622	leukocyte count	IPF
CD8dim Natural Killer T Absolute	GCST90001633	leukocyte count	IPF
CD8dim Natural Killer T Absolute	GCST90001633	leukocyte count	IPF
CD8dim Natural Killer T Absolute	GCST90001633	leukocyte count	IPF
CD8dim Natural Killer T Absolute	GCST90001633	leukocyte count	IPF
CD8dim Natural Killer T Absolute	GCST90001633	leukocyte count	IPF
SSC-A on CD14+ monocyte	GCST90002074	blood protein measurement	IPF
SSC-A on CD14+ monocyte	GCST90002074	blood protein measurement	IPF
SSC-A on CD14+ monocyte	GCST90002074	blood protein measurement	IPF
SSC-A on CD14+ monocyte	GCST90002074	blood protein measurement	IPF
CD86+ myeloid Dendritic Cell %I	GCST90001465	leukocyte count	IPF
CD86+ myeloid Dendritic Cell %I	GCST90001465	leukocyte count	IPF
CD86+ myeloid Dendritic Cell %I	GCST90001465	leukocyte count	IPF

CD86+ myeloid Dendritic Cell %I	GCST90001465	leukocyte count	IPF
CD127 on CD45RA+ CD4+ T cell	GCST90001932	blood protein measurement	IPF
CD127 on CD45RA+ CD4+ T cell	GCST90001932	blood protein measurement	IPF
CD127 on CD45RA+ CD4+ T cell	GCST90001932	blood protein measurement	IPF
SSC-A on T cell	GCST90002080	blood protein measurement	IPF
Secreting CD4 regulatory T cell %	GCST90001494	lymphocyte count	IPF
Secreting CD4 regulatory T cell %	GCST90001494	lymphocyte count	IPF
Secreting CD4 regulatory T cell %	GCST90001494	lymphocyte count	IPF
Secreting CD4 regulatory T cell %	GCST90001494	lymphocyte count	IPF
Secreting CD4 regulatory T cell %	GCST90001494	lymphocyte count	IPF
Secreting CD4 regulatory T cell %	GCST90001494	lymphocyte count	IPF
Resting CD4 regulatory T cell %C	GCST90001482	lymphocyte count	IPF
Resting CD4 regulatory T cell %C	GCST90001482	lymphocyte count	IPF
Resting CD4 regulatory T cell %C	GCST90001482	lymphocyte count	IPF
Resting CD4 regulatory T cell %C	GCST90001482	lymphocyte count	IPF
CD4RA on Terminally Differentia	GCST90002099	blood protein measurement	IPF
CD4RA on Terminally Differentia	GCST90002099	blood protein measurement	IPF
Secreting CD4 regulatory T cell %	GCST90001493	lymphocyte count	IPF
Secreting CD4 regulatory T cell %	GCST90001493	lymphocyte count	IPF
Secreting CD4 regulatory T cell %	GCST90001493	lymphocyte count	IPF
Secreting CD4 regulatory T cell %	GCST90001493	lymphocyte count	IPF
CD28+ CD45RA- CD8+ T cell %(	GCST90001692	lymphocyte count	IPF
CD28+ CD45RA- CD8+ T cell %(	GCST90001692	lymphocyte count	IPF
CD28+ CD45RA- CD8dim T cell '	GCST90001668	lymphocyte count	IPF
Activated & resting CD4 regulator	GCST90001500	lymphocyte count	IPF
Activated & resting CD4 regulator	GCST90001500	lymphocyte count	IPF
Activated & resting CD4 regulator	GCST90001500	lymphocyte count	IPF
Activated & secreting CD4 regulat	GCST90001501	lymphocyte count	IPF
Activated & secreting CD4 regulat	GCST90001501	lymphocyte count	IPF
Activated & secreting CD4 regulat	GCST90001501	lymphocyte count	IPF
Activated & secreting CD4 regulat	GCST90001502	lymphocyte count	IPF
Activated & secreting CD4 regulat	GCST90001502	lymphocyte count	IPF
Activated & secreting CD4 regulat	GCST90001502	lymphocyte count	IPF
Activated & secreting CD4 regulat	GCST90001502	lymphocyte count	IPF
Activated & secreting CD4 regulat	GCST90001502	lymphocyte count	IPF
Activated & secreting CD4 regulat	GCST90001502	lymphocyte count	IPF
Activated & secreting CD4 regulat	GCST90001502	lymphocyte count	IPF
Activated & resting CD4 regulator	GCST90001499	lymphocyte count	IPF
Activated & resting CD4 regulator	GCST90001499	lymphocyte count	IPF
Activated & resting CD4 regulator	GCST90001499	lymphocyte count	IPF
Activated & resting CD4 regulator	GCST90001499	lymphocyte count	IPF
CD45RA on naive CD4+ T cell	GCST90002098	blood protein measurement	IPF
CD45RA on naive CD4+ T cell	GCST90002098	blood protein measurement	IPF
CD45RA on naive CD4+ T cell	GCST90002098	blood protein measurement	IPF
CD45RA on naive CD4+ T cell	GCST90002098	blood protein measurement	IPF
CD45RA on naive CD4+ T cell	GCST90002098	blood protein measurement	IPF
CD45RA on naive CD4+ T cell	GCST90002098	blood protein measurement	IPF
CD45RA on naive CD4+ T cell	GCST90002098	blood protein measurement	IPF
CD45RA on naive CD4+ T cell	GCST90002098	blood protein measurement	IPF
Resting CD4 regulatory T cell %C	GCST90001481	lymphocyte count	IPF
Resting CD4 regulatory T cell %C	GCST90001481	lymphocyte count	IPF
Resting CD4 regulatory T cell %C	GCST90001481	lymphocyte count	IPF
Resting CD4 regulatory T cell %C	GCST90001481	lymphocyte count	IPF
Resting CD4 regulatory T cell %C	GCST90001481	lymphocyte count	IPF
Resting CD4 regulatory T cell %C	GCST90001481	lymphocyte count	IPF
Resting CD4 regulatory T cell %C	GCST90001481	lymphocyte count	IPF
CD25++ CD45RA- CD4 not regul:	GCST90001512	lymphocyte count	IPF
CD25++ CD45RA- CD4 not regul:	GCST90001512	lymphocyte count	IPF

CD28+ CD45RA- CD8dim T cell .	GCST90001669	lymphocyte count	IPF
CD28+ CD45RA- CD8dim T cell .	GCST90001669	lymphocyte count	IPF
Secreting CD4 regulatory T cell A	GCST90001492	lymphocyte count	IPF
Secreting CD4 regulatory T cell A	GCST90001492	lymphocyte count	IPF
Secreting CD4 regulatory T cell A	GCST90001492	lymphocyte count	IPF
CD28+ CD45RA- CD8dim T cell '	GCST90001667	lymphocyte count	IPF
CD28+ CD45RA- CD8dim T cell '	GCST90001667	lymphocyte count	IPF
CD28+ CD45RA+ CD8dim T cell	GCST90001664	lymphocyte count	IPF
CD28+ CD45RA+ CD8dim T cell	GCST90001664	lymphocyte count	IPF
CD28+ CD45RA+ CD8dim T cell	GCST90001664	lymphocyte count	IPF
CD28+ CD45RA+ CD8dim T cell	GCST90001664	lymphocyte count	IPF
CD28+ CD45RA+ CD8dim T cell	GCST90001664	lymphocyte count	IPF
CD28+ CD45RA- CD8+ T cell Ab	GCST90001693	lymphocyte count	IPF
CD28+ CD45RA- CD8+ T cell Ab	GCST90001693	lymphocyte count	IPF
CD28+ CD45RA+ CD8dim T cell	GCST90001665	lymphocyte count	IPF
CD28+ CD45RA+ CD8dim T cell	GCST90001665	lymphocyte count	IPF
CD28+ CD45RA+ CD8dim T cell	GCST90001665	lymphocyte count	IPF
CD28+ CD45RA+ CD8dim T cell	GCST90001665	lymphocyte count	IPF
HLA DR+ Natural Killer %Natura	GCST90001649	leukocyte count	IPF
HLA DR+ Natural Killer %Natura	GCST90001649	leukocyte count	IPF
HLA DR+ Natural Killer %Natura	GCST90001649	leukocyte count	IPF
HLA DR+ Natural Killer %Natura	GCST90001649	leukocyte count	IPF
HLA DR+ Natural Killer %Natura	GCST90001649	leukocyte count	IPF
HLA DR+ Natural Killer %Natura	GCST90001649	leukocyte count	IPF
CD16 on CD14+ CD16+ monocyt	GCST90002005	blood protein measurement	IPF
CD16 on CD14+ CD16+ monocyt	GCST90002005	blood protein measurement	IPF
CD16 on CD14+ CD16+ monocyt	GCST90002005	blood protein measurement	IPF
CD16 on CD14+ CD16+ monocyt	GCST90002005	blood protein measurement	IPF
CD16 on CD14+ CD16+ monocyt	GCST90002005	blood protein measurement	IPF
SSC-A on HLA DR+ Natural Kill	GCST90002077	blood protein measurement	IPF
SSC-A on HLA DR+ Natural Kill	GCST90002077	blood protein measurement	IPF
SSC-A on HLA DR+ Natural Kill	GCST90002077	blood protein measurement	IPF
CD62L- Dendritic Cell Absolute C	GCST90001462	leukocyte count	IPF
CD62L- Dendritic Cell Absolute C	GCST90001462	leukocyte count	IPF
CD62L- Dendritic Cell Absolute C	GCST90001462	leukocyte count	IPF
CD62L- Dendritic Cell Absolute C	GCST90001462	leukocyte count	IPF
Plasmacytoid Dendritic Cell %Der	GCST90001474	leukocyte count	IPF
Plasmacytoid Dendritic Cell %Der	GCST90001474	leukocyte count	IPF
Plasmacytoid Dendritic Cell %Der	GCST90001474	leukocyte count	IPF
HLA DR+ Natural Killer Absolute	GCST90001648	leukocyte count	IPF
HLA DR+ Natural Killer Absolute	GCST90001648	leukocyte count	IPF
HLA DR+ Natural Killer Absolute	GCST90001648	leukocyte count	IPF
CD62L- CD86+ myeloid Dendritic	GCST90001472	leukocyte count	IPF
CD62L- CD86+ myeloid Dendritic	GCST90001472	leukocyte count	IPF
CD62L- CD86+ myeloid Dendritic	GCST90001472	leukocyte count	IPF
CD62L- CD86+ myeloid Dendritic	GCST90001472	leukocyte count	IPF
HLA DR+ Natural Killer %CD3-	GCST90001650	leukocyte count	IPF
HLA DR+ Natural Killer %CD3-	GCST90001650	leukocyte count	IPF
HLA DR+ Natural Killer %CD3-	GCST90001650	leukocyte count	IPF
Myeloid Dendritic Cell %Dendriti	GCST90001459	leukocyte count	IPF
Myeloid Dendritic Cell %Dendriti	GCST90001459	leukocyte count	IPF
CD86+ myeloid Dendritic Cell Ab	GCST90001464	leukocyte count	IPF
CD86+ myeloid Dendritic Cell Ab	GCST90001464	leukocyte count	IPF
CD86+ myeloid Dendritic Cell Ab	GCST90001464	leukocyte count	IPF
CD86+ myeloid Dendritic Cell Ab	GCST90001464	leukocyte count	IPF
CD62L- myeloid Dendritic Cell A	GCST90001468	leukocyte count	IPF
CD62L- myeloid Dendritic Cell A	GCST90001468	leukocyte count	IPF
CD62L- myeloid Dendritic Cell A	GCST90001468	leukocyte count	IPF

CD62L- myeloid Dendritic Cell Al	GCST90001468	leukocyte count	IPF
CD62L- myeloid Dendritic Cell Al	GCST90001468	leukocyte count	IPF
CD62L- Dendritic Cell %Dendritic	GCST90001463	leukocyte count	IPF
CD62L- Dendritic Cell %Dendritic	GCST90001463	leukocyte count	IPF
CD62L- Dendritic Cell %Dendritic	GCST90001463	leukocyte count	IPF
CD62L- Dendritic Cell %Dendritic	GCST90001463	leukocyte count	IPF
CD62L- myeloid Dendritic Cell %	GCST90001469	leukocyte count	IPF
CD62L- myeloid Dendritic Cell %	GCST90001469	leukocyte count	IPF
CD62L- myeloid Dendritic Cell %	GCST90001469	leukocyte count	IPF
CD62L- myeloid Dendritic Cell %	GCST90001469	leukocyte count	IPF
CD62L- myeloid Dendritic Cell %	GCST90001469	leukocyte count	IPF
CD16 on CD14- CD16+ monocyte	GCST90001979	blood protein measurement	IPF
CD16 on CD14- CD16+ monocyte	GCST90001979	blood protein measurement	IPF
CD16 on CD14- CD16+ monocyte	GCST90001979	blood protein measurement	IPF
CD16 on CD14- CD16+ monocyte	GCST90001979	blood protein measurement	IPF
CD16 on CD14- CD16+ monocyte	GCST90001979	blood protein measurement	IPF
CD16 on CD14- CD16+ monocyte	GCST90001979	blood protein measurement	IPF
CD62L- CD86+ myeloid Dendritic	GCST90001473	leukocyte count	IPF
CD62L- CD86+ myeloid Dendritic	GCST90001473	leukocyte count	IPF
CD62L- CD86+ myeloid Dendritic	GCST90001473	leukocyte count	IPF
CD62L- CD86+ myeloid Dendritic	GCST90001473	leukocyte count	IPF
FSC-A on HLA DR+ Natural Kille	GCST90001970	blood protein measurement	IPF
FSC-A on HLA DR+ Natural Kille	GCST90001970	blood protein measurement	IPF
FSC-A on HLA DR+ Natural Kille	GCST90001970	blood protein measurement	IPF
Myeloid Dendritic Cell Absolute C	GCST90001458	leukocyte count	IPF
Myeloid Dendritic Cell Absolute C	GCST90001458	leukocyte count	IPF
Myeloid Dendritic Cell Absolute C	GCST90001458	leukocyte count	IPF
Hematopoietic Stem Cell Absolute	GCST90001514	myeloid white cell count	IPF
Hematopoietic Stem Cell Absolute	GCST90001514	myeloid white cell count	IPF
CD25 on unswitched memory B ce	GCST90001792	blood protein measurement	IPF
CD25 on unswitched memory B ce	GCST90001792	blood protein measurement	IPF
CD25 on unswitched memory B ce	GCST90001792	blood protein measurement	IPF
CD25 on IgD+ CD24+ B cell	GCST90001778	blood protein measurement	IPF
CD25 on IgD+ CD24+ B cell	GCST90001778	blood protein measurement	IPF
CD25 on CD24+ CD27+ B cell	GCST90001777	blood protein measurement	IPF
CD25 on CD24+ CD27+ B cell	GCST90001777	blood protein measurement	IPF
CD25 on CD24+ CD27+ B cell	GCST90001777	blood protein measurement	IPF
CD25 on memory B cell	GCST90001790	blood protein measurement	IPF
CD25 on memory B cell	GCST90001790	blood protein measurement	IPF
CD25 on memory B cell	GCST90001790	blood protein measurement	IPF
HLA DR on CD33+ HLA DR+ CI	GCST90002108	blood protein measurement	IPF
HLA DR on CD33+ HLA DR+ CI	GCST90002108	blood protein measurement	IPF
HLA DR on plasmacytoid Dendrit	GCST90002105	blood protein measurement	IPF
HLA DR on plasmacytoid Dendrit	GCST90002105	blood protein measurement	IPF
HLA DR on plasmacytoid Dendrit	GCST90002105	blood protein measurement	IPF
HLA DR on plasmacytoid Dendrit	GCST90002105	blood protein measurement	IPF
HLA DR on plasmacytoid Dendrit	GCST90002105	blood protein measurement	IPF
HLA DR on myeloid Dendritic Ce	GCST90002104	blood protein measurement	IPF
HLA DR on myeloid Dendritic Ce	GCST90002104	blood protein measurement	IPF
HLA DR on myeloid Dendritic Ce	GCST90002104	blood protein measurement	IPF
HLA DR on myeloid Dendritic Ce	GCST90002104	blood protein measurement	IPF
HLA DR on myeloid Dendritic Ce	GCST90002104	blood protein measurement	IPF
HLA DR on Dendritic Cell	GCST90002106	blood protein measurement	IPF
HLA DR on Dendritic Cell	GCST90002106	blood protein measurement	IPF
HLA DR on Dendritic Cell	GCST90002106	blood protein measurement	IPF
HLA DR on Dendritic Cell	GCST90002106	blood protein measurement	IPF
HLA DR on Dendritic Cell	GCST90002106	blood protein measurement	IPF
HLA DR on Dendritic Cell	GCST90002106	blood protein measurement	IPF

CD45 on CD8+ T cell	GCST90001917	blood protein measurement	IPF
CD45 on CD8+ T cell	GCST90001917	blood protein measurement	IPF
CD4 on Effector Memory CD4+ T	GCST90002025	blood protein measurement	IPF
CD4 on Effector Memory CD4+ T	GCST90002025	blood protein measurement	IPF
CD4 on Central Memory CD4+ T	GCST90002023	blood protein measurement	IPF
CD4 on CD28+ CD4+ T cell	GCST90002062	blood protein measurement	IPF
CD4 on CD28+ CD4+ T cell	GCST90002062	blood protein measurement	IPF
CD4 on secreting CD4 regulatory	GCST90002068	blood protein measurement	IPF
CD4 on resting CD4 regulatory T	GCST90002064	blood protein measurement	IPF
CD4 on CD39+ activated CD4 reg	GCST90002067	blood protein measurement	IPF
CD4 on CD39+ activated CD4 reg	GCST90002067	blood protein measurement	IPF
CD4 on CD39+ activated CD4 reg	GCST90002067	blood protein measurement	IPF
CD4 on CD4 regulatory T cell	GCST90002063	blood protein measurement	IPF
CD4 on CD45RA+ CD4+ T cell	GCST90002027	blood protein measurement	IPF
CD4 on activated CD4 regulatory	GCST90002066	blood protein measurement	IPF
CD4 on activated & secreting CD4	GCST90002070	blood protein measurement	IPF
HLA DR+ CD4+ T cell %T cell	GCST90001625	leukocyte count	IPF
CD127 on CD28+ CD45RA- CD8	GCST90001928	blood protein measurement	IPF
CD19 on memory B cell	GCST90001736	blood protein measurement	IPF
CD19 on memory B cell	GCST90001736	blood protein measurement	IPF
CD19 on CD24+ CD27+ B cell	GCST90001723	blood protein measurement	IPF
CD19 on CD24+ CD27+ B cell	GCST90001723	blood protein measurement	IPF
CD45RA on naive CD8+ T cell	GCST90002100	blood protein measurement	IPF
CD45RA on naive CD8+ T cell	GCST90002100	blood protein measurement	IPF
Memory B cell %lymphocyte	GCST90001436	lymphocyte count	IPF
Memory B cell %lymphocyte	GCST90001436	lymphocyte count	IPF
Switched memory B cell %lympho	GCST90001434	lymphocyte count	IPF
Switched memory B cell %lympho	GCST90001434	lymphocyte count	IPF
Switched memory B cell %lympho	GCST90001434	lymphocyte count	IPF
IgD+ CD24+ B cell %lymphocyte	GCST90001439	lymphocyte count	IPF
IgD+ CD24+ B cell %lymphocyte	GCST90001439	lymphocyte count	IPF
IgD+ B cell %Lymphocyte	GCST90001424	lymphocyte count	IPF
IgD+ B cell %Lymphocyte	GCST90001424	lymphocyte count	IPF
CD24+ CD27+ B cell %lymphocy	GCST90001442	lymphocyte count	IPF
CD24+ CD27+ B cell %lymphocy	GCST90001442	lymphocyte count	IPF
CD24+ CD27+ B cell %lymphocy	GCST90001442	lymphocyte count	IPF
HLA DR on CD14- CD16+ monoc	GCST90001984	blood protein measurement	IPF
HLA DR on CD14- CD16+ monoc	GCST90001984	blood protein measurement	IPF
HLA DR on CD14- CD16+ monoc	GCST90001984	blood protein measurement	IPF
HLA DR on CD14- CD16+ monoc	GCST90001984	blood protein measurement	IPF
HLA DR on CD14- CD16+ monoc	GCST90001984	blood protein measurement	IPF
CD33 on CD33dim HLA DR-	GCST90001953	blood protein measurement	IPF
CD33 on CD33dim HLA DR-	GCST90001953	blood protein measurement	IPF
CD33 on CD33dim HLA DR-	GCST90001953	blood protein measurement	IPF
CD33 on CD33dim HLA DR-	GCST90001953	blood protein measurement	IPF
CD33 on CD33dim HLA DR-	GCST90001953	blood protein measurement	IPF
CD33 on CD33dim HLA DR-	GCST90001953	blood protein measurement	IPF
CD25++ CD45RA- CD4 not regul	GCST90001511	lymphocyte count	IPF
CD25++ CD45RA- CD4 not regul	GCST90001511	lymphocyte count	IPF
CD25++ CD45RA- CD4 not regul	GCST90001511	lymphocyte count	IPF
CD28+ CD45RA- CD8+ T cell %	GCST90001691	lymphocyte count	IPF
CD28+ CD45RA- CD8+ T cell %	GCST90001691	lymphocyte count	IPF
CD28+ CD45RA- CD8+ T cell %	GCST90001691	lymphocyte count	IPF
IgD- CD38dim B cell %lymphocyt	GCST90001426	lymphocyte count	IPF
IgD- CD38dim B cell %lymphocyt	GCST90001426	lymphocyte count	IPF
CD127 on CD28+ CD45RA+ CD8	GCST90001929	blood protein measurement	IPF
CD127 on CD28+ CD45RA+ CD8	GCST90001929	blood protein measurement	IPF
FSC-A on plasmacytoid Dendritic	GCST90001964	blood protein measurement	IPF

FSC-A on plasmacytoid Dendritic	GCST90001964	blood protein measurement	IPF
FSC-A on plasmacytoid Dendritic	GCST90001964	blood protein measurement	IPF
FSC-A on plasmacytoid Dendritic	GCST90001964	blood protein measurement	IPF
BAFF-R on unswitched memory B	GCST90001717	blood protein measurement	IPF
BAFF-R on unswitched memory B	GCST90001717	blood protein measurement	IPF
BAFF-R on unswitched memory B	GCST90001717	blood protein measurement	IPF
BAFF-R on unswitched memory B	GCST90001717	blood protein measurement	IPF
BAFF-R on unswitched memory B	GCST90001717	blood protein measurement	IPF
BAFF-R on CD24+ CD27+ B cell	GCST90001702	blood protein measurement	IPF
BAFF-R on CD24+ CD27+ B cell	GCST90001702	blood protein measurement	IPF
BAFF-R on CD24+ CD27+ B cell	GCST90001702	blood protein measurement	IPF
BAFF-R on CD24+ CD27+ B cell	GCST90001702	blood protein measurement	IPF
BAFF-R on CD24+ CD27+ B cell	GCST90001702	blood protein measurement	IPF
BAFF-R on CD24+ CD27+ B cell	GCST90001702	blood protein measurement	IPF
BAFF-R on CD24+ CD27+ B cell	GCST90001702	blood protein measurement	IPF
BAFF-R on switched memory B ce	GCST90001718	blood protein measurement	IPF
BAFF-R on switched memory B ce	GCST90001718	blood protein measurement	IPF
BAFF-R on switched memory B ce	GCST90001718	blood protein measurement	IPF
BAFF-R on switched memory B ce	GCST90001718	blood protein measurement	IPF
BAFF-R on switched memory B ce	GCST90001718	blood protein measurement	IPF
BAFF-R on switched memory B ce	GCST90001718	blood protein measurement	IPF
BAFF-R on switched memory B ce	GCST90001718	blood protein measurement	IPF
BAFF-R on switched memory B ce	GCST90001718	blood protein measurement	IPF
BAFF-R on IgD- CD38dim B cell	GCST90001714	blood protein measurement	IPF
BAFF-R on IgD- CD38dim B cell	GCST90001714	blood protein measurement	IPF
BAFF-R on IgD- CD38dim B cell	GCST90001714	blood protein measurement	IPF
BAFF-R on IgD- CD38dim B cell	GCST90001714	blood protein measurement	IPF
BAFF-R on IgD- CD38dim B cell	GCST90001714	blood protein measurement	IPF
BAFF-R on IgD- CD38dim B cell	GCST90001714	blood protein measurement	IPF
BAFF-R on memory B cell	GCST90001715	blood protein measurement	IPF
BAFF-R on memory B cell	GCST90001715	blood protein measurement	IPF
BAFF-R on memory B cell	GCST90001715	blood protein measurement	IPF
BAFF-R on memory B cell	GCST90001715	blood protein measurement	IPF
BAFF-R on memory B cell	GCST90001715	blood protein measurement	IPF
BAFF-R on IgD- CD38- B cell	GCST90001712	blood protein measurement	IPF
BAFF-R on IgD- CD38- B cell	GCST90001712	blood protein measurement	IPF
BAFF-R on IgD- CD38- B cell	GCST90001712	blood protein measurement	IPF
BAFF-R on IgD- CD38- B cell	GCST90001712	blood protein measurement	IPF
BAFF-R on IgD- CD38- B cell	GCST90001712	blood protein measurement	IPF
CD28- CD8dim T cell %T cell	GCST90001661	lymphocyte count	IPF
CD28- CD8dim T cell %T cell	GCST90001661	lymphocyte count	IPF
CD28- CD8dim T cell %T cell	GCST90001661	lymphocyte count	IPF
CD40 on CD14+ CD16- monocyte	GCST90001980	blood protein measurement	IPF
CD40 on CD14+ CD16- monocyte	GCST90001980	blood protein measurement	IPF
CD40 on CD14+ CD16- monocyte	GCST90001980	blood protein measurement	IPF
CD40 on CD14+ CD16- monocyte	GCST90001980	blood protein measurement	IPF
CD20- CD38- B cell %B cell	GCST90001422	lymphocyte count	IPF
CD19 on CD20- B cell	GCST90001721	blood protein measurement	IPF
CD20 on IgD- CD38- B cell	GCST90001755	blood protein measurement	IPF
CD20 on IgD- CD38- B cell	GCST90001755	blood protein measurement	IPF
CD20 on IgD- CD38- B cell	GCST90001755	blood protein measurement	IPF
CD20 on IgD- CD38- B cell	GCST90001755	blood protein measurement	IPF
CD28 on activated & secreting CD	GCST90001889	blood protein measurement	IPF
CD28 on CD4 regulatory T cell	GCST90001899	blood protein measurement	IPF
CD28 on CD39+ resting CD4 regu	GCST90001901	blood protein measurement	IPF
CD28 on CD39+ resting CD4 regu	GCST90001901	blood protein measurement	IPF
CD39 on granulocyte	GCST90002033	blood protein measurement	IPF
CD39 on granulocyte	GCST90002033	blood protein measurement	IPF

BAFF-R on IgD+ CD24+ B cell	GCST90001703	blood protein measurement	IPF
BAFF-R on IgD+ CD24+ B cell	GCST90001703	blood protein measurement	IPF
BAFF-R on IgD+ CD24+ B cell	GCST90001703	blood protein measurement	IPF
BAFF-R on IgD+ CD24+ B cell	GCST90001703	blood protein measurement	IPF
BAFF-R on IgD+ CD24+ B cell	GCST90001703	blood protein measurement	IPF
BAFF-R on IgD+ CD24+ B cell	GCST90001703	blood protein measurement	IPF
CD62L on CD62L+ plasmacytoid	GCST90001832	blood protein measurement	IPF
CD62L on CD62L+ plasmacytoid	GCST90001832	blood protein measurement	IPF
CD3 on Natural Killer T	GCST90001848	blood protein measurement	IPF
CD3 on Natural Killer T	GCST90001848	blood protein measurement	IPF
Effector Memory CD8+ T cell %C	GCST90001555	lymphocyte count	IPF
Effector Memory CD8+ T cell %C	GCST90001555	lymphocyte count	IPF
CD45RA+ CD8+ T cell %CD8+ T	GCST90001561	lymphocyte count	IPF
CD45RA+ CD8+ T cell %CD8+ T	GCST90001561	lymphocyte count	IPF
CD45RA+ CD8+ T cell %CD8+ T	GCST90001561	lymphocyte count	IPF
CD28+ CD45RA+ CD8+ T cell A	GCST90001690	lymphocyte count	IPF
CD28+ CD45RA+ CD8+ T cell A	GCST90001690	lymphocyte count	IPF
Natural Killer %CD3- lymphocyte	GCST90001646	leukocyte count	IPF
Natural Killer %CD3- lymphocyte	GCST90001646	leukocyte count	IPF
Natural Killer %CD3- lymphocyte	GCST90001646	leukocyte count	IPF
Natural Killer %CD3- lymphocyte	GCST90001646	leukocyte count	IPF
Natural Killer %CD3- lymphocyte	GCST90001646	leukocyte count	IPF
B cell %CD3- lymphocyte	GCST90001643	leukocyte count	IPF
B cell %CD3- lymphocyte	GCST90001643	leukocyte count	IPF
B cell %CD3- lymphocyte	GCST90001643	leukocyte count	IPF
HLA DR on CD14+ CD16- monoc	GCST90001988	blood protein measurement	IPF
HLA DR on CD14+ CD16- monoc	GCST90001988	blood protein measurement	IPF
HLA DR on CD14+ CD16- monoc	GCST90001988	blood protein measurement	IPF
HLA DR on CD14+ CD16- monoc	GCST90001988	blood protein measurement	IPF
HLA DR on CD14+ CD16- monoc	GCST90001988	blood protein measurement	IPF
HLA DR on CD14+ CD16- monoc	GCST90001988	blood protein measurement	IPF
HLA DR on CD14+ monocyte	GCST90001991	blood protein measurement	IPF
HLA DR on CD14+ monocyte	GCST90001991	blood protein measurement	IPF
HLA DR on CD14+ monocyte	GCST90001991	blood protein measurement	IPF
HLA DR on CD14+ monocyte	GCST90001991	blood protein measurement	IPF
HLA DR on CD14+ monocyte	GCST90001991	blood protein measurement	IPF
HLA DR on CD14+ monocyte	GCST90001991	blood protein measurement	IPF
Monocytic Myeloid-Derived Supp	GCST90001530	myeloid white cell count	IPF
Monocytic Myeloid-Derived Supp	GCST90001530	myeloid white cell count	IPF
Monocytic Myeloid-Derived Supp	GCST90001530	myeloid white cell count	IPF
Monocytic Myeloid-Derived Supp	GCST90001530	myeloid white cell count	IPF
Monocytic Myeloid-Derived Supp	GCST90001530	myeloid white cell count	IPF
Monocytic Myeloid-Derived Supp	GCST90001530	myeloid white cell count	IPF
HLA DR on CD14- CD16-	GCST90002009	blood protein measurement	IPF
HLA DR on CD14- CD16-	GCST90002009	blood protein measurement	IPF
HLA DR on CD14- CD16-	GCST90002009	blood protein measurement	IPF
CD62L on CD62L+ myeloid Dend	GCST90001831	blood protein measurement	IPF
CD62L on CD62L+ myeloid Dend	GCST90001831	blood protein measurement	IPF
CD3 on CD39+ CD4+ T cell	GCST90001860	blood protein measurement	IPF
CD3 on CD39+ CD4+ T cell	GCST90001860	blood protein measurement	IPF
CD3 on CD39+ CD4+ T cell	GCST90001860	blood protein measurement	IPF
CD3 on CD28+ CD45RA+ CD8+	GCST90001864	blood protein measurement	IPF
CD3 on CD28+ CD45RA+ CD8+	GCST90001864	blood protein measurement	IPF
CD3 on CD28+ CD45RA+ CD8+	GCST90001864	blood protein measurement	IPF
CD3 on CD28+ CD45RA+ CD8+	GCST90001864	blood protein measurement	IPF
CD45 on Natural Killer T	GCST90001919	blood protein measurement	IPF
CD3 on CD4+ T cell	GCST90001867	blood protein measurement	IPF
CD3 on CD4+ T cell	GCST90001867	blood protein measurement	IPF

CD3 on resting CD4 regulatory T (	GCST90001869	blood protein measurement	IPF
CD3 on resting CD4 regulatory T (	GCST90001869	blood protein measurement	IPF
CD3 on resting CD4 regulatory T (	GCST90001869	blood protein measurement	IPF
CD3 on resting CD4 regulatory T (	GCST90001869	blood protein measurement	IPF
CD3 on resting CD4 regulatory T (	GCST90001869	blood protein measurement	IPF
CD24 on transitional B cell	GCST90001774	blood protein measurement	IPF
FSC-A on CD4+ T cell	GCST90001973	blood protein measurement	IPF
FSC-A on CD4+ T cell	GCST90001973	blood protein measurement	IPF
CD20 on IgD+ B cell	GCST90001762	blood protein measurement	IPF
CD20 on IgD+ B cell	GCST90001762	blood protein measurement	IPF
CD20 on IgD+ B cell	GCST90001762	blood protein measurement	IPF
CD20 on IgD+ B cell	GCST90001762	blood protein measurement	IPF
CD20 on IgD+ B cell	GCST90001762	blood protein measurement	IPF
CD20 on B cell	GCST90001743	blood protein measurement	IPF
CD20 on B cell	GCST90001743	blood protein measurement	IPF
CD20 on B cell	GCST90001743	blood protein measurement	IPF
CD20 on B cell	GCST90001743	blood protein measurement	IPF
CD25 on IgD- CD38- B cell	GCST90001787	blood protein measurement	IPF
CD25 on IgD- CD38- B cell	GCST90001787	blood protein measurement	IPF
CD25 on IgD- CD38- B cell	GCST90001787	blood protein measurement	IPF
Monocyte Absolute Count	GCST90001583	monocyte count	IPF
CD33 on CD66b++ myeloid cell	GCST90001951	blood protein measurement	IPF
CD33 on CD66b++ myeloid cell	GCST90001951	blood protein measurement	IPF
CD33 on CD66b++ myeloid cell	GCST90001951	blood protein measurement	IPF
CD33 on CD66b++ myeloid cell	GCST90001951	blood protein measurement	IPF
CD33 on CD66b++ myeloid cell	GCST90001951	blood protein measurement	IPF
CD20 on IgD+ CD38- B cell	GCST90001748	blood protein measurement	IPF
CD20 on IgD+ CD38- B cell	GCST90001748	blood protein measurement	IPF
CD20 on IgD+ CD38- B cell	GCST90001748	blood protein measurement	IPF
CD20 on IgD+ CD38- B cell	GCST90001748	blood protein measurement	IPF
CD3 on HLA DR+ T cell	GCST90001847	blood protein measurement	IPF
CD3 on HLA DR+ T cell	GCST90001847	blood protein measurement	IPF
HLA DR on HLA DR+ Natural Ki	GCST90002117	blood protein measurement	IPF
HLA DR on HLA DR+ Natural Ki	GCST90002117	blood protein measurement	IPF
HLA DR on HLA DR+ Natural Ki	GCST90002117	blood protein measurement	IPF
HLA DR on HLA DR+ Natural Ki	GCST90002117	blood protein measurement	IPF
HLA DR on HLA DR+ Natural Ki	GCST90002117	blood protein measurement	IPF
HLA DR on HLA DR+ Natural Ki	GCST90002117	blood protein measurement	IPF
CD4+ CD8dim T cell %lymphocy	GCST90001610	leukocyte count	IPF
CD4+ CD8dim T cell %lymphocy	GCST90001610	leukocyte count	IPF
CD4+ CD8dim T cell %lymphocy	GCST90001610	leukocyte count	IPF
CD80 on granulocyte	GCST90002040	blood protein measurement	IPF
CD80 on granulocyte	GCST90002040	blood protein measurement	IPF
CD80 on granulocyte	GCST90002040	blood protein measurement	IPF
CD86 on granulocyte	GCST90001906	blood protein measurement	IPF
CD86 on granulocyte	GCST90001906	blood protein measurement	IPF
Terminally Differentiated CD4+ T	GCST90001545	lymphocyte count	IPF
Terminally Differentiated CD4+ T	GCST90001545	lymphocyte count	IPF
CD45RA- CD4+ T cell %T cell	GCST90001536	lymphocyte count	IPF
Terminally Differentiated CD4-CD	GCST90001573	lymphocyte count	IPF
CD25 on resting CD4 regulatory T	GCST90001937	blood protein measurement	IPF
CD28 on CD39+ secreting CD4 re	GCST90001888	blood protein measurement	IPF
CD28 on CD39+ secreting CD4 re	GCST90001888	blood protein measurement	IPF
B cell Absolute Count	GCST90001642	leukocyte count	IPF
B cell %lymphocyte	GCST90001644	leukocyte count	IPF
Unswitched memory B cell %lymp	GCST90001432	lymphocyte count	IPF
Unswitched memory B cell %lymp	GCST90001432	lymphocyte count	IPF
Unswitched memory B cell %lymp	GCST90001432	lymphocyte count	IPF

IgD+ CD24+ B cell Absolute Cou	GCST90001412	lymphocyte count	IPF
Memory B cell Absolute Count	GCST90001407	lymphocyte count	IPF
Unswitched memory B cell Absolu	GCST90001398	lymphocyte count	IPF
Unswitched memory B cell Absolu	GCST90001398	lymphocyte count	IPF
IgD+ CD38- B cell %lymphocyte	GCST90001431	lymphocyte count	IPF
T/B cell	GCST90001588	leukocyte count	IPF
T/B cell	GCST90001588	leukocyte count	IPF
CD24+ CD27+ B cell Absolute Co	GCST90001418	lymphocyte count	IPF
CD3 on CD45RA+ CD4+ T cell	GCST90001858	blood protein measurement	IPF
CD3 on CD45RA+ CD4+ T cell	GCST90001858	blood protein measurement	IPF
CD3 on CD45RA+ CD4+ T cell	GCST90001858	blood protein measurement	IPF
CD3 on CD45RA+ CD4+ T cell	GCST90001858	blood protein measurement	IPF
CD28- CD8dim T cell Absolute C	GCST90001663	lymphocyte count	IPF
CD28- CD8dim T cell Absolute C	GCST90001663	lymphocyte count	IPF
CD8 on CD28+ CD45RA- CD8+ T	GCST90002118	blood protein measurement	IPF
CD8 on CD28+ CD45RA- CD8+ T	GCST90002118	blood protein measurement	IPF
Terminally Differentiated CD8+ T	GCST90001558	lymphocyte count	IPF
Terminally Differentiated CD8+ T	GCST90001558	lymphocyte count	IPF
Terminally Differentiated CD8+ T	GCST90001558	lymphocyte count	IPF
Terminally Differentiated CD8+ T	GCST90001558	lymphocyte count	IPF
BAFF-R on IgD- CD27- B cell	GCST90001711	blood protein measurement	IPF
BAFF-R on IgD- CD27- B cell	GCST90001711	blood protein measurement	IPF
BAFF-R on IgD- CD27- B cell	GCST90001711	blood protein measurement	IPF
BAFF-R on IgD- CD27- B cell	GCST90001711	blood protein measurement	IPF
BAFF-R on IgD- CD27- B cell	GCST90001711	blood protein measurement	IPF
BAFF-R on IgD- CD27- B cell	GCST90001711	blood protein measurement	IPF
BAFF-R on IgD- CD27- B cell	GCST90001711	blood protein measurement	IPF
BAFF-R on IgD- CD24- B cell	GCST90001710	blood protein measurement	IPF
BAFF-R on IgD- CD24- B cell	GCST90001710	blood protein measurement	IPF
BAFF-R on IgD- CD24- B cell	GCST90001710	blood protein measurement	IPF
BAFF-R on IgD- CD24- B cell	GCST90001710	blood protein measurement	IPF
BAFF-R on IgD- CD24- B cell	GCST90001710	blood protein measurement	IPF
BAFF-R on IgD- CD24- B cell	GCST90001710	blood protein measurement	IPF
BAFF-R on IgD- CD24- B cell	GCST90001710	blood protein measurement	IPF
CD127 on CD28+ CD4+ T cell	GCST90001924	blood protein measurement	IPF
CD127 on CD28+ CD4+ T cell	GCST90001924	blood protein measurement	IPF
CD127 on CD28+ CD4+ T cell	GCST90001924	blood protein measurement	IPF
CD4+ T cell %leukocyte	GCST90001606	leukocyte count	IPF
HLA DR+ CD4+ T cell Absolute (	GCST90001624	leukocyte count	IPF
HLA DR+ CD4+ T cell Absolute (	GCST90001624	leukocyte count	IPF
CD28 on CD45RA- CD4 not regul	GCST90001891	blood protein measurement	IPF
CD28 on CD45RA- CD4 not regul	GCST90001891	blood protein measurement	IPF
CD14+ CD16- monocyte Absolute	GCST90001582	monocyte count	IPF
CD14+ CD16- monocyte Absolute	GCST90001582	monocyte count	IPF
CD14+ CD16- monocyte Absolute	GCST90001582	monocyte count	IPF
Activated CD4 regulatory T cell %	GCST90001487	lymphocyte count	IPF
Activated CD4 regulatory T cell %	GCST90001487	lymphocyte count	IPF
Activated CD4 regulatory T cell %	GCST90001487	lymphocyte count	IPF
IgD on unswitched memory B cell	GCST90001826	blood protein measurement	IPF
IgD on unswitched memory B cell	GCST90001826	blood protein measurement	IPF
IgD on unswitched memory B cell	GCST90001826	blood protein measurement	IPF
IgD on unswitched memory B cell	GCST90001826	blood protein measurement	IPF
IgD on unswitched memory B cell	GCST90001826	blood protein measurement	IPF
IgD on unswitched memory B cell	GCST90001826	blood protein measurement	IPF
CD127 on granulocyte	GCST90001926	blood protein measurement	IPF
CD127 on granulocyte	GCST90001926	blood protein measurement	IPF
CD127 on granulocyte	GCST90001926	blood protein measurement	IPF
CD127 on granulocyte	GCST90001926	blood protein measurement	IPF

CD127 on granulocyte	GCST90001926	blood protein measurement	IPF
CD127 on granulocyte	GCST90001926	blood protein measurement	IPF
CD127 on granulocyte	GCST90001926	blood protein measurement	IPF
CD28- CD4-CD8- T cell %T cell	GCST90001694	lymphocyte count	IPF
CD28- CD4-CD8- T cell %T cell	GCST90001694	lymphocyte count	IPF
CD28- CD4-CD8- T cell %T cell	GCST90001694	lymphocyte count	IPF
CD4-CD8- T cell %leukocyte	GCST90001613	leukocyte count	IPF
CD4-CD8- T cell %leukocyte	GCST90001613	leukocyte count	IPF
CD4-CD8- T cell %leukocyte	GCST90001613	leukocyte count	IPF
CD4-CD8- T cell %leukocyte	GCST90001613	leukocyte count	IPF
CD8dim T cell %T cell	GCST90001597	leukocyte count	IPF
CD8dim T cell %T cell	GCST90001597	leukocyte count	IPF
CD4-CD8- T cell %T cell	GCST90001599	leukocyte count	IPF
CD4-CD8- T cell %T cell	GCST90001599	leukocyte count	IPF
CD4-CD8- T cell %T cell	GCST90001599	leukocyte count	IPF
CD8dim T cell Absolute Count	GCST90001596	leukocyte count	IPF
CD8dim T cell Absolute Count	GCST90001596	leukocyte count	IPF
CD8dim T cell %leukocyte	GCST90001612	leukocyte count	IPF
CD8dim T cell %leukocyte	GCST90001612	leukocyte count	IPF
CD28 on CD28+ CD4-CD8- T cell	GCST90001895	blood protein measurement	IPF
CD28 on CD28+ CD4-CD8- T cell	GCST90001895	blood protein measurement	IPF
CD28 on CD28+ CD4-CD8- T cell	GCST90001895	blood protein measurement	IPF
Natural Killer T Absolute Count	GCST90001621	leukocyte count	IPF
Natural Killer T Absolute Count	GCST90001621	leukocyte count	IPF
Natural Killer T Absolute Count	GCST90001621	leukocyte count	IPF
Natural Killer T Absolute Count	GCST90001621	leukocyte count	IPF
IgD+ CD38+ B cell Absolute Cour	GCST90001392	lymphocyte count	IPF
IgD+ CD38+ B cell Absolute Cour	GCST90001392	lymphocyte count	IPF
BAFF-R on IgD+ CD38- unswitch	GCST90001707	blood protein measurement	IPF
BAFF-R on IgD+ CD38- unswitch	GCST90001707	blood protein measurement	IPF
BAFF-R on IgD+ CD38- unswitch	GCST90001707	blood protein measurement	IPF
BAFF-R on IgD+ CD38- unswitch	GCST90001707	blood protein measurement	IPF
BAFF-R on IgD+ CD38- unswitch	GCST90001707	blood protein measurement	IPF
CD34 on Hematopoietic Stem Cell	GCST90001870	blood protein measurement	IPF
CD34 on Hematopoietic Stem Cell	GCST90001870	blood protein measurement	IPF
CD34 on Hematopoietic Stem Cell	GCST90001870	blood protein measurement	IPF
CD34 on Hematopoietic Stem Cell	GCST90001870	blood protein measurement	IPF
CD80 on myeloid Dendritic Cell	GCST90002035	blood protein measurement	IPF
CD80 on myeloid Dendritic Cell	GCST90002035	blood protein measurement	IPF
CD80 on myeloid Dendritic Cell	GCST90002035	blood protein measurement	IPF
CD80 on myeloid Dendritic Cell	GCST90002035	blood protein measurement	IPF
CD80 on CD62L+ myeloid Dendri	GCST90002036	blood protein measurement	IPF
CD80 on CD62L+ myeloid Dendri	GCST90002036	blood protein measurement	IPF
CD80 on CD62L+ myeloid Dendri	GCST90002036	blood protein measurement	IPF
CD80 on monocyte	GCST90002039	blood protein measurement	IPF
CD80 on monocyte	GCST90002039	blood protein measurement	IPF
CD80 on monocyte	GCST90002039	blood protein measurement	IPF
CD80 on monocyte	GCST90002039	blood protein measurement	IPF
CD80 on monocyte	GCST90002039	blood protein measurement	IPF
CD80 on monocyte	GCST90002039	blood protein measurement	IPF
IgD+ CD38+ B cell %lymphocyte	GCST90001429	lymphocyte count	IPF
CD25++ CD8+ T cell Absolute Cc	GCST90001681	lymphocyte count	IPF
CD25++ CD8+ T cell Absolute Cc	GCST90001681	lymphocyte count	IPF
CD25++ CD8+ T cell Absolute Cc	GCST90001681	lymphocyte count	IPF
CCR2 on CD14+ CD16- monocyte	GCST90002004	blood protein measurement	IPF
CCR2 on CD14+ CD16- monocyte	GCST90002004	blood protein measurement	IPF
HLA DR on CD33+ HLA DR+ CI	GCST90002109	blood protein measurement	IPF
HLA DR on CD33+ HLA DR+ CI	GCST90002109	blood protein measurement	IPF

HLA DR on CD33+ HLA DR+ CI	GCST90002109	blood protein measurement	IPF
SSC-A on plasmacytoid Dendritic	GCST90002072	blood protein measurement	IPF
SSC-A on plasmacytoid Dendritic	GCST90002072	blood protein measurement	IPF
SSC-A on plasmacytoid Dendritic	GCST90002072	blood protein measurement	IPF
SSC-A on plasmacytoid Dendritic	GCST90002072	blood protein measurement	IPF
FSC-A on CD14+ monocyte	GCST90001967	blood protein measurement	IPF
CCR2 on plasmacytoid Dendritic (	GCST90002015	blood protein measurement	IPF
CCR2 on plasmacytoid Dendritic (	GCST90002015	blood protein measurement	IPF
CCR2 on CD62L+ plasmacytoid D	GCST90002016	blood protein measurement	IPF
CCR2 on CD62L+ plasmacytoid D	GCST90002016	blood protein measurement	IPF
CX3CR1 on CD14- CD16-	GCST90001994	blood protein measurement	IPF
CD14- CD16- Absolute Count	GCST90001581	monocyte count	IPF
CD14- CD16- Absolute Count	GCST90001581	monocyte count	IPF
HLA DR on CD14+ CD16+ mono	GCST90002007	blood protein measurement	IPF
HLA DR on CD14+ CD16+ mono	GCST90002007	blood protein measurement	IPF
HLA DR on CD14+ CD16+ mono	GCST90002007	blood protein measurement	IPF
Terminally Differentiated CD8+ T	GCST90001559	lymphocyte count	IPF
Terminally Differentiated CD8+ T	GCST90001559	lymphocyte count	IPF
FSC-A on Natural Killer T	GCST90001976	blood protein measurement	IPF
FSC-A on Natural Killer T	GCST90001976	blood protein measurement	IPF
CD25 on IgD+ B cell	GCST90001794	blood protein measurement	IPF
CD25 on IgD+ B cell	GCST90001794	blood protein measurement	IPF
CD25 on naive-mature B cell	GCST90001791	blood protein measurement	IPF
CD25 on naive-mature B cell	GCST90001791	blood protein measurement	IPF
CD25 on naive-mature B cell	GCST90001791	blood protein measurement	IPF
CD25 on naive-mature B cell	GCST90001791	blood protein measurement	IPF
CD25 on IgD+ CD24- B cell	GCST90001779	blood protein measurement	IPF
CD25 on IgD+ CD24- B cell	GCST90001779	blood protein measurement	IPF
CD25 on IgD+ CD24- B cell	GCST90001779	blood protein measurement	IPF
CD25 on IgD+ CD24- B cell	GCST90001779	blood protein measurement	IPF
CD25 on IgD+ CD24- B cell	GCST90001779	blood protein measurement	IPF
CD25 on B cell	GCST90001775	blood protein measurement	IPF
CD25 on B cell	GCST90001775	blood protein measurement	IPF
CD25 on B cell	GCST90001775	blood protein measurement	IPF
CD25 on IgD+ CD38dim B cell	GCST90001784	blood protein measurement	IPF
CD25 on IgD+ CD38dim B cell	GCST90001784	blood protein measurement	IPF
CD25 on IgD+ CD38dim B cell	GCST90001784	blood protein measurement	IPF
CD16-CD56 on HLA DR+ Natura	GCST90001885	blood protein measurement	IPF
CD16-CD56 on HLA DR+ Natura	GCST90001885	blood protein measurement	IPF
CD4 on CD39+ resting CD4 regul	GCST90002065	blood protein measurement	IPF
BAFF-R on IgD- CD38+ B cell	GCST90001713	blood protein measurement	IPF
BAFF-R on IgD- CD38+ B cell	GCST90001713	blood protein measurement	IPF
BAFF-R on IgD- CD38+ B cell	GCST90001713	blood protein measurement	IPF
CD64 on CD14- CD16-	GCST90002001	blood protein measurement	IPF
CD64 on CD14- CD16-	GCST90002001	blood protein measurement	IPF
CD64 on CD14- CD16-	GCST90002001	blood protein measurement	IPF
CD20 on IgD+ CD24+ B cell	GCST90001746	blood protein measurement	IPF
CD20 on IgD+ CD24+ B cell	GCST90001746	blood protein measurement	IPF
CD20 on memory B cell	GCST90001758	blood protein measurement	IPF
CD20 on memory B cell	GCST90001758	blood protein measurement	IPF
CD20 on memory B cell	GCST90001758	blood protein measurement	IPF
CD20 on memory B cell	GCST90001758	blood protein measurement	IPF
CD4 regulatory T cell %CD4+ T c	GCST90001478	lymphocyte count	IPF
CD4 regulatory T cell %CD4+ T c	GCST90001478	lymphocyte count	IPF
CD4 regulatory T cell %T cell	GCST90001479	lymphocyte count	IPF
CCR7 on naive CD4+ T cell	GCST90001907	blood protein measurement	IPF
CCR7 on naive CD4+ T cell	GCST90001907	blood protein measurement	IPF
CCR7 on naive CD4+ T cell	GCST90001907	blood protein measurement	IPF

CCR7 on naive CD4+ T cell	GCST90001907	blood protein measurement	IPF
CCR7 on naive CD8+ T cell	GCST90001908	blood protein measurement	IPF
CCR7 on naive CD8+ T cell	GCST90001908	blood protein measurement	IPF
CD127 on CD4+ T cell	GCST90001931	blood protein measurement	IPF
CD3 on T cell	GCST90001851	blood protein measurement	IPF
CD3 on T cell	GCST90001851	blood protein measurement	IPF
CD33- HLA DR+ Absolute Count	GCST90001523	myeloid white cell count	IPF
Granulocytic Myeloid-Derived Su	GCST90001524	myeloid white cell count	IPF
Granulocytic Myeloid-Derived Su	GCST90001524	myeloid white cell count	IPF
CD8 on Effector Memory CD8+ T	GCST90002056	blood protein measurement	IPF
CD8 on Effector Memory CD8+ T	GCST90002056	blood protein measurement	IPF
CD8 on Effector Memory CD8+ T	GCST90002056	blood protein measurement	IPF
CD28- CD127- CD25++ CD8+ T	GCST90001675	lymphocyte count	IPF
CD3 on CD39+ resting CD4 regul	GCST90001852	blood protein measurement	IPF
CD3 on CD39+ CD8+ T cell	GCST90001866	blood protein measurement	IPF
CD3 on HLA DR+ CD4+ T cell	GCST90001849	blood protein measurement	IPF
CD3 on Central Memory CD8+ T	GCST90001846	blood protein measurement	IPF
CD3 on Central Memory CD8+ T	GCST90001846	blood protein measurement	IPF
CD3 on Terminally Differentiated	GCST90001844	blood protein measurement	IPF
CD3 on CD8+ T cell	GCST90001859	blood protein measurement	IPF
CD3 on CD28+ CD45RA- CD8+ T	GCST90001863	blood protein measurement	IPF
CD3 on CD28+ CD45RA- CD8+ T	GCST90001863	blood protein measurement	IPF
CD45 on CD33- HLA DR+	GCST90002046	blood protein measurement	IPF
Effector Memory CD8+ T cell Abs	GCST90001554	lymphocyte count	IPF
Effector Memory CD8+ T cell Abs	GCST90001554	lymphocyte count	IPF
Resting CD4 regulatory T cell Abs	GCST90001480	lymphocyte count	IPF
Resting CD4 regulatory T cell Abs	GCST90001480	lymphocyte count	IPF
Resting CD4 regulatory T cell Abs	GCST90001480	lymphocyte count	IPF
Effector Memory CD8+ T cell %T	GCST90001556	lymphocyte count	IPF
CD45RA+ CD8+ T cell %T cell	GCST90001562	lymphocyte count	IPF
CD45RA+ CD8+ T cell %T cell	GCST90001562	lymphocyte count	IPF
CD45RA- CD4+ T cell Absolute C	GCST90001534	lymphocyte count	IPF
CD25++ CD45RA- CD4 not regul	GCST90001510	lymphocyte count	IPF
Effector Memory CD4+ T cell %C	GCST90001543	lymphocyte count	IPF
CD3 on Effector Memory CD8+ T	GCST90001839	blood protein measurement	IPF
CD3 on Effector Memory CD8+ T	GCST90001839	blood protein measurement	IPF
CD3 on Effector Memory CD8+ T	GCST90001839	blood protein measurement	IPF
CD3 on HLA DR+ CD8+ T cell	GCST90001850	blood protein measurement	IPF
CD3- lymphocyte Absolute Count	GCST90001639	leukocyte count	IPF
CD24 on IgD+ CD38+ B cell	GCST90001768	blood protein measurement	IPF
CD24 on IgD+ CD38+ B cell	GCST90001768	blood protein measurement	IPF
PDL-1 on CD14- CD16-	GCST90002000	blood protein measurement	IPF
HLA DR on monocyte	GCST90002010	blood protein measurement	IPF
HLA DR on monocyte	GCST90002010	blood protein measurement	IPF
HLA DR on monocyte	GCST90002010	blood protein measurement	IPF
HLA DR on monocyte	GCST90002010	blood protein measurement	IPF
HLA DR on monocyte	GCST90002010	blood protein measurement	IPF
HLA DR on monocyte	GCST90002010	blood protein measurement	IPF
CCR2 on CD14- CD16-	GCST90002003	blood protein measurement	IPF
CCR2 on CD14- CD16-	GCST90002003	blood protein measurement	IPF
CCR2 on CD14- CD16-	GCST90002003	blood protein measurement	IPF
FSC-A on monocyte	GCST90001965	blood protein measurement	IPF
FSC-A on monocyte	GCST90001965	blood protein measurement	IPF
CD33dim HLA DR+ CD11b+ %C	GCST90001526	myeloid white cell count	IPF
CD33dim HLA DR+ CD11b+ %C	GCST90001526	myeloid white cell count	IPF
CD33dim HLA DR+ CD11b+ %C	GCST90001526	myeloid white cell count	IPF
CD40 on CD14+ CD16+ monocyt	GCST90001981	blood protein measurement	IPF
CD40 on CD14+ CD16+ monocyt	GCST90001981	blood protein measurement	IPF

CD40 on CD14+ CD16+ monocyte	GCST90001981	blood protein measurement	IPF
CD40 on CD14+ CD16+ monocyte	GCST90001981	blood protein measurement	IPF
CD11c on granulocyte	GCST90002090	blood protein measurement	IPF
CD11c on granulocyte	GCST90002090	blood protein measurement	IPF
CD14 on CD33dim HLA DR+ CD	GCST90002021	blood protein measurement	IPF
CD11b on Monocytic Myeloid-De	GCST90002094	blood protein measurement	IPF
CD33 on CD33dim HLA DR+ CD	GCST90001948	blood protein measurement	IPF
CD33 on CD33dim HLA DR+ CD	GCST90001948	blood protein measurement	IPF
CD33 on CD33dim HLA DR+ CD	GCST90001948	blood protein measurement	IPF
CD33 on CD33dim HLA DR+ CD	GCST90001948	blood protein measurement	IPF
CD33 on CD33dim HLA DR+ CD	GCST90001948	blood protein measurement	IPF
CD33 on CD33dim HLA DR+ CD	GCST90001948	blood protein measurement	IPF
CD33 on CD33dim HLA DR+ CD	GCST90001948	blood protein measurement	IPF
CX3CR1 on monocyte	GCST90001995	blood protein measurement	IPF
CX3CR1 on monocyte	GCST90001995	blood protein measurement	IPF
CD33dim HLA DR+ CD11b- %CI	GCST90001528	myeloid white cell count	IPF
CD33dim HLA DR+ CD11b- %CI	GCST90001528	myeloid white cell count	IPF
CD33dim HLA DR+ CD11b- %CI	GCST90001528	myeloid white cell count	IPF
CD33dim HLA DR+ CD11b+ Abs	GCST90001525	myeloid white cell count	IPF
CD11b on CD14+ monocyte	GCST90002091	blood protein measurement	IPF
CX3CR1 on CD14- CD16+ monoc	GCST90002012	blood protein measurement	IPF
CD27 on T cell	GCST90001799	blood protein measurement	IPF
CD11b on CD33+ HLA DR+ CD1	GCST90002097	blood protein measurement	IPF
PDL-1 on CD14+ CD16+ monocy	GCST90001998	blood protein measurement	IPF
CD33 on CD33dim HLA DR+ CD	GCST90001949	blood protein measurement	IPF
CD33 on CD33dim HLA DR+ CD	GCST90001949	blood protein measurement	IPF
CD33 on CD33dim HLA DR+ CD	GCST90001949	blood protein measurement	IPF
CD33 on CD33dim HLA DR+ CD	GCST90001949	blood protein measurement	IPF
CD33 on CD33dim HLA DR+ CD	GCST90001949	blood protein measurement	IPF
CD33 on CD33dim HLA DR+ CD	GCST90001949	blood protein measurement	IPF
CX3CR1 on CD14+ CD16- monoc	GCST90001997	blood protein measurement	IPF
CX3CR1 on CD14+ CD16- monoc	GCST90001997	blood protein measurement	IPF
CX3CR1 on CD14+ CD16+ mono	GCST90001996	blood protein measurement	IPF
CX3CR1 on CD14+ CD16+ mono	GCST90001996	blood protein measurement	IPF
CD11c on monocyte	GCST90002089	blood protein measurement	IPF
CCR2 on monocyte	GCST90002017	blood protein measurement	IPF
CCR2 on monocyte	GCST90002017	blood protein measurement	IPF
CD25 on CD45RA- CD4 not regul	GCST90001933	blood protein measurement	IPF
Natural Killer Absolute Count	GCST90001645	leukocyte count	IPF
Natural Killer Absolute Count	GCST90001645	leukocyte count	IPF
Natural Killer Absolute Count	GCST90001645	leukocyte count	IPF
CD45 on Natural Killer	GCST90001911	blood protein measurement	IPF
CD45 on Natural Killer	GCST90001911	blood protein measurement	IPF
CD38 on IgD+ CD38+ B cell	GCST90001812	blood protein measurement	IPF
CD38 on IgD+ CD38+ B cell	GCST90001812	blood protein measurement	IPF
CD38 on IgD+ CD38+ B cell	GCST90001812	blood protein measurement	IPF
CD20 on IgD- CD27- B cell	GCST90001754	blood protein measurement	IPF
CD20 on IgD- CD27- B cell	GCST90001754	blood protein measurement	IPF
CD20 on IgD- CD27- B cell	GCST90001754	blood protein measurement	IPF
CD20 on IgD- CD27- B cell	GCST90001754	blood protein measurement	IPF
CD27 on IgD+ CD24+ B cell	GCST90001800	blood protein measurement	IPF
CD27 on IgD+ CD24+ B cell	GCST90001800	blood protein measurement	IPF
CD27 on IgD+ CD24+ B cell	GCST90001800	blood protein measurement	IPF
CD27 on IgD+ CD24+ B cell	GCST90001800	blood protein measurement	IPF
CD27 on IgD+ CD24+ B cell	GCST90001800	blood protein measurement	IPF
CD27 on IgD+ CD24+ B cell	GCST90001800	blood protein measurement	IPF
CD27 on IgD- CD38+ B cell	GCST90001803	blood protein measurement	IPF
CD27 on IgD- CD38+ B cell	GCST90001803	blood protein measurement	IPF

CD25 on IgD- CD27- B cell	GCST90001786	blood protein measurement	IPF
CD25 on IgD- CD27- B cell	GCST90001786	blood protein measurement	IPF
HVEM on CD4+ T cell	GCST90001875	blood protein measurement	IPF
HVEM on CD45RA- CD4+ T cell	GCST90001880	blood protein measurement	IPF
HVEM on Central Memory CD8+	GCST90001882	blood protein measurement	IPF
HVEM on Central Memory CD4+	GCST90001876	blood protein measurement	IPF
HVEM on Effector Memory CD8+	GCST90001873	blood protein measurement	IPF
HVEM on T cell	GCST90001871	blood protein measurement	IPF
CD45 on lymphocyte	GCST90002041	blood protein measurement	IPF
CD45RA on CD39+ resting CD4 r	GCST90002103	blood protein measurement	IPF
CD45RA on CD39+ resting CD4 r	GCST90002103	blood protein measurement	IPF
CD62L- plasmacytoid Dendritic C	GCST90001470	leukocyte count	IPF
CD62L- plasmacytoid Dendritic C	GCST90001470	leukocyte count	IPF
CD62L- plasmacytoid Dendritic C	GCST90001471	leukocyte count	IPF
CD62L- plasmacytoid Dendritic C	GCST90001471	leukocyte count	IPF
CD20 on naive-mature B cell	GCST90001759	blood protein measurement	IPF
CD20 on naive-mature B cell	GCST90001759	blood protein measurement	IPF
CD20 on naive-mature B cell	GCST90001759	blood protein measurement	IPF
CD20 on naive-mature B cell	GCST90001759	blood protein measurement	IPF
CD20 on naive-mature B cell	GCST90001759	blood protein measurement	IPF
CD28 on CD4+ T cell	GCST90001898	blood protein measurement	IPF
CD28 on CD4+ T cell	GCST90001898	blood protein measurement	IPF
CD28 on CD28+ CD4+ T cell	GCST90001894	blood protein measurement	IPF
CD28 on CD28+ CD4+ T cell	GCST90001894	blood protein measurement	IPF
CD45RA+ CD28- CD8+ T cell %(	GCST90001699	lymphocyte count	IPF
CD45RA+ CD28- CD8+ T cell %(	GCST90001699	lymphocyte count	IPF
CD3 on Naive CD4+ T cell	GCST90001842	blood protein measurement	IPF
CD3 on Naive CD4+ T cell	GCST90001842	blood protein measurement	IPF
CD3 on Naive CD4+ T cell	GCST90001842	blood protein measurement	IPF
IgD on IgD+ CD38+ B cell	GCST90001824	blood protein measurement	IPF
IgD on IgD+ CD38+ B cell	GCST90001824	blood protein measurement	IPF
IgD on IgD+ CD38+ B cell	GCST90001824	blood protein measurement	IPF
IgD on IgD+ CD38+ B cell	GCST90001824	blood protein measurement	IPF
IgD on IgD+ CD38- unswitched m	GCST90001823	blood protein measurement	IPF
IgD on IgD+ CD38- unswitched m	GCST90001823	blood protein measurement	IPF
CD20 on IgD+ CD24- B cell	GCST90001747	blood protein measurement	IPF
CD20 on IgD+ CD24- B cell	GCST90001747	blood protein measurement	IPF
CD20 on IgD+ CD24- B cell	GCST90001747	blood protein measurement	IPF
CD20 on IgD+ CD24- B cell	GCST90001747	blood protein measurement	IPF
CD20 on IgD+ CD38+ B cell	GCST90001751	blood protein measurement	IPF
CD20 on IgD+ CD38+ B cell	GCST90001751	blood protein measurement	IPF
CD20 on IgD+ CD38+ B cell	GCST90001751	blood protein measurement	IPF
CD20 on IgD+ CD38+ B cell	GCST90001751	blood protein measurement	IPF
CD20 on IgD+ CD38+ B cell	GCST90001751	blood protein measurement	IPF
CD45 on granulocyte	GCST90001913	blood protein measurement	IPF
CD45 on HLA DR+ CD4+ T cell	GCST90001920	blood protein measurement	IPF
CD45 on HLA DR+ CD4+ T cell	GCST90001920	blood protein measurement	IPF
Plasmacytoid Dendritic Cell Absol	GCST90001460	leukocyte count	IPF
CCR2 on myeloid Dendritic Cell	GCST90002013	blood protein measurement	IPF
CCR2 on myeloid Dendritic Cell	GCST90002013	blood protein measurement	IPF
CCR2 on myeloid Dendritic Cell	GCST90002013	blood protein measurement	IPF
HLA DR+ CD8+ T cell Absolute (	GCST90001627	leukocyte count	IPF
HLA DR+ CD8+ T cell Absolute (	GCST90001627	leukocyte count	IPF
HLA DR+ CD8+ T cell Absolute (	GCST90001627	leukocyte count	IPF
HLA DR+ T cell Absolute Count	GCST90001618	leukocyte count	IPF
HLA DR+ T cell Absolute Count	GCST90001618	leukocyte count	IPF
HLA DR+ T cell Absolute Count	GCST90001618	leukocyte count	IPF
HVEM on Effector Memory CD4+	GCST90001878	blood protein measurement	IPF

HVEM on CD8+ T cell	GCST90001881	blood protein measurement	IPF
HVEM on Terminally Differentiat	GCST90001879	blood protein measurement	IPF
HVEM on naive CD4+ T cell	GCST90001877	blood protein measurement	IPF
HVEM on naive CD8+ T cell	GCST90001872	blood protein measurement	IPF
HVEM on naive CD8+ T cell	GCST90001872	blood protein measurement	IPF
CD24 on IgD- CD38dim B cell	GCST90001770	blood protein measurement	IPF
CD24 on IgD- CD38dim B cell	GCST90001770	blood protein measurement	IPF
CD24 on IgD+ CD38- unswitched	GCST90001767	blood protein measurement	IPF
CD24 on IgD+ CD38- unswitched	GCST90001767	blood protein measurement	IPF
CD24 on memory B cell	GCST90001771	blood protein measurement	IPF
CD24 on memory B cell	GCST90001771	blood protein measurement	IPF
CD24 on IgD- CD38- B cell	GCST90001769	blood protein measurement	IPF
CD24 on IgD- CD38- B cell	GCST90001769	blood protein measurement	IPF
CD24 on unswitched memory B ce	GCST90001772	blood protein measurement	IPF
CD24 on unswitched memory B ce	GCST90001772	blood protein measurement	IPF
CD24 on IgD+ CD38- B cell	GCST90001766	blood protein measurement	IPF
CD24 on IgD+ CD38- B cell	GCST90001766	blood protein measurement	IPF
CD24 on CD24+ CD27+ B cell	GCST90001764	blood protein measurement	IPF
CD24 on CD24+ CD27+ B cell	GCST90001764	blood protein measurement	IPF
CD24 on CD24+ CD27+ B cell	GCST90001764	blood protein measurement	IPF
CD24 on switched memory B cell	GCST90001773	blood protein measurement	IPF
CD24 on switched memory B cell	GCST90001773	blood protein measurement	IPF
CD24 on IgD+ CD24+ B cell	GCST90001765	blood protein measurement	IPF
CD24 on IgD+ CD24+ B cell	GCST90001765	blood protein measurement	IPF
SSC-A on granulocyte	GCST90002078	blood protein measurement	IPF
SSC-A on granulocyte	GCST90002078	blood protein measurement	IPF
IgD- CD27- B cell Absolute Count	GCST90001401	lymphocyte count	IPF
IgD- CD27- B cell Absolute Count	GCST90001401	lymphocyte count	IPF
IgD- CD27- B cell %B cell	GCST90001399	lymphocyte count	IPF
IgD- CD27- B cell %B cell	GCST90001399	lymphocyte count	IPF
IgD- CD27- B cell %B cell	GCST90001399	lymphocyte count	IPF
CD25 on IgD+ CD38- naive B cell	GCST90001781	blood protein measurement	IPF
CD25 on IgD+ CD38- naive B cell	GCST90001781	blood protein measurement	IPF
CD25 on IgD+ CD38- naive B cell	GCST90001781	blood protein measurement	IPF
CD39 on monocyte	GCST90002034	blood protein measurement	IPF
CD39 on monocyte	GCST90002034	blood protein measurement	IPF
CD8 on Natural Killer T	GCST90002059	blood protein measurement	IPF
Immature Myeloid-Derived Suppre	GCST90001515	myeloid white cell count	IPF
Immature Myeloid-Derived Suppre	GCST90001515	myeloid white cell count	IPF
PDL-1 on CD14- CD16+ monocyt	GCST90001999	blood protein measurement	IPF
PDL-1 on CD14- CD16+ monocyt	GCST90001999	blood protein measurement	IPF
CD45 on B cell	GCST90001910	blood protein measurement	IPF
CD45 on B cell	GCST90001910	blood protein measurement	IPF
Naive CD4-CD8- T cell %T cell	GCST90001568	lymphocyte count	IPF
CD8 on HLA DR+ CD8+ T cell	GCST90002060	blood protein measurement	IPF
CD8 on HLA DR+ CD8+ T cell	GCST90002060	blood protein measurement	IPF
CD8 on Terminally Differentiated	GCST90002057	blood protein measurement	IPF
CD8 on Terminally Differentiated	GCST90002057	blood protein measurement	IPF
CD27 on IgD+ CD38- unswitched	GCST90001801	blood protein measurement	IPF
CD27 on IgD+ CD38- unswitched	GCST90001801	blood protein measurement	IPF
CD27 on IgD+ CD38- unswitched	GCST90001801	blood protein measurement	IPF
CD27 on IgD+ CD38- unswitched	GCST90001801	blood protein measurement	IPF
CD27 on IgD+ CD38- unswitched	GCST90001801	blood protein measurement	IPF
CD14 on CD14+ CD16+ monocyt	GCST90001983	blood protein measurement	IPF
CD14 on CD14+ CD16+ monocyt	GCST90001983	blood protein measurement	IPF
IgD- CD38+ B cell %B cell	GCST90001410	lymphocyte count	IPF
FSC-A on myeloid Dendritic Cell	GCST90001963	blood protein measurement	IPF
FSC-A on myeloid Dendritic Cell	GCST90001963	blood protein measurement	IPF

FSC-A on myeloid Dendritic Cell	GCST90001963	blood protein measurement	IPF
CD8 on Central Memory CD8+ T	GCST90002054	blood protein measurement	IPF
CD8 on Central Memory CD8+ T	GCST90002054	blood protein measurement	IPF
CD4+CD8+ T cell %leukocyte	GCST90001608	leukocyte count	IPF
CD4+CD8+ T cell %T cell	GCST90001595	leukocyte count	IPF
IgD on IgD+ CD24- B cell	GCST90001821	blood protein measurement	IPF
IgD on IgD+ CD24- B cell	GCST90001821	blood protein measurement	IPF
IgD on IgD+ CD24- B cell	GCST90001821	blood protein measurement	IPF
IgD on IgD+ CD24- B cell	GCST90001821	blood protein measurement	IPF
IgD on IgD+ CD24- B cell	GCST90001821	blood protein measurement	IPF
IgD on IgD+ B cell	GCST90001827	blood protein measurement	IPF
IgD on IgD+ B cell	GCST90001827	blood protein measurement	IPF
IgD on IgD+ CD38dim B cell	GCST90001825	blood protein measurement	IPF
IgD on IgD+ CD38dim B cell	GCST90001825	blood protein measurement	IPF
IgD on IgD+ CD38dim B cell	GCST90001825	blood protein measurement	IPF
IgD on IgD+ CD38dim B cell	GCST90001825	blood protein measurement	IPF
IgD on IgD+ CD38dim B cell	GCST90001825	blood protein measurement	IPF
CD3 on CD45RA- CD4+ T cell	GCST90001845	blood protein measurement	IPF
CD3 on CD45RA- CD4+ T cell	GCST90001845	blood protein measurement	IPF
CD3 on Central Memory CD4+ T	GCST90001841	blood protein measurement	IPF
CD3 on Central Memory CD4+ T	GCST90001841	blood protein measurement	IPF
CD3 on Central Memory CD4+ T	GCST90001841	blood protein measurement	IPF
CD3 on CD39+ secreting CD4 reg	GCST90001856	blood protein measurement	IPF
CD3 on CD39+ secreting CD4 reg	GCST90001856	blood protein measurement	IPF
CD3 on Terminally Differentiated	GCST90001840	blood protein measurement	IPF
CD3 on CD28+ CD4+ T cell	GCST90001861	blood protein measurement	IPF
CD3 on CD28+ CD4+ T cell	GCST90001861	blood protein measurement	IPF
CD3 on CD28- CD8+ T cell	GCST90001865	blood protein measurement	IPF
CD3 on Effector Memory CD4+ T	GCST90001843	blood protein measurement	IPF
CD8 on CD8+ T cell	GCST90002058	blood protein measurement	IPF
HLA DR+ CD4+ T cell %lymphoc	GCST90001626	leukocyte count	IPF
HLA DR+ CD4+ T cell %lymphoc	GCST90001626	leukocyte count	IPF
HLA DR+ CD8+ T cell %T cell	GCST90001628	leukocyte count	IPF
HLA DR+ CD8+ T cell %T cell	GCST90001628	leukocyte count	IPF
HLA DR+ CD8+ T cell %T cell	GCST90001628	leukocyte count	IPF
HLA DR+ T cell%lymphocyte	GCST90001620	leukocyte count	IPF
HLA DR+ T cell%lymphocyte	GCST90001620	leukocyte count	IPF
HLA DR+ T cell%lymphocyte	GCST90001620	leukocyte count	IPF
HLA DR+ T cell%lymphocyte	GCST90001620	leukocyte count	IPF
HLA DR+ CD8+ T cell %lymphoc	GCST90001629	leukocyte count	IPF
HLA DR+ CD8+ T cell %lymphoc	GCST90001629	leukocyte count	IPF
CD45 on CD4+ T cell	GCST90001916	blood protein measurement	IPF
CD28 on CD39+ CD8+ T cell	GCST90001897	blood protein measurement	IPF
CD28 on CD39+ CD8+ T cell	GCST90001897	blood protein measurement	IPF
CD28 on CD39+ activated CD4 re	GCST90001886	blood protein measurement	IPF
Naive CD4+ T cell Absolute Coun	GCST90001540	lymphocyte count	IPF
Naive CD4+ T cell Absolute Coun	GCST90001540	lymphocyte count	IPF
CD8+ T cell Absolute Count	GCST90001592	leukocyte count	IPF
CD8+ T cell %leukocyte	GCST90001607	leukocyte count	IPF
CD28- CD8+ T cell Absolute Cou	GCST90001687	lymphocyte count	IPF
CD28- CD8+ T cell Absolute Cou	GCST90001687	lymphocyte count	IPF
CD28- CD8+ T cell Absolute Cou	GCST90001687	lymphocyte count	IPF
CD127- CD8+ T cell Absolute Co	GCST90001684	lymphocyte count	IPF
Central Memory CD4+ T cell Abs	GCST90001537	lymphocyte count	IPF
T cell Absolute Count	GCST90001603	leukocyte count	IPF
T cell Absolute Count	GCST90001603	leukocyte count	IPF
CD4+ T cell Absolute Count	GCST90001590	leukocyte count	IPF
CD28 on secreting CD4 regulatory	GCST90001887	blood protein measurement	IPF

CD28 on secreting CD4 regulatory	GCST90001887	blood protein measurement	IPF
CD28 on secreting CD4 regulatory	GCST90001887	blood protein measurement	IPF
CD28+ CD4-CD8- T cell %T cell	GCST90001655	lymphocyte count	IPF
CD4+ CD8dim T cell %leukocyte	GCST90001611	leukocyte count	IPF
CD4+ CD8dim T cell %leukocyte	GCST90001611	leukocyte count	IPF
CD4+ CD8dim T cell Absolute Co	GCST90001609	leukocyte count	IPF
CD20 on IgD- CD24- B cell	GCST90001753	blood protein measurement	IPF
CD20 on IgD- CD24- B cell	GCST90001753	blood protein measurement	IPF
IgD- CD27- B cell %lymphocyte	GCST90001433	lymphocyte count	IPF
IgD- CD27- B cell %lymphocyte	GCST90001433	lymphocyte count	IPF
CCR2 on CD62L+ myeloid Dendr	GCST90002014	blood protein measurement	IPF
CCR2 on CD62L+ myeloid Dendr	GCST90002014	blood protein measurement	IPF
CCR2 on CD62L+ myeloid Dendr	GCST90002014	blood protein measurement	IPF
CD25 on IgD+ CD38- B cell	GCST90001780	blood protein measurement	IPF
CD25 on IgD+ CD38- B cell	GCST90001780	blood protein measurement	IPF
CD25 on IgD- CD38dim B cell	GCST90001789	blood protein measurement	IPF
CD25 on IgD- CD38dim B cell	GCST90001789	blood protein measurement	IPF
CD25 on IgD- CD38dim B cell	GCST90001789	blood protein measurement	IPF
CD25 on IgD+ CD38+ B cell	GCST90001783	blood protein measurement	IPF
CD25 on IgD+ CD38+ B cell	GCST90001783	blood protein measurement	IPF
CD25 on IgD+ CD38+ B cell	GCST90001783	blood protein measurement	IPF
CD14+ CD16+ monocyte Absolut	GCST90001580	monocyte count	IPF
CD14+ CD16+ monocyte Absolut	GCST90001580	monocyte count	IPF
CD14+ CD16+ monocyte %monoc	GCST90001585	monocyte count	IPF
CD14+ CD16+ monocyte %monoc	GCST90001585	monocyte count	IPF
CD86+ plasmacytoid Dendritic Ce	GCST90001467	leukocyte count	IPF
CD33 on CD33+ HLA DR+ CD14	GCST90001947	blood protein measurement	IPF
CD33 on CD33+ HLA DR+ CD14	GCST90001947	blood protein measurement	IPF
CD33 on CD33+ HLA DR+ CD14	GCST90001947	blood protein measurement	IPF
CD33 on CD33+ HLA DR+ CD14	GCST90001947	blood protein measurement	IPF
CD33+ HLA DR+ CD14dim Abso	GCST90001520	myeloid white cell count	IPF
CD33+ HLA DR+ CD14dim Abso	GCST90001520	myeloid white cell count	IPF
CD33 on CD33+ HLA DR+ CD14	GCST90001957	blood protein measurement	IPF
CD33 on CD33+ HLA DR+ CD14	GCST90001957	blood protein measurement	IPF
CD33 on CD33+ HLA DR+ CD14	GCST90001957	blood protein measurement	IPF
CD33 on CD33+ HLA DR+ CD14	GCST90001957	blood protein measurement	IPF
CD33+ HLA DR+ Absolute Count	GCST90001517	myeloid white cell count	IPF
Basophil %CD33dim HLA DR- C	GCST90001533	myeloid white cell count	IPF
Basophil %CD33dim HLA DR- C	GCST90001533	myeloid white cell count	IPF
Basophil %CD33dim HLA DR- C	GCST90001533	myeloid white cell count	IPF
CD33 on Monocytic Myeloid-Deri	GCST90001952	blood protein measurement	IPF
CD33 on Monocytic Myeloid-Deri	GCST90001952	blood protein measurement	IPF
CD33 on Monocytic Myeloid-Deri	GCST90001952	blood protein measurement	IPF
CD33+ HLA DR+ CD14- Absolut	GCST90001518	myeloid white cell count	IPF
CD33 on CD33+ HLA DR+	GCST90001956	blood protein measurement	IPF
CD33 on CD33+ HLA DR+	GCST90001956	blood protein measurement	IPF
CD33 on CD33+ HLA DR+	GCST90001956	blood protein measurement	IPF
CD33 on CD33+ HLA DR+	GCST90001956	blood protein measurement	IPF
CD33 on CD14+ monocyte	GCST90001946	blood protein measurement	IPF
CD33 on CD14+ monocyte	GCST90001946	blood protein measurement	IPF
CD33 on CD14+ monocyte	GCST90001946	blood protein measurement	IPF
CD33 on CD14+ monocyte	GCST90001946	blood protein measurement	IPF
CD33 on CD14+ monocyte	GCST90001946	blood protein measurement	IPF
CD33 on CD14+ monocyte	GCST90001946	blood protein measurement	IPF
CD62L on CD62L+ Dendritic Cell	GCST90001833	blood protein measurement	IPF
CD62L on CD62L+ Dendritic Cell	GCST90001833	blood protein measurement	IPF
CD19 on switched memory B cell	GCST90001740	blood protein measurement	IPF
CD19 on switched memory B cell	GCST90001740	blood protein measurement	IPF

CD25 on switched memory B cell	GCST90001793	blood protein measurement	IPF
CD25 on switched memory B cell	GCST90001793	blood protein measurement	IPF
CCR2 on granulocyte	GCST90002018	blood protein measurement	IPF
FSC-A on granulocyte	GCST90001966	blood protein measurement	IPF
CD4 on HLA DR+ CD4+ T cell	GCST90001959	blood protein measurement	IPF
CD4 on HLA DR+ CD4+ T cell	GCST90001959	blood protein measurement	IPF
CD11c on CD62L+ myeloid Dendı	GCST90002088	blood protein measurement	IPF
CD62L- monocyte %monocyte	GCST90001451	leukocyte count	IPF
CD62L- monocyte %monocyte	GCST90001451	leukocyte count	IPF
CD24+ CD27+ B cell %B cell	GCST90001417	lymphocyte count	IPF
CD24+ CD27+ B cell %B cell	GCST90001417	lymphocyte count	IPF
CD24+ CD27+ B cell %B cell	GCST90001417	lymphocyte count	IPF
CD27 on Plasma Blast-Plasma Cel	GCST90001807	blood protein measurement	IPF
CCR2 on monocyte	GCST90002008	blood protein measurement	IPF
CCR2 on monocyte	GCST90002008	blood protein measurement	IPF
Plasma Blast-Plasma Cell %lymph	GCST90001435	lymphocyte count	IPF
CD28- CD4-CD8- T cell %CD4-C	GCST90001653	lymphocyte count	IPF
CD3- lymphocyte %leukocyte	GCST90001641	leukocyte count	IPF
CD28- CD8+ T cell %CD8+ T cel	GCST90001686	lymphocyte count	IPF
CD28- CD8+ T cell %CD8+ T cel	GCST90001686	lymphocyte count	IPF
CD28- CD8+ T cell %CD8+ T cel	GCST90001686	lymphocyte count	IPF
CD28+ CD4-CD8- T cell %CD4-C	GCST90001656	lymphocyte count	IPF
CD28- CD8dim T cell %CD8dim	GCST90001662	lymphocyte count	IPF
Naive CD8+ T cell Absolute Coun	GCST90001551	lymphocyte count	IPF
CD62L on monocyte	GCST90001834	blood protein measurement	IPF
CD38 on CD3- CD19-	GCST90001810	blood protein measurement	IPF
Dendritic Cell Absolute Count	GCST90001461	leukocyte count	IPF
Dendritic Cell Absolute Count	GCST90001461	leukocyte count	IPF
CD11c+ CD62L- monocyte Absolu	GCST90001452	leukocyte count	IPF
CD28 on CD28+ CD45RA- CD8+	GCST90001893	blood protein measurement	IPF
CD8 on CD28- CD8+ T cell	GCST90002120	blood protein measurement	IPF
CD8 on CD28- CD8+ T cell	GCST90002120	blood protein measurement	IPF
CD8 on CD28- CD8+ T cell	GCST90002120	blood protein measurement	IPF
CD45 on HLA DR+ Natural Killer	GCST90001912	blood protein measurement	IPF
CD14 on CD14+ CD16- monocyte	GCST90001986	blood protein measurement	IPF
CD11c on myeloid Dendritic Cell	GCST90002087	blood protein measurement	IPF
CD11c on myeloid Dendritic Cell	GCST90002087	blood protein measurement	IPF
CD11c on myeloid Dendritic Cell	GCST90002087	blood protein measurement	IPF
CD11c on myeloid Dendritic Cell	GCST90002087	blood protein measurement	IPF
CD11c on myeloid Dendritic Cell	GCST90002087	blood protein measurement	IPF
Lymphocyte Absolute Count	GCST90001601	leukocyte count	IPF
Lymphocyte Absolute Count	GCST90001601	leukocyte count	IPF
Terminally Differentiated CD4-CD	GCST90001572	lymphocyte count	IPF
CD45RA on Terminally Differenti	GCST90002101	blood protein measurement	IPF
CD25 on CD4+ T cell	GCST90001960	blood protein measurement	IPF
CD25++ CD4+ T cell Absolute Co	GCST90001504	lymphocyte count	IPF
CD25++ CD8+ T cell %T cell	GCST90001679	lymphocyte count	IPF
CD25++ CD8+ T cell %T cell	GCST90001679	lymphocyte count	IPF
CD25 on CD28+ CD4+ T cell	GCST90001962	blood protein measurement	IPF
CD25 on CD28+ CD4+ T cell	GCST90001962	blood protein measurement	IPF
CD25++ CD4+ T cell %T cell	GCST90001506	lymphocyte count	IPF
CD25++ CD4+ T cell %CD4+ T c	GCST90001505	lymphocyte count	IPF
CD28 on activated CD4 regulatory	GCST90001902	blood protein measurement	IPF
CD28 on resting CD4 regulatory T	GCST90001900	blood protein measurement	IPF
CD28 on resting CD4 regulatory T	GCST90001900	blood protein measurement	IPF
CD28 on CD45RA+ CD4+ T cell	GCST90001890	blood protein measurement	IPF
CD28 on CD45RA+ CD4+ T cell	GCST90001890	blood protein measurement	IPF
CD45 on CD33dim HLA DR-	GCST90002050	blood protein measurement	IPF

HLA DR++ monocyte %monocyte	GCST90001475	leukocyte count	IPF
HLA DR++ monocyte %monocyte	GCST90001475	leukocyte count	IPF
HLA DR++ monocyte %monocyte	GCST90001475	leukocyte count	IPF
HLA DR++ monocyte %monocyte	GCST90001475	leukocyte count	IPF
HLA DR++ monocyte %monocyte	GCST90001475	leukocyte count	IPF
CD123 on CD62L+ plasmacytoid I	GCST90001945	blood protein measurement	IPF
CD123 on CD62L+ plasmacytoid I	GCST90001945	blood protein measurement	IPF
CD80 on CD62L+ plasmacytoid D	GCST90002038	blood protein measurement	IPF
CD80 on CD62L+ plasmacytoid D	GCST90002038	blood protein measurement	IPF
CD80 on plasmacytoid Dendritic C	GCST90002037	blood protein measurement	IPF
CD80 on plasmacytoid Dendritic C	GCST90002037	blood protein measurement	IPF
CD123 on plasmacytoid Dendritic	GCST90001944	blood protein measurement	IPF
CD123 on plasmacytoid Dendritic	GCST90001944	blood protein measurement	IPF
CD25 on transitional B cell	GCST90001795	blood protein measurement	IPF
CD25 on transitional B cell	GCST90001795	blood protein measurement	IPF
CD14 on Monocytic Myeloid-Deri	GCST90002019	blood protein measurement	IPF
CD25 on CD39+ activated CD4 re	GCST90001940	blood protein measurement	IPF
CD25 on CD39+ CD4 regulatory T	GCST90001935	blood protein measurement	IPF
CD25 on CD39+ resting CD4 regu	GCST90001938	blood protein measurement	IPF
Terminally Differentiated CD8+ T	GCST90001557	lymphocyte count	IPF
CD28- CD4-CD8- T cell Absolute	GCST90001654	lymphocyte count	IPF
CD28- CD4-CD8- T cell Absolute	GCST90001654	lymphocyte count	IPF
Central Memory CD4+ T cell %CI	GCST90001538	lymphocyte count	IPF
Activated & resting CD4 regulator	GCST90001498	lymphocyte count	IPF
CD4-CD8- T cell Absolute Count	GCST90001598	leukocyte count	IPF
CD4-CD8- T cell Absolute Count	GCST90001598	leukocyte count	IPF
CD33dim HLA DR+ CD11b- Abso	GCST90001527	myeloid white cell count	IPF
CD11c+ HLA DR++ monocyte %i	GCST90001457	leukocyte count	IPF
IgD on IgD+ CD38- B cell	GCST90001822	blood protein measurement	IPF
CD86 on myeloid Dendritic Cell	GCST90001903	blood protein measurement	IPF
CD19 on IgD- CD38- B cell	GCST90001733	blood protein measurement	IPF
CD33+ HLA DR+ CD14- %CD33	GCST90001519	myeloid white cell count	IPF
CD33 on Granulocytic Myeloid-De	GCST90001950	blood protein measurement	IPF
CD33 on Granulocytic Myeloid-De	GCST90001950	blood protein measurement	IPF
CD33 on Granulocytic Myeloid-De	GCST90001950	blood protein measurement	IPF
Memory B cell %B cell	GCST90001406	lymphocyte count	IPF
Naive-mature B cell %B cell	GCST90001408	lymphocyte count	IPF
CD45 on CD33+ HLA DR+ CD14	GCST90002042	blood protein measurement	IPF
CD25++ CD8+ T cell %CD8+ T c	GCST90001680	lymphocyte count	IPF
CD4 on monocyte	GCST90001958	blood protein measurement	IPF
BAFF-R on CD20- CD38- B cell	GCST90001701	blood protein measurement	IPF
Unswitched memory B cell %B ce	GCST90001397	lymphocyte count	IPF
CD8+ T cell %T cell	GCST90001593	leukocyte count	IPF
CD4+/CD8+ T cell	GCST90001589	leukocyte count	IPF
CD28- CD8+ T cell %T cell	GCST90001685	lymphocyte count	IPF
CD19 on naive-mature B cell	GCST90001737	blood protein measurement	IPF
CD19 on naive-mature B cell	GCST90001737	blood protein measurement	IPF
IgD+ CD24- B cell %B cell	GCST90001415	lymphocyte count	IPF
CD11b on basophil	GCST90002096	blood protein measurement	IPF
CD11b on CD33dim HLA DR-	GCST90002095	blood protein measurement	IPF
CD38 on IgD+ B cell	GCST90001818	blood protein measurement	IPF
CD8+ Natural Killer T %T cell	GCST90001631	leukocyte count	IPF
Lymphocyte %leukocyte	GCST90001602	leukocyte count	IPF
Granulocyte %leukocyte	GCST90001652	leukocyte count	IPF
FSC-A on CD8+ T cell	GCST90001974	blood protein measurement	IPF
PDL-1 on CD14+ CD16- monocyt	GCST90001993	blood protein measurement	IPF
CD127 on CD8+ T cell	GCST90001927	blood protein measurement	IPF
CD8+ Natural Killer T %lymphoc	GCST90001632	leukocyte count	IPF

CD8+ Natural Killer T Absolute C	GCST90001630	leukocyte count	IPF
CD8+ Natural Killer T Absolute C	GCST90001630	leukocyte count	IPF
CD20 on transitional B cell	GCST90001763	blood protein measurement	IPF
HLA DR on Hematopoietic Stem (	GCST90002107	blood protein measurement	IPF
HLA DR on Hematopoietic Stem (	GCST90002107	blood protein measurement	IPF
HLA DR on Hematopoietic Stem (	GCST90002107	blood protein measurement	IPF
SSC-A on lymphocyte	GCST90002079	blood protein measurement	IPF
CD28- CD25++ CD8+ T cell Abso	GCST90001678	lymphocyte count	IPF
HLA DR++ monocyte %leukocyte	GCST90001476	leukocyte count	IPF
HLA DR++ monocyte %leukocyte	GCST90001476	leukocyte count	IPF
HLA DR++ monocyte %leukocyte	GCST90001476	leukocyte count	IPF
HLA DR++ monocyte Absolute C	GCST90001477	leukocyte count	IPF
CD33- HLA DR- Absolute Count	GCST90001522	myeloid white cell count	IPF

SNP	Chr	Position	EA.exposure	NEA.exposure
rs1006368	10	126346603	T	C
rs1006368	10	126346603	T	C
rs1091954З	1	161508617	G	A
rs9912354	17	33805730	T	C
rs1007337	22	39781585	T	C
rs2066399	6	15880540	A	G
rs3448676ʻ	19	49915911	A	G
rs4939384	11	60273642	G	A
rs72836542	6	14091682	T	C
rs7404890	16	11007193	A	G
rs9270560	6	32560741	C	T
rs1012252З	9	99511751	A	G
rs10400782	14	35130401	C	A
rs1048406ε	14	99653123	T	C
rs1050573ς	12	10043518	C	A
rs1082415ε	10	53915868	T	G
rs1106023ϵ	12	129773605	G	A
rs1106305	11	12065087	T	C
rs1111276(	12	106241673	T	C
rs11139981	7	146550168	T	G
rs11164874	13	28485345	A	G
rs1117459ϵ	3	67860216	C	T
rs11186737	6	159962229	A	G
rs11190247	3	14459099	G	A
rs11202534	10	89500755	T	G
rs11212152	12	119037578	C	T
rs1121377ϵ	2	41540149	C	T
rs1125177(	10	91013835	A	G
rs11252942	12	129340399	T	C
rs11260481	19	46587710	T	C
rs1127857З	2	127831527	A	G
rs1128804З	6	130091902	A	G
rs11297384	5	128365521	G	A
rs11336991	20	21420694	T	C
rs11369881	1	207678128	G	A
rs1136991ϵ	14	58571589	A	G
rs1142301ʻ	1	102796337	A	G
rs1144255ϵ	5	176911575	T	C
rs1145079ς	1	21892266	A	G
rs11461981	4	71875684	G	A
rs11468587	4	138349095	A	G
rs1147915(	1	81668305	T	C
rs1148711ϵ	5	24165437	G	T
rs11504034	3	64543612	T	C
rs1151352ς	3	105859785	A	G
rs11517541	1	100459655	A	G
rs1152127ε	3	74527776	T	C
rs11522194	7	56724250	G	T
rs1153869ʻ	20	50004560	A	G
rs11551731	2	170436614	C	T
rs11566042	3	195531004	A	C
rs11567701	4	167555345	T	C
rs11575547	5	168914162	T	C
rs1157656З	1	6118693	T	C
rs1158213З	3	115341107	G	A
rs1158791ε	3	194347376	G	A

rs11594572	2	199934892	A	G
rs11639975	4	176754667	A	G
rs11643133	6	66416572	C	T
rs11649878	4	183314518	A	G
rs11651015	1	195636177	C	A
rs11658171	5	29413612	C	T
rs11661882	18	70504468	A	G
rs11671916	3	130201546	T	G
rs11691659	11	121746998	G	A
rs11692183	7	153895185	T	C
rs11694097	18	38279589	T	C
rs11700371	10	124406240	A	G
rs11707903	7	69510130	C	T
rs11712771	8	31315428	A	G
rs11717725	13	77081029	T	C
rs11718509	6	119514715	T	C
rs11721138	15	51649599	A	G
rs11724808	6	119632972	T	C
rs11727036	11	60694375	C	A
rs11729336	12	52437438	T	C
rs11743309	7	6934892	A	G
rs11744086	8	98513319	C	T
rs11744826	8	82400020	C	T
rs11746991	7	79791344	A	C
rs11755542	16	13128176	C	A
rs11761343	12	93540934	A	G
rs11770299	9	83435830	G	A
rs11774582	18	3305228	C	T
rs11778562	12	132283582	T	G
rs11780145	15	29421113	T	C
rs11780804	8	73079210	A	G
rs11782202	6	86346609	C	T
rs11784014	22	36506460	A	C
rs11792257	21	27289871	T	C
rs11792293	17	30028367	G	A
rs11798326	8	124174485	A	C
rs11801453	11	3830358	A	G
rs11807604	8	144562245	A	G
rs11808986	19	24477916	T	C
rs11818047	20	37027035	C	T
rs12130486	1	81455274	C	T
rs12279408	11	84269972	T	C
rs12332976	6	40616989	A	G
rs12378893	9	79057643	A	G
rs12499962	4	99029457	G	A
rs12501785	4	180565400	A	G
rs12616335	2	159969605	T	C
rs12750174	1	184765000	A	G
rs13010105	2	42107980	G	A
rs13104493	4	32690871	G	A
rs132751	22	36610967	T	C
rs13794293	20	33104122	T	G
rs13794319	12	10963475	A	C
rs13799728	7	115706613	C	T
rs13815289	2	40546780	G	A
rs13824639	20	54228961	A	G
rs13825956	11	76470121	A	G
rs13832778	6	1730982	A	G

rs13840105	3	166256176	A	G
rs13844903	7	29025284	T	C
rs13856797	2	8152821	A	G
rs13882236	1	5236853	A	G
rs13887189	3	162179186	C	T
rs13893792	16	51095561	T	C
rs13894362	4	114293931	A	G
rs13894860	10	132596864	A	G
rs13895449	4	32184694	C	T
rs13907563	12	102082782	G	A
rs13942364	1	236238830	A	G
rs13958447	8	57056704	A	G
rs13973637	17	67625638	T	C
rs14008754	6	168136303	T	C
rs14017352	2	195788562	A	C
rs14032206	10	1765793	A	G
rs14036749	4	131786765	C	T
rs14080034	19	50001170	T	C
rs14092958	2	169297185	T	C
rs14096328	1	24640467	T	C
rs14164406	1	220781210	A	G
rs14207642	16	5628078	A	G
rs14215188	16	1071217	A	C
rs14260404	2	87503702	C	A
rs14268668	12	29095512	C	T
rs14286949	11	87291175	T	C
rs14292219	5	145713781	C	A
rs14339313	17	30967385	T	C
rs14339550	10	69536834	T	C
rs14343513	12	129545747	A	G
rs14377290	10	101494118	T	C
rs14387129	19	1191667	G	A
rs14394079	1	52644883	A	G
rs14410465	17	5023557	A	G
rs14420685	9	77966779	T	G
rs14425817	8	2859400	G	A
rs14427594	14	34065832	G	T
rs14461337	5	81311763	C	T
rs14468244	12	53402638	A	G
rs14494283	1	111842326	T	C
rs14502176	10	22411454	A	G
rs14533640	6	78745175	T	C
rs14556351	9	106801353	G	A
rs14558353	1	66490103	C	T
rs14572028	13	43404037	C	T
rs14574366	10	16995517	C	T
rs14583573	10	66659421	G	T
rs14597810	18	7839215	A	C
rs14610142	19	6753000	A	C
rs14613572	5	154015729	T	C
rs14621703	2	41343706	C	T
rs14631422	5	141469416	C	A
rs14637448	4	85578601	A	G
rs14646185	22	23709958	A	G
rs14665086	19	22454852	A	G
rs14666120	12	100494123	T	C
rs14675636	3	76660068	T	C
rs14693704	14	46740502	G	T

rs14702694	4	100588891	T	C
rs14756362	10	5377215	T	C
rs14757273	19	49416171	T	C
rs14759182	1	47595103	G	A
rs14768196	7	37590076	T	C
rs14795938	8	124197307	A	G
rs14796874	10	54704473	G	A
rs14838008	9	28759554	T	C
rs14851382	20	61268143	T	C
rs14859091	4	187560601	A	G
rs14865840	3	153311399	T	C
rs14881693	5	71107850	G	A
rs14883508	9	10278984	T	C
rs14893950	5	12480500	G	A
rs14894748	2	10326418	T	C
rs14920883	6	10017676	C	A
rs14943807	15	30234373	G	A
rs14964997	9	10889621	T	C
rs14980115	6	162457335	G	A
rs14982855	6	84898123	T	C
rs14984427	4	87144762	A	G
rs14986020	10	31026572	T	C
rs14987615	2	24167677	C	T
rs14990831	15	54344919	T	C
rs15005362	17	80299376	T	C
rs15005548	14	55295865	A	G
rs15040023	7	136855654	T	C
rs15047640	13	113289664	A	G
rs1508064	2	72979172	T	C
rs15088938	14	100822892	C	T
rs15090574	18	70586990	A	G
rs15099408	18	56708792	T	C
rs15108617	1	223544915	A	G
rs15108617	1	223544915	A	G
rs15124681	16	23373955	A	G
rs15125036	2	240644273	T	C
rs16864501	3	152385976	A	G
rs17642570	5	148699825	C	T
rs17791728	20	17556587	C	T
rs17878699	3	3132754	A	G
rs17882143	17	41836082	T	C
rs18071679	2	150956800	C	A
rs18074590	6	20965785	C	A
rs18097248	20	24457030	A	G
rs18122822	15	84950824	A	G
rs18166007	8	117040660	T	C
rs18328062	8	30627573	A	G
rs18389538	2	22576943	T	C
rs18418452	14	45055780	T	G
rs18440113	7	83368658	T	C
rs18491002	12	117089403	A	G
rs18503442	3	90216749	A	C
rs18558735	3	117384981	A	G
rs18575492	7	62917010	G	A
rs18625157	7	13558580	C	A
rs18799073	3	144835290	G	A
rs18807411	12	19311236	A	G
rs18808767	15	29114742	A	G

rs18829270	2	162960872	T	C
rs18837624	9	102390885	T	C
rs18913134	6	167014418	T	C
rs18936911	5	142967016	T	C
rs18941234	5	75564943	T	C
rs18979612	11	109281106	T	C
rs19066519	2	106438361	G	A
rs19132581	10	120573449	C	A
rs19160721	4	123414413	A	C
rs19239706	6	81984275	T	C
rs19255600	5	135639453	A	G
rs20019880	19	46387941	A	G
rs2384564	10	36442592	G	A
rs2496150	6	13374132	G	A
rs34000508	8	25574426	T	C
rs34039708	8	12500832	A	C
rs34539402	17	39959627	A	G
rs34609592	9	136915695	A	G
rs34772471	1	112269677	T	C
rs35275693	12	88561479	G	A
rs35706199	7	131191874	T	C
rs35730839	7	109248384	T	C
rs35948774	2	118664032	A	G
rs37496461	9	504491	C	A
rs3848018	13	99034552	G	A
rs459056	20	15614140	G	T
rs4853078	2	75148081	G	T
rs53643408	18	63832285	T	C
rs54855325	13	114441234	C	T
rs55787444	3	185766414	T	C
rs55856613	17	79094162	A	G
rs57121931	19	55017690	C	T
rs58243030	5	180495180	G	A
rs58817739	12	46009960	G	A
rs59745713	19	34075835	A	G
rs61755651	14	72431599	T	C
rs61784602	1	46796909	C	T
rs62247421	3	64390467	A	G
rs62321187	4	120605817	T	C
rs62337106	5	1957403	G	T
rs62424171	6	118769058	A	G
rs6517382	21	38156170	C	T
rs6916896	6	5734886	T	C
rs71419987	14	34919717	A	G
rs71466029	12	82065434	T	C
rs7254864	19	9246656	A	G
rs72704856	9	10774031	G	A
rs72723889	4	146773005	C	T
rs72768736	9	137149408	A	G
rs72810820	10	92600913	T	C
rs72931475	2	159316835	G	A
rs72996438	2	211172214	C	T
rs73034498	19	44937435	T	C
rs73116391	7	46417626	G	T
rs73206236	7	113679246	A	G
rs73780868	6	152137377	C	T
rs7426047	2	107184424	T	C
rs74322543	10	85916001	G	A

rs74336249	9	135625608	T	C
rs74540476	11	29902098	A	G
rs74565504	14	89207147	T	G
rs74842782	7	131413341	G	T
rs74932477	2	229222112	A	G
rs75157411	11	95180478	A	G
rs75255396	1	35892939	C	T
rs75361870	8	9202609	A	G
rs75391939	12	133446245	T	C
rs75438005	18	26017285	A	G
rs75510626	6	64401131	C	T
rs75550713	13	112088993	T	C
rs75551427	3	168644376	G	A
rs75561723	2	199542954	T	C
rs75592465	13	105831838	T	G
rs75600477	20	40197928	G	T
rs75641434	1	181280885	T	C
rs75660425	3	129232254	A	G
rs75669615	15	39668760	A	G
rs75760272	5	10850589	A	G
rs75774401	12	48211641	A	C
rs7597139	2	107560107	A	G
rs7613821	3	105611213	G	A
rs76385522	11	10620991	T	C
rs76444609	1	249084997	A	G
rs76543451	9	122201008	A	G
rs76552842	11	83433494	A	G
rs76568972	2	1592272	T	C
rs76636736	1	99542806	A	C
rs76679968	4	129754843	T	C
rs76736944	1	180881632	C	T
rs76808079	18	22735939	C	A
rs77009235	8	4499026	T	C
rs77146551	4	108096534	G	A
rs77156603	2	104590904	A	C
rs77370289	8	57917248	C	T
rs77390603	10	4688192	A	C
rs77434296	4	22140311	A	G
rs77465871	7	47504039	G	A
rs77485746	11	129187484	A	C
rs77538429	1	110742985	G	A
rs77577050	12	116077199	T	G
rs77587134	5	3635021	A	G
rs77689186	4	176614955	A	C
rs77725246	17	55639212	T	C
rs77733172	9	140235501	T	C
rs77814979	4	131041099	C	A
rs77955766	19	17307102	G	T
rs78085370	1	186023973	A	G
rs78085398	11	33121166	A	G
rs78100215	17	8298873	A	G
rs78275221	6	90900544	A	G
rs78313301	3	11075184	T	C
rs78470553	7	158334355	A	G
rs7850783	9	100968226	G	A
rs78524989	11	94284134	G	T
rs78622624	10	33359425	C	T
rs78656354	1	166905650	A	G

rs7870363€	19	33953595	C	T
rs7874114€	3	182409985	C	T
rs7875013€	14	26308944	C	T
rs78773852	12	71181930	C	T
rs78881177	11	127881478	C	A
rs78888262	16	88701572	A	C
rs7892211€	11	25534610	T	C
rs79027575	2	146371302	C	T
rs79395509	4	176348299	T	C
rs79418578	3	141886501	A	C
rs79518488	6	28389143	C	T
rs79548047	16	11614838	C	T
rs79647440	7	142438302	T	C
rs79725470	5	94705605	C	T
rs7974138€	11	109660227	A	G
rs79757812	17	65896989	A	G
rs79851411	8	13716390	A	G
rs79888370	4	180201786	T	G
rs80018878	7	46985321	T	C
rs80305889	7	34681601	G	T
rs80331575	4	109566866	T	C
rs80334718	14	33998519	G	A
rs8178689	8	42060072	G	A
rs9471016	6	39196392	T	C
rs9479093	6	151992984	G	A
rs10183338	2	111610816	T	G
rs11656270	6	31360602	G	A
rs14380923	2	167484735	G	T
rs10205992	2	87279109	T	C
rs1264570	6	30365210	T	C
rs12660712	6	29734634	A	C
rs130065	6	31122500	A	G
rs1302443€	2	86534521	A	G
rs1356625	2	86022747	C	T
rs6903896	6	31168029	A	G
rs938487	2	87009488	G	A
rs1037633	3	23750521	G	T
rs200474	6	27767640	A	G
rs2018404	14	105695446	T	C
rs709589	14	106306841	T	C
rs10422871	19	8789227	T	C
rs10765790	11	95852734	C	T
rs10426452	19	49907690	A	G
rs10501383	11	60164527	T	C
rs11751198	6	31753526	A	G
rs1569590	6	15898884	G	A
rs35039517	10	94522438	A	G
rs55809481	19	49872521	C	T
rs61731791	19	50940778	T	C
rs9271146	6	32577282	C	A
rs10505739	12	10043518	C	A
rs10824158	10	53915868	T	G
rs1117474€	3	74016318	A	G
rs11423015	1	102796337	A	G
rs11458199	2	235962017	G	A
rs11639975	4	176754667	A	G
rs11658171	5	29413612	C	T
rs11691502	18	62442252	T	C

rs11691659	11	121746998	G	A
rs11717320	11	24544063	A	G
rs11747841	8	56081028	T	C
rs11769289	7	39587008	T	G
rs11786758	8	15699657	A	G
rs11808986	19	24477916	T	C
rs12279408	11	84269972	T	C
rs12499962	4	99029457	G	A
rs12501785	4	180565400	A	G
rs13794293	20	33104122	T	G
rs13832778	6	1730982	A	G
rs13893792	16	51095561	T	C
rs14096328	1	24640467	T	C
rs14339550	10	69536834	T	C
rs14709366	15	81422902	T	G
rs14865840	3	153311399	T	C
rs14920883	6	10017676	C	A
rs15088524	17	49002799	A	G
rs16864501	3	152385976	A	G
rs17791728	20	17556587	C	T
rs18097248	20	24457030	A	G
rs18558735	3	117384981	A	G
rs18575492	7	62917010	G	A
rs18979612	11	109281106	T	C
rs19066519	2	106438361	G	A
rs34039708	8	12500832	A	C
rs35068665	14	101798387	G	T
rs35772393	16	47727514	T	C
rs37535689	6	165166578	G	A
rs4853078	2	75148081	G	T
rs62321187	4	120605817	T	C
rs72758450	15	69811049	C	T
rs72931475	2	159316835	G	A
rs75438005	18	26017285	A	G
rs75993384	4	55305871	C	T
rs76444609	1	249084997	A	G
rs76552842	11	83433494	A	G
rs77113660	12	83249816	G	A
rs77465871	7	47504039	G	A
rs77467380	16	78662086	T	C
rs78088167	4	130098084	G	A
rs78322254	12	48207497	A	G
rs78750136	14	26308944	C	T
rs79027575	2	146371302	C	T
rs79395509	4	176348299	T	C
rs79518488	6	28389143	C	T
rs79647440	7	142438302	T	C
rs79851411	8	13716390	A	G
rs80331554	15	59013643	G	A
rs10512469	17	33770802	T	C
rs9916257	17	33797371	T	G
rs10512469	17	33770802	T	C
rs18305332	1	25076059	T	C
rs9916257	17	33797371	T	G
rs10512469	17	33770802	T	C
rs9916257	17	33797371	T	G
rs1064086	2	237034061	A	G
rs10774625	12	111910219	G	A

rs11615628	12	6903631	A	G
rs10786199	10	97069472	A	G
rs10882701	10	97741071	C	A
rs12268044	10	98794615	T	C
rs14101809	10	98169221	T	G
rs5014871	10	97522795	G	A
rs72809762	10	97294154	T	C
rs10786199	10	97069472	A	G
rs10882701	10	97741071	C	A
rs12268044	10	98794615	T	C
rs6584022	10	97509641	G	T
rs72809762	10	97294154	T	C
rs7918541	10	98168559	G	A
rs10786199	10	97069472	A	G
rs10882701	10	97741071	C	A
rs11188757	10	98205222	C	A
rs7088584	10	97607644	T	G
rs72809762	10	97294154	T	C
rs10786199	10	97069472	A	G
rs10882701	10	97741071	C	A
rs12268044	10	98794615	T	C
rs14101809	10	98169221	T	G
rs7094484	10	97490998	G	A
rs72809762	10	97294154	T	C
rs10786281	10	98116936	C	T
rs11745883	10	96773382	A	G
rs12778618	10	95633120	C	T
rs4293064	10	107757380	A	C
rs7086986	10	97507312	G	A
rs1081003	22	42525756	A	G
rs11770511	22	41957510	A	G
rs14980658	22	40774205	G	A
rs1894588	22	40629980	T	C
rs2018340	22	43390319	A	G
rs5757678	22	39843409	T	C
rs76146864	22	40246055	T	C
rs1081003	22	42525756	A	G
rs11770511	22	41957510	A	G
rs14980658	22	40774205	G	A
rs1894588	22	40629980	T	C
rs5757678	22	39843409	T	C
rs5759079	22	43393437	A	G
rs76146864	22	40246055	T	C
rs1081003	22	42525756	A	G
rs11770511	22	41957510	A	G
rs14980658	22	40774205	G	A
rs1801274	1	161479745	G	A
rs1894588	22	40629980	T	C
rs5757678	22	39843409	T	C
rs5759079	22	43393437	A	G
rs76146864	22	40246055	T	C
rs1081003	22	42525756	A	G
rs11770511	22	41957510	A	G
rs14980658	22	40774205	G	A
rs1801274	1	161479745	G	A
rs1894588	22	40629980	T	C
rs5757678	22	39843409	T	C
rs5759079	22	43393437	A	G

rs76146864	22	40246055	T	C
rs1081003	22	42525756	A	G
rs11770511	22	41957510	A	G
rs14488623	22	42326166	C	T
rs14980658	22	40774205	G	A
rs1801274	1	161479745	G	A
rs1894588	22	40629980	T	C
rs5757678	22	39843409	T	C
rs5759079	22	43393437	A	G
rs76146864	22	40246055	T	C
rs1081003	22	42525756	A	G
rs11770511	22	41957510	A	G
rs14980658	22	40774205	G	A
rs1801274	1	161479745	G	A
rs1894588	22	40629980	T	C
rs5757678	22	39843409	T	C
rs5759079	22	43393437	A	G
rs76146864	22	40246055	T	C
rs1081003	22	42525756	A	G
rs11770511	22	41957510	A	G
rs14488623	22	42326166	C	T
rs14980658	22	40774205	G	A
rs1801274	1	161479745	G	A
rs1894588	22	40629980	T	C
rs5757678	22	39843409	T	C
rs5759079	22	43393437	A	G
rs76146864	22	40246055	T	C
rs1081003	22	42525756	A	G
rs11770511	22	41957510	A	G
rs14980658	22	40774205	G	A
rs1894588	22	40629980	T	C
rs5757678	22	39843409	T	C
rs5759079	22	43393437	A	G
rs73165129	22	42315371	T	C
rs76146864	22	40246055	T	C
rs1081003	22	42525756	A	G
rs11770511	22	41957510	A	G
rs14980658	22	40774205	G	A
rs1894588	22	40629980	T	C
rs4657041	1	161478859	C	T
rs5757678	22	39843409	T	C
rs5759079	22	43393437	A	G
rs5996087	22	42321591	G	A
rs76146864	22	40246055	T	C
rs1084560	16	10824872	G	A
rs3087456	16	10970902	A	G
rs3104414	6	32582612	A	G
rs7032773	9	117645562	T	C
rs10882573	10	97009657	A	G
rs10882701	10	97741071	C	A
rs14101809	10	98169221	T	G
rs3814159	10	97515137	A	G
rs3824789	10	98800099	A	G
rs72809757	10	97285715	G	A
rs10882573	10	97009657	A	G
rs10882701	10	97741071	C	A
rs10883093	10	100183774	G	A
rs11188757	10	98205222	C	A

rs1410600	10	97070694	T	C
rs2805590	10	98812166	T	C
rs4918962	10	97475574	C	A
rs10882573	10	97009657	A	G
rs10882701	10	97741071	C	A
rs11188465	10	97506400	T	C
rs1410600	10	97070694	T	C
rs14555726	10	95850055	G	A
rs7918541	10	98168559	G	A
rs10882573	10	97009657	A	G
rs10882701	10	97741071	C	A
rs10883093	10	100183774	G	A
rs14101809	10	98169221	T	G
rs2805590	10	98812166	T	C
rs61854874	10	89173298	A	G
rs6584018	10	97480752	G	A
rs72809757	10	97285715	G	A
rs10882655	10	97474371	A	G
rs9263475	6	31050348	G	A
rs9265951	6	31315270	T	C
rs10882659	10	97510704	C	T
rs12138291	1	166464476	A	G
rs2949661	1	167424924	T	C
rs35055340	1	165314063	T	C
rs10882701	10	97741071	C	A
rs1410600	10	97070694	T	C
rs2901833	10	96998591	A	G
rs6584017	10	97480462	A	G
rs10882701	10	97741071	C	A
rs12778797	10	96998688	C	T
rs1410600	10	97070694	T	C
rs14555726	10	95850055	G	A
rs1539433	10	97519645	T	C
rs1923692	10	98173715	G	T
rs10882701	10	97741071	C	A
rs11188465	10	97506400	T	C
rs12778797	10	96998688	C	T
rs1410600	10	97070694	T	C
rs9663948	10	98238236	C	T
rs10882701	10	97741071	C	A
rs2901833	10	96998591	A	G
rs10882701	10	97741071	C	A
rs11745883	10	96773382	A	G
rs12778618	10	95633120	C	T
rs1539433	10	97519645	T	C
rs7426056	2	204612058	G	A
rs10882701	10	97741071	C	A
rs14101809	10	98169221	T	G
rs3824789	10	98800099	A	G
rs4336961	10	97497563	C	A
rs72809762	10	97294154	T	C
rs11306360	1	161246613	G	A
rs1801274	1	161479745	G	A
rs34658867	20	44977184	C	A
rs745307	20	44747086	A	G
rs10905716	10	6114010	T	C
rs10905719	10	6115171	A	G
rs11124653	2	39112166	A	C

rs11579717	1	199999121	A	G
rs6183637(	1	188691469	G	A
rs1090571¢	10	6115171	A	G
rs11124653	2	39112166	A	C
rs11579717	1	199999121	A	G
rs6183637(	1	188691469	G	A
rs1090571¢	10	6115171	A	G
rs10919544	1	161508763	C	T
rs1115791£	11	111848073	T	C
rs1121443€	11	112830782	T	G
rs11753307	11	112710664	T	C
rs1488491¢	11	113153223	T	C
rs1750467£	11	110737507	A	G
rs7729173€	11	112749800	T	C
rs10919544	1	161508763	C	T
rs1261459¢	2	232290640	A	G
rs1102503€	11	19175633	A	G
rs2857229	12	6900616	G	A
rs11086984	20	44511627	T	C
rs162076	20	46785499	A	G
rs1801274	1	161479745	G	A
rs1883832	20	44746982	C	T
rs2250246	12	6553016	G	A
rs3557480?	3	32533246	C	T
rs7591825£	3	32945761	A	G
rs1106597¢	12	112059557	T	C
rs11275557	1	149716369	G	A
rs1162753(	1	150863650	A	G
rs14696322	4	17530633	A	G
rs1501457¢	1	149738590	C	T
rs1801274	1	161479745	G	A
rs18826841	1	150401679	T	C
rs11074932	16	10968336	C	T
rs11074932	16	10968336	C	T
rs11074934	16	10979440	T	C
rs6174252£	6	33072522	A	G
rs9269109	6	32443266	C	T
rs11074934	16	10979440	T	C
rs1632994	6	29926597	C	T
rs6174252£	6	33072522	A	G
rs9269109	6	32443266	C	T
rs1108035(	17	33774635	T	C
rs73402222	6	31388449	C	T
rs11086984	20	44511627	T	C
rs14771461	12	6605299	T	C
rs1883832	20	44746982	C	T
rs25680	12	6554628	A	G
rs3557480?	3	32533246	C	T
rs56237381	3	32924804	A	G
rs7248581€	20	45314435	C	T
rs7591825£	3	32945761	A	G
rs11086984	20	44511627	T	C
rs14771461	12	6605299	T	C
rs162076	20	46785499	A	G
rs1801274	1	161479745	G	A
rs1883832	20	44746982	C	T
rs25680	12	6554628	A	G
rs34101571	1	31211894	A	G

rs35574803	3	32533246	C	T
rs75918255	3	32945761	A	G
rs7621492	3	32302456	A	G
rs9989924	20	42019380	C	T
rs11086984	20	44511627	T	C
rs14464237	3	36349691	G	A
rs14771461	12	6605299	T	C
rs1801274	1	161479745	G	A
rs25680	12	6554628	A	G
rs35574803	3	32533246	C	T
rs4810485	20	44747947	G	T
rs56237381	3	32924804	A	G
rs709589	14	106306841	T	C
rs75918255	3	32945761	A	G
rs11086984	20	44511627	T	C
rs14464237	3	36349691	G	A
rs14771461	12	6605299	T	C
rs162076	20	46785499	A	G
rs1801274	1	161479745	G	A
rs1883832	20	44746982	C	T
rs25680	12	6554628	A	G
rs34101571	1	31211894	A	G
rs35574803	3	32533246	C	T
rs75918255	3	32945761	A	G
rs11086984	20	44511627	T	C
rs14771461	12	6605299	T	C
rs162076	20	46785499	A	G
rs1801274	1	161479745	G	A
rs1883832	20	44746982	C	T
rs25680	12	6554628	A	G
rs34101571	1	31211894	A	G
rs35574803	3	32533246	C	T
rs75918255	3	32945761	A	G
rs11123178	5	112408670	T	C
rs11124653	2	39112166	A	C
rs11579717	1	199999121	A	G
rs12712610	2	38897249	G	A
rs11124653	2	39112166	A	C
rs11579717	1	199999121	A	G
rs12712610	2	38897249	G	A
rs11124653	2	39112166	A	C
rs11579717	1	199999121	A	G
rs13891577	1	190079170	A	C
rs78689347	2	40773763	C	A
rs11125917	6	32640351	G	A
rs11187166	10	94516008	T	C
rs2304938	11	60167744	T	C
rs55809481	19	49872521	C	T
rs6459417	6	15858996	T	C
rs9461557	6	29765925	T	C
rs11128304	16	79205197	C	A
rs11225864	12	55672838	T	C
rs11257146	7	111704231	G	A
rs11344722	9	105346840	C	T
rs11507717	2	53872294	G	T
rs11521843	4	67836131	G	T
rs11656270	6	31360602	G	A
rs11676788	6	24745313	T	C

rs11698453	13	22348885	T	C
rs11747573	6	148775706	T	C
rs11749408	14	56862154	A	G
rs11792748	12	49315200	A	G
rs11801638	12	28662288	C	T
rs12755474	1	216352525	G	A
rs13785858	5	173002007	C	T
rs13920089	9	12799038	A	G
rs14220888	19	51829114	T	C
rs14253017	1	62672889	G	A
rs14349879	8	140342260	A	C
rs14459133	16	59010741	G	A
rs14582711	20	57132122	T	C
rs14607300	17	11512358	T	C
rs14698529	3	170169608	G	A
rs14902318	1	232754676	G	A
rs14992607	21	41143748	A	G
rs18223633	7	124954943	G	A
rs18408274	7	92791863	C	T
rs18874853	19	8271140	C	T
rs18980125	10	68336237	A	C
rs73127385	4	31396800	G	A
rs76325389	9	122676137	G	A
rs77047785	14	28364836	T	C
rs77935824	18	68968361	G	A
rs80194890	10	91332621	G	T
rs11751901	10	97403248	A	C
rs17190839	6	31138707	A	G
rs72847706	2	87055981	C	T
rs9357097	6	30285121	T	C
rs11136335	8	145003777	A	G
rs11554159	19	18285944	A	G
rs1800973	12	69744014	A	C
rs445	7	92408370	T	C
rs75994780	12	70949085	A	G
rs80216229	12	67839087	C	T
rs11138864	12	28246470	A	G
rs11233425	11	82732689	A	G
rs11413595	2	8930530	C	T
rs11421610	5	140131486	T	C
rs11476381	2	191319109	A	C
rs11595461	2	174896997	A	G
rs11700664	10	108143796	G	A
rs11742878	19	43422957	A	C
rs11797510	12	52408142	T	G
rs12656604	5	72679056	A	C
rs130071	6	31116210	A	G
rs14393017	10	70543706	A	G
rs14602778	11	54986405	C	T
rs14726442	15	78644995	G	A
rs14948718	8	131518018	T	C
rs14971697	16	75062625	G	A
rs14999465	3	18550864	A	C
rs14999465	3	18550864	A	C
rs19027229	2	9944052	A	G
rs19230267	16	85685140	T	C
rs2977034	8	88503714	T	C
rs34375694	16	19193363	G	A

rs53826469	12	97040021	C	A
rs55881385	1	34724192	T	G
rs55971473	1	117061778	T	C
rs61772921	1	80897562	C	T
rs62021713	15	50365389	T	C
rs62409210	4	25412773	A	G
rs62488802	8	7162695	T	C
rs742867	14	68217724	T	C
rs74707627	15	86484743	C	T
rs75420474	18	39828718	C	T
rs76343453	5	81353920	C	T
rs76506268	4	187981624	G	A
rs76938777	7	31473901	A	G
rs79190516	2	151197759	G	T
rs11141754	11	36584481	T	C
rs11195385	14	84194540	G	A
rs11197877	1	21025910	C	T
rs11256860	15	27818525	T	C
rs11283918	2	238422350	A	G
rs11285771	6	148462118	T	C
rs11343257	21	30128360	T	C
rs11381965	13	100586942	T	C
rs11421610	5	140131486	T	C
rs11450799	1	21892266	A	G
rs11476381	2	191319109	A	C
rs11480164	6	66316172	A	C
rs11537940	6	18617129	A	G
rs11587918	3	194347376	G	A
rs11595461	2	174896997	A	G
rs11658171	5	29413612	C	T
rs11687618	17	16446446	C	T
rs11695904	8	53929771	G	T
rs11703638	20	3521072	G	A
rs11716645	3	29402908	G	A
rs11719611	9	83853182	A	G
rs11726219	16	5991815	T	C
rs11727189	8	112881674	A	G
rs11741380	11	16290338	C	T
rs11742878	19	43422957	A	C
rs11743122	11	44356396	G	A
rs11746991	7	79791344	A	C
rs11751404	21	26316207	C	A
rs11778562	12	132283582	T	G
rs11780440	16	5944116	G	A
rs12464465	2	176542288	A	G
rs130071	6	31116210	A	G
rs13044559	20	36073660	A	G
rs13797846	2	228411157	A	G
rs13801033	1	161526187	T	C
rs13879946	2	9441279	T	C
rs13882958	1	79094990	A	G
rs13896234	2	146534594	C	T
rs13945742	12	47679643	T	C
rs13977203	3	10035985	G	T
rs14013439	16	17341866	C	T
rs14031117	4	8174723	T	C
rs14060563	16	68607748	T	C
rs14096328	1	24640467	T	C

rs14102361	3	15790899	A	G
rs14247041	13	73498369	T	C
rs14252285	7	1084031	T	C
rs14291181	12	114081812	A	G
rs14382502	8	28561116	T	C
rs14457187	1	65133062	G	A
rs14471172	2	145836600	T	C
rs14499340	12	38374287	A	G
rs14533640	6	78745175	T	C
rs14694009	15	94946993	T	G
rs14865840	3	153311399	T	C
rs14971697	16	75062625	G	A
rs14995752	6	52375282	A	G
rs14999465	3	18550864	A	C
rs14999465	3	18550864	A	C
rs15038357	14	43151367	C	T
rs15042732	11	118189351	A	G
rs15065056	1	83046200	C	A
rs15085626	16	26527473	T	C
rs15112080	9	96711406	A	G
rs15114592	18	38375388	A	G
rs15123748	16	78214566	C	A
rs16864501	3	152385976	A	G
rs17212021	6	32637474	C	T
rs17212998	12	13243168	G	A
rs18278438	1	18635367	T	G
rs18338276	4	116470715	C	A
rs18577892	7	148905542	A	G
rs18659590	15	78554710	A	G
rs18839460	16	70343845	G	T
rs18968389	10	89153882	A	C
rs19230267	16	85685140	T	C
rs19255327	4	114347303	T	C
rs2615702	16	81462243	T	C
rs34592474	7	57279055	C	T
rs34609592	9	136915695	A	G
rs45507192	10	21304171	A	G
rs53500571	8	144233069	A	G
rs55830085	4	106862213	T	C
rs55881385	1	34724192	T	G
rs58860560	6	79460848	A	C
rs59236867	8	42077996	C	T
rs61772921	1	80897562	C	T
rs61988500	14	86640070	C	T
rs62021713	15	50365389	T	C
rs62369101	5	97959184	A	G
rs62409210	4	25412773	A	G
rs62488802	8	7162695	T	C
rs62531041	19	48379346	T	C
rs72728362	8	127054086	C	T
rs72815709	2	80415786	C	T
rs72847598	17	71902183	C	T
rs72929459	2	200181613	T	C
rs74603821	12	101714936	C	A
rs74902462	12	59066617	T	C
rs74932477	2	229222112	A	G
rs75346347	18	7862061	T	C
rs75420474	18	39828718	C	T

rs75828981	2	144233823	A	G
rs75884646	15	95776793	A	G
rs76343453	5	81353920	C	T
rs76506268	4	187981624	G	A
rs76595423	1	102745011	G	T
rs76732756	1	221000197	A	G
rs77152285	4	93935039	G	A
rs77164608	7	21673701	A	G
rs77243672	14	76846837	G	T
rs77937827	20	20799344	C	T
rs78656354	1	166905650	A	G
rs78939697	6	67374008	C	A
rs79407975	10	28109090	A	C
rs79489889	3	39505173	A	G
rs79647440	7	142438302	T	C
rs79870119	4	64142694	A	G
rs79888820	15	96888705	C	T
rs80012883	13	94341740	T	G
rs80142250	13	110903174	T	C
rs80347872	6	562203	A	G
rs11143632	20	42701487	T	C
rs13937678	1	161847528	A	G
rs14876587	1	161221713	C	T
rs1801274	1	161479745	G	A
rs34658867	20	44977184	C	A
rs6032211	20	44092301	A	G
rs6687275	1	161515638	G	T
rs745307	20	44747086	A	G
rs11153472	10	98139264	T	G
rs11579717	1	199999121	A	G
rs11818051	10	97972294	T	C
rs14313281	10	96890004	C	T
rs17111341	10	97881031	C	T
rs18547438	10	96311553	T	G
rs3740373	10	95256200	A	C
rs4918969	10	97562938	A	G
rs77520132	10	98160835	A	G
rs11153472	10	98139264	T	G
rs11764821	10	98816434	T	C
rs11818051	10	97972294	T	C
rs12778618	10	95633120	C	T
rs12778837	10	96998744	C	T
rs17111341	10	97881031	C	T
rs7083411	10	97606602	T	C
rs72812685	10	95845989	A	G
rs11153472	10	98139264	T	G
rs11818051	10	97972294	T	C
rs12778618	10	95633120	C	T
rs12778797	10	96998688	C	T
rs17111341	10	97881031	C	T
rs72812685	10	95845989	A	G
rs7913082	10	97561886	C	T
rs11153472	10	98139264	T	G
rs11579717	1	199999121	A	G
rs11679417	1	194010208	C	T
rs11818051	10	97972294	T	C
rs11890666	2	38745822	A	G
rs12712610	2	38897249	G	A

rs12778837	10	96998744	C	T
rs1410600	10	97070694	T	C
rs17111341	10	97881031	C	T
rs18270257	10	96658384	A	C
rs34645541	1	195875605	A	C
rs35909109	10	98014850	T	C
rs4918969	10	97562938	A	G
rs77520132	10	98160835	A	G
rs11153472	10	98139264	T	G
rs11579717	1	199999121	A	G
rs11679417	1	194010208	C	T
rs11890666	2	38745822	A	G
rs12712610	2	38897249	G	A
rs14313281	10	96890004	C	T
rs18547438	10	96311553	T	G
rs34645541	1	195875605	A	C
rs35909109	10	98014850	T	C
rs7079761	10	97842115	A	G
rs72809762	10	97294154	T	C
rs77520132	10	98160835	A	G
rs11153472	10	98139264	T	G
rs11818051	10	97972294	T	C
rs12778618	10	95633120	C	T
rs17111341	10	97881031	C	T
rs2901833	10	96998591	A	G
rs4381306	10	97484131	T	C
rs72812685	10	95845989	A	G
rs11158593	14	65859984	A	G
rs2844608	6	31247414	T	C
rs3184504	12	111884608	C	T
rs951818	12	6896055	A	C
rs11169569	19	51897760	A	C
rs56043070	1	247719769	A	G
rs58483275	19	51868443	T	C
rs7245846	19	51731176	A	G
rs7351079	19	51574038	G	A
rs75773078	19	51742145	A	G
rs79377141	19	51538952	A	G
rs11169569	19	51897760	A	C
rs58483275	19	51868443	T	C
rs7245846	19	51731176	A	G
rs7351079	19	51574038	G	A
rs75773078	19	51742145	A	G
rs79377141	19	51538952	A	G
rs11169569	19	51897760	A	C
rs273640	19	51725824	T	G
rs11178044	11	132210362	T	C
rs12921174	16	430304	C	T
rs2995089	1	167431193	A	G
rs4657649	1	167173140	C	T
rs4657706	1	167728447	T	C
rs61814886	1	167136405	A	G
rs78132817	1	165191695	G	T
rs11187166	10	94516008	T	C
rs2070770	11	60230531	T	C
rs55809481	19	49872521	C	T
rs61731791	19	50940778	T	C
rs909846	6	15897003	G	T

rs9271146	6	32577282	C	A
rs9469053	6	31755776	G	A
rs11187515	10	95327429	T	C
rs11359423	10	98103215	T	C
rs12778618	10	95633120	C	T
rs35025323	10	97089991	C	T
rs4075310	10	97574689	T	C
rs7079761	10	97842115	A	G
rs11190298	1	157954450	A	G
rs11687711	8	135490341	A	G
rs11190298	1	157954450	A	G
rs13924954	6	102294761	G	A
rs6710361	2	87047145	A	G
rs9896155	17	33794697	G	A
rs11198349	6	32205324	A	G
rs14330512	6	13991643	C	T
rs14721093	6	13892767	A	G
rs2286975	16	11114006	A	G
rs3869145	6	31851354	A	G
rs7192768	16	11004905	C	T
rs74405933	6	14095755	A	G
rs9270599	6	32561656	A	G
rs9271768	6	32594188	G	A
rs9271768	6	32594188	G	A
rs11198349	6	32205324	A	G
rs6755452	2	87035076	G	T
rs11214436	11	112830782	T	G
rs11714740	6	101980039	G	A
rs77738700	11	112751407	A	G
rs79251210	6	100468602	A	G
rs11240590	6	31809504	A	G
rs4447195	11	60249734	T	C
rs6459417	6	15858996	T	C
rs6509428	19	49853881	T	C
rs11241731	1	206855483	A	G
rs11381251	1	207276336	G	A
rs14474380	1	207018206	A	G
rs11241731	1	206855483	A	G
rs11643297	16	28973268	G	A
rs11242140	6	28205826	T	C
rs13908417	6	43351686	A	C
rs1794511	6	32671207	G	T
rs709589	14	106306841	T	C
rs9265515	6	31297744	C	T
rs11249570	5	177033794	A	G
rs4987369	1	169662742	T	G
rs11579717	1	199999121	A	G
rs12712610	2	38897249	G	A
rs13891577	1	190079170	A	C
rs13896071	4	123422719	C	A
rs14783814	22	47814108	G	T
rs61839660	10	6094697	T	C
rs74830391	7	37427289	T	C
rs11258182	2	204650296	A	G
rs72928038	6	90976768	A	G
rs11263784	6	149114553	A	C
rs11579717	1	199999121	A	G
rs11679417	1	194010208	C	T

rs12314907	12	79661771	G	A
rs12712610	2	38897249	G	A
rs61038907	2	87061070	A	C
rs74577787	3	84799491	T	C
rs11275557	1	149716369	G	A
rs11627530	1	150863650	A	G
rs14582812	1	120233380	A	G
rs15014579	1	149738590	C	T
rs1801274	1	161479745	G	A
rs18826841	1	150401679	T	C
rs58775931	1	121480104	T	G
rs80178684	8	59726631	G	A
rs11275557	1	149716369	G	A
rs11627530	1	150863650	A	G
rs14582812	1	120233380	A	G
rs15014579	1	149738590	C	T
rs1801274	1	161479745	G	A
rs18826841	1	150401679	T	C
rs80178684	8	59726631	G	A
rs11311620	1	198830942	C	T
rs11311620	1	198830942	C	T
rs11578049	6	32501627	G	T
rs13952934	1	161563554	G	A
rs13994705	12	25434182	A	G
rs1800973	12	69744014	A	C
rs4910742	11	5306509	A	G
rs80297185	11	5488177	T	C
rs62400929	6	31159546	G	A
rs9501107	6	31392047	T	G
rs12712610	2	38897249	G	A
rs62400929	6	31159546	G	A
rs14803171	6	31148873	C	T
rs11339844	6	161871755	C	A
rs11343534	1	198151688	G	A
rs11343534	1	198151688	G	A
rs13891577	1	190079170	A	C
rs11350090	6	100774499	T	C
rs13210358	6	102272234	T	C
rs34546581	2	87021659	A	G
rs5996087	22	42321591	G	A
rs11381251	1	207276336	G	A
rs14474380	1	207018206	A	G
rs3934748	1	206879659	T	G
rs11381251	1	207276336	G	A
rs3934748	1	206879659	T	G
rs11381251	1	207276336	G	A
rs14474380	1	207018206	A	G
rs55712453	1	206902437	G	A
rs11381251	1	207276336	G	A
rs14474380	1	207018206	A	G
rs55712453	1	206902437	G	A
rs11381251	1	207276336	G	A
rs78760135	1	206909820	G	A
rs79856590	1	205241642	A	G
rs11381251	1	207276336	G	A
rs14474380	1	207018206	A	G
rs55712453	1	206902437	G	A
rs11381251	1	207276336	G	A

rs11643297	16	28973268	G	A
rs55712453	1	206902437	G	A
rs11386504	2	176969703	A	G
rs2455069	19	51728641	G	A
rs11406846	6	33054463	T	C
rs2760980	6	32565201	A	G
rs9268498	6	32375431	C	T
rs11413595	2	8930530	C	T
rs130071	6	31116210	A	G
rs14038592	15	69903187	G	A
rs17212021	6	32637474	C	T
rs1736913	6	29704400	C	T
rs19031265	16	81625215	A	G
rs450175	2	38400812	C	A
rs72815709	2	80415786	C	T
rs742867	14	68217724	T	C
rs11459059	1	185852065	T	C
rs3865444	19	51727962	A	C
rs78744187	19	33754548	T	C
rs11459059	1	185852065	T	C
rs78744187	19	33754548	T	C
rs11467253	1	168306279	A	G
rs2949661	1	167424924	T	C
rs4657649	1	167173140	C	T
rs11467253	1	168306279	A	G
rs12138291	1	166464476	A	G
rs2949661	1	167424924	T	C
rs35055340	1	165314063	T	C
rs11467253	1	168306279	A	G
rs12138291	1	166464476	A	G
rs2949661	1	167424924	T	C
rs35055340	1	165314063	T	C
rs11467253	1	168306279	A	G
rs2949661	1	167424924	T	C
rs35055340	1	165314063	T	C
rs11496804	6	32666960	C	T
rs3104369	6	32602482	C	T
rs11506220	4	15956227	G	A
rs572542	4	14029370	T	C
rs76112278	4	15915069	A	G
rs76354299	4	15037408	A	G
rs11506220	4	15956227	G	A
rs76112278	4	15915069	A	G
rs11506220	4	15956227	G	A
rs14985299	4	15044338	T	G
rs197429	1	112267500	T	C
rs572542	4	14029370	T	C
rs76112278	4	15915069	A	G
rs11506220	4	15956227	G	A
rs76112278	4	15915069	A	G
rs76354299	4	15037408	A	G
rs11506220	4	15956227	G	A
rs76112278	4	15915069	A	G
rs76354299	4	15037408	A	G
rs11538327	1	161531340	A	G
rs77640671	16	58607804	T	C
rs11538327	1	161531340	A	G
rs74975395	1	166026114	T	C

rs11538327	1	161531340	A	G
rs11538327	1	161531340	A	G
rs15014579	1	149738590	C	T
rs11538327	1	161531340	A	G
rs74975395	1	166026114	T	C
rs11538327	1	161531340	A	G
rs3826007	15	80263217	T	C
rs11544989	8	145057868	G	A
rs11553660	6	101417409	T	C
rs11714740	6	101980039	G	A
rs77738700	11	112751407	A	G
rs79251210	6	100468602	A	G
rs11544989	8	145057868	G	A
rs11553660	6	101417409	T	C
rs11714740	6	101980039	G	A
rs77738700	11	112751407	A	G
rs79251210	6	100468602	A	G
rs9916257	17	33797371	T	G
rs11548656	16	81916912	G	A
rs41294937	10	6106621	C	T
rs11553660	6	101417409	T	C
rs11714740	6	101980039	G	A
rs7569868	2	87019683	T	C
rs77738700	11	112751407	A	G
rs79251210	6	100468602	A	G
rs11553660	6	101417409	T	C
rs11714740	6	101980039	G	A
rs7569868	2	87019683	T	C
rs77738700	11	112751407	A	G
rs79251210	6	100468602	A	G
rs11553660	6	101417409	T	C
rs11714740	6	101980039	G	A
rs7218011	17	33773022	G	A
rs77738700	11	112751407	A	G
rs7819099	8	144992862	G	A
rs79251210	6	100468602	A	G
rs9912354	17	33805730	T	C
rs11553660	6	101417409	T	C
rs11714740	6	101980039	G	A
rs77291736	11	112749800	T	C
rs79251210	6	100468602	A	G
rs11553660	6	101417409	T	C
rs11714740	6	101980039	G	A
rs77738700	11	112751407	A	G
rs7819099	8	144992862	G	A
rs79251210	6	100468602	A	G
rs11553660	6	101417409	T	C
rs11714740	6	101980039	G	A
rs77738700	11	112751407	A	G
rs79251210	6	100468602	A	G
rs9916257	17	33797371	T	G
rs11554159	19	18285944	A	G
rs1800973	12	69744014	A	C
rs445	7	92408370	T	C
rs75994780	12	70949085	A	G
rs11562414	1	161701708	A	C
rs11580516	1	161410253	T	C
rs71632979	1	161526397	G	A

rs71639910	1	161937774	A	G
rs1573735	16	423696	T	C
rs3129891	6	32415080	A	G
rs9271772	6	32594275	A	G
rs11575242	1	205560814	A	G
rs11579717	1	199999121	A	G
rs12712610	2	38897249	G	A
rs13891577	1	190079170	A	C
rs13896071	4	123422719	C	A
rs74830391	7	37427289	T	C
rs7893324	10	6111075	C	T
rs11579717	1	199999121	A	G
rs13891577	1	190079170	A	C
rs408686	2	39177320	A	C
rs6751481	2	38897810	C	T
rs11579717	1	199999121	A	G
rs7559619	2	38892207	G	A
rs11579717	1	199999121	A	G
rs11679417	1	194010208	C	T
rs17607399	2	41884212	T	C
rs6751481	2	38897810	C	T
rs11579717	1	199999121	A	G
rs2105899	6	32590473	C	T
rs11579717	1	199999121	A	G
rs11579717	1	199999121	A	G
rs408686	2	39177320	A	C
rs6751481	2	38897810	C	T
rs11579717	1	199999121	A	G
rs13891577	1	190079170	A	C
rs61839660	10	6094697	T	C
rs11579717	1	199999121	A	G
rs13891577	1	190079170	A	C
rs17607399	2	41884212	T	C
rs18708934	1	182091538	C	A
rs34645541	1	195875605	A	C
rs6751481	2	38897810	C	T
rs7530076	1	195825276	A	G
rs11579717	1	199999121	A	G
rs11679417	1	194010208	C	T
rs17607399	2	41884212	T	C
rs6751481	2	38897810	C	T
rs11579717	1	199999121	A	G
rs13891577	1	190079170	A	C
rs14594721	2	38596674	T	C
rs14769248	2	39864242	T	C
rs34558770	2	38933040	T	C
rs7605193	2	38698112	G	T
rs79747818	2	41757305	T	C
rs917116	7	28172739	G	T
rs11579717	1	199999121	A	G
rs13891577	1	190079170	A	C
rs17607399	2	41884212	T	C
rs18708934	1	182091538	C	A
rs60220946	1	193505625	T	C
rs6751481	2	38897810	C	T
rs7530076	1	195825276	A	G
rs11579717	1	199999121	A	G
rs61839660	10	6094697	T	C

rs11579717	1	199999121	A	G
rs13891577	1	190079170	A	C
rs11579717	1	199999121	A	G
rs13891577	1	190079170	A	C
rs6183966(	10	6094697	T	C
rs11579717	1	199999121	A	G
rs11675976	1	189981116	A	G
rs11579717	1	199999121	A	G
rs11679417	1	194010208	C	T
rs1271261(	2	38897249	G	A
rs4832054	2	87058580	A	G
rs79827501	2	38431267	T	G
rs11579717	1	199999121	A	G
rs5413673(	1	198283058	A	G
rs11579717	1	199999121	A	G
rs11679417	1	194010208	C	T
rs1271261(	2	38897249	G	A
rs2367376	2	87009742	C	T
rs11580516	1	161410253	T	C
rs569911	1	160798209	C	T
rs71632989	1	161533055	G	T
rs7180804	15	80259074	A	G
rs865239	1	156896164	G	A
rs9916257	17	33797371	T	G
rs11580516	1	161410253	T	C
rs1476784(	1	161035780	A	G
rs18719756	1	161006723	T	C
rs71632979	1	161526397	G	A
rs7163991(	1	161937774	A	G
rs11580516	1	161410253	T	C
rs569911	1	160798209	C	T
rs71632979	1	161526397	G	A
rs11580516	1	161410253	T	C
rs19121795	1	160940671	A	G
rs71632979	1	161526397	G	A
rs7163991(	1	161937774	A	G
rs11580516	1	161410253	T	C
rs71632989	1	161533055	G	T
rs876036	7	50307710	C	T
rs11580516	1	161410253	T	C
rs71632989	1	161533055	G	T
rs7163991(	1	161937774	A	G
rs11580516	1	161410253	T	C
rs19121795	1	160940671	A	G
rs71632979	1	161526397	G	A
rs7163991(	1	161937774	A	G
rs11580516	1	161410253	T	C
rs569911	1	160798209	C	T
rs71632979	1	161526397	G	A
rs11580516	1	161410253	T	C
rs71632979	1	161526397	G	A
rs11580516	1	161410253	T	C
rs19121795	1	160940671	A	G
rs71632979	1	161526397	G	A
rs7163991(	1	161937774	A	G
rs11580516	1	161410253	T	C
rs14314055	1	160972255	T	C
rs14876587	1	161221713	C	T

rs71632979	1	161526397	G	A
rs71639910	1	161937774	A	G
rs11580516	1	161410253	T	C
rs6537835	1	115108914	A	G
rs71632989	1	161533055	G	T
rs71639910	1	161937774	A	G
rs11580516	1	161410253	T	C
rs14876587	1	161221713	C	T
rs19121795	1	160940671	A	G
rs71632979	1	161526397	G	A
rs71639910	1	161937774	A	G
rs11580516	1	161410253	T	C
rs3845548	1	161507448	T	C
rs3856266	1	157973889	G	T
rs4656984	1	161078909	C	T
rs77418963	1	162197834	G	A
rs78569595	1	162032153	T	G
rs11580516	1	161410253	T	C
rs71632979	1	161526397	G	A
rs71639910	1	161937774	A	G
rs78600824	1	160841013	G	A
rs11580516	1	161410253	T	C
rs569911	1	160798209	C	T
rs71632979	1	161526397	G	A
rs11580516	1	161410253	T	C
rs569911	1	160798209	C	T
rs71632979	1	161526397	G	A
rs11593724	2	136121922	A	G
rs74529033	2	137747326	G	A
rs11598494	10	6178941	C	T
rs1887027	10	6113782	T	C
rs3793662	9	95886051	T	C
rs11598494	10	6178941	C	T
rs1887027	10	6113782	T	C
rs11598494	10	6178941	C	T
rs1887027	10	6113782	T	C
rs3793662	9	95886051	T	C
rs11598494	10	6178941	C	T
rs1887027	10	6113782	T	C
rs3793662	9	95886051	T	C
rs11600782	6	31569745	G	A
rs9270588	6	32561327	T	C
rs11600782	6	31569745	G	A
rs11644019	16	10863414	T	C
rs34039593	6	32570311	G	T
rs7748976	6	33890451	A	G
rs9296042	6	32736005	T	C
rs11600782	6	31569745	G	A
rs2858885	6	32699782	T	C
rs35525122	6	32561283	A	C
rs55971447	1	161515326	T	C
rs6925683	6	33894292	G	T
rs11600782	6	31569745	G	A
rs34039593	6	32570311	G	T
rs362522	6	29545208	T	C
rs55971447	1	161515326	T	C
rs7748976	6	33890451	A	G
rs9296042	6	32736005	T	C

rs11603081	1	42691989	C	T
rs7559619	2	38892207	G	A
rs11615628	12	6903631	A	G
rs9272242	6	32602790	C	T
rs11615628	12	6903631	A	G
rs11615628	12	6903631	A	G
rs2844608	6	31247414	T	C
rs11615628	12	6903631	A	G
rs11615628	12	6903631	A	G
rs11615628	12	6903631	A	G
rs2253487	6	31249127	A	G
rs9263475	6	31050348	G	A
rs11615628	12	6903631	A	G
rs11615628	12	6903631	A	G
rs11615628	12	6903631	A	G
rs11615628	12	6903631	A	G
rs11641185	16	10986596	T	C
rs11642314	5	174310054	A	G
rs11643297	16	28973268	G	A
rs75968672	1	206890212	G	A
rs11643297	16	28973268	G	A
rs75968672	1	206890212	G	A
rs11651760	1	198922068	T	C
rs12712613	2	38948860	T	G
rs11653761	17	2716535	T	G
rs12874404	13	108993494	G	A
rs11653761	17	2716535	T	G
rs11777835	8	144986103	C	T
rs9520836	13	108957063	G	A
rs11657118	17	2714118	C	T
rs12874404	13	108993494	G	A
rs11657531	17	2731370	T	C
rs12874404	13	108993494	G	A
rs11657759	17	2715961	C	A
rs12874404	13	108993494	G	A
rs73192651	13	50212885	T	C
rs11667181	6	31450274	A	G
rs61742525	6	33072522	A	G
rs6808893	3	188133439	T	C
rs73499473	16	10978885	T	C
rs9269109	6	32443266	C	T
rs11669394	19	53262327	A	G
rs13871252	19	51544665	A	G
rs3865444	19	51727962	A	C
rs56043070	1	247719769	A	G
rs7351079	19	51574038	G	A
rs75773078	19	51742145	A	G
rs11679417	1	194010208	C	T
rs17583875	1	197924770	A	G
rs61839660	10	6094697	T	C
rs11679417	1	194010208	C	T
rs17583875	1	197924770	A	G
rs60220946	1	193505625	T	C
rs11687597	2	136926146	G	A
rs7840395	8	144984956	G	A
rs11703762	16	423809	G	A
rs4521502	5	35842748	G	T
rs11706770	17	62128083	G	A

rs14484397	17	63066415	T	C
rs62045817	16	89045233	T	C
rs722926	17	8677215	T	C
rs11708621	22	39876838	G	A
rs1305859C	22	39641412	T	C
rs14606972	22	43339331	T	C
rs2142827	22	42874781	A	G
rs5758649	22	42605548	T	C
rs11708621	22	39876838	G	A
rs1305859C	22	39641412	T	C
rs1439447€	22	44378670	T	C
rs14488623	22	42326166	C	T
rs14606972	22	43339331	T	C
rs2142827	22	42874781	A	G
rs5758649	22	42605548	T	C
rs11708621	22	39876838	G	A
rs1305859C	22	39641412	T	C
rs1439447€	22	44378670	T	C
rs14606972	22	43339331	T	C
rs2142827	22	42874781	A	G
rs4657041	1	161478859	C	T
rs5758649	22	42605548	T	C
rs77657071	22	42324463	A	G
rs11708621	22	39876838	G	A
rs14278001	22	44395926	A	G
rs2142827	22	42874781	A	G
rs4657041	1	161478859	C	T
rs5758649	22	42605548	T	C
rs75056525	22	43349884	A	G
rs11708621	22	39876838	G	A
rs1305859C	22	39641412	T	C
rs14606972	22	43339331	T	C
rs2142827	22	42874781	A	G
rs5758649	22	42605548	T	C
rs11708621	22	39876838	G	A
rs14278001	22	44395926	A	G
rs2142827	22	42874781	A	G
rs5758649	22	42605548	T	C
rs75056525	22	43349884	A	G
rs1171474C	6	101980039	G	A
rs12986962	2	111808558	G	A
rs4796089	17	33814758	C	T
rs11729508	20	45333273	G	A
rs1801274	1	161479745	G	A
rs60629714	9	95889160	C	T
rs745307	20	44747086	A	G
rs11730825	14	31993501	C	T
rs11748905	5	80934634	C	T
rs11751198	6	31753526	A	G
rs55809481	19	49872521	C	T
rs61731791	19	50940778	T	C
rs78287249	11	60157281	T	G
rs62183989	2	204609402	T	C
rs5620941€	2	204627500	C	T
rs11765339	10	97551952	A	G
rs4707609	6	90946479	C	T
rs11766311	7	77134132	A	G
rs445	7	92408370	T	C

rs11770511	22	41957510	A	G
rs1180403(	22	42616172	T	C
rs1217000З	22	37748690	T	C
rs1482897ϵ	22	43472311	C	A
rs1905296ƽ	22	38163121	A	G
rs5996088	22	42323913	T	C
rs1177376З	7	50271499	T	C
rs4987358	1	169665551	T	G
rs1179134(	6	102173117	G	A
rs79086781	6	100526652	T	C
rs1758387ƽ	1	197924770	A	G
rs2463509	2	103672986	T	C
rs1509561ȣ	1	214625781	C	T
rs1758387ƽ	1	197924770	A	G
rs2463509	2	103672986	T	C
rs12901644	15	36456429	T	C
rs3131627	6	31415065	G	A
rs1195422З	5	67413499	A	G
rs12874404	13	108993494	G	A
rs18305332	1	25076059	T	C
rs7218011	17	33773022	G	A
rs9916257	17	33797371	T	G
rs1195422З	5	67413499	A	G
rs9520836	13	108957063	G	A
rs9916257	17	33797371	T	G
rs1196177	6	32379535	C	T
rs1800973	12	69744014	A	C
rs9270585	6	32561300	T	C
rs9271768	6	32594188	G	A
rs9271768	6	32594188	G	A
rs9469245	6	32692084	T	C
rs1196177	6	32379535	C	T
rs1800973	12	69744014	A	C
rs9270585	6	32561300	T	C
rs9271768	6	32594188	G	A
rs9271768	6	32594188	G	A
rs9469245	6	32692084	T	C
rs1196177	6	32379535	C	T
rs1354106	19	51737991	G	T
rs2071473	6	32782605	T	C
rs9270591	6	32561424	T	C
rs9271768	6	32594188	G	A
rs9271768	6	32594188	G	A
rs1196409ƽ	6	29501792	A	C
rs35118762	6	32560631	T	C
rs394199	6	33553580	T	C
rs12075684	1	169620673	G	A
rs6427213	1	169713146	G	A
rs12138291	1	166464476	A	G
rs2949661	1	167424924	T	C
rs858496	1	167500918	G	T
rs12138291	1	166464476	A	G
rs2995089	1	167431193	A	G
rs4657649	1	167173140	C	T
rs7519927	1	167100361	A	G
rs12145132	1	198636736	G	A
rs41289614	3	45972492	T	C
rs4657649	1	167173140	C	T

rs12752576	1	168605042	G	A
rs61814886	1	167136405	A	G
rs6658665	1	166460696	G	A
rs6668517	1	26556870	C	A
rs74620952	11	117753136	C	T
rs12206866	6	107159822	T	C
rs3129765	6	32591428	G	A
rs7745305	6	30913475	C	T
rs12212931	6	15913304	G	T
rs30003	19	49846398	C	T
rs4939384	11	60273642	G	A
rs72836542	6	14091682	T	C
rs9270560	6	32560741	C	T
rs12212931	6	15913304	G	T
rs1941018	11	60268994	G	A
rs35139284	6	32561370	T	C
rs354026	19	49841054	T	G
rs12342539	9	4327960	T	C
rs12722531	10	6076076	A	G
rs706779	10	6098824	C	T
rs12346499	9	113902881	T	C
rs12459503	19	52161035	C	T
rs3865444	19	51727962	A	C
rs55862503	19	51858911	A	G
rs7351079	19	51574038	G	A
rs78949787	19	51591138	T	C
rs12554596	9	37002142	G	A
rs2066399	6	15880540	A	G
rs2070770	11	60230531	T	C
rs30003	19	49846398	C	T
rs12596540	16	10970476	G	A
rs1723018	1	167433420	G	A
rs12598451	16	10982607	A	G
rs13917417	6	32592834	A	C
rs71632979	1	161526397	G	A
rs805288	6	31678028	T	C
rs885947	6	29696656	A	G
rs9268628	6	32405865	C	A
rs1264580	6	30301230	A	G
rs9270657	6	32566021	G	T
rs9461744	6	32388317	G	A
rs12675296	8	6828795	A	G
rs1886654	1	236105910	C	T
rs2981398	8	6801712	G	A
rs12675298	8	6828813	A	G
rs445	7	92408370	T	C
rs12712610	2	38897249	G	A
rs9271537	6	32589982	T	C
rs12712610	2	38897249	G	A
rs12712610	2	38897249	G	A
rs12722600	10	6054765	T	C
rs12778618	10	95633120	C	T
rs3181132	10	97601894	G	A
rs12865434	13	108970631	G	A
rs12874404	13	108993494	G	A
rs12874404	13	108993494	G	A
rs6032660	20	44730245	A	G
rs73192651	13	50212885	T	C

rs12874404	13	108993494	G	A
rs12874404	13	108993494	G	A
rs12874404	13	108993494	G	A
rs73192651	13	50212885	T	C
rs12874404	13	108993494	G	A
rs12874404	13	108993494	G	A
rs9892810	17	2716155	C	T
rs12874404	13	108993494	G	A
rs12921174	16	430304	C	T
rs2949661	1	167424924	T	C
rs4657649	1	167173140	C	T
rs79179998	3	45947066	A	G
rs12986962	2	111808558	G	A
rs58745116	17	33803592	A	G
rs56262516	6	29930939	A	G
rs9263601	6	31071758	C	T
rs13023673	2	38594483	C	T
rs54136730	1	198283058	A	G
rs6707421	2	38897781	G	A
rs79827501	2	38431267	T	G
rs13058590	22	39641412	T	C
rs14278001	22	44395926	A	G
rs14606972	22	43339331	T	C
rs15061210	22	40175781	A	G
rs4657041	1	161478859	C	T
rs5758649	22	42605548	T	C
rs73165129	22	42315371	T	C
rs13058590	22	39641412	T	C
rs14278001	22	44395926	A	G
rs14606972	22	43339331	T	C
rs15061210	22	40175781	A	G
rs4657041	1	161478859	C	T
rs5758649	22	42605548	T	C
rs73165129	22	42315371	T	C
rs13172065	5	35887454	G	A
rs1573734	16	423619	T	C
rs28680958	1	173848808	A	G
rs13210358	6	102272234	T	C
rs1322067	9	117660933	G	A
rs3087456	16	10970902	A	G
rs13390252	2	204596729	G	A
rs59639671	2	39003961	T	C
rs1375493	2	182323766	A	G
rs2418135	9	113901309	A	G
rs445	7	92408370	T	C
rs13891577	1	190079170	A	C
rs17583875	1	197924770	A	G
rs7530076	1	195825276	A	G
rs13908417	6	43351686	A	C
rs241407	6	32862740	T	C
rs28723652	14	106644507	A	C
rs709589	14	106306841	T	C
rs9267517	6	31573110	A	G
rs9276702	6	32756741	G	A
rs13913272	17	55643849	T	C
rs14258741	17	58777014	A	G
rs14613046	17	61576413	A	G
rs14717344	17	59945942	T	G

rs14785074	17	56374364	T	C
rs445	7	92408370	T	C
rs77771777	17	55927801	T	C
rs13924954	6	102294761	G	A
rs6755452	2	87035076	G	T
rs9896155	17	33794697	G	A
rs13924954	6	102294761	G	A
rs7569868	2	87019683	T	C
rs7925121(	6	100468602	A	G
rs9896155	17	33794697	G	A
rs13924954	6	102294761	G	A
rs79086781	6	100526652	T	C
rs13924954	6	102294761	G	A
rs7569868	2	87019683	T	C
rs7925121(	6	100468602	A	G
rs13924954	6	102294761	G	A
rs7925121(	6	100468602	A	G
rs13924954	6	102294761	G	A
rs79086781	6	100526652	T	C
rs13924954	6	102294761	G	A
rs6218401(	2	204633784	T	C
rs79086781	6	100526652	T	C
rs13924954	6	102294761	G	A
rs75494211	6	101911859	A	G
rs7819099	8	144992862	G	A
rs7826811(	11	112705025	T	C
rs1392607(	2	105578959	T	C
rs80330977	5	158276610	A	G
rs1392905(	22	44349795	G	A
rs14606972	22	43339331	T	C
rs1506121(	22	40175781	A	G
rs5758649	22	42605548	T	C
rs7316512(	22	42315371	T	C
rs1394899(	1	208017826	A	G
rs3610215(	15	50363583	A	G
rs4406271	6	109598213	A	G
rs586030	1	208170487	T	C
rs13979522	1	92842367	C	A
rs2325259	6	90850164	C	T
rs6445506	3	52528292	C	T
rs7163297(	1	161526397	G	A
rs13979522	1	92842367	C	A
rs2325259	6	90850164	C	T
rs6445484	3	52503800	G	A
rs13979522	1	92842367	C	A
rs1412643(	1	90530314	C	T
rs3507515(	3	53273195	C	T
rs758800	3	52529266	C	T
rs7606494(	1	93926697	G	T
rs7901543(	1	94803124	T	C
rs1400885(	2	105581937	C	T
rs1403755	18	30263492	T	C
rs2875404	3	76674553	T	C
rs7866727(	2	54957709	A	G
rs4683184	3	46187707	A	G
rs7964138(	3	46882338	C	A
rs1421864(	6	31473707	G	A
rs613165	6	31834398	A	G

rs9270588	6	32561327	T	C
rs14245623	11	4960640	T	C
rs17552904	7	50318308	T	G
rs722926	17	8677215	T	C
rs78736654	11	5544477	G	T
rs14245623	11	4960640	T	C
rs14340733	7	1095701	T	C
rs3092963	3	46396938	G	A
rs14340733	7	1095701	T	C
rs3092963	3	46396938	G	A
rs876036	7	50307710	C	T
rs1800973	12	69744014	A	C
rs445	7	92408370	T	C
rs62395272	6	31394424	T	C
rs6934244	6	33024032	A	C
rs9275511	6	32674329	A	G
rs14662058	1	198524264	T	G
rs6707421	2	38897781	G	A
rs14676534	11	11413255	T	C
rs60499622	10	126551228	T	C
rs14748747	10	6191376	A	C
rs41294937	10	6106621	C	T
rs14748747	10	6191376	A	C
rs17403348	10	5827901	C	T
rs3793662	9	95886051	T	C
rs41294937	10	6106621	C	T
rs14748747	10	6191376	A	C
rs17403348	10	5827901	C	T
rs3793662	9	95886051	T	C
rs62626323	10	6115678	A	C
rs709589	14	106306841	T	C
rs14748747	10	6191376	A	C
rs3793662	9	95886051	T	C
rs41294937	10	6106621	C	T
rs14748747	10	6191376	A	C
rs14966210	7	47871972	A	G
rs62626323	10	6115678	A	C
rs14764033	1	161536758	C	T
rs78358948	11	112751974	A	G
rs14776376	10	97548303	A	G
rs14919284	22	42270864	C	T
rs4822134	22	42867332	C	T
rs73165129	22	42315371	T	C
rs15014579	1	149738590	C	T
rs558721	6	32570573	T	C
rs9597833	13	59334076	G	T
rs1569590	6	15898884	G	A
rs55809481	19	49872521	C	T
rs1569590	6	15898884	G	A
rs2070770	11	60230531	T	C
rs30003	19	49846398	C	T
rs9461562	6	29778271	G	A
rs1571025	10	6134869	G	A
rs6602392	10	6078079	A	C
rs1571026	10	6135025	G	T
rs1573732	16	423420	T	C
rs2228015	17	38715186	C	T
rs61968809	13	106399307	A	G

rs75983909	17	38723925	T	C
rs1573732	16	423420	T	C
rs2228015	17	38715186	C	T
rs1573734	16	423619	T	C
rs1617988	1	167436300	C	T
rs4657649	1	167173140	C	T
rs876036	7	50307710	C	T
rs16988872	19	51580161	T	C
rs3865444	19	51727962	A	C
rs17201560	6	32047268	C	T
rs2571390	6	29923554	C	T
rs3020726	2	87016506	G	A
rs17203563	6	32446459	A	G
rs1723016	1	167433056	C	T
rs1723018	1	167433420	G	A
rs1723018	1	167433420	G	A
rs1723018	1	167433420	G	A
rs61814886	1	167136405	A	G
rs1723018	1	167433420	G	A
rs1723018	1	167433420	G	A
rs1723018	1	167433420	G	A
rs62028293	16	50264054	C	T
rs17552904	7	50318308	T	G
rs17583875	1	197924770	A	G
rs3130685	6	31206206	T	C
rs17583875	1	197924770	A	G
rs408686	2	39177320	A	C
rs6751481	2	38897810	C	T
rs17583875	1	197924770	A	G
rs2596459	6	31417540	T	G
rs6707421	2	38897781	G	A
rs653178	12	112007756	T	C
rs61839660	10	6094697	T	C
rs4959028	6	32383138	G	A
rs17664049	18	48685445	T	C
rs2480679	1	167407517	G	A
rs2988279	1	167419839	A	G
rs1773542	1	167432949	C	T
rs17824379	3	5810961	G	A
rs1799693	6	106992464	G	A
rs9398107	6	107155687	G	A
rs1800973	12	69744014	A	C
rs1800973	12	69744014	A	C
rs62395272	6	31394424	T	C
rs9270588	6	32561327	T	C
rs9271768	6	32594188	G	A
rs9271768	6	32594188	G	A
rs9469245	6	32692084	T	C
rs1800973	12	69744014	A	C
rs762789	3	46402627	A	G
rs77465361	5	159009803	T	G
rs1800973	12	69744014	A	C
rs77762937	2	203467470	T	G
rs1801274	1	161479745	G	A
rs3820097	1	161049358	A	G
rs3826007	15	80263217	T	C
rs1801274	1	161479745	G	A
rs2664523	20	45318042	A	G

rs6687275	1	161515638	G	T
rs745307	20	44747086	A	G
rs1801274	1	161479745	G	A
rs8046381	16	31379355	C	T
rs1801274	1	161479745	G	A
rs1801274	1	161479745	G	A
rs1801274	1	161479745	G	A
rs3865444	19	51727962	A	C
rs4802858	19	52253150	C	T
rs5586250	19	51858911	A	G
rs5848327	19	51868443	T	C
rs7351079	19	51574038	G	A
rs7894978	19	51591138	T	C
rs1801274	1	161479745	G	A
rs7286595	3	39355919	A	G
rs1801274	1	161479745	G	A
rs3820097	1	161049358	A	G
rs3826007	15	80263217	T	C
rs1801274	1	161479745	G	A
rs1801274	1	161479745	G	A
rs1801274	1	161479745	G	A
rs1801274	1	161479745	G	A
rs1801274	1	161479745	G	A
rs1801274	1	161479745	G	A
rs1801274	1	161479745	G	A
rs3865444	19	51727962	A	C
rs5586250	19	51858911	A	G
rs7305193	19	48582916	G	A
rs7351079	19	51574038	G	A
rs7894978	19	51591138	T	C
rs1801274	1	161479745	G	A
rs7286595	3	39355919	A	G
rs1801274	1	161479745	G	A
rs2649752	3	39351805	A	C
rs1801274	1	161479745	G	A
rs1801274	1	161479745	G	A
rs4683184	3	46187707	A	G
rs6183966	10	6094697	T	C
rs1830533	1	25076059	T	C
rs7216096	17	33772954	A	G
rs9916257	17	33797371	T	G
rs1830533	1	25076059	T	C
rs6721978	2	232290157	C	T
rs1860906	4	8970919	A	C
rs7611227	4	15915069	A	G
rs807784	4	13906853	T	G
rs1883832	20	44746982	C	T
rs30006	19	49847916	C	T
rs4716010	6	15898371	A	G
rs61731791	19	50940778	T	C
rs1883832	20	44746982	C	T
rs25680	12	6554628	A	G
rs3557480	3	32533246	C	T
rs7591825	3	32945761	A	G
rs7621492	3	32302456	A	G
rs7692986	3	31134534	C	A
rs1883832	20	44746982	C	T
rs2250246	12	6553016	G	A

rs1883832	20	44746982	C	T
rs41295093	10	6120173	A	G
rs1886730	1	2488608	C	T
rs1886730	1	2488608	C	T
rs1886730	1	2488608	C	T
rs1886730	1	2488608	C	T
rs1886730	1	2488608	C	T
rs1886730	1	2488608	C	T
rs18888277	10	16716607	C	A
rs19175322	1	196321168	T	C
rs73735764	6	46441386	T	C
rs19177913	4	82502357	G	A
rs6535445	4	84151427	C	T
rs19177913	4	82502357	G	A
rs6535445	4	84151427	C	T
rs1941018	11	60268994	G	A
rs30003	19	49846398	C	T
rs388354	7	40190449	C	A
rs72836542	6	14091682	T	C
rs9270560	6	32560741	C	T
rs1973872	2	204631016	G	T
rs72928038	6	90976768	A	G
rs1973872	2	204631016	G	T
rs72928038	6	90976768	A	G
rs19979771	4	120352238	C	T
rs7559619	2	38892207	G	A
rs28607988	3	45913338	C	A
rs2949661	1	167424924	T	C
rs4657649	1	167173140	C	T
rs2018404	14	105695446	T	C
rs2338377	14	106671275	A	G
rs28669075	15	40856521	T	C
rs709589	14	106306841	T	C
rs2040406	6	32603007	G	A
rs709589	14	106306841	T	C
rs2066399	6	15880540	A	G
rs4939384	11	60273642	G	A
rs72836542	6	14091682	T	C
rs9270560	6	32560741	C	T
rs2066399	6	15880540	A	G
rs34486765	19	49915911	A	G
rs72836542	6	14091682	T	C
rs7768875	6	32560752	T	C
rs7939177	11	60266070	T	C
rs2070901	1	161185058	T	G
rs2092305	6	33100189	G	A
rs72770017	16	10973612	A	G
rs2164210	2	136580287	C	T
rs2169052	1	161557154	C	T
rs35587265	3	46259296	G	A
rs55971447	1	161515326	T	C
rs2181036	9	117649136	C	T
rs3087456	16	10970902	A	G
rs72779785	16	10569039	G	A
rs2181036	9	117649136	C	T
rs3087456	16	10970902	A	G
rs72779785	16	10569039	G	A
rs2182176	1	2499254	A	G

rs2227313	1	2487766	C	T
rs2227313	1	2487766	C	T
rs2234161	1	2491205	T	C
rs2234161	1	2491205	T	C
rs2844594	6	31258393	A	G
rs2236073	6	106898421	A	G
rs72948545	6	107168477	G	A
rs2236073	6	106898421	A	G
rs9400058	6	107164076	G	A
rs2236073	6	106898421	A	G
rs72948545	6	107168477	G	A
rs2236073	6	106898421	A	G
rs9400058	6	107164076	G	A
rs2236073	6	106898421	A	G
rs9400058	6	107164076	G	A
rs2236073	6	106898421	A	G
rs9400058	6	107164076	G	A
rs2236073	6	106898421	A	G
rs7246669	19	16442023	G	A
rs72948545	6	107168477	G	A
rs2236073	6	106898421	A	G
rs72948545	6	107168477	G	A
rs2236073	6	106898421	A	G
rs9400058	6	107164076	G	A
rs2236947	3	50371432	A	C
rs445	7	92408370	T	C
rs2250246	12	6553016	G	A
rs4239702	20	44749251	C	T
rs2250246	12	6553016	G	A
rs35574803	3	32533246	C	T
rs4810485	20	44747947	G	T
rs2275848	9	95887320	T	G
rs41294937	10	6106621	C	T
rs709589	14	106306841	T	C
rs2337147	18	46483810	A	G
rs77346511	11	5268498	T	C
rs2395191	6	32447085	C	T
rs2455069	19	51728641	G	A
rs4499342	19	8786913	T	C
rs2490437	1	161937517	C	T
rs71632989	1	161533055	G	T
rs2517515	6	31029258	T	C
rs5750829	22	39838018	G	A
rs2523614	6	31320654	C	T
rs2523882	6	31042217	T	C
rs3020726	2	87016506	G	A
rs2523887	6	31040725	G	A
rs2571390	6	29923554	C	T
rs25680	12	6554628	A	G
rs35574803	3	32533246	C	T
rs4810485	20	44747947	G	T
rs56237381	3	32924804	A	G
rs75918255	3	32945761	A	G
rs2569177	5	139992162	T	G
rs6671847	1	161478810	A	G
rs2792563	1	213100225	A	G
rs28424875	4	42664508	C	A
rs55971447	1	161515326	T	C

rs72926952	2	203485086	T	G
rs2844804	6	29942126	G	T
rs3020726	2	87016506	G	A
rs2857229	12	6900616	G	A
rs2857229	12	6900616	G	A
rs28634695	15	40856113	A	G
rs28723652	14	106644507	A	C
rs35501848	17	62021003	T	G
rs709589	14	106306841	T	C
rs9639820	7	40094641	G	A
rs28669075	15	40856521	T	C
rs709589	14	106306841	T	C
rs28669075	15	40856521	T	C
rs28723652	14	106644507	A	C
rs35501848	17	62021003	T	G
rs709589	14	106306841	T	C
rs9639820	7	40094641	G	A
rs2949661	1	167424924	T	C
rs4657649	1	167173140	C	T
rs2949661	1	167424924	T	C
rs4657649	1	167173140	C	T
rs57319220	3	45919992	A	G
rs2949661	1	167424924	T	C
rs76540213	1	166035343	T	C
rs2949669	1	167417884	T	C
rs2988277	1	167431352	T	C
rs4657649	1	167173140	C	T
rs2988279	1	167419839	A	G
rs2995089	1	167431193	A	G
rs3020726	2	87016506	G	A
rs3087456	16	10970902	A	G
rs7032773	9	117645562	T	C
rs3087456	16	10970902	A	G
rs542160	11	107676543	T	C
rs72779785	16	10569039	G	A
rs3087456	16	10970902	A	G
rs55724950	16	10993712	A	G
rs62029480	16	10651993	T	C
rs7032773	9	117645562	T	C
rs3087456	16	10970902	A	G
rs72779785	16	10569039	G	A
rs3104369	6	32602482	C	T
rs3116493	2	204589281	T	G
rs75380201	18	39778311	T	C
rs3116493	2	204589281	T	G
rs3129891	6	32415080	A	G
rs9271771	6	32594274	C	T
rs3130685	6	31206206	T	C
rs3130685	6	31206206	T	C
rs3130685	6	31206206	T	C
rs36058358	6	32636282	A	G
rs4796089	17	33814758	C	T
rs3130685	6	31206206	T	C
rs3184504	12	111884608	C	T
rs3184504	12	111884608	C	T
rs9271622	6	32591845	A	G
rs3184504	12	111884608	C	T
rs34460712	2	38935236	A	G

rs3757247	6	90957463	T	C
rs6218398{	2	204609402	T	C
rs34546581	2	87021659	A	G
rs9270657	6	32566021	G	T
rs9461744	6	32388317	G	A
rs9461744	6	32388317	G	A
rs354020	19	49838891	A	C
rs3556746З	6	32633970	A	C
rs3557480З	3	32533246	C	T
rs4810485	20	44747947	G	T
rs3558726ʻ	3	46259296	G	A
rs6180233З	1	161589982	T	G
rs6687275	1	161515638	G	T
rs3793662	9	95886051	T	C
rs4129509З	10	6120173	A	G
rs3793662	9	95886051	T	C
rs4129493ʼ	10	6106621	C	T
rs7078614	10	6075831	T	G
rs3793662	9	95886051	T	C
rs6262632З	10	6115678	A	C
rs709589	14	106306841	T	C
rs7163297{	1	161526397	G	A
rs7163991(	1	161937774	A	G
rs7163297{	1	161526397	G	A
rs7163991(	1	161937774	A	G
rs3845548	1	161507448	T	C
rs3865444	19	51727962	A	C
rs5586250З	19	51858911	A	G
rs7351079	19	51574038	G	A
rs7894978ʼ	19	51591138	T	C
rs3865444	19	51727962	A	C
rs7425387(	18	5858361	T	C
rs3865444	19	51727962	A	C
rs5586250З	19	51858911	A	G
rs7351079	19	51574038	G	A
rs7894978ʼ	19	51591138	T	C
rs3865444	19	51727962	A	C
rs3865444	19	51727962	A	C
rs4499342	19	8786913	T	C
rs7351079	19	51574038	G	A
rs3865444	19	51727962	A	C
rs6211414ʼ	19	51588018	T	C
rs7351079	19	51574038	G	A
rs3865444	19	51727962	A	C
rs3865444	19	51727962	A	C
rs5586250З	19	51858911	A	G
rs7351079	19	51574038	G	A
rs7894978ʼ	19	51591138	T	C
rs3865444	19	51727962	A	C
rs4802858	19	52253150	C	T
rs5586250З	19	51858911	A	G
rs5848327ʻ	19	51868443	T	C
rs7351079	19	51574038	G	A
rs7894978ʼ	19	51591138	T	C
rs3917768	1	169572947	G	T
rs7526645	1	169728916	T	C
rs3934748	1	206879659	T	G
rs7189927	16	28913787	C	T

rs41294937	10	6106621	C	T
rs7078614	10	6075831	T	G
rs445	7	92408370	T	C
rs445	7	92408370	T	C
rs4371876	6	31067550	A	G
rs9357097	6	30285121	T	C
rs4493054	16	31361988	A	G
rs4987360	1	169664867	G	A
rs7139370	13	109976273	T	C
rs45608038	2	73679013	G	A
rs75766272	1	8009985	T	C
rs76477280	13	50147180	G	A
rs4657041	1	161478859	C	T
rs4683184	3	46187707	A	G
rs79825014	3	46880193	T	G
rs4785074	16	59885737	T	C
rs4796089	17	33814758	C	T
rs4796089	17	33814758	C	T
rs4796089	17	33814758	C	T
rs6904670	6	31192797	G	A
rs7728865	5	59819941	A	G
rs4796089	17	33814758	C	T
rs4796089	17	33814758	C	T
rs4849134	2	111615015	G	A
rs4987369	1	169662742	T	G
rs548316	11	128077683	A	G
rs55971447	1	161515326	T	C
rs75072970	1	161058351	C	T
rs55971447	1	161515326	T	C
rs56216383	2	204631712	G	A
rs56262516	6	29930939	A	G
rs7425430	2	87056480	G	A
rs9263601	6	31071758	C	T
rs56568421	10	68270803	A	G
rs58027502	5	139978241	T	C
rs58408589	16	31362741	G	A
rs61802329	1	161589626	T	C
rs75841521	2	59592691	A	G
rs78520150	1	19786101	A	C
rs79298699	16	31402042	T	C
rs597808	12	111973358	G	A
rs9271629	6	32591976	A	C
rs61126090	6	32586382	G	A
rs61141933	2	39388479	T	C
rs61839660	10	6094697	T	C
rs61839660	10	6094697	T	C
rs61839660	10	6094697	T	C
rs78667279	2	54957709	A	G
rs61839660	10	6094697	T	C
rs9268791	6	32421073	C	T
rs61839660	10	6094697	T	C
rs61839660	10	6094697	T	C
rs62183989	2	204609402	T	C
rs62184016	2	204623481	C	T
rs72928038	6	90976768	A	G
rs62184016	2	204623481	C	T
rs72928038	6	90976768	A	G
rs62325303	4	111000603	T	C

rs62395272	6	31394424	T	C
rs9271296	6	32581501	G	A
rs9271768	6	32594188	G	A
rs9271768	6	32594188	G	A
rs9296042	6	32736005	T	C
rs62447171	7	50309890	A	G
rs7658518	4	84160279	A	G
rs62447171	7	50309890	A	G
rs7658518	4	84160279	A	G
rs62447171	7	50309890	A	G
rs7658518	4	84160279	A	G
rs62447171	7	50309890	A	G
rs7658518	4	84160279	A	G
rs62626323	10	6115678	A	C
rs709589	14	106306841	T	C
rs6453485	5	79151752	T	C
rs7078614	10	6075831	T	G
rs7078614	10	6075831	T	G
rs6681289	1	119421209	A	G
rs6707421	2	38897781	G	A
rs6710361	2	87047145	A	G
rs9896155	17	33794697	G	A
rs6751481	2	38897810	C	T
rs6751481	2	38897810	C	T
rs6755452	2	87035076	G	T
rs9896155	17	33794697	G	A
rs6776036C	15	80260872	A	G
rs6808893	3	188133439	T	C
rs709589	14	106306841	T	C
rs71632979	1	161526397	G	A
rs7189927	16	28913787	C	T
rs7245846	19	51731176	A	G
rs7245846	19	51731176	A	G
rs7351079	19	51574038	G	A
rs78949787	19	51591138	T	C
rs7263597C	2	73868977	G	T
rs7263597C	2	73868977	G	T
rs72677097	1	61134076	G	A
rs72915529	2	54970478	A	G
rs9302635	16	72144174	C	T
rs73165129	22	42315371	T	C
rs73192651	13	50212885	T	C
rs73402222	6	31388449	C	T
rs73402222	6	31388449	C	T
rs74657611	4	53172274	A	G
rs75320486	1	206842241	A	G
rs7985659C	1	205241642	A	G
rs75766272	1	8009985	T	C
rs760462	21	46328099	C	T
rs760462	21	46328099	C	T
rs76112278	4	15915069	A	G
rs76161425	11	112704995	T	C
rs77650974	18	12394263	A	G
rs77650974	18	12394263	A	G
rs77789294	14	29178686	C	T
rs778582	5	140004816	T	C
rs7801342	7	31163003	C	T
rs78268116	11	112705025	T	C

rs78268116	11	112705025	T	C
rs9269109	6	32443266	C	T
rs879299	6	15897007	G	T
rs9270591	6	32561424	T	C
rs9271768	6	32594188	G	A
rs9271768	6	32594188	G	A
rs9268832	6	32427789	C	T
rs9269109	6	32443266	C	T
rs9270591	6	32561424	T	C
rs9271768	6	32594188	G	A
rs9271768	6	32594188	G	A
rs9275271	6	32662533	C	T
rs9916257	17	33797371	T	G

EA.outcome	NEA.outcome	BETA.exposure	BETA.outcome
T	C	0.4144	0.0691
T	C	0.389	0.0691
G	A	-0.1509	0.0701
T	C	0.1606	0.0047
T	C	-0.1389	-0.0662
A	G	0.2375	0.0744
A	G	0.167	-0.0963
G	A	-0.236	0.0608
T	C	0.1752	-0.0714
A	G	0.1788	0.1047
C	T	-0.2177	0.0187
A	G	359.1	0.0708
C	A	280.3	-0.0559
T	C	118.5	-0.1275
C	A	985.5	0.0591
T	G	407.5	0.0145
G	A	216.9	-0.0563
T	C	95.49	-0.1613
T	C	82	0.1028
T	G	303	0.1121
A	G	308.6	0.0262
C	T	196.5	-0.0034
A	G	206.9	-0.0301
G	A	237.6	0.2464
T	G	387.4	-0.0825
C	T	249.4	-0.0221
C	T	292.3	0.18
A	G	95.85	-0.0658
T	C	345.5	-0.0176
T	C	1015	-0.1165
A	G	206.8	-0.2543
A	G	312.7	-0.0614
G	A	386.6	-0.1356
T	C	214.8	0.0111
G	A	189.8	0.3645
A	G	337.1	0.111
A	G	324.4	-0.143
T	C	172.3	-0.0933
A	G	234.6	0.0351
G	A	725.8	-0.1551
A	G	662.4	-0.2204
T	C	139.3	-0.251
G	T	239.1	0.0469
T	C	160.1	0.6174
A	G	623.6	-1.0858
A	G	342.3	-0.0437
T	C	274.8	0.1591
G	T	192.2	-0.0103
A	G	221.5	-0.1298
C	T	177.5	0.1099
A	C	207.9	-0.5188
T	C	315.7	-0.1446
T	C	771.6	-0.1079
T	C	794.2	-0.1622
G	A	759.4	0.0147
G	A	571.9	-0.0127

A	G	896.2	-0.1558
A	G	889.9	-0.1426
C	T	159.2	0.4404
A	G	268.3	0.0344
C	A	297	-0.0642
C	T	1006	0.1416
A	G	-233.6	0.2413
T	G	420	-0.2509
G	A	834.4	-0.2166
T	C	126.2	0.0387
T	C	469.1	-0.0856
A	G	194.3	-0.1966
C	T	163.2	-0.0318
A	G	180.4	0.1677
T	C	255.1	0.2906
T	C	208.3	-0.3456
A	G	447.6	0.1016
T	C	378.1	0.0973
C	A	186	0.2007
T	C	437.7	0.1178
A	G	264.9	0.0801
C	T	375.1	0.1548
C	T	196.1	-0.4049
A	C	272.5	0.136
C	A	414.6	-0.0276
A	G	250.3	0.4328
G	A	215.2	0.1485
C	T	854.9	0.201
T	G	573	0.323
T	C	224.8	-0.2395
A	G	206.4	0.3356
C	T	141	-0.1023
A	C	246.8	0.0699
T	C	172.1	0.0588
G	A	785.2	-0.1064
A	C	111	0.0045
A	G	248.6	-0.3587
A	G	248.5	-0.3063
T	C	516.5	-0.0735
C	T	534.9	0.0977
C	T	76.42	0.0786
T	C	699.1	-0.5411
A	G	169.6	-0.2131
A	G	245.4	-0.0333
G	A	1129	0.172
A	G	620.5	0.0511
T	C	330.2	-0.049
A	G	166.6	-0.017
G	A	98.72	0.0821
G	A	84.59	-1.3127
T	C	417.3	0.0592
T	G	1000	0.2108
A	C	515.7	-0.0813
C	T	219.9	0.1595
G	A	244	0.1932
A	G	262.1	-0.383
A	G	187	-1.0989
A	G	1285	0.0887

A	G	139.5	-0.1171
T	C	419.3	0.0688
A	G	155.1	-0.005
A	G	200.7	-0.2164
C	T	570.9	0.1111
T	C	842.4	0.0271
A	G	257.5	0.3457
A	G	59.98	0.2182
C	T	836.1	-0.289
G	A	516.8	0.0299
A	G	381.7	0.6836
A	G	172.5	0.1835
T	C	443.1	0.0348
T	C	176.3	-0.291
A	C	199.4	0.0858
A	G	204.4	0.0667
C	T	460.1	0.1411
T	C	69.9	-0.162
T	C	210.6	0.0934
T	C	2073	0.0344
A	G	636.7	-0.2095
A	G	181.9	-0.0565
A	C	274.8	0.1674
C	A	216.5	0.0595
C	T	440.2	0.1434
T	C	394.5	-0.3114
C	A	127.1	-0.1824
T	C	189.1	0.2418
T	C	1232	0.3383
A	G	279.2	0.1539
T	C	328.1	0.161
G	A	111.8	-0.0015
A	G	43.94	0.134
A	G	591.4	-0.3
T	G	494.7	0.3245
G	A	369.2	0.1453
G	T	730	0.2811
C	T	159.5	0.1884
A	G	144.1	-0.102
T	C	210.7	-0.1441
A	G	77.03	0.1113
T	C	186.1	0.0408
G	A	185.9	0.0402
C	T	465.8	-0.2247
C	T	202.4	-0.0915
C	T	124.1	0.382
G	T	479.2	0.014
A	C	207	-0.4634
A	C	417.2	0.6499
T	C	235.3	0.2146
C	T	143.9	-0.2957
C	A	225.4	-0.2711
A	G	1165	0.0075
A	G	355.7	-0.2667
A	G	338.4	0.0439
T	C	152.8	-0.0069
T	C	313.5	0.1209
G	T	399.5	0.1423

T	C	181.2	0.0629
T	C	549.6	-0.0402
T	C	826.8	0.2022
G	A	301	-0.0339
T	C	114.2	0.344
A	G	123.5	0.4332
G	A	183.9	0.4682
T	C	149.3	0.1368
T	C	282.1	0.311
A	G	262.7	-0.0385
T	C	2047	0.0485
G	A	438.9	0.1232
T	C	751.9	-0.1653
G	A	266.7	-0.1099
T	C	257.4	0.0122
C	A	1445	0.1675
G	A	159.7	-0.2337
T	C	708.1	0.0426
G	A	203.9	0.0008
T	C	339.6	0.2622
A	G	240.6	0.0934
T	C	453.6	-0.3907
C	T	562.4	0.0619
T	C	416.3	-0.1175
T	C	496.1	-0.1546
A	G	262.4	0.1494
T	C	299.6	-0.1263
A	G	432.3	0.0257
T	C	163.3	0.0986
C	T	829.8	-0.0342
A	G	311	0.3713
T	C	316	-0.1059
A	C	731.4	1.3811
A	G	731.4	-0.0303
A	G	331.7	-0.1567
T	C	320.1	0.1897
A	G	2073	-0.5947
C	T	150.2	0.0502
C	T	751.8	-0.1313
A	G	235.6	-0.1457
T	C	121.9	-0.0686
C	A	282.9	-0.147
C	A	301.9	0.0064
A	G	489.9	0.0984
A	G	628.3	0.1681
T	C	137	0.1857
A	G	253.3	0.2472
T	C	230.6	0.0152
T	G	306.1	0.0746
T	C	181.1	-0.0022
A	G	271.1	0.008
A	C	548.1	-0.4947
A	G	887.3	0.2218
G	A	300.6	0.2776
C	A	750.9	-0.3545
G	A	428.3	-0.1324
A	G	1200	-0.0325
A	G	246	0.0308

T	C	413.3	0.0757
T	C	159.6	-0.093
T	C	175.4	0.3781
T	C	358.2	-0.0185
T	C	988.1	0.425
T	C	122.6	-0.7748
G	A	1581	-0.0876
C	A	177.1	0.1569
A	C	580.3	0.0594
T	C	436.5	-0.1868
A	G	189.6	-0.2619
A	G	319.6	-0.1343
G	A	156.1	0.0201
G	A	-388.2	0.0317
T	C	164.3	-0.083
A	C	395.7	-0.1795
A	G	211.7	-0.248
A	G	1008	0.0213
T	C	270.7	-0.5055
G	A	542.5	-0.0827
T	C	336.7	-0.0125
T	C	475	0.0781
A	G	158.9	0.1527
C	A	196.4	0.0171
G	A	257	-0.2276
G	T	197.3	-0.2209
G	T	353.7	-0.0395
T	C	286.8	0.0612
C	T	353.5	0.0027
T	C	212.6	-0.0049
A	G	330.6	-0.2073
C	T	214.7	-0.0386
G	A	160.9	0.5836
G	A	398.3	-0.2987
A	G	149.1	-0.0221
T	C	211.5	0.239
C	T	360.9	0.0185
A	G	255.1	-0.081
T	C	1757	0.0573
G	T	235.6	0.03
A	G	263	-0.106
C	T	-548.4	0.2554
T	C	466.1	0.0024
A	G	584.1	0.11
T	C	397	0.0146
A	G	643.8	-0.3837
G	A	424.9	0.0171
C	T	381.2	0.0198
A	G	520	-0.1228
T	C	584.7	-0.3072
G	A	1003	0.0185
C	T	568.9	-0.0952
T	C	250	-0.0945
G	T	204.6	-0.1712
A	G	130.7	0.0088
C	T	218.4	0.0674
T	C	166.4	0.1597
G	A	658.4	0.0624

T	C	125.6	-0.0257
A	G	90.23	-0.0883
T	G	442.9	-0.087
G	T	184.9	-0.119
A	G	658.4	0.4389
A	G	212.8	0.0467
C	T	104.8	-0.0982
A	G	269.8	0.0686
T	C	416.6	-0.1517
A	G	939.1	0.2586
C	T	239.6	-0.1121
T	C	207	-0.1333
G	A	358.8	0.2887
T	C	353	-0.0099
T	G	559.9	-0.003
G	T	559.6	0.2817
T	C	605.2	0.2562
A	G	632.9	0.147
A	G	185	0.1223
A	G	348.1	-0.1075
A	C	851.2	0.2378
A	G	-87.22	-0.2222
G	A	-287.7	-0.0053
T	C	335	-0.1137
A	G	2896	0.1059
A	G	130.9	-0.0541
A	G	2721	0.2602
T	C	203	-0.1183
A	C	106.8	-0.3843
T	C	634.9	0.2334
C	T	134.6	0.1004
C	A	364.4	-0.1119
T	C	116.6	0.068
G	A	475.9	-0.2022
A	C	278.8	-0.2466
C	T	404.4	0.0071
A	C	336.9	0.2061
A	G	438.9	-0.6117
G	A	413.6	0.1407
A	C	287.4	0.3767
G	A	264	-0.2102
T	G	322	0.0584
A	G	308.9	0.1034
A	C	326.1	0.2608
T	C	268.4	0.0381
T	C	188.4	0.0907
C	A	338.9	0.2102
G	T	217.6	0.0963
A	G	317.1	-0.12
A	G	105.3	-0.2517
A	G	352	-0.2332
A	G	101.4	0.1958
T	C	225.9	-0.0152
A	G	132.5	0.0559
G	A	143.3	-0.1165
G	T	183	-0.2947
C	T	97.68	-0.329
A	G	287	-0.1551

C	T	1057	0.007
C	T	212.5	-0.062
C	T	3549	0.1092
C	T	303.6	0.0619
C	A	297	-0.2419
A	C	434.2	0.1958
T	C	263.6	0.1284
C	T	331.8	-0.0967
T	C	1584	-0.1626
A	C	961.3	0.1518
C	T	857.2	0.3525
C	T	163.6	0.001
T	C	1574	0.1153
C	T	1117	-0.2919
A	G	141.4	-0.0806
A	G	99.34	-0.1182
A	G	1826	-0.0768
T	G	47.59	-0.1277
T	C	774.8	-0.2324
G	T	404.7	0.5388
T	C	168.6	0.1438
G	A	391.1	0.308
G	A	301.1	0.0861
T	C	85.79	0.0868
G	A	193.7	0.1272
T	G	0.1206	-0.0362
G	A	0.3952	-0.181
G	T	-12.04	0.2093
T	C	-0.2955	-0.0034
T	C	0.1984	0.0203
A	C	-0.2799	0.1297
A	G	-0.3127	0.1634
A	G	0.1591	0.0428
C	T	0.192	-0.0772
A	G	-0.3411	-0.366
G	A	-0.5168	-0.0378
G	T	0.234	-0.005
A	G	-0.1677	-0.0443
T	C	-0.2444	0.0679
T	C	-0.5713	0.0067
T	C	-0.2765	-0.1022
C	T	0.1554	-0.0256
A	G	-0.1391	-0.0538
T	C	-0.2991	0.1394
A	G	0.2182	-0.5309
G	A	0.207	0.0253
A	G	0.1894	-0.0394
C	T	-0.3215	0.0549
T	C	0.2358	0.0366
C	A	-0.1728	-0.1029
C	A	18.81	0.0591
T	G	9.768	0.0145
A	G	26.82	0.3903
A	G	9.265	-0.143
G	A	8.874	0.2249
A	G	17.96	-0.1426
C	T	22.4	0.1416
T	C	10.65	-0.1832

G	A	19.07	-0.2166
A	G	18.77	0.0509
T	C	32.87	-0.1481
T	G	33.95	0.0856
A	G	36.56	0.2356
T	C	12.62	-0.0735
T	C	16	-0.5411
G	A	25.15	0.172
A	G	10.8	0.0511
T	G	23.37	0.2108
A	G	21.5	0.0887
T	C	23.94	0.0271
T	C	33.4	0.0344
T	C	51.17	0.3383
T	G	42.63	0.3025
T	C	32.81	0.0485
C	A	25.69	0.1675
A	G	31.1	-0.1105
A	G	33.31	-0.5947
C	T	15.79	-0.1313
A	G	12.36	0.0984
A	G	18.17	0.2218
G	A	8.527	0.2776
T	C	4.47	-0.7748
G	A	35.37	-0.0876
A	C	12.51	-0.1795
G	T	53.41	0.1019
T	C	38.7	0.0486
G	A	4.521	0.2592
G	T	9.274	-0.0395
T	C	49	0.0573
C	T	33.05	-0.1134
G	A	31.04	0.0185
A	G	38.95	0.2586
C	T	38.22	0.0408
A	G	51.58	0.1059
A	G	49.64	0.2602
G	A	52.91	0.2319
G	A	13.03	0.1407
T	C	34.38	0.0569
G	A	19.81	0.2067
A	G	23.9	0.2363
C	T	100.8	0.1092
C	T	9.036	-0.0967
T	C	26.23	-0.1626
C	T	24.55	0.3525
T	C	37.38	0.1153
A	G	35.15	-0.0768
G	A	44.6	-0.2206
T	C	0.2345	0.0994
T	G	-0.1918	0.0148
T	C	0.249	0.0994
T	C	-0.408	0.2087
T	G	-0.2323	0.0148
T	C	-0.2345	0.0994
T	G	0.1918	0.0148
A	G	-0.1062	-0.0899
G	A	-0.1633	-0.088

A	G	-0.2753	0.0045
A	G	-0.2188	-0.0383
C	A	-0.4118	-0.0083
T	C	-0.1846	0.0511
T	G	0.5248	0.3729
G	A	1.011	-0.0167
T	C	0.4486	-0.0398
A	G	-0.1827	-0.0383
C	A	-0.3962	-0.0083
T	C	-0.1937	0.0511
G	T	1.014	-0.0166
T	C	0.4759	-0.0398
G	A	-0.5171	-0.4266
A	G	-0.189	-0.0383
C	A	-0.3631	-0.0083
C	A	-0.2494	-0.0385
T	G	0.8768	-0.0165
T	C	0.3373	-0.0398
A	G	-0.1864	-0.0383
C	A	-0.3538	-0.0083
T	C	-0.1886	0.0511
T	G	0.4456	0.3729
G	A	0.9156	-0.0158
T	C	0.3615	-0.0398
C	T	0.1739	0.0317
A	G	0.8979	-0.1854
C	T	0.5908	-0.8331
A	C	0.2681	0.1138
G	A	0.4907	-0.0167
A	G	-0.5202	0.1813
A	G	-0.7129	0.1523
G	A	-0.5047	-0.0619
T	C	-0.607	0.1328
A	G	-0.472	-0.002
T	C	0.2286	-0.05
T	C	-0.7454	0.1686
A	G	-0.5412	0.1813
A	G	-0.7379	0.1523
G	A	-0.5355	-0.0619
T	C	-0.5121	0.1328
T	C	0.2107	-0.05
A	G	-0.4487	-0.0126
T	C	-0.6949	0.1686
A	G	-0.5028	0.1813
A	G	-0.7505	0.1523
G	A	-0.5223	-0.0619
G	A	-0.1765	-0.025
T	C	-0.5308	0.1328
T	C	0.2184	-0.05
A	G	-0.4356	-0.0126
T	C	-0.7149	0.1686
A	G	-0.5131	0.1813
A	G	-0.7498	0.1523
G	A	-0.5364	-0.0619
G	A	-0.1694	-0.025
T	C	-0.5248	0.1328
T	C	0.218	-0.05
A	G	-0.4367	-0.0126

T	C	-0.7095	0.1686
A	G	-0.4975	0.1813
A	G	-0.763	0.1523
C	T	-1.509	-0.0567
G	A	-0.5384	-0.0619
G	A	-0.1805	-0.025
T	C	-0.532	0.1328
T	C	0.2191	-0.05
A	G	-0.4296	-0.0126
T	C	-0.7283	0.1686
A	G	-0.5029	0.1813
A	G	-0.7531	0.1523
G	A	-0.5248	-0.0619
G	A	-0.1866	-0.025
T	C	-0.5235	0.1328
T	C	0.2161	-0.05
A	G	-0.4336	-0.0126
T	C	-0.7259	0.1686
A	G	-0.5326	0.1813
A	G	-0.7406	0.1523
C	T	-1.496	-0.0567
G	A	-0.5338	-0.0619
G	A	-0.1724	-0.025
T	C	-0.5142	0.1328
T	C	0.2136	-0.05
A	G	-0.4435	-0.0126
T	C	-0.7056	0.1686
A	G	-0.4717	0.1813
A	G	-0.6734	0.1523
G	A	-0.4771	-0.0619
T	C	-0.4832	0.1328
T	C	0.2113	-0.05
A	G	-0.4046	-0.0126
T	C	-1.385	-0.0615
T	C	-0.6664	0.1686
A	G	-0.4922	0.1813
A	G	-0.7389	0.1523
G	A	-0.4826	-0.0619
T	C	-0.5142	0.1328
C	T	-0.1522	-0.0249
T	C	0.2246	-0.05
A	G	-0.4284	-0.0126
G	A	-1.434	-0.0605
T	C	-0.7005	0.1686
G	A	-0.1328	-0.0267
A	G	-0.4611	-0.0987
A	G	0.1693	-0.0904
T	C	-0.1471	-0.0706
A	G	0.4987	-0.0135
C	A	-0.4034	-0.0083
T	G	0.5578	0.3729
A	G	1.039	-0.0221
A	G	-0.1833	0.0559
G	A	0.562	0.0331
A	G	0.4553	-0.0135
C	A	-0.4069	-0.0083
G	A	-0.4298	0.1344
C	A	-0.2549	-0.0385

T	C	-0.2387	0.0141
T	C	0.1594	-0.0566
C	A	-0.9707	0.0187
A	G	0.4441	-0.0135
C	A	-0.3969	-0.0083
T	C	0.9616	-0.0221
T	C	-0.2419	0.0141
G	A	0.6746	-0.3269
G	A	-0.5209	-0.4266
A	G	0.5396	-0.0135
C	A	-0.4485	-0.0083
G	A	-0.4197	0.1344
T	G	0.6399	0.3729
T	C	0.2158	-0.0566
A	G	0.269	-0.0401
G	A	-1.111	0.0215
G	A	0.6144	0.0331
A	G	-0.2945	0.0158
G	A	0.2076	-0.0379
T	C	-0.173	-0.0186
C	T	-0.2226	-0.022
A	G	-0.2588	0.0786
T	C	0.5797	0.0324
T	C	-0.4505	-0.0072
C	A	-0.3837	-0.0083
T	C	-0.2329	0.0141
A	G	0.2703	-0.0195
A	G	-1.026	0.0215
C	A	-0.4542	-0.0083
C	T	0.3052	-0.0194
T	C	-0.2642	0.0141
G	A	0.569	-0.3269
T	C	1.175	-0.0167
G	T	-0.4413	-0.2293
C	A	-0.3988	-0.0083
T	C	1.015	-0.0221
C	T	0.2849	-0.0194
T	C	-0.2442	0.0141
C	T	-0.22	0.0092
C	A	-0.1936	-0.0083
A	G	0.1839	-0.0195
C	A	-0.1597	-0.0083
A	G	0.6927	-0.1854
C	T	0.5196	-0.8331
T	C	0.5445	-0.0167
G	A	0.3005	-0.1348
C	A	-0.3997	-0.0083
T	G	0.5572	0.3729
A	G	-0.2053	0.0559
C	A	1.021	-0.0162
T	C	0.4246	-0.0398
G	A	0.2178	-0.0329
G	A	0.5089	-0.025
C	A	0.3665	0.1295
A	G	0.715	0.0212
T	C	-0.4537	0.0044
A	G	0.1841	-0.032
A	C	-0.2138	-0.0103

	A	G	1.418	-0.0066
	G	A	0.6318	0.0529
	A	G	0.1734	-0.032
	A	C	-0.1952	-0.0103
	A	G	1.391	-0.0066
	G	A	0.6084	0.0529
	A	G	0.2281	-0.032
	C	T	-0.2555	0.0701
	T	C	-0.5632	0.2639
	T	G	0.3221	-0.0482
	T	C	0.2856	-0.0398
	T	C	-0.7822	-0.0882
	A	G	-0.4696	0.1655
	T	C	-1.076	0.1588
	C	T	0.238	0.0701
	A	G	0.2608	-0.0365
	A	G	-0.4567	0.1597
	G	A	0.273	0.0122
	T	C	0.2106	0.0529
	A	G	-0.143	-0.0163
	G	A	-0.2111	-0.025
	C	T	-0.4707	-0.0003
	G	A	-0.2059	-0.0475
	C	T	0.6071	0.2704
	A	G	0.4158	-0.3229
	T	C	0.1497	0.1056
	G	A	-0.3203	0.1054
	A	G	0.6796	-0.1088
	A	G	-0.5728	-1.0664
	C	T	0.5924	0.0964
	G	A	0.3834	-0.025
	T	C	-0.4684	-0.194
	C	T	-0.1674	0.113
	C	T	-0.2433	0.113
	T	C	-0.3589	0.1071
	A	G	0.4117	0.6199
	C	T	0.3633	-0.0623
	T	C	-0.4126	0.1071
	C	T	-0.2424	0.0369
	A	G	0.3935	0.6199
	C	T	0.3702	-0.0623
	T	C	-0.1548	0.031
	C	T	-0.1904	-0.0129
	T	C	0.199	0.0529
	T	C	0.2563	0.1138
	C	T	-0.5071	-0.0003
	A	G	-0.213	0.0648
	C	T	0.6708	0.2704
	A	G	0.4286	-0.0516
	C	T	-0.4093	0.0855
	A	G	0.4366	-0.3229
	T	C	0.256	0.0529
	T	C	0.2579	0.1138
	A	G	-0.1453	-0.0163
	G	A	-0.2208	-0.025
	C	T	-0.5841	-0.0003
	A	G	-0.2616	0.0648
	A	G	-0.4501	0.2157

C	T	0.7589	0.2704
A	G	0.5011	-0.3229
A	G	0.2196	-0.1401
C	T	0.1525	-0.0276
T	C	0.1988	0.0529
G	A	0.2712	0.0121
T	C	0.2484	0.1138
G	A	-0.1586	-0.025
A	G	-0.2612	0.0648
C	T	0.7465	0.2704
G	T	-0.4783	-0.0022
A	G	0.4379	-0.0516
T	C	-0.1755	0.0067
A	G	0.5086	-0.3229
T	C	0.2761	0.0529
G	A	0.2781	0.0121
T	C	0.299	0.1138
A	G	-0.1635	-0.0163
G	A	-0.2933	-0.025
C	T	-0.5853	-0.0003
A	G	-0.3147	0.0648
A	G	-0.5359	0.2157
C	T	0.6995	0.2704
A	G	0.478	-0.3229
T	C	0.2742	0.0529
T	C	0.264	0.1138
A	G	-0.1634	-0.0163
G	A	-0.2036	-0.025
C	T	-0.6404	-0.0003
A	G	-0.2122	0.0648
A	G	-0.534	0.2157
C	T	0.6579	0.2704
A	G	0.4177	-0.3229
T	C	1.244	-0.3159
A	C	0.1345	-0.0103
A	G	-1.081	-0.0066
G	A	-0.1902	-0.0263
A	C	-0.1861	-0.0103
A	G	1.342	-0.0066
G	A	0.2347	-0.0263
A	C	-0.2555	-0.0103
A	G	1.499	-0.0066
A	C	1.586	-0.1549
C	A	0.698	0.2014
G	A	0.2545	-0.0398
T	C	0.1756	-0.0409
T	C	-0.2878	0.1861
C	T	-0.283	0.0549
T	C	0.2138	0.0355
T	C	0.2943	0.1406
C	A	4.463	-0.1975
T	C	3.436	0.2919
G	A	3.787	0.252
C	T	4.765	-0.0767
G	T	7.992	0.2488
G	T	4.272	-0.1323
G	A	0.4024	-0.181
T	C	9.1	-0.0736

T	C	8.74	0.1925
T	C	1.818	0.1817
A	G	3.894	-0.001
A	G	3.783	0.1117
C	T	7.82	-0.0446
G	A	6.643	-0.1189
C	T	3.974	-0.5766
A	G	3.794	-0.1784
T	C	3.814	-0.1469
G	A	3.783	0.0676
A	C	3.717	-0.1498
G	A	3.946	0.0681
T	C	4.893	-0.0233
T	C	7.599	0.2777
G	A	6.493	0.0959
G	A	4.52	-0.0712
A	G	3.533	-0.4489
G	A	4.848	0.1309
C	T	4.025	-0.0381
C	T	13.7	0.311
A	C	4.11	-0.3519
G	A	3.855	-0.0089
G	A	4.877	0.0186
T	C	3.833	-0.0254
G	A	3.755	-0.1125
G	T	4.68	0.0418
A	C	0.4352	0.0414
A	G	-0.2839	-0.0759
C	T	-0.2551	-0.0218
T	C	0.1925	-0.101
A	G	-0.1653	0.0448
A	G	-0.3745	-0.0168
A	C	-1.218	-0.0063
T	C	0.2088	0.017
A	G	-0.4161	0.2142
C	T	-0.5777	0.0869
A	G	26.26	0.289
A	G	23.77	0.2321
C	T	32	-0.2605
T	C	30.16	0.0121
A	C	47.98	-0.0662
A	G	9.757	-0.0863
G	A	16.44	0.5879
A	C	24.71	-0.0203
T	G	15.84	0.9243
A	C	5.388	0.0924
A	G	1.086	0.0781
A	G	4.44	0.0395
C	T	20.33	-1.131
G	A	19.07	0.0212
T	C	20.95	-0.3145
G	A	4.577	-0.0343
A	C	22.84	-0.1276
A	G	22.84	-0.3344
A	G	24.63	-0.1327
T	C	12.63	0.0359
T	C	9.254	-0.4613
G	A	18.3	-0.5194

C	A	20.46	0.1308
T	G	25.81	-0.0224
T	C	11.36	0.1358
C	T	31.35	-0.2993
T	C	52.08	0.0539
A	G	29.68	-0.0324
T	C	29.98	0.2017
T	C	7.048	0.2672
C	T	20.97	-0.1165
C	T	28.44	-0.0748
C	T	10.98	0.4724
G	A	6.353	0.0252
A	G	18.92	-0.1028
G	T	10.56	0.4465
T	C	303.3	0.1868
G	A	137.6	-0.4335
C	T	123.8	0.0011
T	C	87.63	0.1486
A	G	444.1	0.0753
T	C	286.9	0.0458
T	C	345.8	0.3121
T	C	442.7	-0.143
T	C	591	0.0121
A	G	176.7	0.0351
A	C	979.6	-0.0662
A	C	436	0.0411
A	G	450.6	-0.404
G	A	281.9	-0.0127
A	G	213	-0.0863
C	T	384.3	0.1416
C	T	647.9	-0.0763
G	T	172.7	0.4063
G	A	84.27	-0.404
G	A	159.7	0.188
A	G	201.5	0.2254
T	C	270.5	-0.0823
A	G	192.8	-0.0185
C	T	364.8	0.0101
A	C	472.2	-0.0203
G	A	385.7	-0.0395
A	C	132.8	0.136
C	A	462.3	-0.3403
T	G	232.5	0.323
G	A	2185	0.1378
A	G	189.7	-0.2051
A	G	22.81	0.0781
A	G	1678	0.1196
A	G	641.1	-0.0635
T	C	111	0.1377
T	C	791.9	-0.2184
A	G	463.8	-0.1144
C	T	259.1	-0.027
T	C	71.36	0.1399
G	T	550.9	-0.1257
C	T	151.8	-0.2394
T	C	142.3	0.031
T	C	161.7	0.2169
T	C	760.9	0.0344

A	G	556.4	-0.4122
T	C	259.6	-0.2455
T	C	595.1	-0.2109
A	G	320.2	0.1649
T	C	251.2	-0.2508
G	A	725.9	-0.1614
T	C	429	0.0647
A	G	1279	-0.3027
T	C	148.2	0.0408
T	G	285.3	-0.0324
T	C	751.4	0.0485
G	A	118.8	-0.0343
A	G	913.1	-0.0463
A	C	473.4	-0.1276
A	G	473.4	-0.3344
C	T	61.29	0.1703
A	G	352.4	-0.0543
C	A	530.8	-0.1576
T	C	337.2	-0.3267
A	G	402.2	-0.0997
A	G	613.8	0.4768
C	A	416.3	0.3139
A	G	760.5	-0.5947
C	T	-22.92	0.0378
G	A	101.4	-0.1244
T	G	328.7	0.0732
C	A	1005	-0.0016
A	G	332.4	-0.2739
A	G	402.2	0.07
G	T	654.7	-0.4367
A	C	138.5	-0.0784
T	C	317.4	0.0359
T	C	293.3	-0.1433
T	C	-397.5	0.0157
C	T	256.7	-0.0422
A	G	420	0.0213
A	G	416.6	-0.1032
A	G	557.6	0.0283
T	C	548.7	0.0162
T	G	721.2	-0.0224
A	C	404.2	-0.0216
C	T	178.9	0.1005
C	T	644.1	-0.2993
C	T	296.2	-0.1171
T	C	1065	0.0539
A	G	251.6	-0.0235
A	G	610.4	-0.0324
T	C	591.6	0.2017
T	C	351.9	1.7493
C	T	375.7	-0.0816
C	T	185.1	-0.2167
C	T	319.5	-0.1491
T	C	485.7	-0.1325
C	A	591.3	0.0528
T	C	123.2	-0.0092
A	G	405.6	0.4389
T	C	88.88	-0.4706
C	T	538.9	-0.0748

A	G	283	-0.1167
A	G	186.4	-0.0779
C	T	224.8	0.4724
G	A	154.5	0.0252
G	T	147.3	0.0979
A	G	187	0.0075
G	A	285.1	-0.4718
A	G	379.6	-0.1417
G	T	109	-0.1946
C	T	358.8	-0.0565
A	G	263.3	-0.1551
C	A	74.28	0.3255
A	C	389.2	-0.1343
A	G	239.7	0.1156
T	C	521.4	0.1153
A	G	397.6	-0.181
C	T	514.7	0.0161
T	G	368.8	0.0741
T	C	128.8	0.0481
A	G	1621	-0.0955
T	C	0.4119	0.0484
A	G	-0.3125	-0.2047
C	T	-0.3497	-0.0851
G	A	0.5292	-0.025
C	A	0.4042	0.1295
A	G	0.189	-0.0195
G	T	0.339	0.0272
A	G	0.7089	0.0212
T	G	0.8581	0.3639
A	G	1.128	-0.0066
T	C	0.7676	-0.2008
C	T	0.7376	-0.4351
C	T	0.7229	0.0212
T	G	0.4997	0.0804
A	C	0.2846	-0.0494
A	G	0.8891	-0.0204
A	G	0.6555	0.3799
T	G	0.7307	0.3639
T	C	1.27	-0.4383
T	C	0.6769	-0.2008
C	T	0.8332	-0.8331
C	T	0.2205	-0.0194
C	T	0.6237	0.0212
T	C	1.01	-0.0165
A	G	0.6459	-0.0997
T	G	0.7282	0.3639
T	C	0.7156	-0.2008
C	T	0.8528	-0.8331
C	T	0.2118	-0.0194
C	T	0.6424	0.0212
A	G	0.6785	-0.0997
C	T	1.01	-0.0163
T	G	0.8056	0.3639
A	G	1.718	-0.0066
C	T	1.311	-0.1383
T	C	0.6998	-0.2008
A	G	-0.1612	0.0768
G	A	0.201	-0.0263

C	T	0.1965	-0.0194
T	C	-0.2216	0.0141
C	T	0.6853	0.0212
A	C	0.5464	-0.0086
A	C	0.7843	0.1286
T	C	0.3811	-0.0312
A	G	0.8426	-0.0204
A	G	0.6067	0.3799
T	G	0.7719	0.3639
A	G	1.608	-0.0066
C	T	1.189	-0.1383
A	G	-0.1566	0.0768
G	A	0.1912	-0.0263
C	T	0.6358	-0.4351
T	G	0.5292	0.0804
A	C	0.7461	0.1286
T	C	0.3601	-0.0312
A	G	0.7212	-0.1309
T	C	0.4336	-0.0398
A	G	0.5828	0.3799
T	G	0.7675	0.3639
T	C	0.6977	-0.2008
C	T	0.8186	-0.8331
C	T	0.6402	0.0212
A	G	0.2205	-0.0195
T	C	-0.9998	0.0158
A	G	0.6238	-0.0997
A	G	0.1981	0.0002
T	C	0.1993	-0.0825
C	T	-0.1576	-0.0885
A	C	-0.1549	0.0137
A	C	-0.5454	0.0862
A	G	0.4881	-0.0202
T	C	0.5176	-0.2186
A	G	-1.131	0.0745
G	A	-0.3642	-0.0199
A	G	-0.4918	0.0656
A	G	0.3421	0.1288
A	C	-0.5128	0.0862
T	C	0.5046	-0.2186
A	G	-1.115	0.0745
G	A	-0.3351	-0.0199
A	G	-0.5252	0.0656
A	G	0.3376	0.1288
A	C	0.3963	0.0862
T	G	-0.5579	-0.0484
T	C	-0.3659	0.0711
C	T	-0.1937	0.0899
A	G	0.6185	0.0203
C	T	0.4823	0.0434
T	C	0.1921	-0.0023
A	G	0.3184	-0.0121
G	T	-0.4992	0.2807
T	C	0.1757	-0.0409
T	C	-0.2657	0.1705
C	T	-0.3192	0.0549
T	C	0.2688	0.0366
G	T	0.2058	0.0251

C	A	-0.1886	-0.1029
G	A	0.2372	-0.395
T	C	-0.2152	0.0222
T	C	-0.6028	1.0122
C	T	-0.6486	-0.8331
C	T	-0.2986	-0.0204
T	C	-0.7175	-0.0146
A	G	-0.4818	-0.1309
A	G	0.5134	0.2362
A	G	0.2343	-0.198
A	G	0.4732	0.2362
G	A	0.3984	-0.1522
A	G	0.177	-0.0548
G	A	0.1522	-0.012
A	G	-0.3007	0.1047
C	T	-0.3957	0.0795
A	G	-0.4296	0.3073
A	G	0.1738	-0.0569
A	G	-0.3894	0.0287
C	T	-0.4789	0.1065
A	G	-0.7731	-0.0375
A	G	0.5573	-0.1516
A	T	0.4094	-0.0422
G	A	0.4094	-0.0124
A	G	-0.2166	0.1047
G	T	0.1903	-0.0657
T	G	0.2713	-0.0482
G	A	0.6523	0.0154
A	G	-0.7478	0.1574
A	G	0.4508	0.0375
A	G	0.2604	-0.7219
T	C	-0.2525	0.161
T	C	0.2282	0.0355
T	C	-0.2404	0.0548
A	G	0.5594	0.2651
G	A	0.5583	0.0069
A	G	0.5079	-0.1535
A	G	0.5373	0.2651
G	A	0.167	-0.0189
T	C	-0.1982	-0.2846
A	C	0.3593	0.0624
G	T	-0.2439	0.0468
T	C	-0.6123	0.0067
C	T	-0.221	0.0336
A	G	-0.1525	0.0645
T	G	0.2378	0.0151
A	G	-1.573	-0.0066
G	A	-0.1572	-0.0263
A	C	-1.565	-0.1549
C	A	-2.923	0.3771
G	T	-2.662	-0.0312
T	C	0.4097	0.1293
T	C	0.1992	-0.0597
A	G	-0.2422	-0.1754
A	G	0.5885	0.0145
A	C	3.334	0.0887
A	G	1.176	-0.0066
C	T	1.018	-0.1383

G	A	8.184	-0.0077
G	A	0.1631	-0.0263
A	C	0.1398	0.0012
T	C	3.341	-0.1508
G	A	-0.5111	0.1054
A	G	0.8426	-0.1088
A	G	-0.3359	0.1138
C	T	0.7751	0.0964
G	A	0.429	-0.025
T	C	-0.6011	-0.194
T	G	-0.7116	0.0076
G	A	-0.6409	-0.2302
G	A	-0.446	0.1054
A	G	0.8229	-0.1088
A	G	-0.3227	0.1138
C	T	0.7181	0.0964
G	A	0.3944	-0.025
T	C	-0.5571	-0.194
G	A	-0.6492	-0.2302
C	T	1.189	0.0917
C	T	1.068	0.0917
G	T	0.34	-0.0365
G	A	0.2016	0.0394
A	G	0.4676	-0.1398
A	C	-0.3487	-0.0063
A	G	0.3805	-0.038
T	C	-0.4081	0.2937
G	A	0.3169	-0.3704
T	G	0.2185	-0.1708
G	A	0.1457	-0.0263
G	A	0.307	-0.3704
C	T	0.2974	-0.3704
C	A	0.1793	-0.0284
G	A	-1.226	-0.0665
G	A	-1.414	-0.0665
A	C	-1.288	-0.1549
T	C	0.3476	0.0433
T	C	0.4707	-0.1731
A	G	0.2019	-0.0575
G	A	-0.4215	-0.0605
G	A	0.6788	0.0069
A	G	0.6138	-0.1535
T	G	0.6504	0.2651
G	A	0.5095	0.0069
T	G	0.5195	0.2651
G	A	0.5863	0.0069
A	G	0.539	-0.1535
G	A	0.5582	0.2483
G	A	0.7181	0.0069
A	G	0.6567	-0.1535
G	A	0.7788	0.2483
G	A	0.5314	0.0069
G	A	0.5237	0.2653
A	G	0.281	0.0035
G	A	0.5404	0.0069
A	G	0.4919	-0.1535
G	A	0.5735	0.2483
G	A	0.5546	0.0069

G	A	0.148	-0.0189
G	A	0.5738	0.2483
A	G	-0.5378	0.0318
G	A	0.2522	-0.0422
T	C	0.3671	0.3901
A	G	1.049	-0.1496
C	T	-0.4511	0.057
C	T	53.93	-0.2605
A	G	2.545	0.0781
G	A	28.75	-0.0216
C	T	-2.715	0.0378
C	T	2.095	0.1422
A	G	26.32	-0.1973
C	A	-1.704	-0.0492
C	T	18.44	-0.2167
T	C	14.88	0.2672
T	C	2.421	-0.2453
A	C	-0.4855	0.0895
T	C	-0.5045	-0.0432
T	C	2.431	-0.2453
T	C	-0.5108	-0.0432
A	G	-0.6777	0.1699
T	C	0.6149	0.0324
C	T	0.3533	0.0434
A	G	-0.6462	0.1699
A	G	-0.2836	0.0786
T	C	0.6324	0.0324
T	C	-0.4665	-0.0072
A	G	-0.6177	0.1699
A	G	-0.2802	0.0786
T	C	0.6184	0.0324
T	C	-0.4927	-0.0072
A	G	-0.6683	0.1699
T	C	0.6285	0.0324
T	C	-0.4838	-0.0072
C	T	-0.1586	0.0277
C	T	0.1747	0.0266
G	A	-0.8972	0.0967
T	C	-0.4696	0.3016
A	G	-1.065	0.1768
A	G	-0.502	0.0213
G	A	-0.6992	0.0967
A	G	-0.7826	0.1768
G	A	-0.912	0.0967
T	G	-0.5334	0.0218
T	C	0.1669	0.0081
T	C	-0.4513	0.3016
A	G	-1.046	0.1768
G	A	-0.7874	0.0967
A	G	-0.8747	0.1768
A	G	-0.4181	0.0213
G	A	-0.8942	0.0967
A	G	-0.9249	0.1768
A	G	-0.4635	0.0213
A	G	0.2574	0.0347
T	C	0.3476	-0.1035
A	G	-0.5431	0.0347
T	C	0.2556	0.012

A	G	-0.4624	0.0347
A	G	0.2927	0.0347
C	T	0.2869	0.0964
A	G	0.5518	0.0347
T	C	-0.254	0.012
A	G	-0.4468	0.0347
T	C	-0.1739	0.0549
G	A	0.1399	0.0195
T	C	0.3978	0.1338
G	A	0.7354	0.0154
A	G	-0.3265	0.1574
A	G	0.5985	0.0375
G	A	0.1407	0.0195
T	C	0.3865	0.1338
G	A	0.7078	0.0154
A	G	-0.3205	0.1574
A	G	0.5857	0.0375
T	G	0.1437	0.0148
G	A	-0.2363	-0.0366
C	T	0.3718	0.1699
T	C	0.3576	0.1338
G	A	0.675	0.0154
T	C	0.2197	-0.0571
A	G	-0.3774	0.1574
A	G	0.5505	0.0375
T	C	0.3694	0.1338
G	A	0.7037	0.0154
T	C	0.2272	-0.0571
A	G	-0.3832	0.1574
A	G	0.564	0.0375
T	C	0.3109	0.1338
G	A	0.5685	0.0154
G	A	-0.1866	0.1096
A	G	-0.4214	0.1574
G	A	0.1488	0.0558
A	G	0.4014	0.0375
T	C	0.1443	0.0047
T	C	0.316	0.1338
G	A	0.6264	0.0154
T	C	-0.3692	0.1588
A	G	0.5148	0.0375
T	C	0.3253	0.1338
G	A	0.6063	0.0154
A	G	-0.435	0.1574
G	A	0.148	0.0558
A	G	0.4184	0.0375
T	C	0.3487	0.1338
G	A	0.6679	0.0154
A	G	-0.3231	0.1574
A	G	0.5556	0.0375
T	G	0.1472	0.0148
A	G	-0.2988	-0.0168
A	C	-1.048	-0.0063
T	C	0.229	0.017
A	G	-0.3607	0.2142
A	C	-0.3742	0.097
T	C	-0.2598	-0.1838
G	A	0.8089	0.0718

A	G	0.3612	-0.232
T	C	-0.2069	0.0448
A	G	-0.2373	-0.071
A	G	0.2608	-0.0568
A	G	0.482	-0.4022
A	G	-1.625	-0.0066
G	A	-0.1818	-0.0263
A	C	-1.514	-0.1549
C	A	-2.942	0.3771
T	C	0.1999	-0.0597
C	T	0.2816	0.1288
A	G	1.52	-0.0066
A	C	1.439	-0.1549
A	C	-0.2655	-0.1084
C	T	0.2762	-0.0477
A	G	1.19	-0.0066
G	A	-0.2144	0.0081
A	G	-1.776	-0.0066
C	T	-1.417	-0.1383
T	C	-0.2213	0.0005
C	T	-0.2879	-0.0477
A	G	-1.249	-0.0066
C	T	-0.2355	-0.0565
A	G	-1.217	-0.0066
A	G	1.174	-0.0066
A	C	-0.2228	-0.1084
C	T	0.2086	-0.0477
A	G	-1.179	-0.0066
A	C	-1.518	-0.1549
T	C	0.3316	0.1293
A	G	-1.821	-0.0066
A	C	-1.784	-0.1549
T	C	-0.2077	0.0005
C	A	-0.9956	-0.1808
A	C	-0.6555	0.1286
C	T	-0.2903	-0.0477
A	G	0.4611	0.0025
A	G	1.815	-0.0066
C	T	1.427	-0.1383
T	C	0.218	0.0005
C	T	0.2886	-0.0477
A	G	1.394	-0.0066
A	C	1.33	-0.1549
T	C	0.7147	-0.4438
T	C	0.6089	0.3216
T	C	0.4963	0.1638
G	T	-0.3245	-0.051
T	C	0.3184	-0.0026
G	T	0.1762	-0.0656
A	G	1.864	-0.0066
A	C	1.793	-0.1549
T	C	0.209	0.0005
C	A	0.9806	-0.1808
T	C	0.4297	-0.2378
C	T	0.2946	-0.0477
A	G	-0.463	0.0025
A	G	-1.078	-0.0066
T	C	0.5758	0.1293

A	G	-1.125	-0.0066
A	C	-1.228	-0.1549
A	G	-1.201	-0.0066
A	C	-1.455	-0.1549
T	C	0.3436	0.1293
A	G	-1.201	-0.0066
A	G	-0.9923	-0.0807
A	G	1.303	-0.0066
C	T	1.164	-0.1383
G	A	0.1724	-0.0263
A	G	0.1726	-0.0252
T	G	-0.267	0.2385
A	G	-1.253	-0.0066
A	G	-2.393	-0.2321
A	G	1.307	-0.0066
C	T	1.191	-0.1383
G	A	0.1611	-0.0263
C	T	0.1716	-0.0256
T	C	0.2582	-0.1838
C	T	-0.1436	0.0375
G	T	-0.6848	0.0631
A	G	-0.2525	0.059
G	A	-0.1371	0.0133
T	G	0.1457	0.0148
T	C	0.306	-0.1838
A	G	-0.3505	0.0299
T	C	-0.5269	-0.1672
G	A	-0.8542	0.0718
A	G	-0.3755	-0.232
T	C	0.2464	-0.1838
C	T	-0.1723	0.0375
G	A	-0.5652	0.0718
T	C	-0.2709	-0.1838
A	G	0.7543	0.584
G	A	0.7524	0.0718
A	G	0.3586	-0.232
T	C	0.2023	-0.1838
G	T	-0.5889	0.0631
C	T	-0.1835	-0.0555
T	C	0.2631	-0.1838
G	T	-0.6984	0.0631
A	G	-0.3019	-0.232
T	C	-0.3094	-0.1838
A	G	0.7724	0.584
G	A	0.9207	0.0718
A	G	0.425	-0.232
T	C	0.2697	-0.1838
C	T	-0.1534	0.0375
G	A	-0.7124	0.0718
T	C	-0.2046	-0.1838
G	A	0.6258	0.0718
T	C	-0.2673	-0.1838
A	G	0.7582	0.584
G	A	0.8088	0.0718
A	G	0.3667	-0.232
T	C	-0.2833	-0.1838
T	C	0.8211	0.651
C	T	-0.3791	-0.0851

G	A	0.8094	0.0718
A	G	0.401	-0.232
T	C	-0.2722	-0.1838
A	G	-0.2072	0.0718
G	T	0.7582	0.0631
A	G	0.3617	-0.232
T	C	-0.2838	-0.1838
C	T	-0.3975	-0.0851
A	G	0.8012	0.584
G	A	0.8232	0.0718
A	G	0.4169	-0.232
T	C	0.3636	-0.1838
T	C	-1.165	0.0549
G	T	-0.2278	-0.0955
C	T	0.2065	-0.0579
G	A	-0.507	-0.0851
T	G	0.4617	0.0584
T	C	-0.3069	-0.1838
G	A	0.929	0.0718
A	G	0.4245	-0.232
G	A	-0.2319	-0.1246
T	C	0.2393	-0.1838
C	T	-0.1551	0.0375
G	A	-0.5493	0.0718
T	C	-0.2265	-0.1838
C	T	0.1529	0.0375
G	A	0.6546	0.0718
A	G	1.543	-0.2762
G	A	1.453	-0.079
C	T	-0.1584	-0.0549
T	C	-0.4047	0.0044
T	C	-0.167	-0.0066
C	T	-0.1524	-0.0549
T	C	-0.3962	0.0044
C	T	-0.1762	-0.0549
T	C	-0.4244	0.0044
T	C	-0.1594	-0.0066
C	T	-0.1768	-0.0549
T	C	-0.4203	0.0044
T	C	-0.1626	-0.0066
G	A	0.6643	-1.1028
T	C	-0.5992	0.0165
G	A	0.751	-1.1028
T	C	-0.291	-0.0217
G	T	1.217	-0.1511
A	G	0.2325	0.0097
T	C	-0.294	0.0483
G	A	0.8001	-1.1028
T	C	-0.3187	0.0352
A	C	0.8525	-0.149
T	C	-0.4802	0.049
G	T	0.2917	0.0332
G	A	0.8336	-1.1028
G	T	1.075	-0.1511
T	C	0.3225	-0.0393
T	C	-0.3418	0.049
A	G	0.2659	0.0097
T	C	-0.3191	0.0483

	C	T	-3.125	0.825
	G	A	0.1424	0.0081
	A	G	-0.3219	0.0045
	C	T	0.2215	-0.014
	A	G	-0.3032	0.0045
	A	G	-0.2032	0.0045
	T	C	0.1884	-0.0825
	A	G	-0.2046	0.0045
	A	G	-0.1847	0.0045
	A	G	-0.2062	0.0045
	A	G	0.2209	-0.0211
	G	A	0.2029	-0.0379
	A	G	-0.2417	0.0045
	A	G	-0.3264	0.0045
	A	G	-0.2479	0.0045
	A	G	-0.2367	0.0045
	T	C	-0.2989	-0.0922
	A	G	0.8507	-0.0433
	G	A	0.1475	-0.0189
	G	A	0.5116	0.265
	G	A	0.1545	-0.0189
	G	A	0.5474	0.265
	T	C	1.147	0.0906
	T	G	-0.1917	-0.0245
	T	G	-0.1359	-0.004
	G	A	0.2221	-0.0564
	T	G	-0.1491	-0.004
	C	T	-0.1437	0.0452
	G	A	0.1567	-0.0841
	C	T	-0.1428	-0.0328
	G	A	0.2335	-0.0564
	T	C	-0.1468	-0.0176
	G	A	0.1898	-0.0564
	C	A	-0.1352	-0.0227
	G	A	0.2114	-0.0564
	T	C	0.5056	0.0382
	A	G	0.3239	-1.0535
	A	G	0.313	0.6199
	T	C	-0.2043	-0.0261
	T	C	0.3801	-0.1077
	C	T	0.3511	-0.0623
	A	G	-0.2604	0.0505
	A	G	0.3426	0.1372
	A	C	-1.138	0.0895
	A	G	0.5006	-0.0202
	G	A	-0.3645	-0.0199
	A	G	-0.4918	0.0656
	C	T	-1.101	-0.1383
	A	G	-1.737	-0.208
	T	C	0.6146	0.1293
	C	T	-1.073	-0.1383
	A	G	-2.154	-0.208
	T	C	-0.5154	-0.2378
	G	A	0.1644	0.0815
	G	A	-0.1408	0.0451
	G	A	-0.1647	0.0449
	G	T	-0.3173	0.0703
	G	A	0.6413	0.165

T	C	0.3683	0.7883
T	C	-0.2143	0.0296
T	C	0.1525	0.0022
G	A	-0.5	-0.1476
T	C	-0.3847	-0.283
T	C	-0.5628	0.0844
A	G	-0.3298	0.0425
T	C	-0.6019	0.0639
G	A	-0.5014	-0.1476
T	C	-0.3738	-0.283
T	C	-0.3468	0.0938
C	T	-1.492	-0.0567
T	C	-0.574	0.0844
A	G	-0.3402	0.0425
T	C	-0.6147	0.0639
G	A	-0.4893	-0.1476
T	C	-0.3673	-0.283
T	C	-0.3347	0.0938
T	C	-0.5915	0.0844
A	G	-0.3514	0.0425
C	T	-0.1521	-0.0249
T	C	-0.6396	0.0639
A	G	-1.465	-0.0606
G	A	-0.503	-0.1476
A	G	-0.3452	0.0942
A	G	-0.3391	0.0425
C	T	-0.1739	-0.0249
T	C	-0.6019	0.0639
A	G	-0.5742	0.0456
G	A	-0.5069	-0.1476
T	C	-0.3667	-0.283
T	C	-0.5694	0.0844
A	G	-0.3357	0.0425
T	C	-0.6016	0.0639
G	A	-0.4887	-0.1476
A	G	-0.3333	0.0942
A	G	-0.3562	0.0425
T	C	-0.6648	0.0639
A	G	-0.6176	0.0456
G	A	0.4819	0.0154
G	A	-0.1604	-0.1005
C	T	-0.2225	0.0653
G	A	0.5503	0.1153
G	A	0.4371	-0.025
C	T	0.1864	-0.0028
A	G	0.6636	0.0212
C	T	-0.8348	-0.0883
C	T	-1.009	-0.1135
A	G	0.1995	-0.5309
C	T	-0.2424	0.0549
T	C	0.2328	0.0366
T	G	-0.255	0.1388
T	C	-0.4077	0.1408
C	T	-0.3545	0.1547
A	G	0.4455	0.0314
C	T	0.1619	0.0535
A	G	-0.1526	0.0223
T	C	0.2094	0.017

	A	G	-0.731	0.1523
	T	C	-0.6022	-0.0099
	T	C	-0.322	-0.0413
	C	A	-0.3511	0.0328
	A	G	-1.005	0.072
	T	C	-1.396	-0.0606
	T	C	-0.1856	-0.053
	T	G	0.3259	0.0772
	G	A	0.4285	-0.059
	T	C	0.4289	0.03
	A	G	-1.619	-0.208
	T	C	1.154	0.2063
	C	T	3.098	0.1711
	A	G	1.755	-0.208
	T	C	-1.139	0.2063
	T	C	-0.1685	0.0677
	G	A	0.1259	0.0543
	A	G	-2.093	0.0128
	G	A	-0.1456	-0.0564
	T	C	-0.4163	0.2087
	G	A	0.1822	0.1096
	T	G	-0.1909	0.0148
	A	G	2.135	0.0128
	G	A	0.1363	-0.0841
	T	G	0.1693	0.0148
	C	T	0.4573	0.3224
	A	C	-0.3123	-0.0063
	T	C	-0.6054	0.0165
	A	T	-0.5252	-0.0422
	G	A	-0.5252	-0.0124
	T	C	-0.2672	0.042
	C	T	0.4454	0.3224
	A	C	-0.315	-0.0063
	T	C	-0.5871	0.0165
	A	T	-0.5084	-0.0422
	G	A	-0.5084	-0.0124
	T	C	-0.2607	0.042
	C	T	-0.4695	0.3224
	G	T	-0.4306	0.0739
	T	C	-0.2405	0.0355
	T	C	0.4161	0.0165
	A	T	0.3668	-0.0422
	G	A	0.3668	-0.0124
	A	C	0.3789	-0.3513
	T	C	0.8124	-0.149
	T	C	-0.1722	-0.1032
	G	A	0.1927	0.0313
	G	A	0.3455	0.0576
	A	G	-0.2773	0.0786
	T	C	0.644	0.0324
	G	T	0.1732	-0.0381
	A	G	-0.2709	0.0786
	A	G	0.6021	0.0203
	C	T	0.447	0.0434
	A	G	-0.1899	-0.0131
	G	A	0.209	0.0493
	T	C	0.1883	-0.095
	C	T	0.4334	0.0434

G	A	-0.7279	0.2503
A	G	0.3619	-0.0121
G	A	0.1538	-0.0611
C	A	-0.2306	-0.1116
C	T	-1.355	0.179
T	C	-0.4459	0.3137
G	A	-0.2158	-0.0568
C	T	0.2065	-0.0375
G	T	0.2233	0.0348
C	T	-0.1786	0.0525
G	A	-0.2011	0.0608
T	C	0.1521	-0.0714
C	T	-0.1793	0.0187
G	T	0.2121	0.0348
G	A	-0.1808	0.062
T	C	0.183	-0.1019
T	G	0.1996	-0.0532
T	C	-0.1657	0.0705
A	G	0.4479	-0.0845
C	T	-0.293	0.0576
T	C	-0.1378	-0.0505
C	T	0.2704	0.0152
A	C	-0.9497	0.0895
A	G	-0.6061	-0.1488
G	A	-0.3103	-0.0199
T	C	0.2681	-0.0242
G	A	-0.1558	-0.1103
A	G	0.187	0.0744
T	C	-0.2571	0.1705
C	T	-0.1948	0.0525
G	A	0.2057	0.0514
G	A	0.3893	0.0192
A	G	-0.378	0.0608
A	C	0.4977	0.2211
G	A	-0.4762	0.0718
T	C	-0.1801	0.0455
A	G	0.2608	0.0603
C	A	-0.2999	0.1087
A	G	0.1861	0.1216
G	T	-0.1713	0.035
G	A	0.3015	-0.0014
A	G	-0.6824	-0.076
C	T	-0.2078	-0.0133
G	A	-0.2231	-0.0013
A	G	-0.5258	-0.0769
T	C	0.2079	0.017
G	A	0.183	-0.0263
T	C	-0.1866	0.0604
G	A	-0.1632	-0.0263
G	A	0.1603	-0.0263
T	C	0.3212	-0.196
C	T	0.4215	-0.8331
G	A	0.4156	-0.0123
G	A	0.1537	-0.1124
G	A	0.2272	-0.0564
G	A	0.1827	-0.0564
A	G	-0.1525	-0.0026
T	C	0.5455	0.0382

	G	A	0.1963	-0.0564
	G	A	0.173	-0.0564
	G	A	0.1523	-0.0564
	T	C	0.4968	0.0382
	G	A	0.1714	-0.0564
	G	A	-0.2201	-0.0564
	C	T	0.1572	-0.0137
	G	A	0.1691	-0.0564
	C	T	-0.2044	0.0899
	T	C	0.6441	0.0324
	C	T	0.5386	0.0434
	A	G	0.2304	-0.098
	G	A	-0.1568	-0.1005
	A	G	0.1662	-0.0407
	A	G	0.2531	-0.1375
	C	T	0.2002	0.056
	C	T	0.1837	0.0518
	A	G	1.79	-0.2321
	G	A	0.246	-0.0013
	T	G	-0.3142	0.2385
	T	C	-0.3797	-0.283
	A	G	-0.3313	0.0942
	T	C	-0.5638	0.0844
	A	G	-0.725	0.1445
	C	T	-0.1503	-0.0249
	T	C	-0.5779	0.0639
	T	C	-1.439	-0.0615
	T	C	-0.355	-0.283
	A	G	-0.3245	0.0942
	T	C	-0.5779	0.0844
	A	G	-0.7252	0.1445
	C	T	-0.1479	-0.0249
	T	C	-0.6004	0.0639
	T	C	-1.427	-0.0615
	G	A	-0.2835	0.1038
	T	C	-0.182	0.0443
	A	G	0.1855	0.0136
	T	C	-0.3968	-0.1731
	G	A	-0.1511	-0.0699
	A	G	-0.2926	-0.0987
	G	A	-0.4805	0.1154
	T	C	0.1962	0.1246
	A	G	0.1517	-0.0982
	A	G	-0.1487	-0.0505
	T	C	-0.1822	0.017
	A	C	-1.347	-0.1549
	A	G	-1.532	-0.208
	A	G	0.4628	0.0025
	A	C	0.3794	0.0624
	T	C	-0.201	-0.0339
	A	C	0.4108	-0.0879
	T	C	-0.5907	0.0067
	A	G	-0.2671	-0.0163
	G	A	0.2205	-0.1236
	T	C	-0.659	0.472
	A	G	-0.8103	-0.2813
	A	G	-0.9089	0.1078
	T	G	-1.096	-0.0129

T	C	-0.7524	0.0606
T	C	0.3156	0.017
T	C	-0.6814	-0.1064
G	A	0.4291	-0.1522
G	T	0.2159	-0.0657
G	A	0.1701	-0.012
G	A	0.415	-0.1522
T	C	0.2327	-0.0571
A	G	0.3543	0.0375
G	A	0.1408	-0.012
G	A	0.5664	-0.1522
T	C	0.4888	0.03
G	A	0.4569	-0.1522
T	C	0.246	-0.0571
A	G	0.3685	0.0375
G	A	0.4806	-0.1522
A	G	0.425	0.0375
G	A	0.5228	-0.1522
T	C	0.471	0.03
G	A	-0.4403	-0.1522
T	C	-0.4347	0.1658
T	C	-0.4661	0.03
G	A	0.4624	-0.1522
A	G	0.4706	0.0575
G	A	0.1387	0.0558
T	C	-0.4086	0.0388
T	C	-0.3567	0.3647
A	G	-0.2576	-0.0421
G	A	-0.4589	0.0707
T	C	-0.5625	0.0844
A	G	-0.6961	0.1445
T	C	-0.6208	0.0639
T	C	-1.495	-0.0615
A	G	0.5509	0.4085
A	G	0.2745	0.1098
A	G	-0.2587	0.0155
T	C	0.2107	-0.0243
C	A	-0.5725	-0.1699
C	T	0.1995	0.0459
C	T	0.4055	-0.0116
G	A	0.3323	0.0718
C	A	-0.5867	-0.1699
C	T	0.2265	0.0459
G	A	0.4659	-0.0153
C	A	-1.147	-0.1699
C	T	-0.5868	0.2918
C	T	-0.1646	0.0644
C	T	0.6619	-0.0192
G	T	-0.35	-0.1
T	C	-0.7914	0.0577
C	T	-0.3597	0.3947
T	C	0.2696	-0.0031
T	C	-0.1537	0.042
A	G	-0.7492	-0.0607
A	G	0.2454	-0.0655
C	A	-0.4165	0.0475
G	A	0.4579	-1.0602
A	G	-0.2784	0.0016

T	C	-0.4302	0.0165
T	C	-0.3341	-0.3335
T	G	-0.1851	-0.0391
T	C	0.1642	0.0022
G	T	-0.4036	0.2068
T	C	-0.3287	-0.3335
T	C	0.2439	-0.0654
G	A	-0.4405	-0.0131
T	C	0.2427	-0.0654
G	A	-0.4395	-0.0131
C	T	0.1655	-0.0555
A	C	0.3084	-0.0063
T	C	-0.1786	0.017
T	C	-0.2623	0.1606
A	C	0.4143	-0.0055
A	G	0.3664	-0.0023
T	G	1.574	-0.2792
G	A	0.1972	-0.0013
T	C	-0.3331	0.1248
T	C	0.2272	0.0447
A	C	0.3795	-0.0083
C	T	0.476	0.1699
A	C	0.4465	-0.0083
C	T	0.2314	0.1048
T	C	-0.1953	-0.0066
C	T	0.5626	0.1699
A	C	0.4264	-0.0083
C	T	0.2334	0.1048
T	C	-0.1898	-0.0066
A	C	0.5847	0.1705
T	C	-0.183	0.0067
A	C	0.4239	-0.0083
T	C	-0.1654	-0.0066
C	T	0.4838	0.1699
A	C	0.4191	-0.0083
A	G	-0.9269	-0.2712
A	C	0.5433	0.1705
C	T	0.2402	0.0491
A	G	-0.3396	0.1572
A	G	0.3722	0.0314
C	T	-0.3114	0.0556
C	T	0.1467	-0.0173
T	C	-0.5288	-0.0615
C	T	0.2881	0.0964
T	C	0.3331	-0.1511
G	T	-0.1381	-0.0055
G	A	0.2082	0.0253
C	T	-0.2125	0.0549
G	A	0.2257	0.0253
T	C	-0.2717	0.1705
C	T	-0.2717	0.0525
G	A	0.2896	0.1609
G	A	0.2422	-0.0144
A	C	0.2173	-0.0755
G	T	0.2054	-0.0134
T	C	-0.2745	0.0453
C	T	0.683	-0.0058
A	G	0.2565	-0.0667

T	C	0.5993	-0.0298
T	C	-0.209	0.0453
C	T	0.3833	-0.0058
T	C	-0.1614	0.0443
C	T	0.428	0.0215
C	T	0.3622	0.0434
C	T	-0.2533	-0.0555
T	C	-0.2007	-0.0692
A	C	-0.2687	0.0895
C	T	-0.5186	-0.2891
C	T	0.3643	0.0805
G	A	-0.2979	-0.1105
A	G	0.1789	-0.0241
C	T	0.3265	0.0182
G	A	0.3496	0.0192
G	A	0.4384	0.0192
G	A	0.4235	0.0192
A	G	0.254	-0.0121
G	A	0.2737	0.0192
G	A	0.4112	0.0192
G	A	0.3668	0.0192
C	T	0.2839	0.0658
T	G	-0.2523	-0.0391
A	G	-1.51	-0.208
T	C	0.1803	0.1243
A	G	1.651	-0.208
A	C	-0.2089	-0.1084
C	T	0.2177	-0.0477
A	G	-1.502	-0.208
T	G	0.1499	0.0543
G	A	0.1592	-0.0013
T	C	-0.1619	-0.0943
T	C	0.5547	0.1293
G	A	0.2111	-0.0212
T	C	-0.6608	-0.0755
G	A	0.2289	0.1476
A	G	0.3371	0.0086
C	T	0.2805	0.018
G	A	-1.353	0.5984
G	A	-0.2018	0.1258
G	A	-0.4386	0.314
A	C	-0.2797	-0.0063
A	C	-0.2562	-0.0063
T	C	-0.2546	0.1606
T	C	-0.5388	0.0165
A	T	-0.4741	-0.0422
G	A	-0.4741	-0.0124
T	C	-0.2618	0.042
A	C	-0.2492	-0.0063
A	G	-0.1859	-0.0237
T	G	0.4573	-0.1613
A	C	-0.2824	-0.0063
T	G	0.1733	0.0997
G	A	0.5511	-0.025
A	G	-0.4313	0.0437
T	C	0.361	0.0549
G	A	0.4646	-0.025
A	G	0.3595	0.0601

G	T	0.1985	0.0272
A	G	0.6978	0.0212
G	A	0.248	-0.025
C	T	0.4684	0.0737
G	A	-0.3379	-0.025
G	A	0.461	-0.025
G	A	-0.2575	-0.025
A	C	-1.139	0.0895
C	T	-0.2061	0.084
A	G	-0.675	-0.1488
T	C	0.4941	-0.2186
G	A	-0.3895	-0.0199
T	C	0.2922	-0.0242
G	A	-0.41	-0.025
A	G	0.2391	0.0291
G	A	-0.5517	-0.025
A	G	0.4291	0.0437
T	C	-0.3541	0.0549
G	A	0.2617	-0.025
G	A	0.624	-0.025
G	A	-0.2388	-0.025
G	A	-0.3112	-0.025
G	A	0.4941	-0.025
G	A	0.1446	-0.025
G	A	-0.261	-0.025
A	C	-1.119	0.0895
A	G	-0.6956	-0.1488
G	A	-0.6094	0.232
G	A	-0.3832	-0.0199
T	C	0.2977	-0.0242
G	A	-0.4504	-0.025
A	G	0.2834	0.0291
G	A	-0.4628	-0.025
A	C	0.1581	0.029
G	A	0.2755	-0.025
G	A	0.2581	-0.025
A	G	0.3253	-0.0655
T	C	0.6113	0.1293
T	C	-0.3926	0.2087
A	G	0.2022	0.1089
T	G	-0.2005	0.0148
T	C	0.5102	0.2087
C	T	0.2237	-0.0302
A	C	-0.7905	0.093
A	G	-0.7731	0.1768
T	G	0.3174	-0.1745
C	T	-0.1869	-0.0003
C	T	-0.1907	0.0525
A	G	0.1924	0.0678
T	C	0.2363	0.0366
C	T	-0.4972	-0.0003
A	G	-0.1925	0.0648
C	T	0.7463	0.2704
A	G	0.4946	-0.3229
A	G	0.2435	-0.1401
C	A	0.5735	0.0611
C	T	-0.1431	-0.0003
G	A	-0.1679	-0.0475

C	T	0.2306	-0.0003
A	G	0.3186	0.1607
C	T	0.2928	0.0005
C	T	0.3004	0.0005
C	T	0.3092	0.0005
C	T	0.2857	0.0005
C	T	0.2857	0.0005
C	T	0.2864	0.0005
C	A	0.6261	-0.142
T	C	1.583	-0.2571
T	C	-0.383	0.1546
G	A	-0.2628	0.1889
C	T	-0.2794	-0.0422
G	A	-0.2664	0.1889
C	T	-0.3029	-0.0422
G	A	-0.1965	0.062
C	T	-0.1667	0.0525
C	A	-0.1343	0.0123
T	C	0.1572	-0.0714
C	T	-0.1712	0.0187
G	T	0.3661	-0.0705
A	G	0.233	0.0145
G	T	0.3858	-0.0705
A	G	0.2512	0.0145
C	T	37.99	-0.1261
G	A	-2.745	0.0081
C	A	0.2334	-0.0803
T	C	0.6864	0.0324
C	T	0.5489	0.0434
T	C	-0.2711	0.0679
A	G	0.3485	-0.0554
T	C	-0.1665	-0.1207
T	C	-0.7004	0.0067
G	A	-0.2692	0.0267
T	C	-0.6783	0.0067
A	G	0.2255	0.0744
G	A	-0.2046	0.0608
T	C	0.1645	-0.0714
C	T	-0.1682	0.0187
A	G	0.3353	0.0744
A	G	0.1684	-0.0963
T	C	0.1494	-0.0714
T	C	-0.1706	-0.0452
T	C	-0.1785	0.0624
T	G	0.173	0.0268
G	A	-0.182	-0.0882
A	G	-0.1919	-0.0946
C	T	0.1937	0.0495
C	T	0.2363	-0.0252
G	A	-0.4223	0.0574
T	C	-0.4101	0.049
C	T	-0.1446	-0.0706
A	G	-0.4286	-0.0987
G	A	-0.2122	-0.0751
C	T	-0.1628	-0.0706
A	G	-0.4355	-0.0987
G	A	-0.2015	-0.0751
A	G	0.2882	-0.0444

	C	T	0.2584	-0.0308
	C	T	0.2382	-0.0308
	T	C	0.2484	-0.0378
	T	C	0.2305	-0.0378
	A	G	-0.276	0.1174
	A	G	0.1758	-0.016
	G	A	-0.7439	0.3055
	A	G	0.2114	-0.016
	G	A	-0.8239	0.1121
	A	G	0.1799	-0.016
	G	A	-0.7352	0.3055
	A	G	0.1798	-0.016
	G	A	-0.5307	0.1121
	A	G	0.1623	-0.016
	G	A	-0.6739	0.1121
	A	G	0.1396	-0.016
	G	A	-0.432	0.1121
	A	G	0.1789	-0.016
	G	A	0.1513	-0.0448
	G	A	-0.858	0.3055
	A	G	0.1709	-0.016
	G	A	-0.7073	0.3055
	A	G	0.1681	-0.016
	G	A	-0.7377	0.1121
	A	C	0.1811	0.0208
	T	C	0.4371	0.017
	G	A	0.1359	-0.0475
	C	T	0.2841	0.003
	G	A	0.1466	-0.0475
	C	T	-0.3526	0.2704
	G	T	0.3898	-0.0022
	T	G	-0.2187	-0.0061
	C	T	0.4909	0.1699
	T	C	-0.2484	0.0067
	A	G	-0.165	0.1328
	T	C	-0.2936	0.0333
	C	T	0.1751	-0.0784
	G	A	0.3938	-0.0422
	T	C	-0.2816	-0.0276
	C	T	-0.145	0.0453
	G	T	-0.5526	0.0631
	T	C	-0.1602	-0.1021
	G	A	-0.1537	-0.0486
	C	T	-0.1522	0.0198
	T	C	0.1657	0.0152
	G	A	-0.3008	-0.1105
	G	A	0.1639	0.0614
	C	T	0.3571	0.0805
	A	G	-0.2854	0.0648
	C	T	0.9344	0.2704
	G	T	-0.5719	-0.0022
	A	G	0.5453	-0.0516
	A	G	0.5783	-0.3229
	T	G	0.1645	-0.0003
	A	G	0.1769	0.0134
	A	G	-0.1407	0.0442
	C	A	-0.3193	0.0152
	T	C	-0.2761	0.049

T	G	0.3426	0.1115
G	T	0.1877	0.0905
G	A	-0.4218	-0.1105
G	A	0.2919	0.0122
G	A	0.299	0.0122
A	G	-0.2027	-0.1208
A	C	0.4338	-0.0879
T	G	-0.1633	-0.0345
T	C	-0.6633	0.0067
G	A	0.1554	0.0523
T	C	-0.1749	-0.1207
T	C	-0.5948	0.0067
T	C	-0.2111	-0.1207
A	C	0.4141	-0.0879
T	G	-0.1647	-0.0345
T	C	-0.6238	0.0067
G	A	0.1551	0.0523
T	C	0.521	0.0324
C	T	0.4147	0.0434
T	C	0.567	0.0324
C	T	0.4895	0.0434
A	G	0.2019	-0.076
T	C	0.5477	0.0324
T	C	0.5264	-0.0943
T	C	0.2073	0.0326
T	C	0.4749	0.0204
C	T	0.4331	0.0434
A	G	0.2396	0.0086
A	G	0.4829	0.0203
G	A	-0.5196	-0.1105
A	G	-0.3114	-0.0987
T	C	-0.1334	-0.0706
A	G	-0.4526	-0.0987
T	C	-0.1295	0.0639
G	A	-0.1852	-0.0751
A	G	-0.4689	-0.0987
A	G	-0.2598	0.1255
T	C	0.4397	-0.169
T	C	-0.1552	-0.0706
A	G	-0.4525	-0.0987
G	A	-0.1929	-0.0751
C	T	-0.1999	0.0266
T	G	0.1976	-0.1207
T	C	-1.668	0.0193
T	G	0.303	-0.1207
A	G	-0.2185	-0.071
C	T	0.2514	-0.0568
T	C	0.1887	0.1243
T	C	0.1801	0.1243
T	C	0.1894	0.1243
A	G	-0.22	0.0379
C	T	-0.2103	0.0653
T	C	0.1905	0.1243
C	T	-0.1609	-0.0885
C	T	-0.1468	-0.0885
A	G	0.2154	-0.0568
C	T	-0.1838	-0.0885
A	G	0.1995	0.2122

T	C	0.1526	0.0953
T	C	-0.4161	0.1408
A	G	0.1659	-0.0575
G	T	-0.172	0.035
G	A	0.2827	-0.0014
G	A	0.2434	-0.0014
A	C	0.196	-0.0534
A	C	0.1709	0.0535
C	T	-0.3044	0.2704
G	T	0.317	-0.0022
G	A	-0.4732	0.0574
T	G	0.3125	0.0112
G	T	0.2327	0.0272
T	C	-0.1749	-0.0066
A	G	0.4244	0.1607
T	C	-0.1698	-0.0066
C	T	0.5734	0.1699
T	G	0.2184	-0.0229
T	C	-0.1813	-0.0066
A	C	0.349	0.1705
T	C	-0.2486	0.0067
G	A	-0.6399	0.0718
A	G	-0.3061	-0.232
G	A	-0.7013	0.0718
A	G	-0.2863	-0.232
T	C	-0.2739	0.0549
A	C	-1.214	0.0895
A	G	-0.7134	-0.1488
G	A	-0.326	-0.0199
T	C	0.2876	-0.0242
A	C	-0.3278	0.0895
T	C	-0.2252	0.0067
A	C	-1.223	0.0895
A	G	-0.7209	-0.1488
G	A	-0.3413	-0.0199
T	C	0.2935	-0.0242
A	C	-0.3696	0.0895
A	C	-0.5183	0.0895
T	C	0.2541	-0.0276
G	A	-0.2756	-0.0199
A	C	-1.151	0.0895
T	C	0.2625	-0.0197
G	A	-0.3677	-0.0199
A	C	-0.3767	0.0895
A	C	-1.219	0.0895
A	G	-0.7095	-0.1488
G	A	-0.3366	-0.0199
T	C	0.2938	-0.0242
A	C	-1.143	0.0895
C	T	-0.2031	0.084
A	G	-0.6341	-0.1488
T	C	0.4387	-0.2186
G	A	-0.4007	-0.0199
T	C	0.2828	-0.0242
G	T	0.1797	0.0324
T	C	0.1721	0.0815
T	G	0.4952	0.2651
C	T	0.167	-0.0325

C	T	0.5555	0.1699
T	G	0.2383	-0.0229
T	C	0.2826	0.017
T	C	0.4475	0.017
A	G	0.1831	0.0186
T	C	0.1669	-0.101
A	G	0.3611	0.064
G	A	0.3359	0.0303
T	C	0.2947	0.0659
G	A	0.3527	-0.0306
T	C	-0.4865	-0.0869
G	A	0.4892	0.0383
C	T	-0.1484	-0.0249
A	G	0.2224	-0.0655
T	G	-0.4084	0.0473
T	C	-0.1403	0.0055
C	T	-0.2435	0.0653
C	T	0.2194	0.0653
C	T	-0.2034	0.0653
G	A	-0.1458	-0.0768
A	G	-0.2169	0.0862
C	T	0.2435	0.0653
C	T	-0.2283	0.0653
G	A	0.1004	0.0304
T	G	-0.4474	0.0151
A	G	-0.1994	-0.0381
T	C	0.5889	0.049
C	T	0.3483	0.2031
T	C	-0.1809	0.049
G	A	-0.4779	0.1518
A	G	0.23	-0.1375
G	A	-0.2188	-0.0218
C	T	0.1763	0.056
A	G	1.653	-0.4407
T	C	-0.2591	0.0298
G	A	0.3276	0.0701
T	C	0.1669	0.0188
A	G	-0.442	-0.4301
A	C	0.3947	0.0373
T	C	-0.279	0.0559
G	A	-0.1543	-0.088
A	C	0.1945	-0.0568
G	A	-0.2671	0.0006
T	C	0.2582	-0.0169
T	C	0.386	0.1293
T	C	0.4625	0.1293
T	C	0.3212	0.1293
A	G	-0.7401	-0.0607
T	C	0.4562	0.1293
C	T	-0.2077	-0.0076
T	C	0.5156	0.1293
T	C	0.592	0.1293
T	C	-0.3589	0.1408
C	T	-0.5184	0.139
A	G	0.5273	0.0145
C	T	-0.4171	0.139
A	G	0.3653	0.0145
T	C	-0.5164	0.1135

T	C	-0.2295	0.1606
G	A	-0.3033	0.1049
A	T	-0.2475	-0.0422
G	A	-0.2475	-0.0124
T	C	-0.1923	0.0483
A	G	-0.2184	-0.0375
A	G	-0.3012	0.0134
A	G	-0.2089	-0.0375
A	G	-0.2821	0.0134
A	G	-0.2093	-0.0375
A	G	-0.2855	0.0134
A	G	-0.2192	-0.0375
A	G	-0.3048	0.0134
A	C	0.2969	0.1705
T	C	-0.2255	0.0067
T	C	0.3322	0.0209
T	G	0.1714	-0.0229
T	G	0.182	-0.0229
A	G	-0.186	-0.0023
G	A	0.1912	-0.0013
A	G	0.1921	-0.0548
G	A	0.1857	-0.012
C	T	-0.1726	-0.0477
C	T	0.1507	-0.0477
G	T	0.2134	-0.0657
G	A	0.1433	-0.012
A	G	-0.3414	0.0593
T	C	-0.1582	-0.0261
T	C	-0.5029	0.0067
G	A	0.5196	0.0718
C	T	0.1434	-0.0325
A	G	-0.3128	0.0745
A	G	-0.8358	0.0745
G	A	-0.2821	-0.0199
T	C	0.2277	-0.0242
G	T	0.3572	-0.0214
G	T	-0.3399	-0.0214
G	A	0.3805	-0.1341
A	G	-0.7447	-0.0175
C	T	0.2953	0.0038
T	C	-0.5191	-0.0615
T	C	0.5551	0.0382
C	T	-0.1934	-0.0129
C	T	0.1905	-0.0129
A	G	-0.9757	0.2928
A	G	0.4393	-0.0786
A	G	0.292	0.0035
T	C	0.483	-0.0869
C	T	0.4516	0.0869
C	T	0.4384	0.0869
A	G	-0.6069	0.1768
T	C	-0.406	0.1586
A	G	-0.1502	0.0588
A	G	0.1512	0.0588
C	T	0.2186	0.019
T	C	0.1479	0.0322
C	T	0.1834	-0.0329
T	C	-0.3961	0.0388

T	C	-0.3964	0.0388
C	T	0.1671	-0.0623
G	T	0.287	0.0584
T	C	-0.5658	0.0165
A	T	-0.3876	-0.0422
G	A	-0.3876	-0.0124
C	T	-0.1677	-0.0008
C	T	0.1597	-0.0623
T	C	-0.2715	0.0165
A	T	-0.2168	-0.0422
G	A	-0.2168	-0.0124
C	T	0.2356	-0.0115
T	G	-0.1904	0.0148

EAF.exposure	EAF.outcome	SE.exposure	SE.outcome	Pval.expos	Pval.outcome
0.1224	0.0817	0.04052	0.0842	3.61E-24	0.4115
0.1224	0.0817	0.04003	0.0842	5.08E-22	0.4115
0.5475	0.2659	0.02717	0.0517	3.03E-08	0.1758
0.5254	0.3535	0.02681	0.0478	2.35E-09	0.9214
0.6024	0.7119	0.02509	0.0501	3.34E-08	0.1865
0.821	0.8344	0.03213	0.0618	1.75E-13	0.2283
0.3094	0.1514	0.02642	0.0637	2.90E-10	0.1306
0.6088	0.5047	0.02474	0.0456	2.50E-21	0.1821
0.3161	0.2954	0.0268	0.0497	7.20E-11	0.151
0.8208	0.7863	0.03256	0.0557	4.23E-08	0.0603198
0.5889	0.5173	0.02955	0.0518	2.13E-13	0.7185
0.0012	0.03528	49.2	0.1225	3.58E-13	0.5634
0.0019	0.04052	39.64	0.1144	1.85E-12	0.6254
0.0103	0.08082	17.59	0.0858	1.86E-11	0.1375
0.0004	0.05048	78.14	0.1049	1.12E-35	0.5733
0.0019	0.07432	40.68	0.0854	2.65E-23	0.865
0.0031	0.03129	30.85	0.135	2.49E-12	0.676799
0.0173	0.01398	13.66	0.1974	3.28E-12	0.4139
0.0173	0.002535	13.96	0.4796	4.67E-09	0.8303
0.0015	0.01217	43.46	0.2174	3.75E-12	0.606001
0.0015	0.03513	43.15	0.1295	1.04E-12	0.8397
0.0035	0.009531	29.94	0.2376	6.06E-11	0.9887
0.0026	0.06333	34.55	0.0934	2.34E-09	0.746799
0.0021	0.006678	38.71	0.2919	9.39E-10	0.3986
0.001	0.05642	65.92	0.0984	4.58E-09	0.4022
0.0019	0.03932	42.57	0.1159	5.14E-09	0.8485
0.0019	0.02419	40.49	0.148	6.43E-13	0.2238
0.0114	0.06263	16.51	0.0957	7.06E-09	0.491801
0.0015	0.02801	42.75	0.1433	8.74E-16	0.902
0.0004	0.006228	81.57	0.2922	8.89E-35	0.6902
0.0026	0.01249	32.42	0.2059	2.03E-10	0.2169
0.0015	0.012	46.04	0.2085	1.30E-11	0.768199
0.0012	0.0298	51.06	0.1324	4.77E-14	0.3055
0.0021	0.01304	37.5	0.2025	1.11E-08	0.9564
0.0037	0.0181	29.08	0.1684	7.67E-11	0.0303997
0.0015	0.00489	43.36	0.3375	9.91E-15	0.742299
0.0023	0.01788	37.63	0.1745	1.01E-17	0.4125
0.0038	0.03087	28.61	0.1309	1.93E-09	0.4759
0.0022	0.02511	39.65	0.1479	3.61E-09	0.8125
0.0006	0.03563	66.51	0.1224	2.81E-27	0.2051
0.0004	0.01606	82.11	0.181	9.85E-16	0.2233
0.0059	0.009233	25.24	0.247	3.67E-08	0.3096
0.0019	0.01699	39.88	0.1783	2.24E-09	0.7925
0.0054	0.003958	23.53	0.3657	1.21E-11	0.0914092
0.0003	0.002179	88.02	0.5178	1.69E-12	0.0359998
0.0019	0.004999	48.3	0.3332	1.66E-12	0.8957
0.0018	0.007492	40.08	0.2743	8.40E-12	0.5619
0.0041	0.02102	28.52	0.1581	1.88E-11	0.9479
0.0023	0.02012	34.4	0.1717	1.36E-10	0.4496
0.0034	0.03617	30.9	0.1218	1.01E-08	0.3669
0.0023	0.006918	36.33	0.2807	1.15E-08	0.0645194
0.0016	0.02378	41.99	0.1597	7.06E-14	0.3651
0.0003	0.02656	91.49	0.1421	4.87E-17	0.4476
0.0006	0.04747	70	0.1063	2.61E-29	0.1269
0.0006	0.01865	70.06	0.1661	6.21E-27	0.9295
0.0009	0.01369	61.21	0.2122	1.66E-20	0.9524

0.0003	0.01552	97.49	0.1835	6.49E-20	0.3958
0.0006	0.02165	73.93	0.162	1.04E-32	0.3786
0.0043	0.01139	28.32	0.2101	2.05E-08	0.0360903
0.0015	0.02905	44.19	0.1373	1.40E-09	0.8021
0.0022	0.03139	38.79	0.1329	2.47E-14	0.628801
0.0006	0.01123	78.94	0.2092	2.29E-36	0.4984
0.9974	0.995	40.25	0.3106	7.08E-09	0.4373
0.0012	0.005583	62.49	0.3243	2.10E-11	0.4392
0.0006	0.0029	80.31	0.4615	6.49E-25	0.6389
0.0065	0.01003	21.64	0.2373	5.99E-09	0.8703
0.001	0.02137	52.88	0.1586	1.14E-18	0.5892
0.0035	0.01892	29.94	0.1659	9.89E-11	0.236
0.006	0.001528	23.63	0.5752	6.03E-12	0.9558
0.0032	0.03212	30.92	0.1283	5.91E-09	0.1909
0.0028	0.01135	33.32	0.2077	2.52E-14	0.1618
0.0028	0.003273	32.03	0.4063	8.96E-11	0.395
0.001	0.09368	54.04	0.0779	1.71E-16	0.1919
0.0013	0.06351	57.27	0.0924	4.68E-11	0.2922
0.0032	0.0124	31.2	0.2048	2.73E-09	0.3271
0.0006	0.02242	69.06	0.1608	2.64E-10	0.4636
0.0019	0.01421	39.21	0.208	1.68E-11	0.7003
0.0009	0.005856	62.7	0.3078	2.43E-09	0.615
0.0022	0.004574	35.66	0.3608	4.08E-08	0.2618
0.0035	0.0115	27.65	0.2203	1.31E-22	0.537
0.001	0.01011	53.64	0.236	1.41E-14	0.907
0.0015	0.00616	45.06	0.2934	3.01E-08	0.1402
0.0021	0.01756	39.24	0.1732	4.46E-08	0.391
0.0006	0.02242	74.11	0.1583	3.13E-30	0.2043
0.001	0.02472	57.77	0.148	7.11E-23	0.02913
0.0034	0.02701	31.22	0.1429	7.31E-13	0.0937389
0.0026	0.002771	37.47	0.4348	3.86E-08	0.4403
0.0079	0.004577	20.2	0.3641	3.50E-12	0.7787
0.0021	0.0729	37.81	0.0862	7.63E-11	0.4168
0.0032	0.01371	31.46	0.1958	4.77E-08	0.763999
0.0006	0.01329	70.89	0.2058	4.89E-28	0.604999
0.0079	0.02959	19.94	0.137	2.77E-08	0.974
0.004	0.004823	28.49	0.3662	4.08E-18	0.3273
0.0022	0.01396	37.09	0.1931	2.41E-11	0.1126
0.0018	0.025	52.39	0.1467	1.25E-22	0.6163
0.001	0.01554	55.24	0.1873	6.76E-22	0.601999
0.0251	0.1349	11.29	0.0676	1.55E-11	0.2449
0.0007	0.003013	69.55	0.416	1.90E-23	0.1934
0.0057	0.002896	23.32	0.4256	4.38E-13	0.616601
0.0025	0.02593	36.99	0.1462	3.79E-11	0.8196
0.0006	0.03653	72.17	0.1186	2.50E-53	0.1471
0.0013	0.05676	43.09	0.0961	1.11E-45	0.5947
0.0012	0.04888	60.13	0.1053	4.27E-08	0.6418
0.0035	0.01342	29.17	0.2041	1.22E-08	0.9337
0.0113	0.02516	17.57	0.1471	2.08E-08	0.5768
0.0185	0.001344	13.57	0.7588	5.08E-10	0.08362
0.0012	0.0332	51.4	0.1289	6.59E-16	0.646001
0.0006	0.01141	72.99	0.2184	1.18E-41	0.3347
0.0009	0.02616	55.97	0.1425	5.40E-20	0.5686
0.0028	0.02674	33.8	0.1423	8.78E-11	0.2625
0.0021	0.0004755	36.89	0.9931	4.30E-11	0.8457
0.0016	0.01627	43.79	0.1856	2.38E-09	0.0390598
0.0029	0.0009055	31.68	0.7721	3.98E-09	0.1547
0.0003	0.03052	88.96	0.1353	5.98E-46	0.5121

0.0054	0.02523	25.13	0.1452	3.08E-08	0.42
0.0006	0.002731	64.95	0.4632	1.23E-10	0.882
0.0038	0.03077	28.13	0.1346	3.76E-08	0.9706
0.0034	0.009843	32.7	0.2454	9.35E-10	0.3778
0.0006	0.04998	70.25	0.1046	6.12E-16	0.2879
0.0003	0.03101	89.95	0.1314	1.34E-20	0.8367
0.0015	0.003402	44.78	0.404	9.76E-09	0.3922
0.0318	0.02467	10.31	0.1474	6.47E-09	0.1389
0.0004	0.01578	73.46	0.1811	1.75E-29	0.1105
0.001	0.03317	57.96	0.126	7.67E-19	0.8126
0.0009	0.002861	53.18	0.4512	8.73E-13	0.1298
0.0035	0.01469	29.41	0.1892	4.95E-09	0.3321
0.001	0.02319	51.97	0.1518	2.25E-17	0.8188
0.004	0.01545	27.54	0.1887	1.74E-10	0.123
0.0026	0.001873	34.68	0.5478	9.74E-09	0.8756
0.0032	0.01526	36.08	0.1902	1.58E-08	0.7258
0.0012	0.01369	50.43	0.2023	1.21E-19	0.4854
0.0211	0.002332	12.06	0.5154	7.48E-09	0.7533
0.0026	0.03827	33.76	0.1193	4.98E-10	0.4335
0.0001	0.02148	128.3	0.1523	1.15E-56	0.8212
0.0009	0.0206	65.61	0.161	5.51E-22	0.1932
0.0029	0.03413	32.71	0.1248	2.90E-08	0.6506
0.0019	0.01087	38.79	0.2277	1.69E-12	0.4622
0.0026	0.05144	35.34	0.1045	1.00E-09	0.5692
0.0007	0.01997	64.38	0.164	9.46E-12	0.382
0.0013	0.0104	51.12	0.2202	1.55E-14	0.1573
0.0072	0.02264	21.37	0.1535	3.00E-09	0.2348
0.0028	0.02691	33.53	0.1418	1.84E-08	0.0879995
0.0001	0.03207	203.7	0.1286	1.63E-09	0.00852001
0.0021	0.007837	36.97	0.252	5.42E-14	0.5414
0.001	0.02316	52.32	0.1525	4.02E-10	0.291
0.0092	0.02906	18.01	0.1363	6.12E-10	0.9915
0.0565	0.01343	7.987	0.2005	4.03E-08	0.5041
0.0006	0.01918	73.04	0.1703	7.80E-16	0.0781808
0.0012	0.0005627	55.7	1.1831	1.05E-18	0.7838
0.0007	0.04024	61.96	0.1174	2.79E-09	0.2157
0.0003	0.01868	96.39	0.1716	4.65E-14	0.1013
0.01	0.01478	18.29	0.1943	4.20E-18	0.3324
0.0062	0.0399	25.27	0.1168	1.30E-08	0.3828
0.0025	0.009294	35.78	0.2575	4.25E-09	0.5757
0.0207	0.02748	13.23	0.1411	6.37E-09	0.4303
0.0026	0.03	33.82	0.1376	4.06E-08	0.7668
0.0031	0.04196	33.18	0.1139	2.28E-08	0.724001
0.001	0.0008187	51.16	0.9672	1.42E-19	0.8163
0.0031	0.01478	32.76	0.1922	7.31E-10	0.6341
0.0092	0.007947	19.47	0.2737	2.08E-10	0.1627
0.0004	0.01119	79.88	0.2169	2.19E-09	0.9486
0.006	0.01108	22.8	0.2257	1.80E-19	0.0400396
0.0007	0.00341	62.38	0.4174	2.64E-11	0.1195
0.0016	0.01588	42.7	0.1814	3.85E-08	0.2369
0.005	0.00286	26.11	0.4724	3.78E-08	0.5313
0.0018	0.000532	40.79	0.951	3.49E-08	0.7756
0.0004	0.001519	84.75	0.5992	6.83E-42	0.9901
0.0013	0.02312	52.34	0.1564	1.26E-11	0.0882104
0.0021	0.01951	41.59	0.1643	5.64E-16	0.7893
0.0056	0.03293	23.81	0.1298	1.58E-10	0.9578
0.0018	0.004352	41.76	0.3617	7.66E-14	0.7382
0.0018	0.01484	53.53	0.1916	1.07E-13	0.4577

0.0032	0.02167	33.13	0.1554	4.82E-08	0.685499
0.0009	0.02506	57.08	0.1486	1.16E-21	0.7867
0.0007	0.01923	69.68	0.1678	7.54E-32	0.2281
0.0016	0.03616	43.5	0.1219	5.38E-12	0.781101
0.0094	0.02098	18.22	0.157	4.11E-10	0.0283903
0.0079	0.001848	20	0.5693	7.32E-10	0.4467
0.0057	0.01076	22.31	0.221	2.32E-16	0.0341704
0.0044	0.02861	26.38	0.136	1.64E-08	0.3144
0.0018	0.0009084	43.4	0.8267	9.20E-11	0.706801
0.0015	0.03176	44.6	0.1298	4.22E-09	0.7666
0.0001	0.02786	127.9	0.1371	1.21E-55	0.723299
0.0007	0.01444	64.07	0.1873	8.69E-12	0.5107
0.0004	0.01233	78.49	0.2162	1.83E-21	0.4447
0.0013	0.025	46.83	0.1447	1.34E-08	0.4476
0.0022	0.03807	36.09	0.1184	1.20E-12	0.9178
0.0003	0.02668	93.44	0.1448	3.23E-52	0.2472
0.004	0.0132	28.3	0.2096	1.82E-08	0.2647
0.0007	0.00876	63.75	0.237	3.47E-28	0.8574
0.0028	0.04452	36.54	0.1127	2.60E-08	0.9942
0.0022	0.02374	33.81	0.1508	2.09E-23	0.0819691
0.0019	0.01445	38.16	0.1968	3.26E-10	0.635
0.0013	0.002655	47.7	0.4806	3.55E-21	0.4162
0.0007	0.009802	67.24	0.2378	8.73E-17	0.7947
0.0016	0.03206	45.67	0.1322	1.31E-19	0.3743
0.0009	0.02121	55.44	0.1541	5.81E-19	0.3156
0.0025	0.02159	37.13	0.1599	1.91E-12	0.3501
0.0018	0.01456	41.15	0.1922	4.15E-13	0.5112
0.0012	0.02442	52.29	0.1497	1.92E-16	0.8638
0.0073	0.06426	20.92	0.0938	7.76E-15	0.2931
0.0003	0.03499	104	0.1227	2.04E-15	0.7805
0.0015	0.00901	47.64	0.2447	7.72E-11	0.1292
0.0013	0.01901	49.25	0.1641	1.59E-10	0.5188
0.0006	0.0004565	69.14	1.1944	9.38E-26	0.2476
0.0006	0.03891	69.14	0.1191	9.38E-26	0.799
0.0015	0.01896	44.7	0.1693	1.46E-13	0.3548
0.0012	0.02553	47.71	0.1508	2.29E-11	0.2084
0.0001	0.002981	128.5	0.4287	1.74E-56	0.1653
0.0091	0.01009	18.37	0.2378	4.15E-16	0.8329
0.0006	0.04858	64.6	0.1064	1.00E-30	0.2171
0.0028	0.02745	38.25	0.1475	8.14E-10	0.3234
0.007	0.0105	21.11	0.232	8.32E-09	0.767499
0.0021	0.009284	37.84	0.2378	9.60E-14	0.5363
0.0015	0.01517	44.92	0.1824	2.12E-11	0.9719
0.001	0.02648	50.73	0.1426	8.85E-22	0.4901
0.0006	0.02598	64.46	0.1406	3.68E-22	0.2318
0.0085	0.01558	20.15	0.1861	1.26E-11	0.3185
0.0021	0.004137	38.53	0.3776	5.64E-11	0.5126
0.0026	0.02608	33.78	0.1416	1.02E-11	0.9145
0.0013	0.009251	48.59	0.245	3.39E-10	0.760699
0.0032	0.03721	30.9	0.1214	5.04E-09	0.9858
0.0019	0.006042	40.3	0.3157	2.02E-11	0.9797
0.0006	0.006911	67.17	0.2975	4.65E-16	0.0963297
0.0006	0.009832	70.63	0.238	2.03E-35	0.3512
0.0025	0.03283	36.78	0.1309	4.15E-16	0.0339399
0.0007	0.01749	68.52	0.1758	1.71E-27	0.0437703
0.0009	0.02467	59.76	0.1476	9.36E-13	0.3697
0.0001	0.008567	109.5	0.2401	1.70E-27	0.8923
0.0032	0.01828	36.11	0.178	1.13E-11	0.8626

0.0007	0.02753	62.46	0.1434	4.25E-11	0.5974
0.0044	0.007482	26.7	0.2604	2.51E-09	0.7209
0.0032	0.007987	31.44	0.2534	2.59E-08	0.1357
0.0013	0.007855	46.47	0.2652	1.67E-14	0.9444
0.0004	0.02125	79.27	0.1547	6.58E-35	0.00599805
0.01	0.002617	18.08	0.4878	1.42E-11	0.1122
0.0003	0.06652	93.65	0.0925	1.69E-61	0.3436
0.004	0.01706	31.66	0.1754	2.42E-08	0.3708
0.0007	0.002763	60.69	0.4699	2.16E-21	0.8993
0.0009	0.01872	60.77	0.1745	8.35E-13	0.2845
0.004	0.01829	29.36	0.1684	1.20E-10	0.1199
0.0016	0.01526	44.35	0.1918	7.07E-13	0.4838
0.0066	0.04422	22.14	0.1131	2.15E-12	0.8592
0.9988	0.9563	50.03	0.1125	1.11E-14	0.7782
0.0066	0.01584	21.61	0.1836	3.69E-14	0.6511
0.0019	0.01926	44.12	0.1802	4.89E-19	0.3192
0.0026	0.01881	33.34	0.1649	2.42E-10	0.1325
0.0003	0.03902	92.78	0.1184	4.73E-27	0.8573
0.0018	0.001946	41.43	0.5309	7.32E-11	0.341
0.001	0.1363	54.89	0.0662	9.78E-23	0.2116
0.0009	0.0167	53.43	0.1802	3.31E-10	0.9449
0.001	0.01955	55.24	0.1616	1.21E-17	0.628901
0.0035	0.0144	28.76	0.1954	3.53E-08	0.4347
0.0038	0.02925	28.32	0.1357	4.89E-12	0.8997
0.0021	0.006235	38.1	0.2989	1.78E-11	0.4465
0.0034	0.01217	29.42	0.2139	2.34E-11	0.3016
0.0019	0.03554	39.81	0.1229	1.03E-18	0.747699
0.0025	0.004121	38.31	0.3832	9.00E-14	0.8732
0.0018	0.0279	42.26	0.1403	8.66E-17	0.9845
0.0022	0.003825	36.32	0.4014	5.30E-09	0.9903
0.0007	0.009338	59.91	0.241	3.68E-08	0.3896
0.0022	0.004981	38.13	0.3385	1.94E-08	0.9091
0.0037	0.007424	28.33	0.2796	1.47E-08	0.0368799
0.0007	0.03546	60.2	0.1234	4.25E-11	0.0154899
0.0069	0.005057	21.18	0.3232	2.36E-12	0.9456
0.0023	0.003467	36.04	0.4062	4.80E-09	0.5564
0.0015	0.005399	45.09	0.3242	1.62E-15	0.9544
0.0032	0.03206	29.73	0.1328	1.40E-17	0.542199
0.0001	0.008568	129.6	0.2533	8.20E-41	0.8211
0.0022	0.007426	37	0.2698	2.19E-10	0.9115
0.0016	0.03672	43.72	0.1211	1.99E-09	0.3812
0.999	0.9841	57.56	0.1871	2.95E-21	0.1723
0.001	0.02766	53.08	0.1436	2.51E-18	0.9865
0.0006	0.03677	69.32	0.1215	5.22E-17	0.3654
0.0015	0.01838	48.27	0.1664	2.74E-16	0.9302
0.0006	0.007731	80.36	0.2726	1.54E-15	0.1592
0.0013	0.03738	49.05	0.1214	7.02E-18	0.888
0.0012	0.0187	58.11	0.1736	6.19E-11	0.9092
0.0007	0.05015	59.93	0.1049	6.19E-18	0.2415
0.0004	0.004399	76.44	0.3552	2.60E-14	0.3871
0.0003	0.01378	98.17	0.2054	3.69E-24	0.9283
0.0004	0.0255	78.84	0.1441	6.60E-13	0.509
0.0016	0.0262	43.75	0.1472	1.20E-08	0.5208
0.004	0.01551	27.04	0.1831	4.93E-14	0.3498
0.0116	0.02754	17.31	0.1417	5.64E-14	0.9507
0.0029	0.01824	33.18	0.1703	5.41E-11	0.692299
0.0041	0.02564	27.44	0.1428	1.46E-09	0.2635
0.0004	0.03689	82.87	0.1231	2.61E-15	0.612099

0.0076	0.02038	19.43	0.1651	1.16E-10	0.8763
0.0122	0.0127	16.28	0.2079	3.21E-08	0.671001
0.001	0.06206	54.79	0.0948	8.69E-16	0.3587
0.0032	0.01958	31.03	0.1667	2.77E-09	0.4752
0.0004	0.001121	82.54	0.728	2.02E-15	0.5466
0.0022	0.01324	37.83	0.2022	2.00E-08	0.8174
0.0095	0.03164	18.61	0.1324	1.97E-08	0.4582
0.0015	0.008318	44.42	0.2656	1.39E-09	0.7963
0.0012	0.01692	51.47	0.1818	7.89E-16	0.4038
0.0001	0.01884	130.1	0.1662	6.41E-13	0.1197
0.0022	0.0127	36.44	0.2088	5.54E-11	0.5915
0.0029	0.003964	33.71	0.3957	9.17E-10	0.7362
0.0009	0.002867	54.73	0.4501	6.40E-11	0.5213
0.0013	0.02493	46.68	0.1507	5.07E-14	0.9478
0.0006	0.03136	69.63	0.1319	1.22E-15	0.9818
0.0009	0.01404	56.91	0.1947	1.61E-22	0.1479
0.0007	0.01413	62.86	0.1929	1.15E-21	0.1842
0.0004	0.01855	75.09	0.1694	5.08E-17	0.3857
0.0037	0.00201	29.82	0.5588	6.24E-10	0.8268
0.0016	0.05192	45.47	0.1026	2.50E-14	0.2949
0.0003	0.01937	92.57	0.1651	6.37E-20	0.1498
0.9869	0.9853	15.38	0.1912	1.55E-08	0.2452
0.9987	0.9576	48.86	0.112	4.25E-09	0.9626
0.0016	0.02105	44.28	0.1563	4.92E-14	0.4672
0.0001	0.02639	127.1	0.1443	5.00E-107	0.4631
0.0079	0.02736	20.78	0.138	3.35E-10	0.6948
0.0001	0.01206	123.4	0.2141	8.41E-101	0.2241
0.0029	0.03356	35.7	0.1265	1.41E-08	0.3496
0.0088	0.008223	19.44	0.267	4.27E-08	0.1501
0.0004	0.01823	80.9	0.1743	5.64E-15	0.1805
0.0063	0.008168	22.06	0.2529	1.16E-09	0.6912
0.001	0.03774	54.53	0.1216	2.74E-11	0.3575
0.0066	0.03821	21.02	0.1206	3.19E-08	0.5727
0.0012	0.005835	53.26	0.3235	6.51E-19	0.532
0.0018	0.003909	42.64	0.3724	7.23E-11	0.5078
0.0007	0.02372	62.01	0.1506	7.99E-11	0.9623
0.0015	0.02526	45.79	0.1479	2.36E-13	0.1635
0.0012	0.006124	52.07	0.2979	5.06E-17	0.0400304
0.0015	0.03025	45.5	0.1312	1.66E-19	0.2836
0.0015	0.008495	43.92	0.2469	6.96E-11	0.1271
0.0018	0.03904	40.5	0.1202	8.16E-11	0.08041
0.0016	0.0718	41.59	0.0879	1.29E-14	0.5063
0.0018	0.01461	44.43	0.1913	4.29E-12	0.588901
0.0019	0.007971	38.46	0.2785	3.32E-17	0.3491
0.0016	0.02516	41.66	0.1489	1.35E-10	0.7978
0.0037	0.02334	31.51	0.1631	2.46E-09	0.578
0.0018	0.007155	40.47	0.2671	7.89E-17	0.4313
0.0023	0.002789	37.05	0.4408	4.72E-09	0.827
0.0013	0.00687	49.08	0.2771	1.19E-10	0.665001
0.0094	0.0181	18.42	0.1734	1.18E-08	0.1465
0.0015	0.02608	44.95	0.1477	6.36E-15	0.1143
0.0103	0.03732	17.52	0.1197	7.68E-09	0.1018
0.0018	0.05518	40.82	0.1023	3.37E-08	0.8819
0.0097	0.1146	17.32	0.0714	2.61E-14	0.4339
0.0079	0.009412	20.28	0.2525	1.89E-12	0.6446
0.0029	0.003841	33.09	0.3829	3.42E-08	0.4416
0.0128	0.008979	15.15	0.2515	1.30E-10	0.1908
0.0013	0.03326	50.66	0.1271	1.60E-08	0.2225

0.0001	0.04963	134.1	0.1038	4.18E-15	0.9461
0.0023	0.04315	36.29	0.1186	5.23E-09	0.6011
0	0.03086	263.7	0.1348	2.85E-40	0.4177
0.0016	0.02625	45.76	0.1416	3.79E-11	0.661899
0.0016	0.02375	43.55	0.1486	1.07E-11	0.1035
0.0006	0.008882	63.13	0.2559	7.20E-12	0.4442
0.0029	0.01385	31.58	0.1957	1.02E-16	0.511601
0.0022	0.01047	38.87	0.2299	2.06E-17	0.6742
0.0006	0.002231	97.29	0.487	1.79E-57	0.738401
0.0003	0.008833	93.43	0.2515	1.80E-24	0.5461
0.0003	0.006677	107	0.2719	1.54E-15	0.1948
0.0053	0.09565	28.37	0.077	8.71E-09	0.9901
0.0004	0.02592	113.7	0.1458	1.91E-42	0.4292
0.0004	0.01464	84.15	0.1968	3.23E-39	0.1379
0.0073	0.004551	20.64	0.3565	8.60E-12	0.8211
0.0116	0.01747	16.98	0.1798	5.33E-09	0.5109
0.0001	0.02948	132.8	0.139	6.61E-42	0.5807
0.0433	0.01417	8.691	0.1878	4.67E-08	0.4964
0.0004	0.006105	85.45	0.3017	2.03E-19	0.4411
0.0007	0.005822	62.22	0.319	8.89E-11	0.0912494
0.0043	0.03475	26.99	0.1264	4.71E-10	0.2551
0.0012	0.01487	49.95	0.1927	6.43E-15	0.1099
0.0018	0.011	42.76	0.2174	2.29E-12	0.692
0.0161	0.01749	14.27	0.181	2.00E-09	0.6317
0.0031	0.04884	32.62	0.1058	3.18E-09	0.2292
0.1892	0.29	0.02163	0.0503	2.66E-08	0.4717
0.0191	0.04703	0.06626	0.1075	2.71E-09	0.0923103
0	0.02609	2.063	0.1436	5.88E-09	0.145
0.4729	0.602	0.02653	0.0469	3.00E-28	0.9424
0.7204	0.6075	0.03266	0.047	1.42E-09	0.6659
0.1152	0.01396	0.04891	0.1985	1.15E-08	0.513499
0.1642	0.1496	0.04043	0.0671	1.42E-14	0.01494
0.3759	0.4	0.02774	0.0465	1.08E-08	0.3573
0.463	0.4824	0.02696	0.0464	1.37E-12	0.0963696
0.1269	0.01544	0.04595	0.1845	1.49E-13	0.0473304
0.2476	0.4082	0.02954	0.0466	2.91E-65	0.4176
0.124	0.06438	0.03837	0.0942	1.19E-09	0.9579
0.3117	0.1327	0.02967	0.0679	1.72E-08	0.5138
0.1325	0.1866	0.0376	0.0595	9.14E-11	0.2541
0.4557	0.49	0.03022	0.0487	4.00E-76	0.8913
0.0888	0.04555	0.04672	0.112	3.60E-09	0.3618
0.5524	0.4935	0.02741	0.0458	1.55E-08	0.5766
0.3815	0.2061	0.02491	0.0565	2.51E-08	0.3408
0.0971	0.05673	0.04099	0.0996	3.61E-13	0.1615
0.1839	0.008356	0.03712	0.2583	4.52E-09	0.0398602
0.8748	0.8829	0.03723	0.0712	2.88E-08	0.722
0.203	0.1824	0.0315	0.0592	2.00E-09	0.5057
0.2425	0.3066	0.02865	0.0498	9.31E-29	0.2701
0.0972	0.027	0.04238	0.1409	2.83E-08	0.795
0.7067	0.6684	0.03061	0.058	1.77E-08	0.0760099
0.0004	0.05048	3.393	0.1049	3.21E-08	0.5733
0.0019	0.07432	1.725	0.0854	1.62E-08	0.865
0.0003	0.003652	4.296	0.3673	4.81E-10	0.288
0.0023	0.01788	1.585	0.1745	5.47E-09	0.4125
0.0022	0.006809	1.564	0.2859	1.51E-08	0.4315
0.0006	0.02165	3.205	0.162	2.27E-08	0.3786
0.0006	0.01123	3.301	0.2092	1.35E-11	0.4984
0.0016	0.01286	1.782	0.2099	2.50E-09	0.3829

0.0006	0.0029	3.407	0.4615	2.33E-08	0.6389
0.0004	0.004817	3.342	0.336	2.10E-08	0.8796
0.0001	0.00569	5.796	0.3117	1.53E-08	0.634699
0.0001	0.03561	5.804	0.1243	5.41E-09	0.490999
0.0001	0.01246	6.272	0.2079	6.06E-09	0.257
0.0018	0.025	2.243	0.1467	1.98E-08	0.6163
0.0007	0.003013	2.92	0.416	4.60E-08	0.1934
0.0006	0.03653	3.141	0.1186	1.59E-15	0.1471
0.0013	0.05676	1.881	0.0961	1.00E-08	0.5947
0.0006	0.01141	3.154	0.2184	1.58E-13	0.3347
0.0003	0.03052	3.886	0.1353	3.38E-08	0.5121
0.0003	0.03101	3.906	0.1314	9.81E-10	0.8367
0.0001	0.02148	5.736	0.1523	6.30E-09	0.8212
0.0001	0.03207	8.845	0.1286	7.92E-09	0.00852001
0.0001	0.006401	7.413	0.3078	9.65E-09	0.3256
0.0001	0.02786	5.716	0.1371	1.03E-08	0.723299
0.0003	0.02668	4.095	0.1448	3.97E-10	0.2472
0.0003	0.02849	3.924	0.136	3.08E-15	0.4165
0.0001	0.002981	5.744	0.4287	7.31E-09	0.1653
0.0006	0.04858	2.811	0.1064	2.10E-08	0.2171
0.001	0.02648	2.205	0.1426	2.22E-08	0.4901
0.0006	0.009832	3.072	0.238	3.62E-09	0.3512
0.0025	0.03283	1.56	0.1309	4.90E-08	0.0339399
0.0099	0.002617	0.763	0.4878	5.14E-09	0.1122
0.0003	0.06652	4.058	0.0925	4.44E-18	0.3436
0.0019	0.01926	1.85	0.1802	1.58E-11	0.3192
0.0001	0.03867	5.65	0.1158	5.87E-21	0.3788
0	0.01599	6.463	0.1762	2.34E-09	0.7826
0.0092	0.02121	0.8043	0.1553	2.04E-08	0.0950802
0.0019	0.03554	1.681	0.1229	3.74E-08	0.747699
0.0001	0.008568	5.703	0.2533	1.26E-17	0.8211
0.0001	0.01705	5.644	0.1787	5.19E-09	0.525801
0.0003	0.01378	4.31	0.2054	7.31E-13	0.9283
0.0001	0.01884	5.695	0.1662	9.31E-12	0.1197
0.0001	0.02256	4.737	0.1573	9.74E-16	0.7954
0.0001	0.02639	5.751	0.1443	4.82E-19	0.4631
0.0001	0.01206	5.557	0.2141	6.60E-19	0.2241
0.0001	0.01669	5.64	0.1805	1.16E-20	0.1987
0.0015	0.03025	1.94	0.1312	2.13E-11	0.2836
0.0001	0.02344	5.85	0.1482	4.56E-09	0.7009
0.0006	0.018	3.224	0.1743	8.99E-10	0.2358
0.0003	0.01938	4.049	0.1651	3.92E-09	0.1525
0	0.03086	11.59	0.1348	5.21E-18	0.4177
0.0022	0.01047	1.652	0.2299	4.84E-08	0.6742
0.0006	0.002231	4.191	0.487	4.38E-10	0.738401
0.0003	0.006677	4.474	0.2719	4.37E-08	0.1948
0.0004	0.02592	4.922	0.1458	3.94E-14	0.4292
0.0001	0.02948	5.793	0.139	1.44E-09	0.5807
0.0001	0.01697	7.703	0.1794	7.70E-09	0.2188
0.1639	0.2492	0.03229	0.0522	4.61E-13	0.0569102
0.5685	0.3828	0.02391	0.047	1.41E-15	0.753101
0.1639	0.2492	0.03122	0.0522	1.98E-15	0.0569102
0.0354	0.007566	0.06431	0.2613	2.51E-10	0.4245
0.5685	0.3828	0.02306	0.047	1.51E-23	0.753101
0.1639	0.2492	0.03229	0.0522	4.61E-13	0.0569102
0.5685	0.3828	0.02391	0.047	1.41E-15	0.753101
0.328	0.4255	0.01937	0.0466	4.56E-08	0.0537205
0.5294	0.5705	0.02863	0.0461	1.29E-08	0.0560803

0.2464	0.2876	0.03163	0.0507	5.31E-18	0.9298
0.2895	0.4056	0.02869	0.0463	3.14E-14	0.4084
0.5139	0.5759	0.0249	0.0465	3.85E-59	0.858
0.3446	0.318	0.02711	0.0494	1.18E-11	0.301
0.0524	0.003504	0.05593	0.4016	1.14E-20	0.3531
0.4682	0.5392	0.01936	0.0454	0.00E+00	0.713001
0.0675	0.01732	0.05056	0.1799	1.12E-18	0.825
0.2888	0.4056	0.02838	0.0463	1.39E-10	0.4084
0.5141	0.5759	0.02472	0.0465	8.03E-56	0.858
0.3439	0.318	0.02673	0.0494	5.32E-13	0.301
0.4687	0.5392	0.01892	0.0454	0.00E+00	0.714
0.0675	0.01732	0.0499	0.1799	2.70E-21	0.825
0.9472	0.9963	0.0553	0.3874	1.53E-20	0.2708
0.2894	0.4056	0.02875	0.0463	5.63E-11	0.4084
0.514	0.5759	0.02528	0.0465	1.80E-45	0.858
0.2213	0.2727	0.03146	0.0511	3.06E-15	0.4505
0.4687	0.5392	0.02116	0.0454	3.74E-304	0.717
0.0675	0.01732	0.0511	0.1799	4.74E-11	0.825
0.2888	0.4056	0.02836	0.0463	5.61E-11	0.4084
0.5141	0.5759	0.02489	0.0465	1.32E-44	0.858
0.3439	0.318	0.02672	0.0494	2.05E-12	0.301
0.0528	0.003504	0.05532	0.4016	1.08E-15	0.3531
0.4686	0.5392	0.02033	0.0454	0.00E+00	0.727301
0.0675	0.01732	0.05014	0.1799	6.86E-13	0.825
0.6527	0.7737	0.02896	0.0547	2.18E-09	0.562101
0.0187	0.009385	0.1053	0.231	2.34E-17	0.4224
0.0457	0.003723	0.06675	0.3907	1.47E-18	0.0329602
0.0884	0.04667	0.04886	0.1096	4.45E-08	0.2993
0.4728	0.5392	0.0262	0.0454	5.68E-74	0.713801
0.0887	0.01822	0.05995	0.1728	8.86E-18	0.294
0.0346	0.02483	0.09169	0.1486	1.25E-14	0.3053
0.0517	0.00594	0.07979	0.3084	3.18E-10	0.8408
0.061	0.07194	0.07005	0.0886	9.72E-18	0.1341
0.1375	0.1086	0.04978	0.073	7.45E-21	0.9785
0.6486	0.7395	0.03604	0.0518	2.82E-10	0.3339
0.025	0.01548	0.1101	0.1927	1.74E-11	0.3817
0.0872	0.01822	0.04473	0.1728	4.59E-33	0.294
0.0364	0.02483	0.06516	0.1486	3.00E-29	0.3053
0.0527	0.00594	0.05733	0.3084	1.61E-20	0.8408
0.0621	0.07194	0.05103	0.0886	2.15E-23	0.1341
0.6417	0.7395	0.02589	0.0518	5.47E-16	0.3339
0.1407	0.1064	0.03597	0.0738	5.11E-35	0.864
0.0263	0.01548	0.07785	0.1927	6.86E-19	0.3817
0.0874	0.01822	0.04481	0.1728	9.61E-29	0.294
0.0365	0.02483	0.06509	0.1486	3.09E-30	0.3053
0.0526	0.00594	0.05739	0.3084	1.44E-19	0.8408
0.325	0.5036	0.02694	0.0456	6.47E-11	0.5836
0.0622	0.07194	0.05104	0.0886	5.44E-25	0.1341
0.6418	0.7395	0.02591	0.0518	5.08E-17	0.3339
0.1406	0.1064	0.03602	0.0738	4.86E-33	0.864
0.0264	0.01548	0.07772	0.1927	5.94E-20	0.3817
0.0875	0.01822	0.04473	0.1728	5.92E-30	0.294
0.0365	0.02483	0.06509	0.1486	3.48E-30	0.3053
0.0526	0.00594	0.05734	0.3084	1.41E-20	0.8408
0.325	0.5036	0.02694	0.0456	3.64E-10	0.5836
0.0622	0.07194	0.05105	0.0886	1.87E-24	0.1341
0.6419	0.7395	0.0259	0.0518	5.51E-17	0.3339
0.1407	0.1064	0.036	0.0738	3.06E-33	0.864

0.0264	0.01548	0.07772	0.1927	1.11E-19	0.3817
0.0874	0.01822	0.0448	0.1728	3.32E-28	0.294
0.0365	0.02483	0.065	0.1486	2.97E-31	0.3053
0.099	0.08743	0.03362	0.081	0.00E+00	0.484
0.0526	0.00594	0.05731	0.3084	9.77E-21	0.8408
0.325	0.5036	0.02691	0.0456	2.29E-11	0.5836
0.0622	0.07194	0.051	0.0886	3.97E-25	0.1341
0.6418	0.7395	0.0259	0.0518	3.82E-17	0.3339
0.1406	0.1064	0.03601	0.0738	3.27E-32	0.864
0.0264	0.01548	0.07763	0.1927	1.10E-20	0.3817
0.0874	0.01822	0.04491	0.1728	1.23E-28	0.294
0.0365	0.02483	0.06516	0.1486	2.27E-30	0.3053
0.0526	0.00594	0.05747	0.3084	1.09E-19	0.8408
0.325	0.5036	0.02696	0.0456	5.25E-12	0.5836
0.0622	0.07194	0.05113	0.0886	2.84E-24	0.1341
0.6418	0.7395	0.02596	0.0518	1.16E-16	0.3339
0.1406	0.1064	0.03609	0.0738	1.22E-32	0.864
0.0264	0.01548	0.0778	0.1927	1.78E-20	0.3817
0.0874	0.01822	0.04476	0.1728	4.65E-32	0.294
0.0365	0.02483	0.06518	0.1486	1.97E-29	0.3053
0.099	0.08743	0.03385	0.081	0.00E+00	0.484
0.0526	0.00594	0.05744	0.3084	2.50E-20	0.8408
0.3253	0.5036	0.02698	0.0456	1.86E-10	0.5836
0.0622	0.07194	0.05113	0.0886	1.71E-23	0.1341
0.6419	0.7395	0.02594	0.0518	2.44E-16	0.3339
0.1407	0.1064	0.03602	0.0738	3.75E-34	0.864
0.0264	0.01548	0.07783	0.1927	1.97E-19	0.3817
0.0873	0.01822	0.0449	0.1728	1.86E-25	0.294
0.0365	0.02483	0.06556	0.1486	2.04E-24	0.3053
0.0525	0.00594	0.05763	0.3084	1.73E-16	0.8408
0.0622	0.07194	0.05131	0.0886	7.95E-21	0.1341
0.6419	0.7395	0.026	0.0518	5.84E-16	0.3339
0.1406	0.1064	0.03616	0.0738	1.32E-28	0.864
0.099	0.08647	0.03524	0.0813	3.43E-282	0.449
0.0264	0.01548	0.07803	0.1927	1.94E-17	0.3817
0.0874	0.01822	0.04486	0.1728	1.40E-27	0.294
0.0365	0.02483	0.0653	0.1486	3.37E-29	0.3053
0.0527	0.00594	0.05757	0.3084	7.25E-17	0.8408
0.0622	0.07194	0.05119	0.0886	1.95E-23	0.1341
0.3342	0.5043	0.02702	0.0457	1.93E-08	0.585901
0.6418	0.7395	0.02597	0.0518	7.67E-18	0.3339
0.1406	0.1064	0.03608	0.0738	6.31E-32	0.864
0.0996	0.08693	0.03455	0.0811	0.00E+00	0.4555
0.0264	0.01548	0.0779	0.1927	3.78E-19	0.3817
0.5726	0.6967	0.02309	0.0497	9.71E-09	0.5909
0.7661	0.7338	0.02546	0.0517	3.08E-70	0.0563495
0.3813	0.4421	0.0294	0.0551	9.31E-09	0.1011
0.4182	0.4821	0.02426	0.0454	1.46E-09	0.1199
0.1007	0.1827	0.04195	0.0596	5.73E-32	0.821
0.5141	0.5759	0.02502	0.0465	2.04E-56	0.858
0.0528	0.003504	0.05578	0.4016	3.17E-23	0.3531
0.4262	0.4946	0.01935	0.0455	0.00E+00	0.627001
0.3	0.2515	0.02842	0.0528	1.28E-10	0.2892
0.0588	0.01548	0.05417	0.1886	7.59E-25	0.8607
0.1007	0.1827	0.04256	0.0596	2.76E-26	0.821
0.514	0.5759	0.02528	0.0465	3.19E-56	0.858
0.9692	0.993	0.07576	0.2797	1.52E-08	0.630999
0.2213	0.2727	0.03174	0.0511	1.33E-15	0.4505

0.4073	0.5273	0.02661	0.0456	4.74E-19	0.7571
0.6348	0.662	0.02725	0.0486	5.38E-09	0.2447
0.574	0.5056	0.02069	0.0455	0.00E+00	0.6805
0.1009	0.1827	0.04007	0.0596	4.50E-28	0.821
0.5141	0.5759	0.02378	0.0465	3.17E-60	0.858
0.4259	0.4946	0.01893	0.0455	0.00E+00	0.6276
0.4068	0.5273	0.0251	0.0456	1.06E-21	0.7571
0.0211	0.01338	0.08639	0.2026	7.61E-15	0.1067
0.9472	0.9963	0.0533	0.3874	2.86E-22	0.2708
0.1007	0.1827	0.04083	0.0596	6.27E-39	0.821
0.5141	0.5759	0.02417	0.0465	2.50E-73	0.858
0.9693	0.993	0.07322	0.2797	1.08E-08	0.630999
0.0528	0.003504	0.0542	0.4016	1.49E-31	0.3531
0.6359	0.662	0.0262	0.0486	2.44E-16	0.2447
0.0716	0.02708	0.0485	0.1441	3.13E-08	0.781
0.5738	0.5053	0.01733	0.0454	0.00E+00	0.6363
0.0588	0.01548	0.05272	0.1886	8.25E-31	0.8607
0.5276	0.4608	0.02665	0.0454	7.84E-28	0.7274
0.4865	0.2828	0.02905	0.061	1.11E-12	0.5346
0.4796	0.6244	0.029	0.0478	2.73E-09	0.696899
0.4269	0.4946	0.02713	0.0455	3.43E-16	0.627899
0.1482	0.2489	0.03815	0.0529	1.41E-11	0.1369
0.4466	0.26	0.02517	0.0523	5.30E-108	0.5358
0.0324	0.05182	0.07604	0.1042	3.51E-09	0.945
0.5156	0.5759	0.0273	0.0465	1.78E-43	0.858
0.4053	0.5273	0.02819	0.0456	2.19E-16	0.7571
0.3848	0.3462	0.02839	0.0477	3.43E-21	0.6829
0.5709	0.5053	0.02079	0.0454	0.00E+00	0.6363
0.5156	0.5759	0.02621	0.0465	4.05E-64	0.858
0.3854	0.3468	0.02778	0.0477	1.54E-27	0.6838
0.4053	0.5273	0.02755	0.0456	1.82E-21	0.7571
0.0211	0.01338	0.09629	0.2026	3.84E-09	0.1067
0.4719	0.5392	0.01665	0.0454	0.00E+00	0.713099
0.9466	0.9958	0.05908	0.3665	1.06E-13	0.5316
0.5156	0.5759	0.02725	0.0465	7.28E-47	0.858
0.4288	0.4946	0.02097	0.0455	0.00E+00	0.6276
0.3854	0.3468	0.02836	0.0477	2.24E-23	0.6838
0.4053	0.5273	0.02817	0.0456	6.97E-18	0.7571
0.8479	0.6771	0.03859	0.0488	1.32E-08	0.851
0.5153	0.5759	0.02737	0.0465	1.87E-12	0.858
0.3851	0.3462	0.02761	0.0477	3.22E-11	0.6829
0.5156	0.5759	0.02758	0.0465	7.66E-09	0.858
0.0187	0.009385	0.1041	0.231	3.45E-11	0.4224
0.0457	0.003723	0.06591	0.3907	4.44E-15	0.0329602
0.4719	0.5392	0.02537	0.0454	5.03E-95	0.713099
0.5717	0.8191	0.02793	0.0594	1.67E-26	0.0233201
0.5141	0.5759	0.02377	0.0465	4.54E-61	0.858
0.0528	0.003504	0.05308	0.4016	2.14E-25	0.3531
0.2999	0.2515	0.02702	0.0528	3.88E-14	0.2892
0.4686	0.5393	0.01764	0.0454	0.00E+00	0.7212
0.0674	0.01732	0.04825	0.1799	2.12E-18	0.825
0.1106	0.1393	0.03952	0.0663	3.83E-08	0.6193
0.3258	0.5036	0.02554	0.0456	7.00E-84	0.5836
0.0455	0.02257	0.06056	0.1503	1.58E-09	0.3889
0.146	0.2525	0.0334	0.0524	7.30E-96	0.6859
0.2439	0.1955	0.03721	0.0576	6.15E-33	0.9393
0.2082	0.3629	0.03172	0.0475	7.06E-09	0.5005
0.5911	0.6569	0.02624	0.048	5.23E-16	0.8306

0.0044	0.07472	0.1913	0.0865	1.55E-13	0.9393
0.0145	0.1071	0.1074	0.0746	4.42E-09	0.4781
0.2082	0.3629	0.03172	0.0475	4.89E-08	0.5005
0.5911	0.6569	0.02627	0.048	1.36E-13	0.8306
0.0044	0.07472	0.1912	0.0865	4.29E-13	0.9393
0.0145	0.1071	0.1074	0.0746	1.58E-08	0.4781
0.2083	0.3629	0.02982	0.0475	2.56E-14	0.5005
0.5475	0.2659	0.02745	0.0518	2.40E-20	0.1757
0.0323	0.01568	0.07427	0.1991	4.45E-14	0.1852
0.3055	0.4841	0.02878	0.0457	1.59E-28	0.2911
0.0816	0.007247	0.04932	0.2823	7.71E-09	0.888
0.0124	0.01003	0.1208	0.2313	1.11E-10	0.702901
0.0308	0.04103	0.07632	0.1158	8.59E-10	0.1532
0.1086	0.01183	0.03832	0.2117	6.93E-155	0.453
0.552	0.2659	0.03646	0.0518	8.84E-11	0.1757
0.3428	0.3054	0.03665	0.0494	1.68E-12	0.4608
0.022	0.009927	0.08253	0.2334	3.35E-08	0.4938
0.2581	0.362	0.02764	0.0474	1.03E-22	0.7972
0.1514	0.1234	0.03486	0.0699	1.67E-09	0.4498
0.555	0.6242	0.02522	0.0473	1.54E-08	0.7304
0.325	0.5036	0.02661	0.0456	2.77E-15	0.5836
0.6318	0.73	0.02507	0.0511	3.83E-75	0.9949
0.5339	0.6136	0.02456	0.0467	7.42E-17	0.3091
0.0562	0.01053	0.05356	0.2321	2.74E-29	0.244
0.035	0.01079	0.06821	0.2353	1.20E-09	0.1699
0.469	0.3835	0.02574	0.0469	6.46E-09	0.0243198
0.076	0.1943	0.04909	0.0592	7.78E-11	0.0748497
0.0133	0.05822	0.109	0.0989	5.10E-10	0.271
0.015	0.001029	0.1006	0.7739	1.35E-08	0.1683
0.1328	0.0614	0.03627	0.0966	6.58E-58	0.3182
0.3268	0.5036	0.02582	0.0456	1.86E-48	0.5836
0.0453	0.0161	0.05885	0.1861	2.30E-15	0.2973
0.3064	0.3294	0.02775	0.0488	1.83E-09	0.02063
0.3064	0.3294	0.02779	0.0488	3.30E-18	0.02063
0.2194	0.2686	0.04157	0.0516	1.37E-17	0.0379001
0.0918	0.004023	0.06776	0.3601	1.54E-09	0.0852001
0.3574	0.6809	0.04076	0.0497	1.29E-18	0.21
0.2193	0.2686	0.04076	0.0516	2.14E-23	0.0379001
0.7156	0.5149	0.04306	0.0468	2.12E-08	0.4307
0.0917	0.004023	0.06679	0.3601	4.65E-09	0.0852001
0.3575	0.6809	0.04014	0.0497	8.64E-20	0.21
0.4017	0.2858	0.0252	0.0505	9.08E-10	0.5391
0.3458	0.05991	0.03241	0.0971	4.59E-09	0.8941
0.1514	0.1234	0.03488	0.0699	1.25E-08	0.4498
0.1224	0.01739	0.03761	0.1729	1.11E-11	0.5104
0.6318	0.73	0.0249	0.0511	1.88E-87	0.9949
0.1671	0.1852	0.03344	0.0591	2.13E-10	0.2733
0.0562	0.01053	0.05333	0.2321	1.49E-35	0.244
0.0272	0.05897	0.07713	0.0978	2.95E-08	0.5975
0.0304	0.04511	0.0725	0.1093	1.77E-08	0.4341
0.035	0.01079	0.06803	0.2353	1.57E-10	0.1699
0.1514	0.1234	0.03444	0.0699	1.30E-13	0.4498
0.1224	0.01739	0.03725	0.1729	5.23E-12	0.5104
0.555	0.6242	0.02494	0.0473	6.20E-09	0.7304
0.325	0.5036	0.02632	0.0456	7.07E-17	0.5836
0.6318	0.73	0.02417	0.0511	8.47E-120	0.9949
0.1671	0.1852	0.03299	0.0591	2.87E-15	0.2733
0.0239	0.03176	0.07925	0.1276	1.45E-08	0.0908594

0.0562	0.01053	0.05246	0.2321	3.63E-46	0.244
0.035	0.01079	0.06717	0.2353	1.07E-13	0.1699
0.1136	0.01493	0.03939	0.19	2.67E-08	0.461
0.6669	0.8037	0.02627	0.0576	7.02E-09	0.6317
0.1513	0.1234	0.03485	0.0699	1.25E-08	0.4498
0.0718	0.01545	0.04922	0.1842	3.82E-08	0.9478
0.1224	0.01739	0.03759	0.1729	4.48E-11	0.5104
0.3251	0.5036	0.02671	0.0456	3.13E-09	0.5836
0.167	0.1852	0.03338	0.0591	6.60E-15	0.2733
0.0561	0.01053	0.05306	0.2321	8.02E-44	0.244
0.6313	0.729	0.02498	0.051	5.93E-78	0.9652
0.0272	0.05897	0.077	0.0978	1.39E-08	0.5975
0.4557	0.49	0.03152	0.0487	2.78E-08	0.8913
0.035	0.01079	0.06804	0.2353	9.62E-14	0.1699
0.1514	0.1234	0.03467	0.0699	2.23E-15	0.4498
0.0718	0.01545	0.0492	0.1842	1.69E-08	0.9478
0.1224	0.01739	0.03747	0.1729	1.93E-15	0.5104
0.555	0.6242	0.02512	0.0473	8.52E-11	0.7304
0.325	0.5036	0.02635	0.0456	2.48E-28	0.5836
0.6318	0.73	0.02439	0.0511	2.53E-118	0.9949
0.1671	0.1852	0.03313	0.0591	3.76E-21	0.2733
0.0239	0.03176	0.07974	0.1276	2.10E-11	0.0908594
0.0562	0.01053	0.05313	0.2321	1.02E-38	0.244
0.035	0.01079	0.06777	0.2353	2.09E-12	0.1699
0.1514	0.1234	0.03426	0.0699	1.59E-15	0.4498
0.1224	0.01739	0.03709	0.1729	1.33E-12	0.5104
0.555	0.6242	0.02479	0.0473	5.03E-11	0.7304
0.325	0.5036	0.02626	0.0456	1.14E-14	0.5836
0.6318	0.73	0.02368	0.0511	6.21E-147	0.9949
0.1671	0.1852	0.03291	0.0591	1.29E-10	0.2733
0.0239	0.03176	0.07874	0.1276	1.39E-11	0.0908594
0.0562	0.01053	0.05259	0.2321	3.42E-35	0.244
0.035	0.01079	0.06693	0.2353	4.84E-10	0.1699
0.0057	0.008924	0.2236	0.2495	3.04E-08	0.2054
0.5909	0.6569	0.02443	0.048	3.92E-08	0.8306
0.0044	0.07472	0.1759	0.0865	8.87E-10	0.9393
0.2969	0.1244	0.02599	0.0686	3.13E-13	0.7019
0.5907	0.6569	0.0267	0.048	3.74E-12	0.8306
0.0044	0.07472	0.1929	0.0865	4.23E-12	0.9393
0.2972	0.1244	0.02843	0.0686	2.15E-16	0.7019
0.5925	0.6569	0.02636	0.048	6.84E-22	0.8306
0.0041	0.07472	0.1979	0.0865	4.74E-14	0.9393
0.0033	0.01555	0.2396	0.1806	4.28E-11	0.3913
0.0149	0.01898	0.1104	0.1714	3.00E-10	0.2398
0.1947	0.01045	0.04474	0.2224	1.38E-08	0.858
0.1993	0.1823	0.03165	0.0594	3.07E-08	0.490601
0.0917	0.05364	0.04216	0.1022	1.03E-11	0.0685804
0.2425	0.3066	0.0286	0.0498	8.18E-23	0.2701
0.8737	0.8895	0.03693	0.0736	7.67E-09	0.6296
0.1124	0.01404	0.05167	0.1985	1.32E-08	0.4787
0.0001	0.02101	0.7922	0.1588	1.91E-08	0.2136
0.0001	0.006175	0.6021	0.3241	1.25E-08	0.3678
0.0001	0.01536	0.6773	0.1879	2.44E-08	0.1799
0.0001	0.01948	0.8459	0.168	1.92E-08	0.6479
0	0.01241	1.404	0.2057	1.37E-08	0.2265
0.0001	0.01916	0.7543	0.1643	1.60E-08	0.4207
0.0192	0.04703	0.06819	0.1075	3.97E-09	0.0923103
0	0.015	1.583	0.1838	9.88E-09	0.6888

0	0.01115	1.55	0.2176	1.83E-08	0.3764
0.0004	0.01706	0.2979	0.1808	1.17E-09	0.3149
0.0001	0.003444	0.6914	0.4314	1.94E-08	0.9982
0.0001	0.01218	0.6683	0.206	1.63E-08	0.5877
0	0.02147	1.424	0.1618	4.27E-08	0.7826
0.0001	0.01393	1.199	0.1951	3.22E-08	0.542099
0.0001	0.01406	0.7251	0.205	4.54E-08	0.00490897
0.0001	0.04819	0.6678	0.1068	1.44E-08	0.0947305
0.0001	0.009705	0.6612	0.2396	8.68E-09	0.539699
0.0001	0.0231	0.6683	0.1543	1.63E-08	0.6612
0.0001	0.0277	0.67	0.146	3.11E-08	0.3049
0.0001	0.0271	0.6967	0.142	1.60E-08	0.631801
0.0001	0.03123	0.8386	0.1297	5.92E-09	0.8572
0	0.01026	1.346	0.2365	1.80E-08	0.2402
0.0001	0.0212	1.126	0.1589	8.69E-09	0.5461
0.0001	0.01076	0.7897	0.2294	1.13E-08	0.756201
0.0001	0.002281	0.6368	0.4835	3.12E-08	0.3532
0.0001	0.00465	0.8097	0.3461	2.34E-09	0.7052
0.0001	0.0156	0.6399	0.189	3.59E-10	0.8404
0	0.03402	2.197	0.1264	5.06E-10	0.01392
0.0001	0.004404	0.7256	0.3745	1.60E-08	0.3474
0.0001	0.02259	0.6805	0.1533	1.59E-08	0.9535
0.0001	0.02127	0.8599	0.1614	1.53E-08	0.9082
0.0001	0.01913	0.6771	0.1632	1.63E-08	0.8763
0.0001	0.004045	0.6644	0.3691	1.72E-08	0.7605
0.0001	0.02308	0.8111	0.1502	8.65E-09	0.781
0.0427	0.04467	0.06661	0.1101	7.57E-11	0.7071
0.1266	0.05503	0.04341	0.0994	7.21E-11	0.4454
0.228	0.2843	0.03189	0.0504	1.80E-15	0.6656
0.4762	0.2522	0.02804	0.0607	8.08E-12	0.0960904
0.4853	0.3897	0.02755	0.0468	2.20E-09	0.3381
0.2982	0.2507	0.02938	0.0524	3.14E-36	0.7486
0.0844	0.04891	0.04471	0.1071	1.72E-145	0.953
0.1975	0.05865	0.03441	0.098	1.45E-09	0.8625
0.0637	0.01496	0.05614	0.1875	1.64E-13	0.2532
0.0212	0.0311	0.0981	0.1319	4.35E-09	0.5098
0.0001	0.01039	4.684	0.2305	2.22E-08	0.2098
0.0003	0.008699	3.62	0.267	5.93E-11	0.3848
0.0003	0.01669	3.541	0.1884	2.61E-19	0.1668
0.0001	0.02383	4.486	0.1488	2.09E-11	0.9354
0.0001	0.01553	7.59	0.1863	2.92E-10	0.722099
0.0015	0.01707	1.738	0.1772	2.15E-08	0.626299
0.0004	0.003985	2.719	0.3626	1.62E-09	0.1049
0.0004	0.00608	3.261	0.3098	4.54E-14	0.9477
0.0006	0.002347	2.869	0.4887	3.65E-08	0.0585706
0.0047	0.0242	0.8953	0.1472	1.95E-09	0.5303
0.2185	0.2684	0.1686	0.0558	1.37E-10	0.1617
0.0072	0.03469	0.7441	0.1249	2.66E-09	0.7514
0.0004	0.0008742	3.685	0.8495	3.68E-08	0.1831
0.0003	0.01027	3.318	0.226	9.78E-09	0.9254
0.0004	0.021	2.979	0.1609	2.46E-12	0.0506804
0.0057	0.04981	0.79	0.1057	7.53E-09	0.745501
0.0003	0.005156	3.766	0.3261	1.47E-09	0.6957
0.0003	0.001378	3.766	0.6468	1.47E-09	0.6052
0.0003	0.02874	3.673	0.1338	2.35E-11	0.3212
0.0009	0.03815	2.018	0.1219	4.39E-10	0.768199
0.0015	0.002659	1.615	0.4623	1.08E-08	0.3183
0.0003	0.01136	3.188	0.2282	1.04E-08	0.0228302

0.0003	0.007195	3.333	0.2648	9.41E-10	0.6214
0.0001	0.04281	4.27	0.111	1.66E-09	0.8398
0.001	0.01323	1.879	0.2167	1.67E-09	0.5309
0.0001	0.01787	4.91	0.1691	1.95E-10	0.0766496
0.0001	0.05876	8.178	0.0954	2.17E-10	0.572
0.0001	0.02024	4.936	0.1616	2.03E-09	0.8412
0.0003	0.03309	3.911	0.1279	2.31E-14	0.1148
0.0029	0.004329	1.098	0.3665	1.57E-10	0.4661
0.0003	0.05963	3.305	0.0943	2.50E-10	0.2165
0.0003	0.0241	3.429	0.1515	1.57E-16	0.6215
0.0018	0.001548	1.499	0.622	2.98E-13	0.4476
0.0032	0.03261	1.049	0.1323	1.52E-09	0.8492
0.0003	0.009892	3.324	0.226	1.37E-08	0.6494
0.0012	0.01777	1.865	0.1665	1.64E-08	0.00733804
0.0006	0.03298	53.88	0.1256	1.95E-08	0.1369
0.0025	0.01151	24.38	0.222	1.82E-08	0.0507902
0.0043	0.02316	19.64	0.1523	3.21E-10	0.994
0.0076	0.004316	15.24	0.3595	9.60E-09	0.679401
0.0003	0.006687	76.59	0.2873	7.32E-09	0.7934
0.0009	0.04173	45.35	0.1112	2.84E-10	0.6806
0.0006	0.00947	57.03	0.2396	1.48E-09	0.1928
0.0004	0.006754	63.56	0.2912	3.90E-12	0.6233
0.0001	0.02383	96.85	0.1488	1.16E-09	0.9354
0.0022	0.02511	29.35	0.1479	1.92E-09	0.8125
0.0001	0.01553	163.9	0.1863	2.52E-09	0.722099
0.0003	0.03165	75.02	0.1277	6.72E-09	0.7474
0.0003	0.008843	77.86	0.2475	7.79E-09	0.1025
0.0009	0.01369	45.23	0.2122	5.10E-10	0.9524
0.0015	0.01707	36.73	0.1772	7.23E-09	0.626299
0.0006	0.01123	58.05	0.2092	4.14E-11	0.4984
0.0001	0.03453	98.9	0.1247	6.57E-11	0.5409
0.0022	0.006729	28.26	0.2804	1.12E-09	0.1473
0.0075	0.01486	15.32	0.1941	4.04E-08	0.03743
0.0025	0.09396	26.69	0.0775	2.42E-09	0.0152901
0.0018	0.02138	32.14	0.1639	4.11E-10	0.1691
0.0013	0.01366	39.98	0.2026	1.55E-11	0.684699
0.0022	0.009754	34.06	0.2309	1.63E-08	0.936
0.0004	0.02407	66.04	0.1548	3.57E-08	0.9477
0.0004	0.00608	70.2	0.3098	2.03E-11	0.9477
0.0004	0.02194	68.87	0.1575	2.31E-08	0.8022
0.0035	0.0115	20.58	0.2203	1.24E-10	0.537
0.0003	0.003694	82.97	0.3915	2.71E-08	0.3847
0.001	0.02472	42.11	0.148	3.63E-08	0.02913
0	0.0227	391	0.1577	2.46E-08	0.382
0.0015	0.02228	34.49	0.1552	4.09E-08	0.1862
0.2188	0.2684	3.563	0.0558	1.74E-10	0.1617
0	0.03737	304.6	0.1181	3.88E-08	0.3112
0.0004	0.005701	67.08	0.3018	2.24E-21	0.8335
0.0048	0.01656	19.7	0.1848	1.89E-08	0.4562
0.0001	0.01777	98.43	0.1764	1.17E-15	0.2155
0.0004	0.002088	58.31	0.5593	2.46E-15	0.8379
0.0007	0.0466	45.06	0.1099	9.70E-09	0.8059
0.0147	0.02372	10.98	0.1503	9.24E-11	0.3521
0.0001	0.01402	98.17	0.1989	2.17E-08	0.5276
0.0028	0.03515	24.76	0.1259	9.76E-10	0.0571202
0.0037	0.04732	22.5	0.1083	2.87E-10	0.774599
0.0025	0.01734	26.59	0.1695	1.33E-09	0.2008
0.0001	0.02148	100.5	0.1523	4.68E-14	0.8212

0.0004	0.001906	84.21	0.5781	4.51E-11	0.4759
0.0009	0.0153	43.23	0.1892	2.11E-09	0.1945
0.0003	0.01433	71.5	0.1988	1.23E-16	0.2888
0.0006	0.01504	56.32	0.1918	1.42E-08	0.3902
0.001	0.0285	41.16	0.1378	1.15E-09	0.0687907
0.0001	0.02077	100.6	0.1559	6.76E-13	0.3006
0.0003	0.0348	74.81	0.1234	1.06E-08	0.600101
0	0.001206	227.4	0.6469	2.03E-08	0.6398
0.0026	0.03	25.16	0.1376	4.22E-09	0.7668
0.0007	0.01699	47.21	0.1746	1.66E-09	0.853
0.0001	0.02786	100.1	0.1371	7.82E-14	0.723299
0.0057	0.04981	16.87	0.1057	2.27E-12	0.745501
0.0001	0.01338	98.95	0.2058	4.71E-20	0.8219
0.0003	0.005156	81.22	0.3261	6.10E-09	0.6957
0.0003	0.001378	81.22	0.6468	6.10E-09	0.6052
0.0141	0.003683	11.2	0.4116	4.77E-08	0.679
0.0007	0.01234	49.32	0.2134	1.09E-12	0.7993
0.0004	0.001393	58.46	0.6663	1.81E-19	0.813
0.0007	0.01232	53.66	0.2083	3.73E-10	0.1169
0.0004	0.02563	60.78	0.1446	4.24E-11	0.490399
0.0001	0.006139	90.65	0.2918	1.50E-11	0.1023
0.0009	0.0177	43.68	0.1726	2.82E-21	0.0689398
0.0001	0.002981	100.6	0.4287	5.25E-14	0.1653
0.2372	0.0221	4.094	0.1537	2.31E-08	0.8055
0.0053	0.03685	18.3	0.1199	3.25E-08	0.2995
0.0006	0.02616	56.2	0.1438	5.40E-09	0.6109
0.0003	0.004676	130	0.348	1.39E-14	0.9963
0.0006	0.0007598	56.32	1.0227	3.92E-09	0.7888
0.0003	0.007912	71.9	0.2585	2.39E-08	0.7867
0.0003	0.007808	114.1	0.2703	1.06E-08	0.1062
0.0037	0.05163	21.65	0.1056	1.80E-10	0.4577
0.0009	0.03815	43.13	0.1219	2.34E-13	0.768199
0.0007	0.02801	48.87	0.1393	2.16E-09	0.3036
0.9991	0.9832	42.74	0.1868	2.40E-20	0.9329
0.0009	0.06236	45.57	0.0932	1.91E-08	0.6509
0.0003	0.03902	71.13	0.1184	3.87E-09	0.8573
0.0003	0.022	74.65	0.1537	2.58E-08	0.5018
0.0001	0.01482	102	0.1955	4.91E-08	0.8848
0.0004	0.07938	98.8	0.0864	3.01E-08	0.8513
0.0001	0.04281	91.57	0.111	4.50E-15	0.8398
0.0003	0.0485	71.96	0.1064	2.09E-08	0.8389
0.0023	0.01095	27.52	0.2183	9.07E-11	0.6454
0.0001	0.01787	106	0.1691	1.37E-09	0.0766496
0.0007	0.03421	50.18	0.1262	3.93E-09	0.3533
0.0001	0.05876	176.6	0.0954	1.78E-09	0.572
0.001	0.01825	42.22	0.1746	2.78E-09	0.8929
0.0001	0.02024	106.6	0.1616	1.11E-08	0.8412
0.0003	0.03309	84.77	0.1279	3.56E-12	0.1148
0.0006	0.0006401	58.23	0.966	1.68E-09	0.0701504
0.0007	0.004708	59.27	0.3573	2.64E-10	0.8193
0.0022	0.03683	26.88	0.1233	6.76E-12	0.0787608
0.0006	0.02768	53.02	0.1396	1.87E-09	0.2858
0.0003	0.01562	72.98	0.183	3.29E-11	0.4691
0.0001	0.03127	99.63	0.1307	3.23E-09	0.6859
0.004	0.05743	21.68	0.0982	1.43E-08	0.9257
0.0004	0.001121	61.74	0.728	5.84E-11	0.5466
0.0073	0.005086	15.94	0.3581	2.66E-08	0.1888
0.0003	0.0241	73.99	0.1515	4.03E-13	0.6215

0.0007	0.03759	46.74	0.122	1.55E-09	0.3387
0.0022	0.01575	27.77	0.1901	2.26E-11	0.681999
0.0018	0.001548	32.07	0.622	2.83E-12	0.4476
0.0032	0.03261	22.53	0.1323	8.23E-12	0.8492
0.0035	0.07302	23.48	0.0876	3.93E-10	0.2639
0.0016	0.02106	33.11	0.1615	1.74E-08	0.963
0.0006	0.01251	50.77	0.2033	2.11E-08	0.02031
0.0006	0.01727	61.08	0.1866	5.76E-10	0.4475
0.0072	0.01673	15.71	0.1816	4.64E-12	0.284
0.0004	0.02892	56.41	0.1397	2.28E-10	0.6859
0.0013	0.03326	37.24	0.1271	1.90E-12	0.2225
0.0125	0.004147	11.52	0.3745	1.29E-10	0.3848
0.0003	0.01148	70.79	0.2147	4.14E-08	0.531401
0.001	0.01034	42.01	0.2389	1.26E-08	0.6284
0.0004	0.02592	86.78	0.1458	2.08E-09	0.4292
0.0004	0.0252	71.92	0.1497	3.50E-08	0.2265
0.0003	0.01935	82.05	0.1737	3.97E-10	0.9263
0.0004	0.01683	66.09	0.179	2.59E-08	0.679
0.0034	0.03069	22.13	0.1358	6.54E-09	0.723
0	0.05031	252.5	0.104	1.55E-10	0.3586
0.034	0.007596	0.06902	0.2617	2.63E-09	0.8534
0.0758	0.007212	0.04775	0.2818	6.85E-11	0.467599
0.0453	0.007744	0.06275	0.2603	2.69E-08	0.7437
0.3264	0.5036	0.02548	0.0456	1.29E-90	0.5836
0.0456	0.02257	0.06064	0.1503	3.04E-11	0.3889
0.1838	0.1302	0.0324	0.0675	5.95E-09	0.7722
0.2737	0.1558	0.02812	0.0631	7.57E-33	0.6664
0.1464	0.2525	0.03346	0.0524	5.03E-94	0.6859
0.0409	0.006844	0.0628	0.2828	2.01E-41	0.1982
0.0044	0.07472	0.1851	0.0865	1.23E-09	0.9393
0.0317	0.01393	0.07181	0.1959	2.94E-26	0.3054
0.0263	0.01428	0.07787	0.1929	4.91E-21	0.0241502
0.0666	0.02073	0.05006	0.1623	6.09E-46	0.896
0.0209	0.07624	0.08601	0.0853	6.83E-09	0.346
0.0653	0.01568	0.0507	0.1842	2.14E-08	0.7886
0.4267	0.4947	0.02025	0.0455	0.00E+00	0.6534
0.0464	0.003478	0.05812	0.4035	5.35E-29	0.3465
0.041	0.006844	0.06471	0.2828	4.66E-29	0.1982
0.0029	0.002813	0.2262	0.4612	2.14E-08	0.3419
0.0317	0.01393	0.07397	0.1959	9.35E-20	0.3054
0.0462	0.003723	0.06081	0.3907	1.23E-41	0.0329602
0.3594	0.3468	0.0273	0.0477	9.08E-16	0.6838
0.0667	0.02073	0.0518	0.1623	9.80E-33	0.896
0.4688	0.5393	0.01915	0.0454	0.00E+00	0.716901
0.0483	0.0771	0.05873	0.0864	1.13E-27	0.2487
0.0409	0.006844	0.06583	0.2828	5.69E-28	0.1982
0.0317	0.01393	0.07506	0.1959	2.79E-21	0.3054
0.0461	0.003723	0.06178	0.3907	3.23E-42	0.0329602
0.3841	0.3468	0.0271	0.0477	7.19E-15	0.6838
0.0668	0.02073	0.05263	0.1623	1.45E-33	0.896
0.0481	0.0771	0.05954	0.0864	1.49E-29	0.2487
0.4689	0.5391	0.01969	0.0454	0.00E+00	0.7204
0.0409	0.006844	0.06291	0.2828	1.06E-36	0.1982
0.0044	0.07472	0.1835	0.0865	1.35E-20	0.9393
0.0042	0.001315	0.1905	0.6881	6.98E-12	0.8408
0.0317	0.01393	0.0719	0.1959	4.24E-22	0.3054
0.2866	0.1697	0.02787	0.061	7.94E-09	0.2074
0.2975	0.1244	0.02719	0.0686	1.83E-13	0.7019

0.3595	0.3468	0.02651	0.0477	1.55E-13	0.6838
0.4069	0.5273	0.02536	0.0456	3.59E-18	0.7571
0.0666	0.02073	0.05013	0.1623	1.86E-41	0.896
0.0295	0.03282	0.07402	0.127	1.95E-13	0.9459
0.0095	0.02011	0.1289	0.1683	1.29E-09	0.4447
0.0464	0.04164	0.05993	0.1154	2.30E-10	0.786901
0.4267	0.4947	0.02078	0.0455	4.32E-294	0.6534
0.0464	0.003478	0.05819	0.4035	4.44E-25	0.3465
0.0408	0.006844	0.06357	0.2828	3.06E-33	0.1982
0.0044	0.07472	0.1845	0.0865	4.41E-18	0.9393
0.0043	0.001315	0.1915	0.6881	6.05E-10	0.8408
0.2875	0.1697	0.02815	0.061	2.88E-08	0.2074
0.2972	0.1244	0.02749	0.0686	4.17E-12	0.7019
0.0263	0.01428	0.0789	0.1929	1.07E-15	0.0241502
0.021	0.07624	0.08658	0.0853	1.10E-09	0.346
0.0095	0.02011	0.1294	0.1683	8.93E-09	0.4447
0.0464	0.04164	0.06066	0.1154	3.22E-09	0.786901
0.0554	0.01471	0.05595	0.1904	3.75E-37	0.4917
0.0675	0.01732	0.04951	0.1799	3.04E-18	0.825
0.046	0.003478	0.05899	0.4035	1.03E-22	0.3465
0.041	0.006844	0.06467	0.2828	7.28E-32	0.1982
0.0317	0.01393	0.07403	0.1959	7.75E-21	0.3054
0.0462	0.003723	0.06103	0.3907	5.07E-40	0.0329602
0.0667	0.02073	0.05184	0.1623	2.59E-34	0.896
0.383	0.3462	0.02662	0.0477	1.73E-16	0.6829
0.5314	0.4608	0.01941	0.0454	0.00E+00	0.728201
0.0483	0.0771	0.05887	0.0864	7.75E-26	0.2487
0.479	0.4762	0.02746	0.0458	6.89E-13	0.9969
0.4574	0.4372	0.03174	0.0487	3.90E-10	0.0900907
0.5296	0.593	0.02879	0.0464	4.81E-08	0.0564196
0.4856	0.646	0.02715	0.0477	1.29E-08	0.7732
0.0772	0.02778	0.06799	0.1405	2.04E-15	0.5392
0.0566	0.05421	0.07845	0.1021	6.30E-10	0.8432
0.0733	0.004544	0.07238	0.348	1.33E-12	0.5298
0.1939	0.3718	0.03861	0.0471	1.81E-150	0.1134
0.2579	0.1541	0.04217	0.0635	1.40E-17	0.7535
0.0707	0.03757	0.07051	0.1224	4.53E-12	0.592
0.2006	0.05323	0.04576	0.1041	1.26E-13	0.2159
0.0772	0.02778	0.06836	0.1405	1.06E-13	0.5392
0.0734	0.004544	0.07256	0.348	5.21E-12	0.5298
0.1934	0.3718	0.0393	0.0471	6.08E-143	0.1134
0.258	0.1541	0.0424	0.0635	5.07E-15	0.7535
0.0705	0.03757	0.07057	0.1224	1.64E-13	0.592
0.2008	0.05323	0.0459	0.1041	3.07E-13	0.2159
0.0772	0.02778	0.06945	0.1405	1.38E-08	0.5392
0.5441	0.3944	0.03593	0.0466	1.22E-50	0.2997
0.045	0.01191	0.06602	0.2128	3.25E-08	0.7383
0.2041	0.4347	0.03404	0.0458	1.40E-08	0.0498597
0.4459	0.2595	0.02534	0.0523	6.62E-120	0.6974
0.051	0.1128	0.06189	0.0732	9.11E-15	0.5532
0.7893	0.7156	0.03327	0.0507	8.64E-09	0.9643
0.106	0.1082	0.04432	0.0735	8.58E-13	0.8692
0.0296	0.01287	0.08145	0.2144	1.00E-09	0.1904
0.1993	0.1823	0.03163	0.0594	2.97E-08	0.490601
0.0965	0.04117	0.0417	0.1166	2.09E-10	0.1437
0.2425	0.3066	0.02843	0.0498	9.01E-29	0.2701
0.0972	0.027	0.042	0.1409	1.75E-10	0.795
0.8744	0.8829	0.03711	0.0712	3.14E-08	0.723901

0.7067	0.6684	0.0304	0.058	6.09E-10	0.0760099
0.184	0.009685	0.03691	0.2391	1.47E-10	0.0986211
0.1491	0.1124	0.03869	0.0736	2.90E-08	0.7626
0.037	0.002494	0.07314	0.4732	2.54E-16	0.0324198
0.0457	0.003723	0.06633	0.3907	3.04E-22	0.0329602
0.2147	0.08465	0.03345	0.0811	7.71E-19	0.8016
0.4729	0.5396	0.02414	0.0454	7.97E-170	0.747901
0.0557	0.01471	0.06227	0.1904	1.39E-14	0.4917
0.0193	0.06736	0.09207	0.0897	2.65E-08	0.00850609
0.1008	0.01715	0.04232	0.1748	3.32E-08	0.2573
0.0193	0.06736	0.08644	0.0897	4.72E-08	0.00850609
0.0314	0.01471	0.07064	0.2013	1.85E-08	0.4496
0.1985	0.1915	0.03027	0.0589	5.45E-09	0.3524
0.6739	0.4166	0.02556	0.0463	2.84E-09	0.7959
0.2111	0.02089	0.03814	0.1622	4.36E-15	0.5184
0.0359	0.01936	0.07188	0.1747	4.01E-08	0.649201
0.0332	0.004679	0.07489	0.3486	1.06E-08	0.3781
0.3234	0.2363	0.02982	0.0538	6.18E-09	0.2904
0.0817	0.03364	0.0561	0.1271	4.73E-12	0.8217
0.8124	0.786	0.03416	0.0558	2.47E-43	0.0562095
0.0729	0.07649	0.05178	0.0849	1.06E-48	0.658801
0.635	0.5768	0.03189	0.0531	3.06E-65	0.00432404
0.4355	0.245	0.03232	0.0538	6.87E-36	0.433
0.4355	0.2252	0.03232	0.0681	6.87E-36	0.8549
0.2107	0.02089	0.03665	0.1622	3.74E-09	0.5184
0.1886	0.1867	0.03241	0.0595	4.75E-09	0.2692
0.3055	0.4841	0.02873	0.0457	7.05E-21	0.2911
0.0276	0.04052	0.08226	0.1175	3.03E-15	0.8954
0.1066	0.01176	0.04098	0.2125	9.39E-71	0.4589
0.0385	0.02382	0.07013	0.1566	1.49E-10	0.8106
0.1069	0.006002	0.0461	0.307	1.75E-08	0.0186999
0.0982	0.04235	0.04163	0.1145	1.45E-09	0.1598
0.8737	0.8895	0.03694	0.0736	7.25E-10	0.6296
0.2323	0.3066	0.02912	0.0498	2.09E-16	0.2708
0.0201	0.003981	0.089	0.3649	3.65E-10	0.467599
0.0185	0.02561	0.09163	0.143	1.22E-09	0.9616
0.0197	0.01122	0.0892	0.2173	1.34E-08	0.4799
0.0201	0.003981	0.08896	0.3649	1.70E-09	0.467599
0.611	0.6666	0.02538	0.0484	5.48E-11	0.696299
0.2101	0.03411	0.03505	0.1254	1.70E-08	0.0232001
0.052	0.009082	0.05776	0.248	5.51E-10	0.8013
0.2594	0.1007	0.03383	0.0822	6.87E-13	0.5689
0.4558	0.49	0.03013	0.0487	4.43E-87	0.8913
0.3368	0.05968	0.03249	0.0989	1.20E-11	0.7345
0.4581	0.3476	0.02632	0.048	7.66E-09	0.1792
0.2774	0.2623	0.03006	0.0517	3.61E-15	0.7704
0.0044	0.07472	0.1855	0.0865	3.35E-17	0.9393
0.2975	0.1244	0.02752	0.0686	1.21E-08	0.7019
0.0035	0.01555	0.2272	0.1806	6.75E-12	0.3913
0.0006	0.00676	0.4989	0.2846	5.13E-09	0.1851
0.0006	0.01928	0.4776	0.1711	2.68E-08	0.8553
0.0543	0.04058	0.05516	0.1145	1.38E-13	0.2585
0.1525	0.1219	0.03616	0.07	3.87E-08	0.3944
0.1281	0.01649	0.04219	0.187	1.04E-08	0.3482
0.199	0.1145	0.03315	0.0721	5.13E-67	0.8412
0.0003	0.03565	0.5986	0.1254	2.76E-08	0.4795
0.0044	0.07472	0.1662	0.0865	1.77E-12	0.9393
0.0043	0.001315	0.1715	0.6881	3.23E-09	0.8408

0	0.08777	1.461	0.0803	2.27E-08	0.9233
0.2974	0.1244	0.02442	0.0686	2.82E-11	0.7019
0.2664	0.3528	0.02509	0.0479	2.72E-08	0.9799
0.0003	0.01303	0.5986	0.2053	2.58E-08	0.462701
0.0758	0.1943	0.04896	0.0592	3.75E-25	0.0748497
0.0133	0.05822	0.1098	0.0989	2.16E-14	0.271
0.0478	0.06942	0.05863	0.091	1.09E-08	0.2111
0.1327	0.0614	0.03562	0.0966	8.90E-99	0.3182
0.3259	0.5036	0.02588	0.0456	1.45E-59	0.5836
0.0451	0.0161	0.05895	0.1861	4.30E-24	0.2973
0.0163	0.03912	0.1185	0.121	2.13E-09	0.9501
0.011	0.004242	0.1167	0.3916	4.21E-08	0.5566
0.0758	0.1943	0.04948	0.0592	3.16E-19	0.0748497
0.0133	0.05822	0.1104	0.0989	1.15E-13	0.271
0.0478	0.06942	0.05896	0.091	4.73E-08	0.2111
0.1327	0.0614	0.03619	0.0966	2.76E-83	0.3182
0.3259	0.5036	0.02617	0.0456	8.17E-50	0.5836
0.0451	0.0161	0.05946	0.1861	1.25E-20	0.2973
0.011	0.004242	0.1176	0.3916	3.65E-08	0.5566
0.0044	0.01061	0.186	0.224	1.82E-10	0.682501
0.0044	0.01061	0.1846	0.224	8.02E-09	0.682501
0.1868	0.009171	0.05429	0.2401	4.35E-10	0.8793
0.2308	0.1143	0.03334	0.073	1.68E-09	0.5898
0.0266	0.002263	0.08405	0.497	2.89E-08	0.778501
0.0841	0.04891	0.0505	0.1071	6.15E-12	0.953
0.9244	0.9214	0.05405	0.0842	2.39E-12	0.6517
0.0425	0.0156	0.07152	0.1803	1.27E-08	0.1034
0.1087	0.0008819	0.04734	0.8254	2.55E-11	0.6536
0.1503	0.1363	0.03884	0.1009	2.00E-08	0.0905503
0.2969	0.1244	0.02548	0.0686	1.18E-08	0.7019
0.1087	0.0008819	0.04769	0.8254	1.37E-10	0.6536
0.1078	0.000882	0.04937	0.8253	1.91E-09	0.653501
0.1687	0.1811	0.03256	0.0605	3.91E-08	0.6385
0.0032	0.004554	0.2117	0.3402	7.49E-09	0.845
0.0032	0.004554	0.2277	0.3402	5.88E-10	0.845
0.0034	0.01555	0.2345	0.1806	4.23E-08	0.3913
0.0449	0.05731	0.05835	0.0997	2.84E-09	0.664401
0.0312	0.01769	0.07114	0.1832	4.24E-11	0.3447
0.1923	0.1895	0.03063	0.059	5.06E-11	0.3304
0.0998	0.08693	0.0399	0.0811	1.03E-25	0.4555
0.0185	0.02561	0.09244	0.143	2.55E-13	0.9616
0.0197	0.01122	0.09002	0.2173	1.07E-11	0.4799
0.0201	0.003981	0.08983	0.3649	5.43E-13	0.467599
0.0185	0.02561	0.09165	0.143	2.91E-08	0.9616
0.0201	0.003981	0.08901	0.3649	5.79E-09	0.467599
0.0185	0.02561	0.09251	0.143	2.61E-10	0.9616
0.0197	0.01122	0.09004	0.2173	2.36E-09	0.4799
0.0201	0.00403	0.0907	0.3625	8.34E-10	0.4934
0.0215	0.02561	0.116	0.143	7.39E-10	0.9616
0.0224	0.01122	0.1158	0.2173	1.65E-08	0.4799
0.0205	0.00403	0.1195	0.3625	9.18E-11	0.4934
0.0185	0.02561	0.09132	0.143	6.42E-09	0.9616
0.0201	0.003982	0.08866	0.3651	3.80E-09	0.4674
0.0609	0.01743	0.05096	0.1765	3.76E-08	0.984
0.0185	0.02561	0.0918	0.143	4.29E-09	0.9616
0.0197	0.01122	0.08935	0.2173	3.95E-08	0.4799
0.0201	0.00403	0.08992	0.3625	2.02E-10	0.4934
0.0185	0.02561	0.09212	0.143	1.92E-09	0.9616

0.6109	0.6666	0.02557	0.0484	7.82E-09	0.696299
0.0201	0.00403	0.09026	0.3625	2.31E-10	0.4934
0.0307	0.1024	0.09656	0.0748	2.92E-08	0.6712
0.5197	0.3884	0.03266	0.0468	1.86E-14	0.3663
0.0963	0.004735	0.06556	0.3345	2.51E-08	0.2435
0.1202	0.1518	0.06137	0.0641	2.44E-60	0.0195398
0.2911	0.07462	0.04468	0.0877	2.71E-23	0.5155
0.0003	0.01669	8.721	0.1884	6.98E-10	0.1668
0.2185	0.2684	0.4129	0.0558	7.93E-10	0.1617
0.0009	0.02269	5.206	0.1559	3.59E-08	0.89
0.2372	0.0221	0.473	0.1537	1.03E-08	0.8055
0.6474	0.5857	0.3672	0.0492	1.26E-08	0.00383301
0.001	0.01784	4.629	0.1753	1.42E-08	0.2602
0.541	0.4431	0.3071	0.046	3.08E-08	0.2845
0.0022	0.03683	3.073	0.1233	2.16E-09	0.0787608
0.0029	0.004329	2.692	0.3665	3.49E-08	0.4661
0.0016	0.04362	0.4204	0.1118	9.84E-09	0.02828
0.2132	0.3626	0.03861	0.0472	7.51E-35	0.0579002
0.0673	0.1486	0.06722	0.0647	9.46E-14	0.504001
0.0016	0.04362	0.4216	0.1118	9.41E-09	0.02828
0.0673	0.1486	0.06739	0.0647	5.44E-14	0.504001
0.0173	0.01706	0.105	0.1857	1.29E-10	0.3603
0.4466	0.26	0.02529	0.0523	4.33E-119	0.5358
0.0512	0.1128	0.062	0.0732	1.33E-08	0.5532
0.0173	0.01706	0.1049	0.1857	8.19E-10	0.3603
0.1482	0.2489	0.0385	0.0529	2.28E-13	0.1369
0.4466	0.26	0.02507	0.0523	3.46E-127	0.5358
0.0324	0.05182	0.07691	0.1042	1.48E-09	0.945
0.0173	0.01706	0.1046	0.1857	3.99E-09	0.3603
0.1482	0.2489	0.0384	0.0529	3.83E-13	0.1369
0.4466	0.26	0.02511	0.0523	7.26E-122	0.5358
0.0324	0.05182	0.07664	0.1042	1.50E-10	0.945
0.0173	0.01706	0.1039	0.1857	1.46E-10	0.3603
0.4466	0.26	0.02484	0.0523	6.96E-128	0.5358
0.0324	0.05182	0.07619	0.1042	2.49E-10	0.945
0.2996	0.05773	0.02793	0.098	1.48E-08	0.777499
0.7732	0.6698	0.02866	0.0502	1.22E-09	0.5961
0.0115	0.02821	0.1213	0.1359	1.71E-13	0.4767
0.0289	0.01519	0.07316	0.1911	1.54E-10	0.1145
0.0194	0.01518	0.08937	0.1876	3.63E-32	0.3459
0.0322	0.02741	0.06983	0.1394	7.84E-13	0.8787
0.0115	0.02821	0.1209	0.1359	7.97E-09	0.4767
0.0194	0.01518	0.08987	0.1876	4.58E-18	0.3459
0.0115	0.02821	0.1184	0.1359	1.69E-14	0.4767
0.0321	0.02741	0.06859	0.1394	9.69E-15	0.8758
0.233	0.2522	0.02931	0.0524	1.35E-08	0.8767
0.0288	0.01519	0.07246	0.1911	5.24E-10	0.1145
0.0194	0.01518	0.08813	0.1876	6.27E-32	0.3459
0.0115	0.02821	0.1197	0.1359	5.48E-11	0.4767
0.0194	0.01518	0.08908	0.1876	1.76E-22	0.3459
0.0321	0.02741	0.06918	0.1394	1.66E-09	0.8787
0.0115	0.02821	0.1174	0.1359	3.34E-14	0.4767
0.0194	0.01518	0.08708	0.1876	5.62E-26	0.3459
0.0321	0.02741	0.06776	0.1394	9.22E-12	0.8787
0.1689	0.06072	0.03268	0.0961	4.42E-15	0.718199
0.0401	0.01869	0.06205	0.1702	2.29E-08	0.5433
0.1714	0.06072	0.03176	0.0961	4.06E-63	0.718199
0.081	0.01444	0.045	0.1993	1.45E-08	0.9522

0.1714	0.06072	0.03231	0.0961	3.10E-45	0.718199
0.1699	0.06072	0.0332	0.0961	1.81E-18	0.718199
0.1328	0.0614	0.03746	0.0966	2.36E-14	0.3182
0.1714	0.06072	0.03171	0.0961	3.21E-65	0.718199
0.081	0.01444	0.04499	0.1993	1.77E-08	0.9522
0.1714	0.06072	0.03195	0.0961	2.52E-43	0.718199
0.2136	0.2246	0.02973	0.0543	5.41E-09	0.3116
0.6125	0.5608	0.02533	0.0463	3.56E-08	0.673
0.0545	0.02605	0.05455	0.1451	3.73E-13	0.3565
0.0275	0.04052	0.07599	0.1175	6.79E-22	0.8954
0.1067	0.01176	0.03954	0.2125	2.06E-16	0.4589
0.0378	0.02382	0.06505	0.1566	5.81E-20	0.8106
0.6125	0.5608	0.02521	0.0463	2.55E-08	0.673
0.0545	0.02605	0.05433	0.1451	1.35E-12	0.3565
0.0275	0.04052	0.07572	0.1175	1.51E-20	0.8954
0.1067	0.01176	0.03937	0.2125	5.38E-16	0.4589
0.0378	0.02382	0.06479	0.1566	2.47E-19	0.8106
0.5684	0.3828	0.02463	0.047	5.79E-09	0.753101
0.1287	0.04786	0.03676	0.1074	1.47E-10	0.7335
0.1073	0.03644	0.04036	0.1185	5.23E-20	0.1517
0.0545	0.02605	0.05416	0.1451	4.64E-11	0.3565
0.0275	0.04052	0.07557	0.1175	6.50E-19	0.8954
0.1915	0.1895	0.03135	0.0591	2.88E-12	0.3337
0.1067	0.01176	0.03905	0.2125	7.85E-22	0.4589
0.0378	0.02382	0.06465	0.1566	2.39E-17	0.8106
0.0545	0.02605	0.05436	0.1451	1.25E-11	0.3565
0.0275	0.04052	0.07581	0.1175	2.76E-20	0.8954
0.1915	0.1895	0.03147	0.0591	6.36E-13	0.3337
0.1067	0.01176	0.0392	0.2125	2.68E-22	0.4589
0.0378	0.02382	0.06488	0.1566	5.29E-18	0.8106
0.0545	0.02605	0.05547	0.1451	2.25E-08	0.3565
0.0275	0.04052	0.07776	0.1175	3.23E-13	0.8954
0.1634	0.2353	0.03387	0.0532	3.82E-08	0.0392998
0.1067	0.01176	0.03983	0.2125	8.64E-26	0.4589
0.5112	0.4	0.0251	0.0465	3.34E-09	0.23
0.0377	0.02382	0.06656	0.1566	1.79E-09	0.8106
0.5271	0.3535	0.02505	0.0478	9.10E-09	0.9214
0.0548	0.02605	0.05379	0.1451	4.64E-09	0.3565
0.0277	0.04052	0.07504	0.1175	9.75E-17	0.8954
0.1094	0.01183	0.0383	0.2117	9.86E-22	0.453
0.0379	0.02382	0.06419	0.1566	1.41E-15	0.8106
0.0545	0.02605	0.05512	0.1451	3.91E-09	0.3565
0.0275	0.04052	0.07722	0.1175	5.37E-15	0.8954
0.1067	0.01176	0.03955	0.2125	1.04E-27	0.4589
0.5112	0.4	0.02495	0.0465	3.25E-09	0.23
0.0377	0.02382	0.06614	0.1566	2.83E-10	0.8106
0.0548	0.02605	0.05419	0.1451	1.40E-10	0.3565
0.0277	0.04052	0.0755	0.1175	1.38E-18	0.8954
0.1069	0.01176	0.03929	0.2125	2.69E-16	0.4589
0.0379	0.02382	0.0646	0.1566	1.16E-17	0.8106
0.5685	0.3828	0.02455	0.047	2.20E-09	0.753101
0.2995	0.2507	0.0284	0.0524	1.82E-25	0.7486
0.0845	0.04891	0.04423	0.1071	3.30E-114	0.953
0.1968	0.05865	0.03302	0.098	4.94E-12	0.8625
0.0625	0.01496	0.05457	0.1875	4.48E-11	0.2532
0.0381	0.03779	0.06789	0.1177	3.80E-08	0.41
0.2084	0.07845	0.03331	0.0842	8.20E-15	0.0290898
0.2447	0.1086	0.02792	0.0736	4.21E-165	0.329

	0.0688	0.02845	0.0521	0.1391	4.90E-12	0.0954289
	0.3262	0.544	0.02839	0.0455	4.02E-13	0.325
	0.2223	0.2205	0.03434	0.0605	5.88E-12	0.2408
	0.7752	0.7576	0.0354	0.0538	2.23E-13	0.2915
	0.0226	0.01011	0.08487	0.2395	1.47E-08	0.0930894
	0.0044	0.07472	0.1865	0.0865	4.49E-18	0.9393
	0.2975	0.1244	0.02763	0.0686	5.43E-11	0.7019
	0.0035	0.01555	0.2286	0.1806	4.11E-11	0.3913
	0.0006	0.00676	0.5018	0.2846	4.97E-09	0.1851
	0.1525	0.1219	0.03637	0.07	4.14E-08	0.3944
	0.1562	0.1008	0.03509	0.0745	1.37E-15	0.0837202
	0.0044	0.07472	0.1739	0.0865	3.57E-18	0.9393
	0.0035	0.01555	0.2131	0.1806	1.72E-11	0.3913
	0.8142	0.9725	0.03044	0.1435	4.22E-18	0.450001
	0.4709	0.5257	0.0232	0.0454	4.78E-32	0.293
	0.0045	0.07472	0.202	0.0865	4.31E-09	0.9393
	0.3455	0.4221	0.02871	0.0463	1.09E-13	0.8607
	0.0044	0.07472	0.1774	0.0865	2.93E-23	0.9393
	0.0042	0.001315	0.1841	0.6881	1.78E-14	0.8408
	0.1084	0.08133	0.03894	0.0837	1.44E-08	0.9954
	0.4709	0.5257	0.02374	0.0454	3.53E-33	0.293
	0.0044	0.07472	0.188	0.0865	3.55E-11	0.9393
	0.7833	0.7575	0.03476	0.0538	1.47E-11	0.2934
	0.0044	0.07472	0.1857	0.0865	6.36E-11	0.9393
	0.0044	0.07472	0.1853	0.0865	2.66E-10	0.9393
	0.8142	0.9725	0.03216	0.1435	5.06E-12	0.450001
	0.4709	0.5257	0.02476	0.0454	5.19E-17	0.293
	0.0044	0.07472	0.1933	0.0865	1.18E-09	0.9393
	0.0034	0.01555	0.2369	0.1806	1.67E-10	0.3913
	0.0542	0.04058	0.05733	0.1145	7.93E-09	0.2585
	0.0044	0.07472	0.1663	0.0865	1.87E-27	0.9393
	0.0035	0.01555	0.2042	0.1806	3.70E-18	0.3913
	0.1084	0.08133	0.03673	0.0837	1.70E-08	0.9954
	0.0047	0.0143	0.1693	0.188	4.52E-09	0.3362
	0.0095	0.02011	0.1174	0.1683	2.55E-08	0.4447
	0.4709	0.5257	0.02227	0.0454	6.29E-38	0.293
	0.9748	0.9604	0.07373	0.1166	4.50E-10	0.9826
	0.0044	0.07472	0.1783	0.0865	5.33E-24	0.9393
	0.0042	0.001315	0.1851	0.6881	1.62E-14	0.8408
	0.1084	0.08133	0.03915	0.0837	2.77E-08	0.9954
	0.4709	0.5257	0.02387	0.0454	5.49E-33	0.293
	0.0045	0.07472	0.191	0.0865	3.79E-13	0.9393
	0.0036	0.01555	0.2281	0.1806	6.19E-09	0.3913
	0.0375	0.00757	0.07055	0.2738	9.98E-24	0.1051
	0.0246	0.01433	0.08426	0.1959	6.32E-13	0.1007
	0.1871	0.05718	0.03241	0.097	5.75E-51	0.0911591
	0.0643	0.0558	0.05318	0.0984	1.19E-09	0.6042
	0.0689	0.005976	0.0526	0.3009	1.59E-09	0.9932
	0.2548	0.184	0.0299	0.0587	4.26E-09	0.2637
	0.0044	0.07472	0.1678	0.0865	3.37E-28	0.9393
	0.0035	0.01555	0.2061	0.1806	4.93E-18	0.3913
	0.1084	0.08133	0.03723	0.0837	2.14E-08	0.9954
	0.0047	0.0143	0.1707	0.188	1.00E-08	0.3362
	0.0212	0.008734	0.07742	0.2645	3.08E-08	0.3686
	0.4709	0.5257	0.02252	0.0454	3.40E-38	0.293
	0.9748	0.9604	0.07456	0.1166	5.96E-10	0.9826
	0.0044	0.07472	0.1911	0.0865	1.83E-08	0.9393
	0.0543	0.04058	0.05532	0.1145	5.32E-25	0.2585

0.0044	0.07472	0.169	0.0865	3.18E-11	0.9393
0.0034	0.01555	0.2078	0.1806	3.74E-09	0.3913
0.0044	0.07472	0.1934	0.0865	5.94E-10	0.9393
0.0034	0.01555	0.2371	0.1806	9.40E-10	0.3913
0.0542	0.04058	0.05734	0.1145	2.27E-09	0.2585
0.0044	0.07472	0.1771	0.0865	1.42E-11	0.9393
0.0049	0.03129	0.1747	0.132	1.45E-08	0.541
0.0044	0.07472	0.1744	0.0865	1.02E-13	0.9393
0.0042	0.001315	0.1805	0.6881	1.30E-10	0.8408
0.2977	0.1244	0.02569	0.0686	2.27E-11	0.7019
0.243	0.2842	0.02712	0.0506	2.21E-10	0.6178
0.0731	0.02004	0.04503	0.1656	3.34E-09	0.1499
0.0044	0.07472	0.1905	0.0865	5.53E-11	0.9393
0.0032	0.004446	0.2611	0.3457	8.39E-20	0.5019
0.0044	0.07472	0.1858	0.0865	2.37E-12	0.9393
0.0042	0.001315	0.1918	0.6881	5.92E-10	0.8408
0.2977	0.1244	0.02726	0.0686	3.80E-09	0.7019
0.2464	0.4034	0.02881	0.0467	2.85E-09	0.5837
0.2076	0.07845	0.03216	0.0842	1.32E-15	0.0290898
0.4331	0.5038	0.02619	0.0456	4.42E-08	0.4114
0.2474	0.1083	0.02772	0.0738	1.30E-124	0.3928
0.2162	0.2237	0.03019	0.0544	8.74E-17	0.2778
0.5004	0.5374	0.02509	0.0456	4.97E-08	0.7701
0.5698	0.3828	0.02506	0.047	6.72E-09	0.753101
0.2111	0.07845	0.03117	0.0842	1.81E-22	0.0290898
0.0405	0.05056	0.06354	0.1048	3.72E-08	0.7755
0.0299	0.004659	0.07878	0.3483	2.60E-11	0.6312
0.2456	0.1086	0.02593	0.0736	2.60E-208	0.329
0.0702	0.02845	0.0488	0.1391	1.83E-14	0.0954289
0.2099	0.07845	0.03359	0.0842	2.85E-13	0.0290898
0.4305	0.5038	0.02756	0.0456	4.62E-10	0.4114
0.2402	0.1086	0.0301	0.0736	1.98E-74	0.329
0.2083	0.07845	0.03229	0.0842	7.20E-17	0.0290898
0.0096	0.003346	0.1293	0.428	5.90E-09	0.1724
0.2449	0.1086	0.02735	0.0736	1.40E-150	0.329
0.0686	0.02845	0.05081	0.1391	2.05E-12	0.0954289
0.2084	0.07845	0.03202	0.0842	2.99E-10	0.0290898
0.2447	0.1083	0.02815	0.0738	1.63E-91	0.3928
0.2645	0.3008	0.02885	0.0494	2.25E-10	0.261
0.2074	0.07845	0.03144	0.0842	8.32E-17	0.0290898
0.2473	0.1083	0.02689	0.0738	2.05E-136	0.3928
0.0686	0.02845	0.04947	0.1391	1.15E-09	0.0954289
0.2083	0.07845	0.03328	0.0842	2.47E-20	0.0290898
0.0096	0.003346	0.1306	0.428	3.62E-09	0.1724
0.2449	0.1086	0.02691	0.0736	1.34E-220	0.329
0.0686	0.02845	0.05195	0.1391	3.96E-16	0.0954289
0.2076	0.07845	0.0315	0.0842	1.62E-17	0.0290898
0.4331	0.5038	0.02565	0.0456	2.47E-09	0.4114
0.2474	0.1086	0.02686	0.0736	9.97E-142	0.329
0.2084	0.07845	0.03284	0.0842	5.25E-10	0.0290898
0.2447	0.1086	0.0287	0.0736	8.46E-99	0.329
0.2083	0.07845	0.03328	0.0842	1.29E-15	0.0290898
0.0096	0.003346	0.1312	0.428	8.13E-09	0.1724
0.2449	0.1086	0.02785	0.0736	9.71E-166	0.329
0.0686	0.02845	0.05205	0.1391	2.26E-12	0.0954289
0.2083	0.07845	0.03326	0.0842	2.43E-17	0.0290898
0.0094	0.003214	0.1303	0.4327	3.34E-10	0.1325
0.0438	0.007744	0.06661	0.2603	1.36E-08	0.7437

	0.2449	0.1086	0.02787	0.0736	9.85E-166	0.329
	0.0686	0.02845	0.05202	0.1391	1.66E-14	0.0954289
	0.2084	0.07845	0.03316	0.0842	3.19E-16	0.0290898
	0.8488	0.8314	0.03711	0.0605	2.54E-08	0.2359
	0.2447	0.1083	0.02825	0.0738	4.02E-144	0.3928
	0.0688	0.02845	0.05191	0.1391	3.87E-12	0.0954289
	0.2084	0.07845	0.03272	0.0842	6.30E-18	0.0290898
	0.0437	0.007744	0.06549	0.2603	1.43E-09	0.7437
	0.0095	0.003346	0.1303	0.428	8.71E-10	0.1724
	0.2447	0.1086	0.02725	0.0736	8.53E-178	0.329
	0.0688	0.02845	0.05126	0.1391	5.86E-16	0.0954289
	0.2109	0.07845	0.03087	0.0842	1.84E-31	0.0290898
	0.237	0.107	0.02248	0.0741	0.00E+00	0.4584
	0.1482	0.2908	0.03508	0.0504	9.59E-11	0.0579696
	0.5946	0.5797	0.02568	0.0464	1.19E-15	0.2121
	0.0247	0.04112	0.08113	0.1174	4.60E-10	0.4684
	0.0272	0.02289	0.0778	0.154	3.22E-09	0.704399
	0.2084	0.07845	0.03321	0.0842	4.18E-20	0.0290898
	0.2447	0.1086	0.0268	0.0736	1.03E-225	0.329
	0.0688	0.02845	0.05172	0.1391	3.16E-16	0.0954289
	0.1104	0.07198	0.04179	0.09	3.09E-08	0.1661
	0.2099	0.07845	0.03389	0.0842	2.03E-12	0.0290898
	0.4305	0.5038	0.02782	0.0456	2.70E-08	0.4114
	0.2402	0.1086	0.03044	0.0736	3.58E-69	0.329
	0.2083	0.07845	0.03229	0.0842	2.75E-12	0.0290898
	0.4349	0.5038	0.02614	0.0456	5.43E-09	0.4114
	0.2449	0.1086	0.02797	0.0736	2.29E-112	0.329
	0.0086	0.03279	0.1747	0.1261	2.30E-18	0.0284997
	0.0081	0.009645	0.1814	0.2335	2.00E-15	0.735201
	0.5587	0.3298	0.02453	0.0484	1.20E-10	0.2568
	0.2454	0.1955	0.02745	0.0576	7.76E-48	0.9394
	0.7759	0.7776	0.02895	0.0549	8.76E-09	0.9043
	0.5587	0.3298	0.02465	0.0484	6.90E-10	0.2568
	0.2454	0.1955	0.0276	0.0576	1.71E-45	0.9394
	0.5587	0.3298	0.02445	0.0484	6.79E-13	0.2568
	0.2454	0.1955	0.0273	0.0576	8.06E-53	0.9394
	0.7759	0.7776	0.0289	0.0549	3.75E-08	0.9043
	0.5587	0.3298	0.02448	0.0484	6.09E-13	0.2568
	0.2454	0.1955	0.02736	0.0576	1.16E-51	0.9394
	0.7759	0.7776	0.02894	0.0549	2.05E-08	0.9043
	0.0681	0.0008004	0.07716	0.8367	1.75E-17	0.1875
	0.5722	0.5164	0.03915	0.0518	2.14E-49	0.7501
	0.0686	0.0008004	0.06113	0.8367	7.61E-34	0.1875
	0.1398	0.2113	0.03898	0.0558	1.10E-13	0.6975
	0.1173	0.1517	0.0446	0.0641	6.25E-146	0.01842
	0.1367	0.288	0.04076	0.0505	1.28E-08	0.8473
	0.5014	0.5015	0.03041	0.052	8.65E-22	0.3526
	0.0686	0.0008004	0.06015	0.8367	3.14E-39	0.1875
	0.5362	0.4992	0.0308	0.0522	1.13E-24	0.4996
	0.1179	0.1517	0.04857	0.0641	1.39E-65	0.0200198
	0.2371	0.1065	0.03135	0.074	5.94E-51	0.5083
	0.1238	0.2745	0.04331	0.0512	1.95E-11	0.5162
	0.0686	0.0008004	0.06047	0.8367	6.52E-42	0.1875
	0.1174	0.1517	0.04613	0.0641	3.67E-110	0.01842
	0.1553	0.3901	0.04073	0.0506	3.42E-15	0.4372
	0.2372	0.1065	0.03192	0.074	2.91E-26	0.5083
	0.1365	0.288	0.04133	0.0505	1.44E-10	0.8473
	0.5014	0.5015	0.03045	0.052	3.03E-25	0.3526

0.0005	0.004456	0.5674	0.3549	3.93E-08	0.02009
0.345	0.4221	0.02589	0.0463	4.05E-08	0.8607
0.2464	0.2876	0.03112	0.0507	1.21E-24	0.9298
0.6535	0.6046	0.03175	0.0473	3.73E-12	0.767201
0.2464	0.2876	0.03145	0.0507	1.12E-21	0.9298
0.2466	0.2876	0.03171	0.0507	1.71E-10	0.9298
0.4572	0.4372	0.03065	0.0487	9.04E-10	0.0900907
0.2466	0.2876	0.03153	0.0507	1.00E-10	0.9298
0.2467	0.2876	0.03074	0.0507	2.11E-09	0.9298
0.2466	0.2876	0.03133	0.0507	5.49E-11	0.9298
0.4719	0.3699	0.02974	0.0485	1.42E-13	0.6635
0.4865	0.2828	0.02967	0.061	9.61E-12	0.5346
0.2466	0.2876	0.03133	0.0507	1.66E-14	0.9298
0.2465	0.2876	0.03137	0.0507	6.46E-25	0.9298
0.2466	0.2876	0.03131	0.0507	3.36E-15	0.9298
0.2466	0.2876	0.03149	0.0507	7.48E-14	0.9298
0.7744	0.7902	0.02511	0.0566	4.52E-32	0.1036
0.0077	0.06232	0.1494	0.0954	1.37E-08	0.649999
0.611	0.6666	0.02551	0.0484	7.89E-09	0.696299
0.0201	0.003981	0.08936	0.365	1.12E-08	0.4678
0.6109	0.6666	0.02548	0.0484	1.46E-09	0.696299
0.0201	0.003981	0.08918	0.365	9.28E-10	0.4678
0.0041	0.009268	0.201	0.2416	1.29E-08	0.707801
0.4847	0.5676	0.02672	0.0463	9.13E-13	0.5962
0.592	0.5984	0.02451	0.0464	3.18E-08	0.9317
0.3256	0.03521	0.0262	0.1253	3.31E-17	0.6524
0.592	0.5984	0.02489	0.0464	2.30E-09	0.9317
0.5715	0.4562	0.02517	0.0457	1.21E-08	0.3228
0.5044	0.2247	0.02479	0.0549	2.91E-10	0.1255
0.475	0.4026	0.02416	0.0467	3.70E-09	0.4826
0.3256	0.03521	0.02632	0.1253	1.09E-18	0.6524
0.4882	0.382	0.0236	0.0474	5.53E-10	0.7097
0.3257	0.03521	0.02599	0.1253	3.43E-13	0.6524
0.4903	0.4009	0.02397	0.0466	1.81E-08	0.6265
0.3256	0.03521	0.02614	0.1253	8.09E-16	0.6524
0.02	0.0162	0.08768	0.1796	8.75E-09	0.8315
0.0711	0.0008447	0.05515	0.8059	4.66E-09	0.1911
0.0888	0.004023	0.04821	0.3601	9.64E-11	0.0852001
0.5719	0.5607	0.02491	0.0461	3.27E-16	0.5713
0.776	0.7314	0.02898	0.0516	1.91E-38	0.0368799
0.3592	0.6809	0.02921	0.0497	1.19E-32	0.21
0.3039	0.2789	0.0392	0.0513	4.22E-11	0.3248
0.199	0.05088	0.04579	0.1066	1.22E-13	0.1983
0.1845	0.3626	0.03974	0.0472	2.60E-145	0.0579002
0.0565	0.05421	0.0784	0.1021	2.26E-10	0.8432
0.258	0.1541	0.04209	0.0635	1.15E-17	0.7535
0.0709	0.03757	0.07038	0.1224	4.12E-12	0.592
0.0042	0.001315	0.1969	0.6881	2.46E-08	0.8408
0.0029	0.02198	0.2311	0.1568	7.08E-14	0.1846
0.0543	0.04058	0.05523	0.1145	2.78E-28	0.2585
0.0042	0.001315	0.1963	0.6881	4.99E-08	0.8408
0.0029	0.02198	0.2288	0.1568	8.60E-21	0.1846
0.0212	0.008734	0.0867	0.2645	3.04E-09	0.3686
0.2069	0.1508	0.03007	0.0653	4.86E-08	0.2124
0.5715	0.4562	0.02438	0.0457	8.25E-09	0.3237
0.3072	0.544	0.02853	0.0455	8.69E-09	0.3239
0.1051	0.0953	0.04155	0.0779	3.00E-14	0.367
0.0232	0.01469	0.08914	0.1922	7.97E-13	0.3905

0.0519	0.001482	0.06208	0.6165	3.33E-09	0.201
0.1737	0.1294	0.0366	0.069	5.27E-09	0.667999
0.4102	0.3577	0.02762	0.0477	3.66E-08	0.9637
0.0343	0.005012	0.06715	0.3584	1.20E-13	0.6805
0.0468	0.02801	0.05918	0.1422	9.08E-11	0.0465404
0.0756	0.02538	0.04773	0.1442	1.58E-31	0.5585
0.1416	0.2239	0.03584	0.0554	5.84E-20	0.4435
0.0871	0.01114	0.04479	0.2277	3.19E-40	0.7791
0.0343	0.005012	0.06723	0.3584	1.09E-13	0.6805
0.0468	0.02801	0.05926	0.1422	3.16E-10	0.0465404
0.0463	0.01167	0.0588	0.2211	4.03E-09	0.671399
0.099	0.08743	0.0339	0.081	0.00E+00	0.484
0.0756	0.02538	0.04773	0.1442	1.06E-32	0.5585
0.1416	0.2239	0.03585	0.0554	4.12E-21	0.4435
0.0871	0.01114	0.04478	0.2277	7.31E-42	0.7791
0.0343	0.005012	0.06729	0.3584	4.34E-13	0.6805
0.0468	0.02801	0.05933	0.1422	6.66E-10	0.0465404
0.0463	0.01167	0.05887	0.2211	1.41E-08	0.671399
0.0756	0.02538	0.04776	0.1442	1.54E-34	0.5585
0.1416	0.2239	0.03586	0.0554	2.13E-22	0.4435
0.3344	0.5043	0.02702	0.0457	1.93E-08	0.585901
0.0871	0.01114	0.04477	0.2277	4.41E-45	0.7791
0.099	0.08695	0.03426	0.0811	0.00E+00	0.4554
0.0343	0.005012	0.06729	0.3584	9.69E-14	0.6805
0.0463	0.01166	0.05888	0.2214	4.98E-09	0.670601
0.1416	0.2239	0.0359	0.0554	6.20E-21	0.4435
0.3344	0.5043	0.02699	0.0457	1.31E-10	0.585901
0.0871	0.01114	0.04492	0.2277	5.24E-40	0.7791
0.0762	0.0281	0.04755	0.1378	5.97E-33	0.740699
0.0343	0.005012	0.06712	0.3584	5.39E-14	0.6805
0.0468	0.02801	0.0592	0.1422	6.49E-10	0.0465404
0.0756	0.02538	0.04769	0.1442	2.95E-32	0.5585
0.1416	0.2239	0.03582	0.0554	1.22E-20	0.4435
0.0871	0.01114	0.04478	0.2277	3.37E-40	0.7791
0.0343	0.005012	0.06701	0.3584	3.73E-13	0.6805
0.0463	0.01166	0.05866	0.2214	1.44E-08	0.670601
0.1416	0.2239	0.03572	0.0554	3.98E-23	0.4435
0.0871	0.01114	0.04459	0.2277	7.62E-49	0.7791
0.0762	0.0281	0.04729	0.1378	3.88E-38	0.740699
0.0273	0.04052	0.07946	0.1175	1.47E-09	0.8954
0.2827	0.3882	0.02829	0.0466	1.54E-08	0.0312299
0.1343	0.09292	0.0377	0.0777	3.91E-09	0.4006
0.0175	0.01176	0.09653	0.2119	1.29E-08	0.5864
0.3258	0.5036	0.02569	0.0456	1.62E-62	0.5836
0.1835	0.2031	0.03172	0.0565	4.55E-09	0.9602
0.146	0.2525	0.03341	0.0524	2.07E-83	0.6859
0.0066	0.02218	0.1494	0.1518	2.48E-08	0.561
0.0048	0.02246	0.1845	0.1555	4.78E-08	0.4655
0.1839	0.008356	0.03634	0.2583	4.32E-08	0.0398602
0.2425	0.3066	0.02879	0.0498	5.44E-17	0.2701
0.0972	0.027	0.04226	0.1409	3.86E-08	0.795
0.0971	0.05677	0.04098	0.0995	5.41E-10	0.1631
0.1983	0.124	0.03293	0.069	2.33E-34	0.04134
0.1976	0.1253	0.0323	0.0689	1.68E-27	0.0247098
0.0449	0.04459	0.06505	0.11	9.29E-12	0.7751
0.4387	0.2663	0.0273	0.0517	3.44E-09	0.3011
0.411	0.3162	0.02783	0.0495	4.55E-08	0.6517
0.1974	0.05865	0.03421	0.098	1.06E-09	0.8625

0.0365	0.02483	0.06518	0.1486	1.02E-28	0.3053
0.0855	0.004361	0.04531	0.3649	2.12E-39	0.9783
0.0648	0.02027	0.05089	0.1696	2.80E-10	0.8078
0.0409	0.04949	0.06406	0.1042	4.53E-08	0.7526
0.0129	0.03811	0.1086	0.1196	3.59E-20	0.547299
0.1053	0.08695	0.03364	0.0811	0.00E+00	0.455
0.2587	0.3136	0.03003	0.0488	7.29E-10	0.2776
0.2441	0.2165	0.02935	0.0553	4.42E-28	0.1629
0.0321	0.02762	0.07737	0.1489	3.31E-08	0.692201
0.0387	0.02392	0.06851	0.1562	4.36E-10	0.8478
0.0029	0.02198	0.1943	0.1568	1.14E-16	0.1846
0.9971	0.9852	0.2032	0.2027	1.46E-08	0.3086
0.0003	0.04176	0.5579	0.1153	3.03E-08	0.1376
0.0029	0.02198	0.1946	0.1568	3.06E-19	0.1846
0.9971	0.9852	0.2039	0.2027	2.52E-08	0.3086
0.0929	0.1108	0.03056	0.0725	3.80E-08	0.3505
0.5109	0.541	0.021	0.0469	2.27E-09	0.2467
0.001	0.01049	0.3644	0.2273	9.93E-09	0.955
0.319	0.03521	0.02572	0.1253	1.60E-08	0.6524
0.0354	0.007566	0.06609	0.2613	3.35E-10	0.4245
0.1634	0.2353	0.03225	0.0532	1.74E-08	0.0392998
0.5685	0.3828	0.02375	0.047	1.21E-15	0.753101
0.001	0.01049	0.3629	0.2273	4.36E-09	0.955
0.4999	0.2247	0.02361	0.0549	8.45E-09	0.1255
0.5685	0.3828	0.0237	0.047	1.08E-12	0.753101
0.0704	0.002626	0.0559	0.4801	3.84E-16	0.5019
0.0798	0.04891	0.04579	0.1071	1.06E-11	0.953
0.5834	0.5164	0.0279	0.0518	3.04E-98	0.75
0.4388	0.245	0.02936	0.0538	1.21E-68	0.433
0.4388	0.2252	0.02936	0.0681	1.21E-68	0.8549
0.5433	0.505	0.02801	0.0519	2.52E-21	0.4179
0.0704	0.002626	0.05597	0.4801	2.31E-15	0.5019
0.0798	0.04891	0.04587	0.1071	7.74E-12	0.953
0.5834	0.5164	0.02804	0.0518	5.56E-92	0.75
0.4388	0.245	0.02947	0.0538	3.56E-64	0.433
0.4388	0.2252	0.02947	0.0681	3.56E-64	0.8549
0.5433	0.505	0.02805	0.0519	2.56E-20	0.4179
0.072	0.002626	0.0721	0.4801	9.55E-11	0.5019
0.2237	0.3723	0.03825	0.0471	1.72E-28	0.1166
0.3892	0.4641	0.03772	0.0522	2.30E-10	0.4961
0.5755	0.5164	0.03887	0.0518	5.46E-26	0.749701
0.4372	0.245	0.03985	0.0538	8.99E-20	0.433
0.4372	0.2252	0.03985	0.0681	8.99E-20	0.8549
0.0444	0.002425	0.06725	0.4875	1.90E-08	0.4712
0.1171	0.1517	0.04087	0.0641	1.54E-83	0.0200101
0.481	0.4828	0.02583	0.0458	3.02E-11	0.0241102
0.2561	0.3333	0.02975	0.0483	1.09E-10	0.517001
0.1758	0.1344	0.03444	0.067	2.69E-23	0.3903
0.1482	0.2489	0.03854	0.0529	7.97E-13	0.1369
0.4466	0.26	0.02499	0.0523	3.76E-132	0.5358
0.6622	0.731	0.02866	0.0516	1.69E-09	0.4604
0.1482	0.2489	0.03832	0.0529	1.93E-12	0.1369
0.4466	0.2595	0.02516	0.0523	1.10E-115	0.6974
0.0512	0.1128	0.06122	0.0732	3.64E-13	0.5532
0.5978	0.5899	0.02764	0.0468	7.71E-12	0.779301
0.1462	0.1595	0.03794	0.0625	3.88E-08	0.4304
0.1961	0.09891	0.03418	0.0766	3.94E-08	0.215
0.0512	0.1128	0.06029	0.0732	8.31E-13	0.5532

0.0154	0.02295	0.1101	0.1567	4.57E-11	0.1102
0.1057	0.1082	0.04392	0.0735	2.58E-16	0.8692
0.5197	0.2723	0.02746	0.0513	2.35E-08	0.2333
0.1227	0.1276	0.04135	0.0673	2.66E-08	0.097499
0.0031	0.02497	0.247	0.1471	4.46E-08	0.2234
0.0559	0.02981	0.05365	0.1348	1.32E-16	0.0199701
0.7745	0.7574	0.03328	0.0538	1.02E-10	0.2916
0.1635	0.05233	0.03765	0.1027	4.51E-08	0.715201
0.8197	0.8486	0.03173	0.0639	2.33E-12	0.5865
0.2303	0.3065	0.02943	0.0498	1.42E-09	0.2921
0.6088	0.5047	0.02485	0.0456	7.90E-16	0.1821
0.3162	0.2954	0.02688	0.0497	1.64E-08	0.151
0.5887	0.5173	0.02962	0.0518	1.55E-09	0.7185
0.8197	0.8486	0.03182	0.0639	3.02E-11	0.5865
0.6079	0.5115	0.02489	0.0455	4.63E-13	0.173
0.401	0.3458	0.02909	0.0612	3.55E-10	0.0959798
0.7669	0.6927	0.0293	0.0497	1.12E-11	0.2845
0.2216	0.2748	0.02956	0.0509	2.25E-08	0.1658
0.074	0.00329	0.04714	0.4102	3.61E-21	0.8367
0.6084	0.3782	0.02511	0.0469	6.64E-31	0.2198
0.5233	0.534	0.0244	0.0456	1.77E-08	0.2677
0.7148	0.5608	0.04205	0.0461	1.74E-10	0.7416
0.2094	0.3626	0.0394	0.0472	2.39E-108	0.0579002
0.0499	0.02672	0.08664	0.1439	4.02E-12	0.3012
0.2702	0.1541	0.04316	0.0635	1.04E-12	0.7535
0.5574	0.548	0.03799	0.0461	2.62E-12	0.5991
0.3892	0.1267	0.02531	0.0692	8.43E-10	0.1106
0.8211	0.8344	0.03209	0.0618	6.08E-09	0.2283
0.0967	0.04117	0.04166	0.1166	7.49E-10	0.1437
0.2303	0.3065	0.02926	0.0498	3.22E-11	0.2921
0.2521	0.2451	0.03039	0.0531	1.56E-11	0.3337
0.4556	0.2689	0.02598	0.0516	4.92E-49	0.7098
0.2279	0.2471	0.03133	0.053	9.38E-33	0.2517
0.0459	0.004779	0.07912	0.3262	3.62E-10	0.4979
0.2402	0.1086	0.03104	0.0736	3.47E-51	0.329
0.5436	0.3841	0.02926	0.052	8.44E-10	0.3813
0.1067	0.1641	0.04744	0.0639	4.17E-08	0.3457
0.6062	0.1169	0.02977	0.0708	1.72E-23	0.1247
0.2255	0.07128	0.03366	0.0879	3.47E-08	0.1665
0.5065	0.3314	0.02942	0.0523	6.34E-09	0.5031
0.2125	0.0107	0.03386	0.2203	8.28E-19	0.9948
0.0277	0.007397	0.08527	0.2719	1.76E-15	0.780001
0.8374	0.9147	0.03753	0.0822	3.36E-08	0.8716
0.3354	0.4627	0.02895	0.0462	1.77E-14	0.9772
0.0277	0.007408	0.08507	0.2718	7.25E-10	0.777301
0.1973	0.05865	0.0346	0.098	2.11E-09	0.8625
0.2966	0.1244	0.02834	0.0686	1.22E-10	0.7019
0.633	0.4193	0.03113	0.0486	2.26E-09	0.2146
0.2969	0.1244	0.02646	0.0686	7.83E-10	0.7019
0.2969	0.1244	0.0281	0.0686	1.26E-08	0.7019
0.0705	0.002922	0.04962	0.432	1.09E-10	0.649999
0.0457	0.003723	0.06475	0.3907	8.83E-11	0.0329602
0.4733	0.5401	0.02562	0.0456	9.56E-57	0.7871
0.4777	0.208	0.02404	0.0563	1.83E-10	0.0459304
0.319	0.03521	0.02633	0.1253	9.00E-18	0.6524
0.3256	0.03521	0.02625	0.1253	4.02E-12	0.6524
0.6359	0.727	0.02629	0.0509	7.20E-09	0.9587
0.02	0.0162	0.08807	0.1796	6.54E-10	0.8315

0.3256	0.03521	0.02601	0.1253	5.62E-14	0.6524
0.3256	0.03521	0.02661	0.1253	9.14E-11	0.6524
0.3256	0.03521	0.02639	0.1253	8.62E-09	0.6524
0.02	0.0162	0.08845	0.1796	2.09E-08	0.8315
0.3256	0.03521	0.02663	0.1253	1.39E-10	0.6524
0.3195	0.03521	0.02651	0.1253	1.43E-16	0.6524
0.597	0.6099	0.02469	0.0466	2.18E-10	0.7692
0.3256	0.03521	0.02653	0.1253	2.04E-10	0.6524
0.2038	0.4347	0.03371	0.0458	1.49E-09	0.0498597
0.4466	0.26	0.02485	0.0523	1.66E-133	0.5358
0.0512	0.1128	0.06087	0.0732	1.51E-18	0.5532
0.2093	0.1083	0.03386	0.0736	1.23E-11	0.1833
0.2826	0.3882	0.0284	0.0466	3.59E-08	0.0312299
0.5219	0.3083	0.02565	0.0493	1.05E-10	0.4086
0.2757	0.05315	0.03508	0.1008	6.75E-13	0.1725
0.4483	0.3127	0.03074	0.0523	8.61E-11	0.2841
0.1792	0.1525	0.03302	0.0632	2.87E-08	0.4131
0.0032	0.004446	0.2563	0.3457	3.42E-12	0.5019
0.3011	0.1198	0.02696	0.0692	1.21E-19	0.985
0.0732	0.02004	0.04765	0.1656	4.97E-11	0.1499
0.0468	0.02801	0.05894	0.1422	1.33E-10	0.0465404
0.0463	0.01166	0.05854	0.2214	1.63E-08	0.670601
0.0756	0.02538	0.04748	0.1442	6.25E-32	0.5585
0.0261	0.0152	0.07793	0.1935	2.29E-20	0.4553
0.3344	0.5043	0.02684	0.0457	2.29E-08	0.585901
0.0871	0.01114	0.04464	0.2277	1.63E-37	0.7791
0.099	0.08647	0.03429	0.0813	1.76399999	0.449
0.0468	0.02801	0.05912	0.1422	2.10E-09	0.0465404
0.0463	0.01166	0.05867	0.2214	3.41E-08	0.670601
0.0756	0.02538	0.0476	0.1442	2.82E-33	0.5585
0.0261	0.0152	0.07811	0.1935	2.74E-20	0.4553
0.3344	0.5043	0.02692	0.0457	4.18E-08	0.585901
0.0871	0.01114	0.04473	0.2277	3.89E-40	0.7791
0.099	0.08647	0.03453	0.0813	1.85E-306	0.449
0.1101	0.09757	0.04291	0.077	4.67E-11	0.1773
0.3265	0.5442	0.02858	0.0455	2.20E-10	0.3306
0.2366	0.1953	0.03217	0.0573	8.94E-09	0.8123
0.0315	0.01769	0.07123	0.1832	2.73E-08	0.3447
0.4173	0.4836	0.02575	0.0454	4.75E-09	0.1235
0.766	0.7338	0.02777	0.0517	1.40E-25	0.0563495
0.199	0.1175	0.03356	0.0707	5.58E-45	0.1025
0.1779	0.05297	0.03583	0.1008	4.70E-08	0.2164
0.3781	0.6129	0.02476	0.047	9.92E-10	0.0365603
0.5236	0.534	0.02438	0.0456	1.16E-09	0.2676
0.1941	0.05865	0.02999	0.098	1.36E-09	0.8625
0.0035	0.01555	0.2241	0.1806	2.06E-09	0.3913
0.0029	0.02198	0.2241	0.1568	9.63E-12	0.1846
0.9748	0.9604	0.08043	0.1166	9.52E-09	0.9826
0.0523	0.009082	0.05758	0.248	5.09E-11	0.8013
0.223	0.1104	0.03417	0.0728	4.39E-09	0.6409
0.0443	0.1363	0.07319	0.07	2.15E-08	0.2089
0.4567	0.49	0.0302	0.0487	4.31E-81	0.8913
0.2446	0.006063	0.03579	0.3	1.06E-13	0.9568
0.7804	0.9021	0.03527	0.0775	4.55E-10	0.1106
0.014	0.007996	0.1144	0.268	9.40E-09	0.0782096
0.0144	0.01422	0.1169	0.2003	5.08E-12	0.1602
0.0079	0.03755	0.1549	0.119	4.89E-09	0.3653
0.0074	0.02955	0.1607	0.1365	1.12E-11	0.9248

0.0277	0.03914	0.08106	0.1158	3.17E-20	0.6005
0.1974	0.05865	0.03411	0.098	4.02E-20	0.8625
0.0188	0.02379	0.1013	0.1504	2.06E-11	0.4792
0.0312	0.01471	0.07557	0.2013	1.48E-08	0.4496
0.189	0.1867	0.03275	0.0595	5.01E-11	0.2692
0.6741	0.4166	0.02733	0.0463	5.51E-10	0.7959
0.0315	0.01471	0.06958	0.2013	2.69E-09	0.4496
0.1915	0.1895	0.0304	0.0591	2.49E-14	0.3337
0.0378	0.02382	0.06302	0.1566	2.02E-08	0.8106
0.6738	0.4166	0.02517	0.0463	2.39E-08	0.7959
0.0315	0.01471	0.07147	0.2013	2.99E-15	0.4496
0.0378	0.02392	0.06448	0.1562	4.31E-14	0.8478
0.0315	0.01471	0.0704	0.2013	9.72E-11	0.4496
0.1915	0.1895	0.03076	0.0591	1.70E-15	0.3337
0.0378	0.02382	0.0638	0.1566	8.33E-09	0.8106
0.0316	0.01471	0.07016	0.2013	8.63E-12	0.4496
0.0379	0.02382	0.06368	0.1566	2.87E-11	0.8106
0.0315	0.01471	0.07237	0.2013	6.10E-13	0.4496
0.0378	0.02392	0.06515	0.1562	5.86E-13	0.8478
0.0315	0.01471	0.07983	0.2013	3.78E-08	0.4496
0.1973	0.1252	0.03405	0.069	2.31E-36	0.0162499
0.0389	0.02392	0.07094	0.1562	5.94E-11	0.8478
0.0316	0.01471	0.07284	0.2013	2.44E-10	0.4496
0.0328	0.06289	0.07011	0.0943	2.22E-11	0.542099
0.5116	0.4	0.02493	0.0465	2.84E-08	0.23
0.1096	0.01461	0.03904	0.192	2.79E-25	0.8397
0.0368	0.002429	0.06295	0.448	1.57E-08	0.4156
0.0814	0.05682	0.04422	0.0979	6.16E-09	0.667
0.0482	0.01192	0.07926	0.2185	8.25E-09	0.746299
0.0762	0.02538	0.06459	0.1442	6.78E-18	0.5585
0.025	0.0152	0.1109	0.1935	4.23E-10	0.4553
0.0887	0.01114	0.05961	0.2277	1.02E-24	0.7791
0.0944	0.08647	0.04742	0.0813	9.85E-175	0.449
0.1016	0.01051	0.05917	0.2286	3.99E-20	0.0739605
0.1622	0.188	0.04858	0.0586	1.89E-08	0.0609705
0.4036	0.6243	0.036	0.0475	1.01E-12	0.743599
0.5545	0.5302	0.03686	0.0457	1.29E-08	0.5954
0.0479	0.02755	0.0642	0.1354	8.24E-19	0.2095
0.4422	0.2507	0.02793	0.053	1.17E-12	0.3862
0.8955	0.9623	0.04516	0.1209	4.79E-19	0.9239
0.2457	0.1086	0.0314	0.0736	1.06E-25	0.329
0.0479	0.02755	0.06432	0.1354	1.36E-19	0.2095
0.4422	0.2507	0.02793	0.053	7.54E-16	0.3862
0.8964	0.9632	0.04526	0.1222	1.96E-24	0.9004
0.0481	0.02755	0.06195	0.1354	2.34E-72	0.2095
0.016	0.037	0.1067	0.1187	4.18E-08	0.0139701
0.4402	0.3676	0.02843	0.0478	7.77E-09	0.1777
0.8956	0.9633	0.04462	0.1222	5.12E-48	0.8754
0.1204	0.05648	0.04248	0.0986	2.61E-16	0.3102
0.0126	0.03193	0.1209	0.1315	6.94E-11	0.6608
0.0368	0.002595	0.06402	0.4462	2.07E-08	0.3764
0.0864	0.06796	0.04674	0.0958	8.82E-09	0.9739
0.657	0.7849	0.02772	0.0552	3.16E-08	0.4459
0.0096	0.04919	0.1315	0.1046	1.32E-08	0.5615
0.6904	0.6174	0.02618	0.0472	1.17E-20	0.1649
0.0336	0.03386	0.06803	0.1262	1.02E-09	0.7067
0.0688	0.0008435	0.07698	0.8103	3.34E-09	0.1907
0.3188	0.3021	0.04395	0.058	3.10E-10	0.9782

0.5722	0.5164	0.04022	0.0518	7.98E-26	0.7501
0.0573	0.01072	0.05919	0.2217	1.81E-08	0.1324
0.245	0.2063	0.03237	0.0563	1.20E-08	0.487401
0.4102	0.3577	0.02772	0.0477	3.49E-09	0.9637
0.0378	0.01129	0.07353	0.216	4.41E-08	0.3384
0.0572	0.01072	0.05796	0.2217	1.55E-08	0.1324
0.2142	0.01973	0.03399	0.1695	9.17E-13	0.6997
0.4183	0.2887	0.02745	0.0503	1.45E-55	0.793999
0.2142	0.01973	0.03394	0.1695	1.10E-12	0.6997
0.4183	0.2887	0.0274	0.0503	1.56E-55	0.793999
0.2589	0.3008	0.02824	0.0494	5.07E-09	0.261
0.0798	0.04891	0.0448	0.1071	6.79E-12	0.953
0.1941	0.05865	0.03068	0.098	6.35E-09	0.8625
0.2848	0.1071	0.03321	0.0745	3.77E-15	0.0312198
0.1551	0.0567	0.039	0.1015	5.66E-26	0.9565
0.6053	0.4904	0.02907	0.0538	1.07E-35	0.9662
0.0032	0.005652	0.2873	0.3174	4.57E-08	0.3791
0.3011	0.1198	0.02757	0.0692	1.03E-12	0.985
0.0495	0.02453	0.06077	0.1472	4.55E-08	0.3966
0.1078	0.0786	0.04123	0.0855	3.87E-08	0.6016
0.0416	0.09206	0.0615	0.0784	7.55E-10	0.916
0.1076	0.03644	0.04039	0.1185	1.74E-31	0.1517
0.0414	0.09206	0.0617	0.0784	5.60E-13	0.916
0.0997	0.03243	0.04201	0.127	3.86E-08	0.4094
0.7759	0.7776	0.02956	0.0549	4.56E-11	0.9043
0.1075	0.03644	0.04021	0.1185	2.21E-43	0.1517
0.0414	0.09206	0.06126	0.0784	4.00E-12	0.916
0.0997	0.03243	0.04168	0.127	2.29E-08	0.4094
0.7759	0.7776	0.02934	0.0549	1.11E-10	0.9043
0.1075	0.03642	0.03976	0.1186	1.32E-47	0.1504
0.4557	0.49	0.03138	0.0487	5.98E-09	0.8913
0.0415	0.09206	0.06144	0.0784	6.12E-12	0.916
0.776	0.7776	0.0295	0.0549	2.19E-08	0.9043
0.1077	0.03644	0.04036	0.1185	1.68E-32	0.1517
0.0414	0.09206	0.06162	0.0784	1.20E-11	0.916
0.0062	0.004792	0.1592	0.3369	6.27E-09	0.4208
0.1075	0.03642	0.04021	0.1186	1.25E-40	0.1504
0.2437	0.1212	0.03117	0.0717	1.79E-14	0.4933
0.1096	0.01174	0.04218	0.2128	1.17E-15	0.4602
0.0434	0.04459	0.06802	0.11	4.86E-08	0.7751
0.0536	0.02426	0.0547	0.1494	1.35E-08	0.709601
0.4057	0.4544	0.02525	0.0462	6.78E-09	0.7074
0.0991	0.08647	0.04055	0.0813	4.96E-38	0.449
0.1327	0.0614	0.0365	0.0966	3.92E-15	0.3182
0.0986	0.1517	0.05893	0.0641	1.70E-08	0.0183599
0.4249	0.3995	0.02501	0.0465	3.61E-08	0.9053
0.8748	0.8829	0.03692	0.0712	1.85E-08	0.722
0.2425	0.3066	0.0287	0.0498	1.61E-13	0.2701
0.8748	0.8829	0.03693	0.0712	1.10E-09	0.722
0.0965	0.04117	0.04169	0.1166	8.13E-11	0.1437
0.2304	0.3065	0.02911	0.0498	1.71E-20	0.2921
0.1025	0.01417	0.05014	0.1985	8.25E-09	0.4176
0.1766	0.07404	0.0339	0.0874	1.11E-12	0.8694
0.1155	0.07865	0.03926	0.0855	3.34E-08	0.3776
0.1766	0.07395	0.03486	0.0874	4.17E-09	0.8778
0.3258	0.5436	0.02859	0.0455	1.65E-21	0.32
0.0407	0.02283	0.06832	0.1582	3.72E-23	0.971
0.0979	0.1339	0.04671	0.067	4.36E-08	0.3197

0.017	0.02334	0.1066	0.1499	2.10E-08	0.8423
0.3258	0.5436	0.02852	0.0455	3.01E-13	0.32
0.0407	0.02283	0.06868	0.1582	2.61E-08	0.971
0.3265	0.5442	0.02865	0.0455	1.94E-08	0.3306
0.4481	0.2593	0.02617	0.0524	1.39E-57	0.681
0.0512	0.1128	0.06121	0.0732	3.66E-09	0.5532
0.2635	0.3008	0.03729	0.0494	1.49E-11	0.261
0.2659	0.1564	0.03572	0.0626	2.20E-08	0.2689
0.2128	0.3626	0.03761	0.0472	1.29E-12	0.0579002
0.029	0.03364	0.08634	0.1277	2.13E-09	0.02355
0.8223	0.612	0.03754	0.0486	6.16E-22	0.0975911
0.1384	0.09641	0.03961	0.0785	7.15E-14	0.1593
0.2767	0.4343	0.03263	0.0564	4.52E-08	0.6687
0.4579	0.2693	0.02759	0.0516	1.57E-31	0.7247
0.4569	0.2689	0.02643	0.0516	7.82E-39	0.7098
0.4556	0.2689	0.02585	0.0516	1.01E-61	0.7098
0.4562	0.2689	0.02586	0.0516	9.84E-58	0.7098
0.1062	0.1082	0.04309	0.0735	4.21E-09	0.8692
0.4559	0.2689	0.02723	0.0516	2.21E-23	0.7098
0.4568	0.2689	0.02631	0.0516	6.14E-53	0.7098
0.4567	0.2689	0.0263	0.0516	7.25E-43	0.7098
0.0735	0.01994	0.05153	0.1604	3.92E-08	0.6818
0.255	0.2063	0.04091	0.0563	8.80E-10	0.487401
0.0029	0.02198	0.2336	0.1568	1.15E-10	0.1846
0.3439	0.4487	0.03185	0.049	1.63E-08	0.0111699
0.0029	0.02198	0.2111	0.1568	6.94E-15	0.1846
0.814	0.9725	0.03078	0.1435	1.33E-11	0.450001
0.4699	0.5257	0.02348	0.0454	3.10E-20	0.293
0.0029	0.02198	0.2221	0.1568	1.56E-11	0.1846
0.5099	0.541	0.02649	0.0469	1.65E-08	0.2468
0.3011	0.1198	0.02442	0.0692	8.11E-11	0.985
0.5287	0.5857	0.02833	0.0463	1.18E-08	0.04157
0.0542	0.04058	0.05665	0.1145	2.40E-22	0.2585
0.2288	0.1094	0.03351	0.0723	3.35E-10	0.7693
0.013	0.02224	0.12	0.1574	3.96E-08	0.6317
0.8652	0.9043	0.04072	0.0776	2.08E-08	0.0569403
0.4845	0.283	0.02703	0.0508	8.20E-35	0.8655
0.4552	0.2693	0.0264	0.0516	6.52E-26	0.726999
0.0027	0.006415	0.2268	0.3009	2.63E-09	0.0467498
0.8286	0.9321	0.03253	0.0906	6.12E-10	0.1648
0.0555	0.02979	0.0546	0.1349	1.28E-15	0.01992
0.0798	0.04891	0.04402	0.1071	2.37E-10	0.953
0.0798	0.04891	0.04587	0.1071	2.51E-08	0.953
0.2849	0.1071	0.03302	0.0745	1.61E-14	0.0312198
0.5834	0.5164	0.02808	0.0518	3.21E-78	0.7501
0.4388	0.245	0.02935	0.0538	9.60E-57	0.433
0.4388	0.2252	0.02935	0.0681	9.60E-57	0.8549
0.5433	0.505	0.02781	0.0519	8.23E-21	0.4179
0.0798	0.04891	0.04479	0.1071	2.82E-08	0.953
0.447	0.3436	0.02487	0.048	9.48E-14	0.621299
0.0236	0.0176	0.07909	0.1705	8.02E-09	0.344
0.0844	0.04891	0.04984	0.1071	1.61E-08	0.953
0.5089	0.2356	0.02875	0.0543	1.89E-09	0.0661607
0.3334	0.5036	0.03437	0.0456	2.77E-54	0.5836
0.9459	0.8858	0.07416	0.0716	7.11E-09	0.5413
0.2209	0.2246	0.04027	0.0543	7.44E-19	0.3116
0.3267	0.5036	0.02562	0.0456	2.06E-70	0.5836
0.0427	0.1087	0.06265	0.0726	1.03E-08	0.4078

0.2729	0.1558	0.02838	0.0631	3.17E-12	0.6664
0.1461	0.2525	0.03329	0.0524	3.78E-92	0.6859
0.3209	0.5036	0.02982	0.0456	1.36E-16	0.5836
0.8668	0.8708	0.04088	0.069	9.63E-30	0.2859
0.3302	0.5036	0.03798	0.0456	1.47E-18	0.5836
0.3309	0.5036	0.03828	0.0456	5.24E-32	0.5836
0.3298	0.5036	0.0387	0.0456	3.93E-11	0.5836
0.2122	0.3626	0.03358	0.0472	2.39E-191	0.0579002
0.5707	0.5797	0.03609	0.0462	1.33E-08	0.0691799
0.0484	0.02672	0.08124	0.1439	2.01E-16	0.3012
0.0704	0.004544	0.07133	0.348	6.18E-12	0.5298
0.271	0.1541	0.04	0.0635	8.18E-22	0.7535
0.5533	0.548	0.03574	0.0461	5.83E-16	0.5991
0.3262	0.5036	0.02603	0.0456	4.45E-54	0.5836
0.2681	0.2998	0.02846	0.05	6.28E-17	0.560701
0.3334	0.5036	0.03439	0.0456	2.53E-54	0.5836
0.9459	0.8858	0.07422	0.0716	8.70E-09	0.5413
0.2209	0.2246	0.04034	0.0543	3.71E-18	0.3116
0.3334	0.5036	0.03479	0.0456	8.34E-14	0.5836
0.3303	0.5036	0.03633	0.0456	6.27E-61	0.5836
0.3268	0.5036	0.02599	0.0456	6.57E-20	0.5836
0.3166	0.5036	0.03296	0.0456	1.06E-20	0.5836
0.3305	0.5036	0.03702	0.0456	1.43E-38	0.5836
0.3267	0.5036	0.02624	0.0456	3.85E-08	0.5836
0.3299	0.5036	0.03857	0.0456	1.84E-11	0.5836
0.2121	0.3626	0.03392	0.0472	2.40E-183	0.0579002
0.0483	0.02672	0.08096	0.1439	1.96E-17	0.3012
0.0278	0.007014	0.105	0.2805	7.89E-09	0.4083
0.2708	0.1541	0.03992	0.0635	2.91E-21	0.7535
0.5535	0.548	0.03561	0.0461	1.33E-16	0.5991
0.3262	0.5036	0.02605	0.0456	2.10E-64	0.5836
0.2681	0.2998	0.02853	0.05	6.00E-23	0.560701
0.327	0.5036	0.02573	0.0456	2.62E-69	0.5836
0.2677	0.2998	0.02862	0.05	3.51E-08	0.562
0.3209	0.5036	0.02934	0.0456	1.20E-20	0.5836
0.3209	0.5036	0.02951	0.0456	3.69E-18	0.5836
0.6884	0.6174	0.02975	0.0472	2.69E-27	0.1649
0.0544	0.04058	0.05534	0.1145	6.70E-28	0.2585
0.0356	0.007566	0.06648	0.2613	3.83E-09	0.4245
0.1622	0.2354	0.03265	0.0532	6.55E-10	0.0405005
0.5686	0.3828	0.02398	0.047	8.82E-17	0.753101
0.0357	0.007566	0.07408	0.2613	6.88E-12	0.4245
0.3466	0.3084	0.02785	0.0493	1.34E-15	0.5403
0.0097	0.03052	0.1388	0.1453	1.34E-08	0.5222
0.0194	0.01518	0.089	0.1876	5.54E-18	0.3459
0.9403	0.9446	0.05158	0.1001	8.42E-10	0.0813299
0.6318	0.73	0.02576	0.0511	4.90E-13	0.9949
0.2335	0.3065	0.02929	0.0498	8.55E-11	0.2921
0.8194	0.8234	0.0321	0.06	2.24E-09	0.2581
0.0972	0.027	0.04232	0.1409	2.51E-08	0.795
0.6317	0.73	0.02506	0.0511	2.98E-83	0.9949
0.167	0.1852	0.03366	0.0591	1.15E-08	0.2733
0.0561	0.01053	0.05335	0.2321	2.35E-43	0.244
0.035	0.01079	0.06834	0.2353	5.54E-13	0.1699
0.1136	0.01493	0.03994	0.19	1.18E-09	0.461
0.0223	0.0291	0.08703	0.137	5.04E-11	0.6555
0.6323	0.73	0.02594	0.0511	3.69E-08	0.9949
0.534	0.6136	0.02462	0.0467	1.07E-11	0.3091

0.6316	0.73	0.02558	0.0511	3.11E-19	0.9949
0.1067	0.03695	0.0403	0.1178	3.50E-15	0.1723
0.5778	0.4816	0.0385	0.0456	5.64E-14	0.9919
0.5778	0.4816	0.03886	0.0456	2.17E-14	0.9919
0.5778	0.4816	0.03847	0.0456	2.10E-15	0.9919
0.5778	0.4816	0.03841	0.0456	1.91E-13	0.9919
0.5778	0.4816	0.03952	0.0456	8.55E-13	0.9919
0.5778	0.4816	0.03948	0.0456	7.00E-13	0.9919
0.0278	0.001989	0.1109	0.5605	1.96E-08	0.8
0.0043	0.0229	0.2431	0.1521	8.93E-11	0.0909997
0.0413	0.03954	0.06981	0.117	4.50E-08	0.1864
0.0865	0.00412	0.04325	0.3749	1.38E-09	0.614301
0.3435	0.2549	0.02525	0.0525	5.63E-28	0.4215
0.0867	0.00412	0.04411	0.3749	1.72E-09	0.614301
0.344	0.2549	0.02564	0.0525	1.35E-31	0.4215
0.6081	0.5115	0.02481	0.0455	3.16E-15	0.173
0.2304	0.3065	0.02948	0.0498	1.67E-08	0.2921
0.5205	0.6131	0.0244	0.0468	3.93E-08	0.793201
0.3161	0.2954	0.02688	0.0497	5.42E-09	0.151
0.5889	0.5173	0.02943	0.0518	6.52E-09	0.7185
0.601	0.8069	0.02791	0.0579	3.15E-38	0.2228
0.199	0.1145	0.03402	0.0721	9.00E-12	0.8412
0.601	0.8069	0.02783	0.0579	2.24E-42	0.2228
0.199	0.1145	0.03397	0.0721	1.84E-13	0.8412
0.001	0.01409	6.487	0.2008	5.20E-09	0.5301
0.3508	0.4221	0.372	0.0463	1.96E-13	0.8607
0.1704	0.09572	0.03629	0.0781	1.46E-10	0.3036
0.446	0.26	0.02442	0.0523	5.66E-154	0.5358
0.051	0.1128	0.06086	0.0732	3.34E-19	0.5532
0.1325	0.1866	0.03734	0.0595	4.69E-13	0.2541
0.0747	0.2712	0.06024	0.0539	7.87E-09	0.3033
0.4074	0.3711	0.02485	0.0474	2.44E-11	0.01087
0.4557	0.49	0.0293	0.0487	2.05E-117	0.8913
0.3212	0.2328	0.04397	0.0572	1.12E-09	0.6409
0.4528	0.49	0.04125	0.0487	9.01E-57	0.8913
0.821	0.8344	0.03219	0.0618	2.95E-12	0.2283
0.6088	0.5047	0.02482	0.0456	2.29E-16	0.1821
0.3161	0.2954	0.02685	0.0497	9.87E-10	0.151
0.5889	0.5173	0.02952	0.0518	1.31E-08	0.7185
0.821	0.8344	0.03177	0.0618	1.13E-25	0.2283
0.3094	0.1514	0.02631	0.0637	1.75E-10	0.1306
0.3161	0.2954	0.02674	0.0497	2.45E-08	0.151
0.461	0.3691	0.03073	0.052	3.05E-08	0.3854
0.6102	0.5114	0.02475	0.0455	6.73E-13	0.1703
0.2834	0.2828	0.02971	0.0508	6.37E-09	0.5974
0.265	0.1443	0.03168	0.0649	1.00E-08	0.1743
0.7709	0.7355	0.03092	0.0518	6.18E-10	0.06785
0.3244	0.7077	0.02628	0.0501	2.12E-13	0.3229
0.1476	0.3094	0.03961	0.0521	2.75E-09	0.628001
0.0624	0.1244	0.05867	0.0692	7.75E-13	0.407
0.2369	0.1065	0.03167	0.074	2.59E-37	0.5083
0.4167	0.4839	0.02608	0.0454	3.17E-08	0.1197
0.766	0.7338	0.0278	0.0517	5.57E-52	0.0563495
0.1636	0.06788	0.03296	0.0918	1.36E-10	0.4132
0.4168	0.4839	0.02568	0.0454	2.59E-10	0.1197
0.7659	0.7338	0.02736	0.0517	3.62E-55	0.0563495
0.1635	0.06788	0.03253	0.0918	6.46E-10	0.4132
0.5882	0.4766	0.03927	0.0456	3.86E-13	0.3302

0.5754	0.4832	0.03896	0.0456	4.90E-11	0.499401
0.5747	0.4832	0.03976	0.0456	2.74E-09	0.499401
0.593	0.4885	0.03809	0.0456	1.02E-10	0.4071
0.593	0.4885	0.03896	0.0456	4.25E-09	0.4071
0.3841	0.1362	0.04325	0.0671	2.44E-10	0.0801401
0.5995	0.4955	0.02511	0.0457	2.96E-12	0.725499
0.0586	0.0299	0.05193	0.1344	2.52E-45	0.02305
0.5961	0.4955	0.03543	0.0457	2.92E-09	0.725499
0.0617	0.0998	0.07087	0.0749	3.42E-30	0.1346
0.5996	0.4955	0.0249	0.0457	6.00E-13	0.725499
0.0586	0.0299	0.05156	0.1344	6.21E-45	0.02305
0.5996	0.4955	0.02528	0.0457	1.36E-12	0.725499
0.0651	0.0998	0.05059	0.0749	2.21E-25	0.1346
0.5997	0.4955	0.02499	0.0457	9.31E-11	0.725499
0.065	0.0998	0.04955	0.0749	3.93E-41	0.1346
0.5998	0.4955	0.02534	0.0457	3.86E-08	0.725499
0.0651	0.0998	0.05081	0.0749	2.66E-17	0.1346
0.5998	0.4955	0.02478	0.0457	6.38E-13	0.725499
0.7284	0.7932	0.0275	0.0569	4.04E-08	0.4315
0.0584	0.0299	0.05093	0.1344	2.16E-61	0.02305
0.5997	0.4955	0.02524	0.0457	1.46E-11	0.725499
0.0586	0.0299	0.05233	0.1344	1.21E-40	0.02305
0.5997	0.4955	0.02484	0.0457	1.54E-11	0.725499
0.065	0.0998	0.04904	0.0749	1.16E-49	0.1346
0.6173	0.424	0.02845	0.0462	2.24E-10	0.6529
0.1965	0.05865	0.03249	0.098	3.84E-40	0.8625
0.5341	0.6136	0.02451	0.0467	3.13E-08	0.3091
0.6002	0.7114	0.02593	0.0502	1.64E-27	0.9527
0.5339	0.6136	0.02442	0.0467	2.13E-09	0.3091
0.0562	0.01053	0.05371	0.2321	5.92E-11	0.244
0.6314	0.729	0.02585	0.051	7.20E-50	0.9652
0.7711	0.7765	0.0399	0.0548	4.81E-08	0.9107
0.1068	0.03644	0.05533	0.1185	1.67E-18	0.1517
0.4529	0.49	0.04396	0.0487	1.84E-08	0.8913
0.3544	0.1742	0.02926	0.06	1.86E-08	0.0269799
0.0756	0.0361	0.05364	0.1219	4.79E-08	0.7848
0.3703	0.7412	0.02906	0.0532	1.89E-09	0.1404
0.5197	0.3884	0.03247	0.0468	1.25E-32	0.3663
0.1527	0.2218	0.04685	0.0548	2.20E-09	0.615
0.3672	0.3655	0.02605	0.0475	2.77E-08	0.3395
0.2461	0.1083	0.02805	0.0738	3.82E-82	0.3928
0.4243	0.171	0.02905	0.0606	3.74E-08	0.0917297
0.5967	0.7391	0.02608	0.0518	4.14E-09	0.3475
0.2642	0.2596	0.02611	0.0629	6.10E-09	0.753401
0.5685	0.3628	0.0284	0.0575	5.97E-09	0.791701
0.1412	0.09641	0.03802	0.0785	3.53E-15	0.1593
0.5359	0.3413	0.02894	0.0591	1.63E-08	0.2988
0.8222	0.612	0.0369	0.0486	8.13E-22	0.0975911
0.1672	0.1852	0.04636	0.0591	9.11E-10	0.2733
0.0564	0.01053	0.07016	0.2321	1.08E-38	0.244
0.6302	0.729	0.03295	0.051	1.33E-62	0.9652
0.0294	0.05897	0.09922	0.0978	4.44E-08	0.5975
0.0376	0.01079	0.0916	0.2353	3.41E-10	0.1699
0.3057	0.3555	0.02658	0.0475	6.78E-10	0.9956
0.2995	0.4549	0.02681	0.0459	4.70E-11	0.770999
0.567	0.4958	0.02479	0.0457	1.49E-08	0.3331
0.0627	0.05345	0.05783	0.1025	3.67E-08	0.8819
0.2371	0.1065	0.03208	0.074	1.22E-17	0.5083

0.4991	0.2763	0.0281	0.0512	2.21E-33	0.0294503
0.6009	0.4913	0.03056	0.047	9.29E-10	0.0539796
0.1385	0.09641	0.03923	0.0785	1.84E-26	0.1593
0.2578	0.362	0.02741	0.0474	4.29E-26	0.7972
0.2578	0.362	0.0274	0.0474	2.66E-27	0.7972
0.408	0.3711	0.02492	0.0474	5.70E-16	0.0108101
0.0442	0.1363	0.0731	0.07	3.22E-09	0.2089
0.4878	0.3484	0.02494	0.0478	6.58E-11	0.4712
0.4557	0.49	0.02979	0.0487	4.57E-103	0.8913
0.3917	0.3246	0.02512	0.0485	6.86E-10	0.2809
0.4074	0.3711	0.02521	0.0474	4.71E-12	0.01087
0.4557	0.49	0.03043	0.0487	5.06E-81	0.8913
0.4075	0.3711	0.02508	0.0474	5.47E-17	0.01087
0.0442	0.1363	0.07351	0.07	1.89E-08	0.2089
0.4878	0.3484	0.02506	0.0478	5.73E-11	0.4712
0.4557	0.49	0.03019	0.0487	9.25E-90	0.8913
0.3917	0.3246	0.02525	0.0485	8.85E-10	0.2809
0.4462	0.26	0.02584	0.0523	1.05E-84	0.5358
0.051	0.1128	0.06162	0.0732	2.05E-11	0.5532
0.4463	0.26	0.0258	0.0523	3.45E-99	0.5358
0.0512	0.1128	0.06186	0.0732	3.56E-15	0.5532
0.1715	0.0963	0.03657	0.0777	3.70E-08	0.3285
0.4466	0.26	0.02564	0.0523	3.54E-94	0.5358
0.0214	0.03003	0.09617	0.1325	4.79E-08	0.4768
0.472	0.2634	0.02706	0.052	2.49E-14	0.5311
0.4462	0.2595	0.02583	0.0523	1.61E-71	0.6971
0.0512	0.1128	0.0606	0.0732	1.12E-12	0.5532
0.4849	0.283	0.02713	0.0508	1.75E-18	0.8655
0.4461	0.2595	0.02629	0.0523	2.05E-71	0.6974
0.1412	0.09641	0.03725	0.0785	6.09E-43	0.1593
0.7662	0.7338	0.02551	0.0517	1.34E-33	0.0563495
0.4182	0.4821	0.02345	0.0454	1.39E-08	0.1199
0.7662	0.7338	0.02659	0.0517	1.47E-62	0.0563495
0.5089	0.4988	0.02364	0.0455	4.62E-08	0.1597
0.1634	0.06788	0.03176	0.0918	5.96E-09	0.4132
0.7661	0.7338	0.02616	0.0517	7.09E-69	0.0563495
0.0654	0.02075	0.0468	0.1631	3.05E-08	0.4414
0.0236	0.03706	0.07676	0.1246	1.10E-08	0.1748
0.4182	0.4821	0.0249	0.0454	5.15E-10	0.1199
0.7662	0.7338	0.027	0.0517	9.23E-61	0.0563495
0.1634	0.06788	0.03221	0.0918	2.31E-09	0.4132
0.7716	0.6698	0.03334	0.0502	2.25E-09	0.5961
0.5635	0.8162	0.02744	0.0591	7.57E-13	0.0412895
0.0022	0.0583	0.2802	0.0975	2.96E-09	0.8429
0.5639	0.8162	0.02642	0.0591	8.44E-30	0.0412895
0.2239	0.2205	0.03362	0.0605	9.33E-11	0.2408
0.7751	0.7576	0.03424	0.0538	2.62E-13	0.2915
0.3428	0.4487	0.0284	0.049	3.52E-11	0.0111699
0.3428	0.4487	0.02952	0.049	1.16E-09	0.0111699
0.3439	0.4487	0.02987	0.049	2.56E-10	0.0111699
0.2229	0.0221	0.03784	0.1537	6.68E-09	0.805
0.1339	0.09292	0.03733	0.0777	1.90E-08	0.4006
0.3449	0.4487	0.03102	0.049	9.17E-10	0.0111699
0.5292	0.593	0.02705	0.0464	2.99E-09	0.0564196
0.5304	0.593	0.02633	0.0464	2.65E-08	0.0564196
0.7473	0.7576	0.03382	0.0538	2.14E-10	0.2915
0.5305	0.593	0.02676	0.0464	7.66E-12	0.0564196
0.1712	0.05407	0.03596	0.0999	3.17E-08	0.0336001

0.5264	0.3648	0.02725	0.0475	2.34E-08	0.0451398
0.1983	0.124	0.03368	0.069	3.31E-34	0.04134
0.1927	0.1895	0.02878	0.059	8.92E-09	0.3304
0.5065	0.3314	0.02953	0.0523	6.22E-09	0.5031
0.2125	0.0107	0.03403	0.2203	1.38E-16	0.9948
0.2128	0.0107	0.03534	0.2203	6.70E-12	0.9948
0.7665	0.6927	0.02941	0.0497	3.03E-11	0.283
0.3506	0.2164	0.02875	0.0853	3.02E-09	0.530401
0.0562	0.01053	0.05397	0.2321	1.83E-08	0.244
0.6313	0.729	0.02622	0.051	4.98E-33	0.9652
0.0624	0.1244	0.05907	0.0692	1.64E-15	0.407
0.4627	0.5093	0.02782	0.0471	1.13E-28	0.8117
0.2742	0.1558	0.03137	0.0631	1.56E-13	0.6664
0.7759	0.7776	0.02923	0.0549	2.38E-09	0.9043
0.1068	0.03695	0.0402	0.1178	1.11E-25	0.1723
0.7759	0.7776	0.02871	0.0549	3.69E-09	0.9043
0.1075	0.03644	0.03893	0.1185	9.24E-48	0.1517
0.35	0.3621	0.02531	0.0474	9.24E-18	0.6282
0.7758	0.7776	0.02898	0.0549	4.38E-10	0.9043
0.1074	0.03642	0.03994	0.1186	3.54E-18	0.1504
0.4557	0.49	0.03091	0.0487	1.19E-15	0.8913
0.2479	0.1086	0.02726	0.0736	1.56E-113	0.329
0.0704	0.02845	0.04812	0.1391	2.25E-10	0.0954289
0.2479	0.1086	0.02722	0.0736	1.41E-134	0.329
0.0704	0.02845	0.04872	0.1391	4.58E-09	0.0954289
0.2345	0.107	0.02938	0.0741	1.98E-20	0.4584
0.1934	0.3626	0.03655	0.0472	7.84E-184	0.0579002
0.0476	0.02672	0.085	0.1439	1.04E-16	0.3012
0.2613	0.1541	0.04234	0.0635	2.43E-14	0.7535
0.5593	0.548	0.03706	0.0461	1.51E-14	0.5991
0.2131	0.3626	0.03915	0.0472	1.09E-16	0.0579002
0.2177	0.1353	0.03955	0.0669	1.45E-08	0.9208
0.1936	0.3626	0.0362	0.0472	9.19E-189	0.0579002
0.0475	0.02672	0.08485	0.1439	4.46E-17	0.3012
0.2617	0.1541	0.04213	0.0635	1.08E-15	0.7535
0.5596	0.548	0.03701	0.0461	4.12E-15	0.5991
0.2131	0.3626	0.03875	0.0472	4.36E-21	0.0579002
0.2132	0.3626	0.03788	0.0472	1.14E-40	0.0579002
0.1527	0.2218	0.04602	0.0548	3.85E-08	0.615
0.2722	0.1541	0.03696	0.0635	1.37E-13	0.7535
0.1896	0.3626	0.03884	0.0472	1.71E-153	0.0579002
0.5586	0.5454	0.03721	0.0458	2.56E-12	0.666801
0.2604	0.1541	0.04232	0.0635	9.03E-18	0.7535
0.2131	0.3626	0.03917	0.0472	2.13E-21	0.0579002
0.1938	0.3626	0.03627	0.0472	2.80E-187	0.0579002
0.0475	0.02672	0.08484	0.1439	1.33E-16	0.3012
0.2619	0.1541	0.04212	0.0635	2.54E-15	0.7535
0.5592	0.548	0.03696	0.0461	3.54E-15	0.5991
0.2121	0.3626	0.03378	0.0472	1.05E-190	0.0579002
0.571	0.5797	0.03621	0.0462	2.39E-08	0.0691799
0.0483	0.02672	0.08187	0.1439	1.67E-14	0.3012
0.0704	0.004544	0.07202	0.348	1.39E-09	0.5298
0.2708	0.1541	0.04013	0.0635	7.98E-23	0.7535
0.5535	0.548	0.03593	0.0461	6.32E-15	0.5991
0.3642	0.5598	0.02746	0.0462	7.06E-11	0.4838
0.4599	0.3609	0.02612	0.0474	5.33E-11	0.0858697
0.0201	0.003981	0.0895	0.3649	3.38E-08	0.467599
0.5982	0.6163	0.02504	0.0467	2.98E-11	0.4864

0.1075	0.03644	0.03963	0.1185	1.57E-43	0.1517
0.35	0.3621	0.02566	0.0474	2.59E-20	0.6282
0.1975	0.05865	0.03426	0.098	2.41E-16	0.8625
0.1975	0.05865	0.03413	0.098	3.45E-38	0.8625
0.4923	0.3695	0.02869	0.0496	1.99E-10	0.7076
0.476	0.2522	0.02793	0.0607	2.57E-09	0.0960904
0.7366	0.6028	0.02998	0.0465	1.22E-32	0.1681
0.2763	0.2576	0.02969	0.052	4.37E-29	0.559899
0.0676	0.07622	0.0539	0.0856	4.96E-08	0.441199
0.0421	0.03658	0.0624	0.12	1.71E-08	0.7988
0.0201	0.07416	0.08656	0.0868	2.05E-08	0.317
0.0206	0.01612	0.08811	0.1796	3.03E-08	0.8312
0.3347	0.5043	0.02581	0.0457	9.69E-09	0.585901
0.6904	0.6174	0.0265	0.0472	6.59E-17	0.1649
0.0336	0.03387	0.06877	0.1262	3.13E-09	0.7079
0.6349	0.6917	0.02562	0.0496	4.62E-08	0.9113
0.1343	0.09292	0.0371	0.0777	6.13E-11	0.4006
0.134	0.09292	0.0369	0.0777	2.99E-09	0.4006
0.1343	0.09292	0.03346	0.0777	1.35E-09	0.4006
0.6414	0.4146	0.02634	0.0476	3.34E-08	0.1064
0.1019	0.09671	0.03774	0.0779	9.95E-09	0.2688
0.1343	0.09292	0.0371	0.0777	6.13E-11	0.4006
0.1343	0.09292	0.03646	0.0777	4.32E-10	0.4006
0.6455	0.7959	0.0178	0.0567	1.86E-08	0.5921
0.279	0.2623	0.02945	0.0517	3.51E-50	0.7704
0.5251	0.3346	0.03417	0.0482	6.38E-09	0.4292
0.2358	0.1065	0.02846	0.074	1.20E-89	0.5083
0.0532	0.02957	0.05681	0.1333	9.74E-10	0.1277
0.231	0.1065	0.03084	0.074	4.95E-09	0.5083
0.1976	0.1256	0.03389	0.0688	9.69E-44	0.0273899
0.2757	0.05315	0.03381	0.1008	1.24E-11	0.1725
0.2317	0.2843	0.03213	0.0504	1.19E-11	0.6654
0.4483	0.3127	0.02994	0.0523	4.31E-09	0.2841
0.0024	0.008725	0.2843	0.2427	6.79E-09	0.0693697
0.1375	0.2717	0.03497	0.0509	1.57E-13	0.5581
0.3505	0.1401	0.02803	0.0654	7.19E-31	0.2839
0.4673	0.5088	0.02718	0.047	9.48E-10	0.6895
0.028	0.003416	0.08067	0.4415	4.65E-08	0.33
0.0376	0.02796	0.07143	0.1396	3.57E-08	0.7896
0.0825	0.1008	0.04807	0.0764	7.22E-09	0.4647
0.5363	0.587	0.02682	0.0463	9.39E-09	0.0575003
0.7416	0.7576	0.03437	0.0538	1.65E-08	0.2915
0.1973	0.008696	0.03751	0.2457	1.31E-12	0.9981
0.1242	0.1137	0.04145	0.0712	5.39E-10	0.8126
0.0575	0.04058	0.0569	0.1145	1.42E-11	0.2585
0.0542	0.04058	0.05656	0.1145	4.05E-16	0.2585
0.0541	0.04058	0.05805	0.1145	3.41E-08	0.2585
0.0096	0.04919	0.13	0.1046	1.37E-08	0.5615
0.0575	0.04058	0.05531	0.1145	2.38E-16	0.2585
0.7493	0.5448	0.03213	0.0535	1.18E-10	0.8874
0.0543	0.04058	0.05562	0.1145	3.21E-20	0.2585
0.0543	0.04058	0.05509	0.1145	1.64E-26	0.2585
0.1983	0.124	0.03258	0.069	1.13E-27	0.04134
0.1987	0.1243	0.03354	0.069	7.38E-52	0.0439097
0.199	0.1145	0.03289	0.0721	1.61E-55	0.8412
0.1986	0.1243	0.03444	0.069	5.77E-33	0.0439097
0.199	0.1145	0.03405	0.0721	2.26E-26	0.8412
0.0401	0.04908	0.09417	0.1067	4.85E-08	0.2872

0.2819	0.1071	0.03657	0.0745	3.91E-10	0.0312198
0.5733	0.2959	0.03044	0.0526	4.43E-23	0.0462605
0.4356	0.245	0.03213	0.0538	1.68E-14	0.433
0.4356	0.2252	0.03213	0.0681	1.68E-14	0.8549
0.5013	0.5015	0.02928	0.052	5.86E-11	0.3526
0.2429	0.2064	0.03318	0.0563	5.49E-11	0.5054
0.294	0.2197	0.03056	0.0554	1.44E-22	0.8088
0.2426	0.2064	0.03307	0.0563	3.09E-10	0.5054
0.2941	0.2197	0.03052	0.0554	4.45E-20	0.8088
0.2426	0.2064	0.03307	0.0563	2.85E-10	0.5054
0.2941	0.2197	0.03051	0.0554	1.58E-20	0.8088
0.2429	0.2064	0.03318	0.0563	4.68E-11	0.5054
0.294	0.2197	0.03055	0.0554	4.48E-23	0.8088
0.1075	0.03642	0.04016	0.1186	1.78E-13	0.1504
0.4557	0.49	0.03114	0.0487	5.41E-13	0.8913
0.0997	0.246	0.06048	0.0534	4.64E-08	0.6953
0.3413	0.3621	0.0264	0.0474	9.59E-11	0.6282
0.3414	0.3621	0.02618	0.0474	4.31E-12	0.6282
0.1884	0.1099	0.03337	0.0733	2.73E-08	0.9745
0.3009	0.1198	0.02822	0.0692	1.45E-11	0.985
0.1991	0.1915	0.03166	0.0589	1.45E-09	0.3524
0.6743	0.4166	0.02686	0.0463	5.64E-12	0.7959
0.4704	0.5257	0.0256	0.0454	1.82E-11	0.293
0.4699	0.5257	0.02494	0.0454	1.68E-09	0.293
0.1925	0.1867	0.02962	0.0595	7.04E-13	0.2692
0.6737	0.4166	0.02475	0.0463	7.59E-09	0.7959
0.2209	0.2237	0.0375	0.0543	2.20E-19	0.2754
0.5717	0.5607	0.02739	0.0461	8.47E-09	0.5713
0.4558	0.49	0.03076	0.0487	5.00E-58	0.8913
0.2454	0.1086	0.031	0.0736	3.13E-60	0.329
0.5982	0.6163	0.025	0.0467	1.07E-08	0.4864
0.2236	0.3718	0.03922	0.0471	2.63E-15	0.1134
0.1853	0.3718	0.04903	0.0471	1.02E-58	0.1134
0.2544	0.1541	0.048	0.0635	5.38E-09	0.7535
0.5691	0.548	0.04091	0.0461	3.22E-08	0.5991
0.0444	0.03315	0.06109	0.1251	5.45E-09	0.864
0.0444	0.03315	0.06121	0.1251	3.01E-08	0.864
0.0861	0.0685	0.06635	0.0902	1.16E-08	0.137
0.0103	0.04937	0.1252	0.1044	3.01E-09	0.8668
0.1984	0.1743	0.03367	0.0601	2.87E-18	0.9497
0.0987	0.08647	0.04044	0.0813	6.75E-37	0.449
0.02	0.0162	0.08989	0.1796	7.32E-10	0.8315
0.3458	0.05991	0.03245	0.0971	2.76E-09	0.8941
0.3456	0.05991	0.03406	0.0971	2.39E-08	0.8941
0.0049	0.001891	0.1761	0.5838	3.26E-08	0.616
0.0246	0.006545	0.07886	0.2825	2.72E-08	0.7808
0.0609	0.01743	0.05091	0.1765	1.05E-08	0.984
0.0201	0.07416	0.08768	0.0868	3.86E-08	0.317
0.8258	0.7972	0.04677	0.0567	1.85E-21	0.1256
0.8262	0.7972	0.04722	0.0567	5.30E-20	0.1256
0.0194	0.01518	0.09018	0.1876	1.97E-11	0.3459
0.1088	0.01194	0.03854	0.2092	1.41E-25	0.4485
0.3227	0.1244	0.02624	0.0701	1.12E-08	0.401
0.3227	0.1244	0.0265	0.0701	1.25E-08	0.401
0.0957	0.03265	0.03992	0.129	4.71E-08	0.8831
0.3067	0.3289	0.02672	0.0485	3.34E-08	0.5066
0.2342	0.4085	0.03032	0.0464	1.63E-09	0.4784
0.1095	0.01461	0.03859	0.192	2.18E-24	0.8397

	0.1097	0.01461	0.0389	0.192	4.65E-24	0.8397
	0.3642	0.6809	0.03018	0.0497	3.31E-08	0.21
	0.8198	0.8205	0.03212	0.0596	6.36E-19	0.3268
	0.5719	0.5164	0.0375	0.0518	2.93E-48	0.749701
	0.4345	0.245	0.03943	0.0538	3.41E-22	0.433
	0.4345	0.2252	0.03943	0.0681	3.41E-22	0.8549
	0.7326	0.5143	0.03001	0.0541	2.50E-08	0.9887
	0.3593	0.6809	0.02782	0.0497	1.04E-08	0.21
	0.5793	0.5164	0.03137	0.0518	7.44E-18	0.749701
	0.4356	0.245	0.03267	0.0538	3.72E-11	0.433
	0.4356	0.2252	0.03267	0.0681	3.72E-11	0.8549
	0.6292	0.5869	0.03057	0.0534	1.68E-14	0.8292
	0.5737	0.3828	0.03354	0.047	1.59E-08	0.753101

Samplesize.exposur	samplesize.outcome	F-statistics	R2
3113	198014	104.5925184	0.007218
3113	198014	94.433921	0.006517
3113	198014	30.84602475	0.00491
3113	198014	35.88371956	0.005749
3657	198014	30.64807268	0.004015
3657	198014	54.63938178	0.004391
3657	198014	39.95464863	0.004669
3657	198014	90.9964847	0.011852
3657	198014	42.73646692	0.005053
3657	198014	30.15547936	0.002426
3657	198014	54.27525688	0.007186
3408	198014	53.27219661	3.75E-05
3408	198014	50.00102461	5.56E-05
3408	198014	45.38423525	0.000272
3408	198014	159.0620017	3.73E-05
3408	198014	100.3444456	0.000112
3408	198014	49.4320666	8.97E-05
3408	198014	48.86685485	0.000488
3408	198014	34.5030008	0.000344
3408	198014	48.60778296	4.27E-05
3408	198014	51.14818689	4.5E-05
3408	198014	43.07462621	8.82E-05
3408	198014	35.86120495	5.46E-05
3408	198014	37.67441609	4.63E-05
3408	198014	34.53701788	2.02E-05
3408	198014	34.32302826	3.82E-05
3408	198014	52.11492021	5.8E-05
3408	198014	33.70467066	0.000223
3408	198014	65.31664444	5.74E-05
3408	198014	154.8357207	3.63E-05
3408	198014	40.68881556	6.19E-05
3408	198014	46.13017823	4.05E-05
3408	198014	57.32737796	4.03E-05
3408	198014	32.809984	4.03E-05
3408	198014	42.59941271	9.22E-05
3408	198014	60.4420183	5.31E-05
3408	198014	74.31787008	0.0001
3408	198014	36.26893802	8.06E-05
3408	198014	35.00818798	4.51E-05
3408	198014	119.0858147	4.19E-05
3408	198014	65.08014971	1.53E-05
3408	198014	30.45955342	0.000105
3408	198014	35.94585788	4E-05
3408	198014	46.29550326	0.000146
3408	198014	50.19373166	8.83E-06
3408	198014	50.22495274	5.59E-05
3408	198014	47.00867726	4.96E-05
3408	198014	45.41587902	0.000109
3408	198014	41.46012203	5.58E-05
3408	198014	32.99740262	6.56E-05
3408	198014	32.74750242	4.41E-05
3408	198014	56.52719228	5.3E-05
3408	198014	71.12744078	1.25E-05
3408	198014	128.7252327	4.53E-05
3408	198014	117.4900043	4.13E-05
3408	198014	87.29621906	4.61E-05

3408	198014	84.50642673	1.49E-05
3408	198014	144.8908637	5.1E-05
3408	198014	31.60091289	7.94E-05
3408	198014	36.86322629	3.24E-05
3408	198014	58.62371542	7.55E-05
3408	198014	162.4058681	5.72E-05
3408	198014	33.6832437	5.13E-05
3408	198014	45.17285416	3.18E-05
3408	198014	107.9466922	3.8E-05
3408	198014	34.00980931	0.000129
3408	198014	78.69515098	4.61E-05
3408	198014	42.11550467	8.62E-05
3408	198014	47.69939444	0.000167
3408	198014	34.0403562	6.37E-05
3408	198014	58.61529186	9.6E-05
3408	198014	42.29262704	6.93E-05
3408	198014	68.60400581	4.02E-05
3408	198014	43.58720173	3.32E-05
3408	198014	35.53994083	6.65E-05
3408	198014	40.16982551	1.41E-05
3408	198014	45.64258489	5.08E-05
3408	198014	35.78978147	1.89E-05
3408	198014	30.24074664	3.9E-05
3408	198014	97.12761887	0.000199
3408	198014	59.74217878	3.5E-05
3408	198014	30.85597858	2.71E-05
3408	198014	30.0764474	3.7E-05
3408	198014	133.0690351	4.68E-05
3408	198014	98.37947686	5.77E-05
3408	198014	51.84738015	0.000103
3408	198014	30.34254465	4.62E-05
3408	198014	48.7231644	0.000224
3408	198014	42.60660756	5.24E-05
3408	198014	29.92569905	5.6E-05
3408	198014	122.6847632	4.32E-05
3408	198014	30.98815001	0.000143
3408	198014	76.14078502	0.000178
3408	198014	44.88891616	5.78E-05
3408	198014	97.19498449	0.000102
3408	198014	93.76437346	5.5E-05
3408	198014	45.81695081	0.000658
3408	198014	101.0379057	4.15E-05
3408	198014	52.89256198	0.000176
3408	198014	44.01294754	6.44E-05
3408	198014	244.7228227	8.61E-05
3408	198014	207.3626735	0.000158
3408	198014	30.15586082	2.12E-05
3408	198014	32.61948769	6.68E-05
3408	198014	31.56943217	0.000207
3408	198014	38.85781306	0.000414
3408	198014	65.91291693	4.64E-05
3408	198014	187.7038898	6.61E-05
3408	198014	84.89530032	4.48E-05
3408	198014	42.32695809	6.94E-05
3408	198014	43.74841692	5.38E-05
3408	198014	35.8248311	3.36E-05
3408	198014	34.84278549	5.91E-05
3408	198014	208.6491918	3.67E-05

3408	198014	30.81508983	9.71E-05
3408	198014	41.67651673	1.47E-05
3408	198014	30.40073833	6.75E-05
3408	198014	37.67031395	7.49E-05
3408	198014	66.04309672	2.32E-05
3408	198014	87.70702518	1.54E-05
3408	198014	33.06635228	2.91E-05
3408	198014	33.84508369	0.000612
3408	198014	129.5431934	3.04E-05
3408	198014	79.50386764	4.66E-05
3408	198014	51.51665471	2.72E-05
3408	198014	34.40235316	7.04E-05
3408	198014	72.69391627	4.26E-05
3408	198014	40.98045281	9.58E-05
3408	198014	33.05918405	5.03E-05
3408	198014	32.0943604	6.01E-05
3408	198014	83.23893792	5.85E-05
3408	198014	33.59384669	0.000407
3408	198014	38.91453612	5.92E-05
3408	198014	261.0629802	1.53E-05
3408	198014	94.17369792	4.97E-05
3408	198014	30.92461559	5.25E-05
3408	198014	50.18730759	5.59E-05
3408	198014	37.53035834	5.71E-05
3408	198014	46.75177797	1.92E-05
3408	198014	59.55419036	4.54E-05
3408	198014	35.37382775	0.000148
3408	198014	31.80650506	5.21E-05
3408	198014	36.57963416	2.15E-06
3408	198014	57.03374989	7.01E-05
3408	198014	39.32574784	2.31E-05
3408	198014	38.53507259	0.000206
3408	198014	30.26584031	0.000947
3408	198014	65.56032704	2.31E-05
3408	198014	78.88118576	5.55E-05
3408	198014	35.50590335	1.46E-05
3408	198014	57.35638347	1.01E-05
3408	198014	76.04907987	0.000442
3408	198014	32.51752462	0.000118
3408	198014	34.67754937	5.07E-05
3408	198014	33.90003194	0.000403
3408	198014	30.2792797	4.61E-05
3408	198014	31.39106254	5.69E-05
3408	198014	82.89682494	4.86E-05
3408	198014	38.17097297	6.92E-05
3408	198014	40.62671055	0.000217
3408	198014	35.98798298	8.44E-06
3408	198014	82.42728532	0.000288
3408	198014	44.72989294	1.84E-05
3408	198014	30.36603612	2.85E-05
3408	198014	30.37440954	8.87E-05
3408	198014	30.53517496	3.22E-05
3408	198014	188.9611124	4.43E-05
3408	198014	46.18493043	3.52E-05
3408	198014	66.20379238	8.14E-05
3408	198014	41.18394239	0.000135
3408	198014	56.35781023	5.94E-05
3408	198014	55.69793327	5.87E-05

3408	198014	29.91393288	5.6E-05
3408	198014	92.70977332	4.89E-05
3408	198014	140.7941635	5.78E-05
3408	198014	47.88003699	4.49E-05
3408	198014	39.28578744	0.000215
3408	198014	38.130625	0.000175
3408	198014	67.9460662	0.000226
3408	198014	32.03096575	8.23E-05
3408	198014	42.25	4.45E-05
3408	198014	34.69368477	3.05E-05
3408	198014	256.1501393	1.5E-05
3408	198014	46.92688359	1.93E-05
3408	198014	91.76805171	2.15E-05
3408	198014	32.43379115	2.47E-05
3408	198014	50.86784286	6.55E-05
3408	198014	239.149807	4.21E-05
3408	198014	31.84468529	7.45E-05
3408	198014	123.3754673	5.06E-05
3408	198014	31.13847688	5.1E-05
3408	198014	100.8892798	0.00013
3408	198014	39.75342154	4.42E-05
3408	198014	90.42933428	6.89E-05
3408	198014	69.95765035	2.87E-05
3408	198014	83.09039022	7.79E-05
3408	198014	80.07408982	4.23E-05
3408	198014	49.94336072	7.31E-05
3408	198014	53.00830763	5.59E-05
3408	198014	68.34916093	4.81E-05
3408	198014	60.93250167	0.000259
3408	198014	63.66198595	1.12E-05
3408	198014	42.61645027	3.75E-05
3408	198014	41.16818264	3.14E-05
3408	198014	111.9054303	3.94E-05
3408	198014	111.9054303	3.94E-05
3408	198014	55.06503211	4.84E-05
3408	198014	45.01450877	3.17E-05
3408	198014	260.2509652	1.53E-05
3408	198014	66.85310035	0.000354
3408	198014	135.4377115	4.77E-05
3408	198014	37.93915844	6.22E-05
3408	198014	33.34501711	0.000136
3408	198014	55.89380037	6.87E-05
3408	198014	45.16965057	3.97E-05
3408	198014	93.25779748	5.47E-05
3408	198014	95.0065433	3.34E-05
3408	198014	46.22650223	0.000229
3408	198014	43.2187659	5.32E-05
3408	198014	46.60143772	7.09E-05
3408	198014	39.68559181	3.02E-05
3408	198014	34.3494622	6.43E-05
3408	198014	45.25316331	5.04E-05
3408	198014	66.58385066	2.34E-05
3408	198014	157.8201859	5.55E-05
3408	198014	66.79662239	9.78E-05
3408	198014	120.0962647	4.93E-05
3408	198014	51.36590819	2.71E-05
3408	198014	120.0975793	7.05E-06
3408	198014	46.41039207	8.69E-05

3408	198014	43.7851509	1.8E-05
3408	198014	35.73084207	9.19E-05
3408	198014	31.1239066	5.83E-05
3408	198014	59.41632933	4.53E-05
3408	198014	155.376052	3.65E-05
3408	198014	45.98160095	0.000267
3408	198014	285.0021764	5.02E-05
3408	198014	31.29070162	7.32E-05
3408	198014	91.42624144	3.75E-05
3408	198014	51.59290991	2.72E-05
3408	198014	41.70273742	9.75E-05
3408	198014	51.93095305	4.87E-05
3408	198014	49.71077948	0.000191
3408	198014	60.20742541	4.23E-05
3408	198014	57.80502497	0.000222
3408	198014	80.43796836	8.95E-05
3408	198014	40.31907176	6.14E-05
3408	198014	118.0354141	2.08E-05
3408	198014	42.69201417	4.5E-05
3408	198014	97.68165822	5.73E-05
3408	198014	39.71146275	2.1E-05
3408	198014	73.94007375	4.33E-05
3408	198014	30.52600922	6.25E-05
3408	198014	48.09461681	0.000107
3408	198014	45.50051322	5.6E-05
3408	198014	44.97475785	8.94E-05
3408	198014	78.9379356	8.79E-05
3408	198014	56.04462789	8.2E-05
3408	198014	69.97128519	7.38E-05
3408	198014	34.2637459	4.41E-05
3408	198014	30.45138564	1.25E-05
3408	198014	31.70519863	4.08E-05
3408	198014	32.25662603	6.98E-05
3408	198014	43.77514873	1.8E-05
3408	198014	49.55681371	0.000199
3408	198014	34.43905126	4.64E-05
3408	198014	64.06388819	5.63E-05
3408	198014	73.62598174	0.000138
3408	198014	183.7949269	1.08E-05
3408	198014	40.54591673	5.22E-05
3408	198014	36.18688418	3.39E-05
3408	198014	90.77229622	5.32E-05
3408	198014	77.10745649	4.52E-05
3408	198014	70.99982927	2.5E-05
3408	198014	67.64355376	5.95E-05
3408	198014	64.18330678	2.26E-05
3408	198014	75.04045156	5.72E-05
3408	198014	43.03323875	3.03E-05
3408	198014	75.28667755	3.09E-05
3408	198014	58.50929038	1.37E-05
3408	198014	104.3864874	1.84E-05
3408	198014	52.06892798	1.22E-05
3408	198014	32.65306122	3.06E-05
3408	198014	57.25295201	0.000134
3408	198014	57.01078742	0.000384
3408	198014	43.32639	7.35E-05
3408	198014	36.77379323	8.81E-05
3408	198014	63.12261275	1.48E-05

3408	198014	41.78627688	0.000185
3408	198014	30.71801256	0.000217
3408	198014	65.34445795	3.83E-05
3408	198014	35.50669676	6.65E-05
3408	198014	63.62835791	1.49E-05
3408	198014	31.64248367	4.08E-05
3408	198014	31.71245172	0.000175
3408	198014	36.89153978	3.24E-05
3408	198014	65.51340842	4.61E-05
3408	198014	52.10376279	3.06E-06
3408	198014	43.23315111	5.57E-05
3408	198014	37.70710462	6.4E-05
3408	198014	42.97876902	2.27E-05
3408	198014	57.18573614	4.36E-05
3408	198014	64.65887509	2.28E-05
3408	198014	96.68926089	5.1E-05
3408	198014	92.69345825	3.81E-05
3408	198014	71.04049562	1.67E-05
3408	198014	38.48825122	8.33E-05
3408	198014	58.60816691	5.49E-05
3408	198014	84.55176979	1.49E-05
3408	198014	32.16025575	0.000244
3408	198014	34.67151337	2.64E-05
3408	198014	57.23667407	5.37E-05
3408	198014	519.1657263	3.05E-05
3408	198014	39.68151152	0.000183
3408	198014	486.2132213	2.85E-05
3408	198014	32.3337178	5.49E-05
3408	198014	30.18213687	0.000154
3408	198014	61.59048315	1.45E-05
3408	198014	37.22880564	0.000137
3408	198014	44.65660894	2.62E-05
3408	198014	30.77029624	0.000118
3408	198014	79.84157314	5.62E-05
3408	198014	42.75147929	4.51E-05
3408	198014	42.5303377	1.75E-05
3408	198014	54.13283035	4.76E-05
3408	198014	71.0486788	5E-05
3408	198014	82.63009781	7.26E-05
3408	198014	42.82021709	3.76E-05
3408	198014	42.49108368	4.48E-05
3408	198014	59.94236898	5.62E-05
3408	198014	48.33738926	5.1E-05
3408	198014	71.89240924	8E-05
3408	198014	41.50749189	3.89E-05
3408	198014	35.74908995	7.73E-05
3408	198014	70.1256224	7.39E-05
3408	198014	34.49382514	4.65E-05
3408	198014	41.7429729	3.18E-05
3408	198014	32.67965708	0.000179
3408	198014	61.32335892	5.39E-05
3408	198014	33.4971383	0.0002
3408	198014	30.62573082	3.23E-05
3408	198014	58.52426676	0.00033
3408	198014	49.92941521	0.00023
3408	198014	30.58501129	5.19E-05
3408	198014	41.57057543	0.000308
3408	198014	32.09470754	2.45E-05

3408	198014	62.12872182	3.65E-06
3408	198014	34.28814043	4.62E-05
3408	198014	181.1305639	0
3408	198014	44.0181213	4.13E-05
3408	198014	46.50895483	4.36E-05
3408	198014	47.30511031	1.66E-05
3408	198014	69.67332881	0.000118
3408	198014	72.86579072	9.39E-05
3408	198014	265.0781602	9.33E-05
3408	198014	105.8632413	1.86E-05
3408	198014	64.17956503	1.13E-05
3408	198014	33.25430893	0.000103
3408	198014	191.6410434	4.5E-05
3408	198014	176.1968361	4.13E-05
3408	198014	46.93312076	0.0002
3408	198014	34.22732349	0.00023
3408	198014	189.0625	1.11E-05
3408	198014	29.98416159	0.000729
3408	198014	82.21576269	1.93E-05
3408	198014	42.30643156	1.74E-05
3408	198014	39.02198624	9.8E-05
3408	198014	61.30623516	4.31E-05
3408	198014	49.58452052	5.23E-05
3408	198014	36.14309918	0.000336
3408	198014	35.26073234	6.39E-05
3427	198014	31.08719782	0.002783
3427	198014	35.57386141	0.000389
3427	198014	34.06077441	0
2920	198014	124.0624834	0.021181
2920	198014	36.90209094	0.005091
2920	198014	32.74993718	0.002286
2920	198014	59.82025773	0.005623
2920	198014	32.89481481	0.005286
2920	198014	50.71806567	0.008637
2920	198014	55.10517772	0.004182
2920	198014	306.0723007	0.039054
3657	198014	37.19187867	0.002209
3657	198014	31.94706994	0.003748
3657	198014	42.25	0.002656
3657	198014	357.3876396	0.04848
3113	198014	35.02552092	0.001821
3113	198014	32.14281864	0.005106
3595	198014	31.18220375	0.004093
3657	198014	53.24477418	0.002553
3657	198014	34.55362036	0.002836
3657	198014	30.91395842	0.001852
3657	198014	36.1525422	0.003199
3657	198014	125.9252153	0.012651
3657	198014	30.95748683	0.001486
3657	198014	31.86844091	0.003613
3425	198014	30.73334787	7.18E-06
3425	198014	32.06513724	3.55E-05
3425	198014	38.97525826	6.83E-06
3425	198014	34.16900357	4.58E-05
3425	198014	32.19328922	4.13E-05
3425	198014	31.4019485	1.1E-05
3425	198014	46.0473867	1.61E-05
3425	198014	35.71772722	3.33E-05

3425	198014	31.32976368	1.1E-05
3425	198014	31.54391938	7.36E-06
3425	198014	32.16197089	1.88E-06
3425	198014	34.21564645	2E-06
3425	198014	33.97819268	1.98E-06
3425	198014	31.6563007	3.32E-05
3425	198014	30.02439482	1.23E-05
3425	198014	64.11211528	2.24E-05
3425	198014	32.96627824	2.5E-05
3425	198014	54.9027435	1.92E-05
3425	198014	30.6105637	5.36E-06
3425	198014	37.56503642	6.58E-06
3425	198014	33.90585093	1.98E-06
3425	198014	33.46841608	1.95E-06
3425	198014	33.07064498	1.93E-06
3425	198014	32.94792134	1.92E-06
3425	198014	39.35685587	6.89E-06
3425	198014	62.81491561	1.1E-05
3425	198014	33.62949943	1.96E-06
3425	198014	31.55313887	1.1E-05
3425	198014	31.420982	1.83E-05
3425	198014	34.98383628	1.22E-05
3425	198014	29.87743631	4.35E-05
3425	198014	34.3214771	0.000196
3425	198014	75.97068359	1.33E-05
3425	198014	45.72683711	5.06E-05
3425	198014	89.36104942	5.22E-06
3425	198014	35.85532127	0
3425	198014	31.59605613	0.000168
3425	198014	30.43678358	3.37E-05
3425	198014	73.82193369	4.31E-06
3425	198014	34.29011309	2E-06
3425	198014	51.86673198	9.08E-06
3425	198014	46.77647244	2.73E-06
3425	198014	65.09904177	3.8E-06
3425	198014	80.44071838	4.7E-06
3425	198014	79.79629957	4.66E-06
3425	198014	88.00701989	5.14E-06
3425	198014	45.11130301	3.95E-05
3425	198014	34.53822485	2.02E-06
3425	198014	37.7553834	1.32E-05
3425	198014	34.84177312	6.1E-06
3425	198014	75.6404654	0
3425	198014	29.91798627	3.84E-05
3425	198014	39.1706934	1.37E-05
3425	198014	30.11001927	5.27E-06
3425	198014	57.67603216	1.35E-05
3425	198014	36.81659344	2.15E-06
3425	198014	33.52354378	1.96E-06
3669	198014	52.74115108	0.00394
3669	198014	64.34844455	0.008605
3669	198014	63.61109869	0.004752
3669	198014	40.2497611	0.000749
3669	198014	101.4798493	0.01357
3669	198014	52.74115108	0.00394
3669	198014	64.34844455	0.008605
3427	198014	30.06005647	0.003867
2912	198014	32.53341652	0.005567

2912	198014	75.75547732	0.009661
3408	198014	58.16122416	0.007021
3408	198014	273.5104918	0.040097
3408	198014	46.3665047	0.006145
3408	198014	88.04364501	0.002566
3408	198014	2727.040609	0.398476
3408	198014	78.72350247	0.002908
3437	198014	41.44310751	0.004953
3437	198014	256.8810156	0.03734
3437	198014	52.51233427	0.006895
3437	198014	2872.325447	0.416216
3437	198014	90.95578331	0.003331
3437	198014	87.43771766	0.002545
3405	198014	43.2163327	0.00522
3405	198014	206.299587	0.03027
3405	198014	62.84568463	0.006361
3405	198014	1716.997867	0.251141
3405	198014	43.57033329	0.001611
3437	198014	43.19956394	0.005163
3437	198014	202.0532609	0.02937
3437	198014	49.82077387	0.006541
3437	198014	64.88226784	0.001888
3437	198014	2028.321577	0.293908
3437	198014	51.98139664	0.001904
2920	198014	36.05803442	0.005598
2920	198014	72.7108465	0.000914
2920	198014	78.33907391	0.00234
2920	198014	30.10833244	0.001662
2920	198014	350.7757269	0.059887
1837	198014	75.29433828	0.006626
1837	198014	60.45236334	0.002198
1837	198014	40.01010391	0.002136
1837	198014	75.08636892	0.004682
1837	198014	89.90300593	0.011608
1837	198014	40.23304357	0.009983
1837	198014	45.83572856	0.001216
3645	198014	146.3921468	0.006394
3645	198014	128.2427639	0.002468
3645	198014	87.24791692	0.00239
3645	198014	100.7067116	0.003218
3645	198014	66.23155785	0.008356
3645	198014	155.6077644	0.010323
3645	198014	79.67597553	0.00112
3657	198014	125.9043253	0.005492
3657	198014	132.9452577	0.002557
3657	198014	82.82617434	0.002257
3657	198014	42.92341198	0.00515
3657	198014	108.1534797	0.00345
3657	198014	71.05104047	0.008933
3657	198014	146.2474577	0.009664
3657	198014	84.61064588	0.001189
3658	198014	131.584954	0.005744
3658	198014	132.6973742	0.002551
3658	198014	87.51090218	0.002384
3658	198014	39.53954153	0.004742
3658	198014	105.6808165	0.00337
3658	198014	70.84569401	0.008904
3658	198014	147.1503781	0.009727

3658	198014	83.33725967	0.001171
3657	198014	123.319042	0.005379
3657	198014	137.7914793	0.00265
3657	198014	2014.573242	0.098276
3657	198014	88.25702531	0.002405
3657	198014	44.99114201	0.005398
3657	198014	108.8135333	0.003471
3657	198014	71.56245435	0.008997
3657	198014	142.3253638	0.009405
3657	198014	88.01596352	0.001237
3657	198014	125.3941169	0.00547
3657	198014	133.5805244	0.002569
3657	198014	83.38832656	0.002273
3657	198014	47.90529326	0.005747
3657	198014	104.8290829	0.003344
3657	198014	69.29472212	0.008712
3657	198014	144.3460098	0.009539
3657	198014	87.0551361	0.001224
3660	198014	141.5866051	0.006171
3660	198014	129.1037042	0.002481
3660	198014	1953.196067	0.095204
3660	198014	86.36311064	0.002352
3660	198014	40.8310579	0.004897
3660	198014	101.1375803	0.003224
3660	198014	67.80513004	0.008517
3660	198014	151.6002199	0.010016
3660	198014	82.19077648	0.001154
3655	198014	110.3669575	0.004812
3655	198014	105.5038564	0.00203
3655	198014	68.53644819	0.001866
3655	198014	88.68492885	0.002831
3655	198014	66.0468787	0.008307
3655	198014	125.1972857	0.008278
3655	198014	1544.641672	0.075393
3655	198014	72.93680968	0.001026
3656	198014	120.3828665	0.005253
3656	198014	128.0397951	0.002463
3656	198014	70.27197769	0.001919
3656	198014	100.9006318	0.00322
3656	198014	31.72916298	0.003862
3656	198014	74.79552323	0.009406
3656	198014	140.9827139	0.009319
3656	198014	1722.670431	0.084512
3656	198014	80.86142004	0.001137
3596	198014	33.07868864	0.004502
3596	198014	327.9996995	0.032689
3596	198014	33.16036142	0.004351
3596	198014	36.76577731	0.004975
3437	198014	141.3236372	0.007447
3437	198014	259.9544026	0.037787
3437	198014	100	0.00291
3437	198014	2883.162737	0.410293
3437	198014	41.59841495	0.005083
3437	198014	107.6353581	0.003466
3405	198014	114.4437453	0.006088
3405	198014	259.072455	0.038013
3405	198014	32.18495382	0.000564
3405	198014	64.49496718	0.006528

3405	198014	80.46649581	0.01141
3405	198014	34.21713324	0.004659
3405	198014	2201.147156	0.316143
3440	198014	122.8352068	0.006479
3440	198014	278.5726639	0.040458
3440	198014	2580.403851	0.366821
3440	198014	92.88044634	0.013031
3440	198014	60.97695394	0.000732
3440	198014	95.51119896	0.002777
3437	198014	174.6566485	0.009204
3437	198014	344.3274085	0.050051
3437	198014	32.85628745	0.000569
3437	198014	139.3880836	0.004057
3437	198014	67.84225861	0.00914
3437	198014	30.76246147	0.00119
3437	198014	4109.897516	0.584865
3437	198014	135.8162112	0.004374
2920	198014	122.1170126	0.020847
2920	198014	51.06959631	0.008738
2920	198014	35.58739596	0.006084
2919	198014	67.32102698	0.011285
2919	198014	46.01924184	0.00398
2919	198014	530.4447674	0.089824
2919	198014	35.09985919	0.000754
2920	198014	197.5414805	0.033793
2920	198014	68.25725601	0.011269
2920	198014	90.64864283	0.014698
2920	198014	2435.486591	0.40865
2920	198014	300.3033783	0.051372
2920	198014	120.6992512	0.019582
2920	198014	91.96496718	0.015183
2920	198014	34.91902906	0.000494
2920	198014	4980.205431	0.850081
2920	198014	55.7939208	0.001932
2920	198014	214.179197	0.036639
2920	198014	2342.800044	0.39303
2920	198014	100.9188854	0.016373
2920	198014	75.14803436	0.012406
2920	198014	32.50093121	0.002871
2915	198014	50.03352614	0.008574
2915	198014	44.36399475	0.007208
2920	198014	33.52905464	0.005736
2920	198014	44.27809383	0.000557
2920	198014	62.14921701	0.001856
2920	198014	460.6327729	0.078626
2920	198014	115.7569516	0.019414
3440	198014	282.7547654	0.041065
3440	198014	110.1946189	0.003204
3440	198014	57.7307513	0.007047
3440	198014	3350.0734	0.485009
3440	198014	77.44	0.00283
3629	198014	30.37259359	0.001647
3629	198014	397.02981	0.048062
3629	198014	36.62488123	0.000877
3629	198014	458.2675965	0.03149
1837	198014	148.6682463	0.029849
3437	198014	33.68536207	0.003231
3437	198014	66.38771797	0.009337

3437	198014	54.94428442	0.00014
3437	198014	34.60594239	0.000288
3437	198014	29.8835253	0.002867
3437	198014	55.21276301	0.007765
3437	198014	52.92714698	0.000135
3437	198014	32.09000968	0.000267
3435	198014	58.51069979	0.005618
3113	198014	86.63574441	0.01379
3113	198014	57.50405151	0.001155
3113	198014	125.2563841	0.017074
3113	198014	33.53283488	0.001615
3113	198014	41.92776907	0.00033
3113	198014	37.85989918	0.000726
3113	198014	788.4488823	0.049037
1635	198014	42.61088806	0.01289
1635	198014	50.63691235	0.013955
3652	198014	30.6223371	0.000361
3652	198014	97.55492889	0.01023
3657	198014	36.49740343	0.002564
3657	198014	32.15006908	0.004343
3657	198014	62.93420023	0.007551
3657	198014	352.5167266	0.044849
3657	198014	70.28391879	0.009565
3657	198014	128.4811245	0.003727
3657	198014	37.15975748	0.000686
3611	198014	33.82412071	0.004665
3611	198014	42.57236854	0.001656
3611	198014	38.87350896	0.000283
3611	198014	32.41977953	0.000265
3611	198014	266.7687713	0.017016
3611	198014	220.4914438	0.026867
3611	198014	63.34915677	0.001517
3060	198014	36.39024105	0.005055
3060	198014	76.64911138	0.010647
1634	198014	74.53956735	0.015625
1634	198014	36.91602567	0.003767
1634	198014	79.44424555	0.022332
1635	198014	102.4684334	0.02146
1635	198014	31.68963084	0.007889
1635	198014	34.71095691	0.003537
1635	198014	85.05857302	0.023899
3668	198014	37.73469388	0.004945
3668	198014	34.51245283	0.004257
3657	198014	32.55016544	0.002287
3657	198014	46.4398219	0.002728
3657	198014	414.7520363	0.052766
3657	198014	40.57202359	0.003088
3657	198014	158.2132823	0.004589
3657	198014	30.8786049	0.000447
3657	198014	31.87186492	0.000514
3657	198014	41.18760327	0.000761
3657	198014	55.25271778	0.003882
3657	198014	47.93471285	0.002816
3657	198014	33.94207043	0.004585
3657	198014	70.37628995	0.008443
3657	198014	584.0111135	0.0743
3657	198014	62.87975606	0.004786
3657	198014	32.2566665	0.000412

3657	198014	209.2726786	0.006071
3657	198014	55.65422108	0.001028
3657	198014	31.0808373	0.001712
3657	198014	33.69919618	0.004094
3656	198014	32.54072279	0.002286
3656	198014	30.35960744	0.001107
3656	198014	43.66752038	0.002566
3656	198014	35.2580744	0.004232
3656	198014	61.23132827	0.00466
3656	198014	197.9361565	0.005734
3656	198014	366.619781	0.046682
3656	198014	32.34211671	0.000468
3656	198014	31.00143693	0.004207
3656	198014	55.87584472	0.001032
3657	198014	63.41983824	0.004456
3657	198014	31.95006692	0.001165
3657	198014	63.67588511	0.003741
3657	198014	42.36393021	0.005722
3657	198014	123.897598	0.014865
3657	198014	575.8819249	0.073266
3657	198014	90.22992867	0.006868
3657	198014	45.16635581	0.000576
3657	198014	173.338808	0.005028
3657	198014	49.74859523	0.000919
3657	198014	64.0560543	0.004501
3657	198014	50.66338312	0.002976
3657	198014	43.44612535	0.005868
3657	198014	60.11268184	0.007212
3657	198014	731.3735505	0.093048
3657	198014	41.57526198	0.003165
3657	198014	45.99299521	0.000587
3657	198014	156.4996718	0.00454
3657	198014	38.94819185	0.000719
1858	198014	30.95260192	0.000189
3427	198014	30.31081646	0.004276
3427	198014	37.76762793	9.66E-05
3427	198014	53.5560411	0.006525
3405	198014	48.5814221	0.006899
3405	198014	48.39946671	0.000125
3405	198014	68.15103563	0.008361
2919	198014	93.94874805	0.015542
2919	198014	57.37354399	0.000161
2919	198014	43.81600107	9.87E-05
2919	198014	39.9735481	0.000402
3657	198014	32.35814478	0.002775
3657	198014	30.78233742	0.002686
3657	198014	46.59940536	0.002123
3657	198014	97.91310089	0.009836
3657	198014	33.51635536	0.002023
3657	198014	32.44169979	0.00177
3440	198014	31.73833191	1.85E-06
3440	198014	32.56634774	1.89E-06
3440	198014	31.26284224	1.82E-06
3440	198014	31.73129232	1.84E-06
3440	198014	32.40236686	0
3440	198014	32.07556186	1.86E-06
3440	198014	34.82367197	0.000381
3440	198014	33.04615647	0

3440	198014	31.79504683	0
3440	198014	37.2431796	8.66E-06
3440	198014	31.71999747	1.84E-06
3440	198014	32.04274845	1.86E-06
3440	198014	30.15737438	0
3440	198014	30.69659036	1.78E-06
3440	198014	30.03723244	1.75E-06
3440	198014	32.27764373	1.88E-06
3440	198014	33.27328559	1.93E-06
3440	198014	32.04274845	1.86E-06
3440	198014	30.77765427	1.79E-06
3440	198014	32.07912699	1.86E-06
3440	198014	34.04401047	1.98E-06
3440	198014	31.87298727	0
3440	198014	33.2517131	1.93E-06
3440	198014	32.76065631	1.9E-06
3440	198014	30.78088947	1.79E-06
3440	198014	35.84899159	2.08E-06
3440	198014	39.56467012	2.3E-06
3440	198014	38.88490305	0
3440	198014	32.08404344	1.87E-06
3440	198014	32.09168389	1.87E-06
3440	198014	32.16692995	1.87E-06
3440	198014	32.04586589	1.86E-06
3440	198014	31.94189187	1.86E-06
3440	198014	33.29223131	1.94E-06
2915	198014	42.68732161	0.001197
2915	198014	42.77117816	0.003245
2915	198014	63.9899659	0.007728
2915	198014	47.13086919	0.008066
2850	198014	36	0.00631
2850	198014	162.4800564	0.023862
2850	198014	742.1389695	0.040246
2850	198014	36.82066632	0.004095
2850	198014	54.93518799	0.002299
2850	198014	34.67901235	0.000505
3425	198014	31.43081705	1.84E-06
3425	198014	43.11627392	7.55E-06
3425	198014	81.66728223	1.43E-05
3425	198014	45.20059336	2.64E-06
3425	198014	39.96105409	2.33E-06
3425	198014	31.51614325	2.76E-05
3425	198014	36.55823424	8.54E-06
3425	198014	57.41744898	1.34E-05
3425	198014	30.48240704	1.07E-05
3425	198014	36.21746133	9.89E-05
3425	198014	41.49010271	0.004137
3425	198014	35.6043721	0.000149
3425	198014	30.43685483	7.11E-06
3425	198014	33.03304604	5.79E-06
3425	198014	49.45691791	1.15E-05
3425	198014	33.56662234	0.000111
3425	198014	36.78168051	6.44E-06
3425	198014	36.78168051	6.44E-06
3425	198014	44.96628068	7.87E-06
3425	198014	39.17097461	2.06E-05
3425	198014	32.8332548	2.87E-05
3425	198014	32.95076896	5.77E-06

3425	198014	37.68258014	6.6E-06
3425	198014	36.53593778	2.13E-06
3425	198014	36.55132312	2.13E-05
3425	198014	40.76731472	2.38E-06
3425	198014	40.55531124	2.37E-06
3425	198014	36.15575969	2.11E-06
3425	198014	58.76078989	1.03E-05
3425	198014	41.20283609	6.96E-05
3425	198014	40.25816109	7.05E-06
3425	198014	68.78982647	1.2E-05
3425	198014	53.65391471	5.63E-05
3425	198014	36.6780919	6.83E-05
3425	198014	32.39815165	5.67E-06
3425	198014	32.06049062	2.24E-05
3408	198014	31.68762184	1.12E-05
3408	198014	31.85440264	4.66E-05
3408	198014	39.73363724	9.98E-05
3408	198014	33.0625	0.000146
3408	198014	33.62152493	5.92E-06
3408	198014	40.022712	2.11E-05
3408	198014	36.76573349	1.29E-05
3408	198014	48.51223316	1.14E-05
3408	198014	37.23708805	2.19E-06
3408	198014	36.24573308	4.67E-05
3408	198014	35.72231911	2.1E-06
3408	198014	33.77682773	5.94E-06
3408	198014	33.49296349	5.89E-06
3408	198014	38.84516551	2.05E-05
3408	198014	33.62926304	2.96E-05
3408	198014	43.82645274	1.54E-05
3408	198014	42.91640912	2.52E-06
3408	198014	37.34567901	4.81E-05
3408	198014	30.25718058	0.000132
3408	198014	35.80244487	5.24E-05
3408	198014	39.30595532	4.14E-05
3408	198014	45.77717198	3.49E-05
3408	198014	32.04238039	4.13E-05
3408	198014	30.51374623	7.16E-06
3408	198014	45.24574476	1.06E-05
3408	198014	31.36455367	7.36E-06
3408	198014	41.63949073	8.52E-05
3408	198014	31.04599668	5.46E-06
3408	198014	30.48424417	1.79E-05
3408	198014	31.2283737	0
3408	198014	30.25159469	2.66E-05
3408	198014	40.98443249	0.004111
3408	198014	30.3475835	0
3408	198014	91.34093353	2.14E-05
3408	198014	31.74779046	8.9E-05
3408	198014	64.72703534	3.8E-06
3408	198014	63.26673257	1.48E-05
3408	198014	33.06377609	1.36E-05
3408	198014	42.23816112	0.000359
3408	198014	31.49110955	1.85E-06
3408	198014	37.58739799	6.16E-05
3408	198014	39.99859753	8.65E-05
3408	198014	36.98140145	5.41E-05
3408	198014	57.32222569	3.36E-06

3408	198014	43.65629826	1.02E-05
3408	198014	36.0610946	1.9E-05
3408	198014	69.27360947	1.22E-05
3408	198014	32.32342428	1.14E-05
3408	198014	37.24676321	2.18E-05
3408	198014	52.06640969	3.06E-06
3408	198014	32.8848053	5.79E-06
3408	198014	31.63444127	0
3408	198014	34.69565844	5.28E-05
3408	198014	36.52040142	1.5E-05
3408	198014	56.34744476	3.31E-06
3408	198014	49.5909829	0.000165
3408	198014	85.15400692	5E-06
3408	198014	33.97273699	5.98E-06
3408	198014	33.97273699	5.98E-06
3408	198014	29.94630182	0.000244
3408	198014	51.0535168	2.1E-05
3408	198014	82.44118133	1.93E-05
3408	198014	39.48878716	1.62E-05
3408	198014	43.78876947	1.03E-05
3408	198014	45.84776309	2.69E-06
3408	198014	90.83381658	4.79E-05
3408	198014	57.14818939	3.35E-06
3408	198014	31.34249389	0.003328
3408	198014	30.70249933	9.5E-05
3408	198014	34.2079286	1.2E-05
3408	198014	59.7647929	1.05E-05
3408	198014	34.83346986	1.23E-05
3408	198014	31.29149781	5.51E-06
3408	198014	32.92406065	5.79E-06
3408	198014	40.92453424	8.85E-05
3408	198014	54.15704112	2.86E-05
3408	198014	36.01964663	1.48E-05
3408	198014	86.49783359	4.56E-05
3408	198014	31.73172488	1.67E-05
3408	198014	34.86526443	6.14E-06
3408	198014	31.14432388	5.48E-06
3408	198014	29.88444444	1.75E-06
3408	198014	30.84295862	7.24E-06
3408	198014	62.03046085	3.64E-06
3408	198014	31.55079516	5.55E-06
3408	198014	42.2594482	5.69E-05
3408	198014	36.9228204	2.17E-06
3408	198014	34.84245874	1.43E-05
3408	198014	36.36786591	2.13E-06
3408	198014	35.51279183	2.08E-05
3408	198014	32.78797842	1.92E-06
3408	198014	48.70482243	8.57E-06
3408	198014	36.52119281	1.29E-05
3408	198014	40.18024085	1.65E-05
3408	198014	47.41920938	6.11E-05
3408	198014	36.31301242	1.28E-05
3408	198014	44.29232612	7.8E-06
3408	198014	35.22374248	2.07E-06
3408	198014	32.29258861	7.55E-05
3408	198014	43.15813234	1.01E-05
3408	198014	31.09076855	0.000132
3408	198014	53.0481585	9.34E-06

3408	198014	36.66025276	1.5E-05
3408	198014	45.05469526	5.8E-05
3408	198014	49.13542241	5.18E-05
3408	198014	47.02562584	8.8E-05
3408	198014	39.35585108	8.06E-05
3408	198014	31.89810267	2.99E-05
3408	198014	31.53407584	1.11E-05
3408	198014	38.6237423	1.36E-05
3408	198014	48.13939477	0.000202
3408	198014	40.45690627	9.49E-06
3408	198014	49.98991216	3.81E-05
3408	198014	41.57562934	0.000301
3408	198014	30.22747276	5.32E-06
3408	198014	32.5559749	1.91E-05
3408	198014	36.09963095	8.47E-06
3408	198014	30.56281791	7.17E-06
3408	198014	39.35058252	6.93E-06
3408	198014	31.13940579	7.31E-06
3408	198014	33.87418837	6.74E-05
3408	198014	41.21385746	0
3621	198014	35.61505948	0.000646
3621	198014	42.83051451	0.001657
3621	198014	31.05730766	0.000742
3621	198014	431.3609467	0.052384
3621	198014	44.4297872	0.001068
3621	198014	34.02777778	0.00282
3621	198014	145.3345143	0.015957
3621	198014	448.867415	0.030982
3437	198014	186.7052441	0.004262
3437	198014	37.13687551	9.47E-05
3437	198014	114.2615415	0.002041
3437	198014	89.72252078	0.001337
3437	198014	208.5329845	0.007543
3437	198014	33.75365472	0.000402
3437	198014	31.51039685	0.001119
3437	198014	1927.752013	0.274414
3437	198014	127.2020706	0.003275
3440	198014	127.5074073	0.002915
3440	198014	31.52261521	5.3E-05
3440	198014	83.74093214	0.001494
3440	198014	187.736413	0.00481
3440	198014	65.23668639	0.008732
3440	198014	144.9746165	0.005247
3440	198014	2781.667337	0.402738
3440	198014	120.9513075	0.003232
3408	198014	122.3639926	0.002817
3408	198014	90.89155712	0.001637
3408	198014	190.5454468	0.004917
3408	198014	61.08201141	0.00848
3408	198014	148.9856104	0.00545
3408	198014	129.8619869	0.003489
3408	198014	2631.18458	0.384537
3437	198014	163.9832709	0.003743
3437	198014	87.65449294	0.000223
3437	198014	47.36040672	0.000115
3437	198014	94.73055801	0.001692
3437	198014	33.45462279	0.00398
3437	198014	54.64792794	0.006646

3437	198014	54.94215047	0.007362
3437	198014	76.35562101	0.010723
3437	198014	186.8813895	0.00676
3437	198014	54.4908045	0.000908
3437	198014	37.02189079	0.000203
3437	198014	40.43796967	0.001041
3437	198014	1644.189321	0.234049
3437	198014	108.7054393	0.002799
3405	198014	147.4407933	0.003389
3405	198014	75.9590191	0.000195
3405	198014	38.55015714	9.69E-05
3405	198014	30.94758162	0.003724
3405	198014	48.37559238	0.005935
3405	198014	64.93622544	0.000977
3405	198014	37.35979703	0.000451
3405	198014	33.244938	0.000184
3405	198014	35.24044881	0.000916
3405	198014	166.1541735	0.005107
3405	198014	76.69952854	0.002836
3405	198014	97.60729872	0.002516
3440	198014	140.8481174	0.00322
3440	198014	88.82228618	0.001585
3440	198014	179.9105954	0.004609
3440	198014	152.511065	0.00552
3440	198014	68.61215899	0.009427
3440	198014	2653.231801	0.384123
3440	198014	112.2800686	0.003001
2910	198014	52.04366651	0.008926
2910	198014	39.42763728	0.006725
2910	198014	29.96602217	0.005131
2910	198014	32.5509125	0.005588
1555	198014	64.34876035	0.005896
1555	198014	38.71076193	0.002659
1555	198014	51.13889949	0.004468
1555	198014	858.0757066	0.172501
1555	198014	74.58863868	0.018361
1555	198014	48.64919064	0.004111
1555	198014	55.89000092	0.011527
1554	198014	56.2719448	0.005159
1554	198014	48.36152022	0.004233
1554	198014	804.9420844	0.161606
1554	198014	62.46218071	0.015389
1554	198014	55.38717312	0.004671
1554	198014	54.09778765	0.011173
1555	198014	32.56144312	0.002984
1555	198014	241.1005985	0.076921
2910	198014	30.71664503	0.000907
2910	198014	32.38024561	0.003615
2910	198014	595.7529298	0.101165
2910	198014	60.72864044	0.00202
2910	198014	33.33873584	0.003811
2910	198014	51.61151585	0.003361
2910	198014	37.56358	0.000742
3657	198014	30.85639171	0.002693
3657	198014	40.59859451	0.001936
3657	198014	126.0584741	0.012664
3657	198014	40.96	0.001966
3657	198014	30.75451143	0.001847

3657	198014	38.48896295	0.004363
3657	198014	41.29916576	0.003391
2920	198014	30.93763238	0.002688
2920	198014	67.9260836	0.001658
2920	198014	95.61673152	0.002856
2920	198014	79.68697898	0.009202
2920	198014	883.4241744	0.150827
2920	198014	59.86539603	0.002157
3427	198014	31.09391753	0.000343
3427	198014	30.65160774	0.001621
3395	198014	29.9681515	0.000334
3395	198014	31.80806706	0.00057
3395	198014	34.19177845	0.003205
3395	198014	35.45745872	0.00459
3060	198014	62.15920893	0.006766
3060	198014	30.3051169	0.000686
3060	198014	32.90643877	0.00069
3060	198014	33.96911763	0.004858
3060	198014	48.1799308	0.002363
3060	198014	196.5413569	0.019578
3060	198014	222.9190863	0.009847
3060	198014	305.4000105	0.046264
3060	198014	160.4548957	0.025782
3060	198014	160.4548957	0.025782
3427	198014	34.92761624	0.00339
3427	198014	34.47620977	0.003079
3113	198014	89.17196472	0.012155
3113	198014	62.88069696	0.001084
3113	198014	332.9867966	0.020374
3113	198014	41.31998326	0.000983
3657	198014	31.90656923	0.001666
3657	198014	36.78831895	0.001782
3657	198014	38.16255981	0.002303
3657	198014	68.15318953	0.006647
3657	198014	39.50616841	0.000426
3657	198014	37.12443754	0.000369
3657	198014	32.4210232	0.000342
3657	198014	36.47910148	0.000393
3657	198014	43.29619259	0.005628
3656	198014	31.97652426	0.002903
3656	198014	38.69546459	0.001044
3656	198014	51.97800482	0.005463
3656	198014	412.9811749	0.056039
3656	198014	46.26846852	0.005654
3019	198014	33.57128133	0.005521
3019	198014	62.58146825	0.00831
3437	198014	71.9067429	0.000183
3437	198014	32.62933511	0.003968
3437	198014	47.44735745	9.63E-05
3437	198014	34.32658684	1.2E-05
3437	198014	31.06615223	1.08E-05
3437	198014	55.16751387	0.001648
3437	198014	30.34742345	0.002282
2920	198014	32.9556003	0.002521
2920	198014	315.1562098	0.034408
3408	198014	31.02114088	5.46E-06
3408	198014	50.06711934	0.000129
3408	198014	35.23443463	8.85E-05

3408	198014	31.37840106	0
3408	198014	44.60839553	0.00547
3408	198014	31.04652665	0.003561
3408	198014	31.15154036	5.48E-06
3622	198014	108.9755184	0.004215
3622	198014	58.88954914	0.000427
3622	198014	32.82314712	0.000825
3622	198014	473.5083518	0.030092
3622	198014	274.7805828	0.033333
3622	198014	103.9742598	0.002473
3622	198014	36.06078513	0.000319
3622	198014	30.16052042	0.000181
3622	198014	81.24756154	0.003143
3622	198014	55.55919926	0.000403
3622	198014	29.955942	0.000753
3622	198014	393.724724	0.025022
3622	198014	227.1257463	0.027552
3622	198014	87.78423063	0.002088
3622	198014	30.47490629	0.000183
3435	198014	40.86371257	0.000104
3435	198014	33.4718034	8.54E-05
2872	198014	39.22095379	0.004149
2872	198014	36.56367707	0.00452
2872	198014	30.95092048	0.000558
2872	198014	47.67834134	0.002557
2872	198014	49.55847405	0.002412
2872	198014	32.55953381	0.000923
3427	198014	44.81134416	0.002534
3427	198014	31.64786666	0.002359
3427	198014	32.69792973	0.003983
3427	198014	41.44022513	0.002343
3113	198014	36.28738512	0.002242
3657	198014	30.32436998	0.002326
3395	198014	33.53815939	6.3E-05
3405	198014	38.56317273	7.23E-05
3405	198014	30.16796614	6E-05
3427	198014	35.48769687	0.000888
3427	198014	43.77847962	0.000772
3427	198014	43.44888584	0.003938
3648	198014	111.5961897	0.005497
3657	198014	53.92172705	0.000535
3657	198014	46.49173471	0.000491
3657	198014	52.42256487	0.000565
3658	198014	30.90462215	0.000307
3658	198014	34.06383196	0.000367
3657	198014	40.16635538	0.000399
3657	198014	35.83492977	0.000378
3657	198014	37.87608567	0.000408
1833	198014	38.32250372	0.00088
1833	198014	32.1600647	0.000768
1833	198014	42.47330684	0.000931
3656	198014	33.86189779	0.000336
3656	198014	34.8907009	0.000376
3656	198014	30.40561563	0.000951
3656	198014	34.65335745	0.000344
3656	198014	30.30850616	0.00032
3656	198014	40.67749946	0.000438
3656	198014	36.24531445	0.00036

3656	198014	33.50132428	0.004356
3656	198014	40.41386901	0.000435
1857	198014	31.02037771	0.000994
1857	198014	59.62903811	0.01603
1635	198014	31.35385021	0.003338
1635	198014	292.1720918	0.037795
1635	198014	101.9340607	0.025731
3425	198014	38.24091433	6.7E-06
3425	198014	37.9914446	0.003788
3425	198014	30.49771984	1.6E-05
3425	198014	32.94711459	0.003481
3425	198014	32.55087816	0.004339
3425	198014	32.32938241	1.89E-05
3425	198014	30.78786318	0.004464
3425	198014	36.00781038	4.62E-05
3425	198014	30.55313305	5.16E-05
1857	198014	33.16379059	5.71E-05
1857	198014	158.117109	0.028566
1857	198014	56.32812887	0.003808
1856	198014	33.24824402	5.72E-05
1856	198014	57.45275572	0.003886
2919	198014	41.65780408	0.000485
2919	198014	591.1685578	0.100107
2919	198014	32.4716155	0.001081
2919	198014	37.94747915	0.000442
2919	198014	54.2613999	0.004693
2919	198014	636.3192395	0.107753
2919	198014	36.79066801	0.00079
2919	198014	34.87320266	0.000406
2919	198014	53.24438477	0.004605
2919	198014	606.5205727	0.102707
2919	198014	41.32892722	0.000888
2919	198014	41.37250595	0.000482
2919	198014	640.1877815	0.108408
2919	198014	40.32141653	0.000866
3395	198014	32.24515691	0.003986
3395	198014	37.1563836	0.003838
3654	198014	54.70876714	0.00034
3654	198014	41.20109732	0.000633
3654	198014	142.0089446	0.001479
3654	198014	51.68010058	0.000882
3656	198014	33.44646609	0.000208
3656	198014	75.83159726	0.000789
3657	198014	59.33162893	0.000369
3657	198014	60.47618919	0.001028
3657	198014	32.42507852	0.003169
3657	198014	38.79127083	0.000593
3657	198014	140.8691288	0.001466
3656	198014	43.27155678	0.000269
3656	198014	96.41793732	0.001003
3656	198014	36.52575652	0.000621
3657	198014	58.01404363	0.000361
3657	198014	112.8113297	0.001174
3657	198014	46.7899609	0.000795
3618	198014	62.03731447	0.004814
3618	198014	31.38166369	0.000668
3629	198014	292.4143073	0.022887
3629	198014	32.2624	0.001324

3629	198014	204.8149882	0.016031
3614	198014	77.72652961	0.006066
3614	198014	58.65777028	0.003738
3629	198014	302.8104846	0.023701
3629	198014	31.8739177	0.001308
3629	198014	195.5620602	0.015307
3629	198014	34.21443287	0.003167
3668	198014	30.50457997	0.003948
3668	198014	53.17898547	0.001494
3668	198014	93.65573188	0.001366
3668	198014	68.18565885	0.003544
3668	198014	84.65131475	0.001679
3668	198014	31.14888487	0.004031
3668	198014	50.60804799	0.001422
3668	198014	87.3775586	0.001274
3668	198014	66.27126157	0.003444
3668	198014	81.72115351	0.001621
3668	198014	34.03962074	0.004553
3649	198014	41.32153421	0.00254
3649	198014	84.8626239	0.004455
3668	198014	43.59510308	0.001225
3668	198014	79.78269479	0.001163
3668	198014	49.11170634	0.004146
3668	198014	93.40319135	0.004854
3668	198014	72.50660795	0.001438
3668	198014	46.17797519	0.001297
3668	198014	86.16325734	0.001256
3668	198014	52.1222516	0.0044
3668	198014	95.56059975	0.004966
3668	198014	75.56775593	0.001499
3669	198014	31.41413548	0.000882
3669	198014	53.45008919	0.000779
3669	198014	30.35238942	0.002262
3669	198014	111.9356562	0.005816
3669	198014	35.14458501	0.004787
3669	198014	36.36872783	0.000719
3669	198014	33.18311879	0.004509
3652	198014	34.51207541	0.000979
3652	198014	69.68155719	0.001028
3652	198014	92.92355937	0.004958
3652	198014	64.31945045	0.001284
3669	198014	34.829698	0.000978
3669	198014	61.64751031	0.000899
3669	198014	120.9721887	0.006285
3669	198014	35.18700728	0.004793
3669	198014	40.01796228	0.000791
3652	198014	41.40622071	0.001175
3652	198014	78.25804307	0.001154
3652	198014	67.62540179	0.003536
3652	198014	73.97065054	0.001477
3652	198014	35.95113675	0.00483
3112	198014	110.6941083	0.014925
3112	198014	561.4210428	0.027912
3112	198014	48.09687134	0.004886
3112	198014	43.69023464	0.001645
3410	198014	30.38057218	0.000653
3410	198014	60.83157039	0.005886
3410	198014	839.3804366	0.090989

3410	198014	48.06401391	0.001806
2920	198014	53.11169923	0.007996
2920	198014	47.75236602	0.005654
2920	198014	54.27610201	0.006478
3113	198014	32.25415526	0.000458
3437	198014	75.91875166	0.000194
3437	198014	43.29382805	0.005265
3437	198014	43.8630976	8.9E-05
3437	198014	34.37352117	1.2E-05
3437	198014	30.20918342	0.002272
3437	198014	64.40188284	0.004939
3437	198014	76.39905943	0.000195
3437	198014	45.59893384	9.25E-05
3437	198014	76.07461351	0.006697
3437	198014	141.7331302	0.020549
2903	198014	34.70493089	0.000107
2903	198014	55.76775696	0.008688
3437	198014	100.2256062	0.000255
3437	198014	59.24232898	0.000144
3437	198014	32.29765071	0.001816
3437	198014	147.0692577	0.021323
3440	198014	44.13764713	0.000112
3440	198014	45.90101482	0.00453
3440	198014	42.94942451	0.000109
3437	198014	40.14073821	0.000102
3437	198014	47.99525383	0.004225
3437	198014	70.97858342	0.010291
3405	198014	37.20179559	9.57E-05
3405	198014	41.0594778	8.17E-05
3405	198014	33.45531784	0.001007
3437	198014	119.9044754	0.000306
3437	198014	76.32700556	0.000155
3437	198014	31.97651548	0.001798
3437	198014	34.58245812	9.41E-05
3437	198014	31.1751964	0.000171
3437	198014	169.9235754	0.024636
3437	198014	39.11125724	0.000559
3437	198014	103.6216665	0.000264
3437	198014	59.43402036	0.000145
3437	198014	31.006265	0.001744
3437	198014	146.1799522	0.021194
2910	198014	53.26707053	0.000164
2910	198014	33.99794848	8.38E-05
2910	198014	102.6250846	0.002546
2910	198014	52.22146348	0.000861
2910	198014	234.4933269	0.024512
2910	198014	37.23340345	0.00154
2910	198014	36.64161691	0.001616
2910	198014	34.72717307	0.004532
3437	198014	123.3979381	0.000315
3437	198014	75.68409036	0.000154
3437	198014	31.51421545	0.001772
3437	198014	33.00021243	8.98E-05
3437	198014	30.80522416	0.000372
3437	198014	171.1310097	0.024811
3437	198014	38.5611683	0.000551
3437	198014	31.82117028	8.11E-05
3437	198014	108.3375421	0.003237

3408	198014	44.31304926	0.000114
3408	198014	34.92252582	6.94E-05
3405	198014	38.56320094	9.92E-05
3405	198014	37.65848798	7.5E-05
3405	198014	35.90797657	0.001081
3440	198014	45.98846272	0.000117
3440	198014	32.2626601	9.15E-05
3440	198014	55.82077608	0.000142
3440	198014	41.58642122	0.000101
3440	198014	45.0346005	0.005474
3440	198014	40.50440085	0.004332
3440	198014	35.15755208	0.001385
3408	198014	43.26255675	0.000111
3408	198014	83.99852376	0.000157
3440	198014	49.48342547	0.000126
3440	198014	38.55904928	9.38E-05
3440	198014	34.92523828	0.004245
3440	198014	35.47709496	0.00383
3596	198014	64.4585298	0.005897
3596	198014	30.0633853	0.004105
3596	198014	610.2967419	0.0632
3596	198014	69.95142062	0.006593
3596	198014	29.85888506	0.004152
3596	198014	33.80313425	0.004609
3617	198014	96.37607791	0.008875
3617	198014	30.42857565	0.000654
3617	198014	44.73263118	0.000717
3617	198014	1085.210784	0.11118
3617	198014	59.20797921	0.002137
2971	198014	53.80980266	0.006007
2971	198014	39.08518109	0.006451
2971	198014	352.5910752	0.043318
3387	198014	70.38529255	0.006854
3387	198014	34.0322894	0.000191
3387	198014	756.8031176	0.08264
3387	198014	49.81064674	0.001879
3410	198014	39.91619268	0.003862
3410	198014	437.6493727	0.047441
3410	198014	40.45577285	0.004616
3580	198014	70.02878984	0.006431
3580	198014	674.5697421	0.070149
3580	198014	37.2428069	0.001329
3387	198014	86.43188477	0.008417
3387	198014	34.97825796	0.000196
3387	198014	1170.601	0.127825
3387	198014	66.92783188	0.002525
3596	198014	73.30621315	0.006707
3596	198014	35.76646186	0.004884
3596	198014	703.4541643	0.072847
3410	198014	38.81548007	0.003756
3410	198014	475.4527067	0.051539
3387	198014	64.51062987	0.006282
3387	198014	33.39641815	0.000187
3387	198014	843.3966782	0.092096
3387	198014	49.63412296	0.001873
3387	198014	72.55187811	0.007065
3387	198014	39.71030958	0.000218
3387	198014	32.3913241	0.000801

3387	198014	843.4366386	0.0921
3387	198014	59.42210734	0.002242
3410	198014	67.3825121	0.00652
3410	198014	31.17436338	0.002347
3410	198014	720.3285958	0.078084
3410	198014	48.55064006	0.001824
3410	198014	75.23123517	0.007279
3410	198014	36.8403956	0.000903
3410	198014	37.80881359	0.000209
3410	198014	912.5942126	0.098925
3410	198014	66.14645692	0.002485
3620	198014	138.7313109	0.012756
3620	198014	2685.710746	0.26832
3620	198014	42.16851139	0.002941
3620	198014	64.66213995	0.008612
3620	198014	39.05287099	0.00052
3620	198014	35.21766477	0.000515
3410	198014	85.39963719	0.008263
3410	198014	1201.605313	0.130254
3410	198014	67.36562707	0.002531
3410	198014	30.79333701	0.001774
2971	198014	49.85885027	0.005566
2971	198014	31.08202778	0.00513
2971	198014	325.6341183	0.040006
3387	198014	49.20399012	0.004791
3387	198014	34.21399632	0.004965
3387	198014	547.7306819	0.05981
1858	198014	78.00923916	0.000716
1858	198014	64.15886362	0.000555
3657	198014	41.69800318	0.005623
3657	198014	217.3610439	0.022013
3657	198014	33.2763594	0.003164
3657	198014	38.22399598	0.005154
3657	198014	206.0681054	0.020869
3657	198014	51.93427595	0.007003
3657	198014	241.6715104	0.024475
3657	198014	30.42152273	0.002893
3657	198014	52.16049383	0.007033
3657	198014	235.9861929	0.023899
3657	198014	31.5677791	0.003002
1579	198014	74.12147736	0.005958
1579	198014	234.2502573	0.07263
2872	198014	150.9284397	0.006715
2872	198014	55.73170228	0.004667
2872	198014	744.5801243	0.053687
2872	198014	32.53700423	0.002674
2872	198014	93.46775694	0.016272
2872	198014	176.936437	0.007873
2872	198014	107.0687405	0.018542
2872	198014	308.0722414	0.022312
2872	198014	234.6222639	0.029554
2872	198014	45.36244877	0.003427
2871	198014	190.0358224	0.008458
2871	198014	543.062756	0.039199
2871	198014	62.69466957	0.005729
2871	198014	114.6616934	0.014452
2871	198014	41.39099477	0.003399
2871	198014	109.8193868	0.019125

3113	198014	30.33342014	9.74E-06
3113	198014	30.25212441	0.004392
2912	198014	106.994826	0.013645
2912	198014	48.66984934	0.007569
2912	198014	92.94308729	0.011853
2920	198014	41.06340166	0.005225
2920	198014	37.78338288	0.006422
2920	198014	42.10787425	0.005358
2919	198014	36.10156796	0.004597
2920	198014	43.31681658	0.005512
2920	198014	55.17082819	0.009417
2920	198014	46.76586821	0.008002
2920	198014	59.51583226	0.007574
2911	198014	108.2608077	0.013815
2920	198014	62.68836144	0.007977
2920	198014	56.50035734	0.00719
3595	198014	141.6962656	0.013772
2920	198014	32.4228861	0.00017
3657	198014	33.43205891	0.004346
3657	198014	32.77741889	0.000353
3656	198014	36.76699366	0.004781
3656	198014	37.67685555	0.000406
2910	198014	32.56377317	9.14E-05
2910	198014	51.47203254	0.008836
3656	198014	30.74342798	0.004062
3656	198014	71.86121147	0.008632
3656	198014	35.88438386	0.004741
3656	198014	32.59470878	0.004367
3656	198014	39.95627002	0.005464
3656	198014	34.93514539	0.004766
3656	198014	78.7049414	0.009454
3657	198014	38.69261706	0.005287
3657	198014	53.33101597	0.006406
3656	198014	31.81392956	0.004349
3656	198014	65.40317015	0.007856
3656	198014	33.25163834	0.000357
3621	198014	34.49299159	0.001258
3621	198014	42.15163376	0.001884
3621	198014	67.26502039	0.009096
3621	198014	172.0279353	0.016516
3621	198014	144.4769434	0.018368
1558	198014	44.12755102	0.011983
1558	198014	55.9800691	0.011455
1558	198014	820.0282207	0.158384
1558	198014	40.77083116	0.00279
1558	198014	74.9958469	0.01843
1558	198014	48.82907799	0.004129
3437	198014	31.26678344	7.61E-05
3437	198014	56.49366443	9.51E-05
3437	198014	123.8326097	0.0037
3440	198014	29.87850372	7.27E-05
3440	198014	88.629716	0.000149
3440	198014	35.33870524	0.000426
3656	198014	29.89074711	0.002683
3656	198014	33.35323067	0.004468
2920	198014	33.32603569	0.004858
2920	198014	58.31739122	0.003757
2872	198014	51.75796831	0.000817

2872	198014	35.19654397	0.001206
2872	198014	34.28326465	0.003427
2872	198014	30.48543096	0.005136
3657	198014	55.44315995	0.001004
3657	198014	42.25659032	0.001031
3657	198014	139.0353738	0.005314
3657	198014	84.67696832	0.005629
3657	198014	180.5870326	0.007853
3657	198014	55.6214667	0.001008
3657	198014	39.7882923	0.000971
3657	198014	34.78592253	0.00084
3657	198014	1937.038487	0.094494
3657	198014	144.6241822	0.005527
3657	198014	90.05136465	0.005986
3657	198014	188.4335577	0.008194
3657	198014	52.87487759	0.000958
3657	198014	38.32597041	0.000935
3657	198014	32.32387012	0.000781
3657	198014	153.3842834	0.005862
3657	198014	96.02469665	0.006383
3657	198014	31.68748264	0.003857
3657	198014	204.0998668	0.008875
3657	198014	1828.523441	0.0892
3657	198014	55.87723576	0.001012
3657	198014	34.37209247	0.00083
3657	198014	89.22091697	0.005931
3657	198014	41.51388662	0.005053
3657	198014	179.5432951	0.007808
3657	198014	145.8227667	0.005614
3657	198014	57.03490031	0.001033
3657	198014	38.36881677	0.000936
3657	198014	142.5542865	0.005448
3657	198014	87.83174667	0.005839
3657	198014	180.4876443	0.007849
3657	198014	53.18699356	0.000963
3657	198014	32.2839384	0.00078
3657	198014	99.44087333	0.00661
3657	198014	222.2834234	0.009666
3657	198014	170.5595127	0.006566
3440	198014	36.78042398	0.000568
3440	198014	32.14717638	0.00379
3440	198014	34.83191326	0.002354
3629	198014	32.49933041	0.000308
3629	198014	289.4898921	0.035044
3629	198014	34.53229631	0.002851
3629	198014	394.5116236	0.027109
3659	198014	31.22221326	0.000112
3656	198014	29.90815285	7.82E-05
3657	198014	30.13810587	0.002474
3657	198014	70.88949805	0.007122
3657	198014	30.34638522	0.001456
3657	198014	38.72009844	0.001857
2920	198014	153.2844011	0.016691
2920	198014	120.4557218	0.013081
2650	198014	46.90302085	0.001518
2650	198014	35.16967892	0.006536
2920	198014	30.06648471	0.004985
2920	198014	37.46685213	0.004066

3657	198014	125.778393	0.002419
3657	198014	176.6417579	0.007554
3657	198014	40.0356461	0.001327
3657	198014	30.03916043	0.000644
3657	198014	85.63917463	0.000596
3657	198014	1722.101965	0.08873
2872	198014	38.19840943	0.005101
2872	198014	123.297	0.015843
3113	198014	30.67301442	0.000612
3113	198014	39.19260743	0.000937
3427	198014	69.43014885	0.000117
3427	198014	32.25256138	5.44E-05
3427	198014	30.83543674	5.4E-06
3427	198014	81.33333298	0.000137
3427	198014	31.20419596	5.27E-05
3408	198014	30.40136689	0.001503
3408	198014	35.94287982	0.005271
3669	198014	32.98995477	1.8E-05
3669	198014	32.04651602	0.003795
3669	198014	39.67722754	0.000739
3669	198014	31.91812031	0.002378
3669	198014	64.6077518	0.008639
3669	198014	34.61161166	1.88E-05
3669	198014	33.32726981	0.004542
3669	198014	51.02901957	0.006824
3629	198014	66.92352175	0.002414
3629	198014	46.51603423	0.001882
3629	198014	470.8433345	0.063067
3629	198014	319.9906822	0.043427
3629	198014	319.9906822	0.043427
3629	198014	91.00111005	0.012444
3629	198014	63.32713079	0.002284
3629	198014	47.15889575	0.001908
3629	198014	438.397574	0.058721
3629	198014	297.6122561	0.04039
3629	198014	297.6122561	0.04039
3629	198014	86.38062284	0.011812
1855	198014	42.40339835	0.003055
1855	198014	126.7316741	0.023728
1855	198014	40.65245645	0.010419
1855	198014	114.5951798	0.030184
1855	198014	84.72313207	0.022476
1855	198014	84.72313207	0.022476
3629	198014	31.74421802	0.000742
3629	198014	395.1214019	0.022513
3629	198014	44.44444444	0.006115
2872	198014	41.95555681	0.005566
2872	198014	100.6398122	0.010155
2919	198014	51.76977989	0.004478
2919	198014	664.1087808	0.112459
2919	198014	36.52106245	0.005597
2919	198014	49.97663479	0.004323
2919	198014	572.6852279	0.096977
2919	198014	53.31241987	0.001774
2919	198014	47.20346202	0.007776
3113	198014	30.34575304	0.002434
2919	198014	30.3498482	0.003278
2919	198014	51.67580446	0.00172

2918	198014	43.70879169	0.000454
2918	198014	67.89729982	0.004399
2918	198014	31.36978955	0.005367
2918	198014	31.10051482	0.002295
2918	198014	30.09433034	6.37E-05
3656	198014	69.07732195	0.001994
3113	198014	42.04711914	0.004718
3113	198014	30.08223855	0.002643
3658	198014	49.5264617	0.004002
3658	198014	36.82836044	0.003569
3658	198014	65.4894518	0.008528
3658	198014	32.01842016	0.003785
3658	198014	36.64295344	0.004851
3660	198014	44.43047809	0.003588
3660	198014	52.76513566	0.006873
3660	198014	39.57443625	0.005194
3660	198014	46.40724994	0.004533
3657	198014	31.42216881	0.002964
3657	198014	90.27821604	0.003383
3657	198014	136.1575773	0.017741
3629	198014	31.89471916	0.004385
1464	198014	41.35055798	0.011516
1464	198014	581.0057525	0.131402
1464	198014	48.93861573	0.00317
1464	198014	51.68930337	0.013924
1464	198014	49.80294686	0.016785
3645	198014	37.8922694	0.004943
3645	198014	33.95813096	0.002737
3645	198014	38.08602271	0.001825
3645	198014	44.32301159	0.004311
3060	198014	45.81494644	0.005646
3060	198014	224.5383433	0.0364
2971	198014	145.5669592	0.017243
2971	198014	39.56969696	0.001167
2971	198014	235.3614641	0.028916
2971	198014	37.88600237	0.006327
2971	198014	30.22218178	0.001939
2971	198014	101.4834592	0.016309
3668	198014	30.56782392	0.002911
3668	198014	33.90231768	0.004621
3668	198014	79.28676992	0.007235
2850	198014	64.04504135	0.00121
2850	198014	30.6573038	0.002929
2850	198014	59.38845189	0.00929
2848	198014	38.20219332	0.000723
2848	198014	36.10412142	0.004015
3395	198014	41.69677706	0.005125
3395	198014	35.93064699	0.004917
3427	198014	38.04176243	0.004635
3427	198014	32.54276162	0.003965
3434	198014	41.90227023	0.001599
2920	198014	42.3755758	0.001266
2920	198014	263.1436091	0.04493
3653	198014	40.87698055	0.005584
3669	198014	74.45861195	0.008817
3656	198014	48.4416	0.005819
3656	198014	33.64794265	0.004262
3656	198014	38.36485112	0.000411

3656	198014	56.95867721	0.006842
3656	198014	42.26710057	0.005077
3656	198014	33.30588869	0.004001
3656	198014	31.54768529	0.000338
3656	198014	41.42660108	0.004976
3653	198014	68.93196037	0.008205
3653	198014	40.53805462	0.00534
3656	198014	40.62680927	0.00488
2919	198014	36.7658218	0.004088
2919	198014	671.8213668	0.113765
2919	198014	78.2935697	0.002606
2919	198014	46.30107154	0.00525
3408	198014	30.48284071	0.003627
3408	198014	41.98433706	0.006148
2920	198014	52.05532004	0.00712
2920	198014	42.41509265	0.007185
3427	198014	30.95025095	0.002657
3427	198014	48.7762996	9.08E-05
3427	198014	83.25885585	0.010225
3427	198014	43.47970126	0.001721
3657	198014	41.50122645	0.001013
3657	198014	32.02856164	0.000773
3657	198014	141.00312	0.005389
3657	198014	86.54991798	0.001203
3657	198014	31.35833087	0.003817
3657	198014	167.5934407	0.007288
3657	198014	1761.110546	0.085912
3657	198014	36.05685599	0.00088
3657	198014	30.59125019	0.000739
3657	198014	147.3979636	0.005633
3657	198014	86.1990174	0.001198
3657	198014	30.18465643	0.003674
3657	198014	180.1704531	0.007835
3657	198014	1707.86992	0.083314
2920	198014	43.6504874	0.002929
2920	198014	40.55256481	0.006108
2920	198014	33.24954496	0.004113
3668	198014	31.03254818	0.000516
3579	198014	34.43296823	0.004679
3579	198014	111.0188908	0.01112
2920	198014	204.9948165	0.022381
2920	198014	29.98502324	0.003004
3629	198014	37.53789203	0.004865
3629	198014	37.20099316	0.005114
3629	198014	36.90998033	0.003182
3437	198014	36.12862875	7.33E-05
3437	198014	46.7340929	7.86E-05
3437	198014	33.10934301	0.000473
3636	198014	43.4161523	0.001184
3636	198014	34.60207612	0.003298
3636	198014	31.50339623	0.000734
3636	198014	382.5780558	0.052215
3636	198014	55.69604788	0.005661
3636	198014	39.08465369	0.003684
2920	198014	33.1832394	0.000314
2920	198014	48.04659946	0.000467
2920	198014	34.42939342	0.000185
2920	198014	46.51460687	0.000234

2920	198014	86.15577134	0.001589
2920	198014	85.60725079	0.00929
2920	198014	45.24653931	0.000572
3440	198014	32.24171871	0.000567
3440	198014	43.45929491	0.003873
3440	198014	38.7373015	0.004948
3668	198014	35.57356127	0.000592
3668	198014	58.59298173	0.004946
3668	198014	31.60716234	0.000627
3668	198014	31.29240039	0.00375
3668	198014	62.80568044	0.001045
3668	198014	57.46618106	0.00114
3668	198014	42.1208375	0.000701
3668	198014	63.95839428	0.005399
3668	198014	33.36058264	0.000662
3652	198014	46.92328108	0.000786
3652	198014	44.54220892	0.000889
3668	198014	52.18600032	0.000868
3668	198014	52.26524303	0.001037
2919	198014	30.42041345	0.000636
2919	198014	162.9840284	0.017686
2919	198014	43.16938261	0.001106
3653	198014	40.29913985	0.000675
3653	198014	45.05509518	0.000783
3653	198014	30.95340037	0.004234
3653	198014	109.5411022	0.005853
3656	198014	32.10811056	0.000623
3656	198014	33.9354991	0.001388
1837	198014	33.52185008	0.001674
1837	198014	75.84282207	0.005813
1837	198014	39.39854425	0.001046
1837	198014	108.4588943	0.009545
1837	198014	993.93798	0.09251
1634	198014	86.68469115	0.009685
1634	198014	31.92785016	0.005311
1634	198014	51.6401929	0.015214
1634	198014	32.67521166	0.00988
2871	198014	79.52083394	0.002526
2871	198014	51.02040816	0.008767
2871	198014	80.62576541	0.005256
2871	198014	111.9957098	0.014459
2871	198014	83.20321444	0.002643
2871	198014	65.76495712	0.0113
2871	198014	105.9635066	0.006855
2850	198014	342.8026846	0.011015
2850	198014	30.24484558	0.000334
2850	198014	33.52014915	0.005797
2850	198014	220.0521572	0.014439
2850	198014	67.88394494	0.005045
2850	198014	42.84888153	0.000374
3656	198014	31.5681794	0.000612
3130	198014	33.27073227	0.001678
3130	198014	30.74406454	0.004427
3130	198014	32.45966488	0.000197
3629	198014	87.86376972	0.01035
3629	198014	37.48254487	0.000671
1578	198014	35.38225218	0.002873
1578	198014	40.12552272	0.011044

1578	198014	114.4080753	0.035495
2872	198014	31.86077028	0.001198
2872	198014	32.69847051	0.004212
2872	198014	35.08810013	0.005912
2872	198014	30.12819983	0.000763
3112	198014	32.16197089	0.001115
2871	198014	51.48980789	0.006037
2871	198014	257.5177256	0.043651
2871	198014	51.13471935	0.005996
2871	198014	257.2862832	0.043611
3590	198014	34.34524442	0.003671
3629	198014	47.38847258	0.001918
3629	198014	33.88848848	0.002921
3618	198014	62.3819333	0.007024
3618	198014	112.8497633	0.008175
3618	198014	158.8623075	0.020981
3427	198014	30.01499007	5.59E-05
3427	198014	51.16114894	0.006283
3113	198014	30.04490206	0.000908
3113	198014	30.36616758	0.001876
3658	198014	38.0779298	0.00083
3658	198014	138.8884717	0.007292
3657	198014	52.3687971	0.001137
3657	198014	30.34040308	0.001489
3657	198014	43.6511437	0.004151
3657	198014	195.7633145	0.010272
3657	198014	48.44850798	0.001052
3657	198014	31.35785032	0.001539
3657	198014	41.84775815	0.003979
3657	198014	216.2586244	0.011347
3657	198014	34.00919098	0.004613
3660	198014	47.60191441	0.001035
3660	198014	31.43597817	0.002986
3660	198014	143.690949	0.007546
3657	198014	46.25854129	0.001004
3657	198014	33.89843415	0.000114
3657	198014	182.5623695	0.009579
2971	198014	59.38440393	0.007368
2971	198014	64.82196803	0.004258
2650	198014	29.94190874	0.000938
3659	198014	32.40877113	0.000899
3659	198014	33.75494951	0.004449
3659	198014	170.0596089	0.008299
3622	198014	62.30182774	0.003959
3622	198014	31.95040615	0.001568
3622	198014	30.49017898	0.004114
3657	198014	31.80080148	0.001905
3657	198014	54.82189902	0.005507
3657	198014	37.35119516	0.002237
3657	198014	42.47324928	0.002025
3657	198014	87.11538614	0.008448
3657	198014	33.36018542	0.001678
3437	198014	51.04449143	0.004319
3437	198014	30.63506895	0.001821
3437	198014	34.71731364	0.002938
2910	198014	92.18417787	0.013917
2910	198014	99.94146056	0.002682
2910	198014	30.15469946	0.00183

2910	198014	31.60633551	0.000363
2910	198014	53.70237957	0.008107
2910	198014	31.14706043	0.000836
2920	198014	31.73641073	0.00478
2919	198014	267.4730886	0.045322
2919	198014	35.01483893	0.001165
1858	198014	46.14085235	0.009639
1856	198014	31.56980102	0.00664
1856	198014	51.04211551	0.009214
2912	198014	36.0778739	0.000698
2912	198014	94.17373732	0.009451
2912	198014	56.56285491	0.004632
3130	198014	30.05983093	0.003844
2649	198014	140.0435045	0.026246
2914	198014	174.9639286	0.029798
3060	198014	287.6206054	0.046626
2910	198014	268.1944512	0.045728
2910	198014	34.74677044	0.002267
2903	198014	101.030921	0.017266
2918	198014	244.2668409	0.041543
2919	198014	194.5123393	0.033068
2919	198014	30.35362051	0.001416
1635	198014	38.03431001	0.008839
3395	198014	41.78382729	7.12E-05
3395	198014	32.04590789	0.00426
3405	198014	61.16707033	0.000104
3405	198014	46.06166675	0.004096
3405	198014	85.96480313	0.012578
3427	198014	45.73437498	7.72E-05
3427	198014	32.02132873	0.00467
3427	198014	42.50057518	0.00522
3395	198014	32.65882446	0.004794
3405	198014	95.8774563	0.002887
3427	198014	39.68511206	0.004087
2912	198014	30.32337778	0.000267
2912	198014	31.59919702	0.002531
2912	198014	155.5340259	0.02668
3060	198014	112.890625	0.018298
3653	198014	35.58848436	5.25E-05
3655	198014	38.48346755	0.002991
3655	198014	64.52855935	0.001851
3629	198014	40.37242685	0.001634
3629	198014	31.19613353	0.001262
3629	198014	59.45147867	0.006675
3629	198014	368.180656	0.049316
3629	198014	260.9298109	0.035412
3629	198014	260.9298109	0.035412
3629	198014	88.62113476	0.012119
3629	198014	30.955224	0.001253
3629	198014	55.87367136	0.007612
3629	198014	33.43176087	0.000425
2850	198014	32.10504755	0.001741
2850	198014	36.33468733	0.006372
1858	198014	257.0998112	0.061506
1858	198014	33.8235723	0.001863
1858	198014	80.36207548	0.014888
3618	198014	328.8517521	0.039987
3618	198014	32.92726347	0.000744

3618	198014	48.92110296	0.005366
3618	198014	439.3739858	0.030301
2850	198014	69.16527108	0.010577
2850	198014	131.2840599	0.010637
1634	198014	79.15283286	0.021427
1570	198014	145.0300486	0.040905
1633	198014	44.27234608	0.011985
1633	198014	1150.498521	0.235554
1633	198014	32.61235938	0.009786
1633	198014	69.0347436	0.003894
1633	198014	47.98276381	0.003846
1633	198014	94.81890625	0.022942
1633	198014	66.84229101	0.020234
3590	198014	248.09578	0.030379
3590	198014	70.58125248	0.007716
1858	198014	257.3603368	0.061568
1858	198014	33.42528578	0.001841
1858	198014	77.0513169	0.014274
1858	198014	56.58464399	0.013537
1635	198014	295.0111181	0.079825
3582	198014	84.42208734	0.01037
1838	198014	89.14659723	0.020988
1578	198014	178.1381259	0.049958
3618	198014	30.36749191	0.003693
1634	198014	45.79116965	0.01239
1634	198014	1088.299641	0.222607
1634	198014	73.82069807	0.004153
1634	198014	33.68420499	0.001114
1634	198014	92.14460986	0.022271
1634	198014	69.88978781	0.021141
3590	198014	298.9381265	0.036604
3590	198014	98.6725038	0.010786
3579	198014	323.5244653	0.039787
3579	198014	30.51578041	0.003343
2850	198014	88.1704358	0.013484
2850	198014	76.49579986	0.011698
2850	198014	119.5622795	0.017998
3435	198014	122.0198486	0.003655
3653	198014	34.87534953	0.000656
3653	198014	38.35269893	0.002853
3653	198014	69.90856642	0.009389
3113	198014	47.43282173	0.001049
3113	198014	64.51809998	0.009387
3652	198014	32.43581512	0.000171
3652	198014	75.45557505	0.000786
3652	198014	37.86615159	0.001164
3657	198014	52.64133388	0.006697
3657	198014	42.38992447	0.004149
3657	198014	35.92527246	0.002907
3657	198014	31.17712938	0.001496
3656	198014	393.6408008	0.0501
3656	198014	32.70643769	0.002489
3656	198014	195.6852249	0.005669
3656	198014	52.37912933	0.000968
3656	198014	37.16907986	0.002047
3656	198014	43.42390516	0.000518
3642	198014	30.43261866	0.003885
3642	198014	46.50774634	0.006355

3651	198014	81.26761709	0.010359
3651	198014	62.50020627	0.003263
1247	198014	57.83898802	0.02263
1247	198014	59.75775373	0.02338
1247	198014	64.60030938	0.025275
1247	198014	55.32633046	0.021647
1247	198014	52.2620702	0.020448
1247	198014	52.62495522	0.02059
1635	198014	31.87310073	0.001054
2650	198014	42.40254386	0.000137
2650	198014	30.09970727	0.000899
3374	198014	36.92142871	0.001729
3374	198014	122.4418939	0.016367
3410	198014	36.4749181	0.001694
3410	198014	139.5604962	0.018471
3657	198014	62.72946461	0.008176
3657	198014	31.97541879	0.003101
3657	198014	30.29509876	0.004135
3657	198014	34.20161033	0.004044
3657	198014	33.83973836	0.00448
2920	198014	172.059947	0.02826
2920	198014	46.90760112	0.005121
2920	198014	192.1755063	0.031564
2920	198014	54.68247668	0.00597
3425	198014	34.29657812	2E-05
3425	198014	54.45011707	0.007241
2910	198014	41.36449885	0.004019
2910	198014	790.0657414	0.134167
2910	198014	81.34344577	0.002706
3656	198014	52.71211086	0.003315
3656	198014	33.46845299	0.001266
3656	198014	44.89269622	0.005929
3656	198014	571.4221016	0.077535
1834	198014	37.48324485	0.008912
1834	198014	270.3931769	0.07306
3657	198014	49.0739639	0.003944
3657	198014	67.95285443	0.008851
3657	198014	37.53558808	0.004438
3657	198014	32.46528925	0.004298
3657	198014	111.3865306	0.008952
3657	198014	40.96778448	0.004787
3657	198014	31.21608173	0.003691
3657	198014	30.8200212	0.004188
3657	198014	52.01469238	0.006766
3112	198014	33.90680766	0.004425
3060	198014	33.0044447	0.004202
3060	198014	38.51864303	0.004446
3374	198014	54.32609887	0.007058
2870	198014	35.58916171	0.00312
2870	198014	51.80956983	0.002112
2870	198014	167.6808288	0.021124
3579	198014	30.74124214	0.004175
3579	198014	237.6920967	0.023808
3579	198014	41.44921912	0.003169
3580	198014	40.19006997	0.005458
3580	198014	253.3635483	0.025378
3580	198014	38.36913199	0.002932
1247	198014	53.85997536	0.020924

1247	198014	43.9893072	0.017237
1245	198014	35.89143007	0.014092
1247	198014	42.52861482	0.016462
1247	198014	35.00289482	0.013549
1247	198014	40.72357914	0.015451
3649	198014	49.01672783	0.00645
3649	198014	205.2071422	0.006205
1832	198014	35.60144793	0.009358
1832	198014	135.1525176	0.008542
3653	198014	52.19917421	0.006861
3653	198014	203.3223775	0.006141
3648	198014	50.58540548	0.006658
3648	198014	110.0446203	0.003672
3655	198014	42.17980109	0.005541
3655	198014	184.970979	0.006151
3638	198014	30.34992453	0.004005
3638	198014	72.2884818	0.002419
3654	198014	52.12164057	0.006848
3654	198014	30.27000331	0.003278
3654	198014	283.8096926	0.008542
3652	198014	45.84641954	0.006027
3652	198014	182.6862439	0.005519
3655	198014	45.79649532	0.006016
3655	198014	226.2864993	0.007525
3112	198014	40.52027267	0.006152
3112	198014	180.9931716	0.018365
3656	198014	30.74342798	0.004185
3656	198014	120.0431641	0.015758
3657	198014	36.03932277	0.004905
3657	198014	43.09772158	0.00125
3657	198014	227.3853993	0.028942
1835	198014	30.04358641	0.00578
1835	198014	78.71630934	0.008184
1835	198014	31.9291837	0.008623
2909	198014	31.79942337	0.005002
2909	198014	29.95950021	0.001439
3103	198014	36.30622312	0.005457
1857	198014	147.0912526	0.039543
1857	198014	36.1281822	0.005034
3621	198014	30.98279184	0.003976
3621	198014	388.1110698	0.039772
3112	198014	30.41114347	0.004774
3112	198014	34.73222141	0.005372
3427	198014	33.97938868	0.003855
3060	198014	34.04147243	0.005458
3060	198014	62.59381742	0.004961
2911	198014	32.07457079	0.005481
2911	198014	93.65413738	0.009406
1836	198014	37.89841813	0.005749
1836	198014	177.3725864	0.010283
1836	198014	301.2515952	0.076477
1836	198014	30.20456253	0.000939
1836	198014	39.85799398	0.001571
3618	198014	38.30203523	0.004494
3618	198014	43.53735468	0.005049
3657	198014	32.21329438	0.004325
2872	198014	30.48536874	0.001248
2872	198014	74.07370951	0.009331

2872	198014	148.6490293	0.025879
2910	198014	37.72435694	0.006218
2910	198014	115.6049778	0.00948
3668	198014	113.4096784	0.011832
3668	198014	119.0806649	0.012424
3656	198014	66.16242602	0.008742
3656	198014	35.21635	0.000814
3656	198014	42.87256537	0.00586
3656	198014	495.7685673	0.06727
3656	198014	38.27037862	0.004988
3657	198014	48.13200217	0.006355
3657	198014	382.0656687	0.051828
3656	198014	70.84698979	0.009358
3656	198014	31.73347376	0.000733
3656	198014	43.19416322	0.005904
3656	198014	426.9377085	0.05793
3656	198014	37.73121851	0.004918
2911	198014	406.5279668	0.069018
2911	198014	45.29233207	0.001506
2912	198014	482.977285	0.081972
2912	198014	62.61603462	0.002089
2912	198014	30.48054424	0.002975
2919	198014	456.2989193	0.077269
2919	198014	29.96073954	0.00043
2911	198014	58.68715001	0.010049
2919	198014	338.030219	0.057231
2919	198014	51.07767485	0.0017
2919	198014	77.99630698	0.013348
2912	198014	337.390802	0.057258
3113	198014	194.5744138	0.015159
3595	198014	149.0103035	0.01485
3595	198014	32.36130041	0.00438
3595	198014	289.729305	0.028874
3595	198014	30.00852108	0.004172
3595	198014	34.00329296	0.002586
3596	198014	321.280958	0.032019
3596	198014	30.81673241	0.001048
3596	198014	32.81277791	0.000421
3596	198014	38.84943791	0.005257
3595	198014	280.8727709	0.027992
3595	198014	35.86600508	0.002728
3113	198014	35.94962771	0.00407
2914	198014	51.85679436	0.008754
2914	198014	35.43690878	5.34E-05
2920	198014	131.5284283	0.022154
3395	198014	42.23840056	0.004324
3395	198014	53.90921833	0.005536
3652	198014	44.14760216	0.005447
3668	198014	37.22157272	0.004572
3408	198014	40.20587371	0.005324
3408	198014	33.80205516	0.003436
3408	198014	31.73675838	0.00216
3371	198014	37.71432794	0.005056
3395	198014	35.3816066	0.005193
3653	198014	31.08496573	0.004239
3653	198014	40.56431919	0.004194
3652	198014	47.17574811	0.006435
2920	198014	30.77842795	0.002991

2920	198014	31.36	0.005355
2920	198014	152.6339931	0.01662
3440	198014	33.22853489	0.003005
3668	198014	33.92579237	0.004624
3668	198014	69.01249343	0.006297
3652	198014	47.43599968	0.004352
3657	198014	44.41422555	0.004347
3657	198014	35.33527108	0.0044
3656	198014	31.81152288	0.000923
3656	198014	146.1682844	0.018612
2870	198014	64.17347104	0.002616
2870	198014	126.1786255	0.02186
2870	198014	55.025466	0.007631
3642	198014	35.80321185	0.003419
3642	198014	111.454766	0.005839
3657	198014	34.97912866	0.003326
3657	198014	216.9434637	0.011383
3657	198014	74.45964782	0.009264
3656	198014	39.13801956	0.003724
3656	198014	76.35451675	0.004004
3656	198014	64.68509771	0.008777
3629	198014	551.0265404	0.05662
3629	198014	40.46461586	0.001459
3629	198014	663.7911167	0.068207
3629	198014	34.53250925	0.001245
3410	198014	86.91214762	0.00915
1577	198014	1103.221231	0.218261
1577	198014	70.44146159	0.00405
1577	198014	59.28345318	0.014512
1577	198014	60.22363059	0.018826
1858	198014	70.10586717	0.012654
1858	198014	32.42229826	0.005944
1578	198014	1141.394493	0.225847
1578	198014	72.18489976	0.004139
1578	198014	65.62807388	0.016071
1578	198014	62.88948538	0.019644
1858	198014	90.97467471	0.016421
1857	198014	187.2157909	0.033823
1857	198014	30.48709903	0.004248
1857	198014	55.60252338	0.011863
1569	198014	878.1975169	0.172003
1569	198014	49.76674945	0.015642
1569	198014	75.49113269	0.018533
1858	198014	92.4877251	0.016694
1579	198014	1129.567791	0.223541
1579	198014	69.9364333	0.004008
1579	198014	63.86332091	0.015637
1579	198014	63.18873452	0.019729
1634	198014	1144.914802	0.234187
1634	198014	31.46029547	0.009433
1634	198014	59.9882131	0.003375
1634	198014	37.10470941	0.002972
1634	198014	99.70119539	0.024098
1634	198014	61.95043723	0.01874
2871	198014	42.82477486	0.006908
2871	198014	43.41255989	0.007512
3657	198014	30.61365625	0.00033
3657	198014	44.47995029	0.005847

3657	198014	196.4807385	0.01031
3657	198014	86.24516961	0.010731
2850	198014	68.04082922	0.007568
2850	198014	171.9148936	0.019121
3060	198014	40.73011086	0.006654
3060	198014	35.70843379	0.005821
2872	198014	145.0747129	0.019601
3042	198014	127.9969475	0.016827
3042	198014	29.89391128	0.001239
3657	198014	31.9478576	0.000705
3657	198014	31.58865929	0.00034
3657	198014	30.82637763	0.00034
3651	198014	33.05915836	0.004033
3629	198014	70.43326451	0.008297
3629	198014	35.26739793	0.000631
3657	198014	29.98866213	0.003802
3440	198014	43.07746238	0.002912
3669	198014	35.35253119	0.002236
3440	198014	36.95302739	0.002498
3440	198014	30.63962931	0.004097
3440	198014	33.03051251	0.001757
3440	198014	43.07746238	0.002912
3440	198014	39.2083435	0.00265
3395	198014	31.81466986	0.004289
2848	198014	230.7923245	0.032602
1834	198014	34.05338985	0.009261
3374	198014	428.1671234	0.045735
3374	198014	37.58870557	0.001122
3017	198014	34.40712577	0.004052
2920	198014	198.8524396	0.021595
2920	198014	46.27701421	0.006329
2920	198014	46.37385334	0.005654
2920	198014	34.67376749	0.005874
2971	198014	33.80585994	5.45E-05
3629	198014	54.89636159	0.003588
2872	198014	136.597135	0.021655
2872	198014	37.70628698	0.006536
2872	198014	30.02067306	0.000569
2872	198014	30.5332443	0.000769
2872	198014	33.68683135	0.001776
3653	198014	33.09895673	0.004507
3653	198014	32.02433698	0.00336
3395	198014	50.70533399	0.004731
2911	198014	38.80283045	0.0029
2920	198014	46.02036688	0.001708
3405	198014	66.86589663	0.002013
3162	198014	30.6158714	0.000991
3162	198014	32.41112485	0.000195
2920	198014	68.03043453	0.002525
2920	198014	41.78799576	0.005377
3437	198014	85.93376667	0.002568
3437	198014	115.4776318	0.003451
2920	198014	121.3513904	0.013214
2919	198014	238.8932447	0.026061
2919	198014	257.0323552	0.028072
2920	198014	146.6743236	0.015989
2920	198014	115.0972936	0.012566
1559	198014	30.07096231	0.001485

3595	198014	39.38360428	0.004435
3595	198014	99.27857261	0.013511
3595	198014	59.33746047	0.008116
3595	198014	59.33746047	0.008116
3595	198014	43.13367122	0.005999
2872	198014	43.32639	0.005549
2872	198014	97.14114882	0.014041
2871	198014	39.90327031	0.005108
2871	198014	85.43525377	0.012356
2871	198014	40.05622952	0.005127
2871	198014	87.56444712	0.012664
2872	198014	43.64438079	0.005589
2872	198014	99.54225988	0.014388
3657	198014	54.65538865	0.002868
3657	198014	52.43917807	0.007113
1570	198014	30.17002647	0.00345
3435	198014	42.15157254	0.005517
3434	198014	48.32851955	0.006329
3118	198014	31.06801278	0.003047
3395	198014	45.90518659	0.005689
3408	198014	36.81568697	0.003445
3408	198014	47.79822737	0.00616
3427	198014	45.45709229	0.006609
3405	198014	36.51183048	0.005342
3652	198014	51.90613857	0.004419
3652	198014	33.52293031	0.004036
1858	198014	82.882816	0.015355
3042	198014	33.36021906	0.005371
3642	198014	267.2948609	0.036409
2871	198014	280.9408533	0.036241
3657	198014	32.901696	0.004325
1858	198014	63.60896222	0.011887
1244	198014	290.5901888	0.070528
1244	198014	34.54010851	0.010533
1244	198014	30.97897914	0.012214
3657	198014	34.18870494	0.000793
3657	198014	30.83596685	0.000716
1579	198014	32.88724304	0.003278
3162	198014	35.37969217	0.000228
3112	198014	76.92024541	0.007862
3592	198014	164.7706155	0.008161
3657	198014	38.13464377	0.000409
3668	198014	35.52086534	0.004381
3652	198014	31.28244378	0.003875
3440	198014	30.69826975	8.7E-05
3653	198014	31.0319353	0.000408
3653	198014	32.89724346	0.00103
3657	198014	30.34542185	0.000327
1556	198014	93.23374445	0.017239
1559	198014	86.19639953	0.015878
3658	198014	45.29119849	0.000471
3668	198014	110.9758925	0.005867
3669	198014	32.76515328	0.003904
3669	198014	32.55456034	0.003879
3113	198014	29.98604925	0.001667
3629	198014	30.63821366	0.00359
2920	198014	36.58813466	0.004495
3668	198014	105.3562072	0.005602

3652	198014	103.8408152	0.005554
3652	198014	30.65592528	0.003888
3657	198014	79.8385646	0.00645
1634	198014	227.647744	0.068219
1634	198014	96.63044568	0.029061
1634	198014	96.63044568	0.029061
3113	198014	31.22727834	0.00393
3130	198014	32.95304724	0.004847
3595	198014	74.90496932	0.010156
3595	198014	44.03725974	0.006023
3595	198014	44.03725974	0.006023
3579	198014	59.39634139	0.007744
1858	198014	32.22610157	0.008484

Supplementary Table 6

Id.exposure	Outcome	Exposure	Method	nsnp
GCST90001392	IPF	IgD+ CD38+ B cell Absolute (	IVW-mre	2
GCST90001424	IPF	IgD+ B cell %Lymphocyte	IVW-mre	2
GCST90001426	IPF	IgD- CD38dim B cell %lymph	IVW-mre	2
GCST90001436	IPF	Memory B cell %lymphocyte	IVW-mre	2
GCST90001458	IPF	Myeloid Dendritic Cell Absol	IVW-mre	3
GCST90001465	IPF	CD86+ myeloid Dendritic Cel	IVW-mre	4
GCST90001473	IPF	CD62L- CD86+ myeloid Den	IVW-mre	4
GCST90001474	IPF	Plasmacytoid Dendritic Cell %	IVW-mre	3
GCST90001475	IPF	HLA DR++ monocyte %monc	IVW-mre	4
GCST90001520	IPF	CD33+ HLA DR+ CD14dim A	IVW-mre	2
GCST90001547	IPF	Terminally Differentiated CD	IVW-mre	2
GCST90001554	IPF	Effector Memory CD8+ T cell	IVW-mre	2
GCST90001558	IPF	Terminally Differentiated CD	IVW-mre	4
GCST90001580	IPF	CD14+ CD16+ monocyte Abs	IVW-mre	2
GCST90001585	IPF	CD14+ CD16+ monocyte %m	IVW-mre	2
GCST90001586	IPF	CD14+ CD16- monocyte %m	IVW-mre	2
GCST90001587	IPF	CD16+ monocyte %monocyte	IVW-mre	2
GCST90001588	IPF	T/B cell	IVW-mre	2
GCST90001596	IPF	CD8dim T cell Absolute Coun	IVW-mre	2
GCST90001597	IPF	CD8dim T cell %T cell	IVW-mre	2
GCST90001599	IPF	CD4-CD8- T cell %T cell	IVW-mre	3
GCST90001612	IPF	CD8dim T cell %leukocyte	IVW-mre	2
GCST90001613	IPF	CD4-CD8- T cell %leukocyte	IVW-mre	4
GCST90001614	IPF	CD8+ and CD8dim T cell %le	IVW-mre	2
GCST90001648	IPF	HLA DR+ Natural Killer Abs	IVW-mre	3
GCST90001649	IPF	HLA DR+ Natural Killer %Na	IVW-mre	6
GCST90001650	IPF	HLA DR+ Natural Killer %C	IVW-mre	3
GCST90001690	IPF	CD28+ CD45RA+ CD8+ T ce	IVW-mre	2
GCST90001748	IPF	CD20 on IgD+ CD38- B cell	IVW-mre	4
GCST90001781	IPF	CD25 on IgD+ CD38- naive B	IVW-mre	3
GCST90001783	IPF	CD25 on IgD+ CD38+ B cell	IVW-mre	3
GCST90001827	IPF	IgD on IgD+ B cell	IVW-mre	2
GCST90001833	IPF	CD62L on CD62L+ Dendritic	IVW-mre	2
GCST90001867	IPF	CD3 on CD4+ T cell	IVW-mre	2
GCST90001870	IPF	CD34 on Hematopoietic Stem	IVW-mre	4
GCST90001911	IPF	CD45 on Natural Killer	IVW-mre	2
GCST90001944	IPF	CD123 on plasmacytoid Dend	IVW-mre	2
GCST90001945	IPF	CD123 on CD62L+ plasmacyt	IVW-mre	2
GCST90001947	IPF	CD33 on CD33+ HLA DR+ C	IVW-mre	4
GCST90001950	IPF	CD33 on Granulocytic Myeloi	IVW-mre	3
GCST90001952	IPF	CD33 on Monocytic Myeloid-	IVW-mre	3
GCST90001956	IPF	CD33 on CD33+ HLA DR+	IVW-mre	4
GCST90001957	IPF	CD33 on CD33+ HLA DR+ C	IVW-mre	4
GCST90001959	IPF	CD4 on HLA DR+ CD4+ T ce	IVW-mre	2
GCST90001965	IPF	FSC-A on monocyte	IVW-mre	2
GCST90001970	IPF	FSC-A on HLA DR+ Natural	IVW-mre	3
GCST90002005	IPF	CD16 on CD14+ CD16+ mon	IVW-mre	5
GCST90002014	IPF	CCR2 on CD62L+ myeloid D	IVW-mre	3
GCST90002017	IPF	CCR2 on monocyte	IVW-mre	2
GCST90002037	IPF	CD80 on plasmacytoid Dendri	IVW-mre	2
GCST90002038	IPF	CD80 on CD62L+ plasmacyto	IVW-mre	2
GCST90002077	IPF	SSC-A on HLA DR+ Natural	IVW-mre	3
GCST90001458	IPF	Myeloid Dendritic Cell Absol	MR Egger	3
GCST90001465	IPF	CD86+ myeloid Dendritic Cel	MR Egger	4
GCST90001473	IPF	CD62L- CD86+ myeloid Den	MR Egger	4
GCST90001474	IPF	Plasmacytoid Dendritic Cell %	MR Egger	3

GCST90001475	IPF	HLA DR++ monocyte %monc	MR Egger	4
GCST90001558	IPF	Terminally Differentiated CD8	MR Egger	4
GCST90001599	IPF	CD4-CD8- T cell %T cell	MR Egger	3
GCST90001613	IPF	CD4-CD8- T cell %leukocyte	MR Egger	4
GCST90001648	IPF	HLA DR+ Natural Killer Abso	MR Egger	3
GCST90001649	IPF	HLA DR+ Natural Killer %Na	MR Egger	6
GCST90001650	IPF	HLA DR+ Natural Killer %C	MR Egger	3
GCST90001748	IPF	CD20 on IgD+ CD38- B cell	MR Egger	4
GCST90001781	IPF	CD25 on IgD+ CD38- naive B	MR Egger	3
GCST90001783	IPF	CD25 on IgD+ CD38+ B cell	MR Egger	3
GCST90001870	IPF	CD34 on Hematopoietic Stem	MR Egger	4
GCST90001947	IPF	CD33 on CD33+ HLA DR+ C	MR Egger	4
GCST90001950	IPF	CD33 on Granulocytic Myeloi	MR Egger	3
GCST90001952	IPF	CD33 on Monocytic Myeloid-	MR Egger	3
GCST90001956	IPF	CD33 on CD33+ HLA DR+	MR Egger	4
GCST90001957	IPF	CD33 on CD33+ HLA DR+ C	MR Egger	4
GCST90001970	IPF	FSC-A on HLA DR+ Natural	MR Egger	3
GCST90002005	IPF	CD16 on CD14+ CD16+ mon	MR Egger	5
GCST90002014	IPF	CCR2 on CD62L+ myeloid D	MR Egger	3
GCST90002077	IPF	SSC-A on HLA DR+ Natural	MR Egger	3
GCST90001458	IPF	Myeloid Dendritic Cell Absolu	Simple mode	3
GCST90001465	IPF	CD86+ myeloid Dendritic Cel	Simple mode	4
GCST90001473	IPF	CD62L- CD86+ myeloid Denc	Simple mode	4
GCST90001474	IPF	Plasmacytoid Dendritic Cell %	Simple mode	3
GCST90001475	IPF	HLA DR++ monocyte %monc	Simple mode	4
GCST90001558	IPF	Terminally Differentiated CD8	Simple mode	4
GCST90001599	IPF	CD4-CD8- T cell %T cell	Simple mode	3
GCST90001613	IPF	CD4-CD8- T cell %leukocyte	Simple mode	4
GCST90001648	IPF	HLA DR+ Natural Killer Abso	Simple mode	3
GCST90001649	IPF	HLA DR+ Natural Killer %Na	Simple mode	6
GCST90001650	IPF	HLA DR+ Natural Killer %C	Simple mode	3
GCST90001748	IPF	CD20 on IgD+ CD38- B cell	Simple mode	4
GCST90001781	IPF	CD25 on IgD+ CD38- naive B	Simple mode	3
GCST90001783	IPF	CD25 on IgD+ CD38+ B cell	Simple mode	3
GCST90001870	IPF	CD34 on Hematopoietic Stem	Simple mode	4
GCST90001947	IPF	CD33 on CD33+ HLA DR+ C	Simple mode	4
GCST90001950	IPF	CD33 on Granulocytic Myeloi	Simple mode	3
GCST90001952	IPF	CD33 on Monocytic Myeloid-	Simple mode	3
GCST90001956	IPF	CD33 on CD33+ HLA DR+	Simple mode	4
GCST90001957	IPF	CD33 on CD33+ HLA DR+ C	Simple mode	4
GCST90001970	IPF	FSC-A on HLA DR+ Natural	Simple mode	3
GCST90002005	IPF	CD16 on CD14+ CD16+ mon	Simple mode	5
GCST90002014	IPF	CCR2 on CD62L+ myeloid D	Simple mode	3
GCST90002077	IPF	SSC-A on HLA DR+ Natural	Simple mode	3
GCST90001458	IPF	Myeloid Dendritic Cell Absolu	Weighted median	3
GCST90001465	IPF	CD86+ myeloid Dendritic Cel	Weighted median	4
GCST90001473	IPF	CD62L- CD86+ myeloid Denc	Weighted median	4
GCST90001474	IPF	Plasmacytoid Dendritic Cell %	Weighted median	3
GCST90001475	IPF	HLA DR++ monocyte %monc	Weighted median	4
GCST90001558	IPF	Terminally Differentiated CD8	Weighted median	4
GCST90001599	IPF	CD4-CD8- T cell %T cell	Weighted median	3
GCST90001613	IPF	CD4-CD8- T cell %leukocyte	Weighted median	4
GCST90001648	IPF	HLA DR+ Natural Killer Abso	Weighted median	3
GCST90001649	IPF	HLA DR+ Natural Killer %Na	Weighted median	6
GCST90001650	IPF	HLA DR+ Natural Killer %C	Weighted median	3
GCST90001748	IPF	CD20 on IgD+ CD38- B cell	Weighted median	4
GCST90001781	IPF	CD25 on IgD+ CD38- naive B	Weighted median	3
GCST90001783	IPF	CD25 on IgD+ CD38+ B cell	Weighted median	3

GCST90001870	IPF	CD34 on Hematopoietic Stem	Weighted median	4
GCST90001947	IPF	CD33 on CD33+ HLA DR+ C	Weighted median	4
GCST90001950	IPF	CD33 on Granulocytic Myeloi	Weighted median	3
GCST90001952	IPF	CD33 on Monocytic Myeloid-	Weighted median	3
GCST90001956	IPF	CD33 on CD33+ HLA DR+	Weighted median	4
GCST90001957	IPF	CD33 on CD33+ HLA DR+ C	Weighted median	4
GCST90001970	IPF	FSC-A on HLA DR+ Natural	Weighted median	3
GCST90002005	IPF	CD16 on CD14+ CD16+ mon	Weighted median	5
GCST90002014	IPF	CCR2 on CD62L+ myeloid D	Weighted median	3
GCST90002077	IPF	SSC-A on HLA DR+ Natural	Weighted median	3
GCST90001458	IPF	Myeloid Dendritic Cell Absol	Weighted mode	3
GCST90001465	IPF	CD86+ myeloid Dendritic Cel	Weighted mode	4
GCST90001473	IPF	CD62L- CD86+ myeloid Den	Weighted mode	4
GCST90001474	IPF	Plasmacytoid Dendritic Cell %	Weighted mode	3
GCST90001475	IPF	HLA DR++ monocyte %mon	Weighted mode	4
GCST90001558	IPF	Terminally Differentiated CD	Weighted mode	4
GCST90001599	IPF	CD4-CD8- T cell %T cell	Weighted mode	3
GCST90001613	IPF	CD4-CD8- T cell %leukocyte	Weighted mode	4
GCST90001648	IPF	HLA DR+ Natural Killer Abs	Weighted mode	3
GCST90001649	IPF	HLA DR+ Natural Killer %N	Weighted mode	6
GCST90001650	IPF	HLA DR+ Natural Killer %C	Weighted mode	3
GCST90001748	IPF	CD20 on IgD+ CD38- B cell	Weighted mode	4
GCST90001781	IPF	CD25 on IgD+ CD38- naive B	Weighted mode	3
GCST90001783	IPF	CD25 on IgD+ CD38+ B cell	Weighted mode	3
GCST90001870	IPF	CD34 on Hematopoietic Stem	Weighted mode	4
GCST90001947	IPF	CD33 on CD33+ HLA DR+ C	Weighted mode	4
GCST90001950	IPF	CD33 on Granulocytic Myeloi	Weighted mode	3
GCST90001952	IPF	CD33 on Monocytic Myeloid-	Weighted mode	3
GCST90001956	IPF	CD33 on CD33+ HLA DR+	Weighted mode	4
GCST90001957	IPF	CD33 on CD33+ HLA DR+ C	Weighted mode	4
GCST90001970	IPF	FSC-A on HLA DR+ Natural	Weighted mode	3
GCST90002005	IPF	CD16 on CD14+ CD16+ mon	Weighted mode	5
GCST90002014	IPF	CCR2 on CD62L+ myeloid D	Weighted mode	3
GCST90002077	IPF	SSC-A on HLA DR+ Natural	Weighted mode	3
GCST90001527	IPF	CD33dim HLA DR+ CD11b-	Wald ratio	1

β coefficient (95%CI)	SE	OR (95%CI)	Pval	FDR	Cochrane Q
0.064 (-0.58 - 0.708)	0.328735	1.066 (0.56 - 2.03)	0.845739	0.92677	0.816709349
0.039 (-0.283 - 0.362)	0.164601	1.04 (0.753 - 1.436)	0.810894	0.914413	0.322040208
0.006 (-0.777 - 0.79)	0.399847	1.006 (0.46 - 2.204)	0.987183	0.989795	2.530979115
-0.047 (-0.293 - 0.199)	0.125522	0.955 (0.746 - 1.221)	0.710855	0.914413	0.184662242
0.177 (-0.078 - 0.433)	0.130503	1.194 (0.925 - 1.542)	0.174162	0.48582	3.323853958
0.071 (-0.199 - 0.342)	0.138164	1.074 (0.819 - 1.408)	0.605359	0.914413	8.427659252
0.099 (-0.142 - 0.34)	0.12295	1.105 (0.868 - 1.406)	0.418716	0.853537	8.5512541
-0.095 (-0.467 - 0.278)	0.18999	0.91 (0.627 - 1.32)	0.61769	0.914413	6.009708763
-0.3 (-0.544 - -0.056)	0.124514	0.741 (0.58 - 0.945)	0.015846	0.116356	3.23821602
-0.227 (-0.414 - -0.04)	0.095486	0.797 (0.661 - 0.961)	0.017563	0.116356	0.54307059
-0.793 (-0.924 - -0.663)	0.066545	0.452 (0.397 - 0.515)	8.85E-33	4.69E-31	0.0214184
0.208 (-0.152 - 0.569)	0.183932	1.231 (0.859 - 1.766)	0.258054	0.651278	3.595479249
-0.076 (-0.344 - 0.193)	0.137074	0.927 (0.709 - 1.213)	0.581246	0.914413	2.902758857
-0.06 (-0.465 - 0.346)	0.206975	0.942 (0.628 - 1.413)	0.772571	0.914413	3.445647414
-0.062 (-0.431 - 0.308)	0.188365	0.94 (0.65 - 1.36)	0.743276	0.914413	3.371773615
0.058 (0.012 - 0.103)	0.023296	1.059 (1.012 - 1.109)	0.013234	0.116356	0.018774687
-0.058 (-0.105 - -0.012)	0.023921	0.943 (0.9 - 0.988)	0.014526	0.116356	0.019217548
-0.014 (-0.29 - 0.262)	0.140671	0.986 (0.749 - 1.299)	0.921285	0.976562	0.286243683
-0.088 (-0.482 - 0.305)	0.20081	0.915 (0.618 - 1.357)	0.659705	0.914413	0.5268596
-0.086 (-0.408 - 0.235)	0.164116	0.917 (0.665 - 1.266)	0.599463	0.914413	0.476994511
-0.185 (-0.389 - 0.019)	0.104069	0.831 (0.678 - 1.019)	0.075941	0.268323	0.60681928
-0.087 (-0.431 - 0.256)	0.17545	0.916 (0.65 - 1.292)	0.6183	0.914413	0.487519239
-0.164 (-0.327 - -0.001)	0.083137	0.848 (0.721 - 0.999)	0.047989	0.231156	0.707479337
-0.122 (-0.361 - 0.116)	0.12166	0.885 (0.697 - 1.123)	0.31424	0.724119	0.195986528
-0.109 (-0.469 - 0.252)	0.184104	0.897 (0.625 - 1.287)	0.555457	0.914413	7.052097017
-0.152 (-0.3 - -0.004)	0.075615	0.859 (0.741 - 0.996)	0.044235	0.231156	4.16292978
-0.167 (-0.397 - 0.064)	0.117716	0.847 (0.672 - 1.066)	0.157037	0.462386	3.194486664
0.074 (-0.734 - 0.882)	0.412249	1.077 (0.48 - 2.417)	0.856825	0.92677	2.142684753
-0.003 (-0.523 - 0.516)	0.264899	0.997 (0.593 - 1.675)	0.989795	0.989795	7.239145228
0.096 (-0.128 - 0.32)	0.114166	1.101 (0.88 - 1.377)	0.399755	0.847481	1.540733789
0.086 (-0.186 - 0.358)	0.138978	1.09 (0.83 - 1.431)	0.535887	0.914413	1.762399782
0.047 (-0.333 - 0.428)	0.194171	1.049 (0.717 - 1.534)	0.807153	0.914413	6.137447657
0.317 (0.03 - 0.604)	0.146284	1.373 (1.031 - 1.829)	0.030311	0.178499	0.605842432
0.011 (-0.408 - 0.431)	0.214132	1.011 (0.665 - 1.539)	0.958161	0.989795	1.884457471
0.113 (-0.202 - 0.428)	0.160724	1.119 (0.817 - 1.534)	0.483738	0.914413	6.096637791
-0.05 (-0.437 - 0.337)	0.197536	0.951 (0.646 - 1.401)	0.800176	0.914413	0.952164743
0.028 (-0.171 - 0.227)	0.101397	1.028 (0.843 - 1.254)	0.784016	0.914413	0.467071155
0.028 (-0.172 - 0.229)	0.102217	1.029 (0.842 - 1.257)	0.780805	0.914413	0.466070298
-0.06 (-0.123 - 0.002)	0.031986	0.941 (0.884 - 1.002)	0.059397	0.231156	2.306278773
-0.082 (-0.133 - -0.03)	0.026112	0.922 (0.876 - 0.97)	0.001796	0.024985	0.48957654
-0.071 (-0.111 - -0.031)	0.020445	0.932 (0.895 - 0.97)	0.000521	0.013816	0.55261937
-0.06 (-0.122 - 0.002)	0.031814	0.942 (0.885 - 1.002)	0.059547	0.231156	2.307752821
-0.06 (-0.122 - 0.003)	0.031793	0.942 (0.885 - 1.003)	0.06106	0.231156	2.322506193
-0.151 (-0.814 - 0.513)	0.338571	0.86 (0.443 - 1.67)	0.656465	0.914413	2.428740542
0.351 (-0.181 - 0.883)	0.271532	1.42 (0.834 - 2.419)	0.196169	0.519848	1.263618711
-0.216 (-0.487 - 0.055)	0.138117	0.806 (0.615 - 1.056)	0.117688	0.36691	2.874674843
-0.089 (-0.295 - 0.118)	0.105293	0.915 (0.744 - 1.125)	0.398926	0.847481	7.479765862
-0.024 (-0.151 - 0.103)	0.064566	0.976 (0.86 - 1.108)	0.710154	0.914413	0.870292264
-0.159 (-0.26 - -0.059)	0.051249	0.853 (0.771 - 0.943)	0.001886	0.024985	0.208894115
0.03 (-0.18 - 0.241)	0.10728	1.031 (0.835 - 1.272)	0.776504	0.914413	0.464710624
0.031 (-0.181 - 0.243)	0.108049	1.032 (0.835 - 1.275)	0.77254	0.914413	0.463438584
-0.208 (-0.467 - 0.052)	0.132347	0.813 (0.627 - 1.053)	0.116689	0.36691	2.866052137
0.037 (-0.471 - 0.545)	0.259148	1.037 (0.624 - 1.724)	0.910355	1	2.28854799
0.004 (-0.752 - 0.76)	0.385787	1.004 (0.471 - 2.138)	0.993216	1	8.270719478
-0.079 (-0.589 - 0.431)	0.260196	0.924 (0.555 - 1.538)	0.788941	1	6.493432987
-0.164 (-1.173 - 0.845)	0.514739	0.849 (0.309 - 2.327)	0.803435	1	5.864679349

-0.375 (-2.016 - 1.266)	0.837334	0.687 (0.133 - 3.547)	0.697993	1	3.224921849
-0.209 (-0.663 - 0.246)	0.232049	0.812 (0.515 - 1.279)	0.463573	1	2.27115219
-0.047 (-1.695 - 1.602)	0.841018	0.954 (0.184 - 4.961)	0.964583	1	0.578521568
-0.243 (-1.223 - 0.737)	0.500097	0.784 (0.294 - 2.09)	0.67512	1	0.679534513
0.057 (-1.153 - 1.267)	0.617333	1.059 (0.316 - 3.552)	0.940969	1	6.491152796
-0.121 (-0.405 - 0.162)	0.144803	0.886 (0.667 - 1.176)	0.449208	1	4.093338577
-0.046 (-0.5 - 0.407)	0.231563	0.955 (0.606 - 1.503)	0.8742	1	2.244554219
-3.037 (-5.598 - -0.477)	1.306448	0.048 (0.004 - 0.621)	0.145658	1	1.753050453
0.628 (-0.305 - 1.561)	0.475888	1.874 (0.737 - 4.762)	0.412857	1	0.191127935
0.661 (-0.811 - 2.132)	0.750929	1.936 (0.444 - 8.435)	0.540678	1	1.09366674
1.333 (0.135 - 2.531)	0.611193	3.793 (1.145 - 12.567)	0.160931	1	1.968005401
-0.084 (-0.217 - 0.048)	0.067632	0.919 (0.805 - 1.049)	0.337912	1	2.111248429
-0.107 (-0.306 - 0.093)	0.101701	0.899 (0.736 - 1.097)	0.485038	1	0.406391254
-0.094 (-0.231 - 0.044)	0.070142	0.911 (0.794 - 1.045)	0.409545	1	0.402368384
-0.085 (-0.218 - 0.048)	0.067674	0.919 (0.804 - 1.049)	0.335924	1	2.101337848
-0.084 (-0.217 - 0.049)	0.067783	0.919 (0.805 - 1.05)	0.339906	1	2.121158193
-0.067 (-0.711 - 0.576)	0.328132	0.935 (0.491 - 1.778)	0.870914	1	2.239857914
0.012 (-0.533 - 0.557)	0.277985	1.012 (0.587 - 1.746)	0.967589	1	7.097581468
-0.364 (-1.116 - 0.388)	0.383719	0.695 (0.328 - 1.475)	0.517084	1	0.032212863
-0.06 (-0.707 - 0.587)	0.330154	0.942 (0.493 - 1.799)	0.885704	1	2.26008913
0.179 (-0.202 - 0.56)	0.194478	1.196 (0.817 - 1.751)	0.45473	1	NA
-0.222 (-0.739 - 0.295)	0.263887	0.801 (0.477 - 1.343)	0.461512	1	NA
0.504 (-0.064 - 1.072)	0.289744	1.655 (0.938 - 2.921)	0.180363	1	NA
0.032 (-0.335 - 0.399)	0.187244	1.032 (0.715 - 1.49)	0.881316	1	NA
-0.273 (-0.687 - 0.14)	0.210864	0.761 (0.503 - 1.15)	0.285653	1	NA
-0.003 (-0.426 - 0.42)	0.215774	0.997 (0.653 - 1.521)	0.988814	1	NA
-0.282 (-0.793 - 0.229)	0.260785	0.754 (0.452 - 1.257)	0.39232	1	NA
-0.224 (-0.701 - 0.253)	0.243587	0.799 (0.496 - 1.288)	0.425519	1	NA
-0.252 (-0.643 - 0.138)	0.199446	0.777 (0.526 - 1.148)	0.333074	1	NA
-0.153 (-0.435 - 0.13)	0.144203	0.858 (0.647 - 1.139)	0.337633	1	NA
-0.175 (-0.531 - 0.182)	0.181838	0.84 (0.588 - 1.199)	0.437829	1	NA
0.387 (-0.469 - 1.242)	0.436431	1.472 (0.626 - 3.463)	0.440692	1	NA
0.001 (-0.399 - 0.401)	0.204047	1.001 (0.671 - 1.494)	0.995768	1	NA
0.004 (-0.424 - 0.432)	0.21844	1.004 (0.654 - 1.541)	0.98643	1	NA
-0.026 (-0.449 - 0.398)	0.215908	0.975 (0.638 - 1.488)	0.913285	1	NA
-0.063 (-0.232 - 0.105)	0.08605	0.939 (0.793 - 1.111)	0.515085	1	NA
-0.098 (-0.268 - 0.073)	0.086998	0.907 (0.765 - 1.075)	0.377336	1	NA
-0.077 (-0.218 - 0.065)	0.072	0.926 (0.804 - 1.067)	0.399153	1	NA
-0.064 (-0.22 - 0.092)	0.079386	0.938 (0.803 - 1.096)	0.478662	1	NA
-0.063 (-0.225 - 0.098)	0.082385	0.939 (0.799 - 1.103)	0.497555	1	NA
-0.186 (-0.568 - 0.195)	0.194516	0.83 (0.567 - 1.215)	0.439258	1	NA
0.012 (-0.364 - 0.387)	0.191702	1.012 (0.695 - 1.473)	0.95483	1	NA
0.062 (-0.208 - 0.333)	0.138022	1.064 (0.812 - 1.395)	0.695447	1	NA
-0.172 (-0.511 - 0.167)	0.17281	0.842 (0.6 - 1.181)	0.424393	1	NA
0.136 (-0.072 - 0.344)	0.106222	1.146 (0.931 - 1.411)	0.199655	0.998717	NA
0.069 (-0.106 - 0.244)	0.08943	1.071 (0.899 - 1.277)	0.4407	1	NA
0.105 (-0.051 - 0.26)	0.07941	1.11 (0.95 - 1.297)	0.186989	0.998717	NA
-0.063 (-0.299 - 0.172)	0.120204	0.939 (0.742 - 1.188)	0.598038	1	NA
-0.308 (-0.595 - -0.021)	0.146254	0.735 (0.552 - 0.979)	0.035183	0.687993	NA
-0.074 (-0.386 - 0.238)	0.159132	0.928 (0.68 - 1.268)	0.640858	1	NA
-0.226 (-0.624 - 0.173)	0.20309	0.798 (0.536 - 1.188)	0.266763	1	NA
-0.179 (-0.582 - 0.224)	0.205481	0.836 (0.559 - 1.251)	0.383767	1	NA
-0.12 (-0.321 - 0.082)	0.102671	0.887 (0.725 - 1.085)	0.243657	1	NA
-0.094 (-0.279 - 0.091)	0.094235	0.91 (0.757 - 1.095)	0.317772	1	NA
-0.129 (-0.322 - 0.064)	0.098579	0.879 (0.724 - 1.066)	0.189797	0.998717	NA
-0.018 (-0.466 - 0.43)	0.228649	0.982 (0.627 - 1.537)	0.936624	1	NA
0.016 (-0.288 - 0.32)	0.155093	1.016 (0.75 - 1.377)	0.919104	1	NA
0.006 (-0.323 - 0.336)	0.168157	1.006 (0.724 - 1.399)	0.970612	1	NA

-0.02 (-0.312 - 0.272)	0.149075	0.98 (0.732 - 1.313)	0.893818	1	NA
-0.071 (-0.145 - 0.002)	0.03732	0.931 (0.865 - 1.002)	0.0557	0.687993	NA
-0.089 (-0.196 - 0.017)	0.054466	0.915 (0.822 - 1.018)	0.100845	0.890796	NA
-0.077 (-0.158 - 0.003)	0.040952	0.925 (0.854 - 1.003)	0.058524	0.687993	NA
-0.071 (-0.147 - 0.004)	0.038577	0.931 (0.863 - 1.004)	0.064905	0.687993	NA
-0.071 (-0.144 - 0.002)	0.037172	0.932 (0.866 - 1.002)	0.057206	0.687993	NA
-0.162 (-0.408 - 0.085)	0.125823	0.851 (0.665 - 1.089)	0.198557	0.998717	NA
-0.084 (-0.248 - 0.079)	0.083385	0.919 (0.781 - 1.082)	0.312722	1	NA
-0.021 (-0.248 - 0.206)	0.115895	0.979 (0.78 - 1.229)	0.855503	1	NA
-0.154 (-0.394 - 0.086)	0.122336	0.857 (0.674 - 1.089)	0.207281	0.998717	NA
0.12 (-0.089 - 0.33)	0.106902	1.128 (0.915 - 1.391)	0.376989	1	NA
0.078 (-0.097 - 0.253)	0.089207	1.081 (0.908 - 1.288)	0.446467	1	NA
0.088 (-0.07 - 0.245)	0.080513	1.092 (0.932 - 1.278)	0.356311	1	NA
-0.066 (-0.291 - 0.158)	0.114605	0.936 (0.748 - 1.172)	0.622234	1	NA
-0.308 (-0.655 - 0.039)	0.176986	0.735 (0.52 - 1.04)	0.180541	1	NA
-0.071 (-0.417 - 0.276)	0.176693	0.932 (0.659 - 1.318)	0.716595	1	NA
-0.25 (-0.698 - 0.197)	0.228416	0.779 (0.498 - 1.218)	0.387382	1	NA
-0.209 (-0.638 - 0.221)	0.219132	0.812 (0.528 - 1.247)	0.411235	1	NA
-0.116 (-0.338 - 0.107)	0.113487	0.891 (0.713 - 1.113)	0.415535	1	NA
-0.1 (-0.303 - 0.103)	0.103772	0.905 (0.738 - 1.109)	0.379476	1	NA
-0.11 (-0.296 - 0.075)	0.094631	0.896 (0.744 - 1.078)	0.364849	1	NA
-0.173 (-0.707 - 0.36)	0.272142	0.841 (0.493 - 1.434)	0.56961	1	NA
-0.007 (-0.404 - 0.389)	0.202379	0.993 (0.668 - 1.476)	0.974504	1	NA
-0.012 (-0.359 - 0.335)	0.176885	0.988 (0.699 - 1.397)	0.951854	1	NA
-0.035 (-0.337 - 0.267)	0.154283	0.966 (0.714 - 1.307)	0.835021	1	NA
-0.073 (-0.147 - 0)	0.037547	0.929 (0.863 - 1)	0.145687	1	NA
-0.09 (-0.203 - 0.022)	0.057418	0.914 (0.817 - 1.023)	0.257382	1	NA
-0.078 (-0.16 - 0.005)	0.042144	0.925 (0.852 - 1.005)	0.206526	1	NA
-0.073 (-0.149 - 0.003)	0.038771	0.93 (0.862 - 1.003)	0.158092	1	NA
-0.073 (-0.146 - -0.001)	0.037059	0.929 (0.864 - 0.999)	0.142662	1	NA
-0.144 (-0.406 - 0.118)	0.133763	0.866 (0.666 - 1.125)	0.394323	1	NA
-0.089 (-0.253 - 0.075)	0.083736	0.915 (0.776 - 1.078)	0.347378	1	NA
-0.003 (-0.247 - 0.241)	0.1243	0.997 (0.781 - 1.272)	0.983225	1	NA
-0.141 (-0.379 - 0.096)	0.121242	0.868 (0.684 - 1.101)	0.36358	1	NA
-0.174 (-0.485 - 0.138)	0.159051	0.841 (0.615 - 1.148)	0.274797	0.662012	NA

Q_pval	Egger_Intercept	Pleiotropy_pval
0.366144	NA	NA
0.570384	NA	NA
0.111631	NA	NA
0.667397	NA	NA
0.189773	0.060627225	0.62306
0.037953	0.043001034	0.863538
0.035892	0.127109011	0.509444
0.049546	0.027252006	0.900701
0.356335	0.018896746	0.935927
0.461163	NA	NA
0.883645	NA	NA
0.057937	NA	NA
0.406862	0.05531707	0.533536
0.063419	NA	NA
0.066322	NA	NA
0.891014	NA	NA
0.889745	NA	NA
0.592638	NA	NA
0.46793	NA	NA
0.489787	NA	NA
0.738297	-0.039925764	0.893902
0.485036	NA	NA
0.871443	0.017071335	0.882612
0.65798	NA	NA
0.029421	-0.095413217	0.817982
0.526205	-0.009998192	0.80714
0.202454	-0.056948062	0.632822
0.143251	NA	NA
0.064654	0.582978602	0.14394
0.462843	-0.147900188	0.452461
0.414286	-0.138125846	0.577512
0.013235	NA	NA
0.436358	NA	NA
0.169828	NA	NA
0.107002	-0.313771056	0.17923
0.329169	NA	NA
0.494338	NA	NA
0.494801	NA	NA
0.511319	0.022854251	0.709199
0.78287	0.016253396	0.821238
0.758578	0.020405358	0.764584
0.511037	0.023716807	0.700928
0.508223	0.023442069	0.705561
0.119129	NA	NA
0.260967	NA	NA
0.237559	-0.053391718	0.688561
0.112605	-0.066464066	0.714662
0.64717	0.118922843	0.528077
0.647636	NA	NA
0.495431	NA	NA
0.496021	NA	NA
0.238586	-0.054446038	0.695833
0.130332	NA	NA
0.015997	NA	NA
0.038902	NA	NA
0.015448	NA	NA

0.199396	NA	NA
0.321237	NA	NA
0.446892	NA	NA
0.711936	NA	NA
0.010841	NA	NA
0.393521	NA	NA
0.134086	NA	NA
0.416227	NA	NA
0.66198	NA	NA
0.29566	NA	NA
0.373812	NA	NA
0.347975	NA	NA
0.523807	NA	NA
0.525869	NA	NA
0.349704	NA	NA
0.346255	NA	NA
0.134493	NA	NA
0.068852	NA	NA
0.857561	NA	NA
0.132746	NA	NA
NA	NA	NA
NA	NA	NA
NA	NA	NA
NA	NA	NA
NA	NA	NA
NA	NA	NA
NA	NA	NA
NA	NA	NA
NA	NA	NA
NA	NA	NA
NA	NA	NA
NA	NA	NA
NA	NA	NA
NA	NA	NA
NA	NA	NA
NA	NA	NA
NA	NA	NA
NA	NA	NA
NA	NA	NA
NA	NA	NA
NA	NA	NA
NA	NA	NA
NA	NA	NA
NA	NA	NA
NA	NA	NA
NA	NA	NA
NA	NA	NA
NA	NA	NA
NA	NA	NA
NA	NA	NA
NA	NA	NA
NA	NA	NA
NA	NA	NA
NA	NA	NA
NA	NA	NA
NA	NA	NA
NA	NA	NA
NA	NA	NA

NA	NA	NA
NA	NA	NA
NA	NA	NA
NA	NA	NA
NA	NA	NA
NA	NA	NA
NA	NA	NA
NA	NA	NA
NA	NA	NA
NA	NA	NA
NA	NA	NA
NA	NA	NA
NA	NA	NA
NA	NA	NA
NA	NA	NA
NA	NA	NA
NA	NA	NA
NA	NA	NA
NA	NA	NA
NA	NA	NA
NA	NA	NA
NA	NA	NA
NA	NA	NA
NA	NA	NA
NA	NA	NA
NA	NA	NA
NA	NA	NA
NA	NA	NA
NA	NA	NA
NA	NA	NA
NA	NA	NA
NA	NA	NA
NA	NA	NA
NA	NA	NA
NA	NA	NA

Supplementary Table 7

Id.exposure	Outcome	Exposure	Types
GCST90001473	IPF	CD62L- CD86+ myeloic	leukocyte count
GCST90002005	IPF	CD16 on CD14+ CD16+	blood protein measurement
GCST90001957	IPF	CD33 on CD33+ HLA I	blood protein measurement
GCST90001956	IPF	CD33 on CD33+ HLA I	blood protein measurement
GCST90001947	IPF	CD33 on CD33+ HLA I	blood protein measurement
GCST90001465	IPF	CD86+ myeloid Dendrit	leukocyte count
GCST90001952	IPF	CD33 on Monocytic My	blood protein measurement
GCST90001650	IPF	HLA DR+ Natural Kille	leukocyte count
GCST90001648	IPF	HLA DR+ Natural Kille	leukocyte count
GCST90001585	IPF	CD14+ CD16+ monocyt	monocyte count
GCST90001649	IPF	HLA DR+ Natural Kille	leukocyte count
GCST90001580	IPF	CD14+ CD16+ monocyt	monocyte count
GCST90001458	IPF	Myeloid Dendritic Cell /	leukocyte count
GCST90001474	IPF	Plasmacytoid Dendritic (	leukocyte count
GCST90001827	IPF	IgD on IgD+ B cell	blood protein measurement
GCST90002077	IPF	SSC-A on HLA DR+ Na	blood protein measurement
GCST90001970	IPF	FSC-A on HLA DR+ Na	blood protein measurement
GCST90001586	IPF	CD14+ CD16- monocyt	monocyte count
GCST90001587	IPF	CD16+ monocyte %mor	monocyte count
GCST90001950	IPF	CD33 on Granulocytic M	blood protein measurement
GCST90002014	IPF	CCR2 on CD62L+ myel	blood protein measurement
GCST90002017	IPF	CCR2 on monocyte	blood protein measurement
GCST90001944	IPF	CD123 on plasmacytoid	blood protein measurement
GCST90001945	IPF	CD123 on CD62L+ plas	blood protein measurement
GCST90001475	IPF	HLA DR++ monocyte %	leukocyte count
GCST90002005	IPF	CD16 on CD14+ CD16+	blood protein measurement
GCST90002037	IPF	CD80 on plasmacytoid I	blood protein measurement
GCST90002038	IPF	CD80 on CD62L+ plasn	blood protein measurement
GCST90001870	IPF	CD34 on Hematopoietic	blood protein measurement
GCST90001558	IPF	Terminally Differentiate	lymphocyte count
GCST90001527	IPF	CD33dim HLA DR+ CD	myeloid white cell count
GCST90001473	IPF	CD62L- CD86+ myeloic	leukocyte count
GCST90001781	IPF	CD25 on IgD+ CD38- na	blood protein measurement
GCST90002017	IPF	CCR2 on monocyte	blood protein measurement
GCST90001952	IPF	CD33 on Monocytic My	blood protein measurement
GCST90001783	IPF	CD25 on IgD+ CD38+ E	blood protein measurement
GCST90001436	IPF	Memory B cell %lymph	lymphocyte count
GCST90001957	IPF	CD33 on CD33+ HLA I	blood protein measurement
GCST90001947	IPF	CD33 on CD33+ HLA I	blood protein measurement
GCST90001956	IPF	CD33 on CD33+ HLA I	blood protein measurement
GCST90001650	IPF	HLA DR+ Natural Kille	leukocyte count
GCST90001588	IPF	T/B cell	leukocyte count
GCST90001649	IPF	HLA DR+ Natural Kille	leukocyte count
GCST90001520	IPF	CD33+ HLA DR+ CD14	myeloid white cell count
GCST90001957	IPF	CD33 on CD33+ HLA I	blood protein measurement
GCST90001648	IPF	HLA DR+ Natural Kille	leukocyte count
GCST90001783	IPF	CD25 on IgD+ CD38+ E	blood protein measurement
GCST90001473	IPF	CD62L- CD86+ myeloic	leukocyte count
GCST90001956	IPF	CD33 on CD33+ HLA I	blood protein measurement
GCST90001911	IPF	CD45 on Natural Killer	blood protein measurement
GCST90002014	IPF	CCR2 on CD62L+ myel	blood protein measurement
GCST90001956	IPF	CD33 on CD33+ HLA I	blood protein measurement
GCST90001957	IPF	CD33 on CD33+ HLA I	blood protein measurement
GCST90001599	IPF	CD4-CD8- T cell %T ce	leukocyte count
GCST90001597	IPF	CD8dim T cell %T cell	leukocyte count
GCST90001649	IPF	HLA DR+ Natural Kille	leukocyte count

GCST90001947	IPF	CD33 on CD33+ HLA I	blood protein measurement
GCST90001947	IPF	CD33 on CD33+ HLA I	blood protein measurement
GCST90001475	IPF	HLA DR++ monocyte %	leukocyte count
GCST90001475	IPF	HLA DR++ monocyte %	leukocyte count
GCST90001597	IPF	CD8dim T cell %T cell	leukocyte count
GCST90001613	IPF	CD4-CD8- T cell %leuk	leukocyte count
GCST90001465	IPF	CD86+ myeloid Dendrit	leukocyte count
GCST90002005	IPF	CD16 on CD14+ CD16+	blood protein measurement
GCST90002014	IPF	CCR2 on CD62L+ myel	blood protein measurement
GCST90001424	IPF	IgD+ B cell %Lymphocy	lymphocyte count
GCST90001612	IPF	CD8dim T cell %leukoc	leukocyte count
GCST90001870	IPF	CD34 on Hematopoietic	blood protein measurement
GCST90001867	IPF	CD3 on CD4+ T cell	blood protein measurement
GCST90001612	IPF	CD8dim T cell %leukoc	leukocyte count
GCST90002077	IPF	SSC-A on HLA DR+ Na	blood protein measurement
GCST90001952	IPF	CD33 on Monocytic My	blood protein measurement
GCST90001558	IPF	Terminally Differentiate	lymphocyte count
GCST90001970	IPF	FSC-A on HLA DR+ Na	blood protein measurement
GCST90001911	IPF	CD45 on Natural Killer	blood protein measurement
GCST90001596	IPF	CD8dim T cell Absolute	leukocyte count
GCST90001458	IPF	Myeloid Dendritic Cell A	leukocyte count
GCST90001465	IPF	CD86+ myeloid Dendrit	leukocyte count
GCST90001596	IPF	CD8dim T cell Absolute	leukocyte count
GCST90001547	IPF	Terminally Differentiate	lymphocyte count
GCST90002005	IPF	CD16 on CD14+ CD16+	blood protein measurement
GCST90001827	IPF	IgD on IgD+ B cell	blood protein measurement
GCST90001944	IPF	CD123 on plasmacytoid	blood protein measurement
GCST90001748	IPF	CD20 on IgD+ CD38- B	blood protein measurement
GCST90001945	IPF	CD123 on CD62L+ plas	blood protein measurement
GCST90001833	IPF	CD62L on CD62L+ Der	blood protein measurement
GCST90001475	IPF	HLA DR++ monocyte %	leukocyte count
GCST90001558	IPF	Terminally Differentiate	lymphocyte count
GCST90001833	IPF	CD62L on CD62L+ Der	blood protein measurement
GCST90001599	IPF	CD4-CD8- T cell %T ce	leukocyte count
GCST90001959	IPF	CD4 on HLA DR+ CD4	blood protein measurement
GCST90001588	IPF	T/B cell	leukocyte count
GCST90001554	IPF	Effector Memory CD8+	lymphocyte count
GCST90002037	IPF	CD80 on plasmacytoid I	blood protein measurement
GCST90002038	IPF	CD80 on CD62L+ plasn	blood protein measurement
GCST90001783	IPF	CD25 on IgD+ CD38+ I	blood protein measurement
GCST90001474	IPF	Plasmacytoid Dendritic	leukocyte count
GCST90001424	IPF	IgD+ B cell %Lymphocy	lymphocyte count
GCST90002077	IPF	SSC-A on HLA DR+ Na	blood protein measurement
GCST90001580	IPF	CD14+ CD16+ monocyt	monocyte count
GCST90001614	IPF	CD8+ and CD8dim T ce	leukocyte count
GCST90001474	IPF	Plasmacytoid Dendritic	leukocyte count
GCST90001475	IPF	HLA DR++ monocyte %	leukocyte count
GCST90001748	IPF	CD20 on IgD+ CD38- B	blood protein measurement
GCST90001748	IPF	CD20 on IgD+ CD38- B	blood protein measurement
GCST90001648	IPF	HLA DR+ Natural Kille	leukocyte count
GCST90001650	IPF	HLA DR+ Natural Kille	leukocyte count
GCST90001950	IPF	CD33 on Granulocytic M	blood protein measurement
GCST90001613	IPF	CD4-CD8- T cell %leuk	leukocyte count
GCST90001614	IPF	CD8+ and CD8dim T ce	leukocyte count
GCST90001690	IPF	CD28+ CD45RA+ CD8-	lymphocyte count
GCST90001965	IPF	FSC-A on monocyte	blood protein measurement
GCST90001959	IPF	CD4 on HLA DR+ CD4	blood protein measurement
GCST90001649	IPF	HLA DR+ Natural Kille	leukocyte count

GCST90001392	IPF	IgD+ CD38+ B cell Abs	lymphocyte count
GCST90001458	IPF	Myeloid Dendritic Cell A	leukocyte count
GCST90001599	IPF	CD4-CD8- T cell %T ce	leukocyte count
GCST90001520	IPF	CD33+ HLA DR+ CD14	myeloid white cell count
GCST90001870	IPF	CD34 on Hematopoietic	blood protein measurement
GCST90001587	IPF	CD16+ monocyte %mon	monocyte count
GCST90001965	IPF	FSC-A on monocyte	blood protein measurement
GCST90001748	IPF	CD20 on IgD+ CD38- B	blood protein measurement
GCST90001781	IPF	CD25 on IgD+ CD38- na	blood protein measurement
GCST90001585	IPF	CD14+ CD16+ monocyt	monocyte count
GCST90001426	IPF	IgD- CD38dim B cell %	lymphocyte count
GCST90001586	IPF	CD14+ CD16- monocyte	monocyte count
GCST90001613	IPF	CD4-CD8- T cell %leuk	leukocyte count
GCST90001613	IPF	CD4-CD8- T cell %leuk	leukocyte count
GCST90001392	IPF	IgD+ CD38+ B cell Abs	lymphocyte count
GCST90001950	IPF	CD33 on Granulocytic M	blood protein measurement
GCST90001436	IPF	Memory B cell %lympho	lymphocyte count
GCST90001870	IPF	CD34 on Hematopoietic	blood protein measurement
GCST90001781	IPF	CD25 on IgD+ CD38- na	blood protein measurement
GCST90001970	IPF	FSC-A on HLA DR+ Na	blood protein measurement
GCST90002005	IPF	CD16 on CD14+ CD16+	blood protein measurement
GCST90001558	IPF	Terminally Differentiate	lymphocyte count
GCST90001649	IPF	HLA DR+ Natural Kille	leukocyte count
GCST90001465	IPF	CD86+ myeloid Dendrit	leukocyte count
GCST90001547	IPF	Terminally Differentiate	lymphocyte count
GCST90001690	IPF	CD28+ CD45RA+ CD8-	lymphocyte count
GCST90001649	IPF	HLA DR+ Natural Kille	leukocyte count
GCST90001473	IPF	CD62L- CD86+ myeloid	leukocyte count
GCST90001867	IPF	CD3 on CD4+ T cell	blood protein measurement
GCST90001554	IPF	Effector Memory CD8+	lymphocyte count
GCST90001426	IPF	IgD- CD38dim B cell %	lymphocyte count

SNP	Samplesize.outcome	Samplesize.exposure	Steiger_dir	Steiger_pval
rs71632979	198014	3410	TRUE	1.77E-231
rs71632979	198014	3617	TRUE	7.04E-212
rs3865444	198014	1578	TRUE	2.61E-202
rs3865444	198014	1579	TRUE	1.42E-200
rs3865444	198014	1577	TRUE	1.30E-196
rs71632979	198014	3410	TRUE	7.66E-167
rs3865444	198014	1569	TRUE	7.42E-162
rs71632979	198014	3596	TRUE	6.66E-142
rs71632989	198014	3580	TRUE	1.33E-136
rs71632979	198014	3629	TRUE	1.92E-134
rs71632989	198014	3596	TRUE	2.20E-124
rs71632979	198014	3629	TRUE	7.47E-113
rs71632979	198014	3387	TRUE	4.95E-112
rs71632989	198014	3410	TRUE	8.31E-91
rs709589	198014	3657	TRUE	1.06E-80
rs71632979	198014	2971	TRUE	9.57E-74
rs71632979	198014	2971	TRUE	2.11E-68
rs115383270	198014	3629	TRUE	1.57E-64
rs115383270	198014	3629	TRUE	1.99E-62
rs7245846	198014	1244	TRUE	1.87E-59
rs61802333	198014	2870	TRUE	2.40E-28
rs4683184	198014	2850	TRUE	2.54E-26
rs7658518	198014	2872	TRUE	9.37E-23
rs7658518	198014	2872	TRUE	3.00E-22
rs9271296	198014	3595	TRUE	1.15E-21
rs115805162	198014	3617	TRUE	5.86E-21
rs7658518	198014	2871	TRUE	3.16E-20
rs7658518	198014	2871	TRUE	8.91E-20
rs139489968	198014	1634	TRUE	1.75E-19
rs6707421	198014	3427	TRUE	2.12E-19
rs67760360	198014	1858	TRUE	6.23E-19
rs115805162	198014	3410	TRUE	9.60E-19
rs41294937	198014	1835	TRUE	6.15E-18
rs1801274	198014	2850	TRUE	9.59E-18
rs7351079	198014	1569	TRUE	1.17E-17
rs62626323	198014	3656	TRUE	3.34E-17
rs12874404	198014	3656	TRUE	9.49E-17
rs558625031	198014	1578	TRUE	1.00E-16
rs558625031	198014	1577	TRUE	2.32E-16
rs558625031	198014	1579	TRUE	2.96E-16
rs115805162	198014	3596	TRUE	3.24E-16
rs12874404	198014	3653	TRUE	3.96E-16
rs7180804	198014	3596	TRUE	4.81E-16
rs3865444	198014	1858	TRUE	5.61E-16
rs7351079	198014	1578	TRUE	1.42E-15
rs115805162	198014	3580	TRUE	1.52E-15
rs709589	198014	3656	TRUE	2.25E-15
rs71639910	198014	3410	TRUE	2.94E-15
rs7351079	198014	1579	TRUE	3.36E-15
rs6721978	198014	3113	TRUE	3.68E-15
rs35587265	198014	2870	TRUE	5.13E-15
rs78949787	198014	1579	TRUE	5.43E-15
rs78949787	198014	1578	TRUE	6.28E-15
rs7569868	198014	3668	TRUE	7.84E-15
rs139249541	198014	3668	TRUE	1.10E-14
rs115805162	198014	3596	TRUE	2.16E-14

rs78949787	198014	1577	TRUE	2.31E-14
rs7351079	198014	1577	TRUE	3.16E-14
rs9271768	198014	3595	TRUE	3.25E-14
rs9271768	198014	3595	TRUE	6.03E-14
rs79086781	198014	3668	TRUE	8.33E-14
rs7569868	198014	3668	TRUE	1.06E-13
rs115805162	198014	3410	TRUE	1.12E-13
rs71639910	198014	3617	TRUE	1.58E-13
rs6687275	198014	2870	TRUE	3.13E-13
rs12874404	198014	3657	TRUE	8.23E-13
rs79086781	198014	3668	TRUE	1.08E-12
rs4406271	198014	1634	TRUE	1.36E-12
rs4657649	198014	2919	TRUE	1.89E-12
rs139249541	198014	3668	TRUE	1.95E-12
rs115805162	198014	2971	TRUE	2.69E-12
rs62114147	198014	1569	TRUE	3.66E-12
rs541367304	198014	3427	TRUE	9.13E-12
rs115805162	198014	2971	TRUE	1.78E-11
rs183053322	198014	3113	TRUE	1.87E-11
rs139249541	198014	3652	TRUE	2.55E-11
rs115805162	198014	3387	TRUE	2.84E-11
rs71639910	198014	3410	TRUE	3.14E-11
rs79251210	198014	3652	TRUE	5.14E-11
rs62400929	198014	3427	TRUE	5.27E-11
rs187197560	198014	3617	TRUE	5.78E-11
rs28669075	198014	3657	TRUE	7.23E-11
rs62447171	198014	2872	TRUE	1.03E-10
rs30003	198014	3645	TRUE	1.18E-10
rs62447171	198014	2872	TRUE	1.21E-10
rs3917768	198014	2871	TRUE	1.59E-10
rs9296042	198014	3595	TRUE	1.88E-10
rs79827501	198014	3427	TRUE	2.37E-10
rs7526645	198014	2871	TRUE	2.64E-10
rs139249541	198014	3668	TRUE	2.69E-10
rs4371876	198014	3060	TRUE	3.56E-10
rs9892810	198014	3653	TRUE	3.92E-10
rs17583875	198014	3395	TRUE	4.87E-10
rs62447171	198014	2871	TRUE	6.08E-10
rs62447171	198014	2871	TRUE	6.55E-10
rs3793662	198014	3656	TRUE	6.78E-10
rs876036	198014	3410	TRUE	7.90E-10
rs11657531	198014	3657	TRUE	1.05E-09
rs569911	198014	2971	TRUE	1.12E-09
rs71639910	198014	3629	TRUE	1.30E-09
rs11080350	198014	3668	TRUE	2.06E-09
rs115805162	198014	3410	TRUE	2.41E-09
rs62395272	198014	3595	TRUE	3.23E-09
rs2070770	198014	3645	TRUE	3.47E-09
rs12554596	198014	3645	TRUE	4.25E-09
rs71639910	198014	3580	TRUE	6.07E-09
rs569911	198014	3596	TRUE	6.38E-09
rs7351079	198014	1244	TRUE	6.44E-09
rs139249541	198014	3668	TRUE	6.72E-09
rs73402222	198014	3668	TRUE	6.90E-09
rs3131627	198014	3408	TRUE	7.36E-09
rs77762937	198014	2850	TRUE	8.79E-09
rs9357097	198014	3060	TRUE	1.11E-08
rs9916257	198014	3596	TRUE	1.13E-08

rs80330977	198014	3656	TRUE	1.16E-08
rs569911	198014	3387	TRUE	1.33E-08
rs79251210	198014	3668	TRUE	1.34E-08
rs74253876	198014	1858	TRUE	1.70E-08
rs586030	198014	1634	TRUE	1.86E-08
rs74975395	198014	3629	TRUE	2.01E-08
rs1800973	198014	2850	TRUE	2.05E-08
rs2066399	198014	3645	TRUE	2.11E-08
rs709589	198014	1835	TRUE	2.22E-08
rs71639910	198014	3629	TRUE	2.27E-08
rs7840395	198014	3656	TRUE	2.40E-08
rs74975395	198014	3629	TRUE	2.44E-08
rs79251210	198014	3668	TRUE	3.21E-08
rs9896155	198014	3668	TRUE	3.81E-08
rs139260757	198014	3656	TRUE	3.88E-08
rs78949787	198014	1244	TRUE	4.20E-08
rs11653761	198014	3656	TRUE	4.39E-08
rs36102158	198014	1634	TRUE	5.39E-08
rs2275848	198014	1835	TRUE	5.66E-08
rs569911	198014	2971	TRUE	5.85E-08
rs147678401	198014	3617	TRUE	5.96E-08
rs13023673	198014	3427	TRUE	6.67E-08
rs865239	198014	3596	TRUE	7.95E-08
rs115624142	198014	3410	TRUE	8.85E-08
rs9501107	198014	3427	TRUE	8.91E-08
rs12901644	198014	3408	TRUE	9.47E-08
rs569911	198014	3596	TRUE	1.06E-07
rs78600824	198014	3410	TRUE	1.08E-07
rs41289614	198014	2919	TRUE	1.10E-07
rs3130685	198014	3395	TRUE	1.33E-07
rs11687597	198014	3656	TRUE	1.59E-07

Steiger FDR
2.57E-229
5.11E-210
1.26E-200
5.14E-199
3.77E-195
1.85E-165
1.54E-160
1.21E-140
2.15E-135
2.78E-133
2.90E-123
9.03E-112
5.52E-111
8.60E-90
1.02E-79
8.67E-73
1.80E-67
1.26E-63
1.52E-61
1.36E-58
1.66E-27
1.67E-25
5.90E-22
1.81E-21
6.69E-21
3.27E-20
1.70E-19
4.62E-19
8.75E-19
1.03E-18
2.91E-18
4.35E-18
2.70E-17
4.09E-17
4.85E-17
1.34E-16
3.72E-16
3.82E-16
8.63E-16
1.07E-15
1.15E-15
1.37E-15
1.62E-15
1.85E-15
4.57E-15
4.80E-15
6.95E-15
8.89E-15
9.95E-15
1.07E-14
1.46E-14
1.51E-14
1.72E-14
2.10E-14
2.89E-14
5.60E-14

5.87E-14
7.90E-14
7.99E-14
1.46E-13
1.98E-13
2.48E-13
2.59E-13
3.58E-13
6.98E-13
1.81E-12
2.34E-12
2.91E-12
3.96E-12
4.03E-12
5.50E-12
7.37E-12
1.81E-11
3.49E-11
3.61E-11
4.86E-11
5.35E-11
5.83E-11
9.43E-11
9.55E-11
1.04E-10
1.28E-10
1.81E-10
2.05E-10
2.06E-10
2.69E-10
3.13E-10
3.91E-10
4.30E-10
4.33E-10
5.68E-10
6.18E-10
7.59E-10
9.37E-10
1.00E-09
1.02E-09
1.18E-09
1.55E-09
1.64E-09
1.88E-09
2.96E-09
3.43E-09
4.54E-09
4.84E-09
5.87E-09
8.30E-09
8.64E-09
8.65E-09
8.95E-09
9.10E-09
9.61E-09
1.14E-08
1.43E-08
1.44E-08

1.47E-08
1.66E-08
1.66E-08
2.09E-08
2.27E-08
2.42E-08
2.46E-08
2.50E-08
2.62E-08
2.66E-08
2.78E-08
2.80E-08
3.66E-08
4.32E-08
4.36E-08
4.69E-08
4.86E-08
5.92E-08
6.18E-08
6.33E-08
6.40E-08
7.11E-08
8.41E-08
9.29E-08
9.30E-08
9.81E-08
1.09E-07
1.10E-07
1.12E-07
1.34E-07
1.59E-07

Supplementary Table 8

Exposure	Outcome	Method	nsnp	β coefficient (95%CI)
IPF	CD4+Temra %T cell	MR Egger	20	-0.016 (-0.06 - 0.029)
		Weighted median		-0.009 (-0.047 - 0.029)
		IVW		0.003 (-0.028 - 0.034)
		Simple mode		0.024 (-0.064 - 0.113)
		Weighted mode		-0.008 (-0.045 - 0.028)

Discovery cohort					
SE	OR (95%CI)	Pval	Cochrane Q	Q pval	Egger Intercept
0.022580716	0.984 (0.942 - 1.029	0.494569	17.15306609	0.512606	NA
0.019197866	0.991 (0.954 - 1.029	0.634933	NA	NA	NA
0.015712857	1.003 (0.973 - 1.034	0.842865	18.50536391	0.488953	0.011659607
0.045180604	1.025 (0.938 - 1.12)	0.594492	NA	NA	NA
0.018610311	0.992 (0.956 - 1.029	0.660485	NA	NA	NA

Pleiotropy pval	MR Presso pval	nsnp	β coefficient (95%CI)	SE	OR (95%CI)
NA			-0.076 (-0.299 - 0.147)	0.11386	0.927 (0.741 - 1.158
NA			-0.012 (-0.048 - 0.025)	0.018466	0.988 (0.953 - 1.025
0.26006248	0.455	6	-0.009 (-0.045 - 0.026)	0.018186	0.991 (0.956 - 1.027
NA			0.006 (-0.069 - 0.08)	0.038001	1.006 (0.933 - 1.083
NA			-0.011 (-0.045 - 0.023)	0.017471	0.989 (0.956 - 1.024

Validation cohort					
**Pval**	**Cochrane (**	**Q pval**	**Egger Intercept**	**Pleiotropy pval**	**MR Presso pval**
0.540587419	4.913783	0.296259	NA	NA	
0.528314752	NA	NA	NA	NA	
0.607535125	5.348604	0.374833	0.098834623	0.583902481	0.61
0.889569379	NA	NA	NA	NA	
0.560842358	NA	NA	NA	NA	

Supplementary Table 9

snp	Traits	Catalog	ref_a1	ref_a2	proxy
rs62400929	White blood cell count	GWAS	A	G	0
rs62400929	Lymphocyte count	GWAS	A	G	1
rs62400929	White blood cell count	GWAS	A	G	1
rs62400929	White blood cell count	GWAS	A	G	1
rs62400929	White blood cell count	GWAS	A	G	1
rs62400929	White blood cell count	GWAS	A	G	1
rs62400929	White blood cell count	GWAS	A	G	1
rs62400929	White blood cell count	GWAS	A	G	1
rs62400929	Lymphocyte count	GWAS	A	G	1
rs62400929	White blood cell count	GWAS	A	G	1
rs62400929	White blood cell count	GWAS	A	G	1
rs62400929	Lymphocyte count	GWAS	A	G	1
rs62400929	White blood cell count	GWAS	A	G	1
rs62400929	White blood cell count	GWAS	A	G	1
rs62400929	White blood cell count	GWAS	A	G	1
rs62400929	White blood cell count	GWAS	A	G	1
rs62400929	White blood cell count	GWAS	A	G	1
rs9501107	Lymphocyte count	GWAS	G	T	0
rs9501107	Monocyte percentage of white cells	GWAS	G	T	0
rs9501107	Neutrophil count	GWAS	G	T	0
rs9501107	Sum basophil neutrophil counts	GWAS	G	T	0
rs9501107	White blood cell count	GWAS	G	T	0
rs9501107	Primary sclerosing cholangitis	GWAS	G	T	0
rs9501107	Asthma	GWAS	G	T	0
rs9501107	Hayfever, allergic rhinitis or eczema	GWAS	G	T	0
rs9501107	Height	GWAS	G	T	0
rs9501107	Intestinal malabsorption	GWAS	G	T	0
rs9501107	No blood clot, bronchitis, emphysen	GWAS	G	T	0
rs9501107	Self-reported asthma	GWAS	G	T	0
rs9501107	Self-reported hypothyroidism or my:	GWAS	G	T	0
rs9501107	Self-reported malabsorption or coeli	GWAS	G	T	0
rs9501107	Sitting height	GWAS	G	T	0
rs9501107	Treatment with levothyroxine sodiu	GWAS	G	T	0
rs9501107	Lymphocyte count	GWAS	G	T	1
rs9501107	Sum basophil neutrophil counts	GWAS	G	T	1
rs9501107	White blood cell count	GWAS	G	T	1
rs9501107	Primary sclerosing cholangitis	GWAS	G	T	1
rs9501107	Asthma	GWAS	G	T	1
rs9501107	Hayfever, allergic rhinitis or eczema	GWAS	G	T	1
rs9501107	Height	GWAS	G	T	1
rs9501107	Intestinal malabsorption	GWAS	G	T	1
rs9501107	No blood clot, bronchitis, emphysen	GWAS	G	T	1
rs9501107	Self-reported asthma	GWAS	G	T	1
rs9501107	Self-reported hypothyroidism or my:	GWAS	G	T	1
rs9501107	Self-reported malabsorption or coeli	GWAS	G	T	1
rs9501107	Sitting height	GWAS	G	T	1
rs9501107	Treatment with levothyroxine sodiu	GWAS	G	T	1
rs9501107	Granulocyte count	GWAS	G	T	1
rs9501107	Lymphocyte count	GWAS	G	T	1
rs9501107	Monocyte percentage of white cells	GWAS	G	T	1
rs9501107	Myeloid white cell count	GWAS	G	T	1
rs9501107	Neutrophil count	GWAS	G	T	1
rs9501107	Sum basophil neutrophil counts	GWAS	G	T	1
rs9501107	Sum neutrophil eosinophil counts	GWAS	G	T	1
rs9501107	White blood cell count	GWAS	G	T	1
rs9501107	Granulocyte count	GWAS	G	T	1

rs9501107	Lymphocyte count	GWAS	G	T	1
rs9501107	Myeloid white cell count	GWAS	G	T	1
rs9501107	Neutrophil count	GWAS	G	T	1
rs9501107	Sum basophil neutrophil counts	GWAS	G	T	1
rs9501107	Sum neutrophil eosinophil counts	GWAS	G	T	1
rs9501107	White blood cell count	GWAS	G	T	1
rs9501107	Primary sclerosing cholangitis	GWAS	G	T	1
rs9501107	Asthma	GWAS	G	T	1
rs9501107	Hayfever, allergic rhinitis or eczema	GWAS	G	T	1
rs9501107	Height	GWAS	G	T	1
rs9501107	No blood clot, bronchitis, emphysen	GWAS	G	T	1
rs9501107	Self-reported asthma	GWAS	G	T	1
rs9501107	Self-reported hypothyroidism or my:	GWAS	G	T	1
rs9501107	Self-reported malabsorption or coeli	GWAS	G	T	1
rs9501107	Self-reported thyroiditis	GWAS	G	T	1
rs9501107	Sitting height	GWAS	G	T	1
rs9501107	Treatment with levothyroxine sodiu	GWAS	G	T	1
rs9501107	Granulocyte count	GWAS	G	T	1
rs9501107	Lymphocyte count	GWAS	G	T	1
rs9501107	Myeloid white cell count	GWAS	G	T	1
rs9501107	Neutrophil count	GWAS	G	T	1
rs9501107	Sum basophil neutrophil counts	GWAS	G	T	1
rs9501107	Sum neutrophil eosinophil counts	GWAS	G	T	1
rs9501107	White blood cell count	GWAS	G	T	1
rs9501107	Primary sclerosing cholangitis	GWAS	G	T	1
rs9501107	Asthma	GWAS	G	T	1
rs9501107	Hayfever, allergic rhinitis or eczema	GWAS	G	T	1
rs9501107	Height	GWAS	G	T	1
rs9501107	No blood clot, bronchitis, emphysen	GWAS	G	T	1
rs9501107	Self-reported asthma	GWAS	G	T	1
rs9501107	Self-reported hypothyroidism or my:	GWAS	G	T	1
rs9501107	Self-reported malabsorption or coeli	GWAS	G	T	1
rs9501107	Self-reported thyroiditis	GWAS	G	T	1
rs9501107	Sitting height	GWAS	G	T	1
rs9501107	Treatment with levothyroxine sodiu	GWAS	G	T	1
rs9501107	Lymphocyte count	GWAS	G	T	1
rs9501107	Lymphocyte percentage of white cel	GWAS	G	T	1
rs9501107	White blood cell count	GWAS	G	T	1
rs9501107	Primary sclerosing cholangitis	GWAS	G	T	1
rs9501107	Asthma	GWAS	G	T	1
rs9501107	Birth weight of first child	GWAS	G	T	1
rs9501107	Hayfever, allergic rhinitis or eczema	GWAS	G	T	1
rs9501107	Height	GWAS	G	T	1
rs9501107	No blood clot, bronchitis, emphysen	GWAS	G	T	1
rs9501107	Self-reported asthma	GWAS	G	T	1
rs9501107	Self-reported hypothyroidism or my:	GWAS	G	T	1
rs9501107	Self-reported malabsorption or coeli	GWAS	G	T	1
rs9501107	Sitting height	GWAS	G	T	1
rs9501107	Treatment with levothyroxine sodiu	GWAS	G	T	1
rs9501107	Lymphocyte count	GWAS	G	T	1
rs9501107	Lymphocyte percentage of white cel	GWAS	G	T	1
rs9501107	Monocyte percentage of white cells	GWAS	G	T	1
rs9501107	White blood cell count	GWAS	G	T	1
rs9501107	Primary sclerosing cholangitis	GWAS	G	T	1
rs9501107	Asthma	GWAS	G	T	1
rs9501107	Birth weight of first child	GWAS	G	T	1
rs9501107	Hayfever, allergic rhinitis or eczema	GWAS	G	T	1
rs9501107	Height	GWAS	G	T	1

rs9501107	No blood clot, bronchitis, emphysen	GWAS	G	T	1
rs9501107	Self-reported asthma	GWAS	G	T	1
rs9501107	Self-reported hypothyroidism or my:	GWAS	G	T	1
rs9501107	Self-reported malabsorption or coeli	GWAS	G	T	1
rs9501107	Sitting height	GWAS	G	T	1
rs9501107	Treatment with levothyroxine sodiu	GWAS	G	T	1
rs9501107	Lymphocyte count	GWAS	G	T	1
rs9501107	Lymphocyte percentage of white cel	GWAS	G	T	1
rs9501107	Monocyte percentage of white cells	GWAS	G	T	1
rs9501107	White blood cell count	GWAS	G	T	1
rs9501107	Primary sclerosing cholangitis	GWAS	G	T	1
rs9501107	Asthma	GWAS	G	T	1
rs9501107	Birth weight of first child	GWAS	G	T	1
rs9501107	Hayfever, allergic rhinitis or eczema	GWAS	G	T	1
rs9501107	Height	GWAS	G	T	1
rs9501107	No blood clot, bronchitis, emphysen	GWAS	G	T	1
rs9501107	Self-reported asthma	GWAS	G	T	1
rs9501107	Self-reported hypothyroidism or my:	GWAS	G	T	1
rs9501107	Self-reported malabsorption or coeli	GWAS	G	T	1
rs9501107	Sitting height	GWAS	G	T	1
rs9501107	Treatment with levothyroxine sodiu	GWAS	G	T	1
rs9501107	Lymphocyte count	GWAS	G	T	1
rs9501107	Lymphocyte percentage of white cel	GWAS	G	T	1
rs9501107	Monocyte percentage of white cells	GWAS	G	T	1
rs9501107	White blood cell count	GWAS	G	T	1
rs9501107	Primary sclerosing cholangitis	GWAS	G	T	1
rs9501107	Asthma	GWAS	G	T	1
rs9501107	Hayfever, allergic rhinitis or eczema	GWAS	G	T	1
rs9501107	Height	GWAS	G	T	1
rs9501107	No blood clot, bronchitis, emphysen	GWAS	G	T	1
rs9501107	Self-reported asthma	GWAS	G	T	1
rs9501107	Self-reported hypothyroidism or my:	GWAS	G	T	1
rs9501107	Self-reported malabsorption or coeli	GWAS	G	T	1
rs9501107	Sitting height	GWAS	G	T	1
rs9501107	Treatment with levothyroxine sodiu	GWAS	G	T	1
rs9501107	Lymphocyte count	GWAS	G	T	1
rs9501107	Lymphocyte percentage of white cel	GWAS	G	T	1
rs9501107	Monocyte percentage of white cells	GWAS	G	T	1
rs9501107	White blood cell count	GWAS	G	T	1
rs9501107	Primary sclerosing cholangitis	GWAS	G	T	1
rs9501107	Asthma	GWAS	G	T	1
rs9501107	Hayfever, allergic rhinitis or eczema	GWAS	G	T	1
rs9501107	Height	GWAS	G	T	1
rs9501107	No blood clot, bronchitis, emphysen	GWAS	G	T	1
rs9501107	Self-reported asthma	GWAS	G	T	1
rs9501107	Self-reported hypothyroidism or my:	GWAS	G	T	1
rs9501107	Self-reported malabsorption or coeli	GWAS	G	T	1
rs9501107	Sitting height	GWAS	G	T	1
rs9501107	Treatment with levothyroxine sodiu	GWAS	G	T	1
rs9501107	Lymphocyte count	GWAS	G	T	1
rs9501107	Lymphocyte percentage of white cel	GWAS	G	T	1
rs9501107	Monocyte percentage of white cells	GWAS	G	T	1
rs9501107	White blood cell count	GWAS	G	T	1
rs9501107	Primary sclerosing cholangitis	GWAS	G	T	1
rs9501107	TCF19	eQTL	G	T	1
rs9501107	TCF19	eQTL	G	T	1
rs9501107	TCF19	eQTL	G	T	1
rs9501107	TCF19	eQTL	G	T	1

rs9501107	POU5F1	eQTL	G	T	1
rs9501107	POU5F1	eQTL	G	T	1
rs9501107	POU5F1	eQTL	G	T	1
rs9501107	TCF19	eQTL	G	T	0
rs9501107	TCF19	eQTL	G	T	1
rs9501107	Percent-splice-in	mQTL	G	T	0
rs9501107	Percent-splice-in	mQTL	G	T	0
rs9501107	cg07103722	mQTL	G	T	0
rs9501107	cg11268327	mQTL	G	T	0
rs9501107	cg14624895	mQTL	G	T	0
rs9501107	cg16655385	mQTL	G	T	0
rs9501107	cg02884661	mQTL	G	T	0
rs9501107	cg05651282	mQTL	G	T	0
rs9501107	cg11268327	mQTL	G	T	0
rs9501107	cg12182649	mQTL	G	T	0
rs9501107	cg14624895	mQTL	G	T	0
rs9501107	cg16655385	mQTL	G	T	0
rs9501107	cg27527338	mQTL	G	T	0
rs9501107	cg14624895	mQTL	G	T	0
rs9501107	cg17964422	mQTL	G	T	0
rs9501107	cg18902440	mQTL	G	T	0
rs9501107	cg17964422	mQTL	G	T	0
rs9501107	Percent-splice-in	mQTL	G	T	1
rs9501107	Percent-splice-in	mQTL	G	T	1
rs9501107	cg07103722	mQTL	G	T	1
rs9501107	cg11268327	mQTL	G	T	1
rs9501107	cg14624895	mQTL	G	T	1
rs9501107	cg16655385	mQTL	G	T	1
rs9501107	cg02884661	mQTL	G	T	1
rs9501107	cg05651282	mQTL	G	T	1
rs9501107	cg11268327	mQTL	G	T	1
rs9501107	cg12182649	mQTL	G	T	1
rs9501107	cg14624895	mQTL	G	T	1
rs9501107	cg16655385	mQTL	G	T	1
rs9501107	cg27527338	mQTL	G	T	1
rs9501107	cg14624895	mQTL	G	T	1
rs9501107	cg17964422	mQTL	G	T	1
rs9501107	cg18902440	mQTL	G	T	1
rs9501107	cg17964422	mQTL	G	T	1
rs9501107	Percent-splice-in	mQTL	G	T	1
rs9501107	Percent-splice-in	mQTL	G	T	1
rs9501107	cg07103722	mQTL	G	T	1
rs9501107	cg11268327	mQTL	G	T	1
rs9501107	cg05651282	mQTL	G	T	1
rs9501107	cg11268327	mQTL	G	T	1
rs9501107	cg14624895	mQTL	G	T	1
rs9501107	cg17964422	mQTL	G	T	1
rs9501107	Percent-splice-in	mQTL	G	T	1
rs9501107	Percent-splice-in	mQTL	G	T	1
rs9501107	cg07103722	mQTL	G	T	1
rs9501107	cg11268327	mQTL	G	T	1
rs9501107	cg05651282	mQTL	G	T	1
rs9501107	cg11268327	mQTL	G	T	1
rs9501107	cg14624895	mQTL	G	T	1
rs9501107	Percent-splice-in	mQTL	G	T	1
rs9501107	Percent-splice-in	mQTL	G	T	1
rs9501107	cg07103722	mQTL	G	T	1
rs9501107	cg11268327	mQTL	G	T	1

rs9501107	cg14624895	mQTL	G	T	1
rs9501107	cg23679615	mQTL	G	T	1
rs9501107	cg05651282	mQTL	G	T	1
rs9501107	cg11268327	mQTL	G	T	1
rs9501107	cg14624895	mQTL	G	T	1
rs9501107	cg27527338	mQTL	G	T	1
rs9501107	cg01190171	mQTL	G	T	1
rs9501107	cg07400063	mQTL	G	T	1
rs9501107	cg17964422	mQTL	G	T	1
rs9501107	cg14624895	mQTL	G	T	1
rs9501107	cg17964422	mQTL	G	T	1
rs9501107	cg18902440	mQTL	G	T	1
rs9501107	cg17964422	mQTL	G	T	1
rs9501107	cg17964422	mQTL	G	T	1
rs9501107	cg17964422	mQTL	G	T	1
rs9501107	Percent-splice-in	mQTL	G	T	1
rs9501107	Percent-splice-in	mQTL	G	T	1
rs9501107	cg07103722	mQTL	G	T	1
rs9501107	cg11268327	mQTL	G	T	1
rs9501107	cg14624895	mQTL	G	T	1
rs9501107	cg23679615	mQTL	G	T	1
rs9501107	cg05651282	mQTL	G	T	1
rs9501107	cg11268327	mQTL	G	T	1
rs9501107	cg14624895	mQTL	G	T	1
rs9501107	cg27527338	mQTL	G	T	1
rs9501107	cg01190171	mQTL	G	T	1
rs9501107	cg07400063	mQTL	G	T	1
rs9501107	cg17964422	mQTL	G	T	1
rs9501107	cg14624895	mQTL	G	T	1
rs9501107	cg17964422	mQTL	G	T	1
rs9501107	cg18902440	mQTL	G	T	1
rs9501107	cg17964422	mQTL	G	T	1
rs9501107	cg17964422	mQTL	G	T	1
rs9501107	cg17964422	mQTL	G	T	1
rs9501107	Percent-splice-in	mQTL	G	T	1
rs9501107	Percent-splice-in	mQTL	G	T	1
rs9501107	cg07103722	mQTL	G	T	1
rs9501107	cg11268327	mQTL	G	T	1
rs9501107	cg14624895	mQTL	G	T	1
rs9501107	cg05651282	mQTL	G	T	1
rs9501107	cg11268327	mQTL	G	T	1
rs9501107	cg14624895	mQTL	G	T	1
rs9501107	cg17964422	mQTL	G	T	1
rs9501107	cg14624895	mQTL	G	T	1
rs9501107	cg17964422	mQTL	G	T	1
rs9501107	cg18902440	mQTL	G	T	1
rs9501107	cg17964422	mQTL	G	T	1
rs9501107	cg17964422	mQTL	G	T	1
rs9501107	cg17964422	mQTL	G	T	1
rs9501107	cg17964422	mQTL	G	T	1
rs9501107	cg14624895	mQTL	G	T	1
rs9501107	cg17964422	mQTL	G	T	1
rs9501107	cg18902440	mQTL	G	T	1
rs9501107	cg17964422	mQTL	G	T	1
rs9501107	cg17964422	mQTL	G	T	1
rs9501107	cg17964422	mQTL	G	T	1
rs9501107	Percent-splice-in	mQTL	G	T	1
rs9501107	Percent-splice-in	mQTL	G	T	1

rs9501107	cg07103722	mQTL	G	T	1
rs9501107	cg11268327	mQTL	G	T	1
rs9501107	cg14624895	mQTL	G	T	1
rs9501107	cg23679615	mQTL	G	T	1
rs9501107	cg05651282	mQTL	G	T	1
rs9501107	cg11268327	mQTL	G	T	1
rs9501107	cg14624895	mQTL	G	T	1
rs9501107	cg27527338	mQTL	G	T	1
rs9501107	cg17964422	mQTL	G	T	1
rs9501107	cg14624895	mQTL	G	T	1
rs9501107	cg17964422	mQTL	G	T	1
rs9501107	cg18902440	mQTL	G	T	1
rs9501107	cg17964422	mQTL	G	T	1
rs9501107	cg17964422	mQTL	G	T	1
rs9501107	cg17964422	mQTL	G	T	1
rs9501107	cg02960716	mQTL	G	T	1
rs9501107	cg08188015	mQTL	G	T	1
rs9501107	cg04710276	mQTL	G	T	1
rs9501107	cg10536916	mQTL	G	T	1
rs9501107	cg26036029	mQTL	G	T	1
rs9501107	cg01247537	mQTL	G	T	1
rs9501107	cg20240154	mQTL	G	T	1
rs9501107	cg26036029	mQTL	G	T	1
rs9501107	cg27300494	mQTL	G	T	1

r2	beta	se	Pval	pmid	ancestry	ref_hg19_coordinates
1	-0.08473	0.01424	2.67E-09	27863252	European	chr6:31159546
1	-0.08307	0.01391	2.34E-09	27863252	European	chr6:31159546
1	-0.08884	0.01385	1.4E-10	27863252	European	chr6:31159546
1	-0.08459	0.01423	2.79E-09	27863252	European	chr6:31159546
1	-0.08486	0.01424	2.51E-09	27863252	European	chr6:31159546
1	-0.08484	0.01424	2.52E-09	27863252	European	chr6:31159546
1	-0.08486	0.01424	2.51E-09	27863252	European	chr6:31159546
1	-0.0885	0.01426	5.38E-10	27863252	European	chr6:31159546
1	-0.07825	0.01433	4.79E-08	27863252	European	chr6:31159546
1	-0.08859	0.01427	5.35E-10	27863252	European	chr6:31159546
1	-0.08499	0.01421	2.22E-09	27863252	European	chr6:31159546
1	-0.07782	0.01425	4.72E-08	27863252	European	chr6:31159546
1	-0.08675	0.01418	9.58E-10	27863252	European	chr6:31159546
1	-0.08627	0.01419	1.2E-09	27863252	European	chr6:31159546
1	-0.08359	0.01408	2.94E-09	27863252	European	chr6:31159546
1	-0.08563	0.01421	1.68E-09	27863252	European	chr6:31159546
1	-0.08558	0.01421	1.71E-09	27863252	European	chr6:31159546
1	-0.08569	0.007693	8.15E-29	27863252	European	chr6:31392047
1	0.04156	0.007621	4.95E-08	27863252	European	chr6:31392047
1	-0.04236	0.007656	3.16E-08	27863252	European	chr6:31392047
1	-0.0432	0.00767	1.78E-08	27863252	European	chr6:31392047
1	-0.06651	0.007658	3.79E-18	27863252	European	chr6:31392047
1	0.3873	0.068	3.45E-09	27992413	European	chr6:31392047
1	0.01126	0.001658	1.1E-11	UKBB	European	chr6:31392047
1	0.01609	0.002188	1.9E-13	UKBB	European	chr6:31392047
1	0.03338	0.003679	1.16E-19	UKBB	European	chr6:31392047
1	0.001267	0.000223	1.38E-08	UKBB	European	chr6:31392047
1	-0.01951	0.002424	8.47E-16	UKBB	European	chr6:31392047
1	0.01131	0.001662	9.91E-12	UKBB	European	chr6:31392047
1	-0.00896	0.001106	5.16E-16	UKBB	European	chr6:31392047
1	0.002726	0.00034	1.09E-15	UKBB	European	chr6:31392047
1	0.02918	0.004002	3.08E-13	UKBB	European	chr6:31392047
1	-0.00653	0.001019	1.47E-10	UKBB	European	chr6:31392047
0.98806	-0.08531	0.0077	1.59E-28	27863252	European	chr6:31392047
0.98806	-0.04238	0.007676	3.38E-08	27863252	European	chr6:31392047
0.98806	-0.06551	0.007665	1.26E-17	27863252	European	chr6:31392047
0.98806	0.4048	0.069	1.19E-09	27992413	European	chr6:31392047
0.98806	0.01114	0.00166	1.9E-11	UKBB	European	chr6:31392047
0.98806	0.01607	0.00219	2.17E-13	UKBB	European	chr6:31392047
0.98806	0.0336	0.003683	7.38E-20	UKBB	European	chr6:31392047
0.98806	0.001264	0.000224	1.57E-08	UKBB	European	chr6:31392047
0.98806	-0.01944	0.002427	1.14E-15	UKBB	European	chr6:31392047
0.98806	0.01119	0.001663	1.72E-11	UKBB	European	chr6:31392047
0.98806	-0.00898	0.001107	5.03E-16	UKBB	European	chr6:31392047
0.98806	0.002722	0.00034	1.31E-15	UKBB	European	chr6:31392047
0.98806	0.02944	0.004006	2.02E-13	UKBB	European	chr6:31392047
0.98806	-0.00647	0.00102	2.19E-10	UKBB	European	chr6:31392047
0.83399	-0.04832	0.007603	2.09E-10	27863252	European	chr6:31392047
0.83399	-0.08832	0.00762	4.58E-31	27863252	European	chr6:31392047
0.83399	0.04888	0.007547	9.4E-11	27863252	European	chr6:31392047
0.83399	-0.04588	0.007623	1.75E-09	27863252	European	chr6:31392047
0.83399	-0.04814	0.007584	2.18E-10	27863252	European	chr6:31392047
0.83399	-0.04919	0.007598	9.54E-11	27863252	European	chr6:31392047
0.83399	-0.04713	0.00759	5.31E-10	27863252	European	chr6:31392047
0.83399	-0.07189	0.007589	2.71E-21	27863252	European	chr6:31392047
0.82644	-0.04361	0.007452	4.83E-09	27863252	European	chr6:31392047

0.82644	-0.08523	0.00747	3.76E-30	27863252	European	chr6:31392047
0.82644	-0.04183	0.00747	2.16E-08	27863252	European	chr6:31392047
0.82644	-0.04338	0.007431	5.29E-09	27863252	European	chr6:31392047
0.82644	-0.04451	0.007446	2.27E-09	27863252	European	chr6:31392047
0.82644	-0.0426	0.007438	1.02E-08	27863252	European	chr6:31392047
0.82644	-0.06795	0.007434	6.17E-20	27863252	European	chr6:31392047
0.82644	0.3729	0.067	7.82E-09	27992413	European	chr6:31392047
0.82644	0.009622	0.001649	5.34E-09	UKBB	European	chr6:31392047
0.82644	0.01463	0.002175	1.77E-11	UKBB	European	chr6:31392047
0.82644	0.03356	0.003658	4.5E-20	UKBB	European	chr6:31392047
0.82644	-0.01754	0.00241	3.44E-13	UKBB	European	chr6:31392047
0.82644	0.00978	0.001652	3.22E-09	UKBB	European	chr6:31392047
0.82644	-0.00873	0.001099	2.06E-15	UKBB	European	chr6:31392047
0.82644	0.002614	0.000338	1.08E-14	UKBB	European	chr6:31392047
0.82644	-0.00063	0.000114	2.69E-08	UKBB	European	chr6:31392047
0.82644	0.03034	0.003979	2.42E-14	UKBB	European	chr6:31392047
0.82644	-0.00643	0.001013	2.19E-10	UKBB	European	chr6:31392047
0.82644	-0.04356	0.007468	5.45E-09	27863252	European	chr6:31392047
0.82644	-0.08642	0.007487	8.07E-31	27863252	European	chr6:31392047
0.82644	-0.04182	0.007487	2.33E-08	27863252	European	chr6:31392047
0.82644	-0.04324	0.007448	6.39E-09	27863252	European	chr6:31392047
0.82644	-0.0444	0.007463	2.69E-09	27863252	European	chr6:31392047
0.82644	-0.04251	0.007454	1.18E-08	27863252	European	chr6:31392047
0.82644	-0.06849	0.007451	3.83E-20	27863252	European	chr6:31392047
0.82644	0.3729	0.067	7.75E-09	27992413	European	chr6:31392047
0.82644	0.009624	0.001649	5.31E-09	UKBB	European	chr6:31392047
0.82644	0.01462	0.002175	1.81E-11	UKBB	European	chr6:31392047
0.82644	0.03357	0.003658	4.47E-20	UKBB	European	chr6:31392047
0.82644	-0.01753	0.00241	3.49E-13	UKBB	European	chr6:31392047
0.82644	0.009783	0.001652	3.2E-09	UKBB	European	chr6:31392047
0.82644	-0.00873	0.001099	2.04E-15	UKBB	European	chr6:31392047
0.82644	0.002614	0.000338	1.08E-14	UKBB	European	chr6:31392047
0.82644	-0.00063	0.000114	2.71E-08	UKBB	European	chr6:31392047
0.82644	0.03035	0.003979	2.39E-14	UKBB	European	chr6:31392047
0.82644	-0.00643	0.001013	2.14E-10	UKBB	European	chr6:31392047
0.82345	-0.1003	0.008277	9.04E-34	27863252	European	chr6:31392047
0.82345	-0.04884	0.008191	2.49E-09	27863252	European	chr6:31392047
0.82345	-0.07036	0.008238	1.32E-17	27863252	European	chr6:31392047
0.82345	0.4048	0.074	1.42E-08	27992413	European	chr6:31392047
0.82345	0.0122	0.001804	1.38E-11	UKBB	European	chr6:31392047
0.82345	0.058	0.01028	1.69E-08	UKBB	European	chr6:31392047
0.82345	0.02	0.002381	4.49E-17	UKBB	European	chr6:31392047
0.82345	0.0393	0.004003	9.55E-23	UKBB	European	chr6:31392047
0.82345	-0.0218	0.002638	1.4E-16	UKBB	European	chr6:31392047
0.82345	0.01237	0.001808	7.76E-12	UKBB	European	chr6:31392047
0.82345	-0.00976	0.001203	4.87E-16	UKBB	European	chr6:31392047
0.82345	0.002921	0.00037	2.96E-15	UKBB	European	chr6:31392047
0.82345	0.03624	0.004355	8.61E-17	UKBB	European	chr6:31392047
0.82345	-0.00703	0.001109	2.29E-10	UKBB	European	chr6:31392047
0.82345	-0.1002	0.008243	5.02E-34	27863252	European	chr6:31392047
0.82345	-0.04767	0.008158	5.11E-09	27863252	European	chr6:31392047
0.82345	0.04525	0.008165	2.99E-08	27863252	European	chr6:31392047
0.82345	-0.07115	0.008203	4.18E-18	27863252	European	chr6:31392047
0.82345	0.4415	0.075	1.06E-09	27992413	European	chr6:31392047
0.82345	0.01206	0.001782	1.31E-11	UKBB	European	chr6:31392047
0.82345	0.05594	0.01014	3.52E-08	UKBB	European	chr6:31392047
0.82345	0.0195	0.002351	1.09E-16	UKBB	European	chr6:31392047
0.82345	0.03799	0.003953	7.33E-22	UKBB	European	chr6:31392047

0.82345	-0.02134	0.002605	2.59E-16	UKBB	European	chr6:31392047
0.82345	0.01224	0.001786	7.18E-12	UKBB	European	chr6:31392047
0.82345	-0.00959	0.001188	6.95E-16	UKBB	European	chr6:31392047
0.82345	0.002865	0.000366	4.55E-15	UKBB	European	chr6:31392047
0.82345	0.03483	0.004301	5.54E-16	UKBB	European	chr6:31392047
0.82345	-0.00697	0.001095	1.93E-10	UKBB	European	chr6:31392047
0.81187	-0.1005	0.008322	1.39E-33	27863252	European	chr6:31392047
0.81187	-0.048	0.008236	5.6E-09	27863252	European	chr6:31392047
0.81187	0.04578	0.008244	0.000000028	27863252	European	chr6:31392047
0.81187	-0.07155	0.008282	5.64E-18	27863252	European	chr6:31392047
0.81187	0.4383	0.078	5.94E-09	27992413	European	chr6:31392047
0.81187	0.01218	0.001803	1.43E-11	UKBB	European	chr6:31392047
0.81187	0.05802	0.01027	1.64E-08	UKBB	European	chr6:31392047
0.81187	0.02002	0.002379	3.91E-17	UKBB	European	chr6:31392047
0.81187	0.03931	0.004001	8.78E-23	UKBB	European	chr6:31392047
0.81187	-0.02181	0.002636	1.31E-16	UKBB	European	chr6:31392047
0.81187	0.01236	0.001807	8.05E-12	UKBB	European	chr6:31392047
0.81187	-0.00975	0.001202	4.94E-16	UKBB	European	chr6:31392047
0.81187	0.00292	0.00037	2.91E-15	UKBB	European	chr6:31392047
0.81187	0.03624	0.004352	8.37E-17	UKBB	European	chr6:31392047
0.81187	-0.00702	0.001108	2.35E-10	UKBB	European	chr6:31392047
0.81164	-0.101	0.00833	7.62E-34	27863252	European	chr6:31392047
0.81164	-0.04726	0.008243	9.88E-09	27863252	European	chr6:31392047
0.81164	0.04633	0.00825	1.95E-08	27863252	European	chr6:31392047
0.81164	-0.07318	0.00829	1.07E-18	27863252	European	chr6:31392047
0.81164	0.4453	0.076	1.28E-09	27992413	European	chr6:31392047
0.81164	0.01219	0.001788	9.27E-12	UKBB	European	chr6:31392047
0.81164	0.01976	0.002359	5.54E-17	UKBB	European	chr6:31392047
0.81164	0.03839	0.003967	3.78E-22	UKBB	European	chr6:31392047
0.81164	-0.02169	0.002614	1.06E-16	UKBB	European	chr6:31392047
0.81164	0.01239	0.001792	4.77E-12	UKBB	European	chr6:31392047
0.81164	-0.00971	0.001192	3.69E-16	UKBB	European	chr6:31392047
0.81164	0.00286	0.000367	6.32E-15	UKBB	European	chr6:31392047
0.81164	0.03566	0.004315	1.41E-16	UKBB	European	chr6:31392047
0.81164	-0.00701	0.001099	1.79E-10	UKBB	European	chr6:31392047
0.80013	-0.09751	0.008194	1.17E-32	27863252	European	chr6:31392047
0.80013	-0.04612	0.008109	1.29E-08	27863252	European	chr6:31392047
0.80013	0.04502	0.008116	0.000000029	27863252	European	chr6:31392047
0.80013	-0.0701	0.008155	8.26E-18	27863252	European	chr6:31392047
0.80013	0.4806	0.074	1.88E-11	27992413	European	chr6:31392047
0.80013	0.0117	0.001764	3.32E-11	UKBB	European	chr6:31392047
0.80013	0.01925	0.002328	1.34E-16	UKBB	European	chr6:31392047
0.80013	0.03697	0.003915	3.61E-21	UKBB	European	chr6:31392047
0.80013	-0.02113	0.00258	2.61E-16	UKBB	European	chr6:31392047
0.80013	0.01193	0.001768	1.52E-11	UKBB	European	chr6:31392047
0.80013	-0.00908	0.001176	1.21E-14	UKBB	European	chr6:31392047
0.80013	0.00287	0.000362	2.17E-15	UKBB	European	chr6:31392047
0.80013	0.03447	0.004258	5.79E-16	UKBB	European	chr6:31392047
0.80013	-0.00654	0.001084	1.59E-09	UKBB	European	chr6:31392047
0.80008	-0.09984	0.008253	1.09E-33	27863252	European	chr6:31392047
0.80008	-0.04748	0.008167	6.09E-09	27863252	European	chr6:31392047
0.80008	0.04626	0.008175	1.53E-08	27863252	European	chr6:31392047
0.80008	-0.07119	0.008213	4.38E-18	27863252	European	chr6:31392047
0.80008	0.5074	0.053	1.06E-22	27992413	European	chr6:31392047
0.82345	-1.5	0.2232	1.81E-11	27863251	European	chr6:31392047
0.82345	-1.5	0.2232	1.81E-11	27863251	European	chr6:31392047
0.81187	-1.498	0.2287	5.78E-11	27863251	European	chr6:31392047
0.80013	-1.464	0.2186	2.14E-11	27863251	European	chr6:31392047

0.82345	-1.281	0.2273	1.73E-08	27863251	European	chr6:31392047
0.82345	-1.281	0.2273	1.73E-08	27863251	European	chr6:31392047
0.80013	-1.252	0.2227	1.88E-08	27863251	European	chr6:31392047
1	-1.159	0.2109	3.91E-08	27863251	European	chr6:31392047
0.98806	-1.159	0.2109	3.91E-08	27863251	European	chr6:31392047
1	1.49	0.1776	4.806E-17	27863251	European	chr6:31392047
1	1.391	0.1829	2.85E-14	27863251	European	chr6:31392047
1	-1.365	0.1816	5.575E-14	27863251	European	chr6:31392047
1	1.384	0.1816	2.533E-14	27863251	European	chr6:31392047
1	-1.12	0.1855	1.575E-09	27863251	European	chr6:31392047
1	-1.056	0.1883	2.036E-08	27863251	European	chr6:31392047
1	1.029	0.1867	3.562E-08	27863251	European	chr6:31392047
1	-1.167	0.1858	3.346E-10	27863251	European	chr6:31392047
1	1.269	0.1836	4.801E-12	27863251	European	chr6:31392047
1	-1.031	0.1869	3.44E-08	27863251	European	chr6:31392047
1	-1.158	0.1856	4.442E-10	27863251	European	chr6:31392047
1	-1.134	0.1865	1.187E-09	27863251	European	chr6:31392047
1	-1.171	0.1847	2.322E-10	27863251	European	chr6:31392047
1	-0.5405	0.08898	1.961E-09	27036880	European	chr6:31392047
1	-0.5186	0.08694	3.76E-09	27036880	European	chr6:31392047
1	-0.4899	0.08659	2.174E-08	27036880	European	chr6:31392047
1	-0.5954	0.08912	4.363E-11	27036880	European	chr6:31392047
0.98806	1.49	0.1776	4.806E-17	27863251	European	chr6:31392047
0.98806	1.391	0.1829	2.85E-14	27863251	European	chr6:31392047
0.98806	-1.365	0.1816	5.575E-14	27863251	European	chr6:31392047
0.98806	1.384	0.1816	2.533E-14	27863251	European	chr6:31392047
0.98806	-1.12	0.1855	1.575E-09	27863251	European	chr6:31392047
0.98806	-1.056	0.1883	2.036E-08	27863251	European	chr6:31392047
0.98806	1.029	0.1867	3.562E-08	27863251	European	chr6:31392047
0.98806	-1.167	0.1858	3.346E-10	27863251	European	chr6:31392047
0.98806	1.269	0.1836	4.801E-12	27863251	European	chr6:31392047
0.98806	-1.031	0.1869	3.44E-08	27863251	European	chr6:31392047
0.98806	-1.158	0.1856	4.442E-10	27863251	European	chr6:31392047
0.98806	-1.134	0.1865	1.187E-09	27863251	European	chr6:31392047
0.98806	-1.171	0.1847	2.322E-10	27863251	European	chr6:31392047
0.98806	-0.5405	0.08898	1.961E-09	27036880	European	chr6:31392047
0.98806	-0.5186	0.08694	3.76E-09	27036880	European	chr6:31392047
0.98806	-0.4899	0.08659	2.174E-08	27036880	European	chr6:31392047
0.98806	-0.5954	0.08912	4.363E-11	27036880	European	chr6:31392047
0.82644	1.466	0.1798	3.517E-16	27863251	European	chr6:31392047
0.82644	1.393	0.1845	4.337E-14	27863251	European	chr6:31392047
0.82644	-1.213	0.186	7.008E-11	27863251	European	chr6:31392047
0.82644	1.243	0.186	2.327E-11	27863251	European	chr6:31392047
0.82644	-1.039	0.1892	4.005E-08	27863251	European	chr6:31392047
0.82644	1.183	0.1867	2.335E-10	27863251	European	chr6:31392047
0.82644	-0.485	0.08683	3.249E-08	27036880	European	chr6:31392047
0.82644	-0.4689	0.08479	4.273E-08	27036880	European	chr6:31392047
0.82644	1.466	0.1798	3.517E-16	27863251	European	chr6:31392047
0.82644	1.393	0.1845	4.337E-14	27863251	European	chr6:31392047
0.82644	-1.213	0.186	7.008E-11	27863251	European	chr6:31392047
0.82644	1.243	0.186	2.327E-11	27863251	European	chr6:31392047
0.82644	-1.039	0.1892	4.005E-08	27863251	European	chr6:31392047
0.82644	1.183	0.1867	2.335E-10	27863251	European	chr6:31392047
0.82644	-0.485	0.08683	3.249E-08	27036880	European	chr6:31392047
0.82345	1.529	0.1966	7.366E-15	27863251	European	chr6:31392047
0.82345	1.372	0.2043	1.862E-11	27863251	European	chr6:31392047
0.82345	-1.358	0.2012	1.468E-11	27863251	European	chr6:31392047
0.82345	1.325	0.2024	5.861E-11	27863251	European	chr6:31392047

0.82345	-1.304	0.2016	9.983E-11	27863251	European	chr6:31392047
0.82345	-1.169	0.2051	1.206E-08	27863251	European	chr6:31392047
0.82345	-1.19	0.2046	5.998E-09	27863251	European	chr6:31392047
0.82345	1.244	0.2034	9.614E-10	27863251	European	chr6:31392047
0.82345	-1.362	0.2014	1.354E-11	27863251	European	chr6:31392047
0.82345	-1.124	0.2047	3.996E-08	27863251	European	chr6:31392047
0.82345	1.418	0.2495	1.319E-08	27863251	European	chr6:31392047
0.82345	-1.366	0.2496	4.428E-08	27863251	European	chr6:31392047
0.82345	-0.5727	0.09388	1.624E-09	27036880	European	chr6:31392047
0.82345	-0.5987	0.093	2.141E-10	27036880	European	chr6:31392047
0.82345	-0.5864	0.09078	1.863E-10	27036880	European	chr6:31392047
0.82345	-0.5107	0.09077	2.594E-08	27036880	European	chr6:31392047
0.82345	-0.6179	0.1025	2.585E-09	27036880	European	chr6:31392047
0.82345	-0.7062	0.09496	2.559E-13	27036880	European	chr6:31392047
0.82345	-0.5159	0.08927	1.104E-08	27036880	European	chr6:31392047
0.82345	1.529	0.1966	7.366E-15	27863251	European	chr6:31392047
0.82345	1.372	0.2043	1.862E-11	27863251	European	chr6:31392047
0.82345	-1.358	0.2012	1.468E-11	27863251	European	chr6:31392047
0.82345	1.325	0.2024	5.861E-11	27863251	European	chr6:31392047
0.82345	-1.304	0.2016	9.983E-11	27863251	European	chr6:31392047
0.82345	-1.169	0.2051	1.206E-08	27863251	European	chr6:31392047
0.82345	-1.19	0.2046	5.998E-09	27863251	European	chr6:31392047
0.82345	1.244	0.2034	9.614E-10	27863251	European	chr6:31392047
0.82345	-1.362	0.2014	1.354E-11	27863251	European	chr6:31392047
0.82345	-1.124	0.2047	3.996E-08	27863251	European	chr6:31392047
0.82345	1.418	0.2495	1.319E-08	27863251	European	chr6:31392047
0.82345	-1.366	0.2496	4.428E-08	27863251	European	chr6:31392047
0.82345	-0.574	0.09316	1.117E-09	27036880	European	chr6:31392047
0.82345	-0.6223	0.09374	6.003E-11	27036880	European	chr6:31392047
0.82345	-0.5929	0.09164	1.761E-10	27036880	European	chr6:31392047
0.82345	-0.5237	0.09158	1.545E-08	27036880	European	chr6:31392047
0.82345	-0.5986	0.1011	4.801E-09	27036880	European	chr6:31392047
0.82345	-0.6681	0.09452	3.326E-12	27036880	European	chr6:31392047
0.82345	-0.543	0.08995	2.489E-09	27036880	European	chr6:31392047
0.81187	1.54	0.2003	1.492E-14	27863251	European	chr6:31392047
0.81187	1.387	0.2081	2.673E-11	27863251	European	chr6:31392047
0.81187	-1.338	0.2055	7.458E-11	27863251	European	chr6:31392047
0.81187	1.308	0.2066	2.414E-10	27863251	European	chr6:31392047
0.81187	-1.365	0.2044	2.402E-11	27863251	European	chr6:31392047
0.81187	-1.181	0.2086	1.51E-08	27863251	European	chr6:31392047
0.81187	1.259	0.207	1.192E-09	27863251	European	chr6:31392047
0.81187	-1.371	0.2053	2.396E-11	27863251	European	chr6:31392047
0.81187	-0.573	0.09487	2.322E-09	27036880	European	chr6:31392047
0.81187	-0.6173	0.09378	8.608E-11	27036880	European	chr6:31392047
0.81187	-0.5957	0.09162	1.434E-10	27036880	European	chr6:31392047
0.81187	-0.5256	0.09157	1.368E-08	27036880	European	chr6:31392047
0.81187	-0.5958	0.1041	1.476E-08	27036880	European	chr6:31392047
0.81187	-0.6996	0.09609	7.731E-13	27036880	European	chr6:31392047
0.81187	-0.5056	0.09024	2.976E-08	27036880	European	chr6:31392047
0.81164	-0.57	0.09416	2.142E-09	27036880	European	chr6:31392047
0.81164	-0.6415	0.09455	2.334E-11	27036880	European	chr6:31392047
0.81164	-0.6054	0.09249	1.083E-10	27036880	European	chr6:31392047
0.81164	-0.5453	0.09236	5.325E-09	27036880	European	chr6:31392047
0.81164	-0.6026	0.103	7.31E-09	27036880	European	chr6:31392047
0.81164	-0.6914	0.09538	9.558E-13	27036880	European	chr6:31392047
0.81164	-0.5567	0.09076	1.393E-09	27036880	European	chr6:31392047
0.80013	1.498	0.1934	9.431E-15	27863251	European	chr6:31392047
0.80013	1.348	0.2008	1.902E-11	27863251	European	chr6:31392047

0.80013	-1.333	0.1979	1.632E-11	27863251	European	chr6:31392047
0.80013	1.259	0.1997	2.919E-10	27863251	European	chr6:31392047
0.80013	-1.339	0.1973	1.141E-11	27863251	European	chr6:31392047
0.80013	-1.135	0.2021	1.951E-08	27863251	European	chr6:31392047
0.80013	-1.151	0.2015	1.113E-08	27863251	European	chr6:31392047
0.80013	1.17	0.2009	5.751E-09	27863251	European	chr6:31392047
0.80013	-1.335	0.1982	1.615E-11	27863251	European	chr6:31392047
0.80013	-1.18	0.2001	3.733E-09	27863251	European	chr6:31392047
0.80013	-0.5357	0.09159	7.073E-09	27036880	European	chr6:31392047
0.80013	-0.5957	0.09303	2.657E-10	27036880	European	chr6:31392047
0.80013	-0.5896	0.09075	1.479E-10	27036880	European	chr6:31392047
0.80013	-0.5417	0.09054	3.367E-09	27036880	European	chr6:31392047
0.80013	-0.5791	0.09937	8.253E-09	27036880	European	chr6:31392047
0.80013	-0.6412	0.09275	9.484E-12	27036880	European	chr6:31392047
0.80013	-0.5154	0.08927	1.143E-08	27036880	European	chr6:31392047
0.80008	0.3161	0.0574	4.984E-08	27036880	European	chr6:31392047
0.80008	0.3759	0.06627	1.994E-08	27036880	European	chr6:31392047
0.80008	-0.3859	0.06697	1.207E-08	27036880	European	chr6:31392047
0.80008	-0.4426	0.06286	4.238E-12	27036880	European	chr6:31392047
0.80008	0.4081	0.06392	2.982E-10	27036880	European	chr6:31392047
0.80008	-0.3352	0.05682	5.326E-09	27036880	European	chr6:31392047
0.80008	-0.3376	0.06085	3.896E-08	27036880	European	chr6:31392047
0.80008	0.3628	0.06043	2.867E-09	27036880	European	chr6:31392047
0.80008	-0.3415	0.0597	1.492E-08	27036880	European	chr6:31392047

Supplementary Table 10

ID	Name	CAS	MolWt	SMI	Pathways	Target	Bioactivity	GBSA dG
T21661L	[23042 Ac	T21661L	1252.5	=O)N[C@(	Others	Others	/al-Cys-Na	-78.6241
TN1124	Veoeriocitri	13241-32-2	596.54	C[C@@H](	Neuroscienc	AChE	and osteog	-77.6935
T22928L	tachykinin]	T22928L	938.04	H](NC(=O)	Others	Others	achykinin I	-74.0751
TP1280	Hexa-His	64134-30-1	840.85	[C(=O)[C@	Others	Others	;. Hexa His	-73.9474
TP1852L	tate(11057	TP1852L	906.17	O)C(=O)N	rter/Ion ch	assium Cha	ntain myo	-73.9124
T7683	ine amine c	96760-69-9	576.6	[C@H]5[C	Protein; Ne	nosine Rec	is an agonis	-71.075
TN1096	nsenoside R	83459-41-0	1211.38	)[C@@H](	e Kinase/A	rosine Kina	itory effec	-70.1928
T19368	[ypromellos	9004-65-3	1261.44	](O[C@H]2	Others	Others	ole hydroph	-68.258
TP1086	TFA(13530	TP1086	1195.25	cc2)[C@@	Protein; Ne	rokinin Rec	tagonist wi	-67.7458
TP1910L1	ALP1 aceta	TP1910L1	902.13	C@@H](/N	n channel;N	m Channel;	iting the op	-67.6467
TP1033L	Peptide Dis	TP1033L	1121.2	(CO)C(=O)	transporter/	ATPase	1. Transder	-67.5533
TP1346	N 154806 T	828432-46-	1311.36	cc4)C(=O)	PCR/G Prot	omatostati	the recomb	-67.2396
T7625	TRAP-6	141136-83-(	748.87	H](NC(=O	PCR/G Prot	-activated I	of protease	-66.7864
T7513	ivated Rece	90383-13-2	614.78	[3+])[C@H	PCR/G Prot	-activated I	mide is an?:	-66.2383
T7616L	159432-28-	T7616L	987.01	)H](NC(=C	inology/Ho	RAAS	tagonist of	-64.9477
T8488	GMB-475	490599-18-	861.93	C)C(=O)N4	aling; Tyro	Abl; PROT	CR-ABL1 p	-64.5848
T8641	Vancomycin	1404-90-6	1449.25	)@H](C(=C	Microbiold	; Antibiotic	tively inhil	-64.0783
T1522	Iopromide	73334-07-3	791.12	@H](O)CC	Others	Others	n-ionic cont	-64.0179
T67834	C004019	417159-57-	1035.29	[C@H](O)C	Cytoskeleta	Microtubule	E3 ligase (V	-63.786
T7769	onapeptide	58563-45-2	1206.5	C(=O)N2[C	Others	Others	which mak	-63.6104
T22933L	ILS-NH2 ac	T22933L	656.82	C(=O)[C@(	PCR/G Prot	-activated I	a PAR-2-ir	-63.1584
T1245	olistin sulfa	1264-72-8	1253.51	N[C@@H]	Inflammati	ibiotic; Au	Bacillus c	-62.7123
T7675	2TFA(6806	T7675	1128.08	@H]2N(C(=	Others	Others	degradatio	-62.1657
TP1289	alog (CTX)	918768-05-	1013.16	(=O)N[C@]	Others	Others	toxin analo	-61.6496
TN1028	Militarine	58139-23-4	726.73	](O)(CC(=(	Others	Others	t the growtl	-61.5502
TP1341L	B nonapep	TP1341L	1023.19	N[C@@H](	biology/vir	cterial; An	ed with poly	-60.7047
T1799	ofungin Ac	79463-17-3	1213.42	H](O)CC[N	biology/vir	iotic; Antif	fermentati	-60.6118
T21498	Phleomycin	11006-33-0	1268.29	(=[NH2+])	amage/DNA	ibiotic; DN	is similar t	-60.0647
TN1957	ulberrofuran	101383-35-1	592.55	c6)(Oc6c(c	Others	Others	nboxane B2	-59.3983
TP1805	7 Tag Pepti	245445-88-9	1098.28	(=O)NCC(=	Others	Others	ag antibodi	-59.0379
TP1913L1	(183241-31	TP1913L1	1193.35	O)[C@@H	PCR/G Prot	besin Rece	y). Enhanc	-58.8824
TP1367	]leucine-en	64963-01-5	569.65	C@H](NC(=	es; GPCR/G	ioid Recep	st used to s	-58.6426
T15805L	ate(108871	T15805L	1250.49	H2+]C(C)C	rotein; Imn	CXCR	st. It preve	-58.2914
T7434	Argipressin	113-79-1	1084.23	O)N)NC(=(	PCR/G Prot	pressin Rec	sin acts as a	-58.0605
T22446	DP disodiun	24292-60-2	787.37	N)c3nc2)O1	Others	Others	er in the me	-57.6557
TP1192L	etate(11131	TP1192L	1088.32	2+])N)NC(	PCR/G Prot	pressin Rec	shows high	-57.5376
TP1419L	ate(926018	TP1419L	821.84	)[C@@H](	PCR/G Prot	cagon Rece	N-terminal	-57.1402
T1104	nomycin Su	1263-89-4	713.71	3+])[C@@	biology/vir	al; Antibiot	by cell deat	-57.0057
T12882L	etate(9200	759937-80-	1177.35	C(=[NH2+])	Protein; Ne	ocortin Re	(MC4R)(hu	-56.8932
T7598	-methyltetr	26560-38-3	497.51	I]2N(C)C=	Cycle/Check	Antifolate	oposed for t	-56.5279
TP1941L1	RWK-NH2	TP1941L1	1072.22	NC(=O)[C@	es; GPCR/G	ioid Recep	ociceptin re	-56.2351
T20655	yquaterniur	75345-27-6	556.99	/C[N+](CC	biology/vir	Antibacteria	c preservat	-55.4908
TP2325L	kin Inhibito	TP2325L	1284.65	CC[NH3+]	Others	Others	nces with d	-55.345
TN7089	Alashinol G	58139-12-1	378.42	C@](O)(CO	Others	Others	folia, a Mo	-54.9662
T4S1616	Sanggenon I	81422-93-7	708.71	))(Oc3cc2O	ane transp	c ;GABA	acterial act	-54.9039
T7766	Remdesivir	809249-37-	602.58	c2n3N=CN	amage/DNA	Synthesis;	ndent RNA	-54.8251
T40555L	yP-1 acetat	T40555L	1052.19	@H](O)C)(	Others	Others	ing to p32 i	-54.4265
T14589	tin-PEG3-a	252881-76-8	447.55	CCCC[C@(	PROTAC	OTAC Lin	l group. Th	-54.282
T7633	Senktide	106128-89-(	842	(=O)[C@H	Protein; Ne	rokinin Rec	ist of tachy	-54.1251
TN1013	ucurbitacin	3877-86-9	516.67	C@]1(C)[C(	Apoptosis	Apoptosis	cin D, a no	-54.0551
TQ0269	Melittoside	19467-03-9	524.47	]2(O[C@H	ation-reduc	Antioxidant	luct and ma	-53.9909
TP1212	he-Leu-Phe	48182-34-1	785.97	C@H](C(=(	Others	Others	ain effects	-53.9595
TN2079	)-Pinoresin	81446-29-9	358.39	[]3[C@H](c	Others	Others	whole plant	-53.93

T4S1521	:affeoylquir	1182-34-9	516.45	/C=C/c2cc(	ology; Prot	HIV Protea	stive class o	-53.6204
T38063	:yltyrosylcy	287585-40-	453.56	@H](NC(=	Metabolism	hione Pero	ogical funct	-53.5732
T9501	Enterobacti	28384-96-5	669.55	(=O)OC[C(	Others	Others	ophore that	-53.4513
T7624	DAPTA	106362-34-	856.88	ccc(O)cc1)(	y/virology;	R; HIV Prot	of CCR5, sh	-53.3959
TP2502	VSVQDI A	TP2502	1008.13	O)CNC(=O	Others	Others	HC class I-r	-52.7947
T4589	thyl salvian	167424-32-	732.7	[C@@H](C	Others	Others		-52.1667
T16340	Nosiheptide	56377-79-8	1222.36	C@@H]6c	biology/vir	cterial; Ant	osiheptide i	-51.426
TP1286	ıbryonic An	68635-85-(	964.07	[C@@H](N	Others	Others	hat is prese	-50.7991
T7627L	: Hormone (	T7627L	1076.12	)H](C(=O)N	Protein; Ne	ocortin Re	) is an agor	-50.6932
T5772	tofuranosyl	59432-60-9	828.72	@H](O)[C@	Others	Others	ch are natur	-50.5721
T7495	:xa-D-argin	73202-67-(	954.14	NC(=[NH2-	Others	Others	ıe activatio	-49.5125
T1470L	adine hydro	38452-21-8	538.12	C[NH+]2C	ology/Infla	mine Rece	ne and long	-49.4668
TP1889	MCL0020	475498-26-	609.73	NC(=O)[C	Protein; Ne	ocortin Re	respectivel	-49.4189
T7484L	FA(187986-	T7484L	1084.16	[]c1)cccc2)(	Others	Others	ormyl pepti	-49.3797
T40233	[so)-MS432	601727-80-	1101.32	O)C1)COC(	Epigenetic	yltransfera	anticancer	-48.5035
TP1378	ide TFA(7	TP1378	891.98	C)C)CCCN	CR/G Prot	-activated	elective pro	-48.4234
TP1328	TB500	885340-08-9	889.01	[]](NC(=O)[	Others	Others	rt-chain ve	-48.3546
T13589	aesappanin	913319-59-	608.59	@](O)(CO)C	Others	Others	., shows str	-48.3061
T17987	-Gly-PEG4	353409-80-	694.77	CNC(=O)C(	Conjugate	ADC Linke	f PEG, whi	-48.1416
T7601L	betocin ace	631754-28-	1048.22	@H](CCC(=	Hormones;	tocin Rece	ntrol postp	-48.012
T65172	denine dinu	146-14-5	785.55	O-])(OC[C@	Metabolism	enous Met	or, a protei	-47.8945
T36798	SW2_110A	579696-95-	760.96	)[C@H](N	matin/Epige	Methyltran	r with a K	-47.8815
TP2373L	tripeptide-	TP2373L	626.72	C[C@@H]1	Others	Others	promoting s	-47.8733
T68151L	llimustine H	18438-45-2	734.08	ı(C)c(C(=O	amage/DNA	DNA	d in severe	-47.7463
TP1085L	cetate(9216	TP1085L	805.9	](NC(=O)[	Protein; Ne	amine Rece	distinct ex	-47.4834
TP1273	LAG peptic	98849-88-8	1012.97	C@@H](NC	Others	Others	)-Lys) with	-46.832
T76373L	ı-alloresact	T76373L	1106.28	H](NC(=O)[	orter/Ion ch	lcium Chan	released by	-46.7593
TP1554L	E7 (86-93)	TP1554L	875.08	])[C@H](O	Others	Others	gen A2.1 re	-46.3252
T7423	fosol tetras	211427-08-(	878.23	[O-])[O-])(	Protein; Ne	2Y Recept	ues Diquaf	-46.2364
T8924	CD289873	610413-97-	678.81	N[C@H](C(	Others	Others	an be used	-46.1386
T1626	ostaglandin	745-65-3	354.48	H](/C=C/[C	nology/Inf	bolite; Pros	s bronchodi	-45.9638
T0978	Pantethine	16816-67-4	554.72	O)(C)C)CC	Metabolism	s Metaboli	t, so most vi	-45.7296
T15711	azotide ace	881851-50-9	785.89	H](NC(=O	keletal Sig	Junction Pr	nction regu	-45.5209
TP1940L1	YRIK-NH2	TP1940L1	999.17	NC(=O)[C	es; GPCR/	oioid Rece	partial agoni	-45.4391
T21249L	entapeptide	T21249L	856.19	NH3+])C)[C	Others	Others	moting the	-45.4108
T20080	Exifone	52479-85-3	278.21	c(O)cc1)c1	tic; DNA D	HDAC	substrate-b	-45.0857
TP1197	Sincalide	25126-32-3	1143.27	(=O)N[C@	Others	Others	e gallbladd	-45.0757
TP2496	peptide-2 A	TP2496	1005.06	@@H](NC(	Others	Others	nd migratio	-44.7841
T21459	opentin dia	105184-37-(	753.84	C@H]([NH	Others	Others	n reproduc	-44.5174
TP1687L	e(153045-2	TP1687L	1003.22	)C(C)C)CC	Others	Others	.-A*0201-re	-44.4793
T7497L	FA(63555-6	T7497L	1052	@@H](NC(	ology/Infla	nplement Sy	correspond	-44.3961
TP1618L	8), human,	TP1618L	1122.32	](NC(=O)[	Others	Others	cacious, an	-44.36
T11428	-Obetichol	863239-60-5	477.68	H]3[C@H](	Metabolism	FXR	c acid. Obe	-44.0661
T16413	OTX008	286936-40-	937.18	:1)Cc1c(O	ology/Infla	Galectin	lective gale	-44.0363
T8299	yl lithospe	875313-64-	746.7	[C@@H](	ransporter/	dium Chan	3 is a select	-43.7408
T1067	nphotericin	1397-89-3	924.08	\C=C/C=C\	biology/vir	iotic; Antif	ies. Ampho	-43.6555
T7743	/ TFA(126	T7743	888.92	H](NC(=O)[	inology/Ho	RAAS	fective ago	-43.6338
T3786	Tomatine	17406-45-0	1034.2	C4)CC3)C	es/Proteaso	tosis; Prote	nt of breast	-43.6094
T1013	Amikacin	37517-28-5	585.6	](O[C@@H	biology/vir	ribosome	um antibiot	-43.2393
T22439	icosin phos	137330-13-3	967.1	)O2)[C@@	biology/vir	cterial; Ant	o treat cattl	-43.0635
T78181	MMP-7-IN-	848717-49-	729.16	(=O)N[C@	ases/Protea	Others	can be use	-43.0116
T7554	stance P (7	51165-05-0	612.78	(NC(=O)[C	Protein; Ne	rokinin Rec	a C-termina	-42.9667
T6S0743	Sesamoside	117479-87-5	420.36	2[C@H](O[	Others	Others	s antinocic	-42.8908
TQ0208	SAH	979-92-0	384.41	1[C@@H]	Metabolism	enous Met	cysteine. S	-42.3571
TP1605L1	FA(12333	TP1605L1	1083.13	)H](C(=O)N	Others	Others	nd in insec	-42.3347

T16083	Mizaglifloz	666843-10-3	564.67	](O)[C@@	GPCR/G Prot	SGLT	zin is used a	-42.1397
T60085	MM3122	574390-27-	665.76	CNC(=[NH2	crobiology/vir	SARS-CoV	MM3122 effec	-41.8626
T20735	xin dihydro	10049-83-9	441.39	nc1)Cc1c(C	Metabolism	genous Meta	n, improve	-41.5875
TP1739L	e(688805-40	TP1739L	1095.26	H](NC(=O)[	Others	Others	capsid prote	-40.9802
T12248	seothricin s	96736-11-7	1359.47	(=O)C[C@	obiology/vir	l; Antibiotio	osynthesis in	-40.9256
T6S0444	vianolic aci	96574-01-5	494.45	)C(=O)/C=	ases/Protea	MMP	se 9 (MMP-	-40.7642
T124010	Dieckol	88095-77-6	742.55	23)cc1O)c	lirology;oxi	cterial;Anti	protective e	-40.7585
T22540L	-1 (16-21),	T22540L	898.01	[C@@H](N	Others	Others	fragment,	-40.3539
T24178	Isatoribine	122970-40-5	316.29	O)[C@@H]	crobiology/	t;HCV Prot	with anti-h	-40.1139
TP1883	eu9,Nle10]-	137565-28-1	804	C@@H](N	Protein; Ne	rokinin Rec	contraction	-40.0679
T19093	caffeoyl-D-	419478-52-	858.71	)cc1)C(=O)	Others	Others	a that inhib	-40.0589
T15656	KGA-2727	666842-36-0	536.62	2O[C@H]2	GPCR/G Prot	SGLT	?The select	-40.0224

PAINS	Lipinski	Pfizer	GSK	logP
['-']	1	0	1	2.316867
9, 20, 22, 2	1	0	1	0.309898
['-']	1	0	1	-1.82604
['-']	1	0	1	-0.78043
['-']	1	0	1	1.293201
['-']	1	0	1	0.021877
['-']	1	0	1	1.086773
['-']	1	0	1	-0.26131
['-']	1	0	1	0.878244
['-']	1	0	1	1.571726
['-']	1	0	1	-2.62219
['-']	1	0	1	0.497526
['-']	1	0	1	-0.39398
['-']	1	0	1	0.107448
['-']	1	0	1	-0.93149
['-']	1	0	1	4.879642
['-']	1	0	1	0.192223
['-']	1	0	1	-1.69548
['-']	1	0	1	1.197773
['-']	1	0	1	1.698694
['-']	1	0	1	0.003478
['-']	1	0	1	0.251303
['-']	1	0	1	-1.63659
['-']	1	0	1	2.356198
['-']	1	0	1	0.93373
['-']	1	0	1	-0.81882
['-']	1	0	1	1.542751
['-']	1	0	1	-2.23782
['-']	0	0	1	4.077131
['-']	1	0	1	-1.88829
['-']	1	0	1	1.25382
['-']	1	0	1	0.392502
['-']	1	0	1	1.284393
['-']	1	0	1	-1.16033
['-']	1	0	1	-3.36106
['-']	1	0	1	-1.30053
['-']	1	0	1	-1.62649
['-']	1	0	1	-2.83022
['-']	1	0	1	-0.79519
['-']	1	0	1	-1.20712
['-']	1	0	1	-1.3537
['-']	0	0	1	-2.09793
['-']	1	0	1	0.643338
['-']	0	0	0	1.238833
['-']	1	0	1	3.995427
['-']	1	0	1	1.814291
['-']	1	0	1	-5.32967
['-']	0	0	1	0.276531
['-']	1	0	1	0.9233
['-']	0	0	1	1.430007
['-']	1	0	1	-2.14722
['-']	1	0	1	4.019019
['-']	0	0	0	1.229532

6, 7, 9, 11,	1	0	1	1.522129
['-']	0	0	1	-0.56364
5, 16, 18, 1	1	0	1	0.363089
['-']	1	0	1	-3.20469
['-']	1	0	1	-0.69656
, 9, 11, 13,	1	0	1	2.800117
['-']	1	0	1	1.08653
['-']	1	0	1	-0.84044
['-']	1	0	1	-0.45805
['-']	1	0	1	-4.02103
['-']	1	0	1	-8.12024
['-']	0	0	1	3.547644
['-']	1	0	1	1.632903
['-']	1	0	1	1.831027
['-']	1	0	1	1.227596
['-']	1	0	1	0.573998
['-']	1	0	1	-1.53605
9, 17, 15, 1	1	0	1	2.475041
['-']	1	0	1	0.408233
['-']	1	0	1	0.56349
['-']	1	0	1	-2.2251
['-']	1	0	1	3.719315
['-']	1	0	1	-0.53982
['-']	1	0	1	1.948077
['-']	1	0	1	1.656386
['-']	1	0	1	-5.70431
['-']	1	0	1	-1.51141
['-']	1	0	1	1.159936
['-']	1	0	1	-5.1937
['-']	1	0	1	2.634693
['-']	0	0	0	3.148209
['-']	1	0	1	-0.8691
['-']	1	0	1	-1.0664
['-']	1	0	1	-1.27789
['-']	1	0	1	3.205219
4, 6, 8, 10,	0	0	0	1.284052
['-']	1	0	1	-0.55789
['-']	1	0	1	-2.08298
['-']	1	0	1	-1.32405
['-']	1	0	1	0.142854
['-']	1	0	1	-1.87544
['-']	1	0	1	-3.23277
['-']	0	0	1	3.334264
['-']	1	0	1	3.514535
, 9, 11, 13,	1	0	1	3.020874
['-']	1	0	1	0.622134
['-']	1	0	1	1.522628
['-']	1	0	1	0.954793
['-']	1	0	1	-3.63449
['-']	1	0	1	3.871225
['-']	1	0	1	1.104677
['-']	1	0	1	1.650141
['-']	1	0	1	-1.27496
['-']	1	0	0	-1.50272
['-']	1	0	1	-0.18013

['-']	1	0	1	1.705513
['-']	1	0	1	0.485195
['-']	0	0	0	-0.50878
['-']	1	0	1	-1.13862
['-']	1	0	1	-3.11924
6, 7, 9, 11,	0	0	1	2.559782
['-']	1	0	1	2.177415
['-']	1	0	1	1.477101
['-']	0	0	0	-1.20924
['-']	1	0	1	1.020937
6, 7, 9, 11,	1	0	1	2.631092
['-']	1	0	1	1.056679

Supplementary Table 11

Application	Sample ID	Gender	Age
scRNA-seq	N1	Female	61
	N2	Male	67
	N3	Female	56
	N4	Female	58
	IPF1	Male	50
	IPF2	Male	48
	IPF3	Male	61
	IPF4	Male	66
Flow Cytometry	N1	Male	56
	N2	Male	51
	N3	Male	54
	N4	Male	47
	N5	Male	65
	IPF1	Male	54
	IPF2	Female	69
	IPF3	Male	69
	IPF4	Male	57
	IPF5	Male	70

Supplementary Table 12

Gene	Direction	Sequence
GAPDH	Forward	CGGAGTCAACGGATTTGGTCGTAT
	Reverse	AGCCTTCTCCATGGTGGTGAAGAC
LGALS7	Forward	AATTCGCGGCTTGGTTCCTC
	Reverse	CTTGCTCCTTGCTGTTGAAGAC
